# Genetic differences among ethnic groups

**DOI:** 10.1186/s12864-015-2328-0

**Published:** 2015-12-21

**Authors:** Tao Huang, Yang Shu, Yu-Dong Cai

**Affiliations:** College of Life Science, Shanghai University, Shanghai, 200444 P. R. China; Institute of Health Sciences, Shanghai Institutes for Biological Sciences, Chinese Academy of Sciences, Shanghai, 200031 P. R. China; Sate Key Laboratory of Biotherapy, Sichuan University, Sichuan, 610041 P. R. China

## Abstract

**Background:**

Many differences between different ethnic groups have been observed, such as skin color, eye color, height, susceptibility to some diseases, and response to certain drugs. However, the genetic bases of such differences have been under-investigated. Since the HapMap project, large-scale genotype data from Caucasian, African and Asian population samples have been available. The project found that these populations were located in different areas of the PCA (Principal Component Analysis) plot. However, as an unsupervised method, PCA does not measure the differences in each single nucleotide polymorphism (SNP) among populations.

**Results:**

We applied an advanced mutual information-based feature selection method to detect associations between SNP status and ethnic groups using the latest HapMap Phase 3 release version 3, which included more sub-populations. A total of 299 SNPs were identified, and they can accurately predicted the ethnicity of all HapMap populations. The 10-fold cross validation accuracy of the SMO (sequential minimal optimization) model on training dataset was 0.901, and the accuracy on independent test dataset was 0.895.

**Conclusions:**

In-depth functional analysis of these SNPs and their nearby genes revealed the genetic bases of skin and eye color differences among populations.

**Electronic supplementary material:**

The online version of this article (doi:10.1186/s12864-015-2328-0) contains supplementary material, which is available to authorized users.

## Background

A single nucleotide polymorphism (SNP) is defined as a single base change in a DNA sequence that occurs in a significant proportion (more than 1 %) of a large population. SNPs occur at a frequency that ranges from 1 in 1000 to 1 in 100 bases. Recently, the NCBI (National Center for Biotechnology Information) released the SNP-138 database, which contains more than 60 million SNP sites (ftp://ftp.ncbi.nlm.nih.gov/snp/). To our knowledge, over the millions of years of evolution, mutations have occurred occasionally and are maintained or lost by inheritance and natural selection. The more than 60 million SNPs are scattered throughout the entire genome, including −50 % on the coding region and the rest on the non-coding region [1]. Based on the change in amino acid sequence, SNPs in the CDS (coding sequence) region can be divided into 2 classes: synonymous SNPs whose variants do not change the protein sequence and non-synonymous SNPs that change the amino acid sequence [2]. Along with the rapid development of next-generation DNA sequencing technologies, hundreds of thousands of novel human SNPs could be discovered in the next several years [3]. In addition to sequencing technology, GWAS (Genome-Wide Association Study) has been applied to discover disease-related SNPs [4–6]. To the best of our knowledge, functional polymorphisms are used not only to develop useful genetic markers but also to facilitate the outcomes of personalized medicines [7]. In addition, understanding the role of SNPs has been important to understanding the molecular mechanisms of evolution because SNPs could be used as evolution markers [8].

Among humans, 99.9 % of the bases in the entire genome are remarkably similar; it is the remaining 0.1 % of the bases that makes a person unique [9]. Among this 0.1 % of bases, more than 90 % are SNPs [10]. Barbujani et al. estimated that −85 % of SNPs are common to all human populations and that only approximately 15 % of SNPs are population-specific [11]. However, among different populations, specific SNPs account for 15 % of all SNPs, and common SNPs account for 85 % of all SNPs; both types contribute to various characteristics, including drug resistance and skin color [12, 13]. For example, Xu et al. found that the incidence of G6PD deficiency varies among populations because of the different proportions of SNP alleles [14]. Similarly, β-thalassemia exhibits a varied incidence among populations from Delhi (India), Lebanon and Sardinia because of the different predominant alleles in these areas [15–17]. In addition to susceptibility to diseases, physical appearance based on skin/hair color and physique varies among populations, especially those traits observed on different continents [12, 18]. The efforts of several groups have led to the identification of a series of SNPs and their corresponding genes, which may influence human pigmentation phenotypes; these include rs885479 at MC1R, rs16891982 at SLC45A2, rs1545397 at OCA2, rs12913832 at HERC2, rs6119471 at ASIP, and rs1426654 at SLC24A5. [19–24]. Although many pivotal SNPs have been discovered, they are far less important to explaining the differences among populations, such as the differences in physical appearance, disease susceptibility [25], and drug responses [26]. The studies performed in developed Caucasian countries may not apply well to developing African and Asian countries [27].

To systemically investigate the genetic differences among ethnic groups, we analyzed the latest HapMap [28] genotype data, which included more ethnic groups than the early releases and allowed us to explore the structure of the data in more detail. Advanced feature selection methods were applied to identify the different SNPs. Four different model construction methods were tested. Finally, a total of 299 SNPs were selected, and the prediction accuracy with SMO (sequential minimal optimization) evaluated using 10-fold cross validation on the training dataset achieved 0.901, and the accuracy on the independent test dataset was 0.895. Some selected SNPs demonstrated a high potential to be ethnic biomarkers, and the genes closest to those SNPs showed interesting functions, such as keratinization, which may reveal the genetic basis of some of the observed phenotype differences, such as skin color, between different ethnic populations.

## Methods

### The genotype data set

We downloaded the genomes of different ethnic groups from the HapMap Phase 3 [28] release version 3 (ftp://ftp.hgsc.bcm.tmc.edu/HapMap3-ENCODE/HapMap3/HapMap3v3), which includes 1397 samples and 1,457,897 SNPs among 11 ethnic groups. Because the Chinese and Japanese samples were very similar [28, 29], they (CHB: Han Chinese in Beijing, China, CHD: Chinese in Metropolitan Denver, Colorado and JPT : Japanese in Tokyo, Japan) were combined. To compile an independent test dataset, we randomly chose 15 % of the samples from each population. The other 85 % of the samples formed the training dataset. The final nine ethnic groups and their sample sizes in the training and independent test dataset are shown in Table 1.

**Table 1:** The 1397 samples from nine ethnic groups

Index	Abbreviation	Full Name	Training Sample Size	Independent Test Sample Size
1	ASW	African ancestry in Southwest USA	74	13
2	CEU	Utah residents with Northern and Western European ancestry from the CEPH collection	140	25
3	CHB/CHD/JPT	Han Chinese in Beijing, China/ Chinese in Metropolitan Denver, Colorado/Japanese in Tokyo, Japan	305	54
4	GIH	Gujarati Indians in Houston, Texas	86	15
5	LWK	Luhya in Webuye, Kenya	94	16
6	MEX	Mexican ancestry in Los Angeles, California	73	13
7	MKK	Maasai in Kinyawa, Kenya	156	28
8	TSI	Tuscan in Italy	87	15
9	YRI	Yoruban in Ibadan, Nigeria (West Africa)	173	30
Total			1188	209

The original PED and MAP files (hapmap3_r3_b36_fwd.consensus.qc.poly.ped.gz and hapmap3_r3_b36_fwd.consensus.qc.poly.map.gz) were transformed into a matrix using PLINK [30] with “--recodeA” and read into R using package adegenet [31] (http://cran.r-project.org/web/packages/adegenet/). The genotype matrix was a matrix of 0, 1 and 2, which were the numbers of the minor SNP alleles in that sample.

### Irrelevant SNPs were excluded using Cramer’s V coefficient

Because there were too many SNPs and because most of them differed among the ethnic groups, we calculated the Cramer’s V coefficient [32] for each SNP and removed the SNPs with Cramer’s V coefficients smaller than or equal to 0.6.

The Cramer’s V coefficient measured the association between SNP status and ethnic groups and was defined as follows:

1 $$ V=\sqrt{\frac{\chi^2/N}{ \min \left(k-1,r-1\right)}} $$

where *N* was the total number of genotype samples, 1397 in our study, *k* was the number of ethnic groups (*k* = 9) and *r* was the number referring to the SNP status (*r* = 3, for “0 minor allele”, “1 minor allele” and “2 minor allele”). $\chi$^2^ is Pearson’s chi-squared statistic, which can be calculated as follows:

2 $$ {\chi}^2={\displaystyle \sum_{i=1}^k{\displaystyle \sum_{j=1}^r\frac{{\left({O}_{i,j}-{E}_{i,j}\right)}^2}{E_{i,j}}}} $$

where *O*_*i*,*j*_ is the number of the occurrences of SNP status *j* among ethnic group *i* and *E*_*i,j*_ is the expected occurrences of SNP status *j* among ethnic group *i*, which can be calculated as follows:

3 $$ {E}_{i,j}=\frac{n_i\times {m}_j}{N} $$

where *n*_*i*_ is the number of samples in ethnic group *i* and *m*_*j*_ is the number of samples with SNP status *j*.

The Cramer’s *V* coefficient ranges from 0 to 1, where 0 indicates no association between the SNP status and ethnic group and 1 indicates a complete association between SNP status and ethnic group.

The Cramer’s *V* coefficients of the 1,457,897 SNPs were calculated using the function CramerV from R package DescTools https://cran.r-project.org/web/packages/DescTools/. The 2,448 SNPs with Cramer’s *V* coefficients greater than 0.6 on the training dataset were considered to be candidate SNPs and were analyzed using more advanced machine learning based feature selections [33–36] to obtain the optimal discriminating SNPs.

### The optimal SNPs were selected using mRMR and IFS

We applied a widely used [37–39] mutual information based method, mRMR (minimal Redundancy Maximal Relevance) [40], to rank the SNPs. The mRMR program was downloaded from http://penglab.janelia.org/proj/mRMR/. Unlike a univariate filter, such as Cramer’s V coefficient, mRMR not only considered the associations between SNPs and ethnic groups but also the redundancies between SNPs.

Ω, Ω_*s*_ and Ω_*t*_ were used to denote the entire set of 2,448 (N) candidate SNPs, the selected m SNPs, and the to-be-selected n SNPs, respectively. The relevance of the SNP *f* from Ω_*t*_ with ethnic group *c* can be measured with mutual information [41, 42] (*I*):

4 $$ D=I\left(f,\;c\right) $$

In addition, the redundancy *R* of the SNP *f* with the selected SNPs can be calculated as follows:

5 $$ R=\frac{1}{m}{\displaystyle \sum_{f_i\in {\varOmega}_s}I\left(f,{f}_i\right)} $$

To obtain the SNP *f*_*j*_ from Ω_*t*_ with maximum relevance with ethnic group *c* and minimum redundancy with the already-selected SNPs, the mRMR function was defined as follows:

6 $$ \underset{f_j\in {\varOmega}_t}{ \max}\left[I\left({f}_j,c\right)-\frac{1}{m}{\displaystyle \sum_{f_j\in {\varOmega}_s}I\left({f}_{j,}{f}_i\right)}\right]\left(j=1,2,\dots, n\right) $$

The mRMR feature evaluation is continued for N rounds, and then a ranked SNP list *S* using the mRMR method is obtained:

7 $$ S=\left\{{f}_1^{\hbox{'}},{f}_2^{\hbox{'}},\dots, {f}_h^{\hbox{'}},\dots, {f}_N^{\hbox{'}}\right\} $$

The SNP with a smaller index h has a better trade-off between relevance and redundancy and is more important for classifying samples from different ethnic groups.

Based on the top 2,448 mRMR SNPs, we constructed 2,448 classifiers and applied an Incremental Feature Selection (IFS) method [43–47] to identify the optimal SNP set. Candidate SNP set *S*_*i*_ = {*f*_1_, *f*_2_, …, *f*_*i*_}(1 ≤ *i* ≤ 2, 448) included the top *i* SNPs.

Based on the prediction performance of each candidate SNP set, an IFS curve was plotted. The x-axis denoted the number of SNPs, and the y-axis denoted the 10-fold cross validation accuracies using these SNPs.

### Different predictive models were compared

**Fig. 1:**
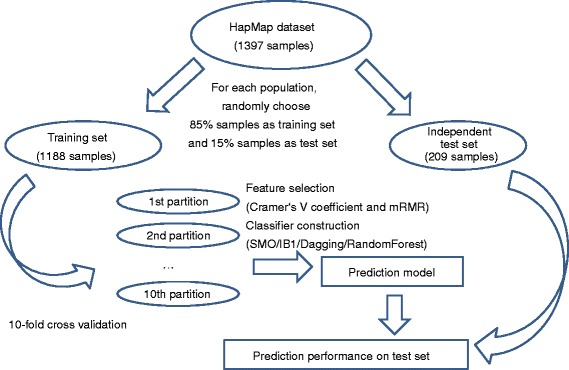
Flowchart for the predictive model construction and performance evaluation. First, we randomly divided the HapMap dataset into the training set (85 % of samples from each population) and independent test set (15 % of samples from each population). Then, the training samples were further partitioned into 10 equally sized partitions for 10-fold cross validation. Based on the training dataset, the features were selected, and the predictive model was constructed. Finally, the constructed model was tested on the independent test dataset

We used 10-fold cross validation [48, 49] to test the predictive performance of the predictive models on the training dataset and then tested the trained model on the independent test dataset. During 10-fold cross validation, all of the samples were randomly divided into 10 equal parts; in each iteration, nine parts were used to train the classifier, and the remaining part was used for the test. After 10 rounds, all samples were predicted with an ethnic group, and the predicted ethnic groups were compared with the actual ethnic groups. The entire training dataset was used to train the final predictive model, which was then tested on the independent test dataset. Figure 1 showed the flowchart of model construction and performance evaluation. The predictive accuracy of ethnic group *i* was

8 $$ {\mathrm{Q}}_i=\frac{{\mathrm{T}}_i}{{\mathrm{N}}_i} $$

where N_*i*_ is the number of samples in ethnic group *i* and T_*i*_ is the number of correctly predicted samples in ethnic group *i*. The total accuracy [50, 51] was

9 $$ \mathrm{Q}=\frac{{\displaystyle \sum_{i=1}^9{\mathrm{T}}_i}}{{\displaystyle \sum_{i=1}^9{\mathrm{N}}_i}} $$

We constructed the classifiers by using four common predictive methods: SMO (sequential minimal optimization), IB1 (nearest neighbor algorithm), Dagging, and RandomForest (random forest) in Weka [52]. Weka is an easy-to-use software package that integrated various machine learning models and can be downloaded from http://www.cs.waikato.ac.nz/ml/weka/.

The SMO method is an algorithm for building support vector machine (SVM) models [53]. The optimization of an SVM was broken into a series of the sub-problems, which were as small as possible and were then solved analytically [53]. Because there were nine ethnic groups, the prediction problem was multi-class, and pairwise coupling [54] was adopted to construct the multi-class predictive model.

IB1 was an application of the nearest neighbor method [55]. The sample similarity was measured using the normalized Euclidean distance. For a test sample, the ethnic group of a training sample with closest distance was assigned as the predicted ethnic group.

Dagging was used as a meta classifier, and the ethnic group of the test sample was predicted by voting [56]. If the training dataset *ℑ* included *N* samples, they were randomly divided into *k* subsets that each contained *n* samples (*kn* ≤ *N*). In each subset, a basic model *M*_*i*_(1 ≤ *i* ≤ *k*), was trained on these *k* subsets. A test sample was predicted to be the ethnic group with most votes.

The random forest algorithm [57] was an ensemble predictor with multiple decision trees. If there were *N* samples and *M* SNPs in the training set, each tree was trained using *n* randomly selected samples. At each node, *m* features were randomly selected and used to optimize the split. The test sample was predicted to be the ethnic group with the most votes from the decision trees.

The IFS prediction accuracies of these four methods were evaluated by 10-fold cross validation and compared, and the selected model was tested on the independent test dataset.

## Results and discussion

### Identify the relevant SNPs

We analyzed the HapMap genotype data, which included 1,457,897 SNPs on 1397 samples from nine ethnic groups. The sample sizes of each ethnic group in the training dataset and independent test dataset are shown in Table 1. The high dimension of the genotype data makes their analysis difficult and time-consuming. To reduce the SNPs and remove the irrelevant SNPs that did not differ among ethnic groups, we calculated the Cramer’s V coefficient that measured the univariate association between SNP status, i.e., the number of minor alleles, and ethnic group categories in the training dataset. The 2,448 SNPs with Cramer’s V coefficient greater than 0.6 in the training dataset were considered to be relevant and were further optimized.

### The SNP set was optimized with the best classifying performance

**Fig. 2:**
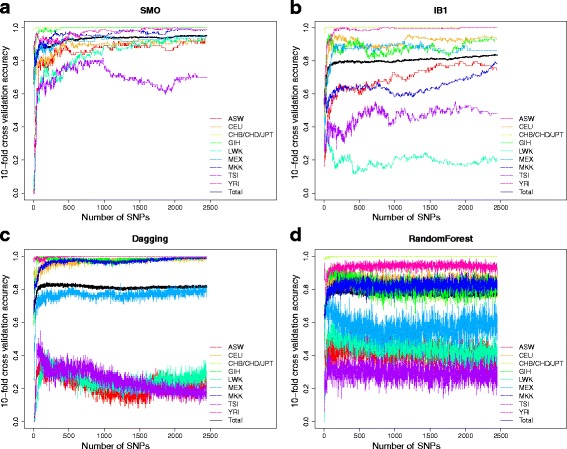
The IFS curves of four different methods. The IFS curves show how the 10-fold cross validation accuracies in each ethnic group (y-axis) change with the number of SNPs (x-axis) using SMO (**a**), IB1 (**b**), Dagging (**c**) and RandomForest (**d**) methods

We applied the mRMR method to rank the 2,448 SNPs. Then, the top SNPs were optimized using the IFS method. The predictive accuracies of the samples and each ethnic group were elevated using 10-fold cross validation. Four widely used predictive models, i.e., SMO, IB1, Dagging and RandomForest, were compared. Their performances based on using different numbers of top SNPs are shown in Fig. 2. IB1 failed to predict LWK and TSI, Dagging performed poorly on ASW, LWK and TSI, and RandomForest did not correctly predict ASW, LWK and TSI. SMO was able to predict all ethnic groups, and its total accuracy was 0.955.

In Table 2, the best predictive accuracies of each method are listed. The SMO performed best not only in total accuracy but also for almost every ethnic group. To make sure the great performances of SMO are not specific to a certain partition of training and independent test datasets, we randomly divided the training (85 % of the samples) and independent test (15 % of the samples) datasets for 30 times and for each time, the training and test processes were repeated. The mean and standard deviation of the accuracies on 30 training and independent test datasets were calculated and shown in Additional file 1. The mean accuracies were close to the accuracies of SMO in Table 2 and the standard deviations were very small which indicated that the partition of training and independent test datasets does not affect the prediction performance.

**Table 2:** The best predictive performance of the different methods

Method	#SNP	ASW	CEU	CHB/CHD/JPT	GIH	LWK	MEX	MKK	TSI	YRI	Total
SMO	2192	0.932	0.921	1.000	1.000	0.926	0.945	0.987	0.724	0.994	0.955
IB1	2413	0.757	0.943	1.000	0.930	0.213	0.863	0.795	0.483	1.000	0.838
Dagging	186	0.338	0.964	1.000	0.988	0.383	0.808	0.968	0.345	0.994	0.840
RandomForest	75	0.459	0.900	0.993	0.884	0.543	0.74	0.853	0.345	0.931	0.815

However, the best SMO model requires too many features. To balance the model complexity and predictive performance, we considered the top 299 SNPs used by the SMO to be the optimal SNP set because subsequently, upon adding more SNPs, the performance did not increase greatly. In other words, the IFS curve shown in Fig. 2a became stable after the top 299 SNPs, and the accuracy was consistently over 90 %. As shown in Table 3, the 10-fold cross validation accuracy of SMO method with the top 299 SNPs on the training dataset was 0.901, and the accuracy on the independent test dataset was 0.895. The 299 SNPs and their annotations, such as dbSNP IDs, minor alleles, chromosome positions and nearby genes (within 500Kb), are provided in Additional file 2.

**Table 3:** The predictive performance of the SMO method in the top 299 SNPs in the training and independent test dataset

Dataset	ASW	CEU	CHB/CHD/JPT	GIH	LWK	MEX	MKK	TSI	YRI	Total
Training (10-fold cross validation)	0.865	0.836	1.000	0.977	0.723	0.904	0.968	0.644	0.919	0.901
Independent test	0.846	0.760	1.000	1.000	0.688	1.000	0.786	0.800	1.000	0.895

### The allele frequency differences among ethnic groups

**Fig. 3:**
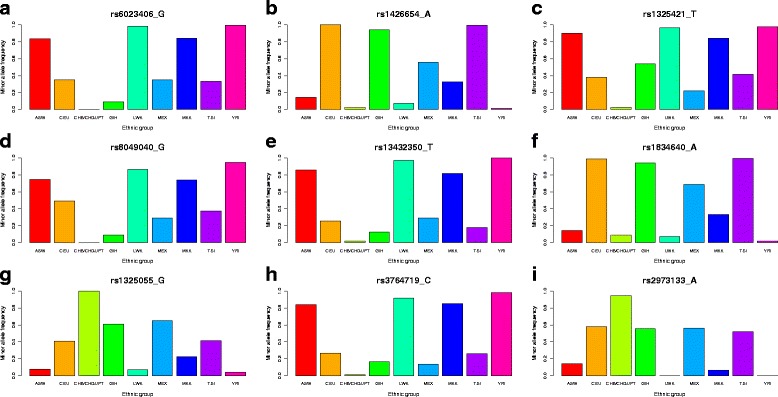
The minor allele frequency of the top nine SNPs in each ethnic group. The minor allele frequencies of the top three SNPs, rs6023406 (**a**), rs1426654 (**b**), rs1325421 (**c**), rs8049040 (**d**), rs13432350 (**e**), rs1834640 (**f**), rs1325055 (**g**), rs3764719 (**h**), rs2973133 (**i**) in the nine ethnic groups were plotted. Each ethnic group has their own specific alleles. For example, the allele frequencies of rs6023406_G, rs1426654_A, rs1325421_T, rs8049040_G, rs13432350_T, rs1834640_A and rs3764719_C were very low, but those of rs1325055_G and rs2973133_A were very high in the Asian population (CHB/CHD/JPT)

We sought to explore how these 299 SNPs differed among ethnic groups and calculated their minor allele frequency in each ethnic group. In Fig. 3, the top nine SNPs are plotted. The same plot for all 299 SNPs are provided in Additional file 3.

As shown in Fig. 3, each ethnic group has its own specific alleles. For example, the allele frequencies of rs6023406_G, rs1426654_A, rs1325421_T, rs8049040_G, rs13432350_T, rs1834640_A and rs3764719_C were very low, but those of rs1325055_G and rs2973133_A were very high in the Asian population (CHB/CHD/JPT).

### The biological relevance of likely ethnicity-related SNPs

In our study, 299 SNPs, which varied significantly among different ethnic groups, were identified. Considering the large number of our SNPs, we selected the 9 SNPs that achieved the highest score in our list. The SNP with the highest score (0.861) was rs6023406, which is located in the intron region of the DOX5 gene. As *Tabassum R* and his colleagues reported, DOX5 was a susceptibility gene for type 2 diabetes [58, 59]. Further, we know that the risk of type 2 diabetes varied greatly among Asian races and European ethnic groups [60, 61]. Globally, some regions, such as South Asians, Pacific Islanders, Latinos, and Native Americans, have a higher likelihood of developing type 2 diabetes [62]. Although the link between the different risk factors of type 2 diabetes and DOX5 was unclear, our findings might offer clues to answer this question.

rs1426654, which is a coding SNP that scores 0.581 and ranks 2nd in our analysis, was located on chromosome 15, where the G- > A transition changes p.A111T in the SLC24A5 protein. Lamason RL et al. revealed that SLC24A5 affects pigmentation in zebrafish and humans [63]. Recently, Wei A et al. identified SLC24A5 as a candidate gene for nonsyndromic oculocutaneous albinism (OSA) [64]. Interestingly, *Mikiko S* and his group investigated the allele frequency of rs1426654 in Chinese, Sinhalese and Tamils from Sri Lanka, Uygurs, Europeans, and Xhosans (Africans) from South Africa, and Ghanaians using polymerase chain reaction-restriction fragment length polymorphism. They found that the A nucleotide was predominant in the European population but exhibited low levels in the Asian population [65]. Notably, another top-ten SNP rs1834640 (6th place, with a score of 0.436) is located 21327 bp upstream of SLC24A5. Intriguingly, rs1426654 and rs1834640 had highly similar distribution of minor allele frequency among the 9 ethnic groups, which also implied the potential synergistic function of the two SNPs. However, the detailed relationship between rs1426654 (or rs1834640) and pigmentation still needs more experimental evidence.

rs1325421, the 3rd SNP, which scored 0.515 in our analysis, is located downstream from the PREP gene. PREP could reportedly play an important role in many biological processes, such as the maturation and degradation of peptide hormones and neuropeptides, learning and memory, cell proliferation and differentiation, and glucose metabolism [66]. Considering the multiple functions of PREP, it might be altered by rs1325421 and thus manifest different characteristics among different populations.

rs8049040, which ranked 4th place in our data and is located on chr15:48392415, is nearest to gene ZNF23, which was widely reported among multiple types of cancers, including liver and ovarian cancer [67–69]. Interestingly, 2 other SNPs in our top-ten list were related to cancers. One, rs1325055, is an SNP that ranked in 7th place and is located downstream of the FAM135B gene. Song Y. et al. identified the mutation on FAM135B in esophageal squamous cell cancer, which implied a biological function of FAM135B in cancer [70]. The other SNP was rs3764719, ranked in 8th place and located in Rbm38, which is a target of the p53 family and could modulate p53 expression via mRNA translation [71]. Xue JQ et al. found that Rbm38 could act as a tumor suppressor in breast cancer [72]. Furthermore, p53 deficiency was common among many types of cancers [73, 74]. In contrast, it is reported that the risk of several cancers, including breast cancer, colorectal cancer, liver cancer and lung cancer, varied among different ethnic groups [75, 76]. Nevertheless, the underlying mechanism leading to the disparities of cancer incidence remain unclear. The differences of the SNPs that were on or near cancer-related genes may shed light on the variation.

rs13432350, an SNP that ranked 5th in our analysis, is located in EXOC6B. As Evers.C et al. reported, EXOC6B might play an important role in the molecular pathogenesis of intellectual disabilities [77]. Intellectual disabilities affect approximately 2–3 % of the general population, whereas approximately 95 million cases were due to unknown causes [78]. In contrast, the highest incidence of intellectual disability was observed in low- and middle-income countries [79]. Although economic disparities should be considered, differences in SNPs such as rs13432350 may also contribute to the varied risks of intellectual disability.

rs2973133, the 9th-ranked SNP in our data, is located upstream of PRR16 gene. Liu X. et.al reported that dysfunction of PRR16 could lead to Coronary Artery Disease (CAD) [80]. In fact, the incidences of CAD varied significantly among different races; for example, almost 60 % of the world’s cardiovascular disease burden occurs in South Asia, although it only accounts for 20 % of the world’s population [81]. However, the potential underlying reasons were not fully answered, and our finding may provide an alternative explanation for the varied risks of CAD.

In addition to the top-nine SNPs on our lists, several other SNPs have a potential relationship with the varied characteristics among ethnic groups, such as rs12913832, an SNP ranked in 42nd place, which was scored as 0.386 and is located within an intron of the non-pigment gene HERC. Visser M et al. found that rs12913832 modulates human pigmentation by attenuating chromatin-loop formation between a long-range enhancer and the OCA2 promoter [82]. Mengel FJ et al. investigated rs12913832 in 395 randomly selected Danes and found that rs12913832 affects eye color [83]. In addition, Amos C et al. found that the 50 % variability in eye color is associated with variations in the rs12913832 SNP based on their GWAS, in which 1804 melanoma cases and 1026 controls were used [84]. Above all, the results of our analysis could enhance our understanding of the mechanisms of different characteristics among ethnic groups.

### The biological relevance of nearby genes

In addition to exploring the SNPs directly, we analyzed the functions of 1,397 genes located within a 500 kb range of the 299 SNPs using DAVID. The results are shown in Table 4. The most enriched gene ontology (GO) biological process (BP) terms were “GO: 0031424 keratinization” and “GO: 0030216 keratinocyte differentiation” [85]. During keratinization, keratinocytes become cornified as keratin protein is incorporated into longer keratin intermediate filaments; they eventually undergo apoptosis and become fully keratinized [86]. Keratinization is indispensable to the development of the epidermis and for hair growth [87]. Therefore, we speculated that the various SNPs may contribute to the differences in hair or skin characteristics among populations by affecting the critical genes related to keratinization. Furthermore, some diseases were also related to keratinization, such as pachyonychia congenita (PC), dyskeratosis congenita (DC), and Darier’s disease [88–90]. Although no population pattern about these diseases have been reported, our results indicated potential possibilities for the population distribution of these diseases. In addition to keratinization, the “GO:0030855: epithelial cell differentiation” and “GO: 0009913 epidermal cell differentiation” were included at the top of our list. Several skin disorders, such as epidermolytic hyperkeratosis and epidermolysis bullosa simplex, occur if epidermis development is disrupted [91]. The most enriched GO cellular component (CC) term was “GO: 0001533 cornified envelope”. To our knowledge, the cornified envelope is a structure that forms beneath the plasma membrane in terminally differentiating stratified squamous epithelia, and it is essential for effective physical and water barrier function in the skin [92]. We surmised that these components could contribute to these differences, especially those that are directly or indirectly related to skin color diversity among populations.

**Table 4:** Gene ontology enrichments of genes close to the 427 SNPs

Term	*P* Value	Fold Enrichment	Benjamini adjusted *P* value
GO:0031424 ~ keratinization (BP)	3.37E-06	4.77	0.00998
GO:0030216 ~ keratinocyte differentiation (BP)	6.41E-06	3.78	0.00948
GO:0030855 ~ epithelial cell differentiation (BP)	1.64E-05	2.67	0.01613
GO:0009913 ~ epidermal cell differentiation (BP)	2.09E-05	3.46	0.01545
GO:0001533 ~ cornified envelope (CC)	4.76E-06	6.95	0.00228

## Conclusions

Above all, we learned that the various SNPs could contribute to different characteristics, including skin color, eye color and the risk of diseases, especially skin-related disorders, among different populations. Our study revealed a large spectrum of SNPs that could facilitate our understanding of the different characteristics between populations and the underlying mechanisms of molecular evolution.

### Data availability

All data are public available from HapMap project at ftp://ftp.hgsc.bcm.tmc.edu/HapMap3-ENCODE/HapMap3/HapMap3v3.

## Additional files

Additional file 1:
**The predictive performance of the SMO method on 30 randomly divided training and independent test datasets.** The training (85 % of the samples) and independent test (15 % of the samples) datasets were randomly divided for 30 times and for each time, the training and test processes were repeated. Then the mean and standard deviation of the accuracies on 30 training and independent test datasets were calculated. As shown in this table, the mean accuracies were close to the accuracies of SMO in Table 2 and the standard deviations were very small which indicated that the partition of training and independent test datasets does not affect the prediction performance. (XLSX 20 kb) Additional file 2:
**The 299 optimal SNPs that can classify the nine ethnic groups with accuracy greater than 90 %.** The order is the mRMR rank, and the SNP name includes the dbSNP ID and the minor allele. (XLSX 37 kb) Additional file 3:
**The minor allele frequencies of the 299 optimal SNPs in each ethnic group.** Each page is a SNP. (PDF 238 kb)

## Abbreviations

SNP: Single nucleotide polymorphism
NCBI: National Center for Biotechnology Information
CDS: Coding sequence
GWAS: Genome-Wide Association Study
SMO: Sequential minimal optimization
mRMR: Minimal Redundancy Maximal Relevance
