# Supplementary material for: Genetic differences among ethnic groups
Source: BMC Genomics. 2015 Dec 21;16:1093. doi: 10.1186/s12864-015-2328-0 (PMC4687076; doi:10.1186/s12864-015-2328-0)
Supplement: Additional file 3: — The minor allele frequencies of the 299 optimal SNPs in each ethnic group. Each page is a SNP. (PDF 238 kb) [file 12864_2015_2328_MOESM3_ESM.pdf]

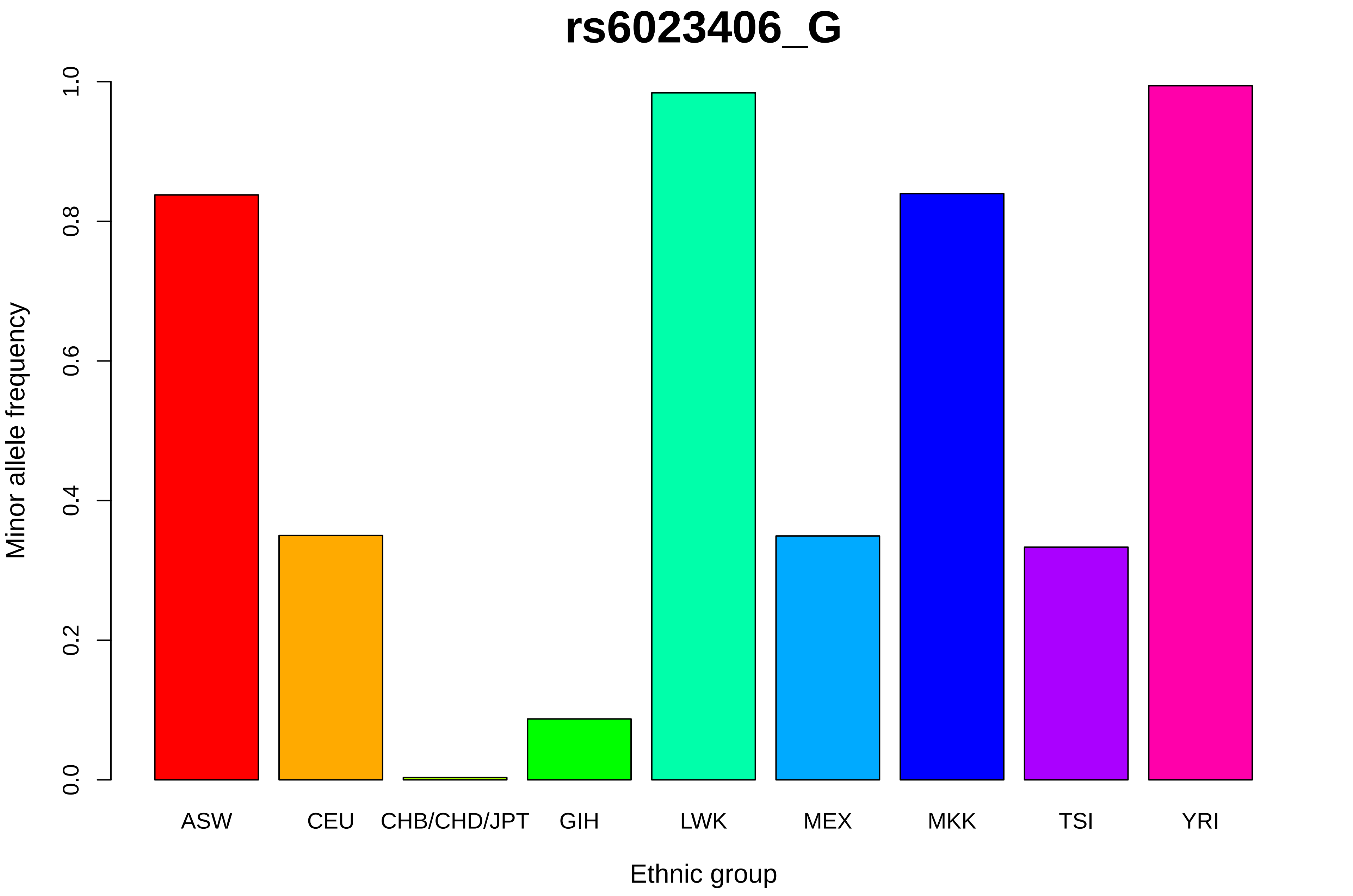
rs6023406_G
Minor allele frequency
0.0
0.2
0.4
0.6
0.8
1.0
ASW
CEU
CHB/CHD/JPT
GIH
LWK
MEX
MKK
TSI
YRI
Ethnic group

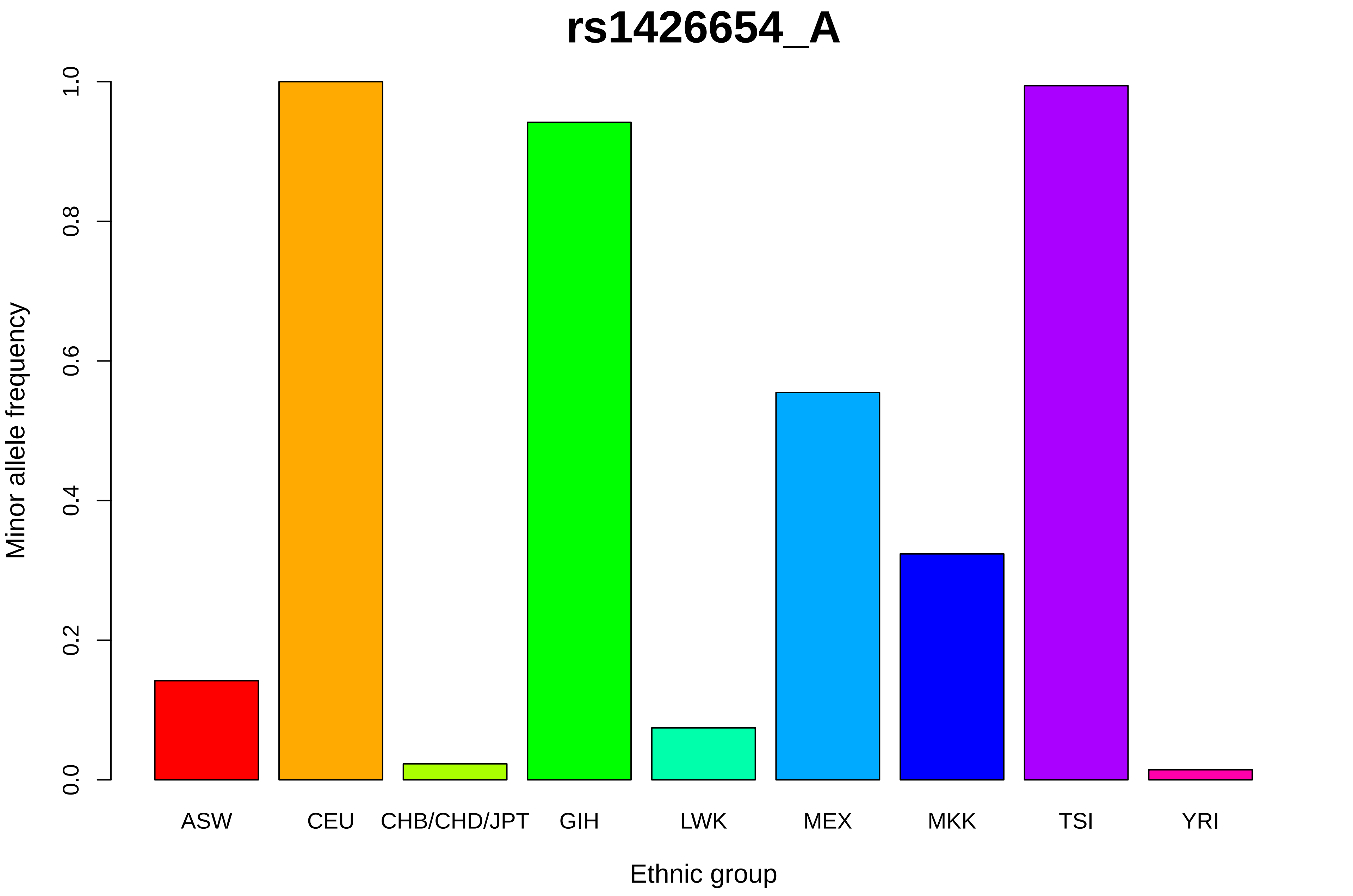

rs1426654_A
Minor allele frequency
0.0
0.2
0.4
0.6
0.8
1.0
ASW
CEU
CHB/CHD/JPT
GIH
LWK
MEX
MKK
TSI
YRI
Ethnic group

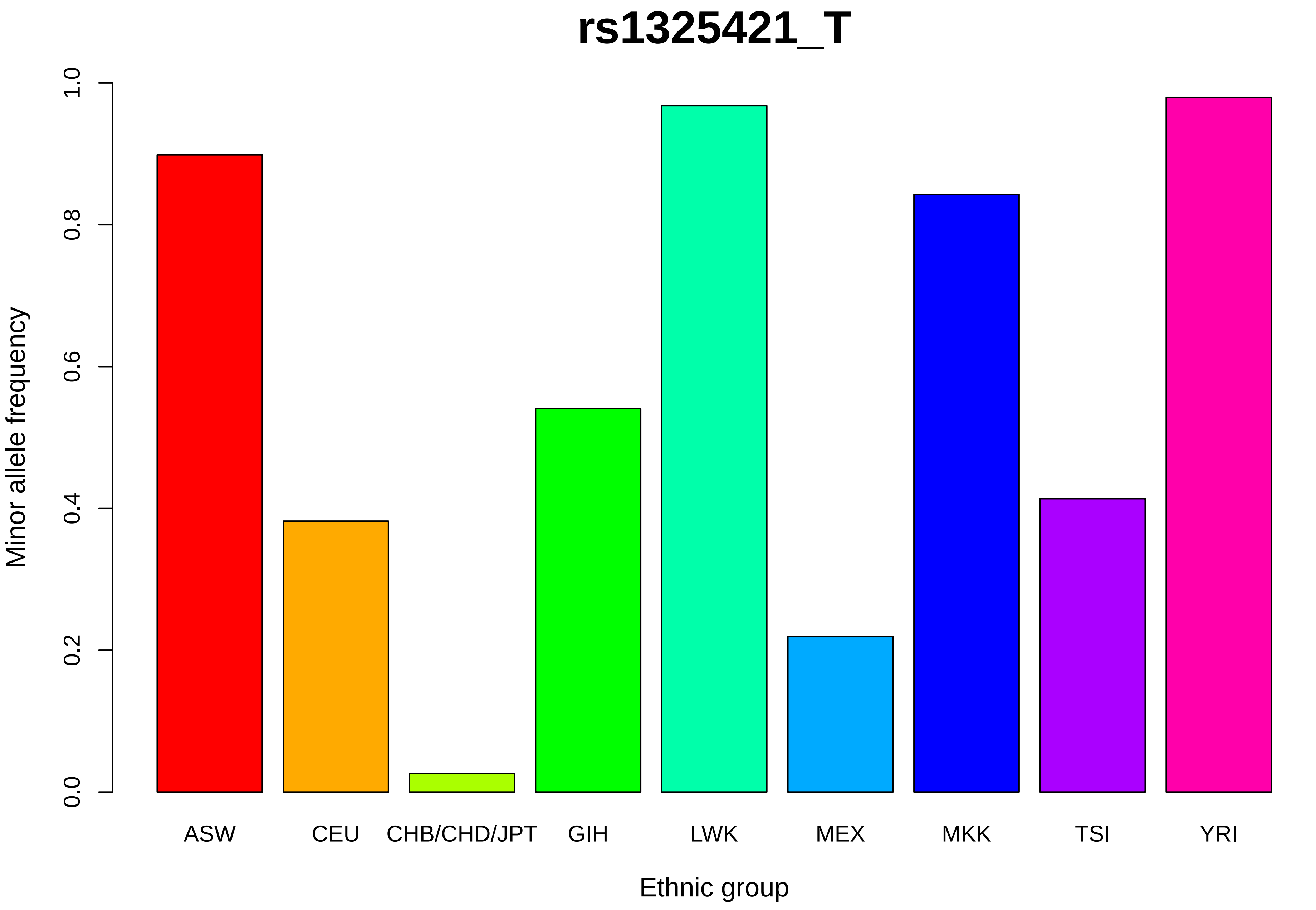

rs1325421_T
Minor allele frequency
0.0
0.2
0.4
0.6
0.8
1.0
ASW
CEU
CHB/CHD/JPT
GIH
LWK
MEX
MKK
TSI
YRI
Ethnic group

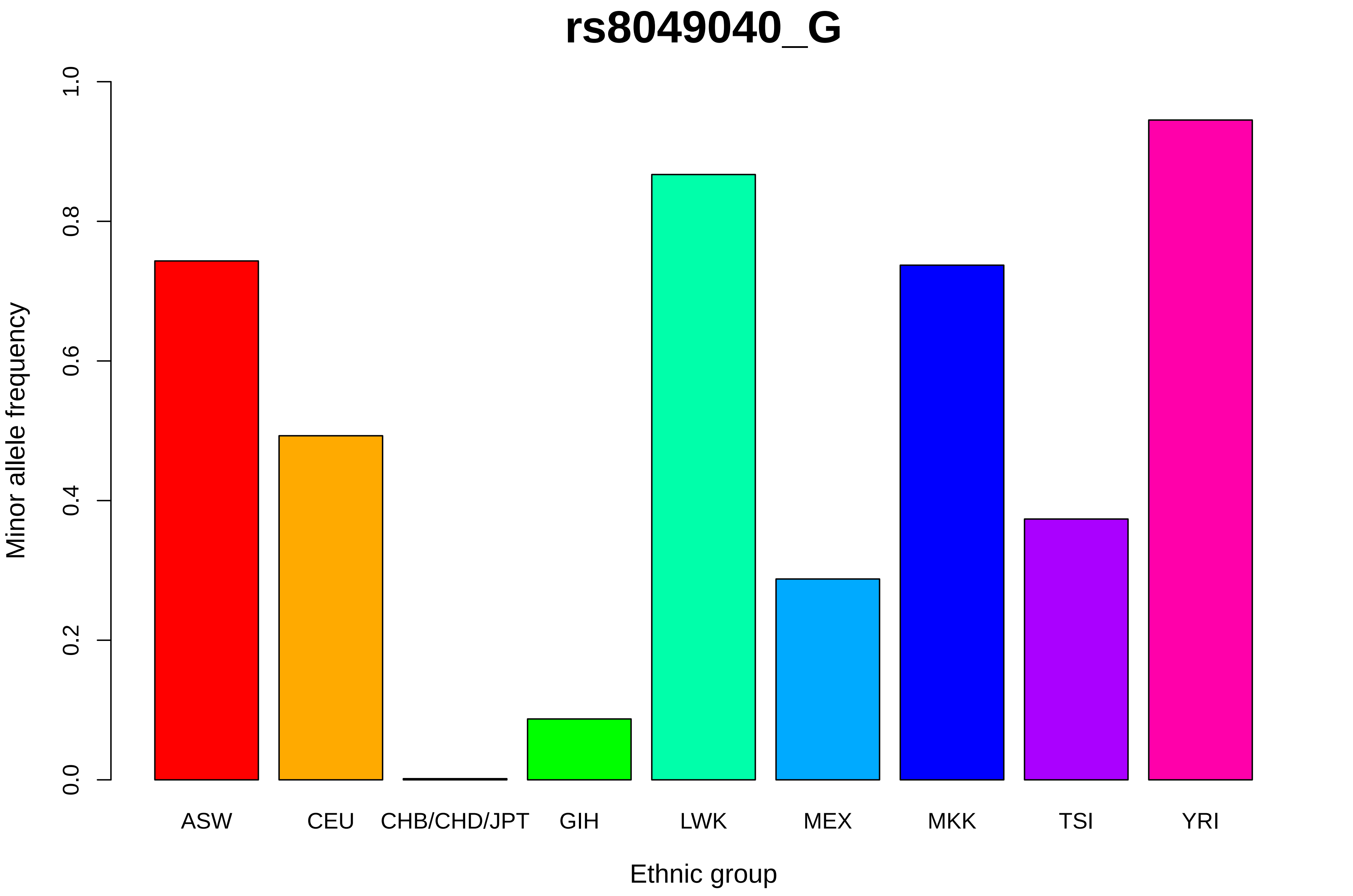
rs8049040_G
Minor allele frequency
0.0
0.2
0.4
0.6
0.8
1.0
ASW
CEU
CHB/CHD/JPT
GIH
LWK
MEX
MKK
TSI
YRI
Ethnic group

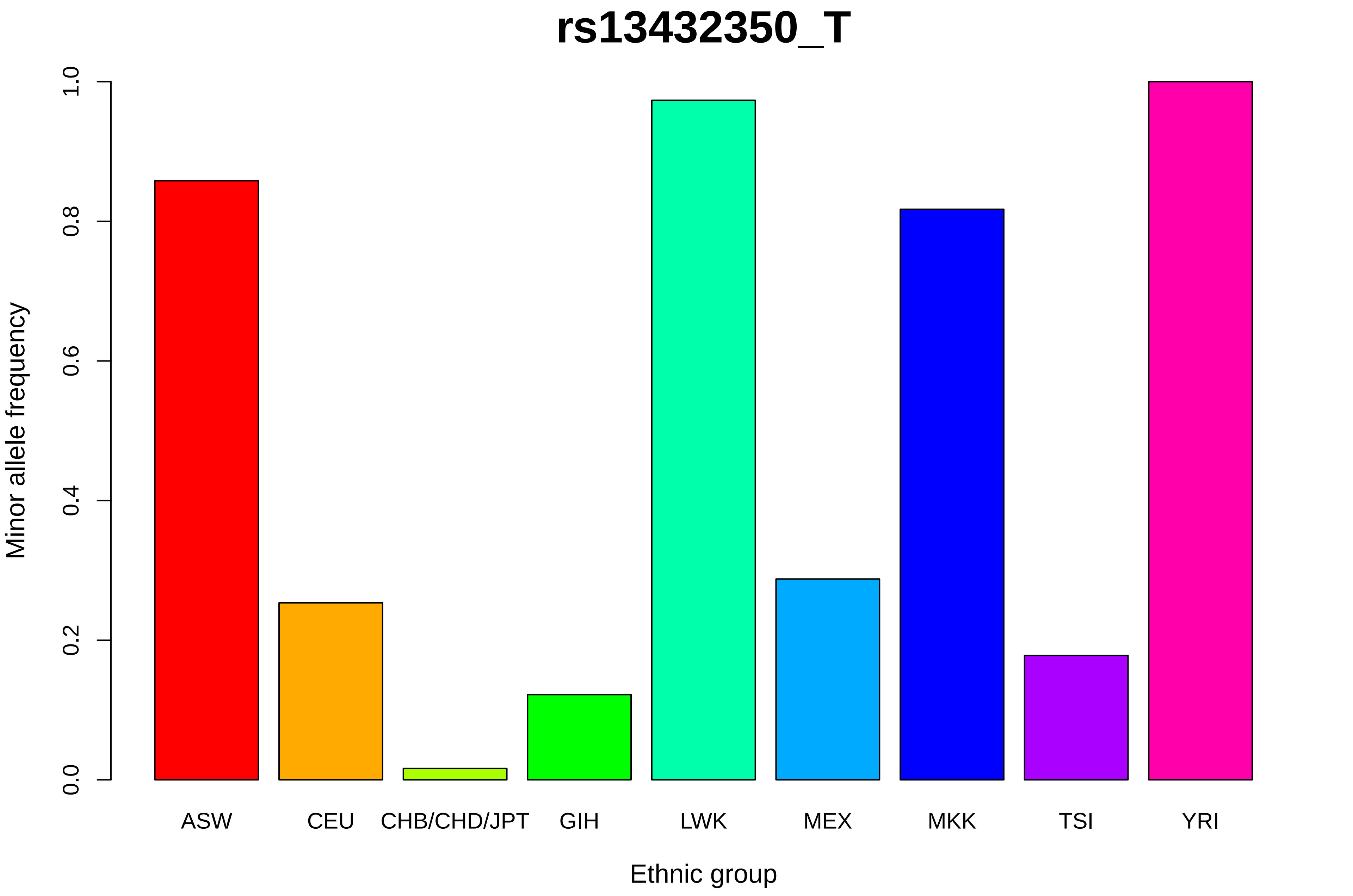
rs13432350_T
Minor allele frequency
0.0
0.2
0.4
0.6
0.8
1.0
ASW
CEU
CHB/CHD/JPT
GIH
LWK
MEX
MKK
TSI
YRI
Ethnic group

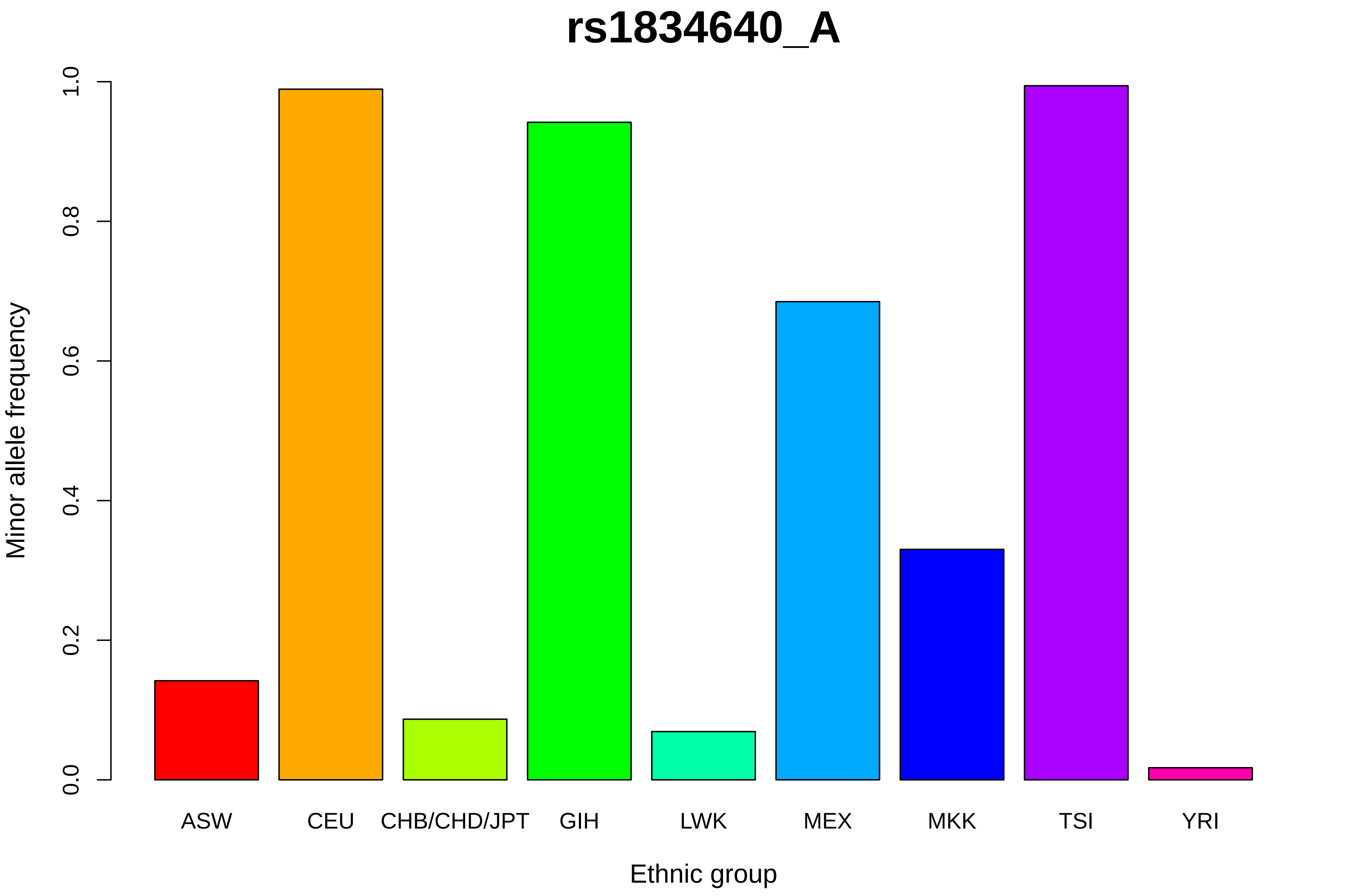
rs1834640_A
Minor allele frequency
0.0
0.2
0.4
0.6
0.8
1.0
ASW
CEU
CHB/CHD/JPT
GIH
LWK
MEX
MKK
TSI
YRI
Ethnic group

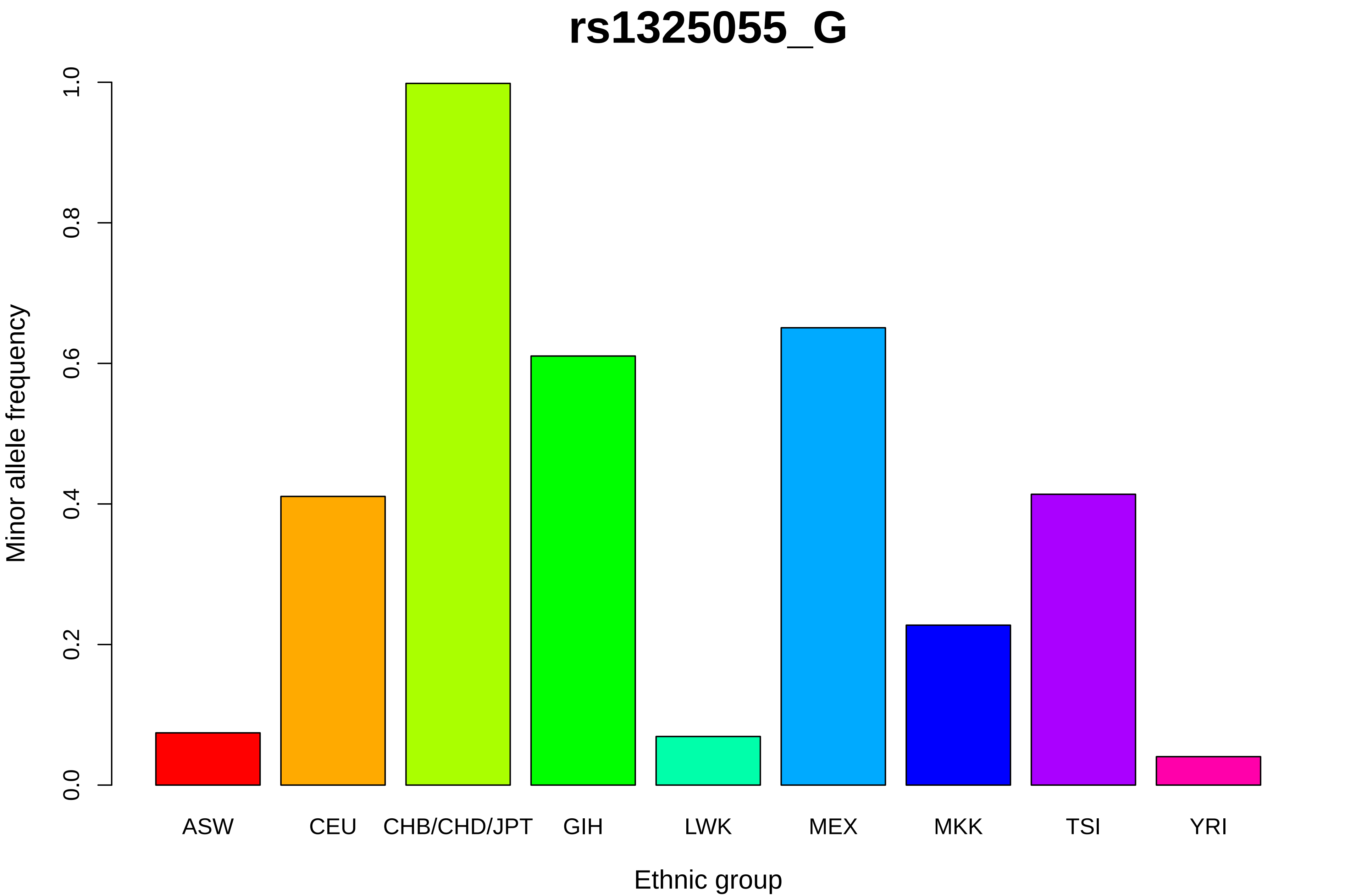
rs1325055_G
Minor allele frequency
0.0
0.2
0.4
0.6
0.8
1.0
ASW
CEU
CHB/CHD/JPT
GIH
LWK
MEX
MKK
TSI
YRI
Ethnic group

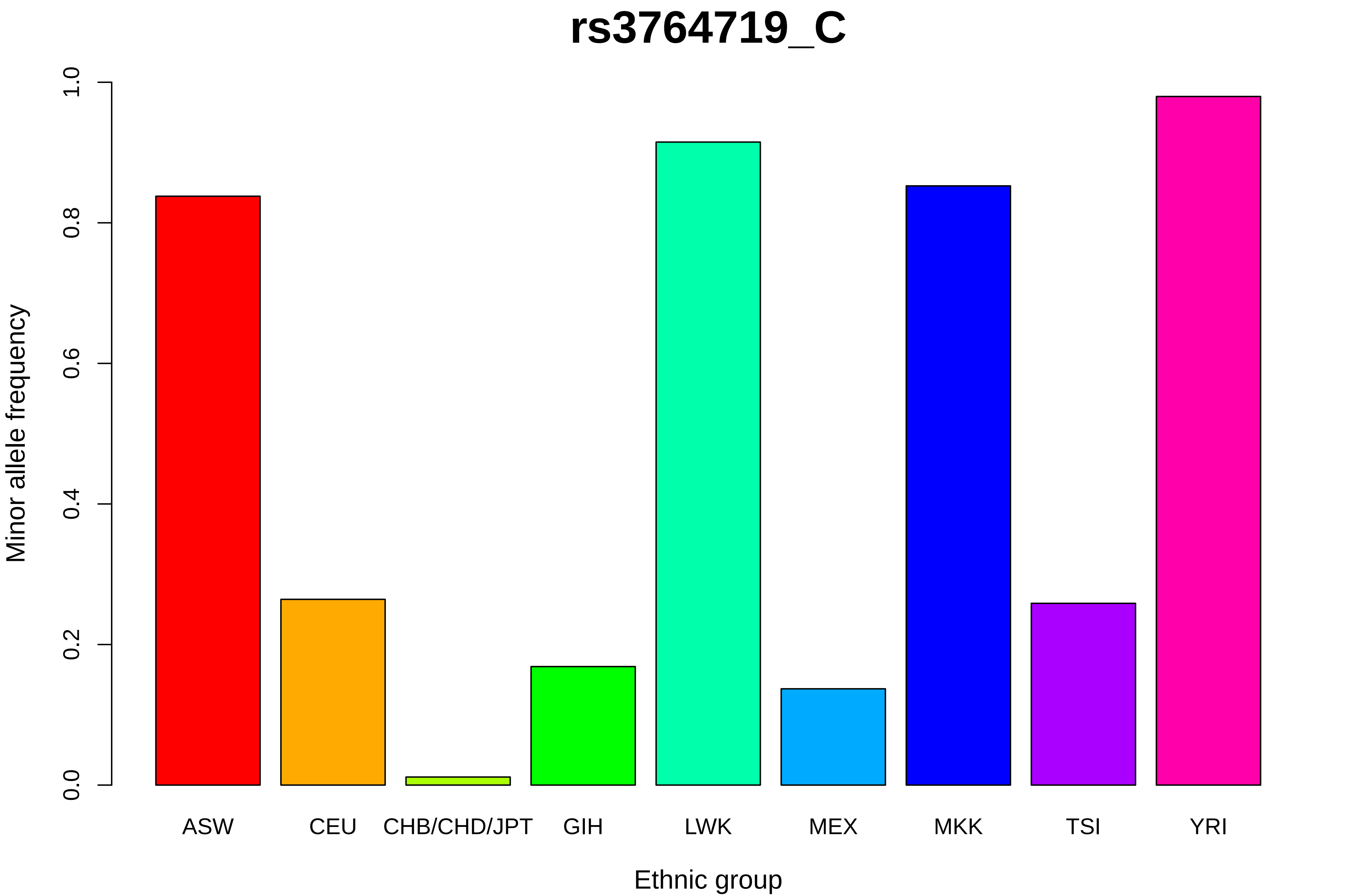
rs3764719_C
Minor allele frequency
0.0
0.2
0.4
0.6
0.8
1.0
ASW
CEU
CHB/CHD/JPT
GIH
LWK
MEX
MKK
TSI
YRI
Ethnic group

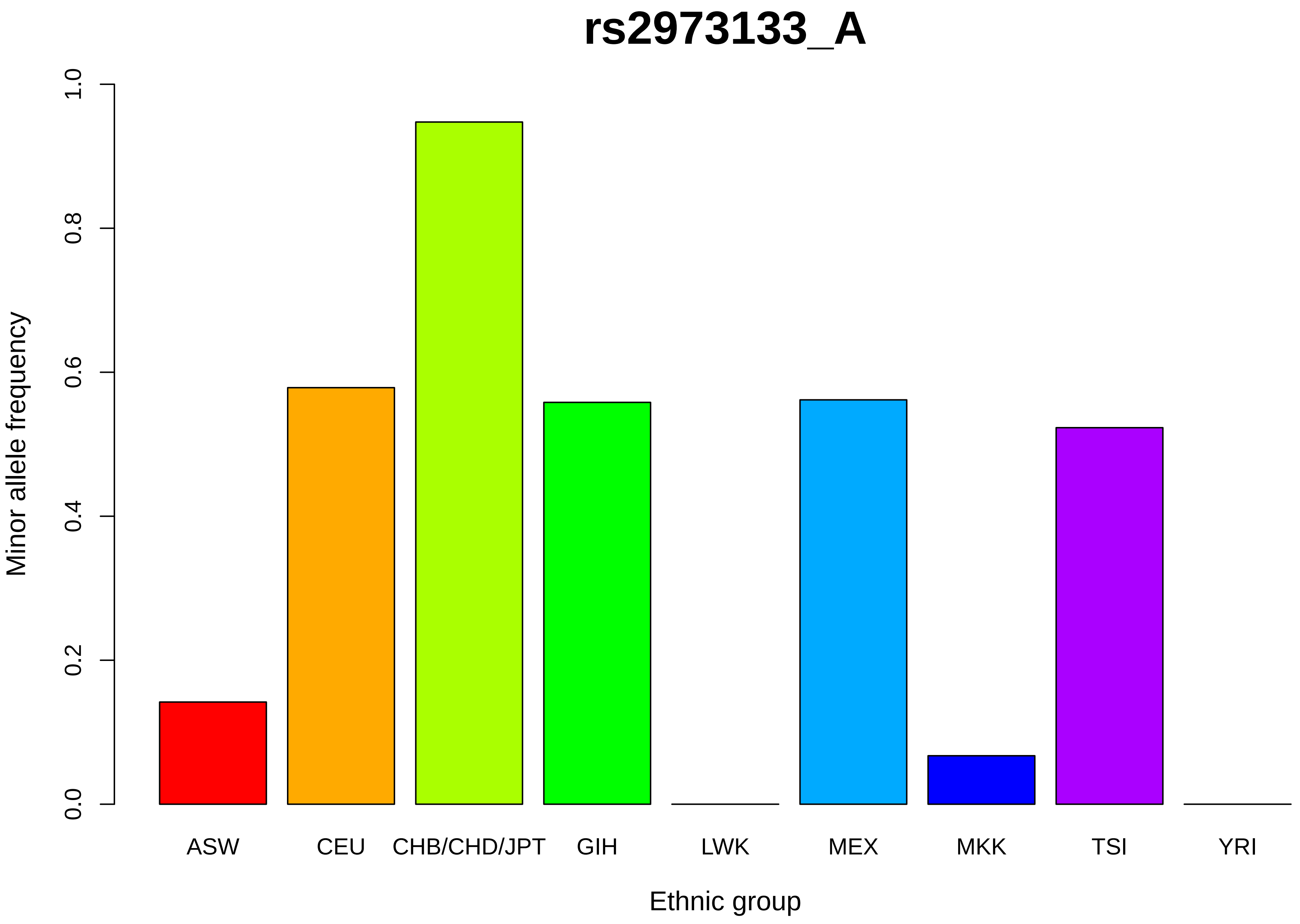

rs2973133_A
Minor allele frequency
0.0
0.2
0.4
0.6
0.8
1.0
ASW
CEU
CHB/CHD/JPT
GIH
LWK
MEX
MKK
TSI
YRI
Ethnic group

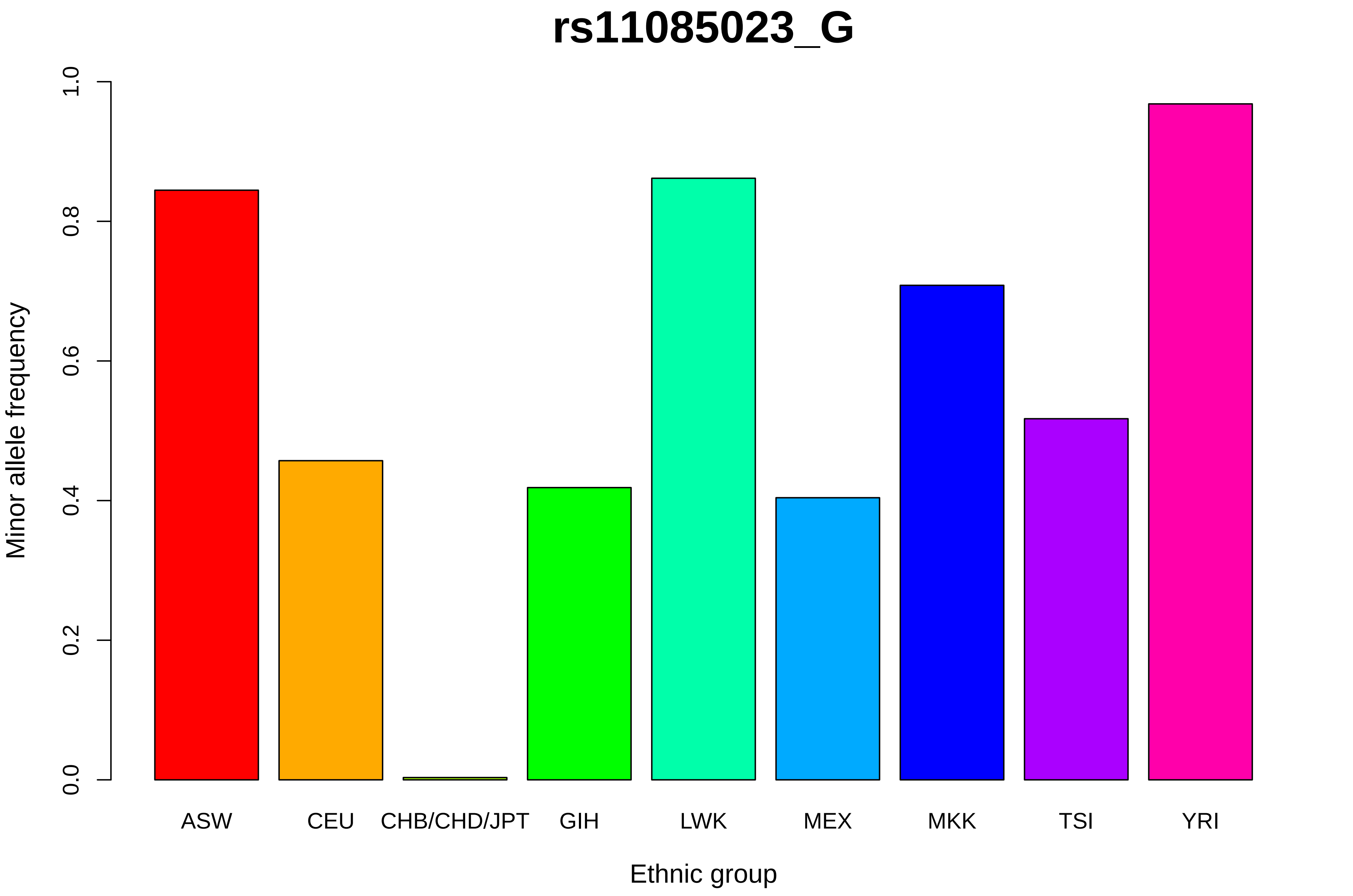
rs11085023_G
Minor allele frequency
0.0
0.2
0.4
0.6
0.8
1.0
ASW
CEU
CHB/CHD/JPT
GIH
LWK
MEX
MKK
TSI
YRI
Ethnic group

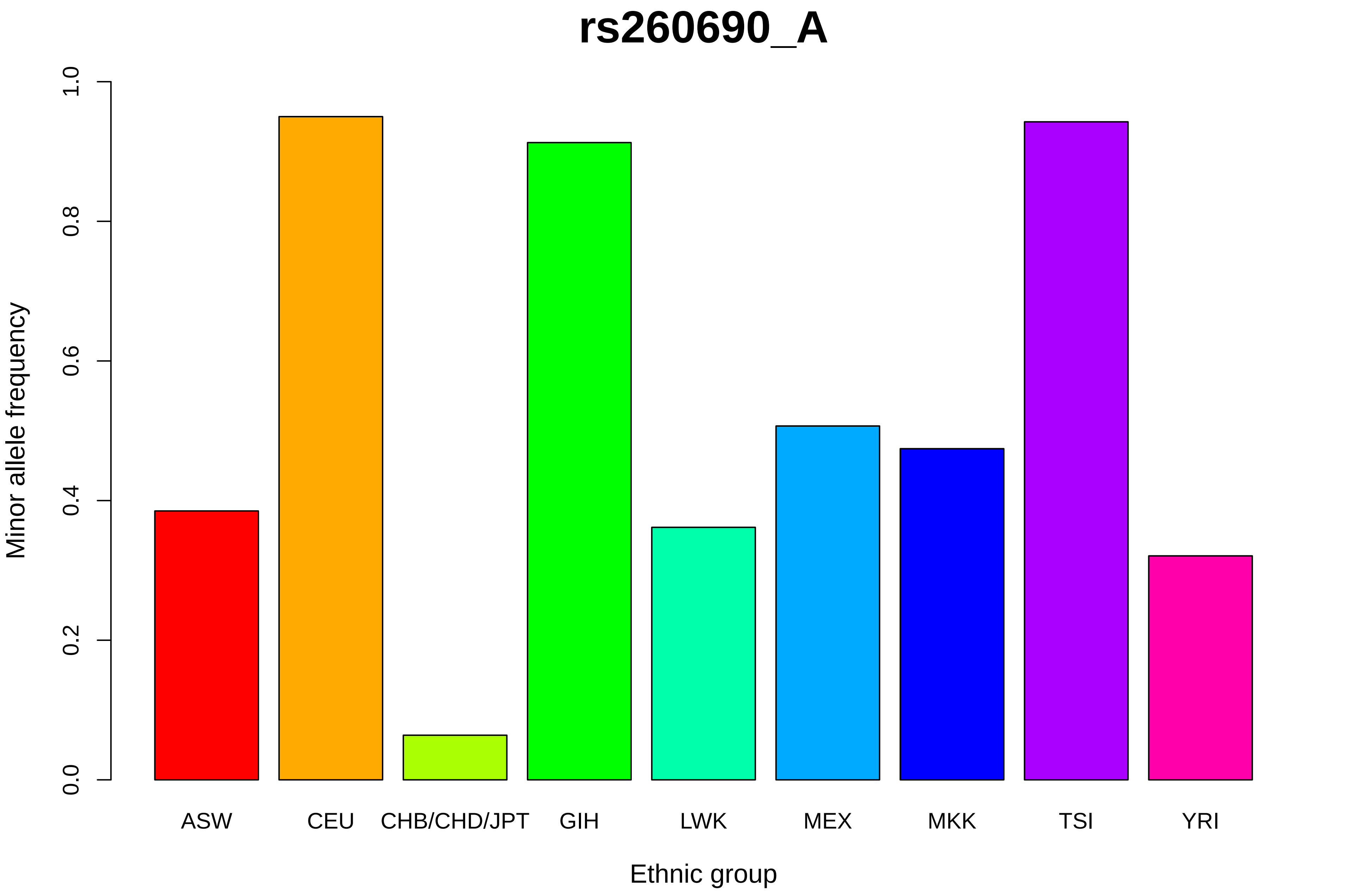
rs260690_A
Minor allele frequency
0.0
0.2
0.4
0.6
0.8
1.0
ASW
CEU
CHB/CHD/JPT
GIH
LWK
MEX
MKK
TSI
YRI
Ethnic group

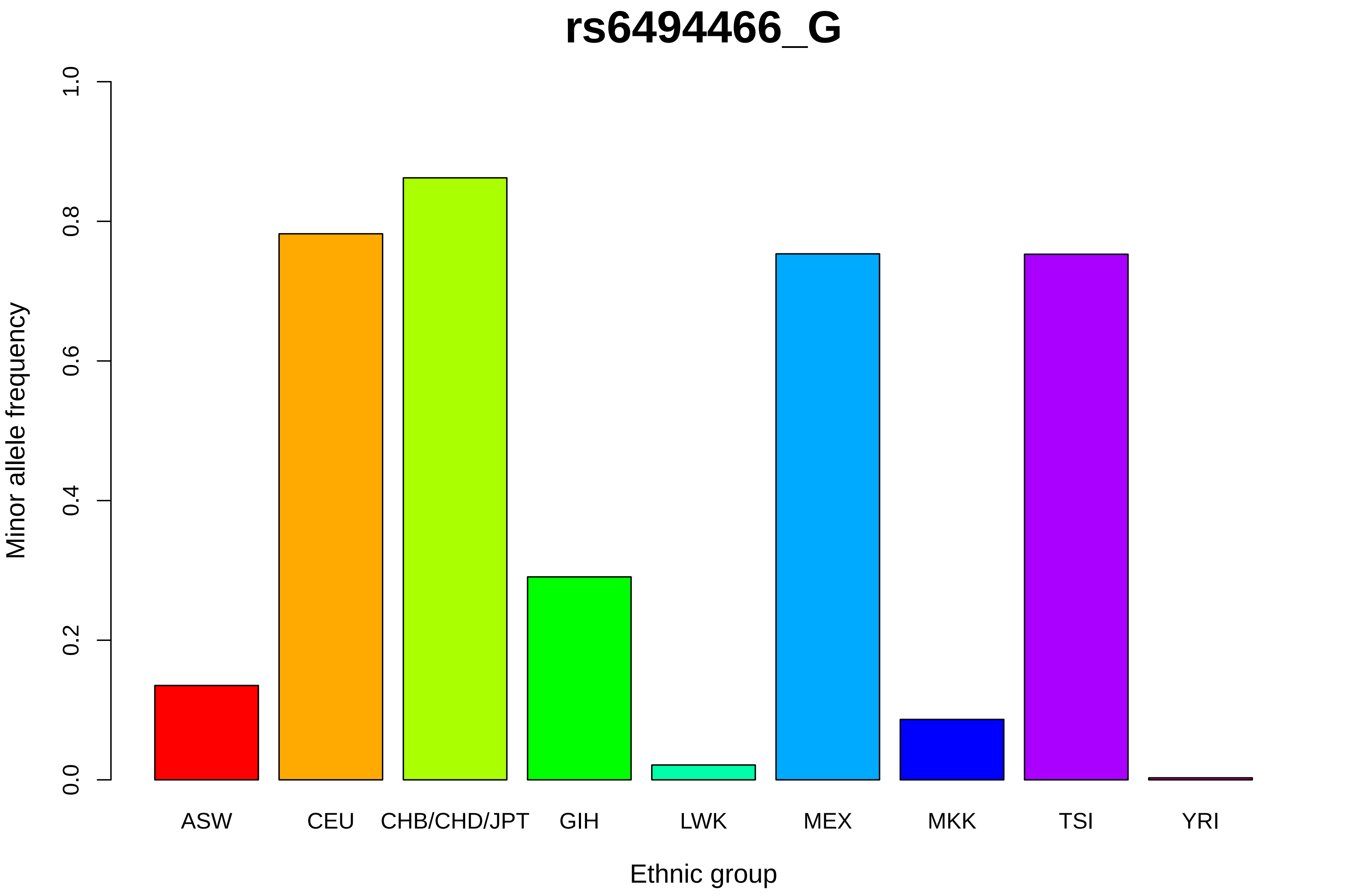
rs6494466_G
Minor allele frequency
0.0
0.2
0.4
0.6
0.8
1.0
ASW
CEU
CHB/CHD/JPT
GIH
LWK
MEX
MKK
TSI
YRI
Ethnic group

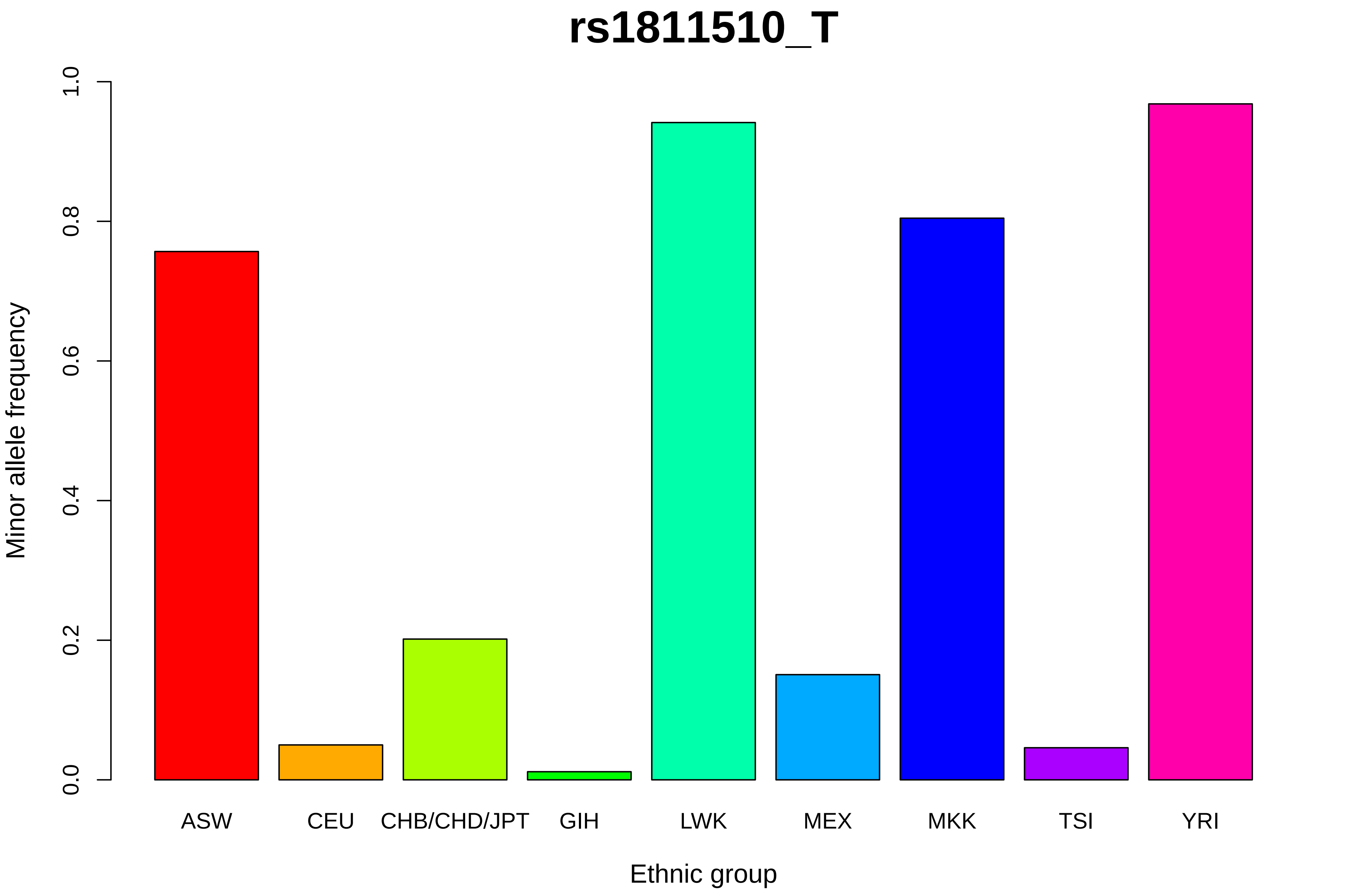
rs1811510_T
Minor allele frequency
0.0
0.2
0.4
0.6
0.8
1.0
ASW
CEU
CHB/CHD/JPT
GIH
LWK
MEX
MKK
TSI
YRI
Ethnic group

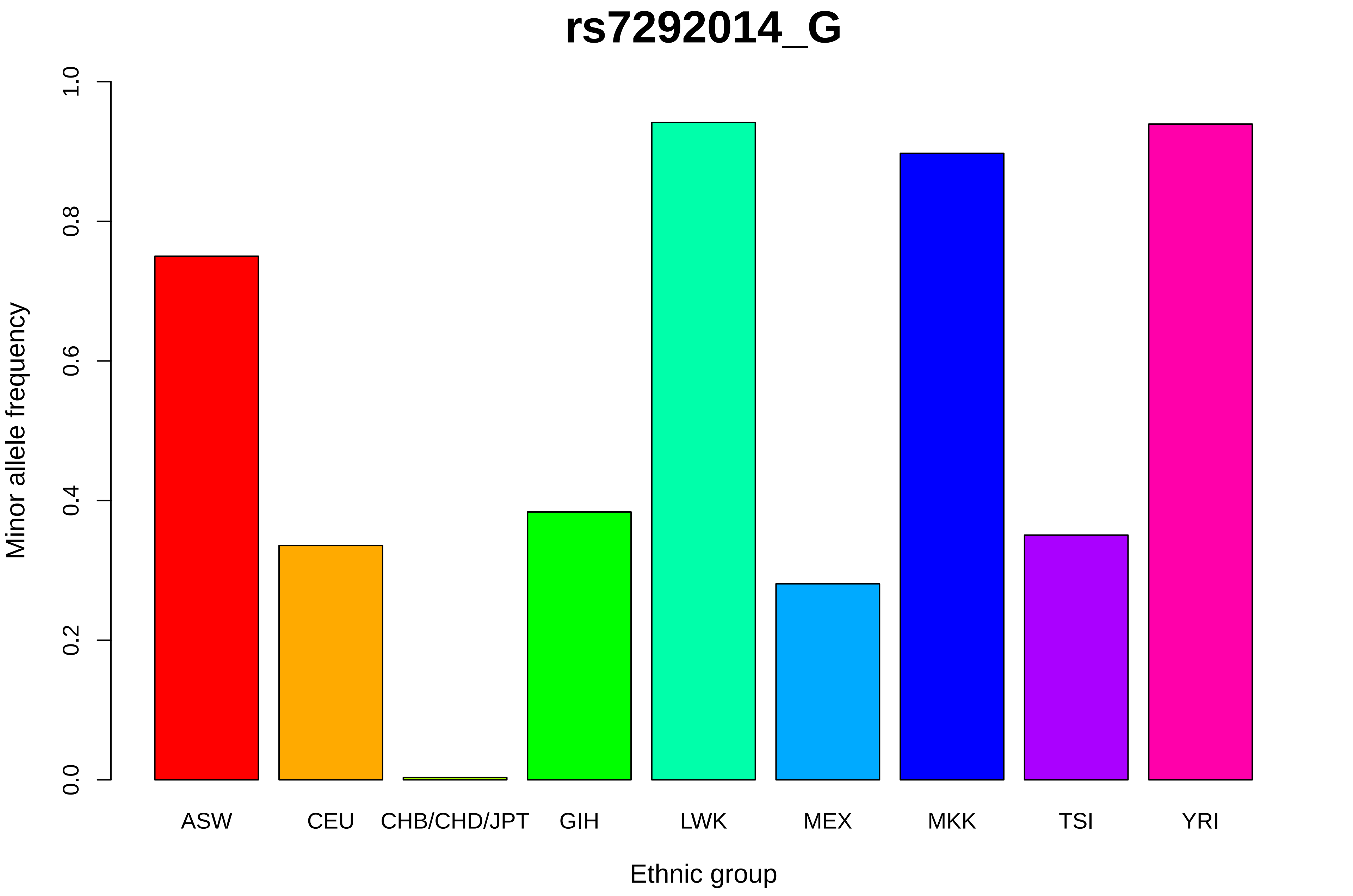
rs7292014_G
Minor allele frequency
0.0
0.2
0.4
0.6
0.8
1.0
ASW
CEU
CHB/CHD/JPT
GIH
LWK
MEX
MKK
TSI
YRI
Ethnic group

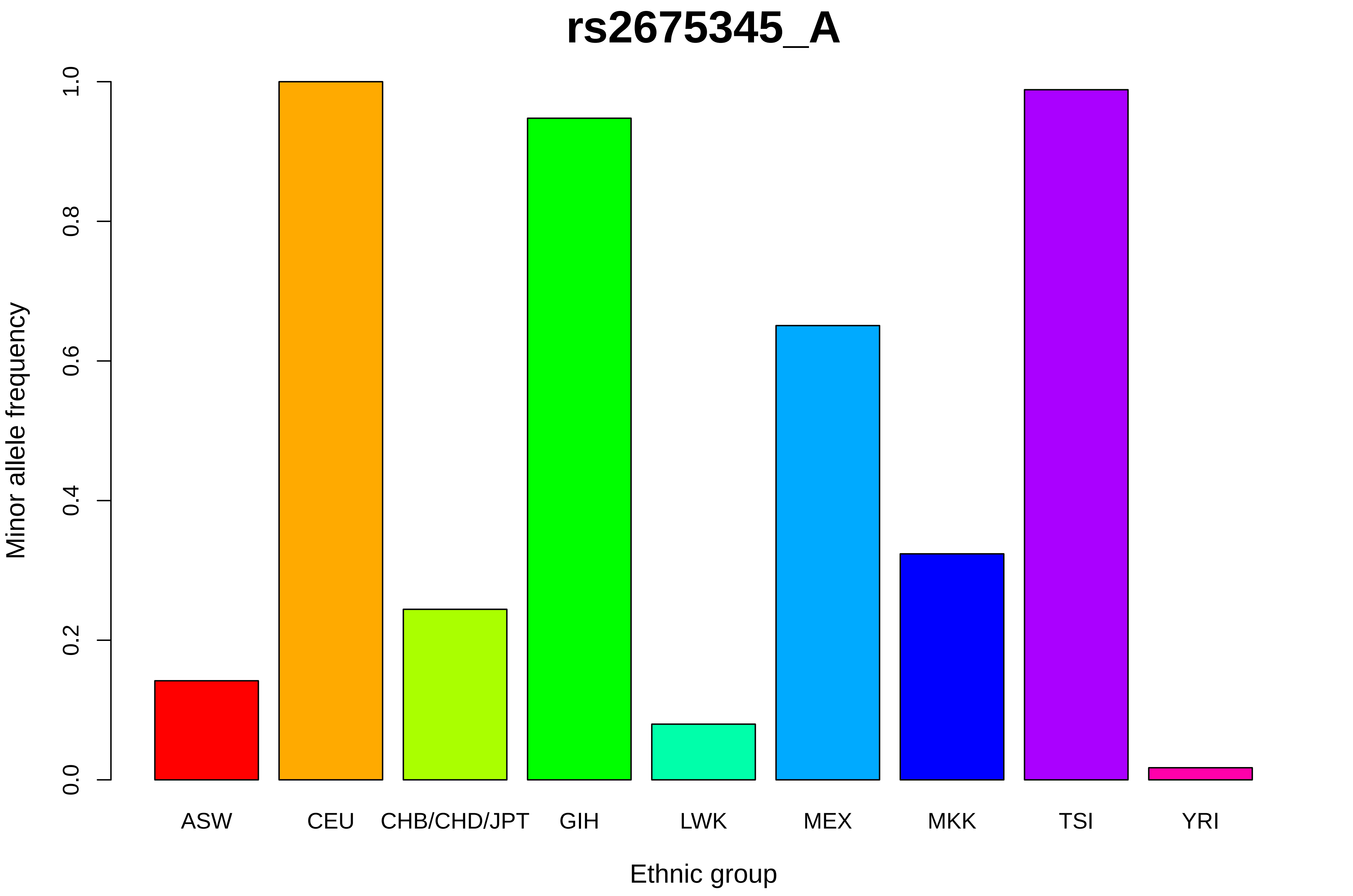
rs2675345_A
Minor allele frequency
0.0
0.2
0.4
0.6
0.8
1.0
ASW
CEU
CHB/CHD/JPT
GIH
LWK
MEX
MKK
TSI
YRI
Ethnic group

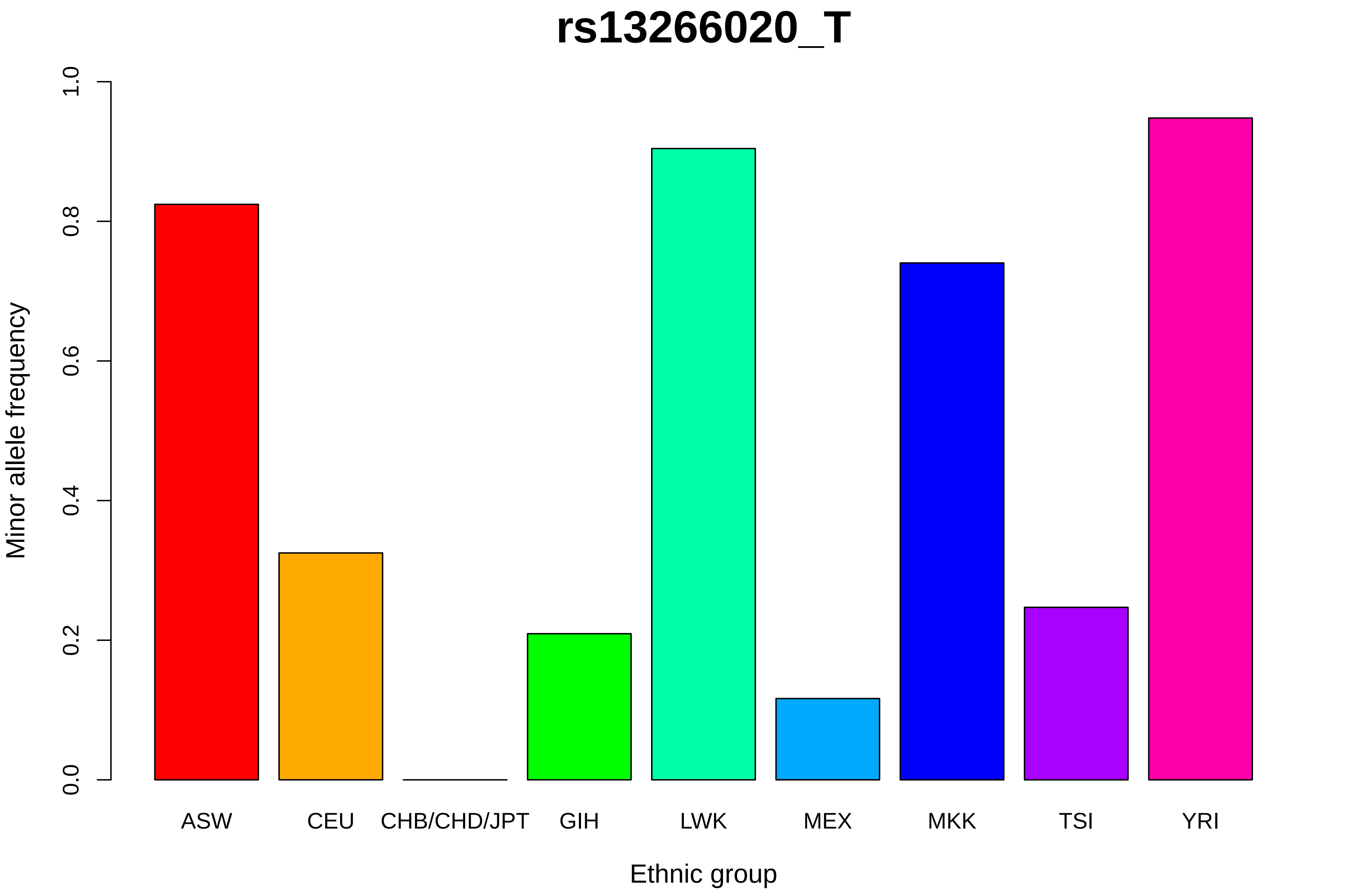
rs13266020_T
Minor allele frequency
0.0
0.2
0.4
0.6
0.8
1.0
ASW
CEU
CHB/CHD/JPT
GIH
LWK
MEX
MKK
TSI
YRI
Ethnic group

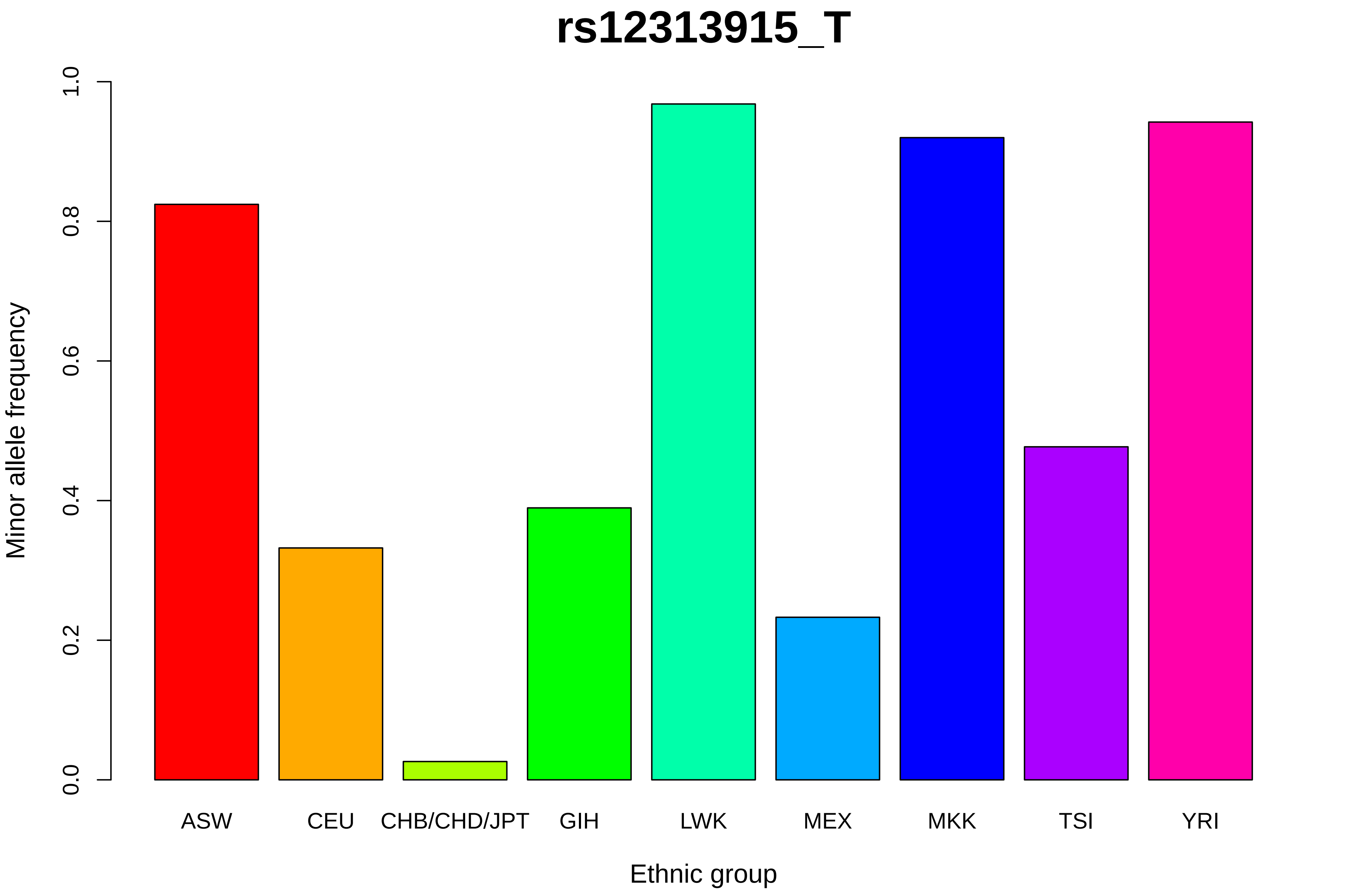
rs12313915_T
Minor allele frequency
0.0
0.2
0.4
0.6
0.8
1.0
ASW
CEU
CHB/CHD/JPT
GIH
LWK
MEX
MKK
TSI
YRI
Ethnic group

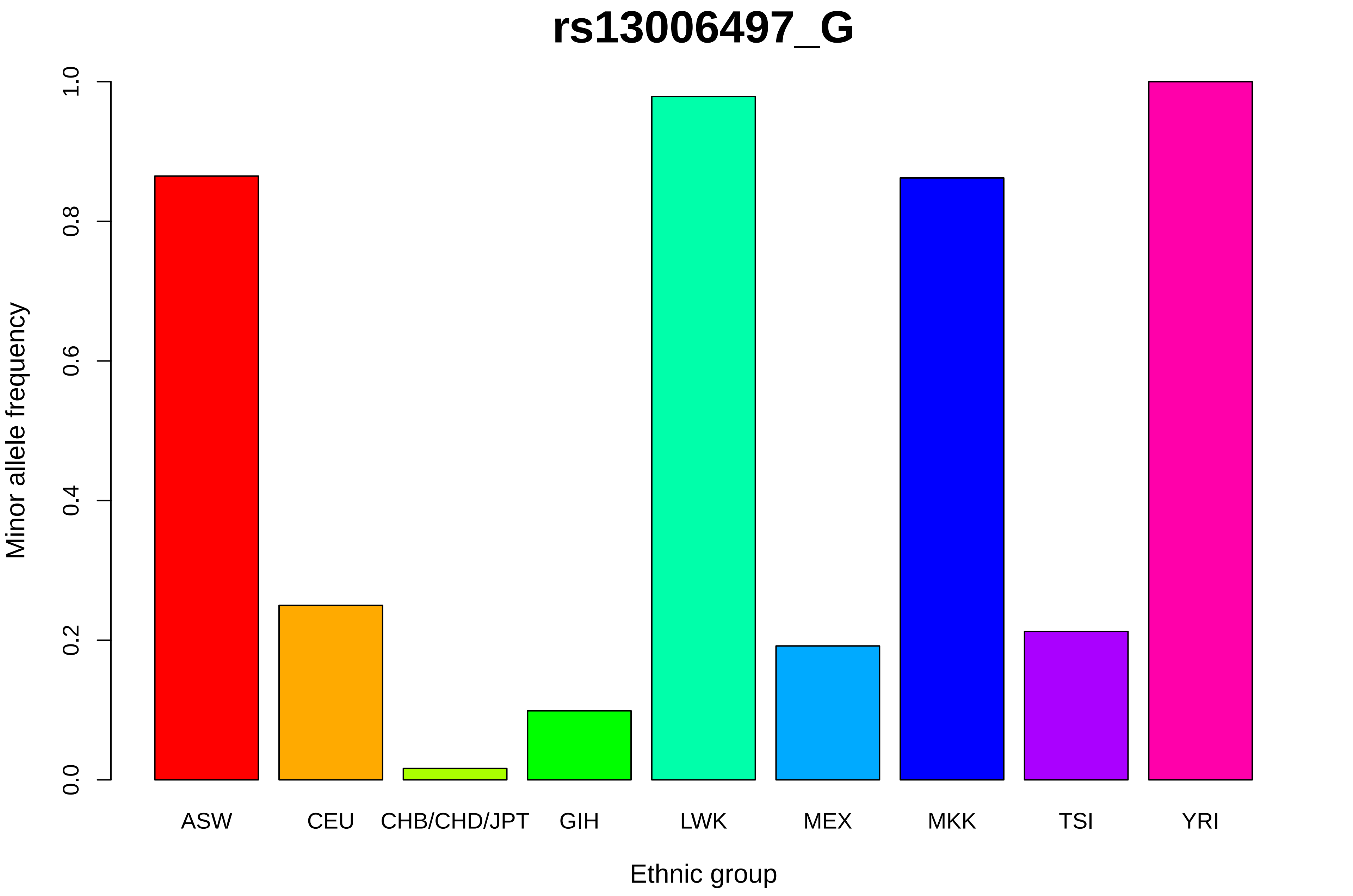
rs13006497_G
Minor allele frequency
0.0
0.2
0.4
0.6
0.8
1.0
ASW
CEU
CHB/CHD/JPT
GIH
LWK
MEX
MKK
TSI
YRI
Ethnic group

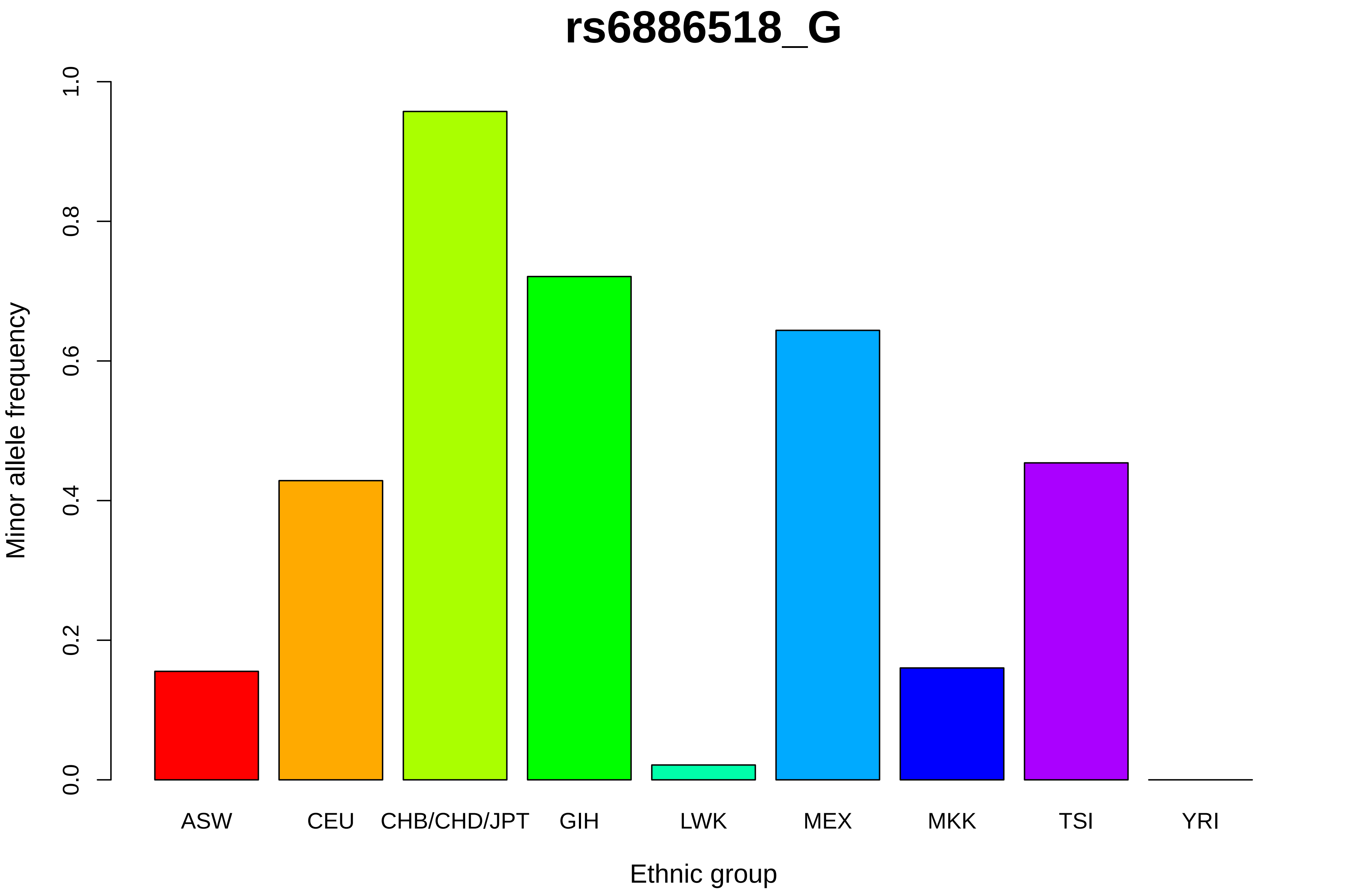
rs6886518_G
Minor allele frequency
0.0
0.2
0.4
0.6
0.8
1.0
ASW
CEU
CHB/CHD/JPT
GIH
LWK
MEX
MKK
TSI
YRI
Ethnic group

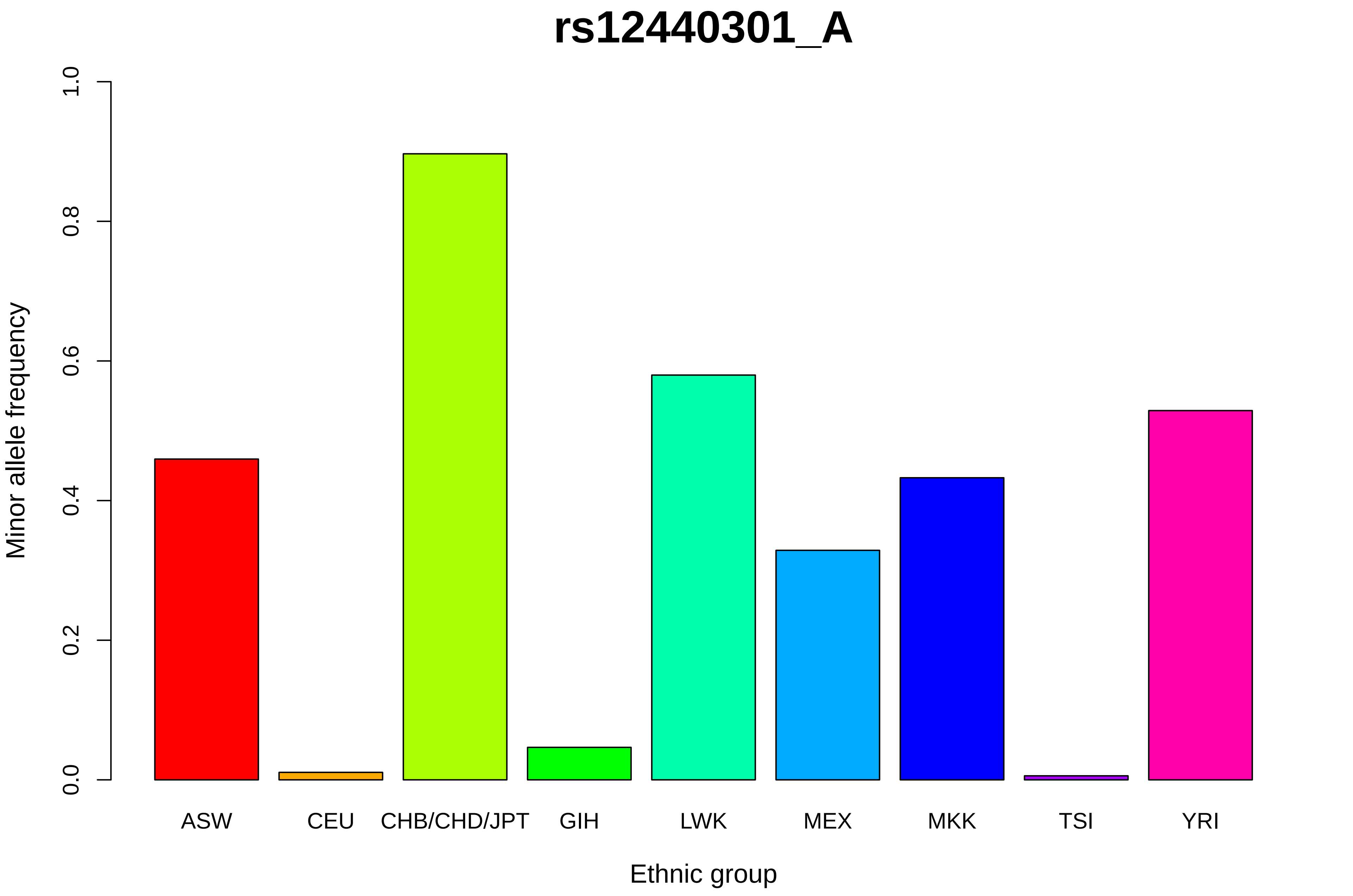

rs12440301_A
Minor allele frequency
0.0
0.2
0.4
0.6
0.8
1.0
ASW
CEU
CHB/CHD/JPT
GIH
LWK
MEX
MKK
TSI
YRI
Ethnic group

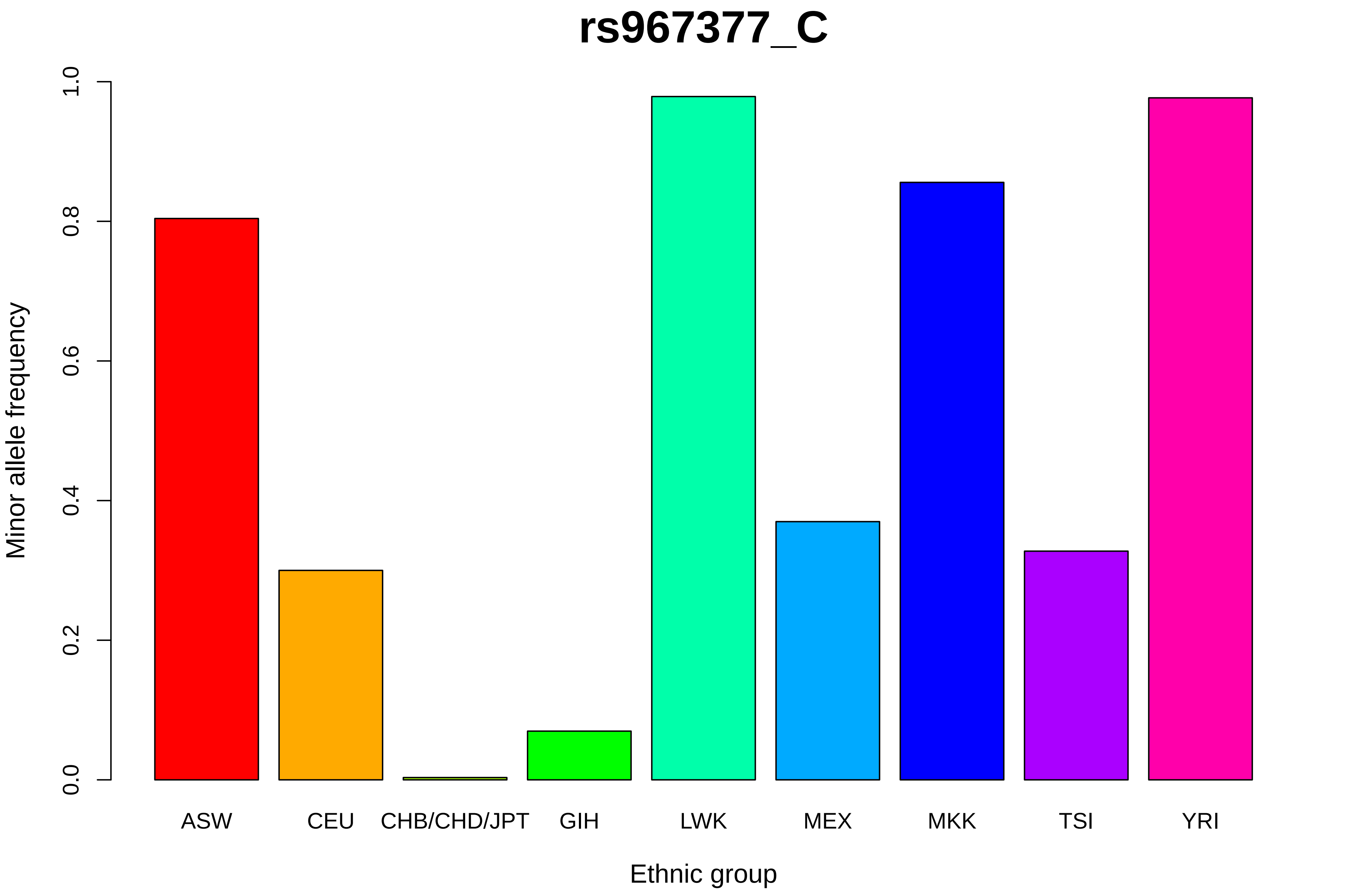
rs967377_C
Minor allele frequency
0.0
0.2
0.4
0.6
0.8
1.0
ASW
CEU
CHB/CHD/JPT
GIH
LWK
MEX
MKK
TSI
YRI
Ethnic group

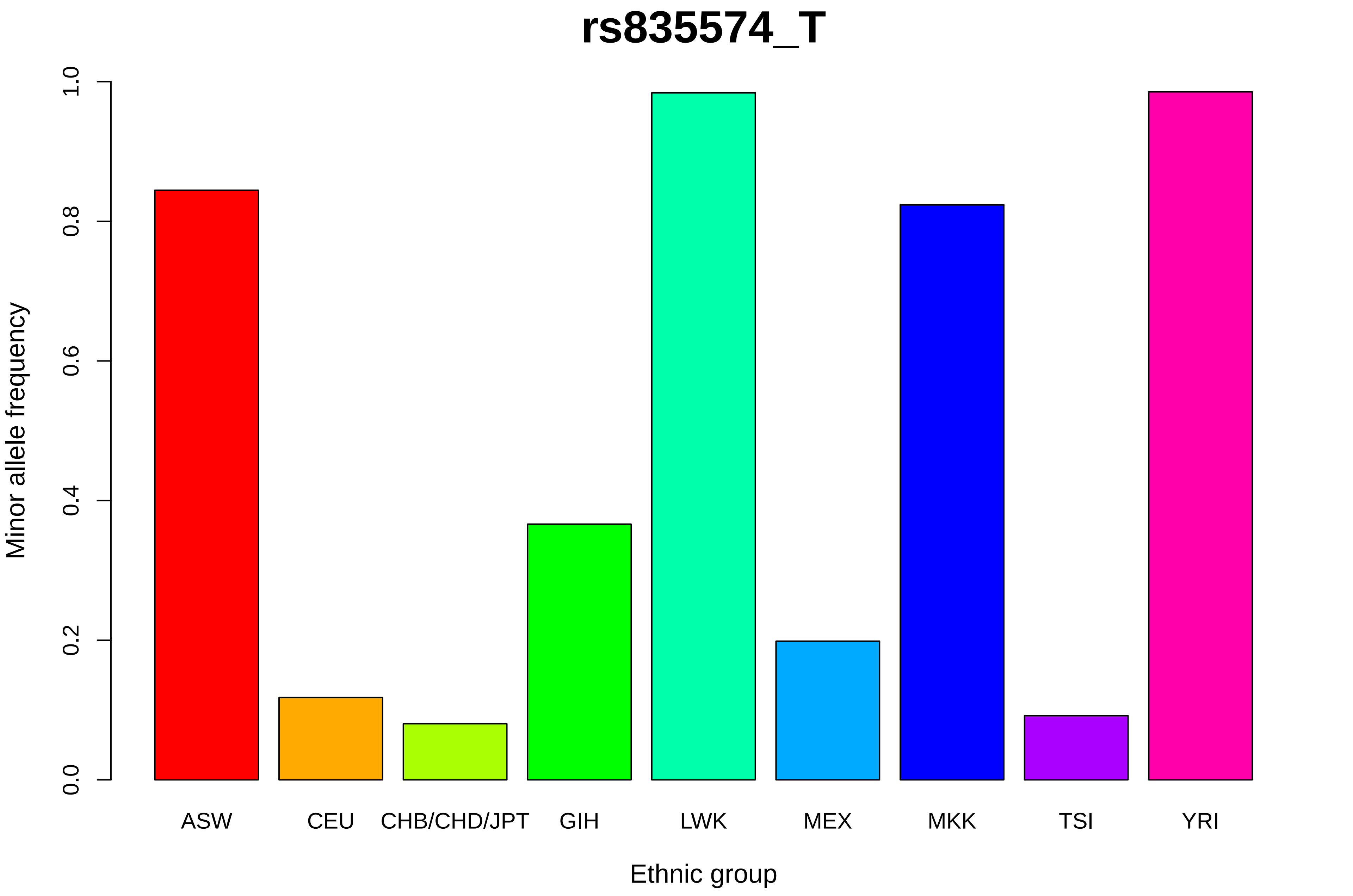
rs835574_T
Minor allele frequency
0.0
0.2
0.4
0.6
0.8
1.0
ASW
CEU
CHB/CHD/JPT
GIH
LWK
MEX
MKK
TSI
YRI
Ethnic group

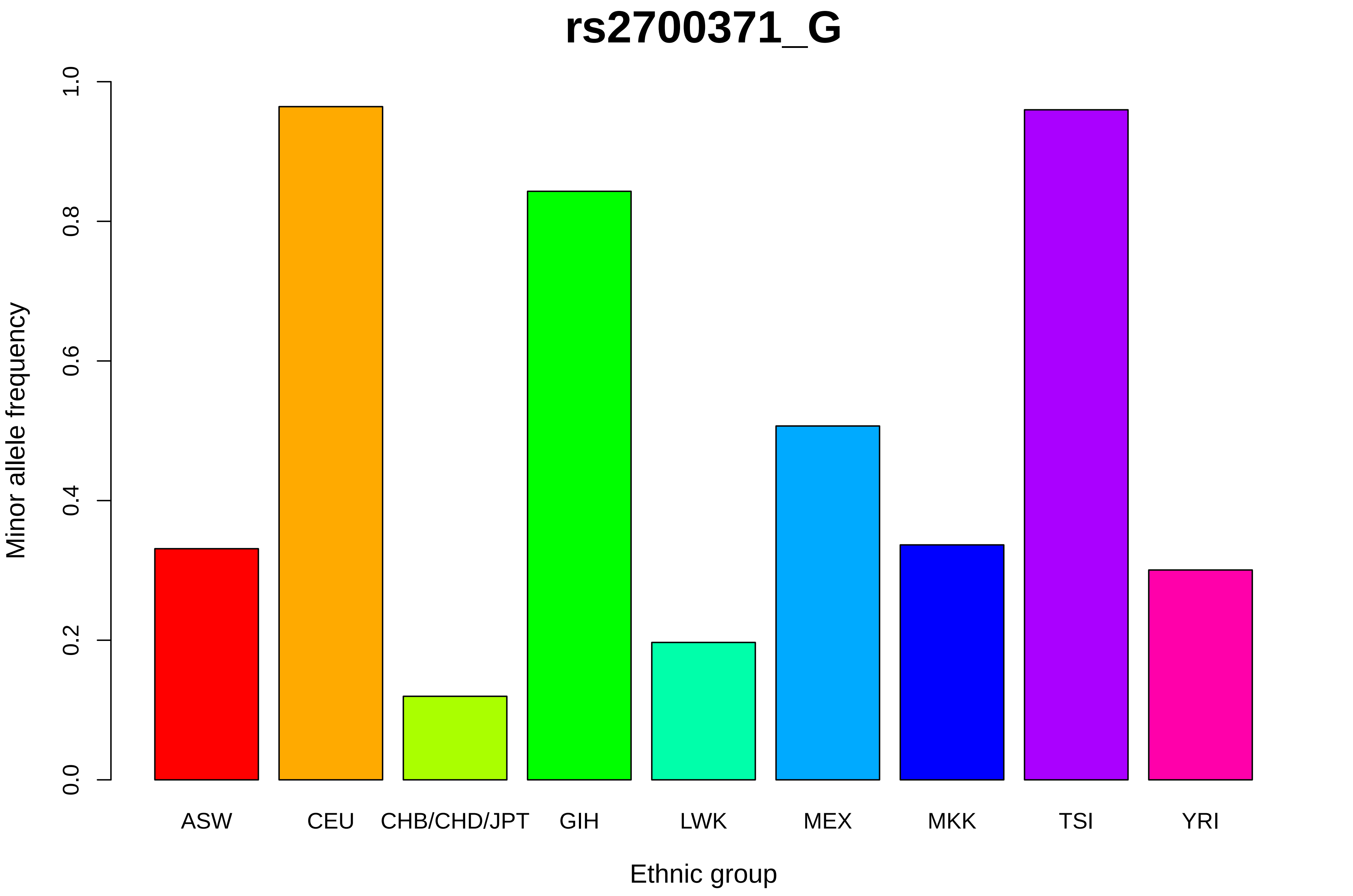
rs2700371_G
Minor allele frequency
0.0
0.2
0.4
0.6
0.8
1.0
ASW
CEU
CHB/CHD/JPT
GIH
LWK
MEX
MKK
TSI
YRI
Ethnic group

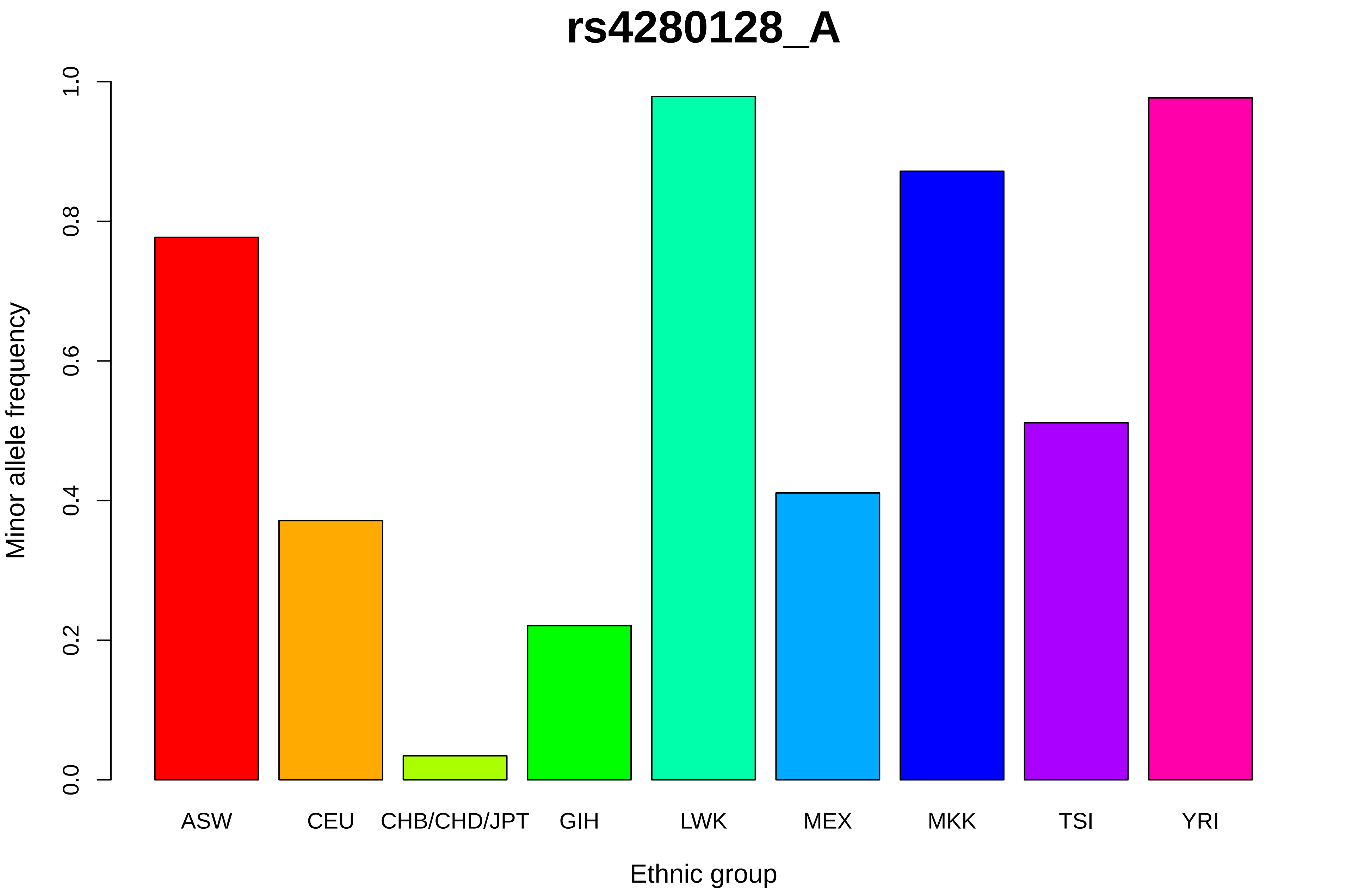
rs4280128_A
Minor allele frequency
0.0
0.2
0.4
0.6
0.8
1.0
ASW
CEU
CHB/CHD/JPT
GIH
LWK
MEX
MKK
TSI
YRI
Ethnic group

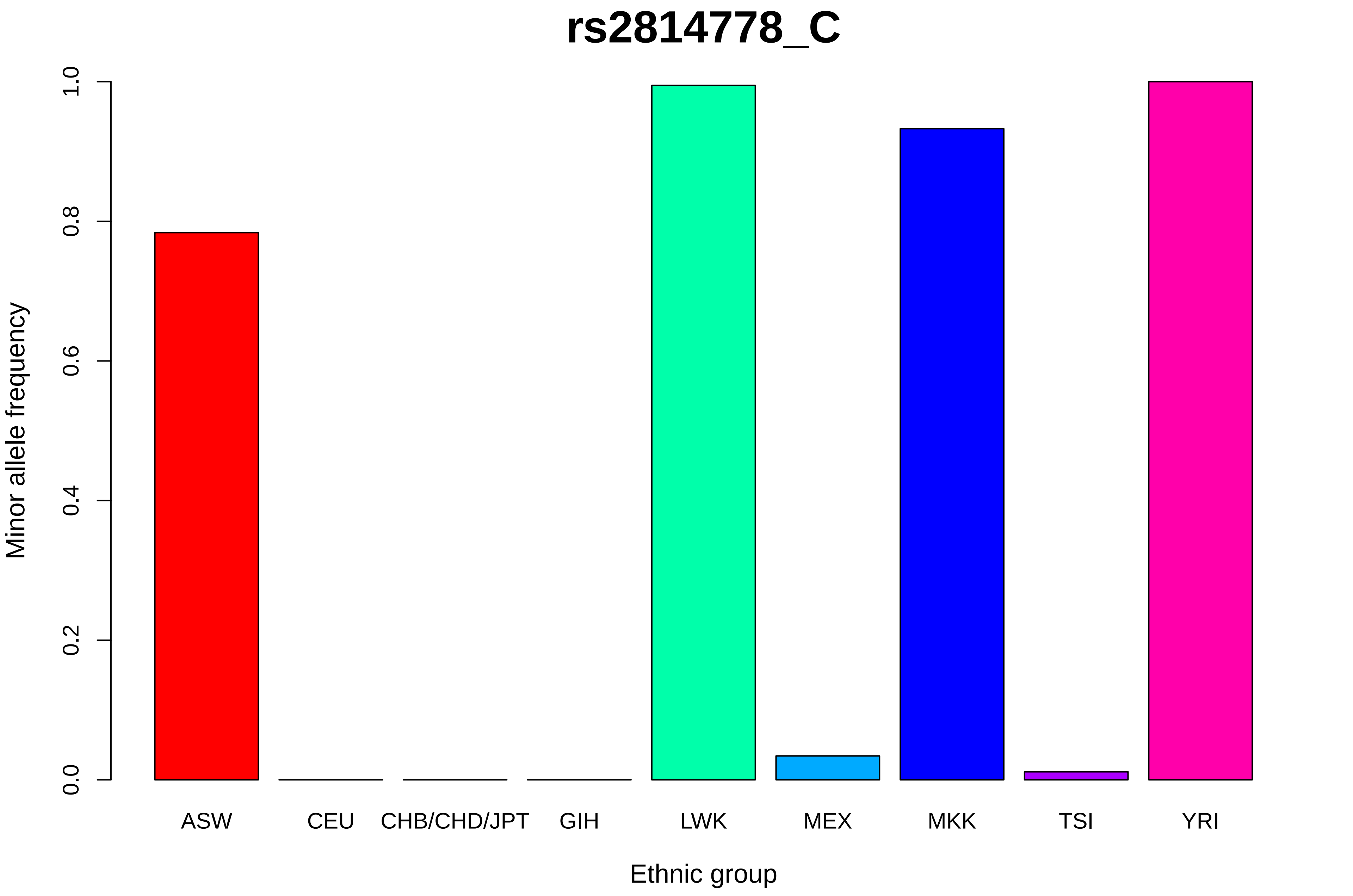
rs2814778_C
Minor allele frequency
0.0
0.2
0.4
0.6
0.8
1.0
ASW
CEU
CHB/CHD/JPT
GIH
LWK
MEX
MKK
TSI
YRI
Ethnic group

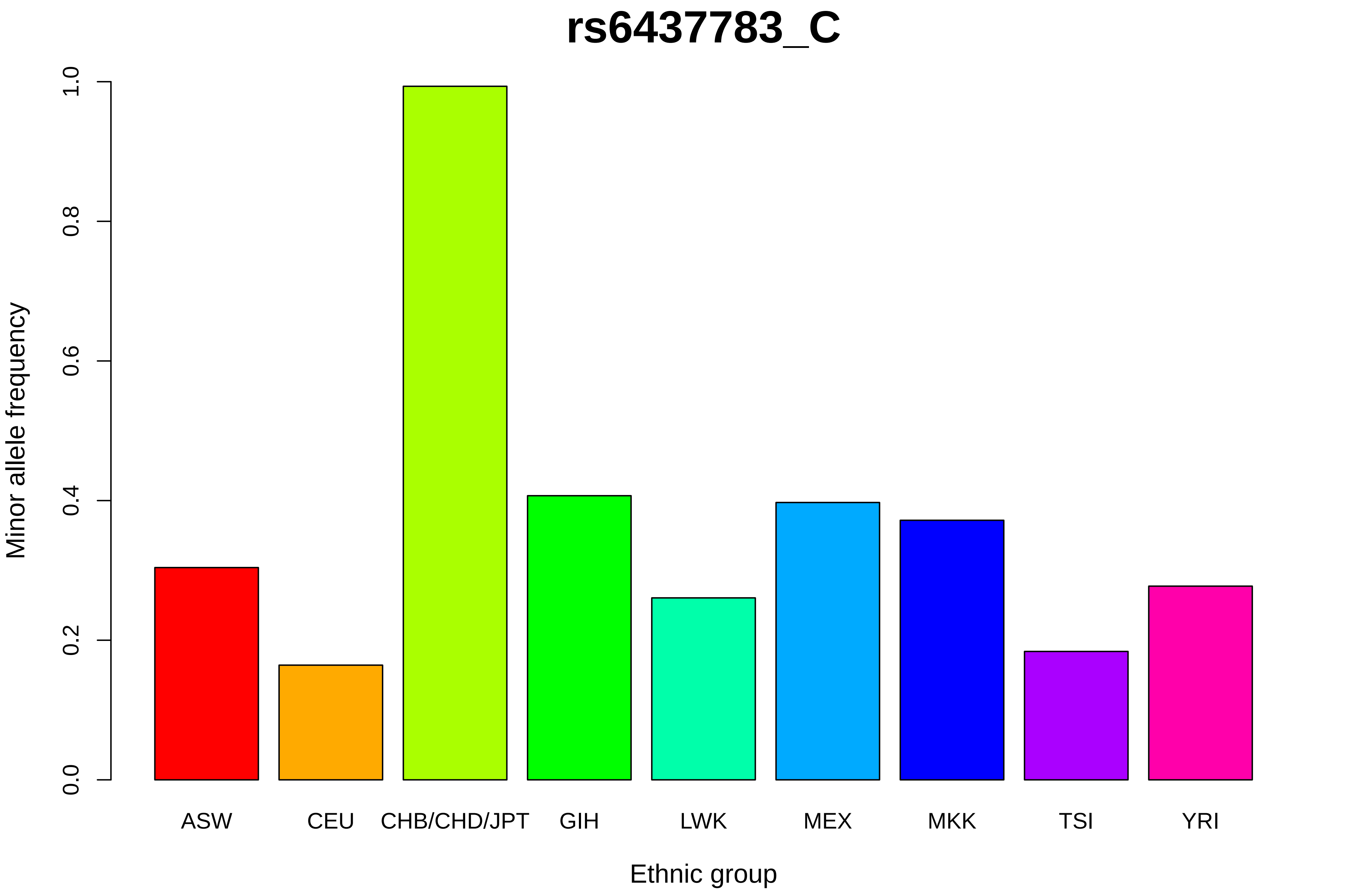
rs6437783_C
Minor allele frequency
0.0
0.2
0.4
0.6
0.8
1.0
ASW
CEU
CHB/CHD/JPT
GIH
LWK
MEX
MKK
TSI
YRI
Ethnic group

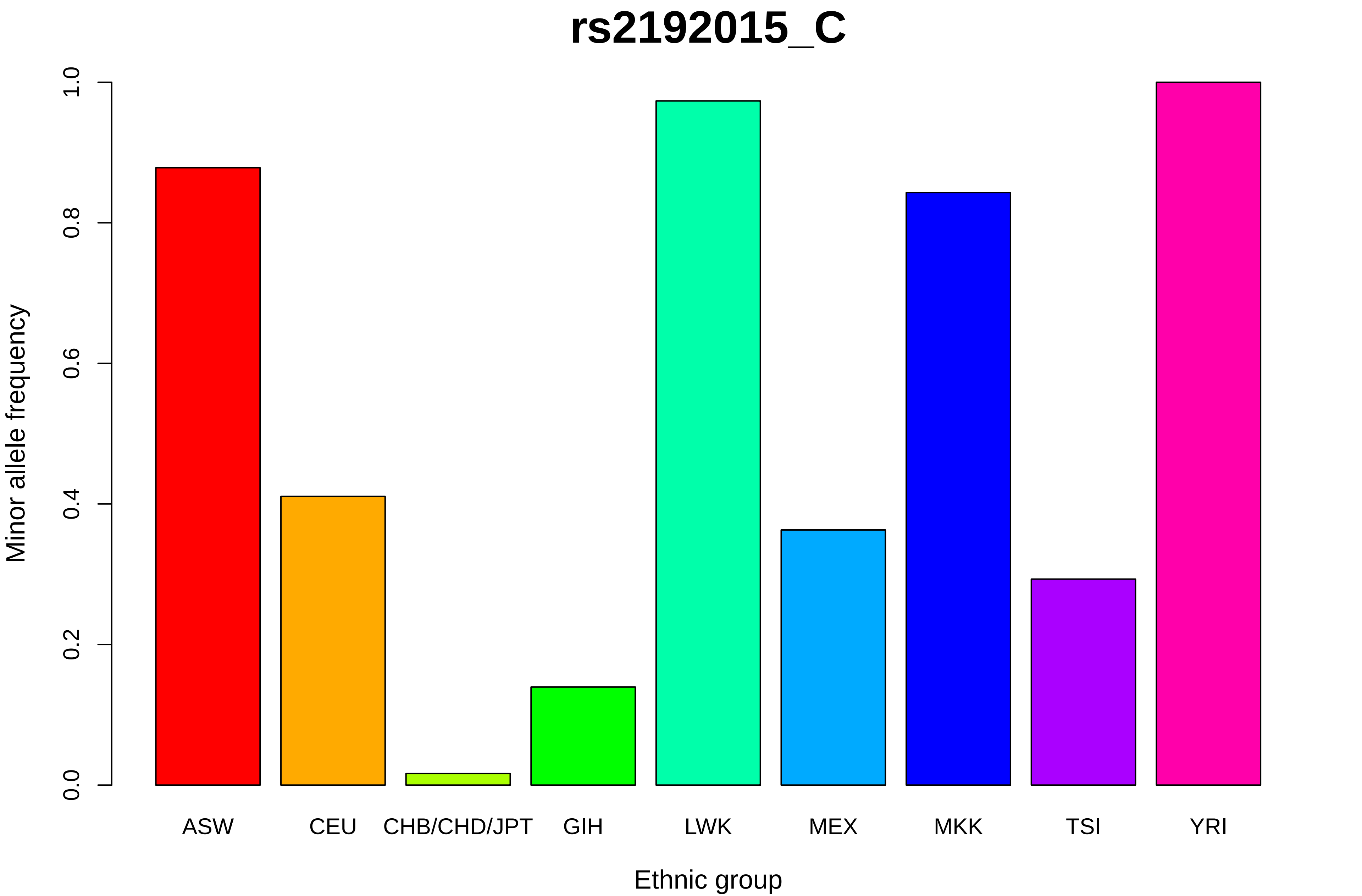

rs2192015_C
Minor allele frequency
0.0
0.2
0.4
0.6
0.8
1.0
ASW
CEU
CHB/CHD/JPT
GIH
LWK
MEX
MKK
TSI
YRI
Ethnic group

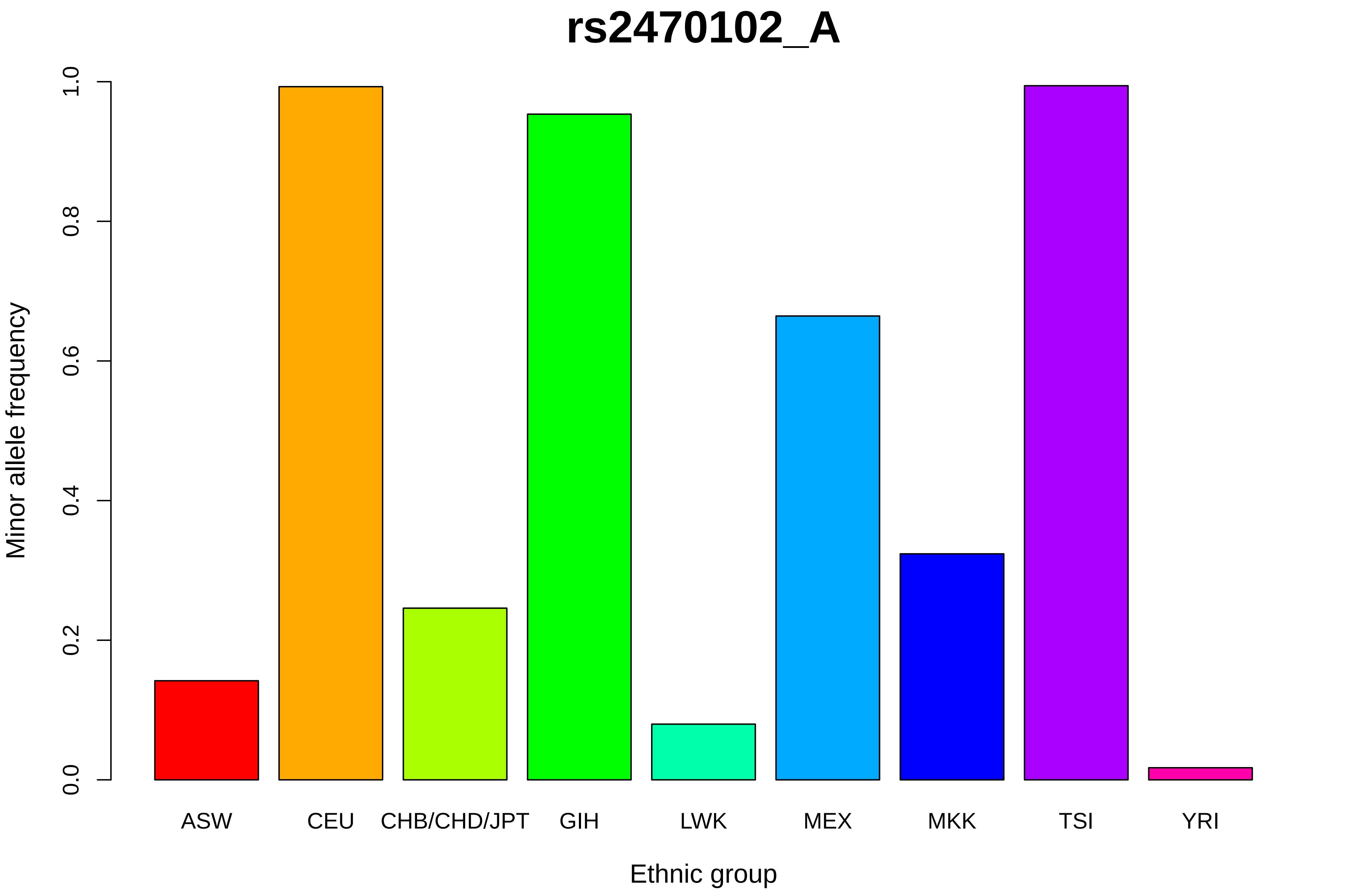

rs2470102_A
Minor allele frequency
0.0
0.2
0.4
0.6
0.8
1.0
ASW
CEU
CHB/CHD/JPT
GIH
LWK
MEX
MKK
TSI
YRI
Ethnic group

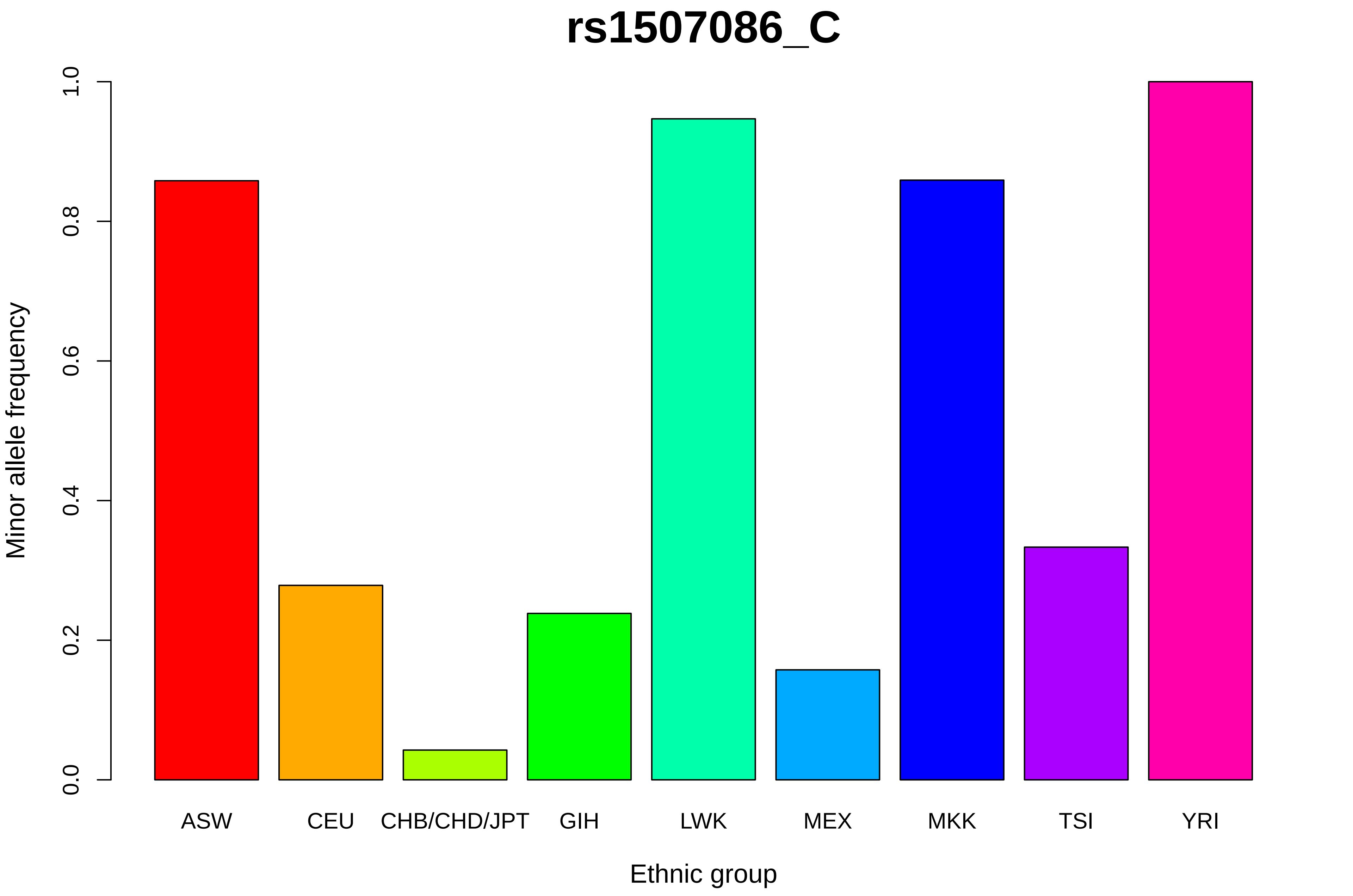
rs1507086_C
Minor allele frequency
0.0
0.2
0.4
0.6
0.8
1.0
ASW
CEU
CHB/CHD/JPT
GIH
LWK
MEX
MKK
TSI
YRI
Ethnic group

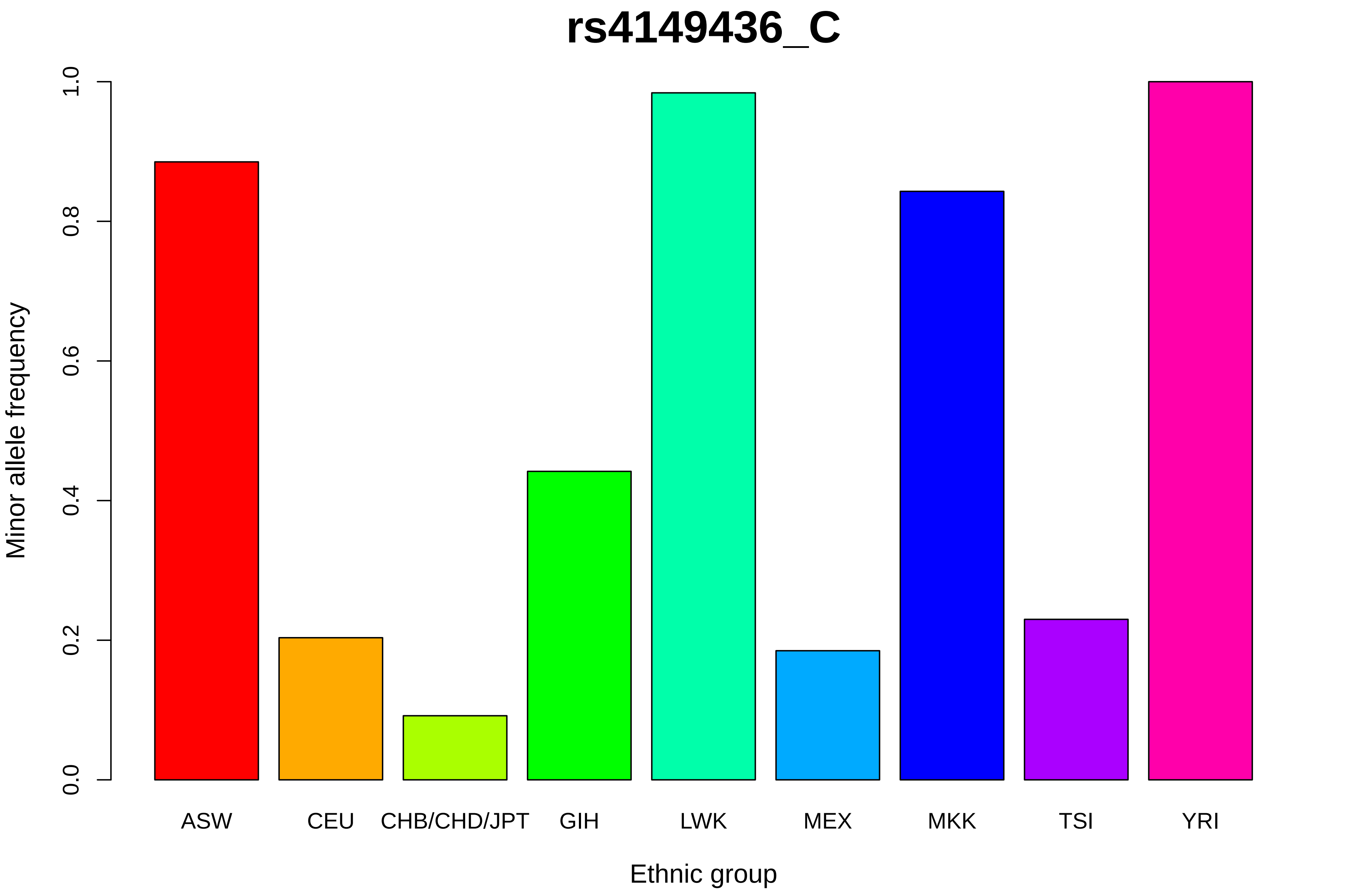
rs4149436_C
Minor allele frequency
0.0
0.2
0.4
0.6
0.8
1.0
ASW
CEU
CHB/CHD/JPT
GIH
LWK
MEX
MKK
TSI
YRI
Ethnic group

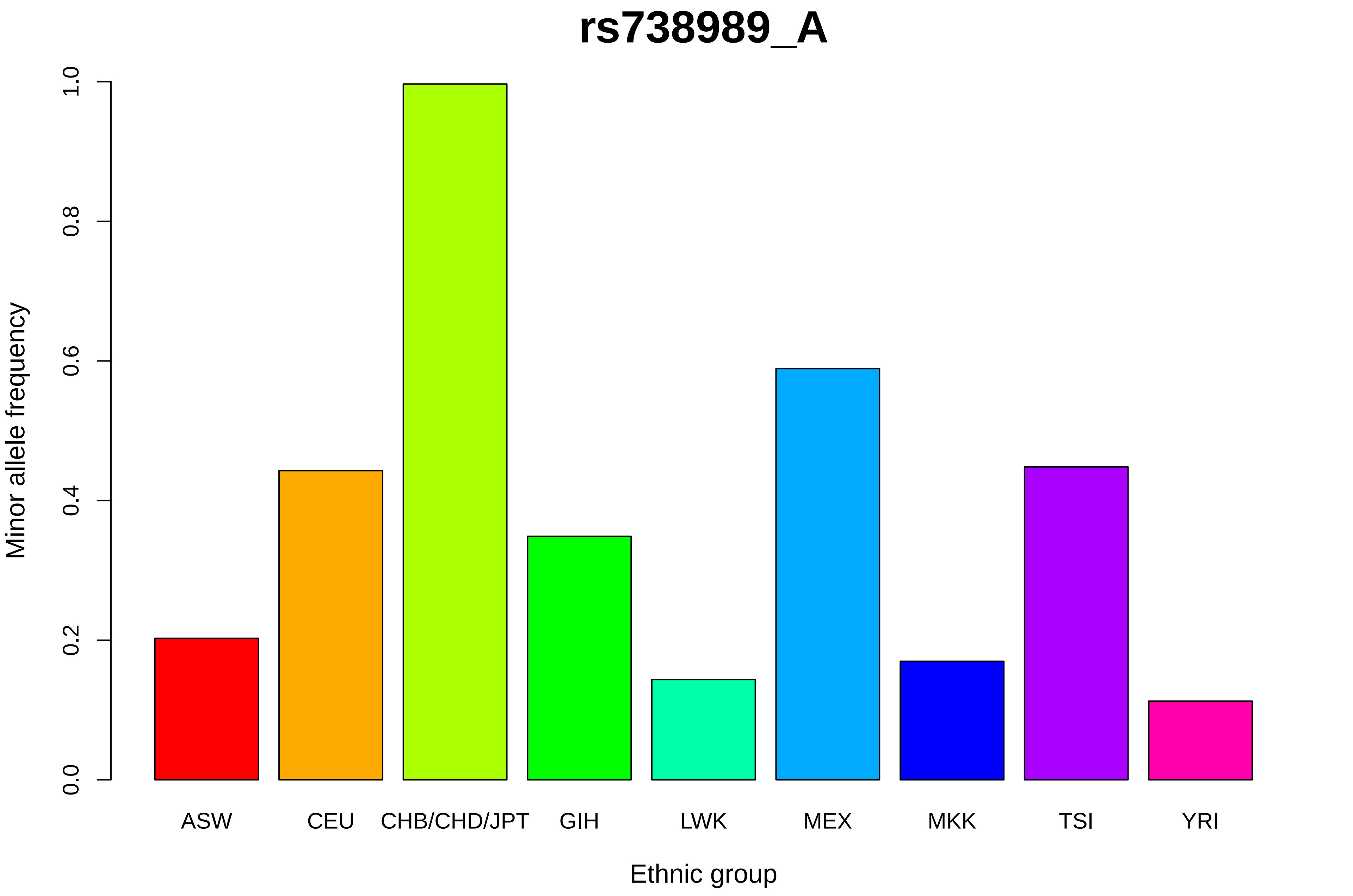
rs738989_A
Minor allele frequency
0.0
0.2
0.4
0.6
0.8
1.0
ASW
CEU
CHB/CHD/JPT
GIH
LWK
MEX
MKK
TSI
YRI
Ethnic group

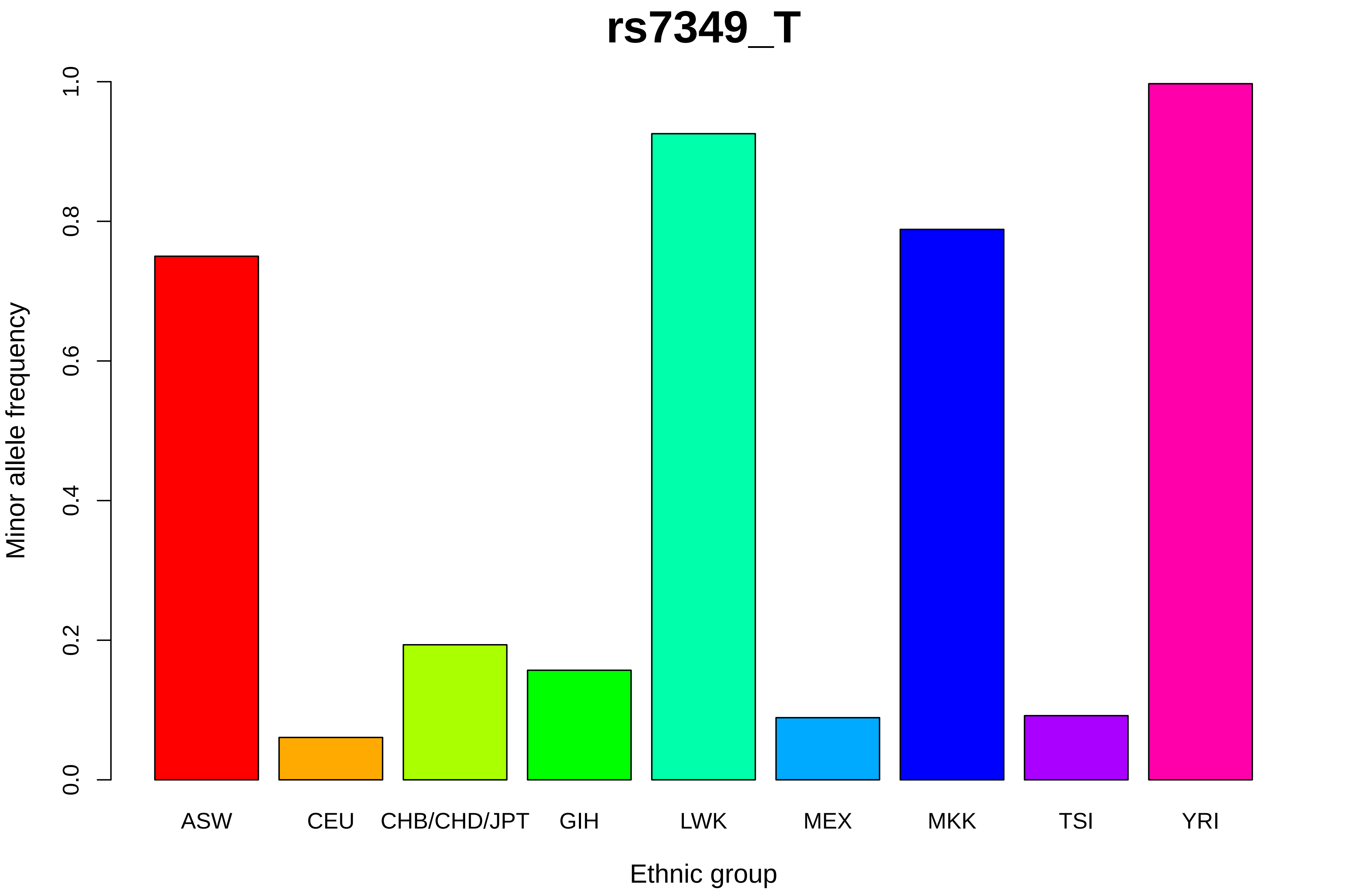

rs7349_T
Minor allele frequency
0.0
0.2
0.4
0.6
0.8
1.0
ASW
CEU
CHB/CHD/JPT
GIH
LWK
MEX
MKK
TSI
YRI
Ethnic group

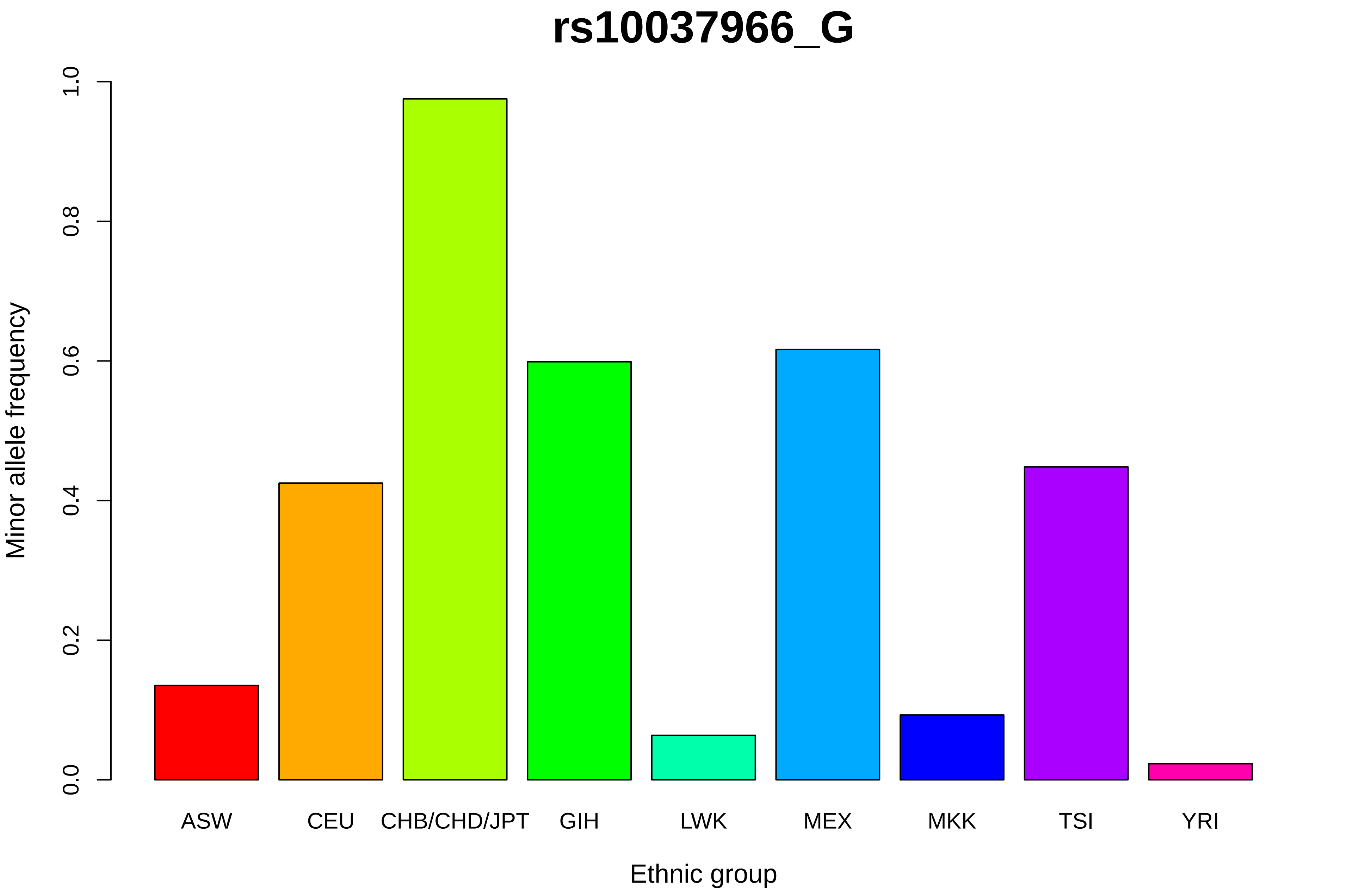
rs10037966_G
Minor allele frequency
0.0
0.2
0.4
0.6
0.8
1.0
ASW
CEU
CHB/CHD/JPT
GIH
LWK
MEX
MKK
TSI
YRI
Ethnic group

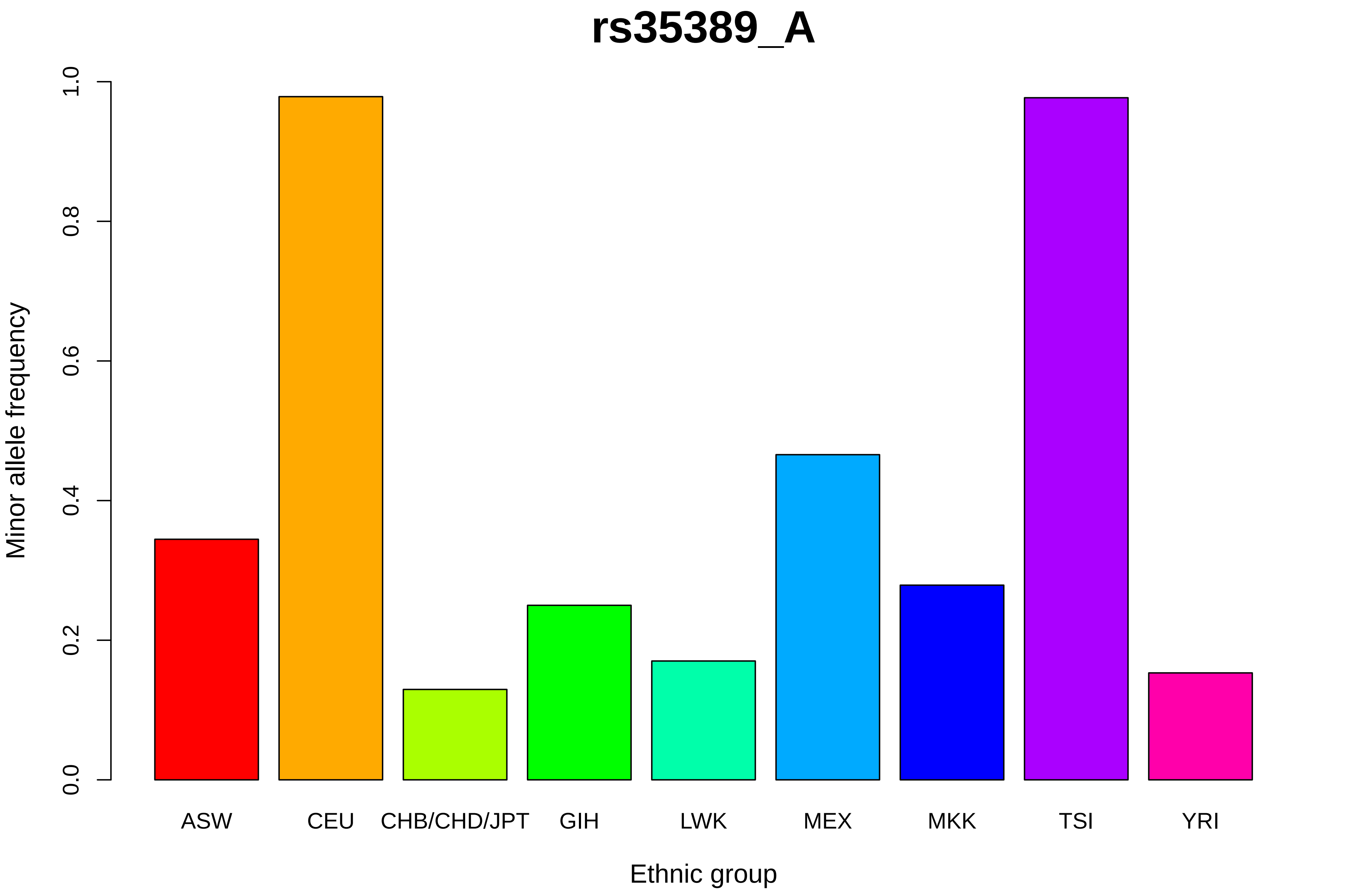
rs35389_A
Minor allele frequency
0.0
0.2
0.4
0.6
0.8
1.0
ASW
CEU
CHB/CHD/JPT
GIH
LWK
MEX
MKK
TSI
YRI
Ethnic group

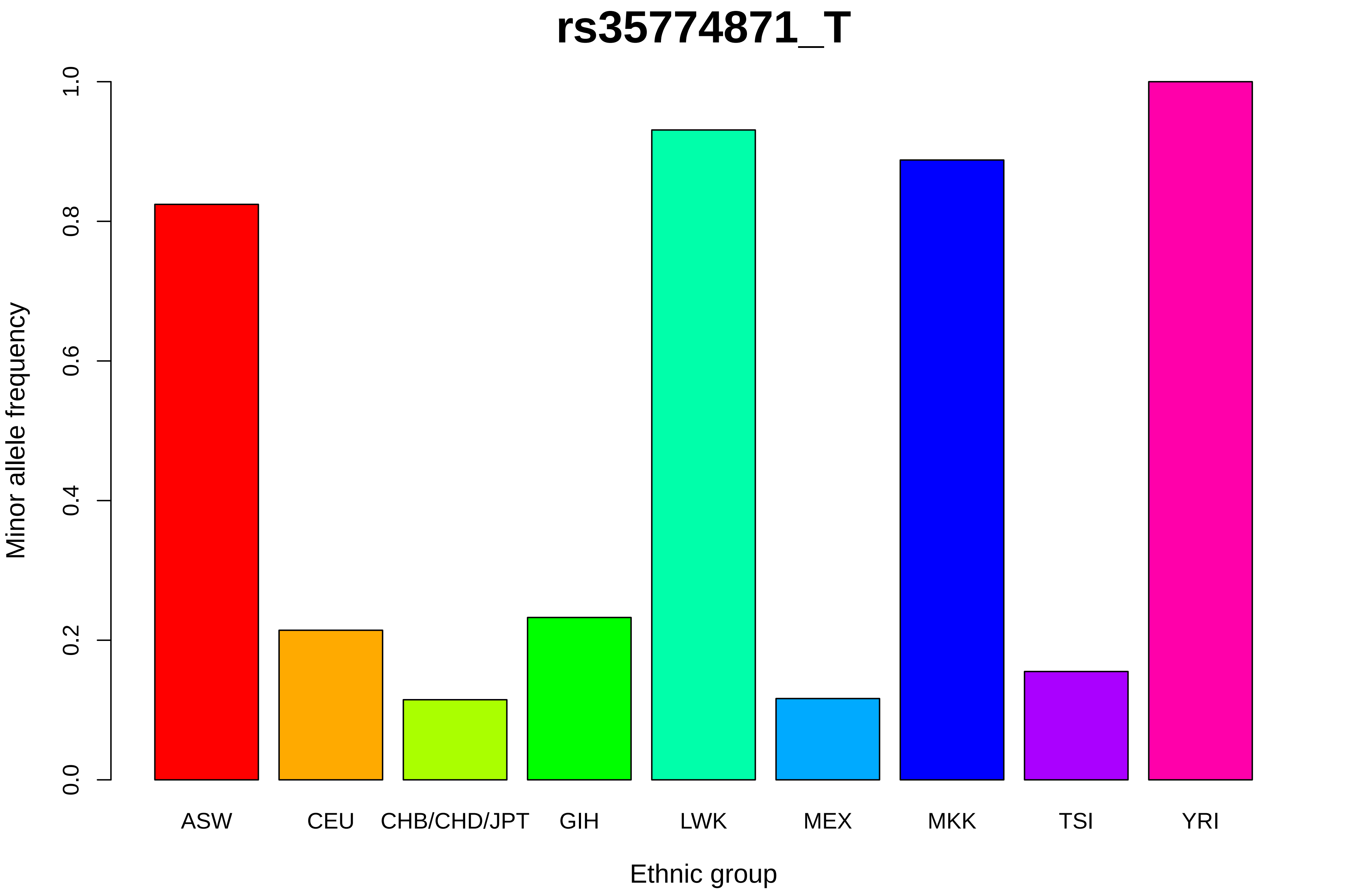

rs35774871_T
Minor allele frequency
0.0
0.2
0.4
0.6
0.8
1.0
ASW
CEU
CHB/CHD/JPT
GIH
LWK
MEX
MKK
TSI
YRI
Ethnic group

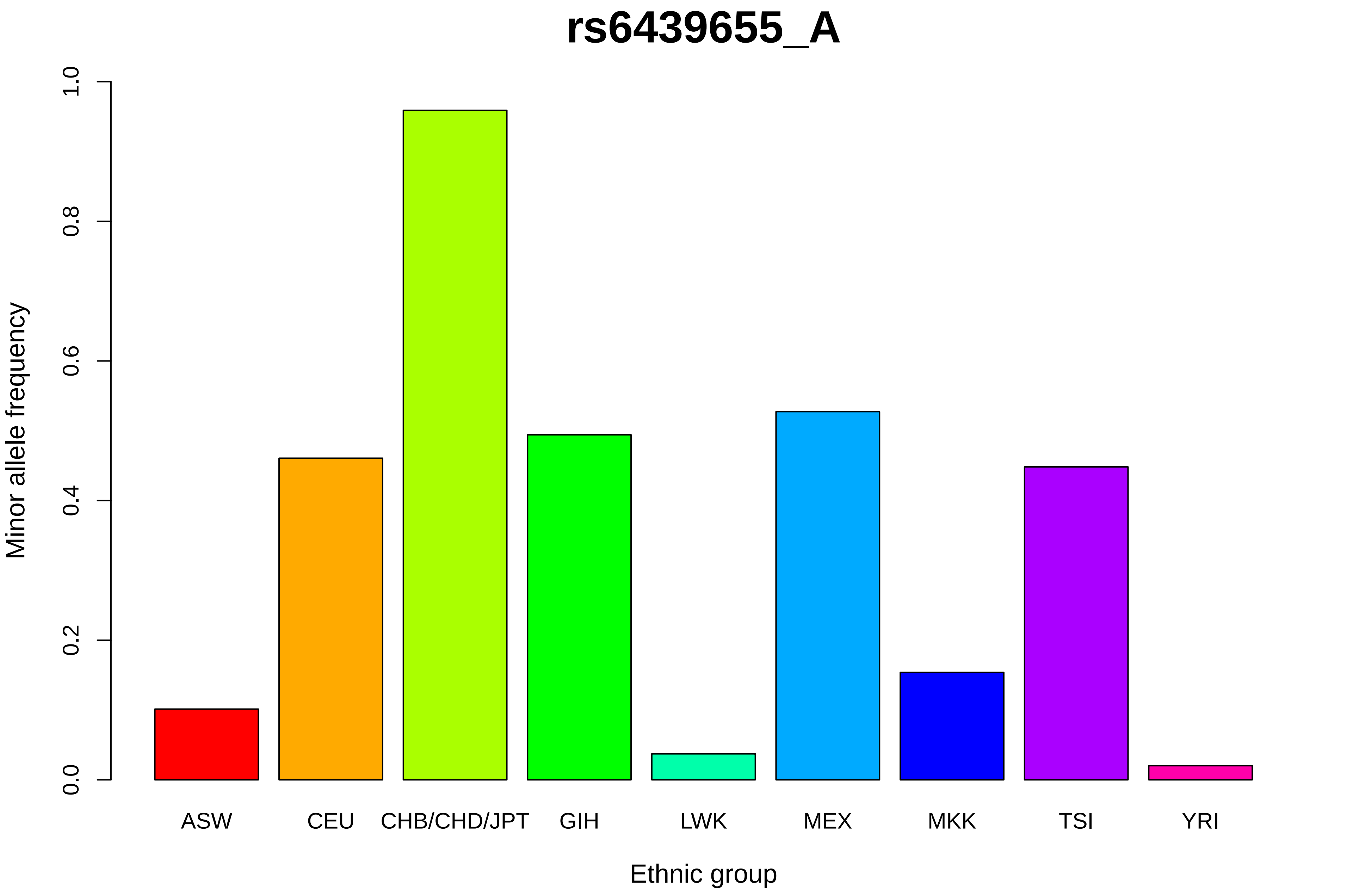
rs6439655_A
Minor allele frequency
0.0
0.2
0.4
0.6
0.8
1.0
ASW
CEU
CHB/CHD/JPT
GIH
LWK
MEX
MKK
TSI
YRI
Ethnic group

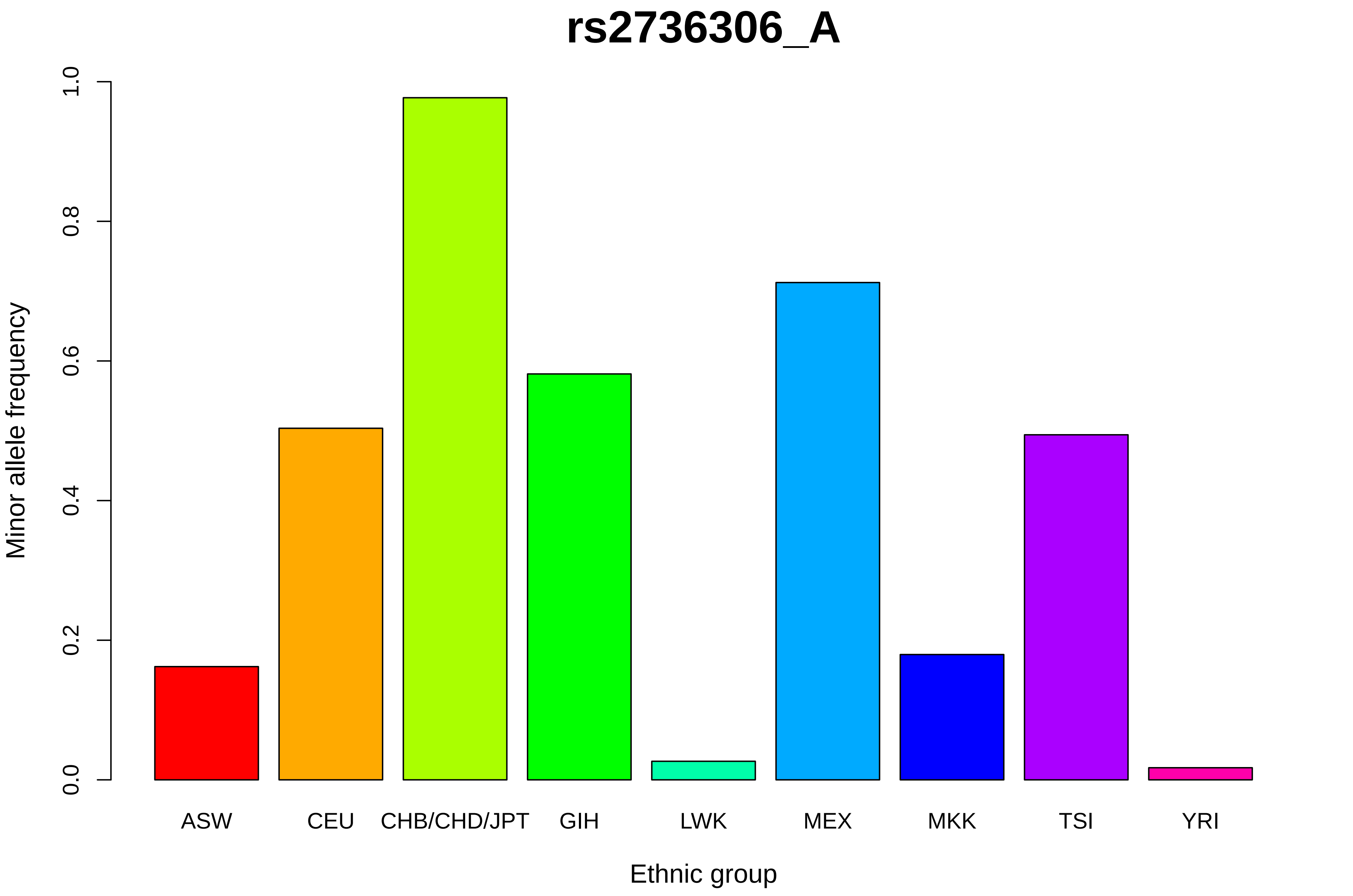
rs2736306_A
Minor allele frequency
0.0
0.2
0.4
0.6
0.8
1.0
ASW
CEU
CHB/CHD/JPT
GIH
LWK
MEX
MKK
TSI
YRI
Ethnic group

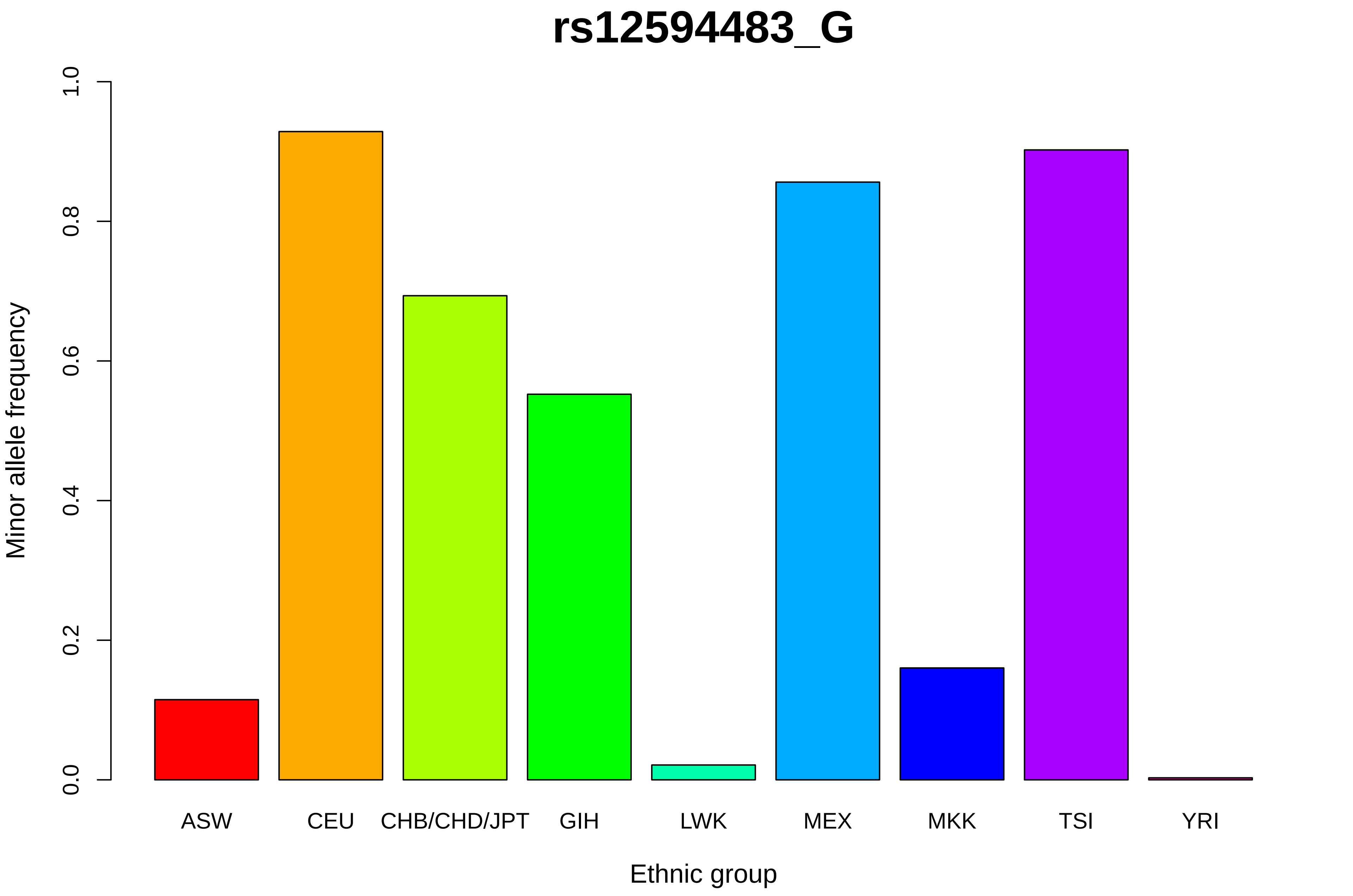

rs12594483_G
Minor allele frequency
0.0
0.2
0.4
0.6
0.8
1.0
ASW
CEU
CHB/CHD/JPT
GIH
LWK
MEX
MKK
TSI
YRI
Ethnic group

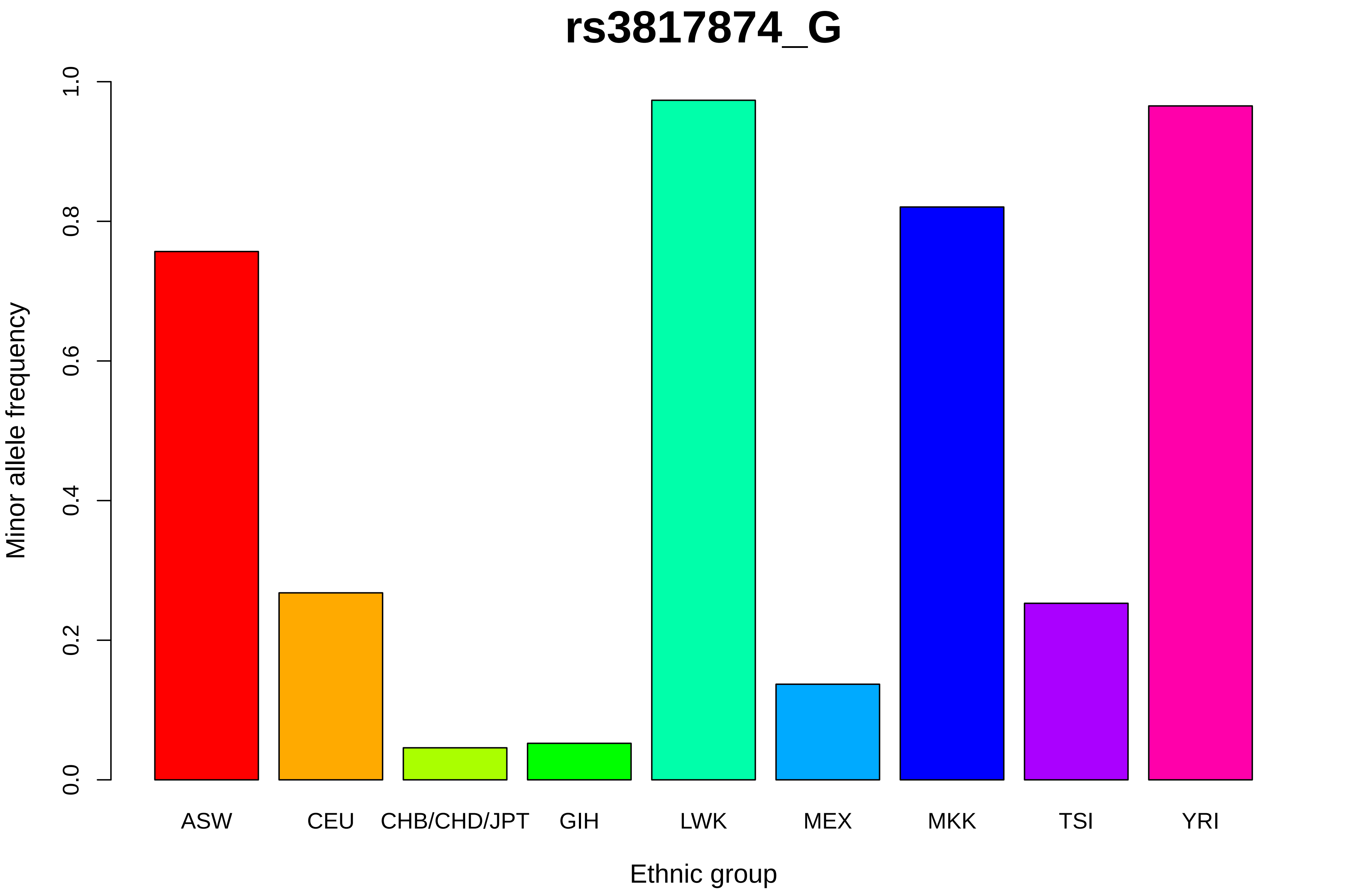
rs3817874_G
Minor allele frequency
0.0
0.2
0.4
0.6
0.8
1.0
ASW
CEU
CHB/CHD/JPT
GIH
LWK
MEX
MKK
TSI
YRI
Ethnic group

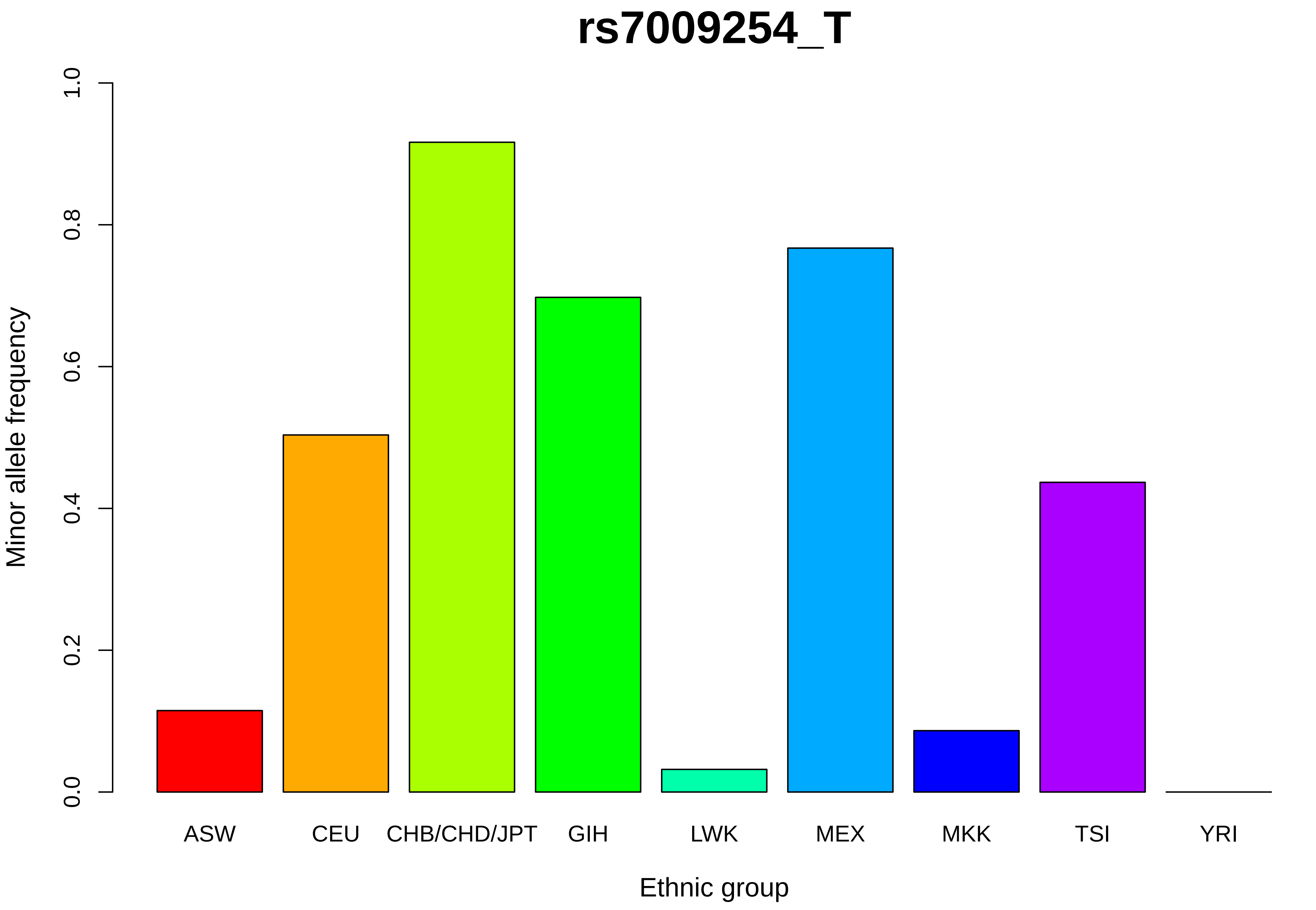
rs7009254_T
Minor allele frequency
0.0
0.2
0.4
0.6
0.8
1.0
ASW
CEU
CHB/CHD/JPT
GIH
LWK
MEX
MKK
TSI
YRI
Ethnic group

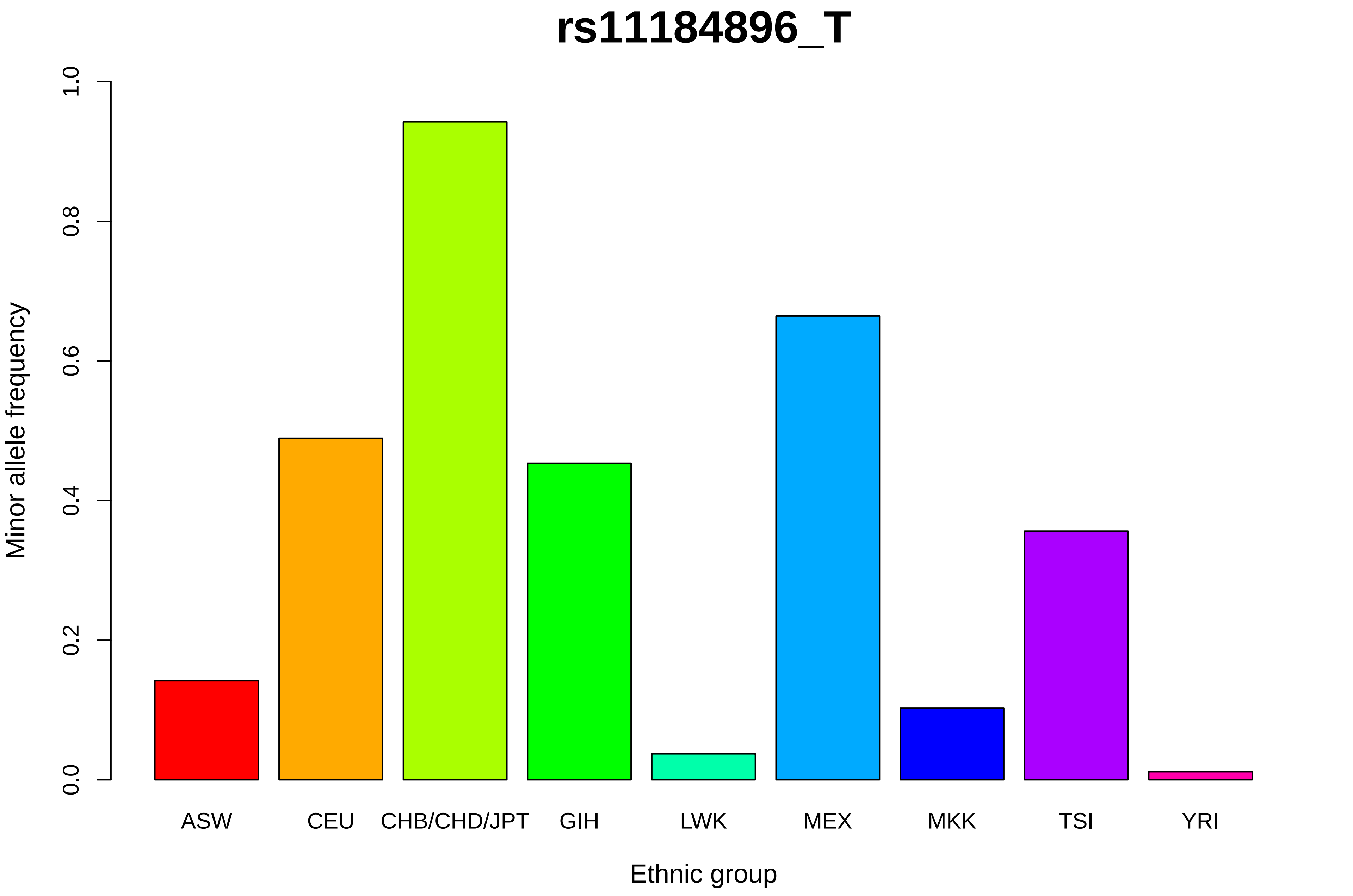
rs11184896_T
Minor allele frequency
0.0
0.2
0.4
0.6
0.8
1.0
ASW
CEU
CHB/CHD/JPT
GIH
LWK
MEX
MKK
TSI
YRI
Ethnic group

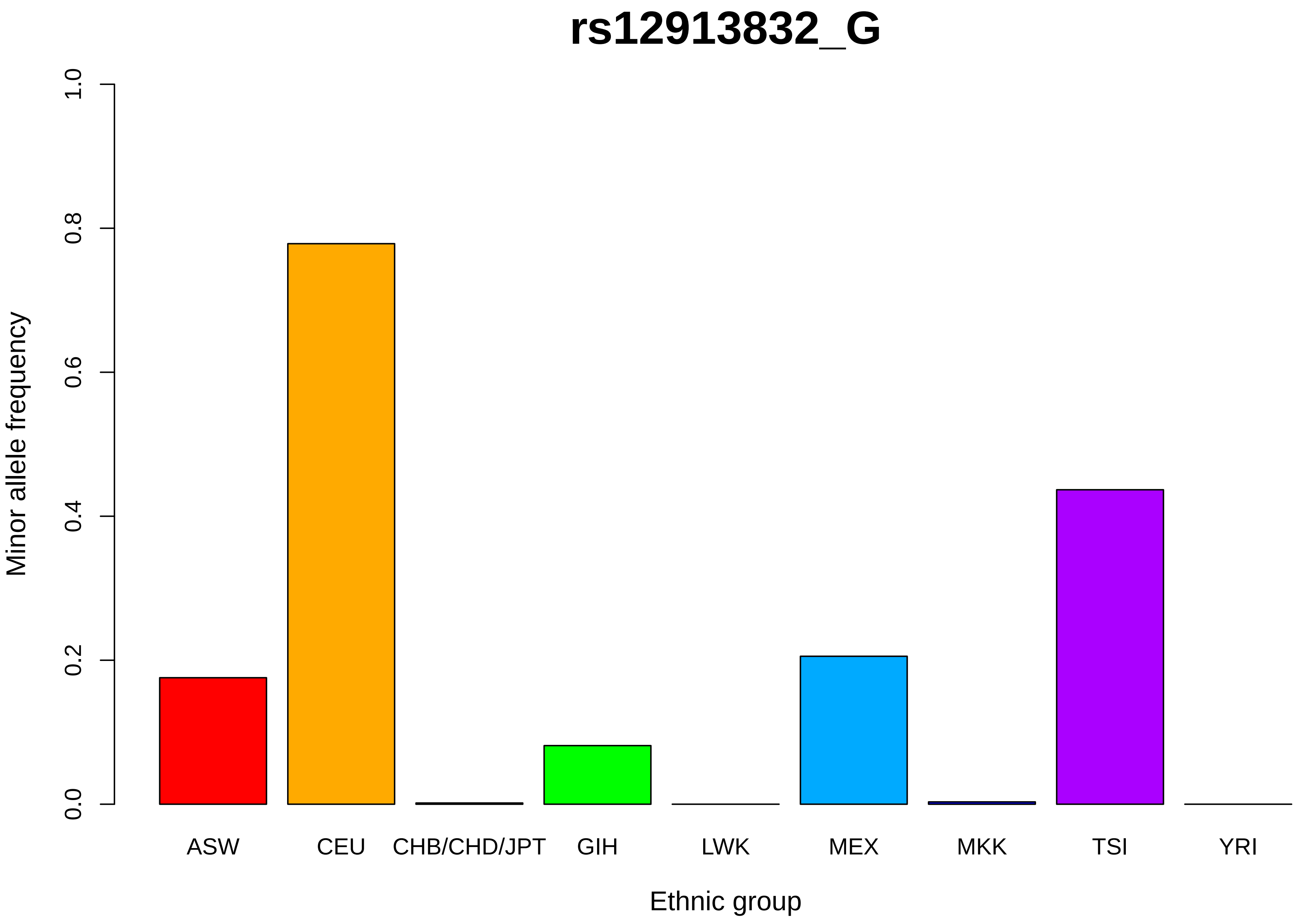
rs12913832_G
Minor allele frequency
0.0
0.2
0.4
0.6
0.8
1.0
ASW
CEU
CHB/CHD/JPT
GIH
LWK
MEX
MKK
TSI
YRI
Ethnic group

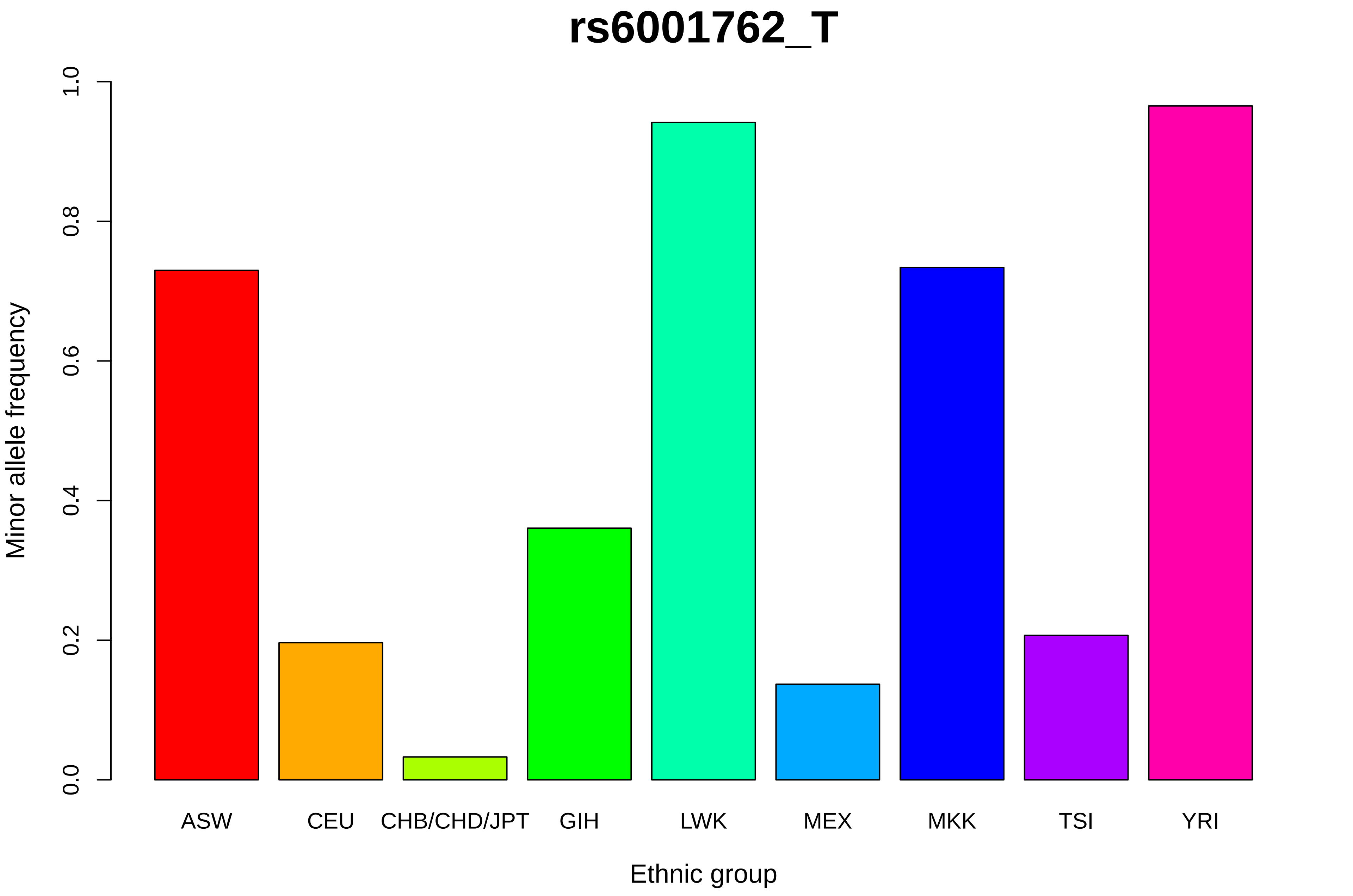
rs6001762_T
Minor allele frequency
0.0
0.2
0.4
0.6
0.8
1.0
ASW
CEU
CHB/CHD/JPT
GIH
LWK
MEX
MKK
TSI
YRI
Ethnic group

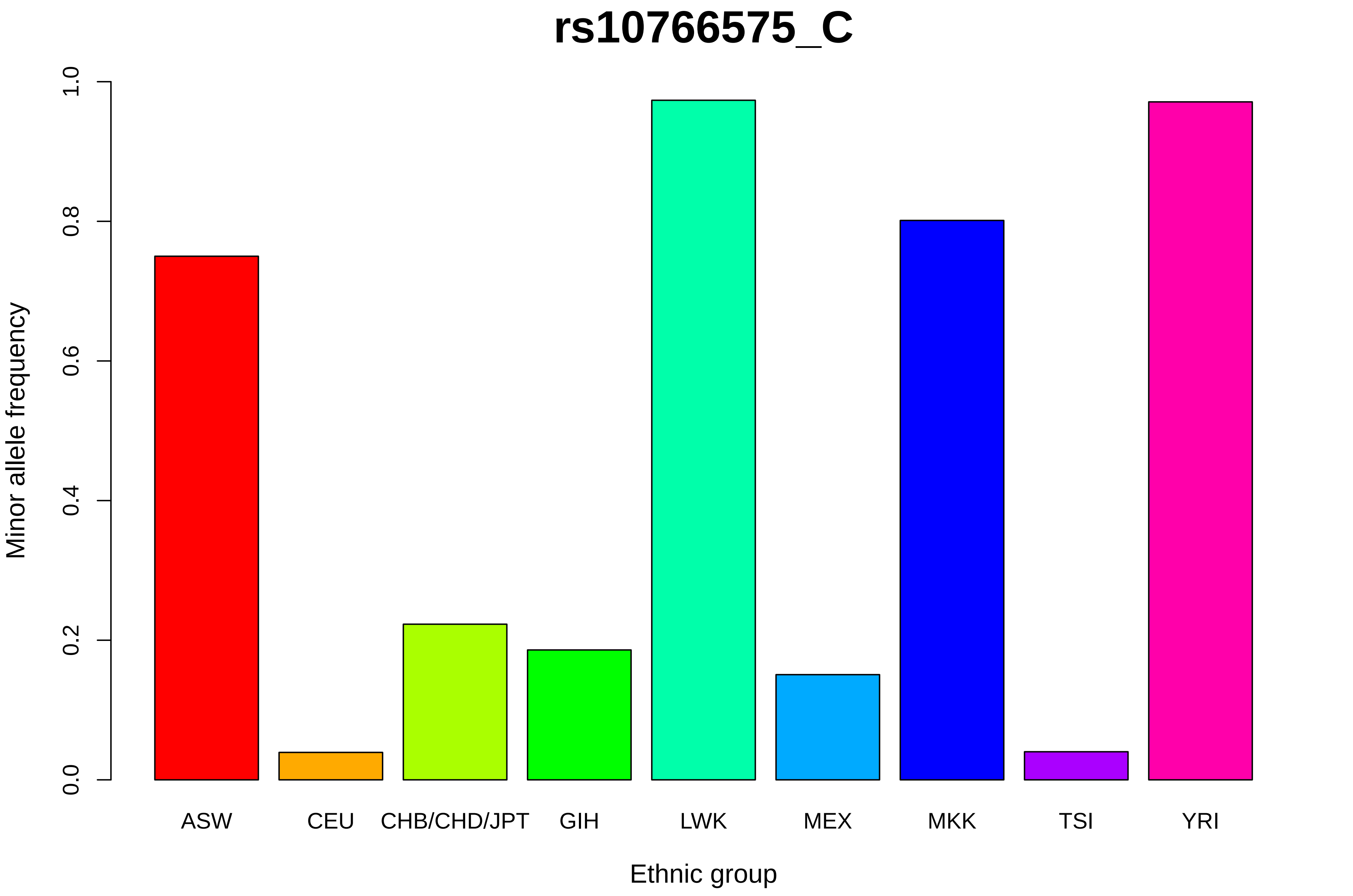
rs10766575_C
Minor allele frequency
0.0
0.2
0.4
0.6
0.8
1.0
ASW
CEU
CHB/CHD/JPT
GIH
LWK
MEX
MKK
TSI
YRI
Ethnic group

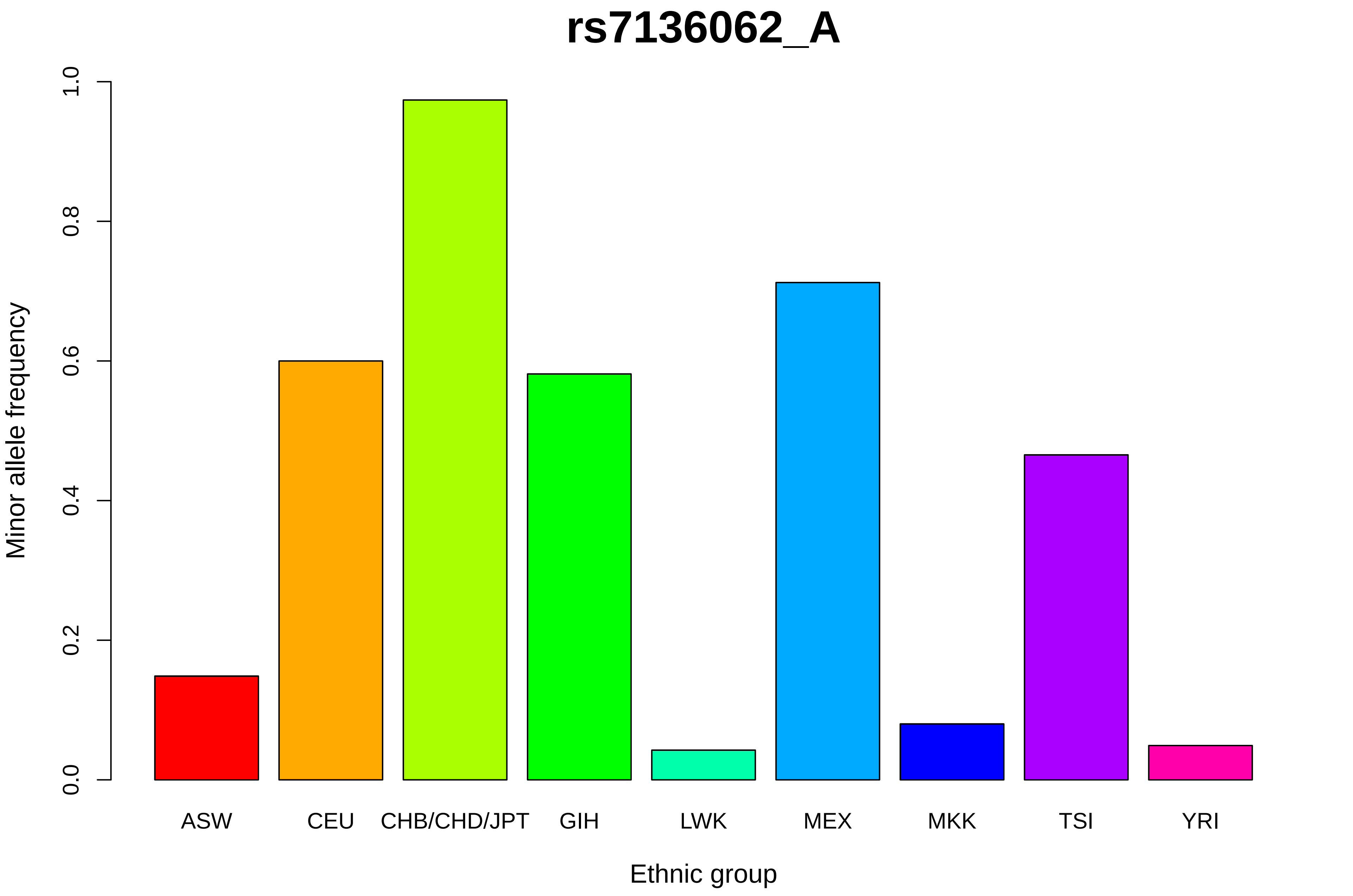

rs7136062_A
Minor allele frequency
0.0
0.2
0.4
0.6
0.8
1.0
ASW
CEU
CHB/CHD/JPT
GIH
LWK
MEX
MKK
TSI
YRI
Ethnic group

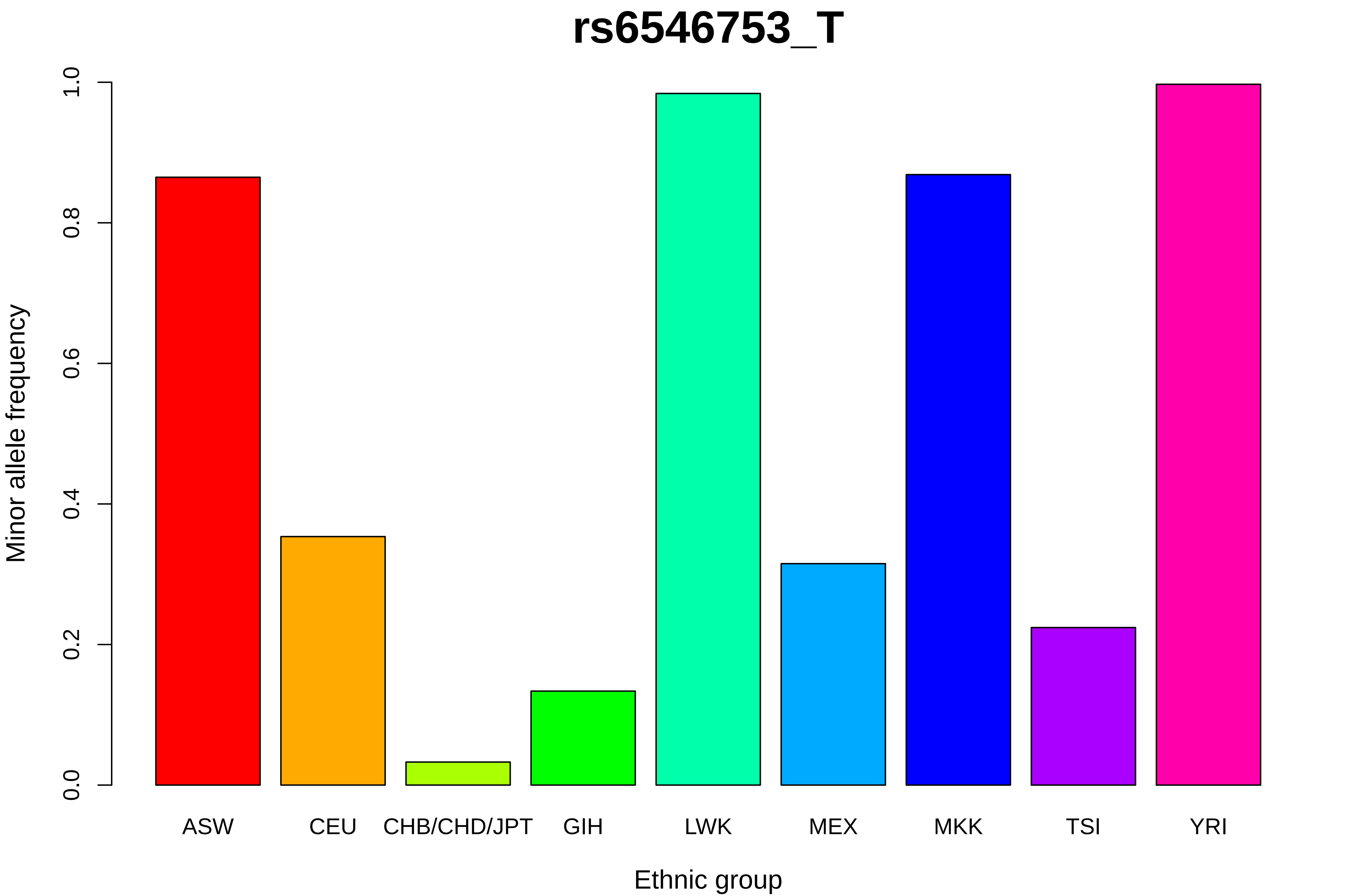

rs6546753_T
Minor allele frequency
0.0
0.2
0.4
0.6
0.8
1.0
ASW
CEU
CHB/CHD/JPT
GIH
LWK
MEX
MKK
TSI
YRI
Ethnic group

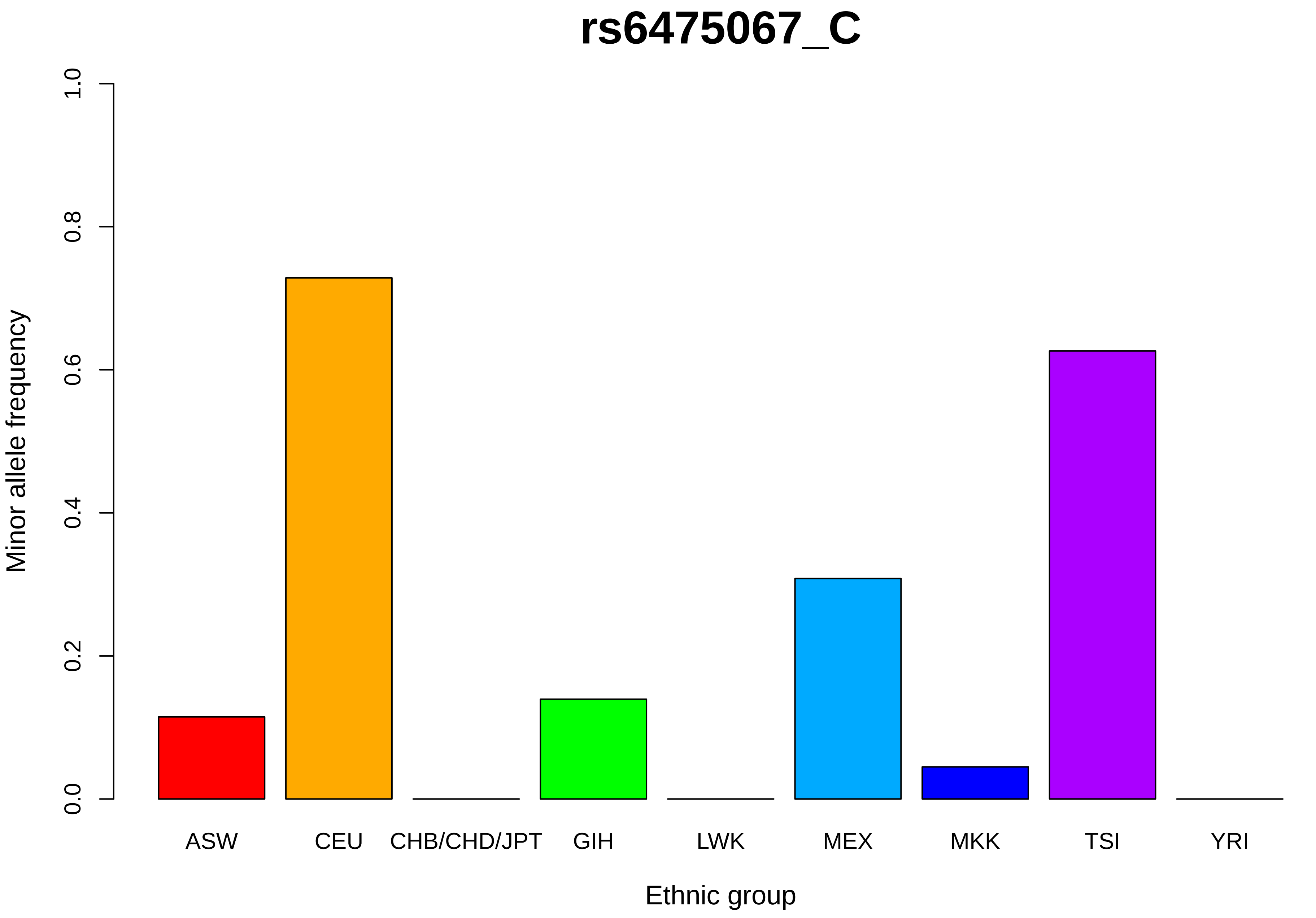
rs6475067_C
Minor allele frequency
0.0
0.2
0.4
0.6
0.8
1.0
ASW
CEU
CHB/CHD/JPT
GIH
LWK
MEX
MKK
TSI
YRI
Ethnic group

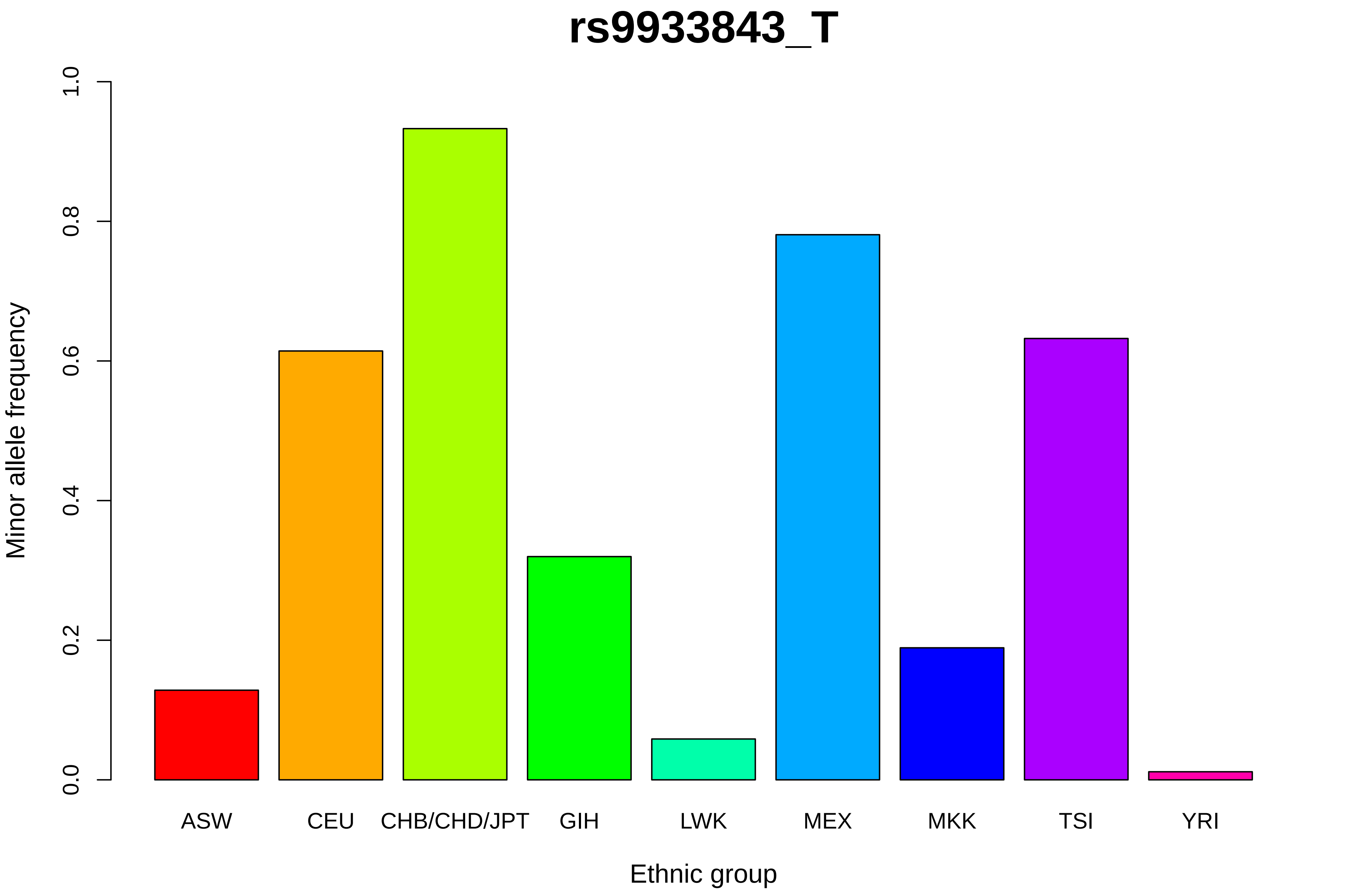
rs9933843_T
Minor allele frequency
0.0
0.2
0.4
0.6
0.8
1.0
ASW
CEU
CHB/CHD/JPT
GIH
LWK
MEX
MKK
TSI
YRI
Ethnic group

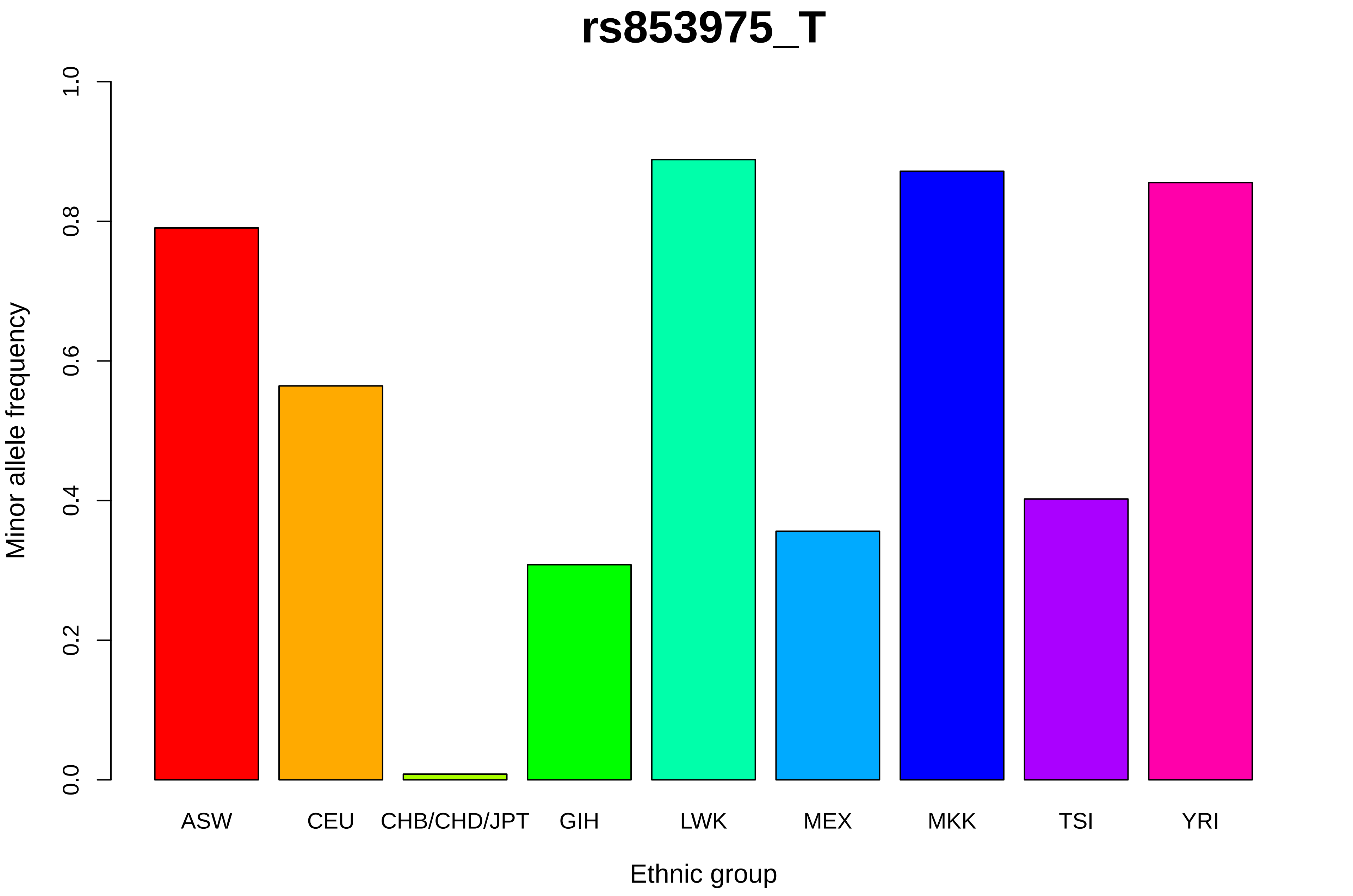
rs853975_T
Minor allele frequency
0.0
0.2
0.4
0.6
0.8
1.0
ASW
CEU
CHB/CHD/JPT
GIH
LWK
MEX
MKK
TSI
YRI
Ethnic group

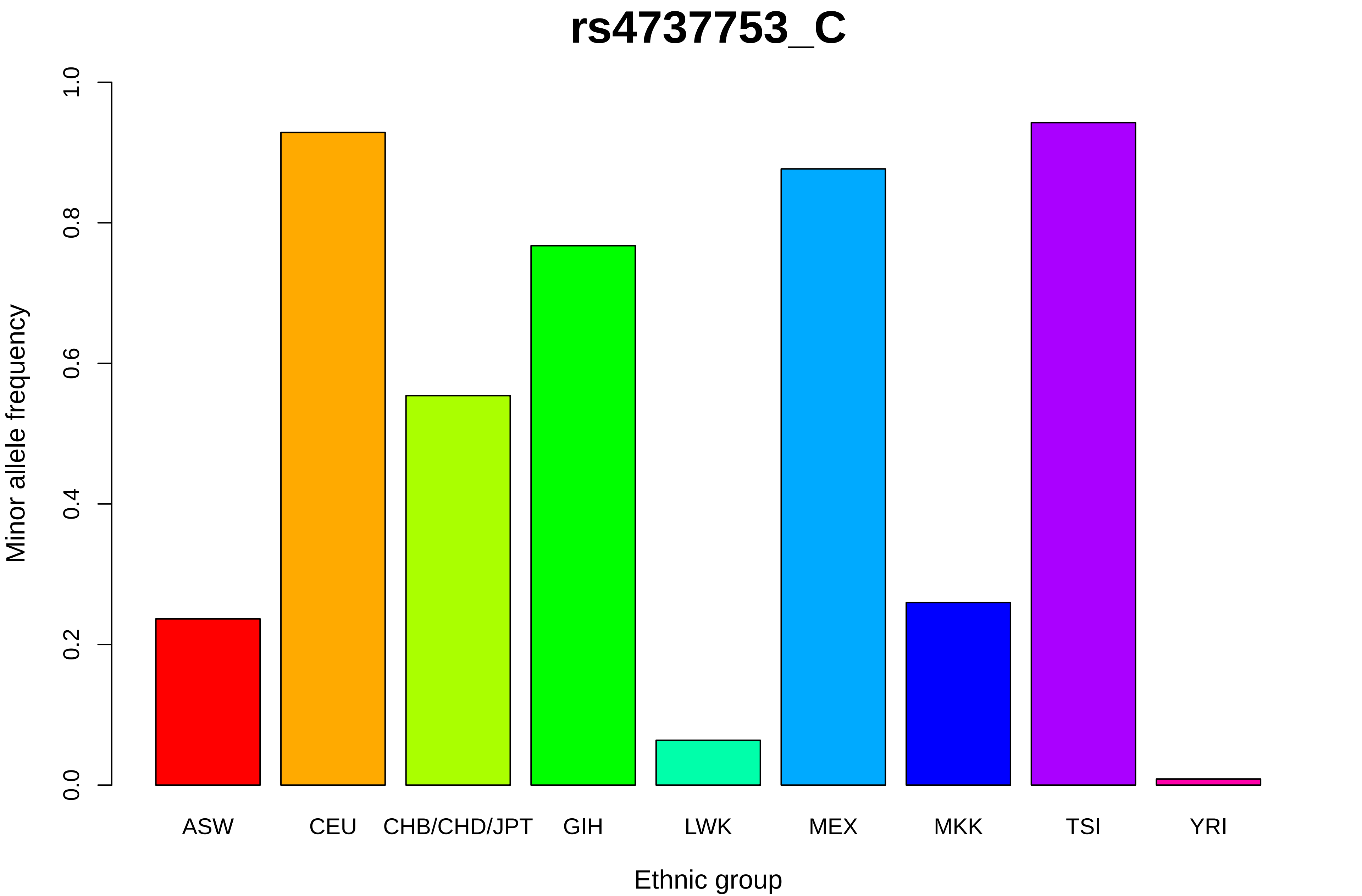

rs4737753_C
Minor allele frequency
0.0
0.2
0.4
0.6
0.8
1.0
ASW
CEU
CHB/CHD/JPT
GIH
LWK
MEX
MKK
TSI
YRI
Ethnic group

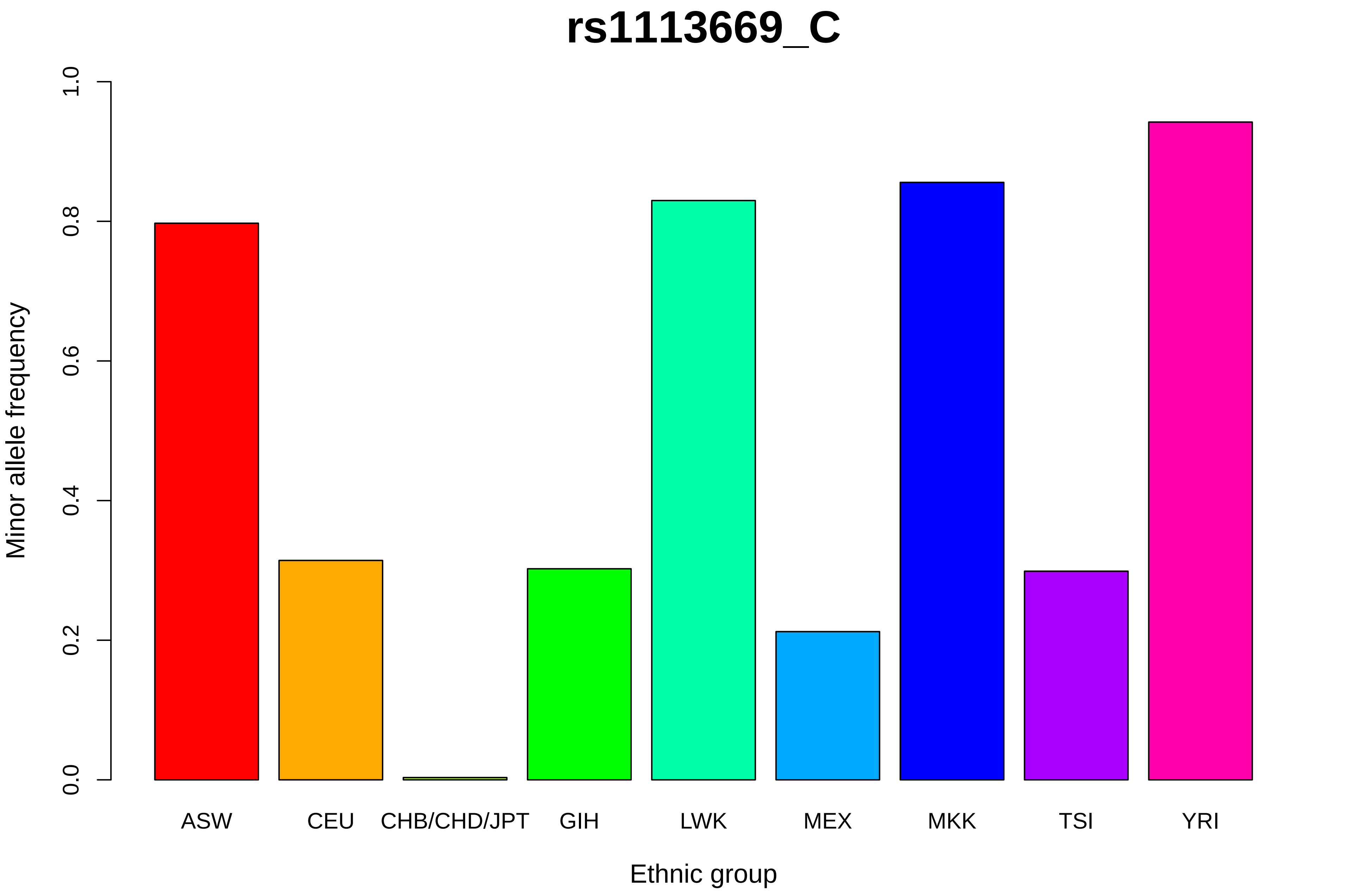
rs1113669_C
Minor allele frequency
0.0
0.2
0.4
0.6
0.8
1.0
ASW
CEU
CHB/CHD/JPT
GIH
LWK
MEX
MKK
TSI
YRI
Ethnic group

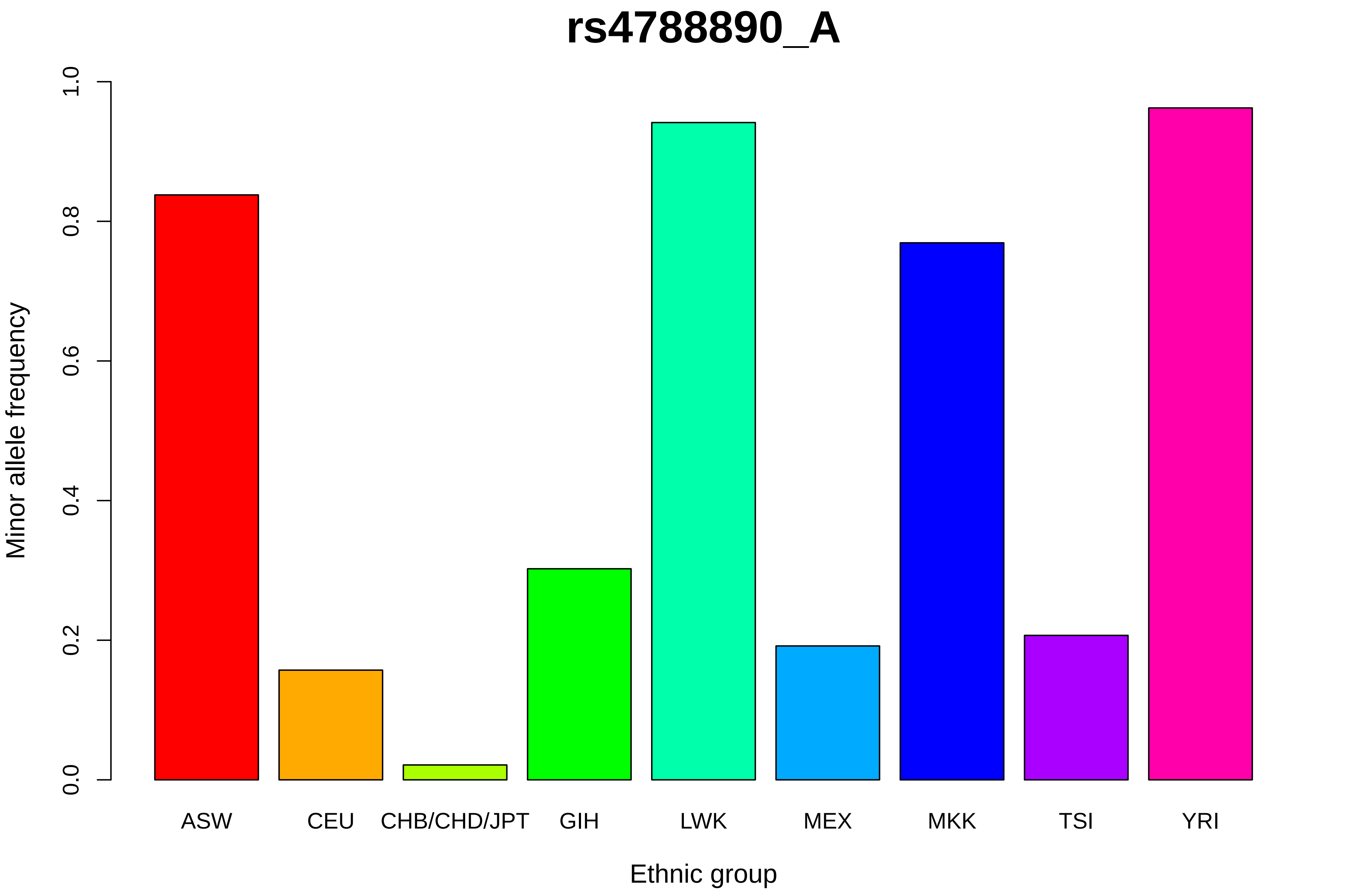
rs4788890_A
Minor allele frequency
0.0
0.2
0.4
0.6
0.8
1.0
ASW
CEU
CHB/CHD/JPT
GIH
LWK
MEX
MKK
TSI
YRI
Ethnic group

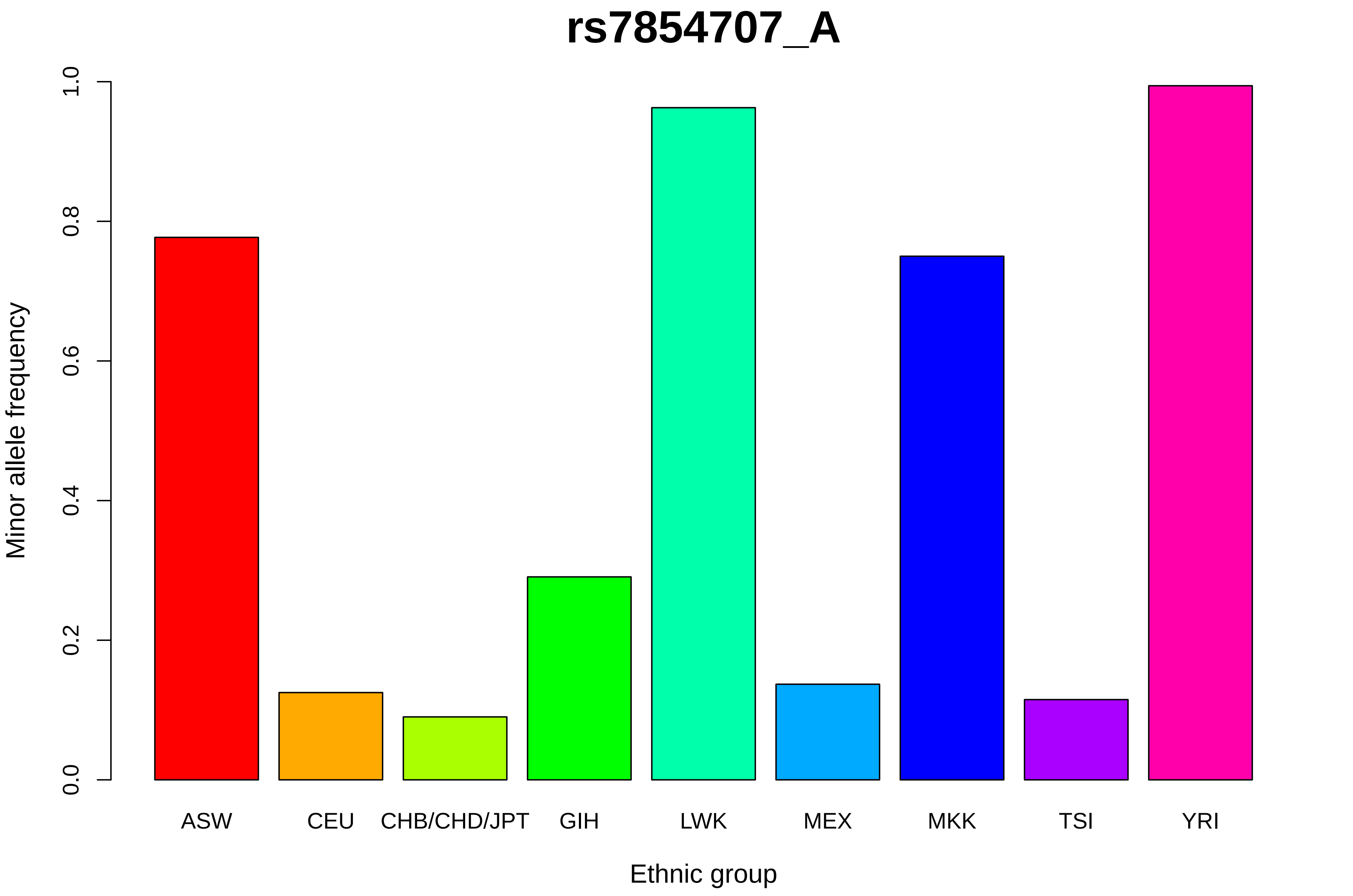
rs7854707_A
Minor allele frequency
0.0
0.2
0.4
0.6
0.8
1.0
ASW
CEU
CHB/CHD/JPT
GIH
LWK
MEX
MKK
TSI
YRI
Ethnic group

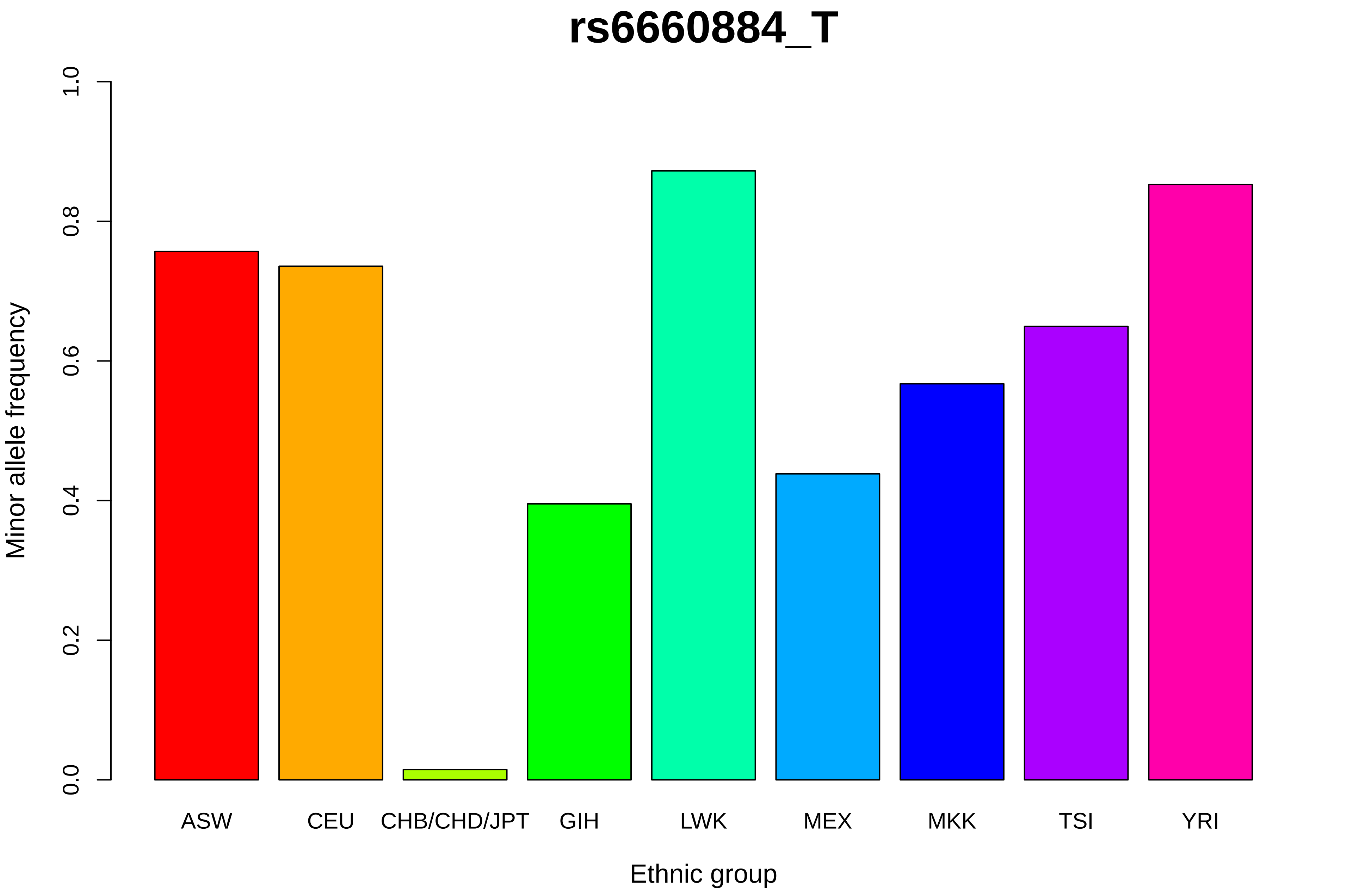
rs6660884_T
Minor allele frequency
0.0
0.2
0.4
0.6
0.8
1.0
ASW
CEU
CHB/CHD/JPT
GIH
LWK
MEX
MKK
TSI
YRI
Ethnic group

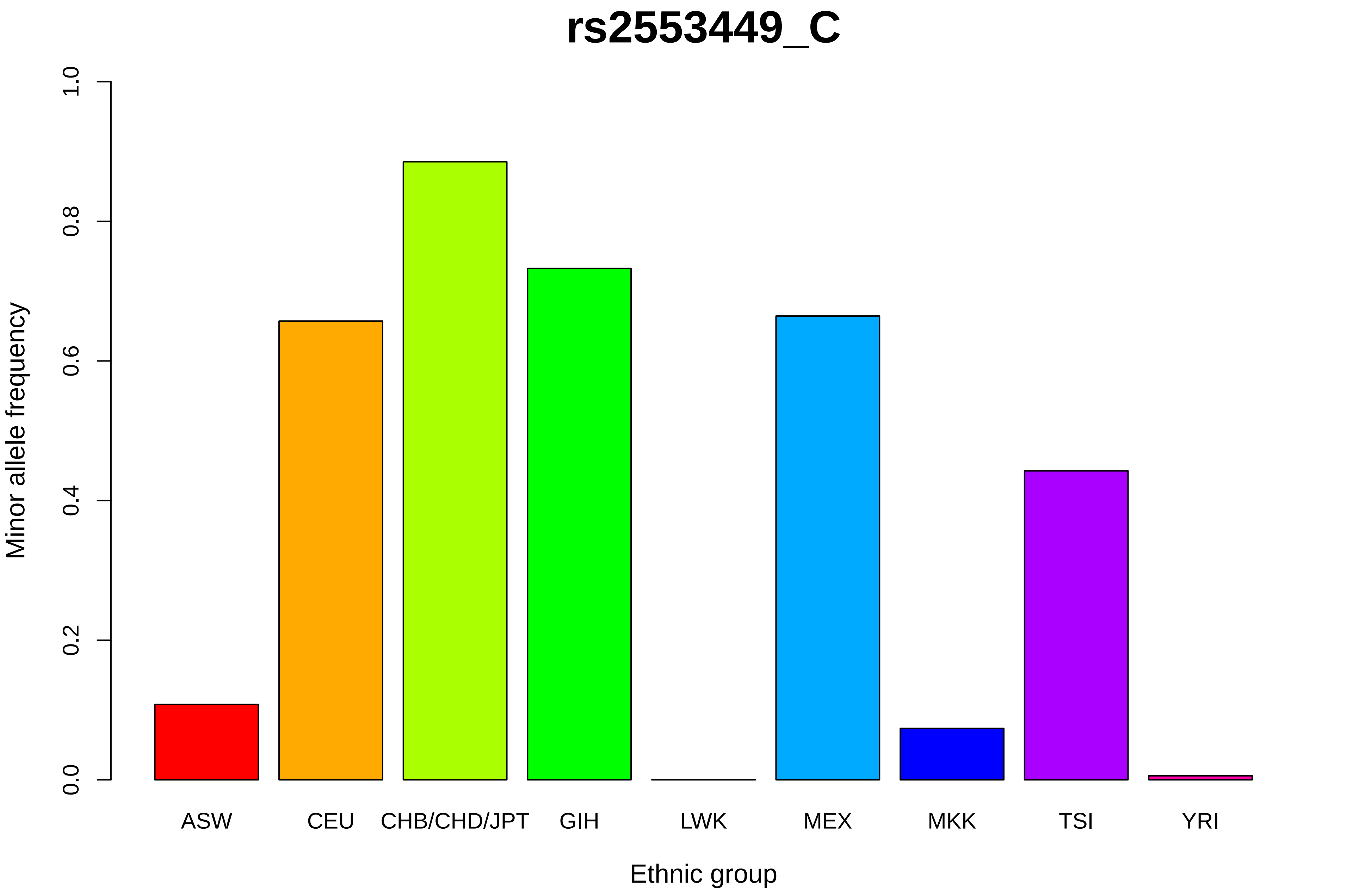
rs2553449_C
Minor allele frequency
0.0
0.2
0.4
0.6
0.8
1.0
ASW
CEU
CHB/CHD/JPT
GIH
LWK
MEX
MKK
TSI
YRI
Ethnic group

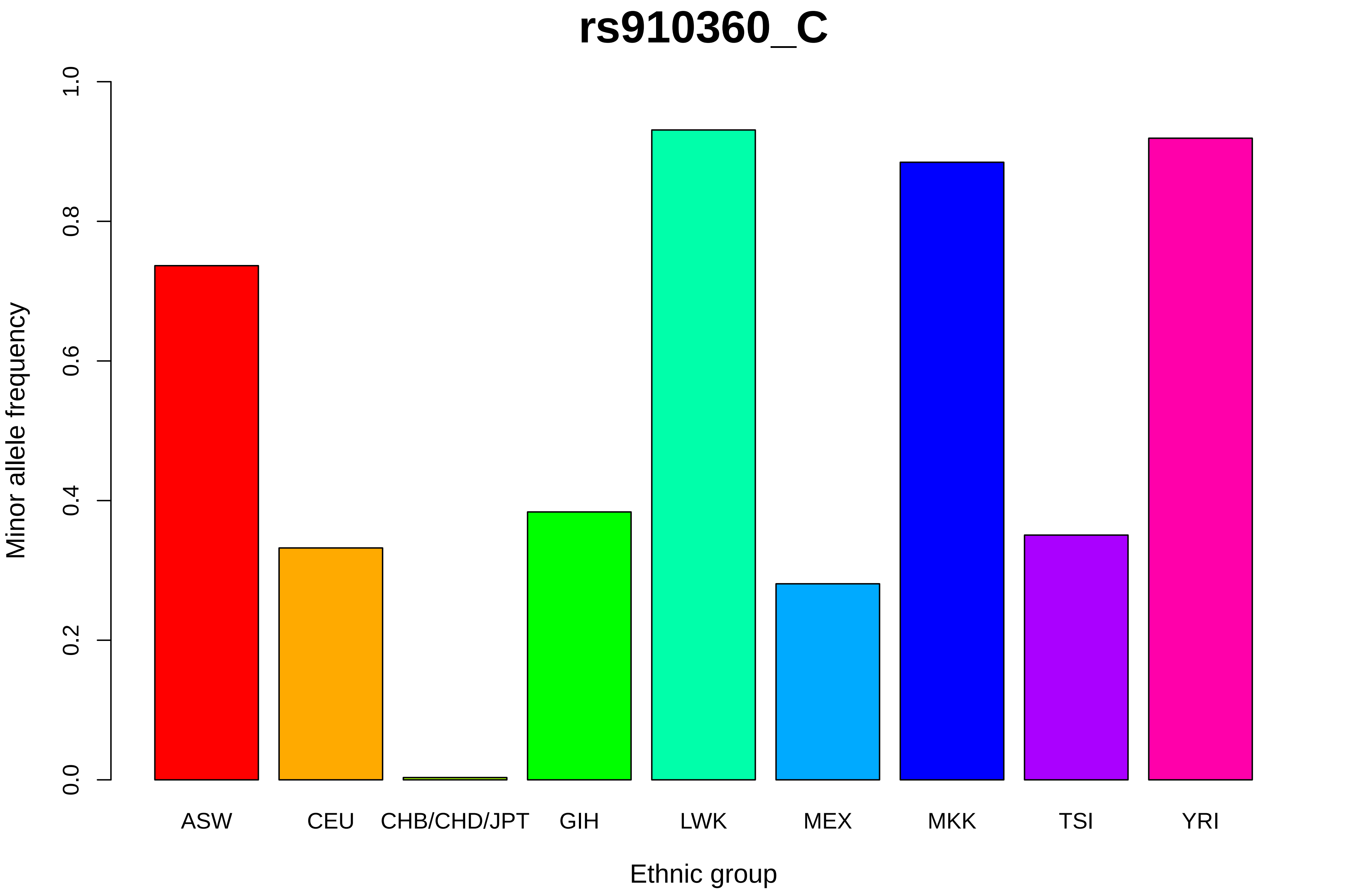

rs910360_C
Minor allele frequency
0.0
0.2
0.4
0.6
0.8
1.0
ASW
CEU
CHB/CHD/JPT
GIH
LWK
MEX
MKK
TSI
YRI
Ethnic group

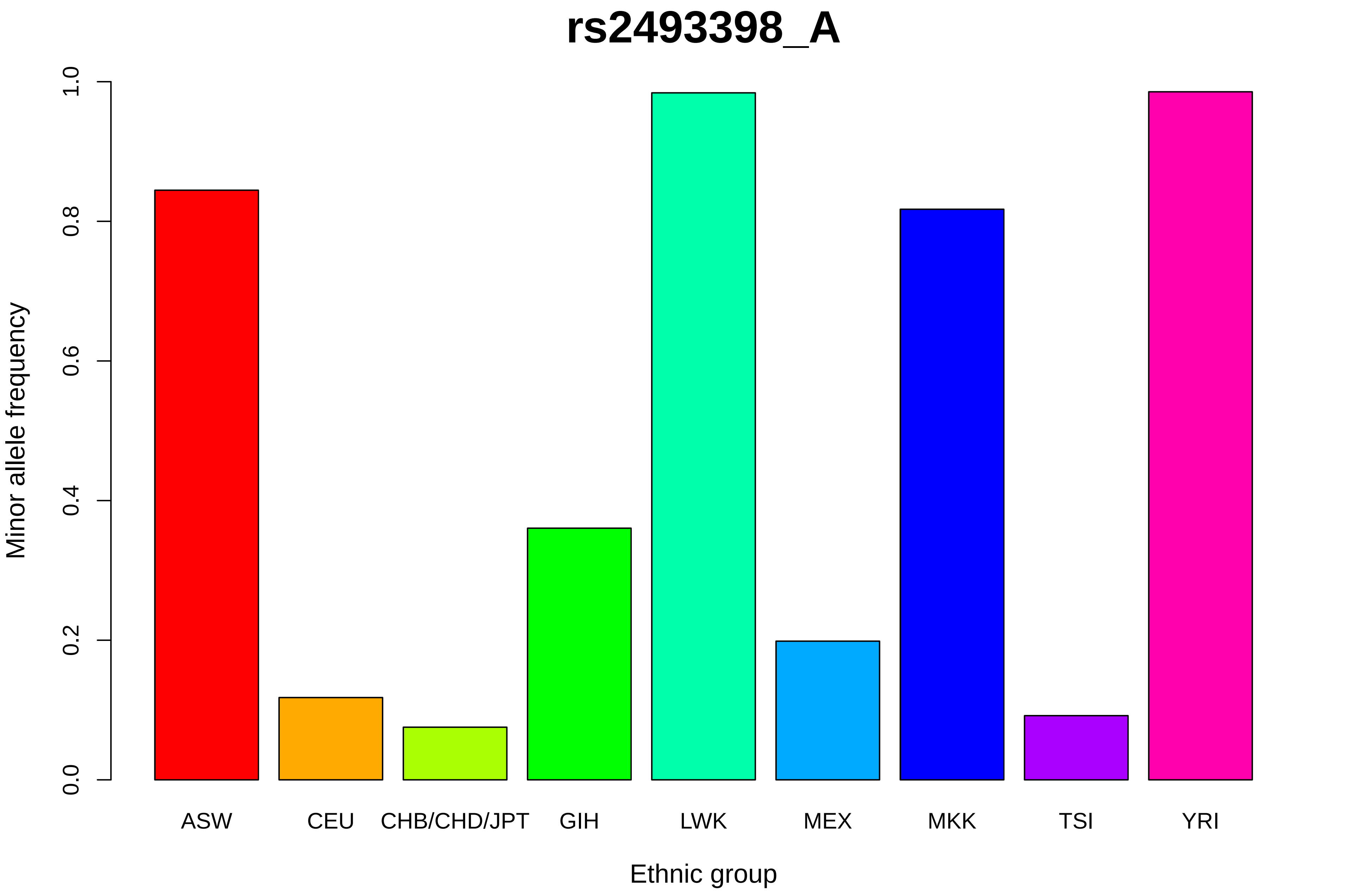

rs2493398_A
Minor allele frequency
0.0
0.2
0.4
0.6
0.8
1.0
ASW
CEU
CHB/CHD/JPT
GIH
LWK
MEX
MKK
TSI
YRI
Ethnic group

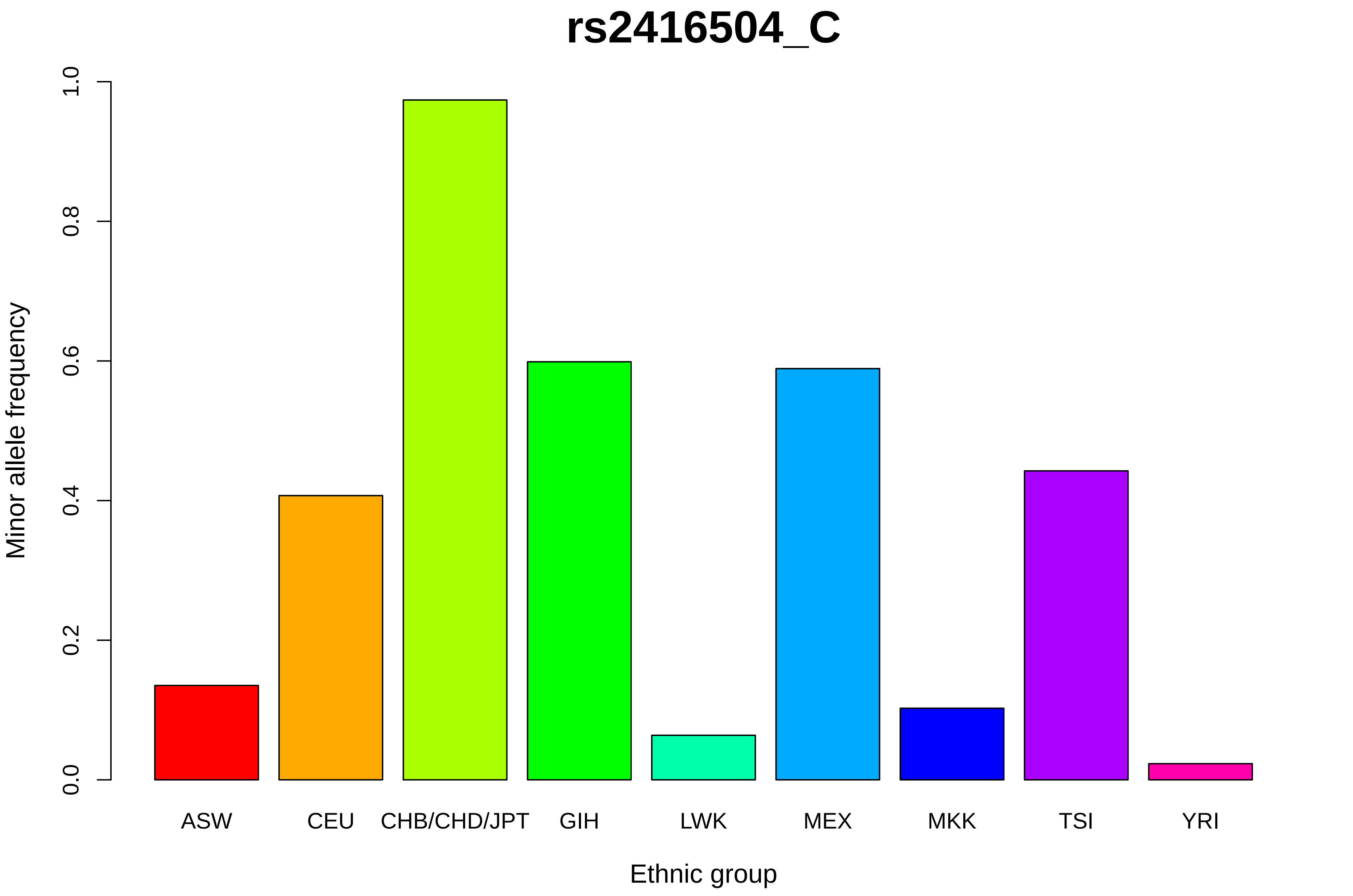
rs2416504_C
Minor allele frequency
0.0
0.2
0.4
0.6
0.8
1.0
ASW
CEU
CHB/CHD/JPT
GIH
LWK
MEX
MKK
TSI
YRI
Ethnic group

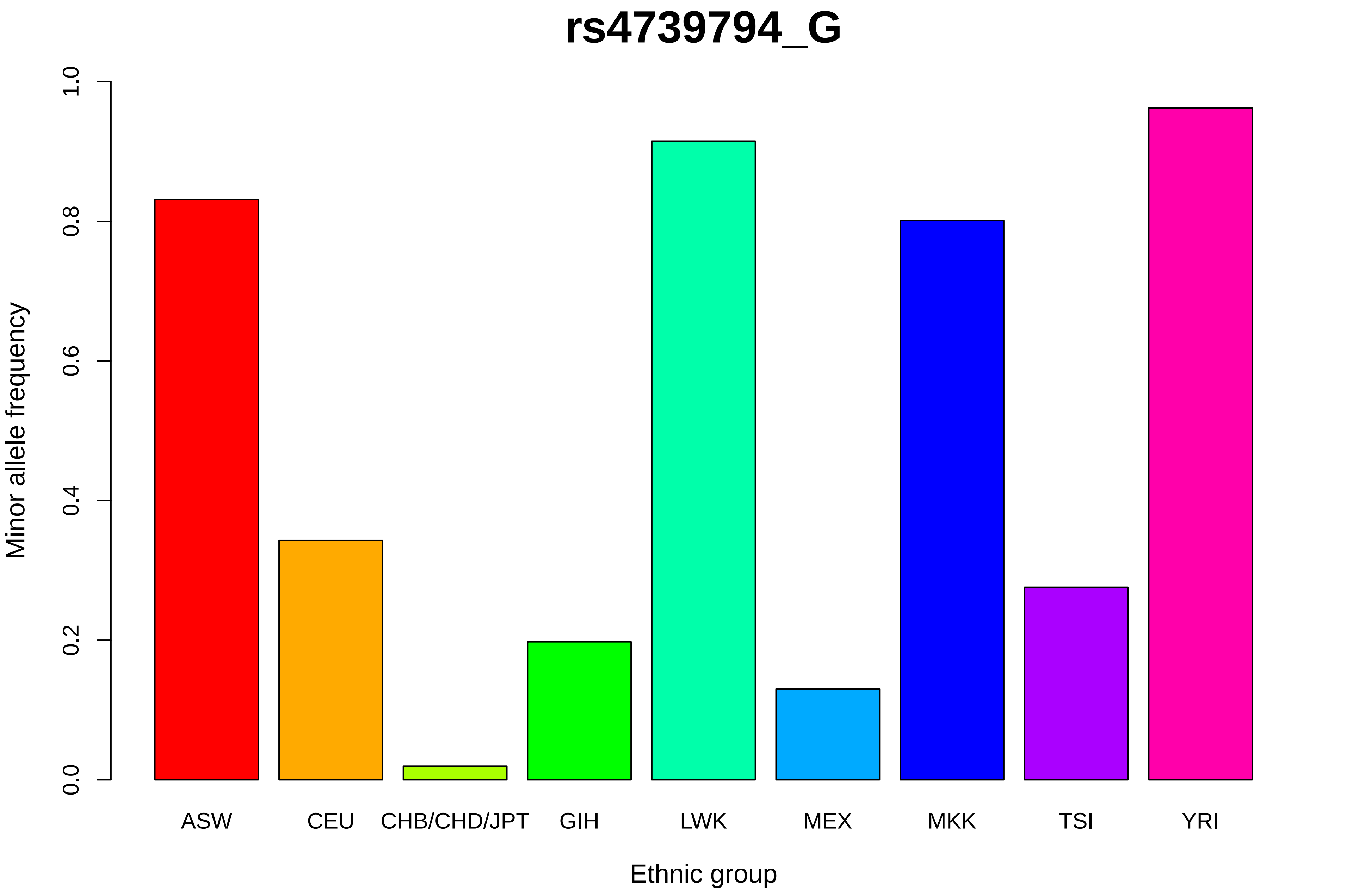
rs4739794_G
Minor allele frequency
0.0
0.2
0.4
0.6
0.8
1.0
ASW
CEU
CHB/CHD/JPT
GIH
LWK
MEX
MKK
TSI
YRI
Ethnic group

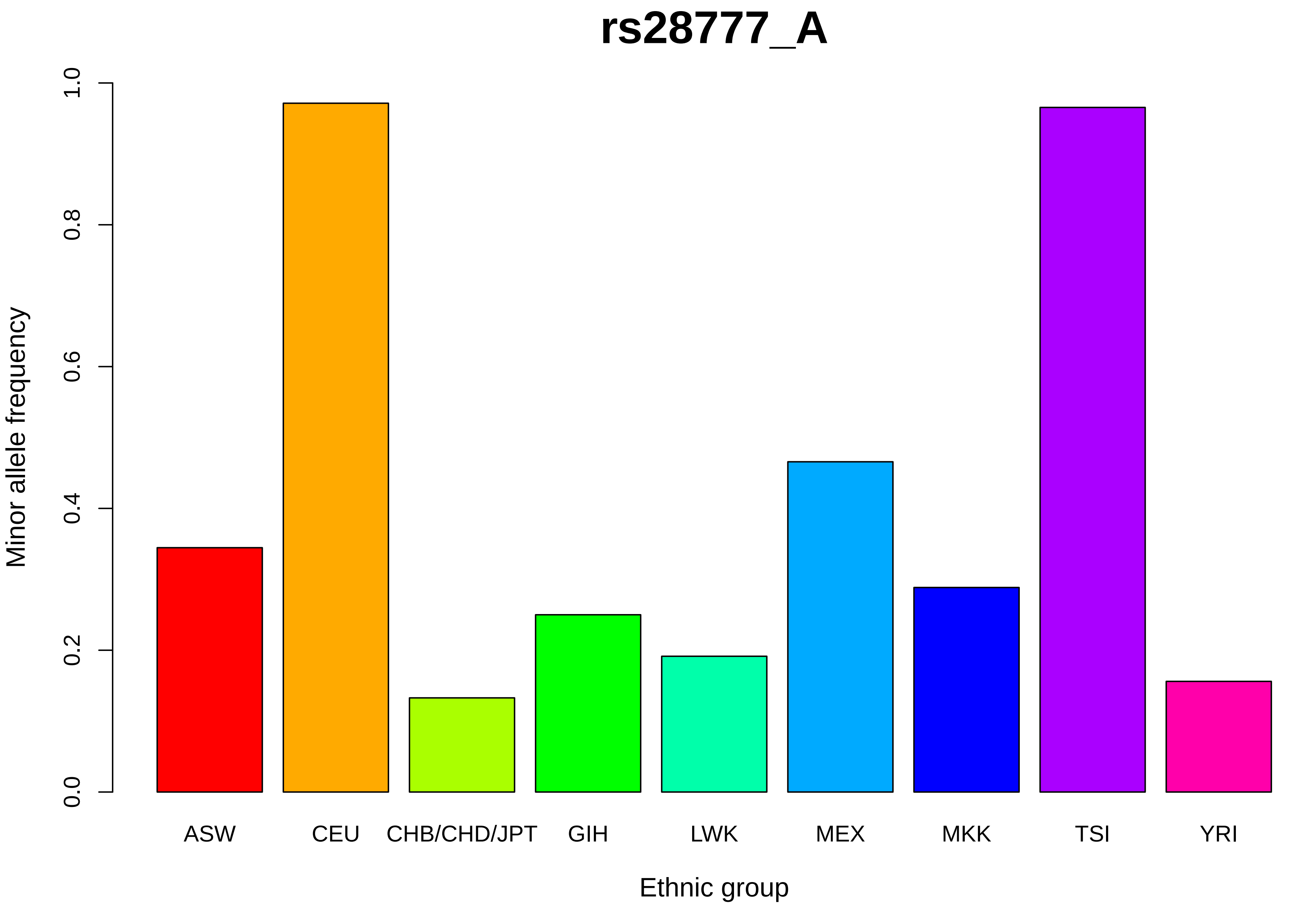
rs28777_A
Minor allele frequency
0.0
0.2
0.4
0.6
0.8
1.0
ASW
CEU
CHB/CHD/JPT
GIH
LWK
MEX
MKK
TSI
YRI
Ethnic group

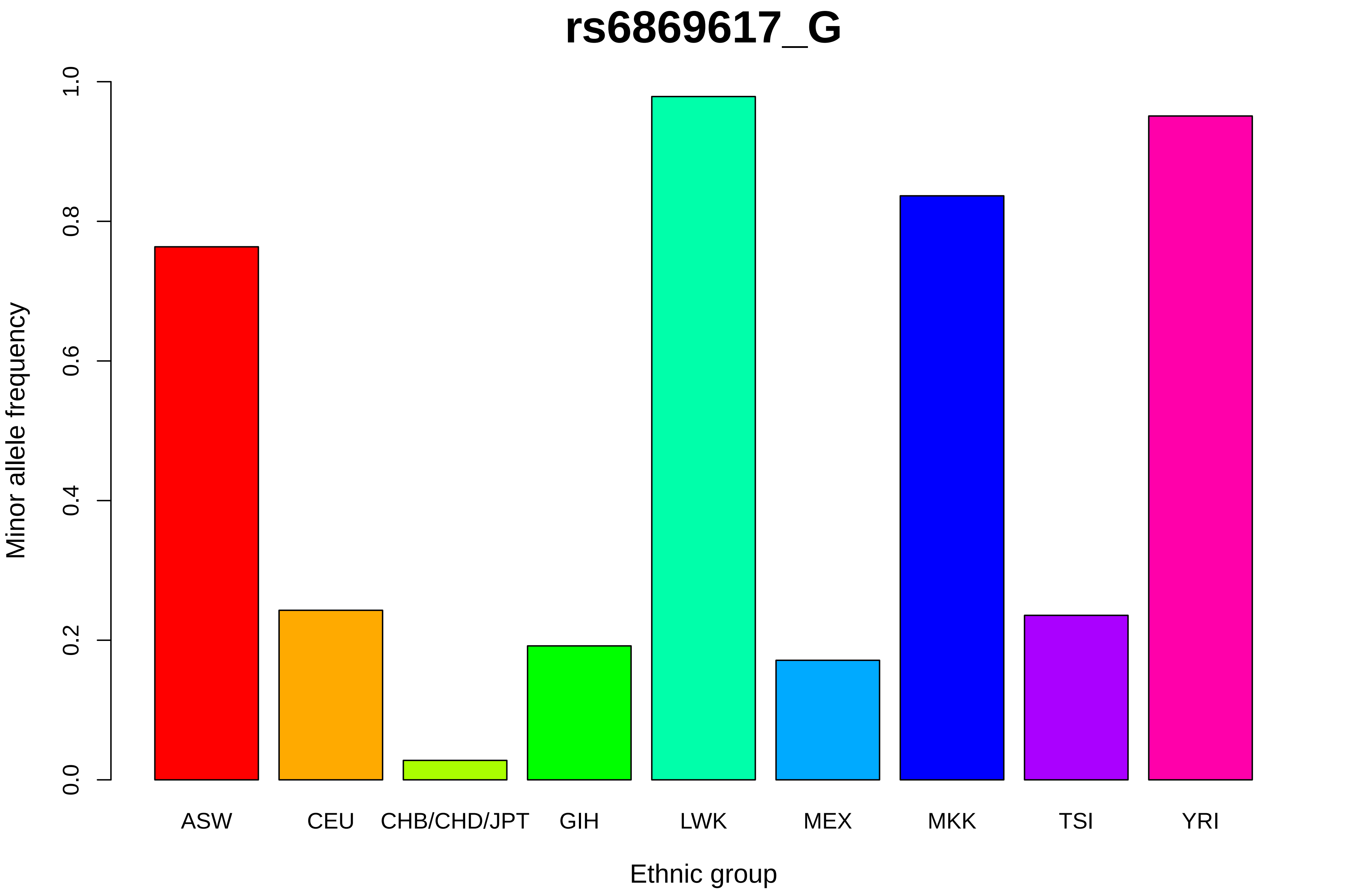
rs6869617_G
Minor allele frequency
0.0
0.2
0.4
0.6
0.8
1.0
ASW
CEU
CHB/CHD/JPT
GIH
LWK
MEX
MKK
TSI
YRI
Ethnic group

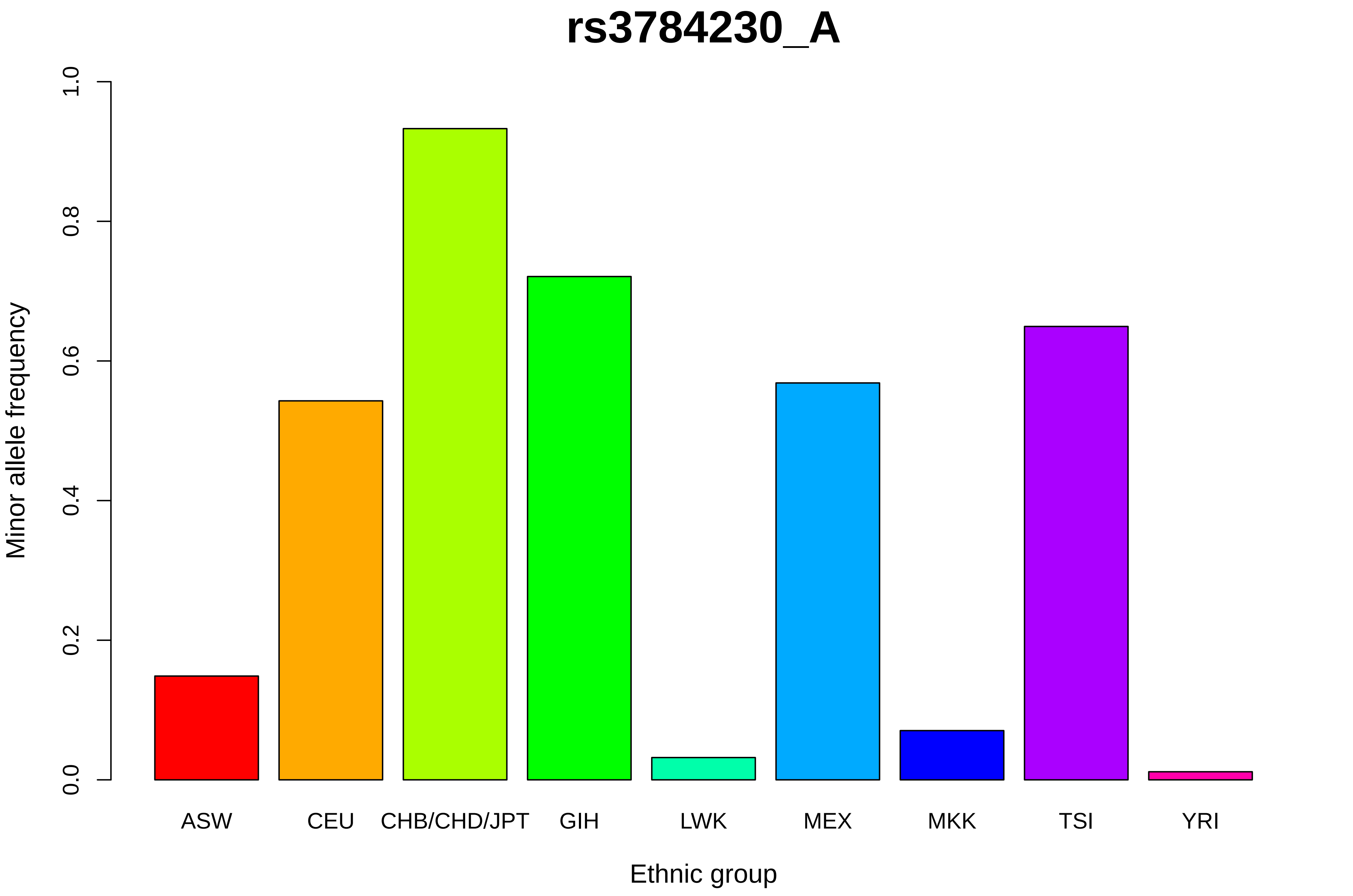
rs3784230_A
Minor allele frequency
0.0
0.2
0.4
0.6
0.8
1.0
ASW
CEU
CHB/CHD/JPT
GIH
LWK
MEX
MKK
TSI
YRI
Ethnic group

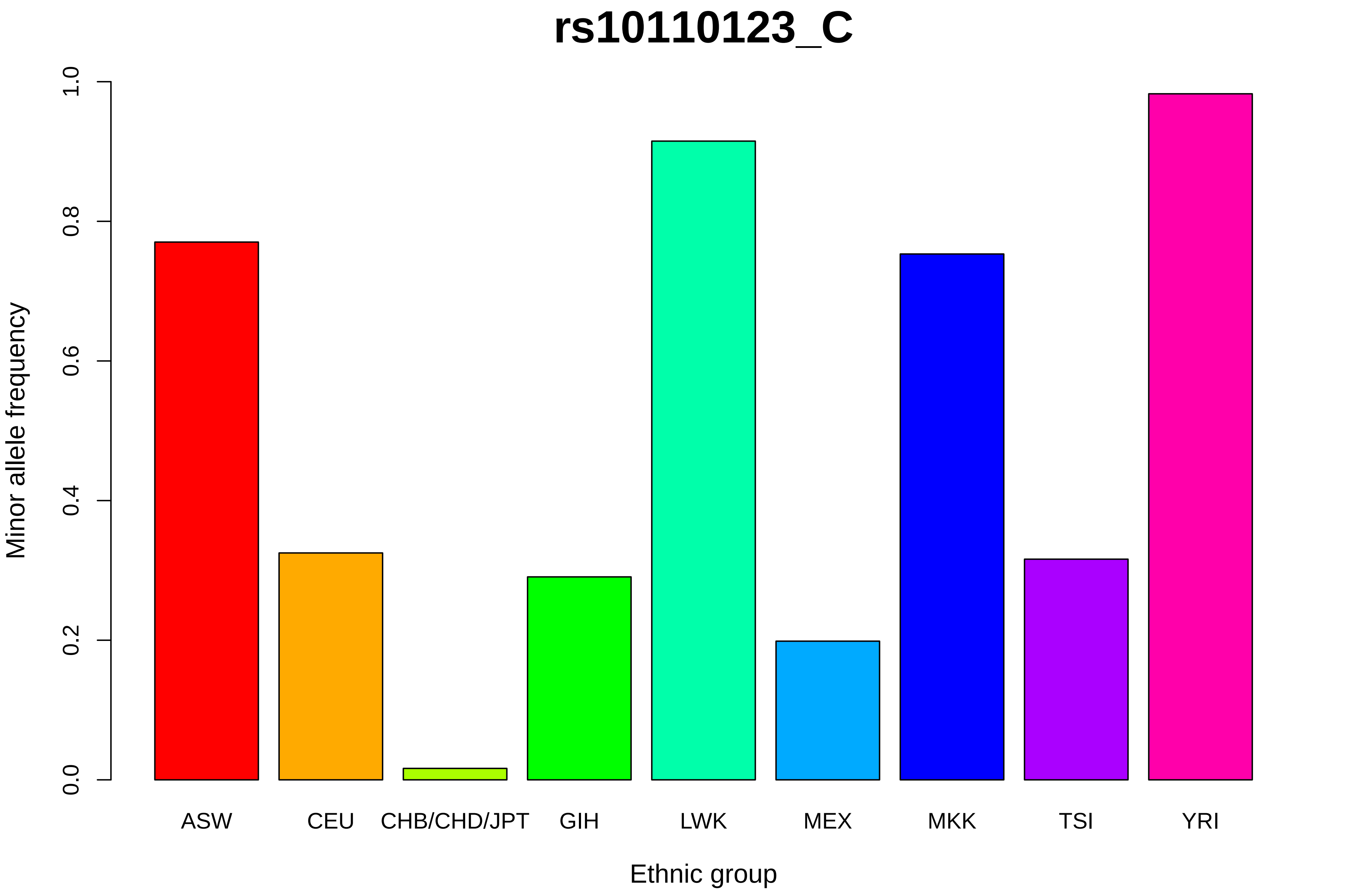
rs10110123_C
Minor allele frequency
0.0
0.2
0.4
0.6
0.8
1.0
ASW
CEU
CHB/CHD/JPT
GIH
LWK
MEX
MKK
TSI
YRI
Ethnic group

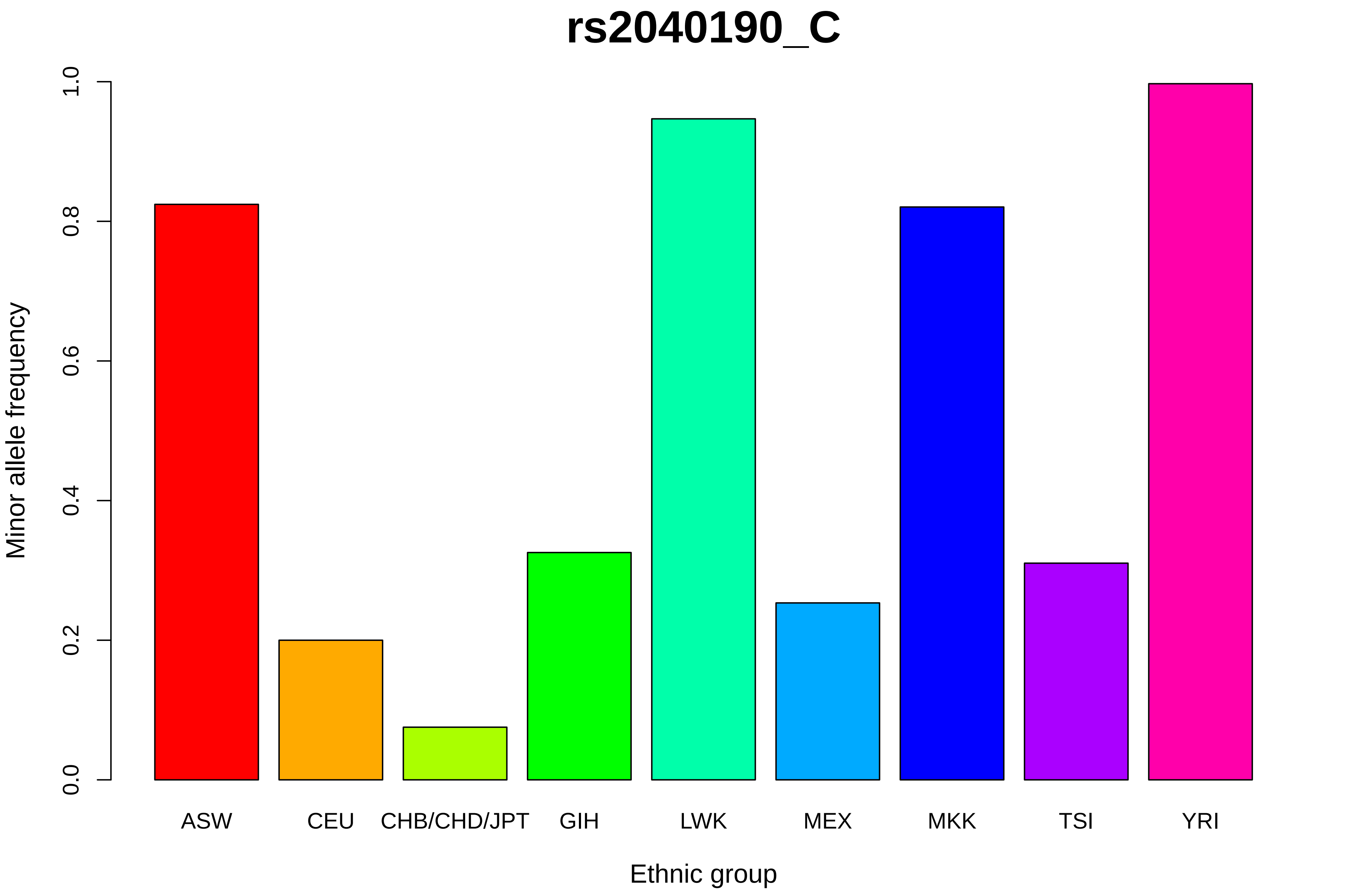

rs2040190_C
Minor allele frequency
0.0
0.2
0.4
0.6
0.8
1.0
ASW
CEU
CHB/CHD/JPT
GIH
LWK
MEX
MKK
TSI
YRI
Ethnic group

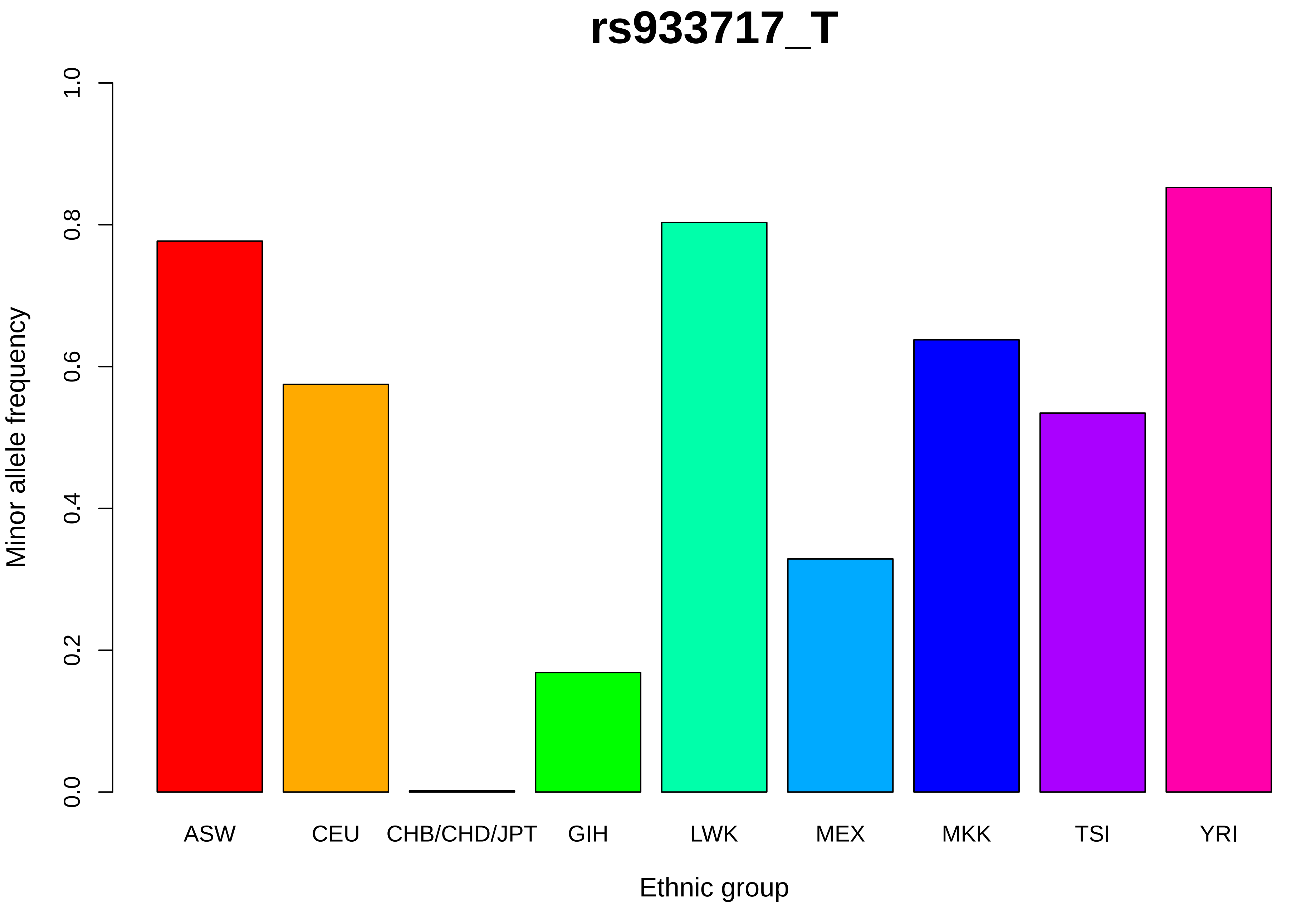
rs933717_T
Minor allele frequency
0.0
0.2
0.4
0.6
0.8
1.0
ASW
CEU
CHB/CHD/JPT
GIH
LWK
MEX
MKK
TSI
YRI
Ethnic group

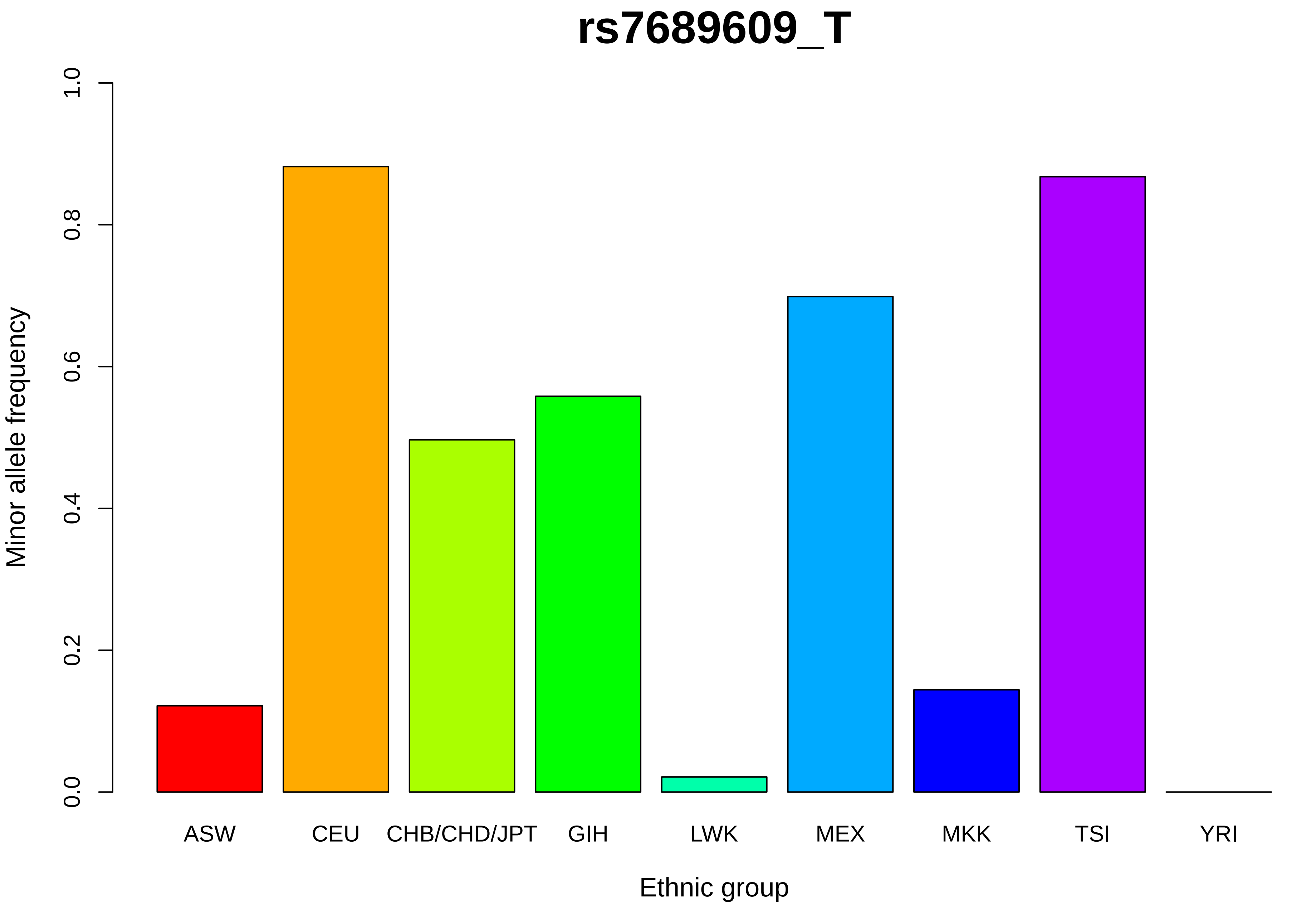
rs7689609_T
Minor allele frequency
0.0
0.2
0.4
0.6
0.8
1.0
ASW
CEU
CHB/CHD/JPT
GIH
LWK
MEX
MKK
TSI
YRI
Ethnic group

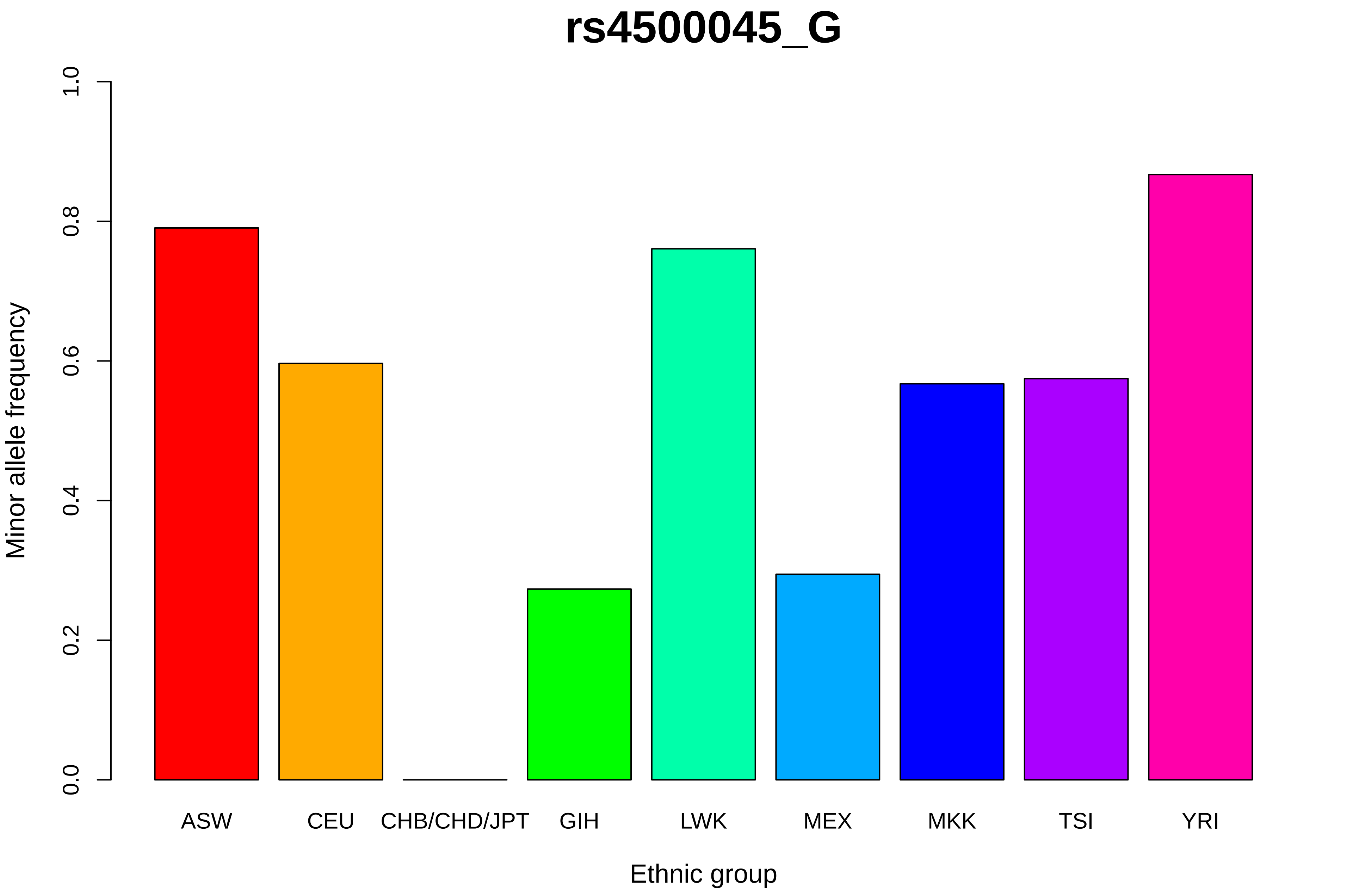
rs4500045_G
Minor allele frequency
0.0
0.2
0.4
0.6
0.8
1.0
ASW
CEU
CHB/CHD/JPT
GIH
LWK
MEX
MKK
TSI
YRI
Ethnic group

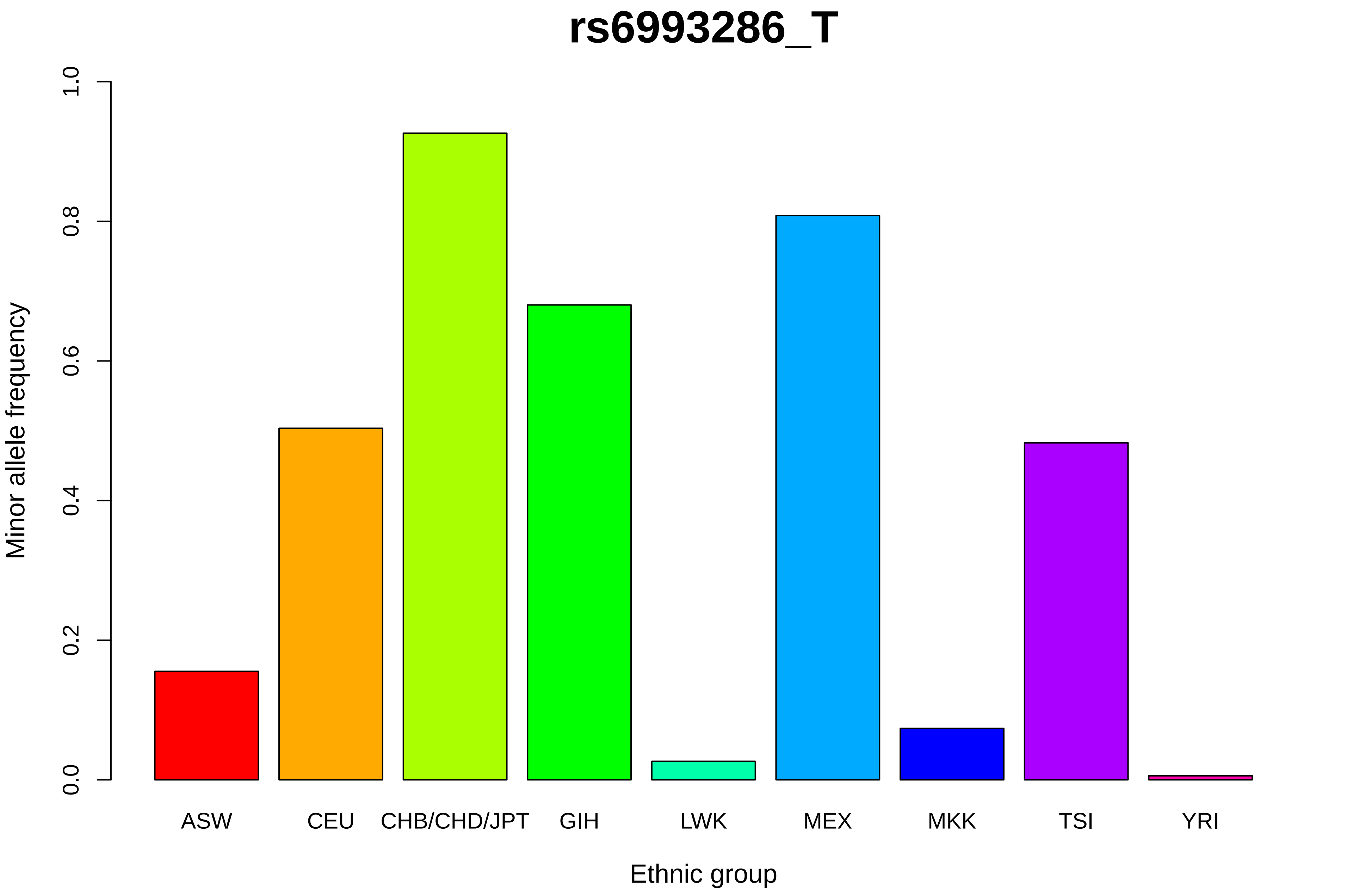
rs6993286_T
Minor allele frequency
0.0
0.2
0.4
0.6
0.8
1.0
ASW
CEU
CHB/CHD/JPT
GIH
LWK
MEX
MKK
TSI
YRI
Ethnic group

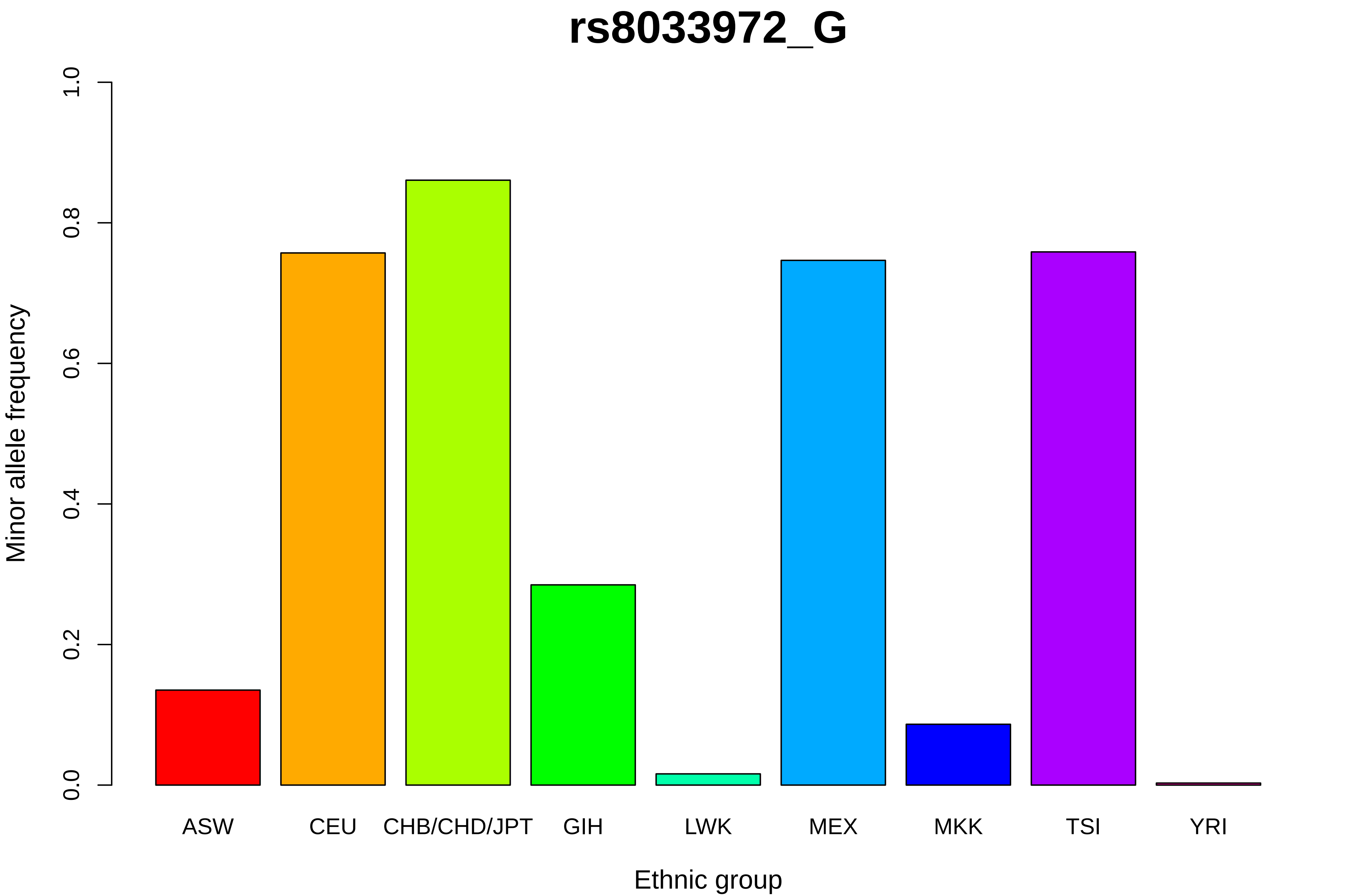
rs8033972_G
Minor allele frequency
0.0
0.2
0.4
0.6
0.8
1.0
ASW
CEU
CHB/CHD/JPT
GIH
LWK
MEX
MKK
TSI
YRI
Ethnic group

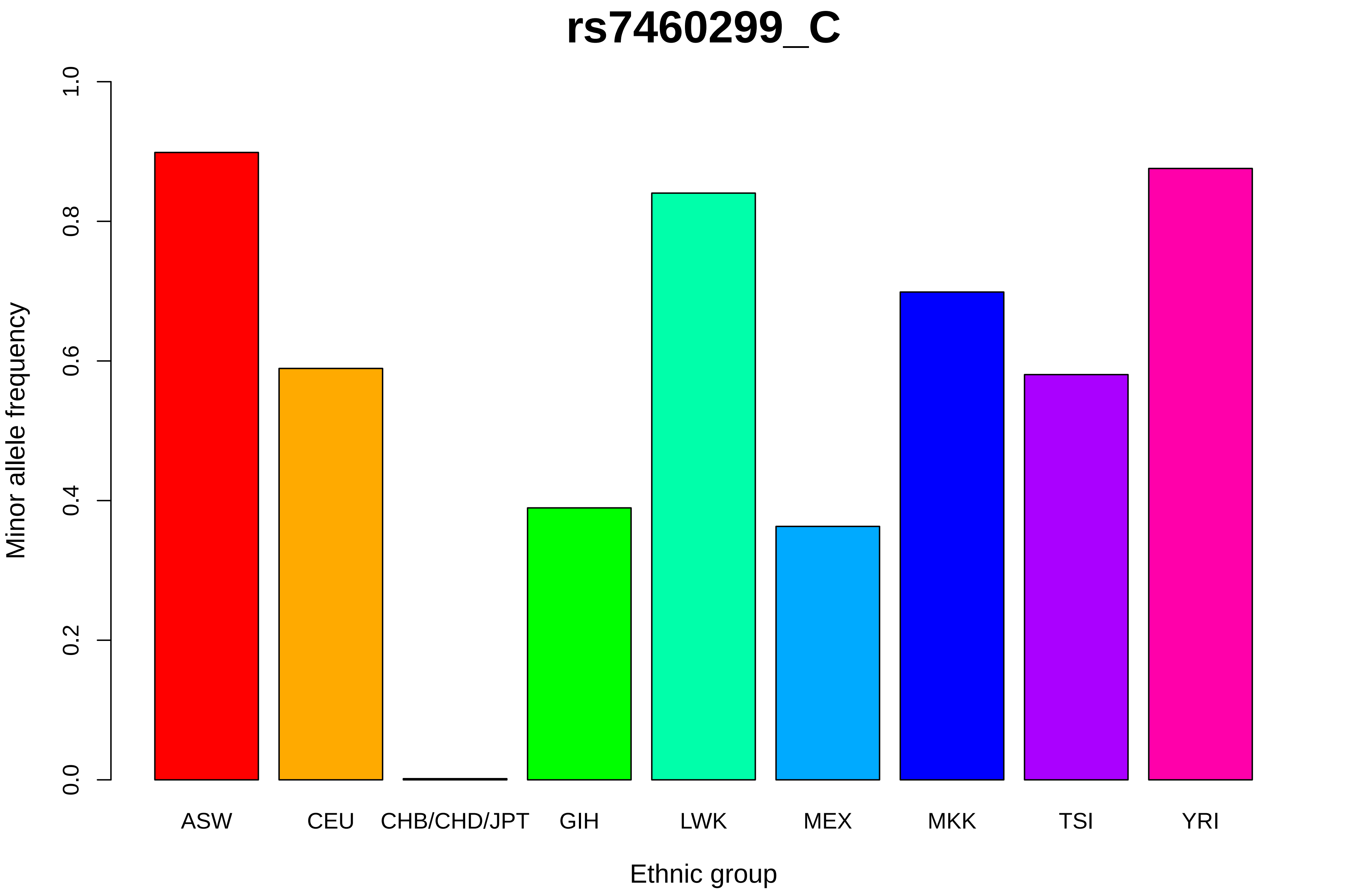

rs7460299_C
Minor allele frequency
0.0
0.2
0.4
0.6
0.8
1.0
ASW
CEU
CHB/CHD/JPT
GIH
LWK
MEX
MKK
TSI
YRI
Ethnic group

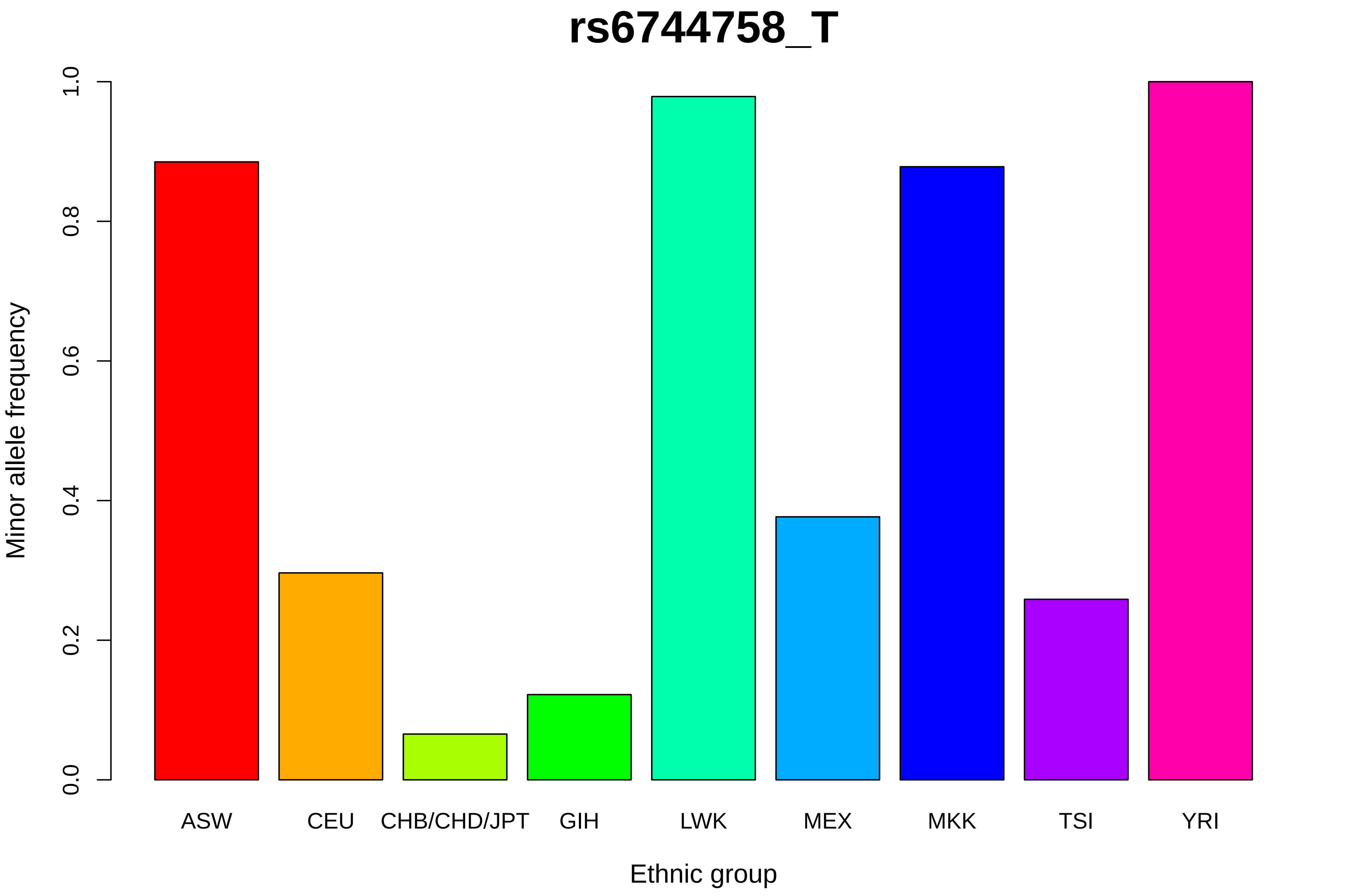

rs6744758_T
Minor allele frequency
0.0
0.2
0.4
0.6
0.8
1.0
ASW
CEU
CHB/CHD/JPT
GIH
LWK
MEX
MKK
TSI
YRI
Ethnic group

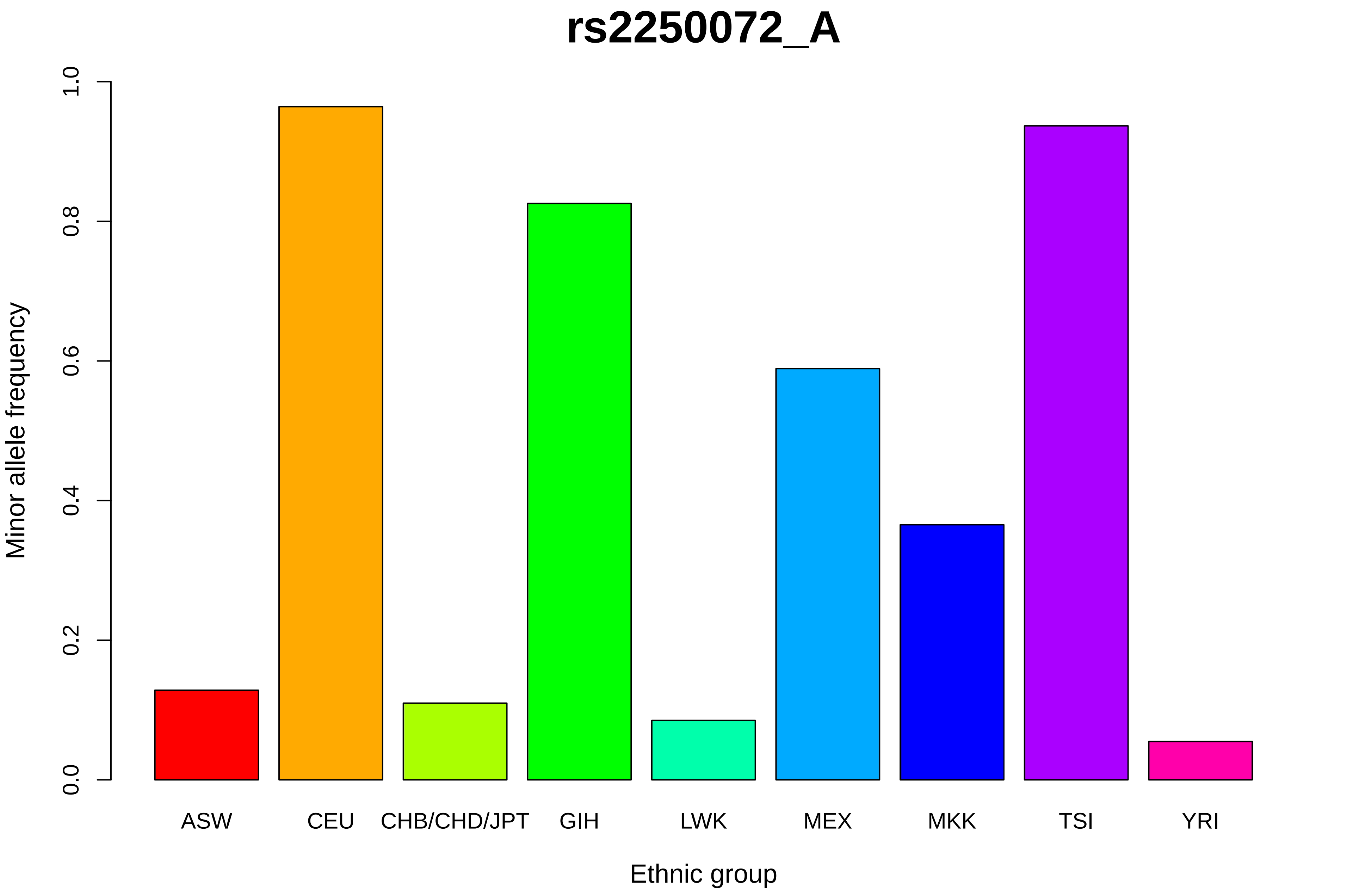

rs2250072_A
Minor allele frequency
0.0
0.2
0.4
0.6
0.8
1.0
ASW
CEU
CHB/CHD/JPT
GIH
LWK
MEX
MKK
TSI
YRI
Ethnic group

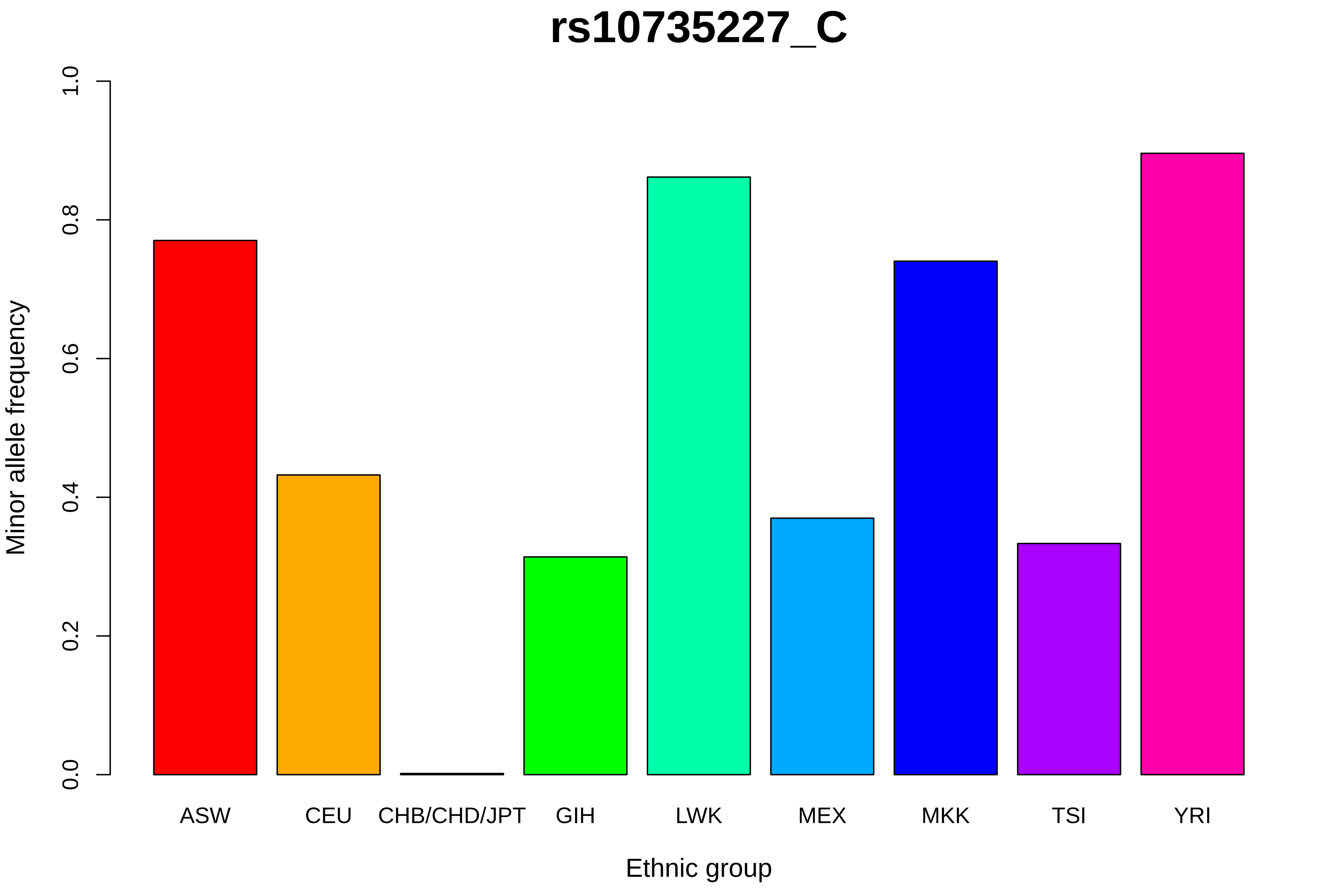

rs10735227_C
Minor allele frequency
0.0
0.2
0.4
0.6
0.8
1.0
ASW
CEU
CHB/CHD/JPT
GIH
LWK
MEX
MKK
TSI
YRI
Ethnic group

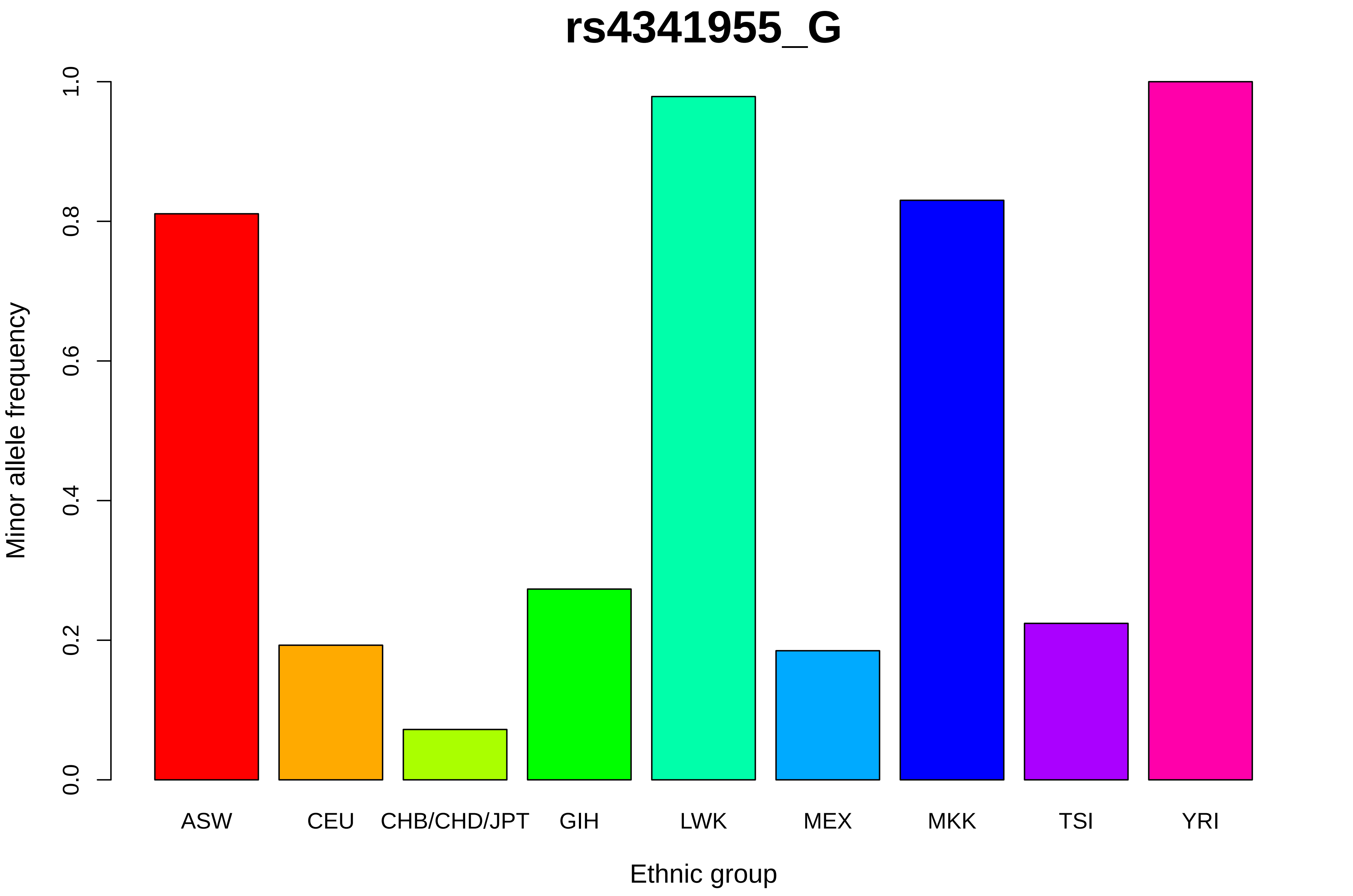
rs4341955_G
Minor allele frequency
0.0
0.2
0.4
0.6
0.8
1.0
ASW
CEU
CHB/CHD/JPT
GIH
LWK
MEX
MKK
TSI
YRI
Ethnic group

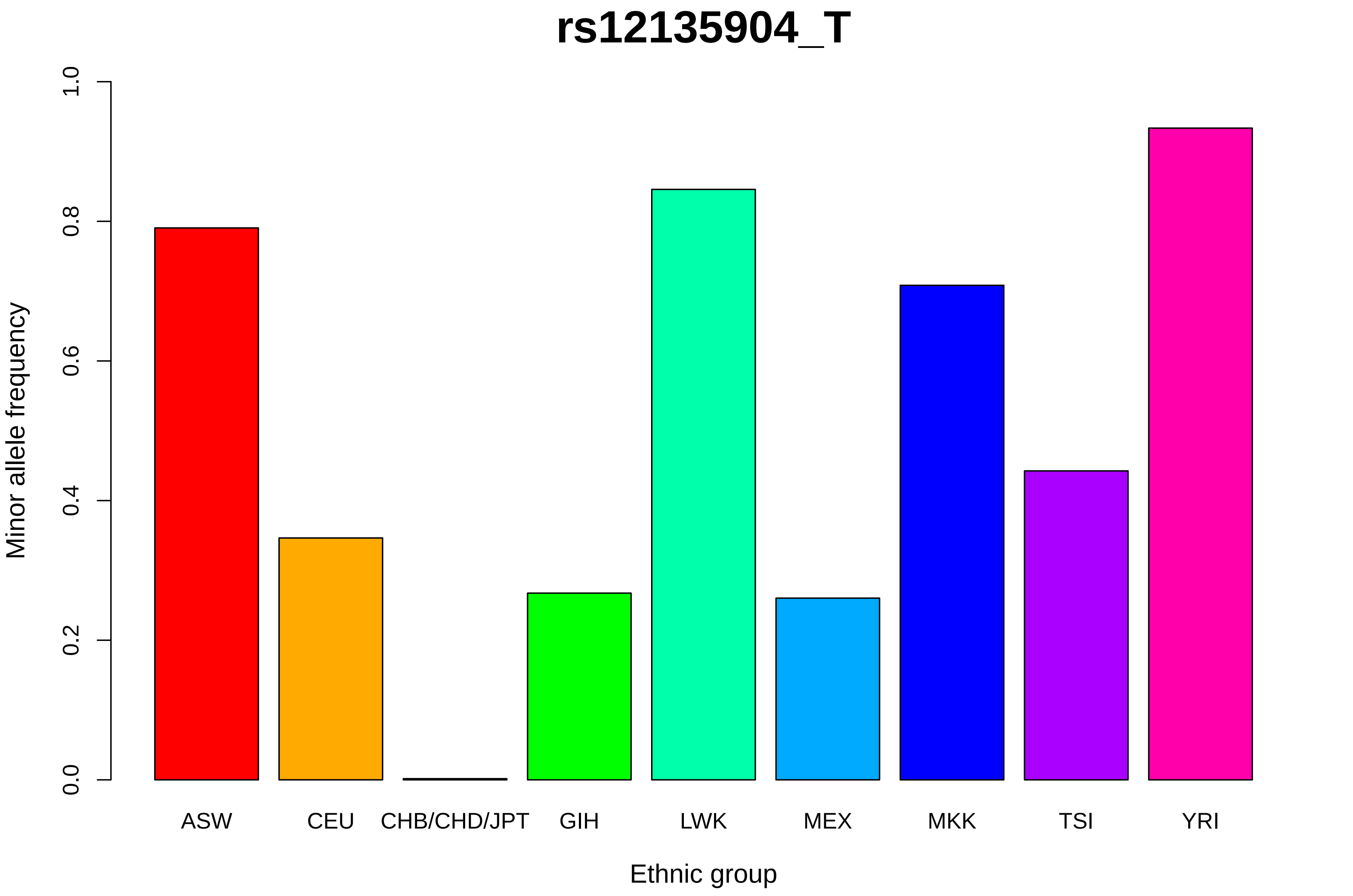
rs12135904_T
Minor allele frequency
0.0
0.2
0.4
0.6
0.8
1.0
ASW
CEU
CHB/CHD/JPT
GIH
LWK
MEX
MKK
TSI
YRI
Ethnic group

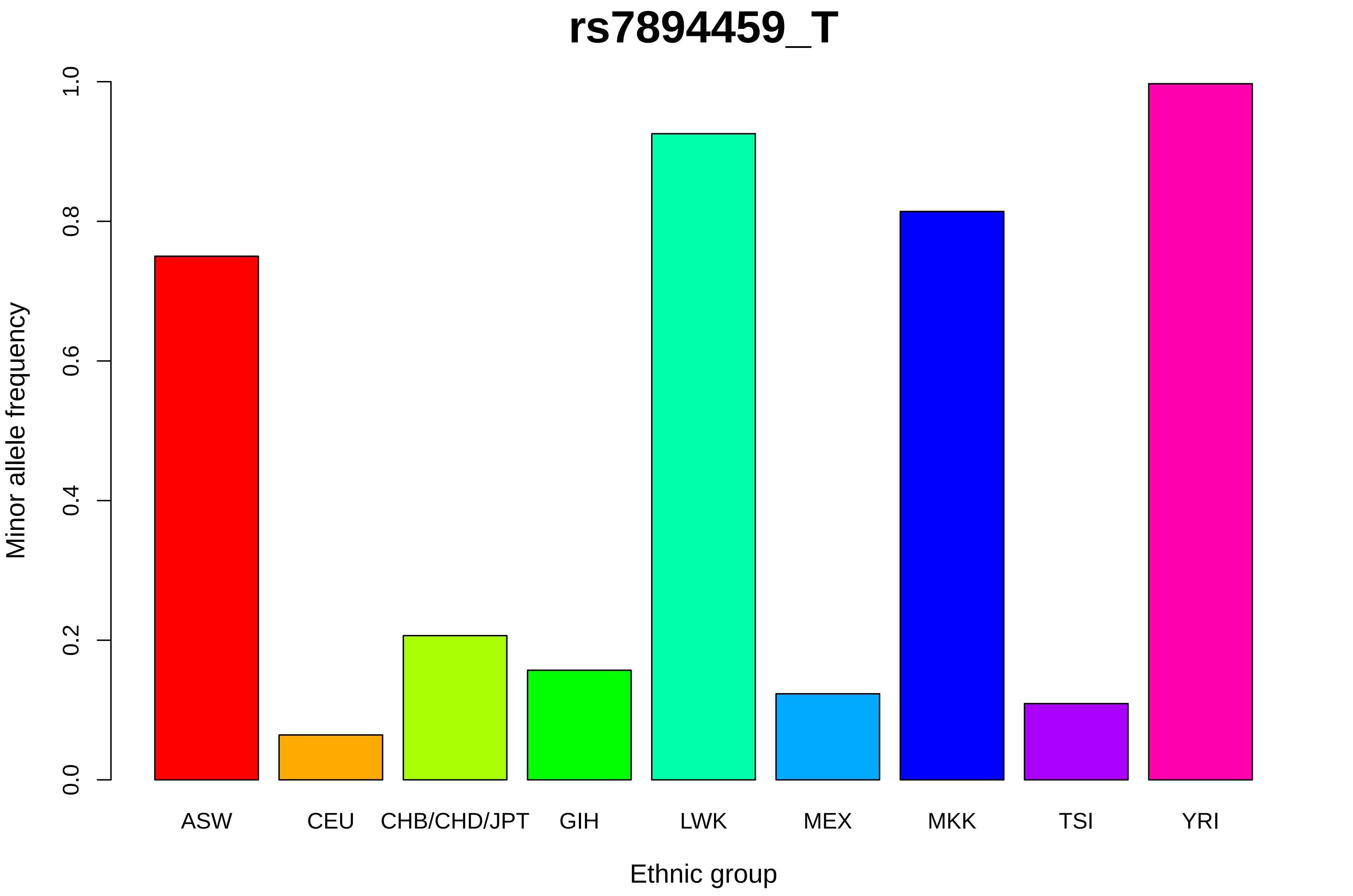

rs7894459_T
Minor allele frequency
0.0
0.2
0.4
0.6
0.8
1.0
ASW
CEU
CHB/CHD/JPT
GIH
LWK
MEX
MKK
TSI
YRI
Ethnic group

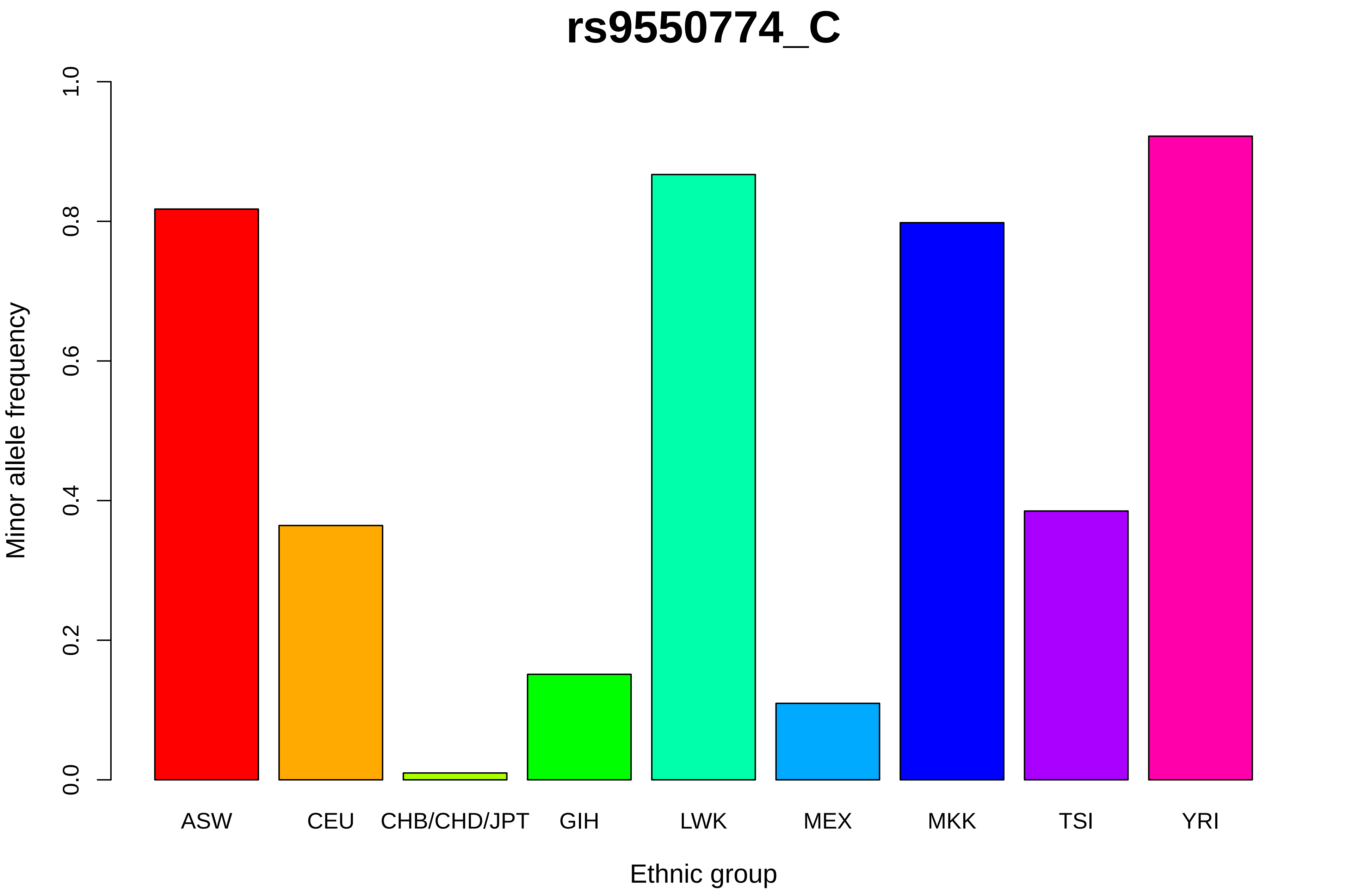
rs9550774_C
Minor allele frequency
0.0
0.2
0.4
0.6
0.8
1.0
ASW
CEU
CHB/CHD/JPT
GIH
LWK
MEX
MKK
TSI
YRI
Ethnic group

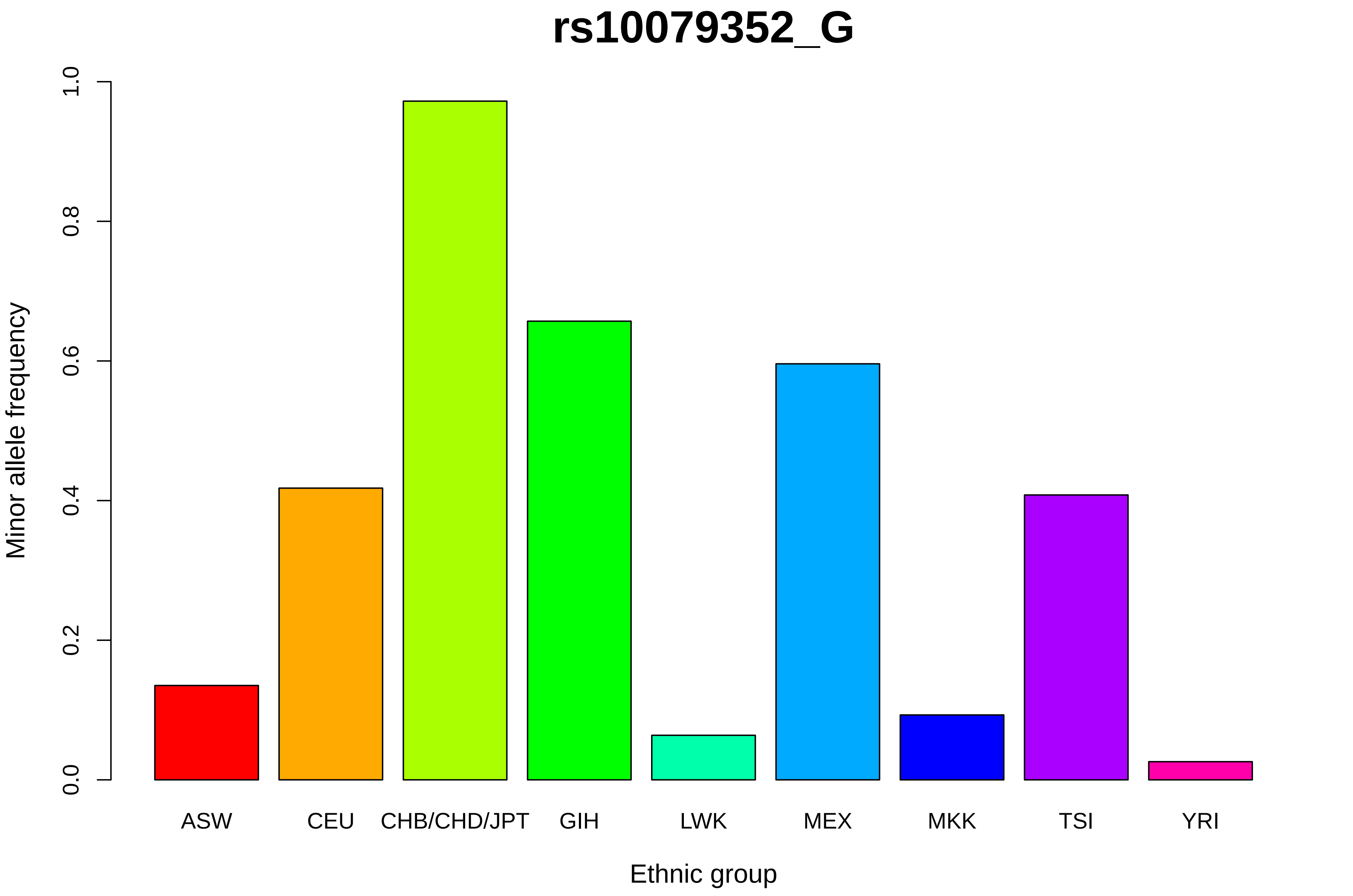
rs10079352_G
Minor allele frequency
0.0
0.2
0.4
0.6
0.8
1.0
ASW
CEU
CHB/CHD/JPT
GIH
LWK
MEX
MKK
TSI
YRI
Ethnic group

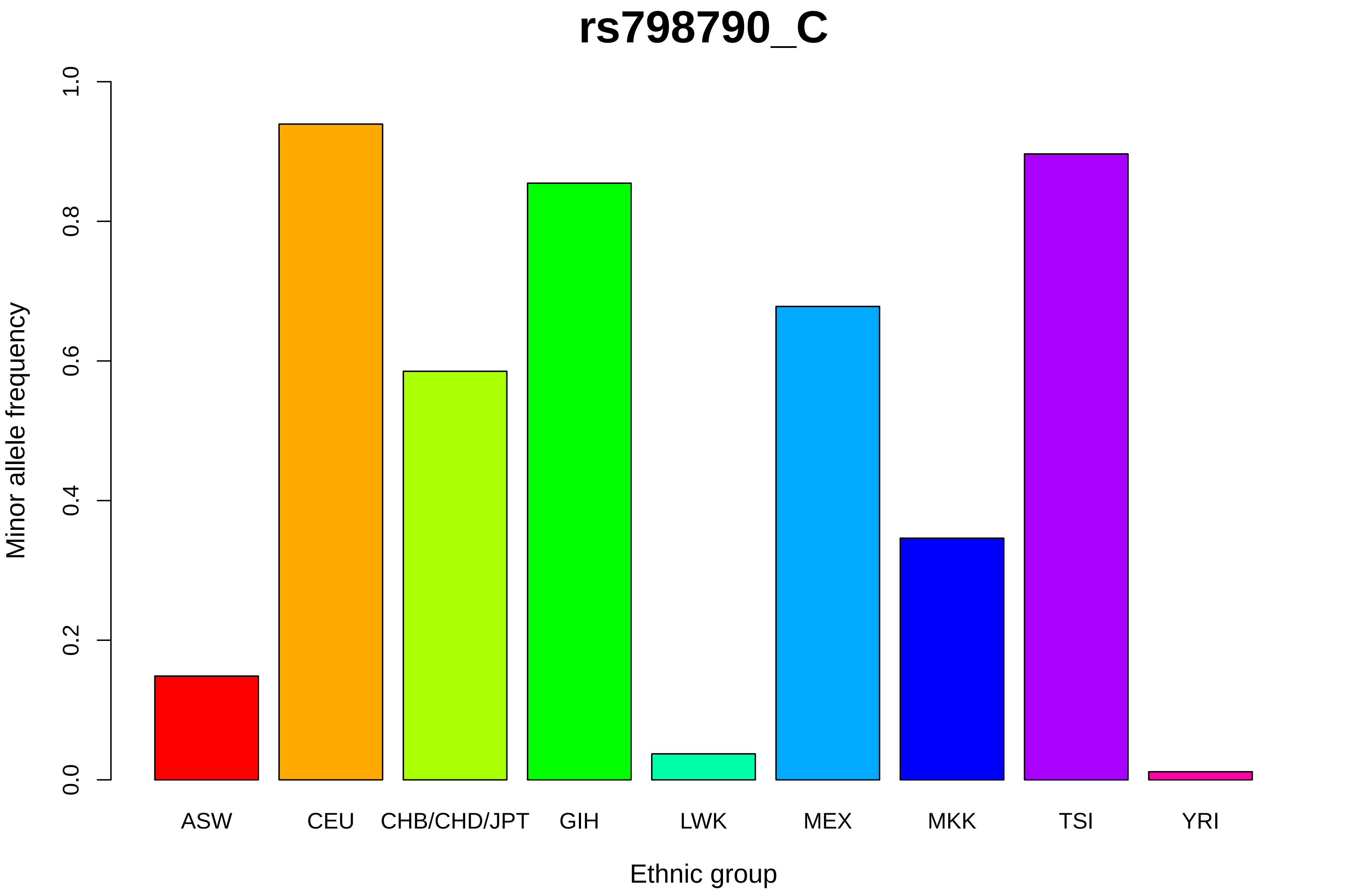
rs798790_C
Minor allele frequency
0.0
0.2
0.4
0.6
0.8
1.0
ASW
CEU
CHB/CHD/JPT
GIH
LWK
MEX
MKK
TSI
YRI
Ethnic group

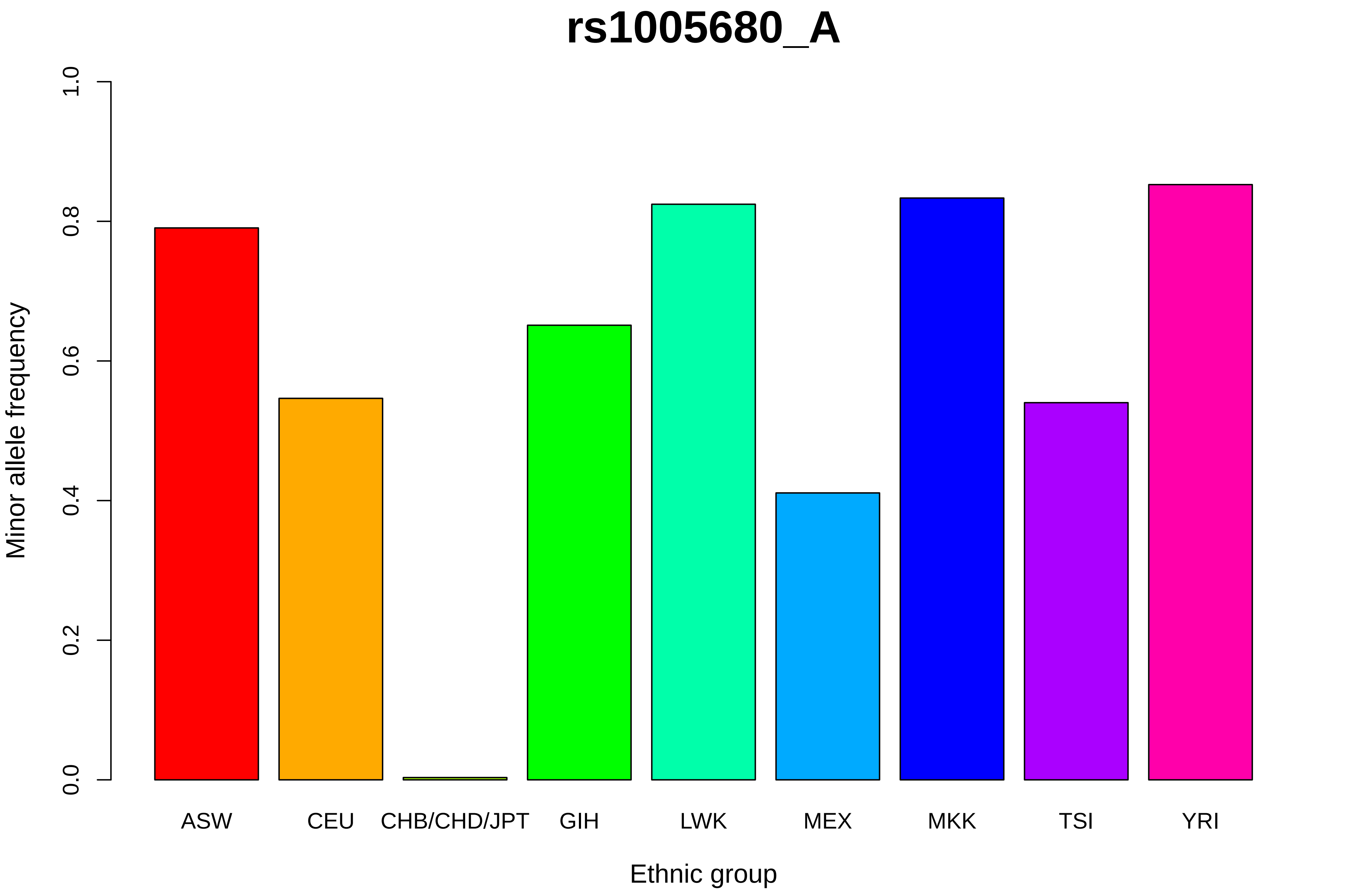
rs1005680_A
Minor allele frequency
0.0
0.2
0.4
0.6
0.8
1.0
ASW
CEU
CHB/CHD/JPT
GIH
LWK
MEX
MKK
TSI
YRI
Ethnic group

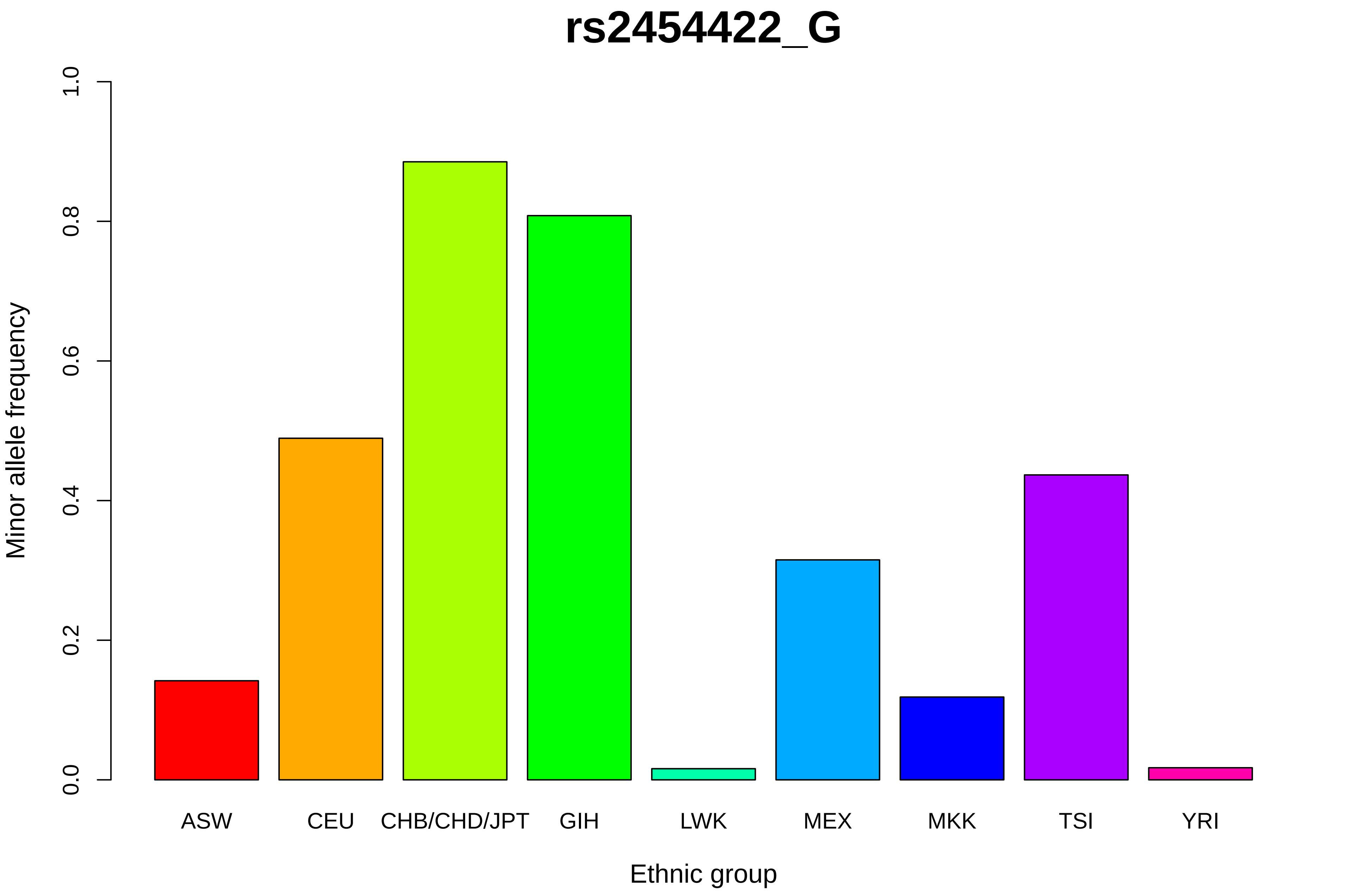
rs2454422_G
Minor allele frequency
0.0
0.2
0.4
0.6
0.8
1.0
ASW
CEU
CHB/CHD/JPT
GIH
LWK
MEX
MKK
TSI
YRI
Ethnic group

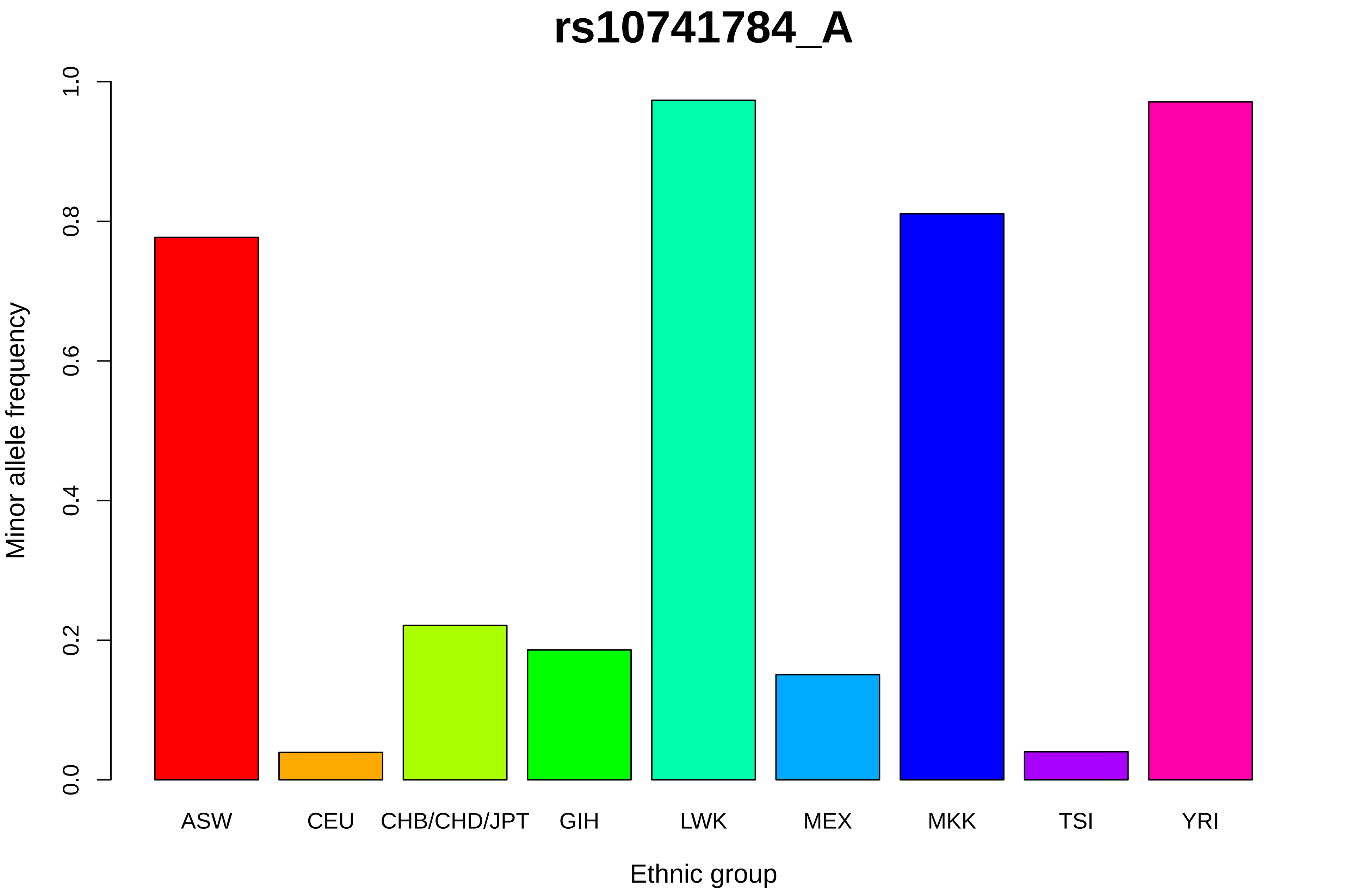

rs10741784_A
Minor allele frequency
0.0
0.2
0.4
0.6
0.8
1.0
ASW
CEU
CHB/CHD/JPT
GIH
LWK
MEX
MKK
TSI
YRI
Ethnic group

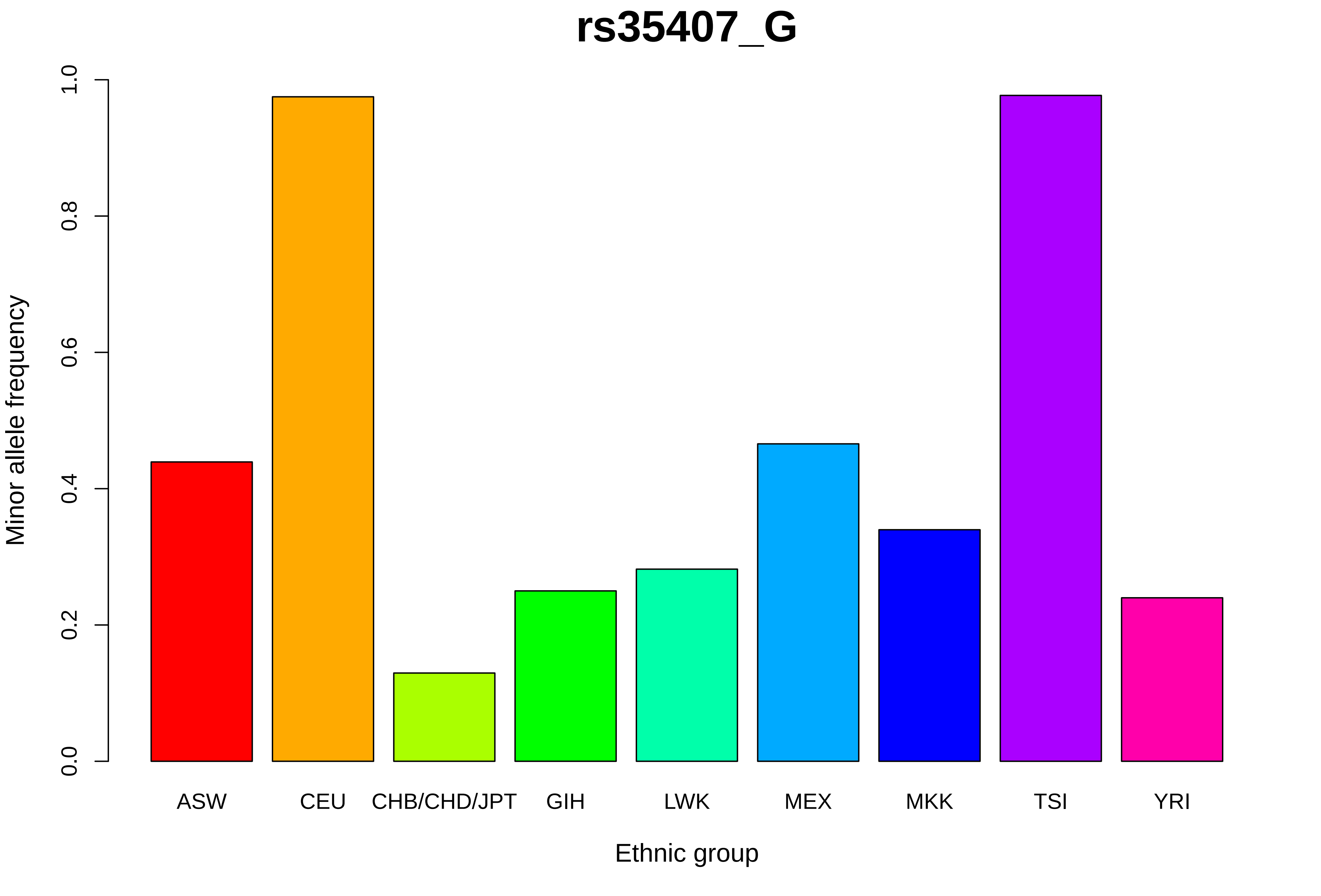
rs35407_G
Minor allele frequency
0.0
0.2
0.4
0.6
0.8
1.0
ASW
CEU
CHB/CHD/JPT
GIH
LWK
MEX
MKK
TSI
YRI
Ethnic group

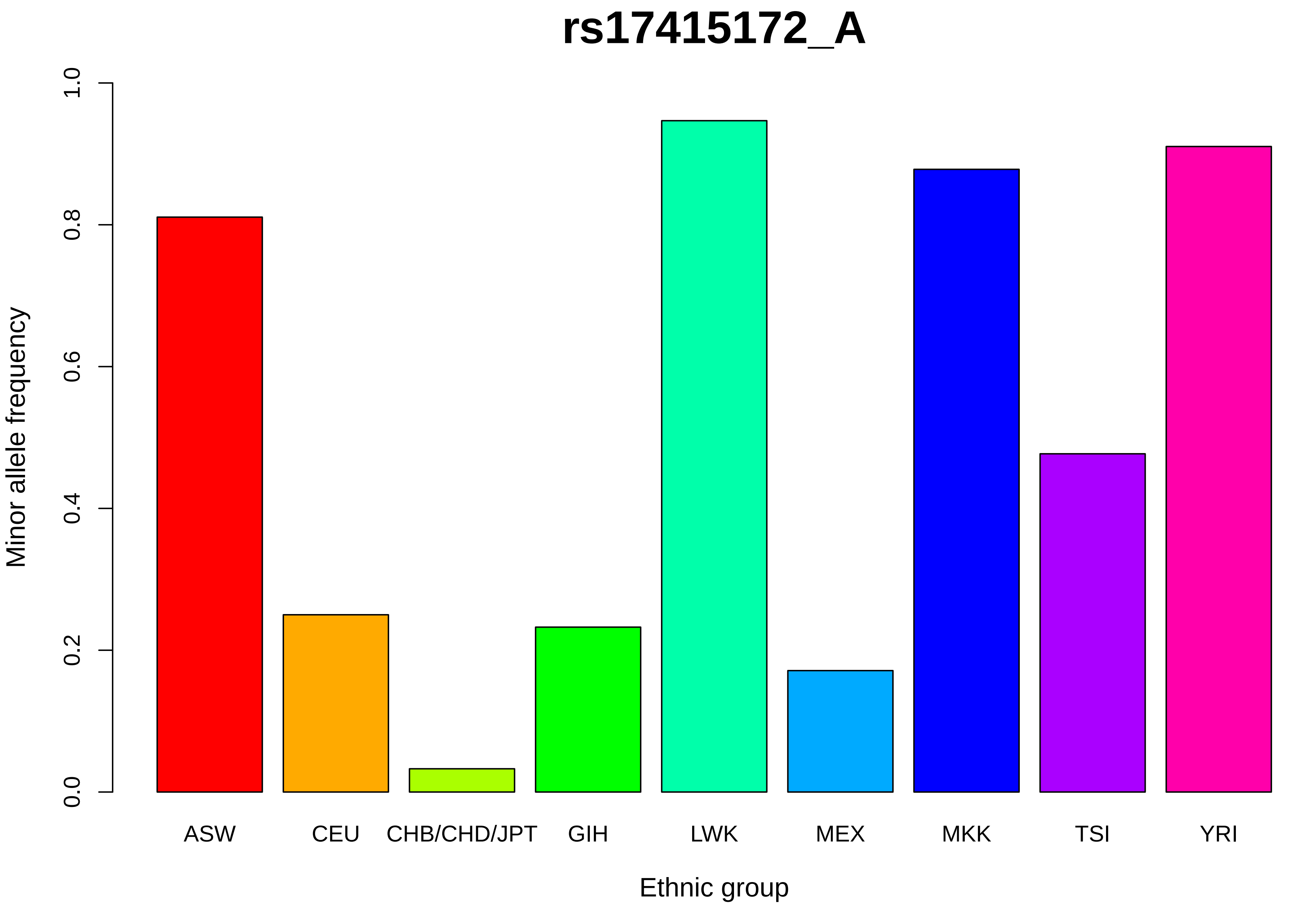
rs17415172_A
Minor allele frequency
0.0
0.2
0.4
0.6
0.8
1.0
ASW
CEU
CHB/CHD/JPT
GIH
LWK
MEX
MKK
TSI
YRI
Ethnic group

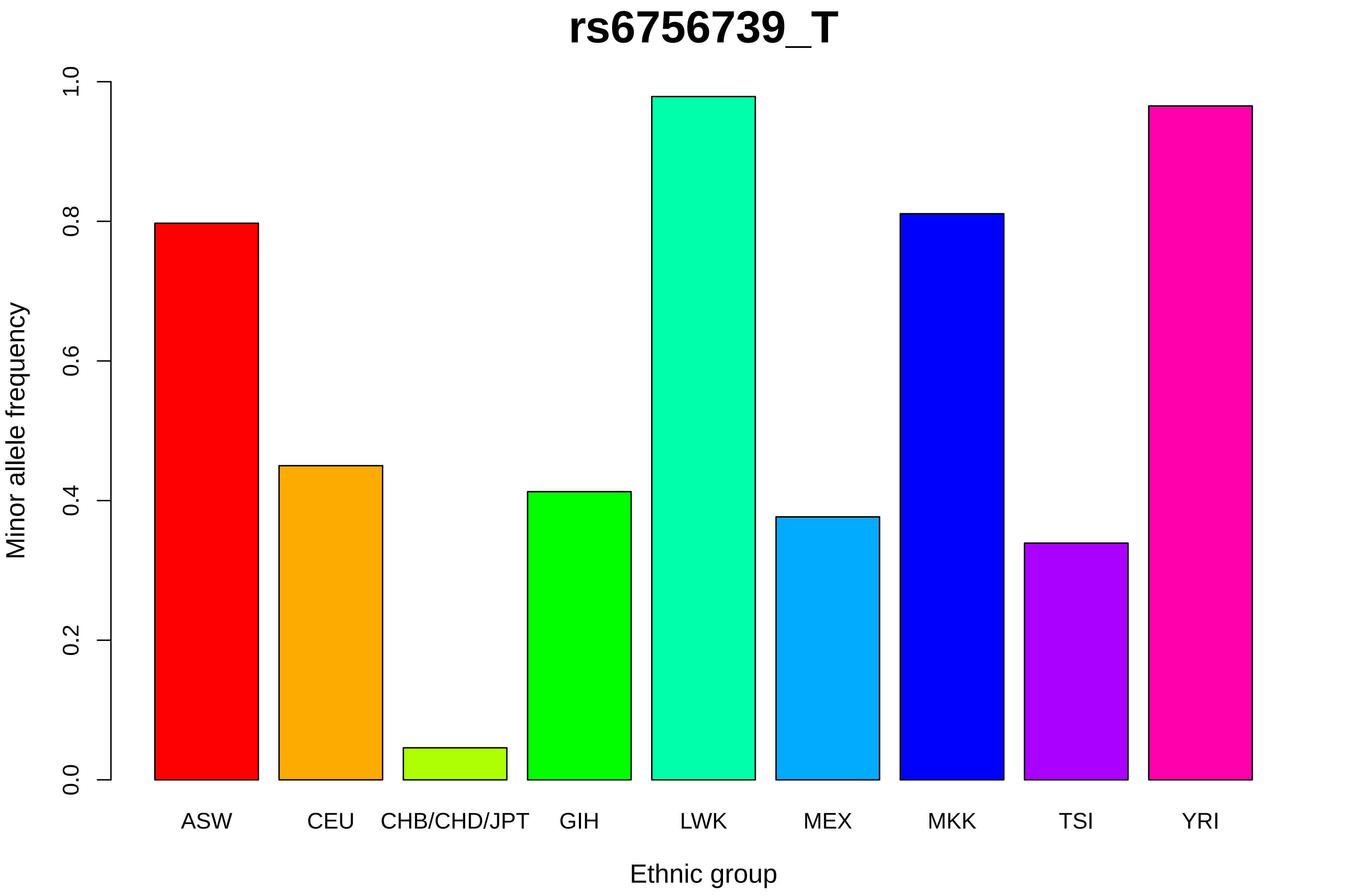
rs6756739_T
Minor allele frequency
0.0
0.2
0.4
0.6
0.8
1.0
ASW
CEU
CHB/CHD/JPT
GIH
LWK
MEX
MKK
TSI
YRI
Ethnic group

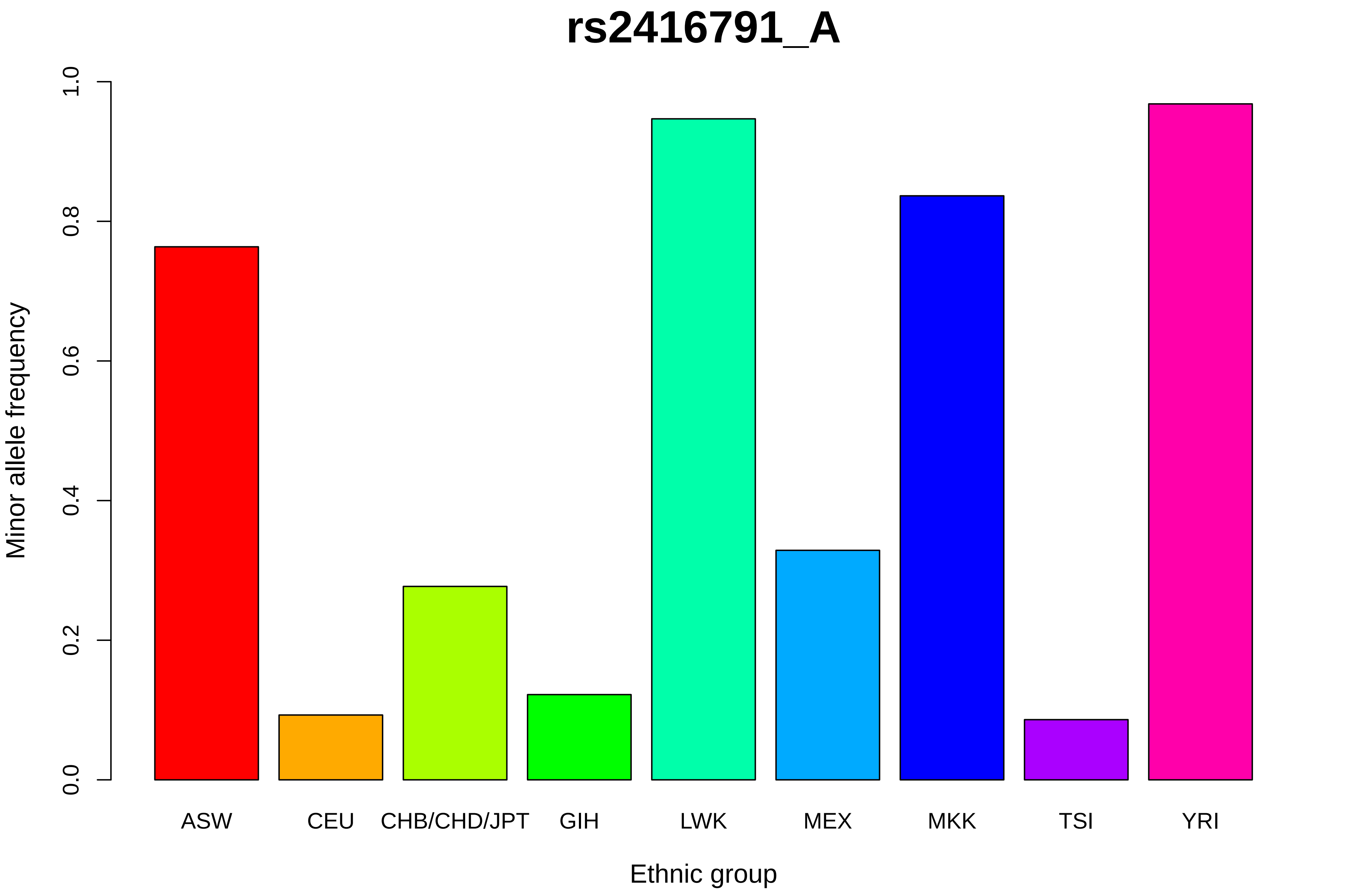
rs2416791_A
Minor allele frequency
0.0
0.2
0.4
0.6
0.8
1.0
ASW
CEU
CHB/CHD/JPT
GIH
LWK
MEX
MKK
TSI
YRI
Ethnic group

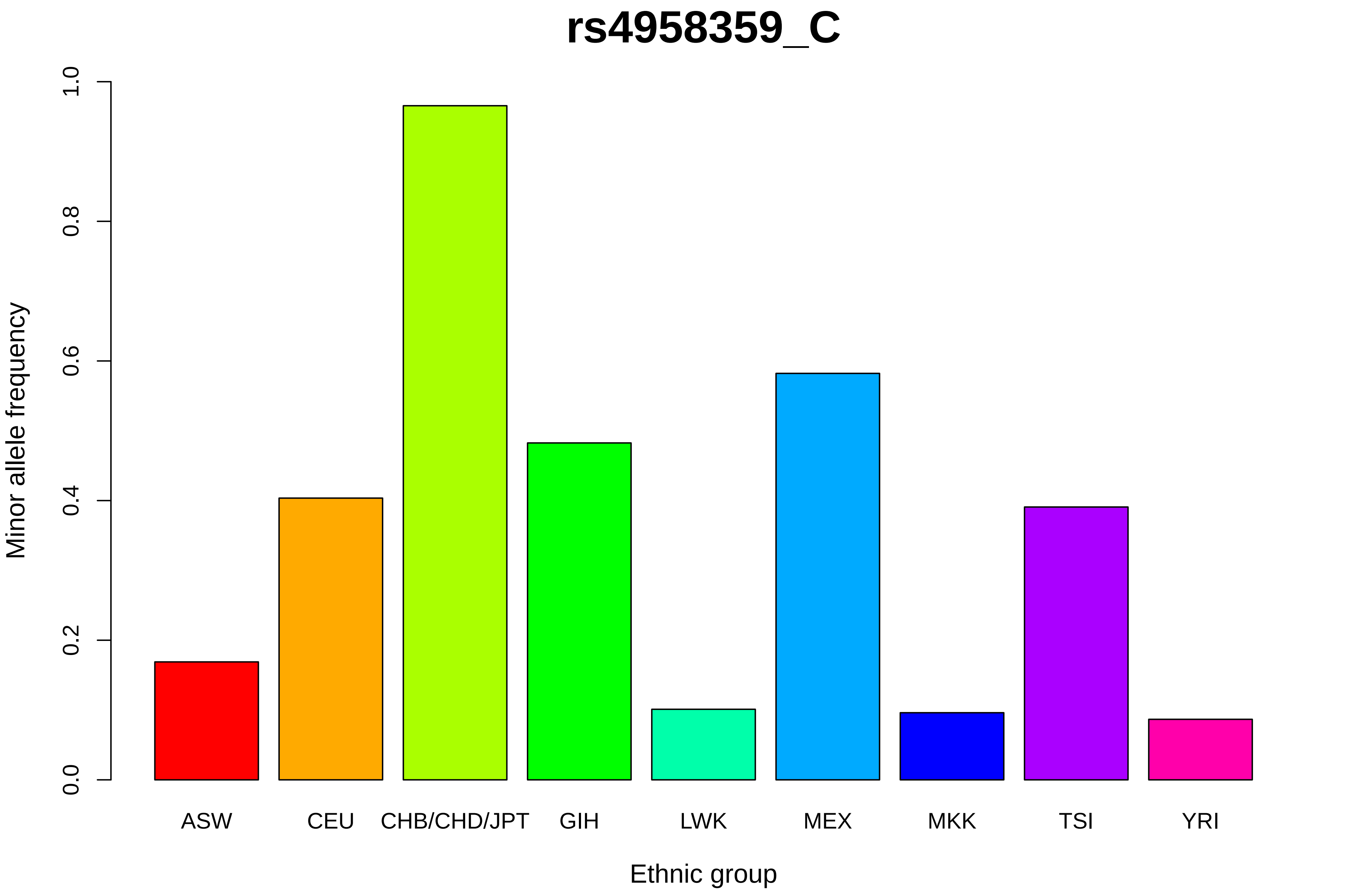

rs4958359_C
Minor allele frequency
0.0
0.2
0.4
0.6
0.8
1.0
ASW
CEU
CHB/CHD/JPT
GIH
LWK
MEX
MKK
TSI
YRI
Ethnic group

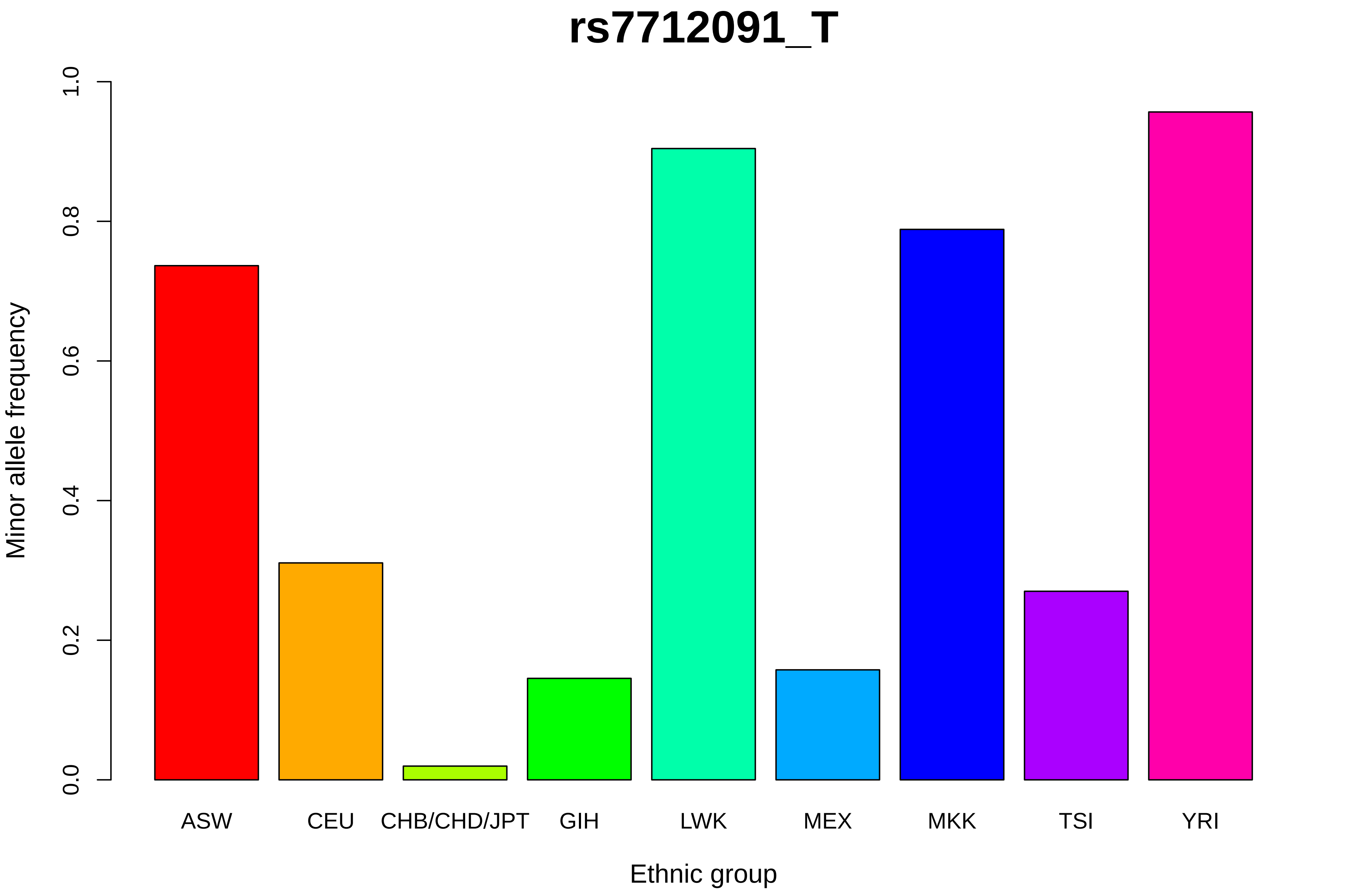
rs7712091_T
Minor allele frequency
0.0
0.2
0.4
0.6
0.8
1.0
ASW
CEU
CHB/CHD/JPT
GIH
LWK
MEX
MKK
TSI
YRI
Ethnic group

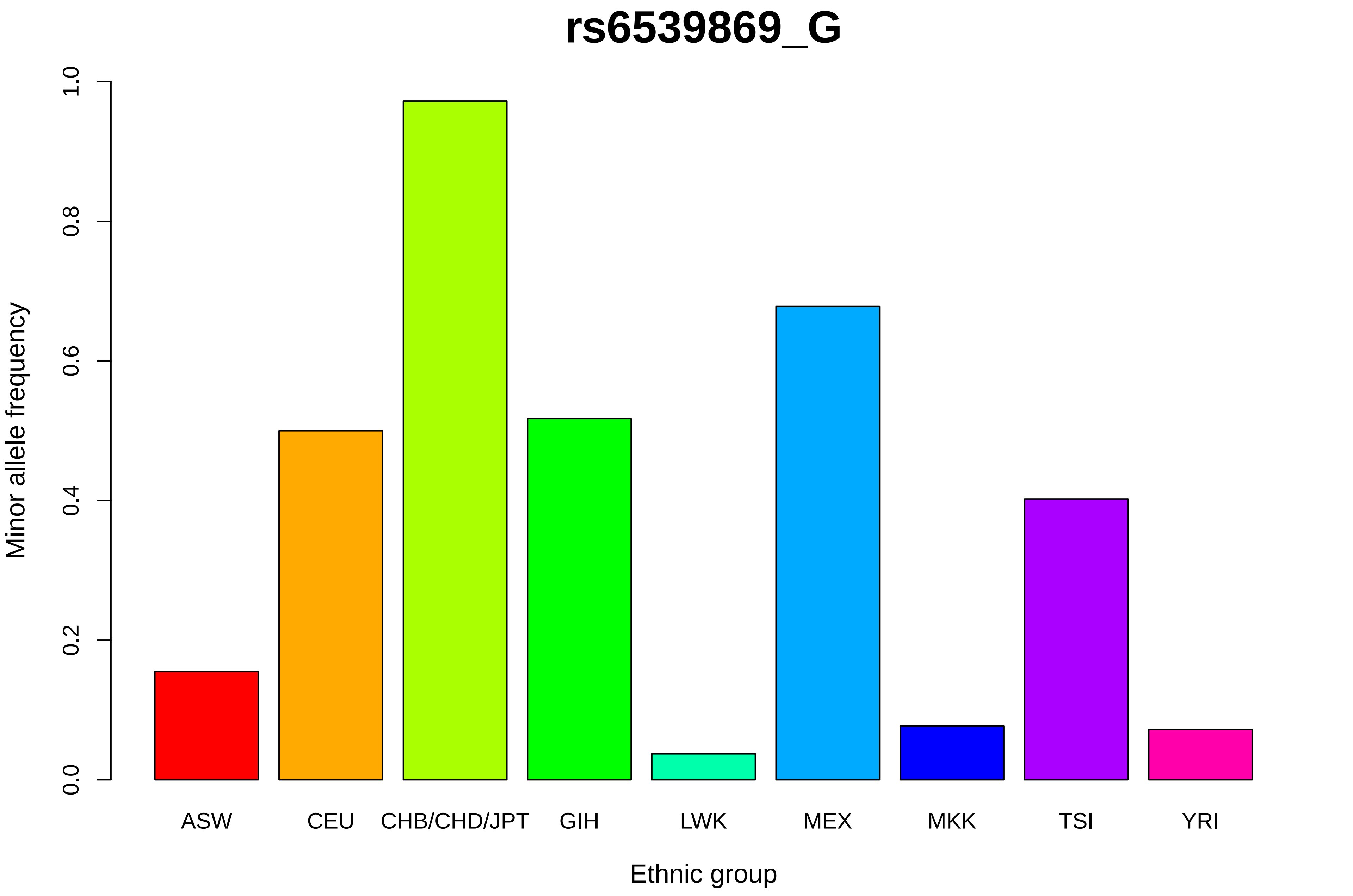

rs6539869_G
Minor allele frequency
0.0
0.2
0.4
0.6
0.8
1.0
ASW
CEU
CHB/CHD/JPT
GIH
LWK
MEX
MKK
TSI
YRI
Ethnic group

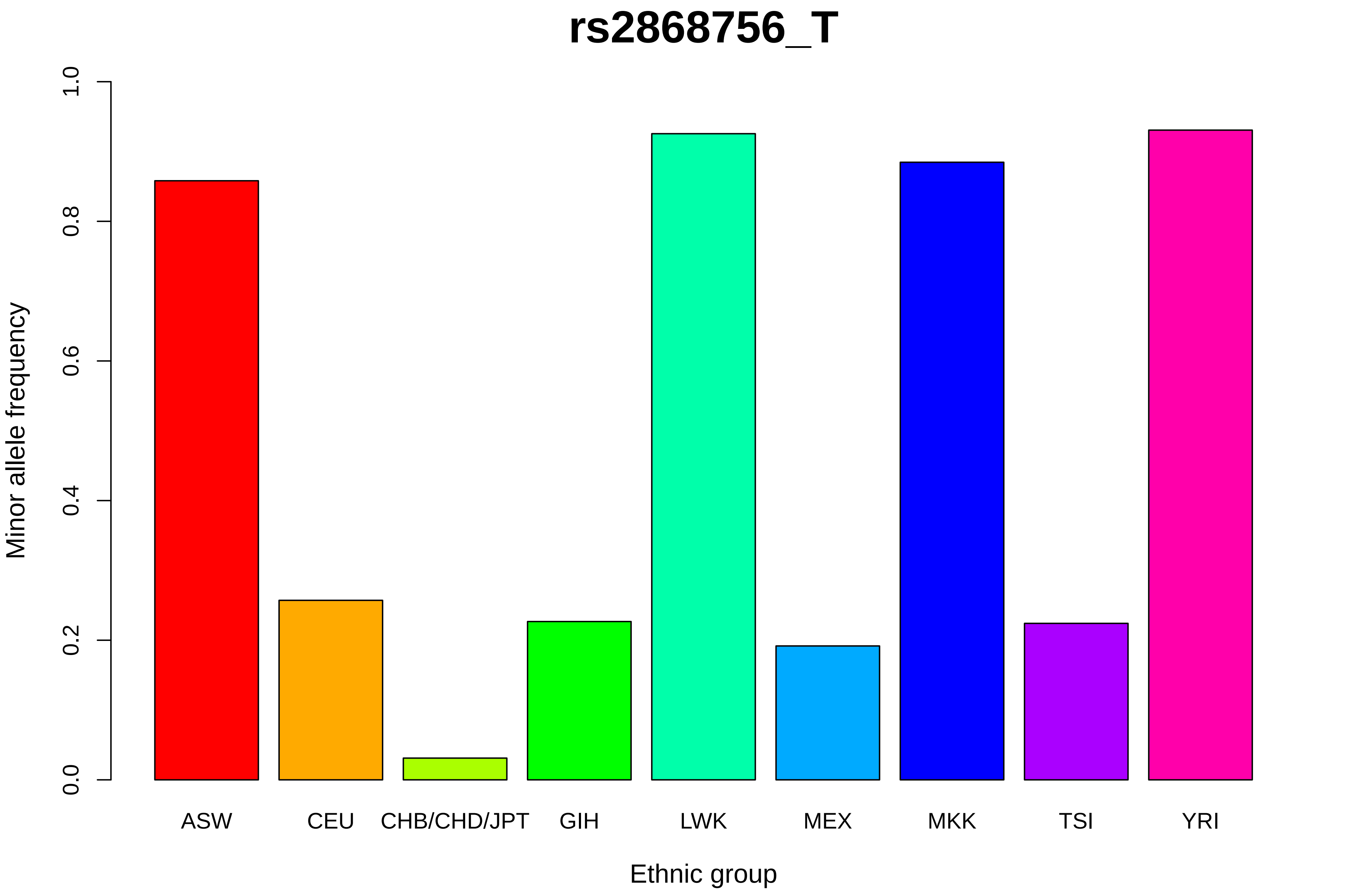
rs2868756_T
Minor allele frequency
0.0
0.2
0.4
0.6
0.8
1.0
ASW
CEU
CHB/CHD/JPT
GIH
LWK
MEX
MKK
TSI
YRI
Ethnic group

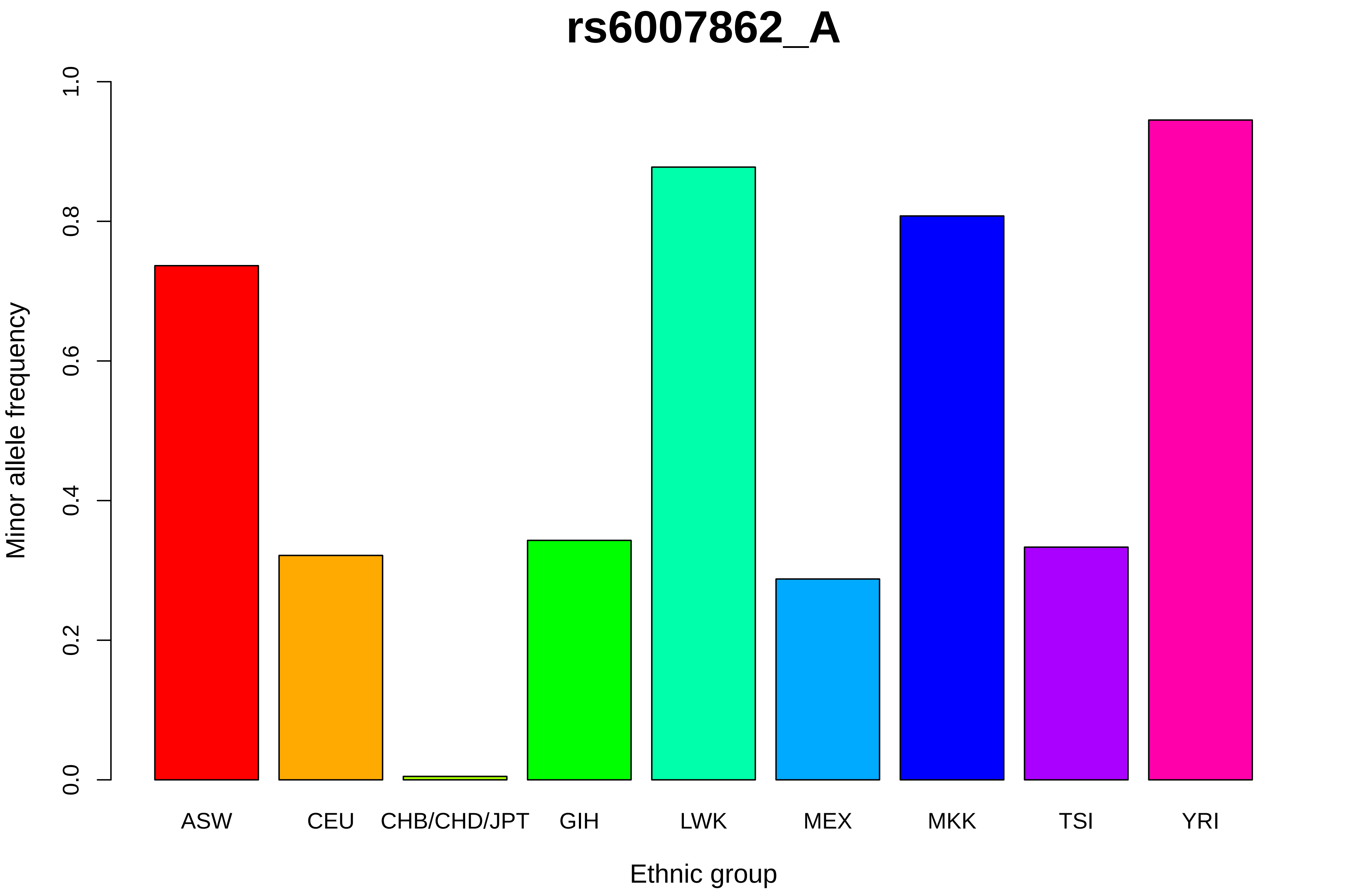
rs6007862_A
Minor allele frequency
0.0
0.2
0.4
0.6
0.8
1.0
ASW
CEU
CHB/CHD/JPT
GIH
LWK
MEX
MKK
TSI
YRI
Ethnic group

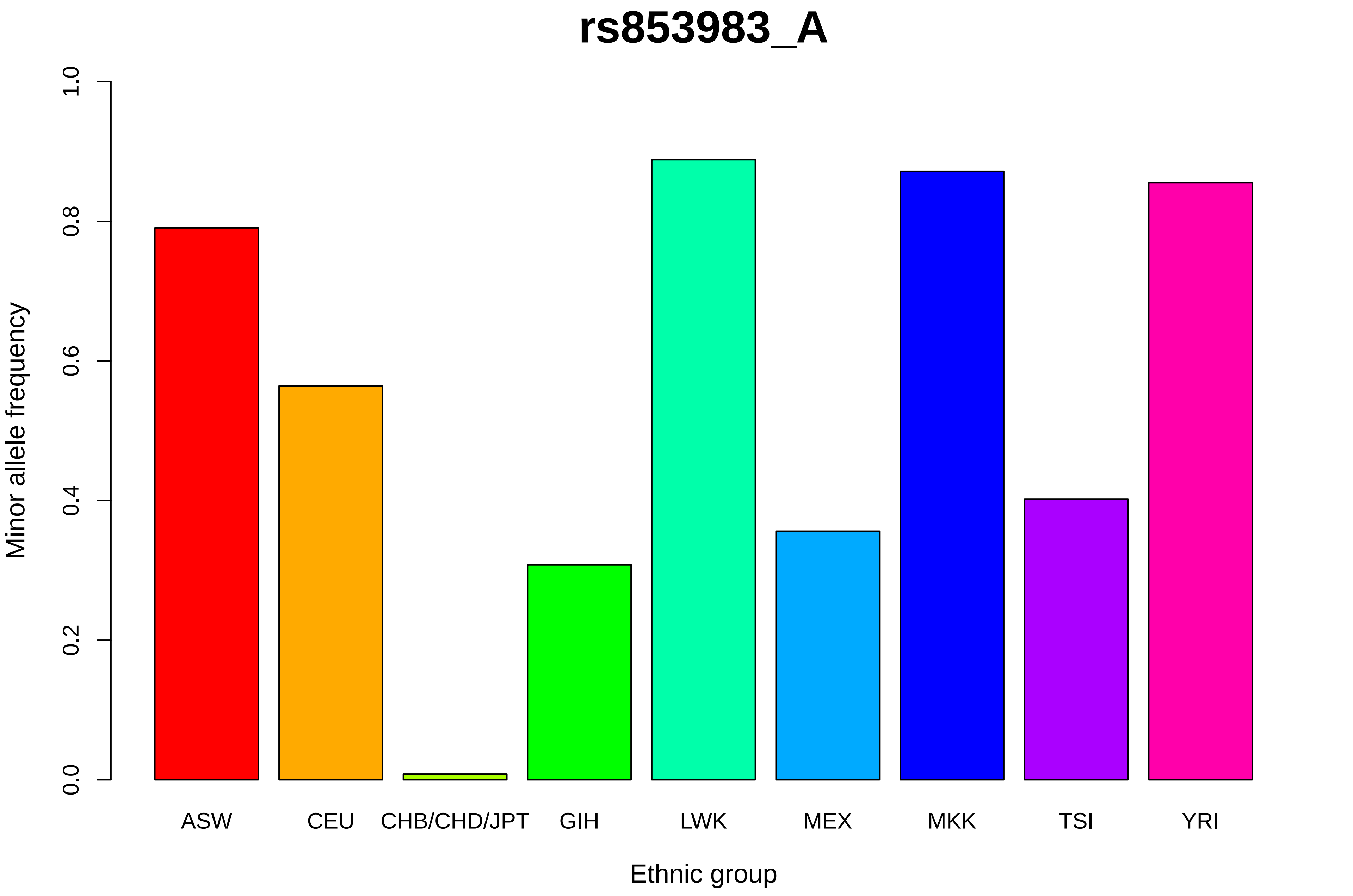
rs853983_A
Minor allele frequency
0.0
0.2
0.4
0.6
0.8
1.0
ASW
CEU
CHB/CHD/JPT
GIH
LWK
MEX
MKK
TSI
YRI
Ethnic group

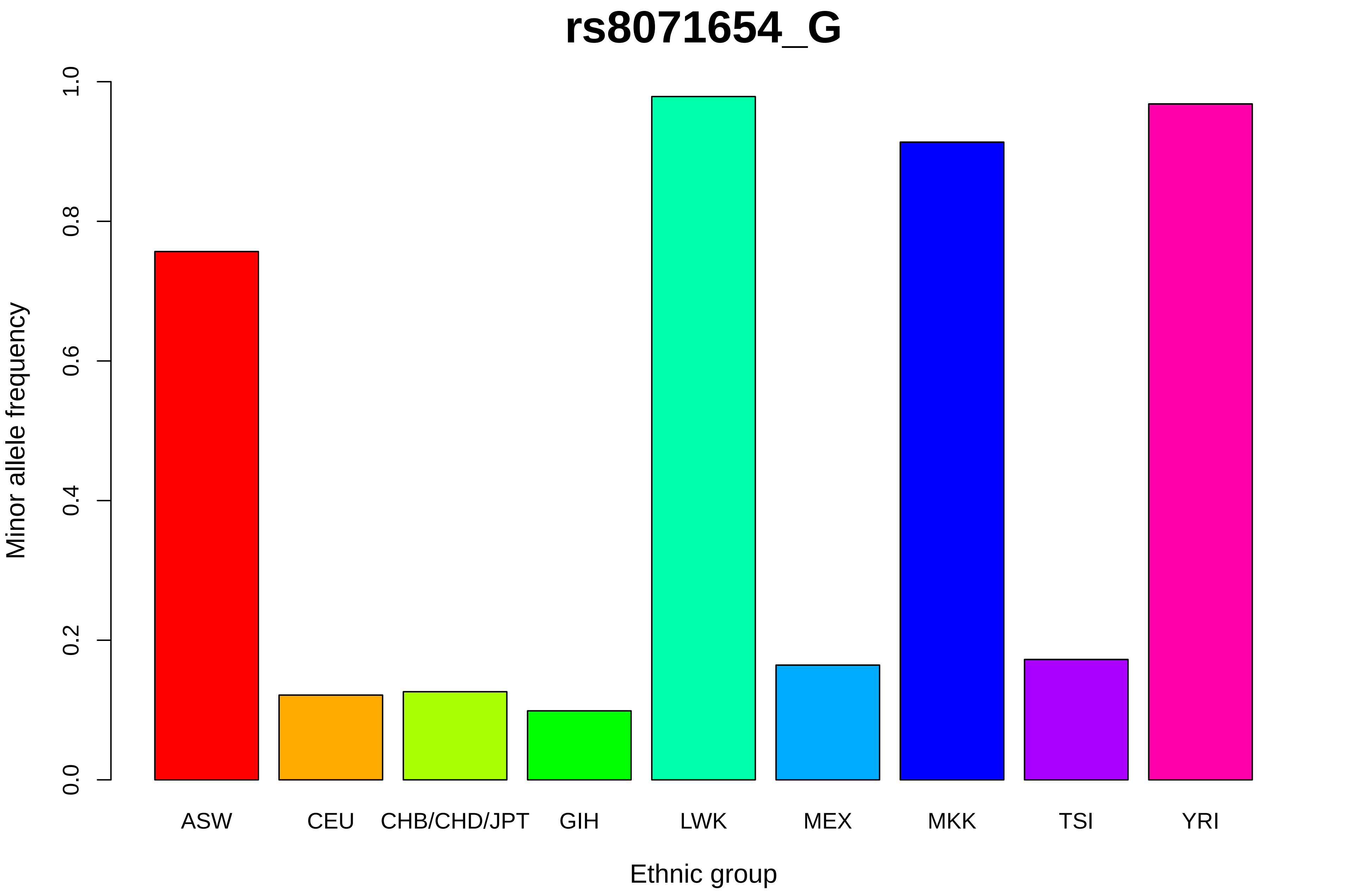

rs8071654_G
Minor allele frequency
0.0
0.2
0.4
0.6
0.8
1.0
ASW
CEU
CHB/CHD/JPT
GIH
LWK
MEX
MKK
TSI
YRI
Ethnic group

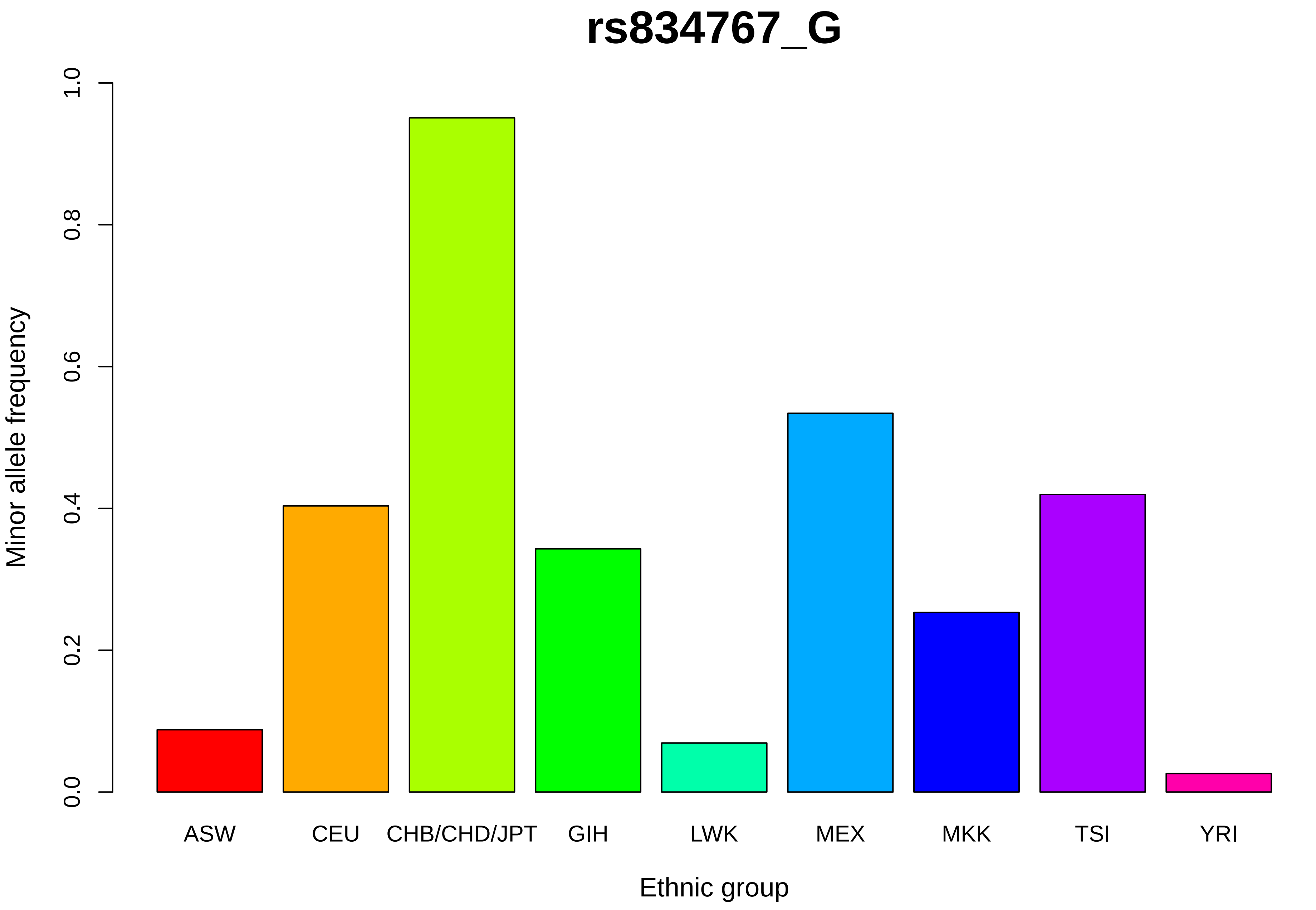
rs834767_G
Minor allele frequency
0.0
0.2
0.4
0.6
0.8
1.0
ASW
CEU
CHB/CHD/JPT
GIH
LWK
MEX
MKK
TSI
YRI
Ethnic group

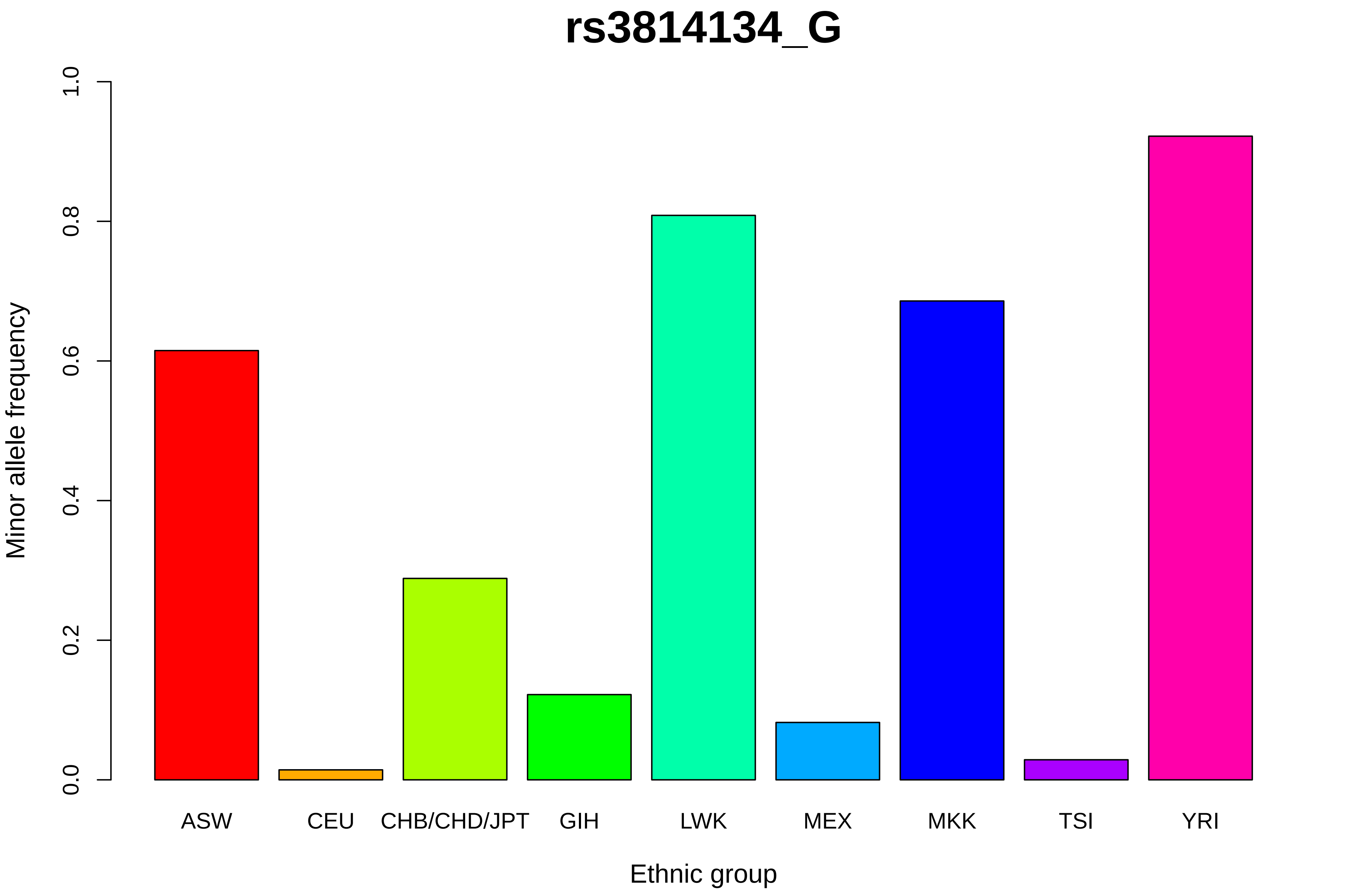
rs3814134_G
Minor allele frequency
0.0
0.2
0.4
0.6
0.8
1.0
ASW
CEU
CHB/CHD/JPT
GIH
LWK
MEX
MKK
TSI
YRI
Ethnic group

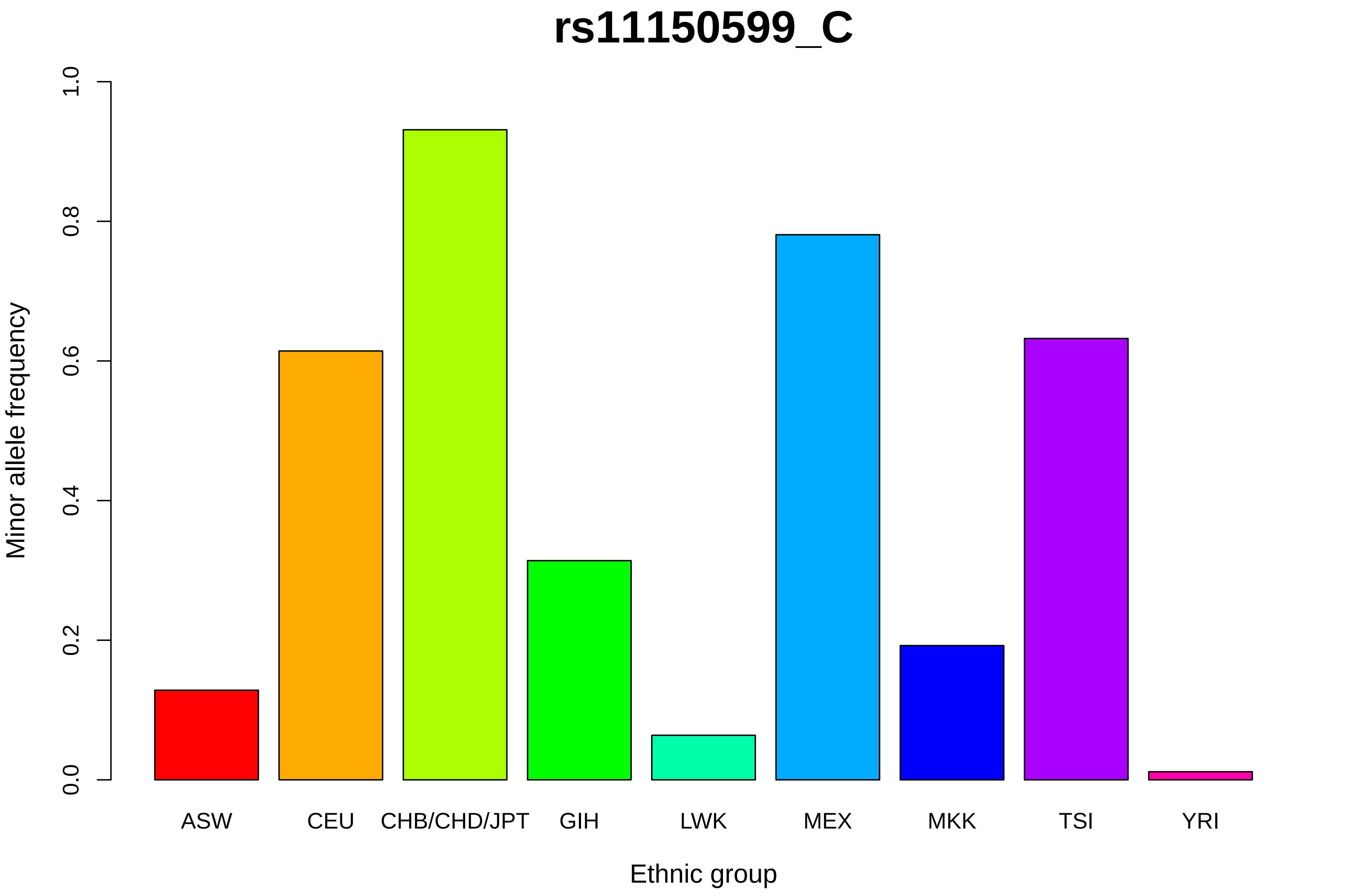
rs11150599_C
Minor allele frequency
0.0
0.2
0.4
0.6
0.8
1.0
ASW
CEU
CHB/CHD/JPT
GIH
LWK
MEX
MKK
TSI
YRI
Ethnic group

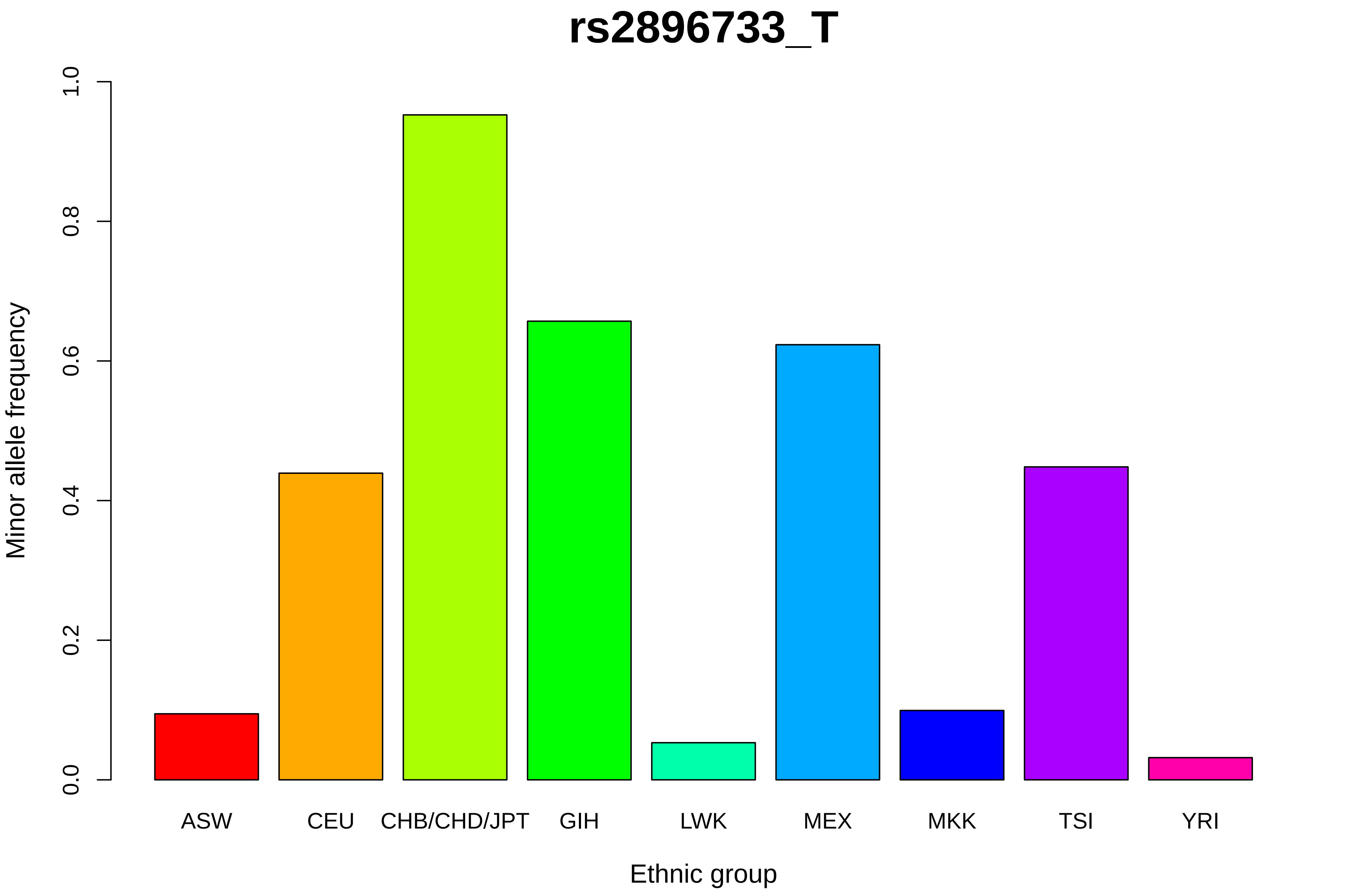
rs2896733_T
Minor allele frequency
0.0
0.2
0.4
0.6
0.8
1.0
ASW
CEU
CHB/CHD/JPT
GIH
LWK
MEX
MKK
TSI
YRI
Ethnic group

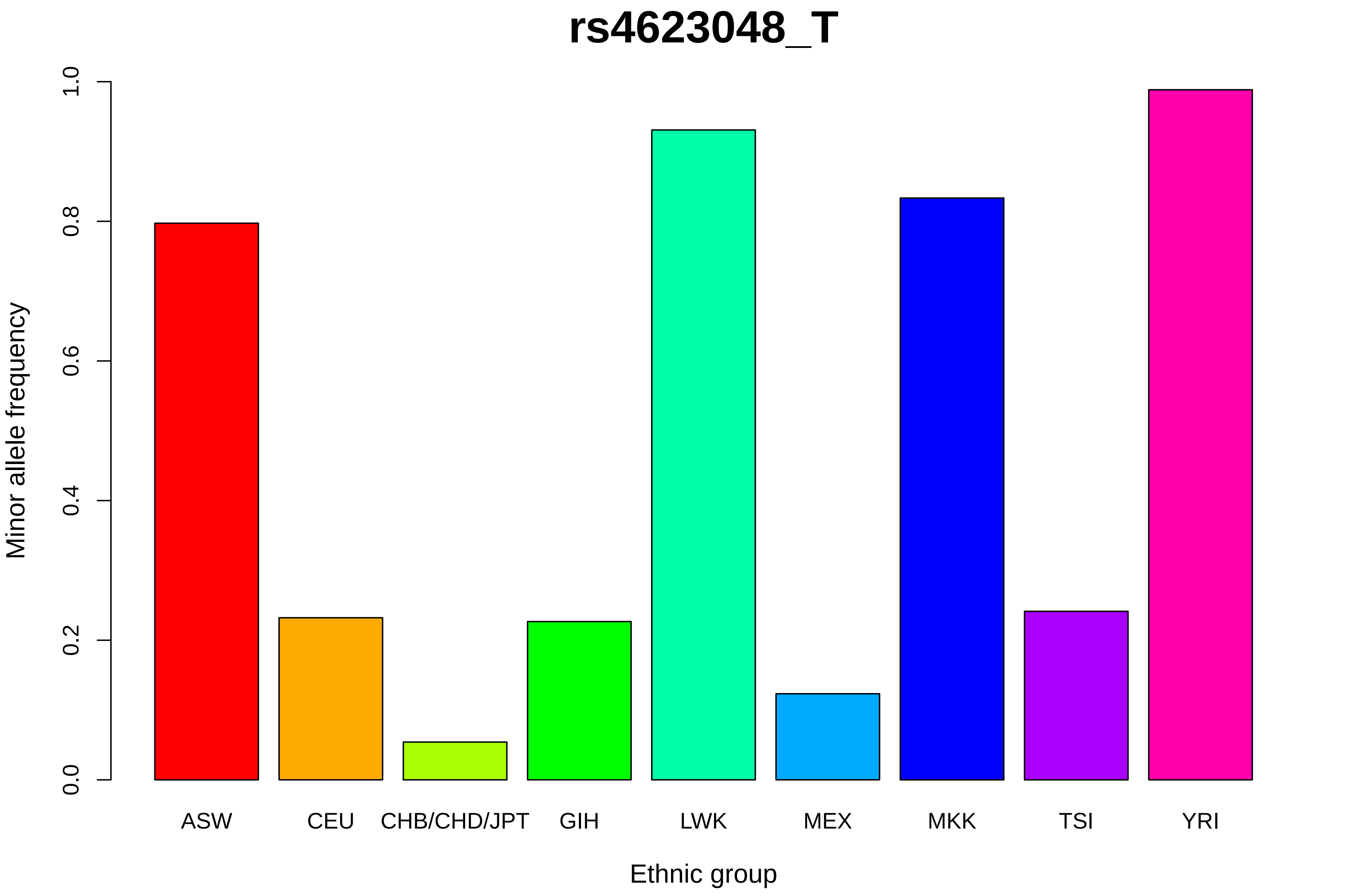
rs4623048_T
Minor allele frequency
0.0
0.2
0.4
0.6
0.8
1.0
ASW
CEU
CHB/CHD/JPT
GIH
LWK
MEX
MKK
TSI
YRI
Ethnic group

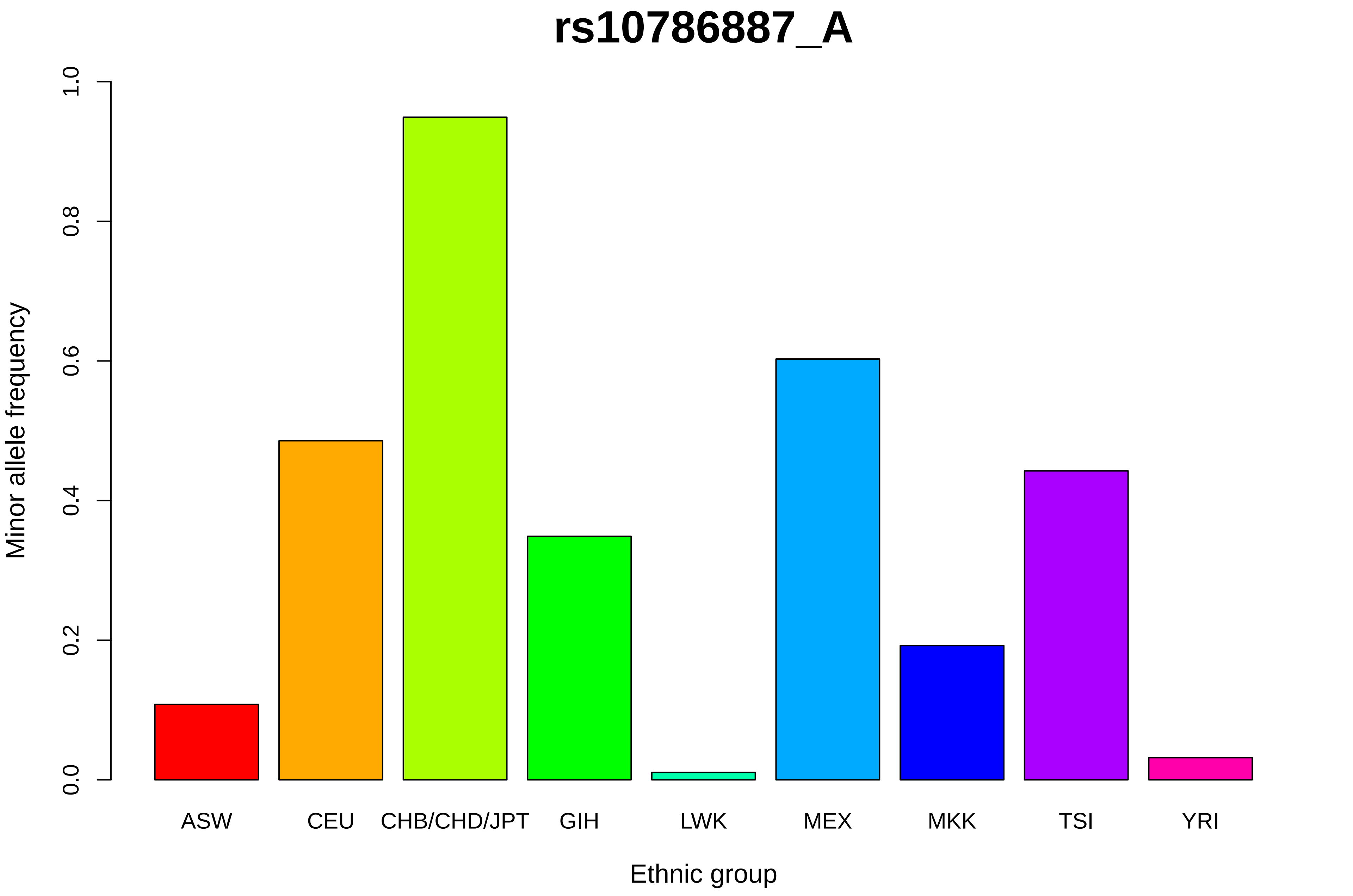

rs10786887_A
Minor allele frequency
0.0
0.2
0.4
0.6
0.8
1.0
ASW
CEU
CHB/CHD/JPT
GIH
LWK
MEX
MKK
TSI
YRI
Ethnic group

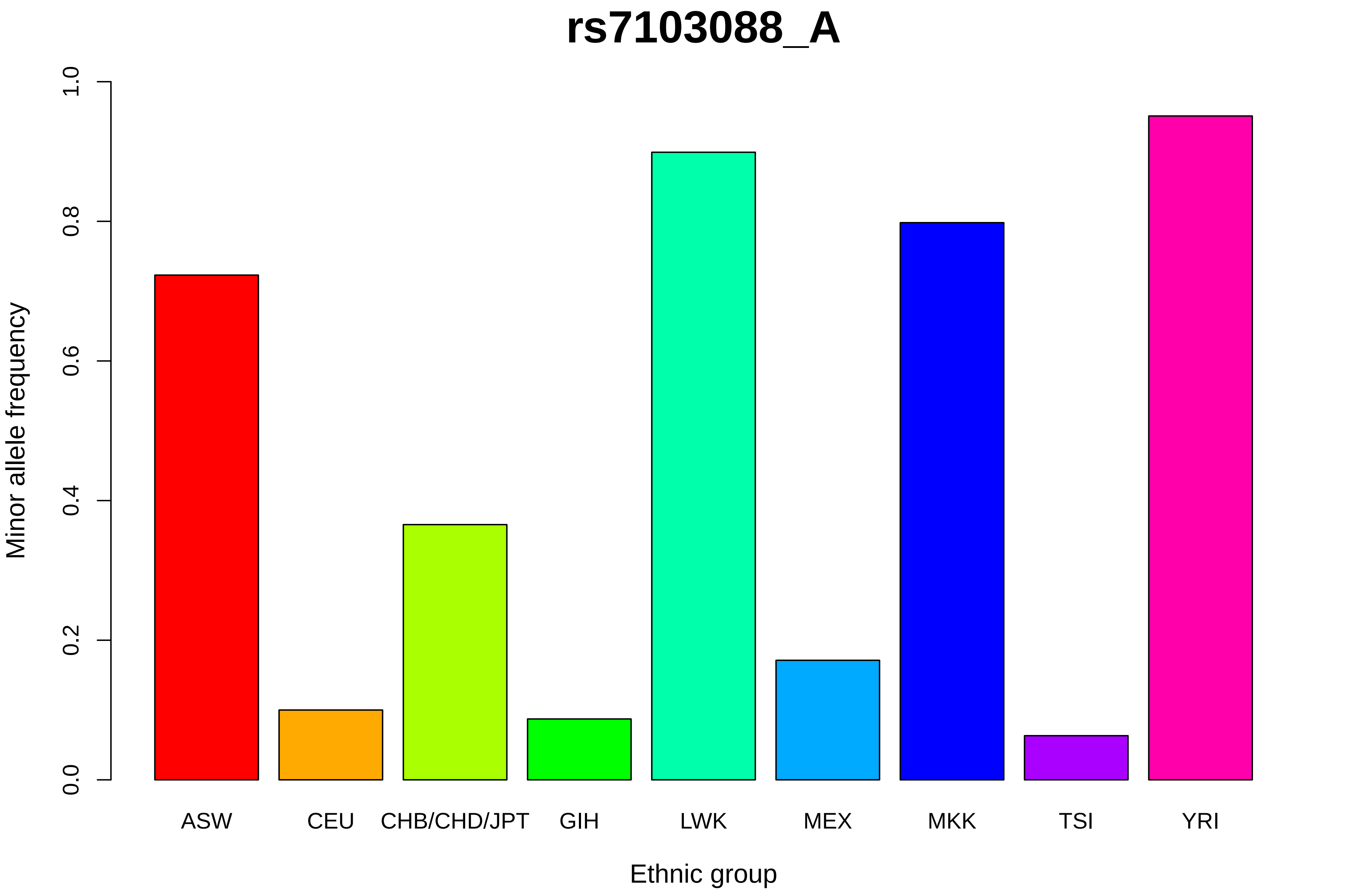
rs7103088_A
Minor allele frequency
0.0
0.2
0.4
0.6
0.8
1.0
ASW
CEU
CHB/CHD/JPT
GIH
LWK
MEX
MKK
TSI
YRI
Ethnic group

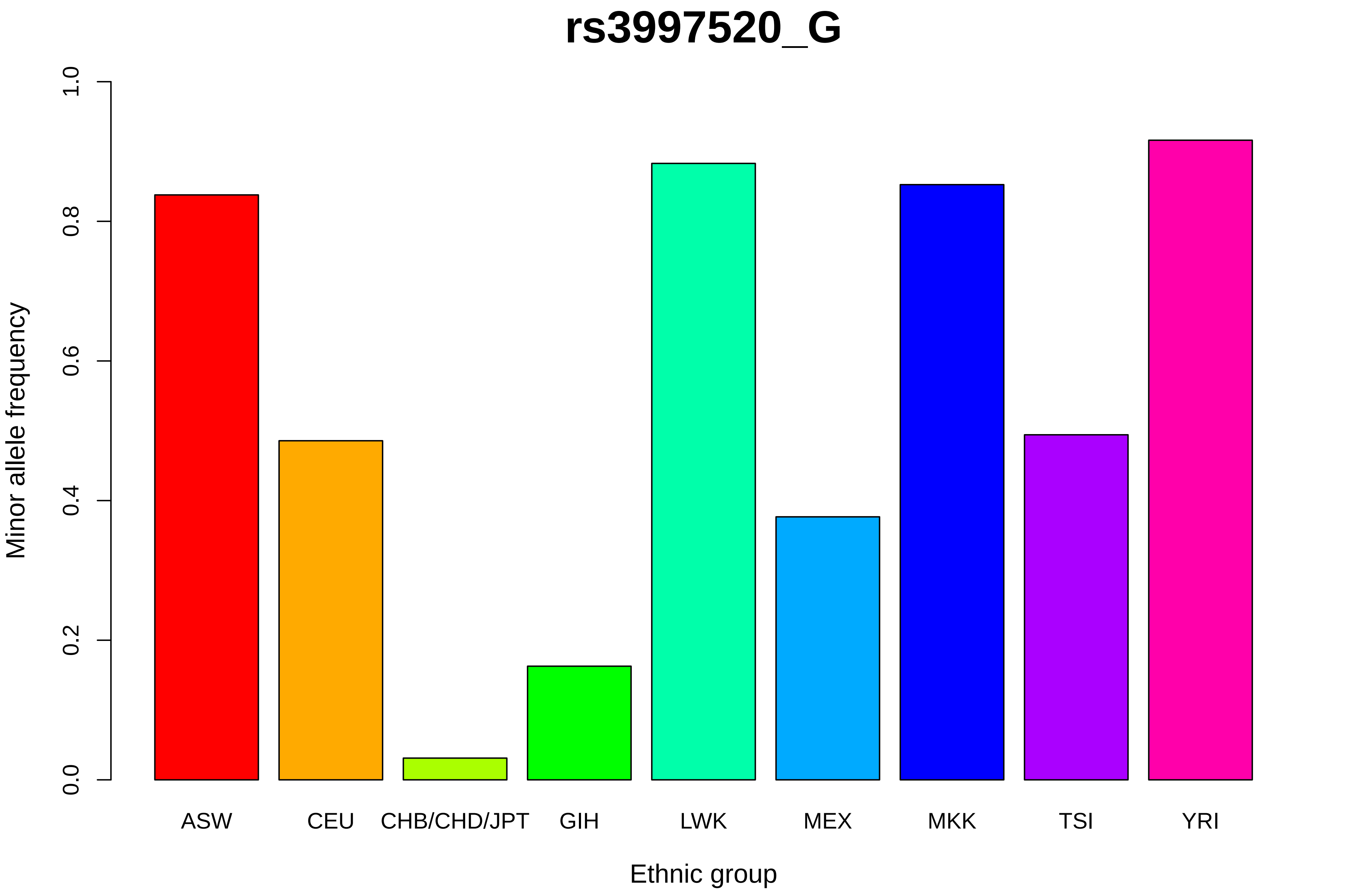
rs3997520_G
Minor allele frequency
0.0
0.2
0.4
0.6
0.8
1.0
ASW
CEU
CHB/CHD/JPT
GIH
LWK
MEX
MKK
TSI
YRI
Ethnic group

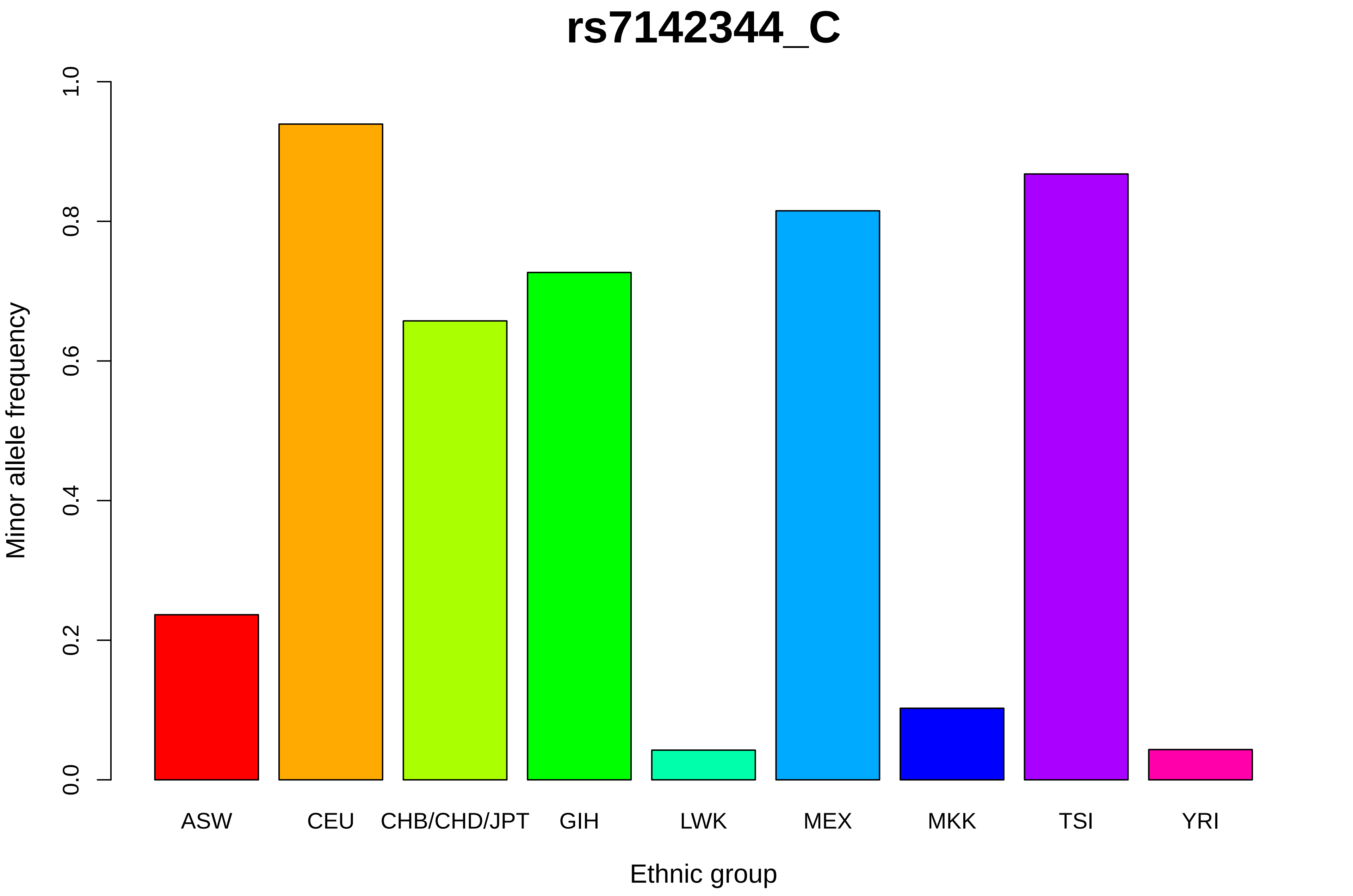
rs7142344_C
Minor allele frequency
0.0
0.2
0.4
0.6
0.8
1.0
ASW
CEU
CHB/CHD/JPT
GIH
LWK
MEX
MKK
TSI
YRI
Ethnic group

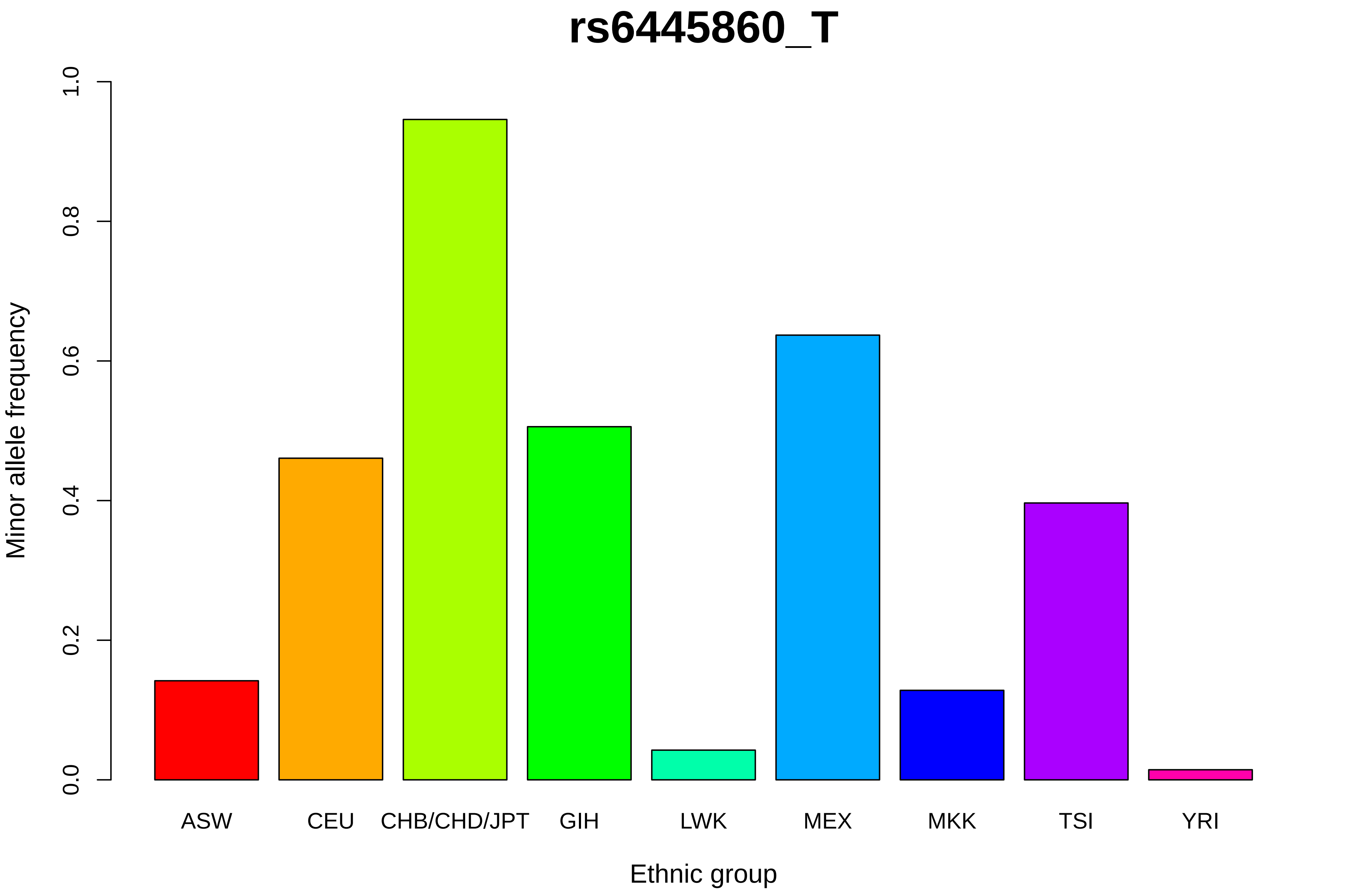
rs6445860_T
Minor allele frequency
0.0
0.2
0.4
0.6
0.8
1.0
ASW
CEU
CHB/CHD/JPT
GIH
LWK
MEX
MKK
TSI
YRI
Ethnic group

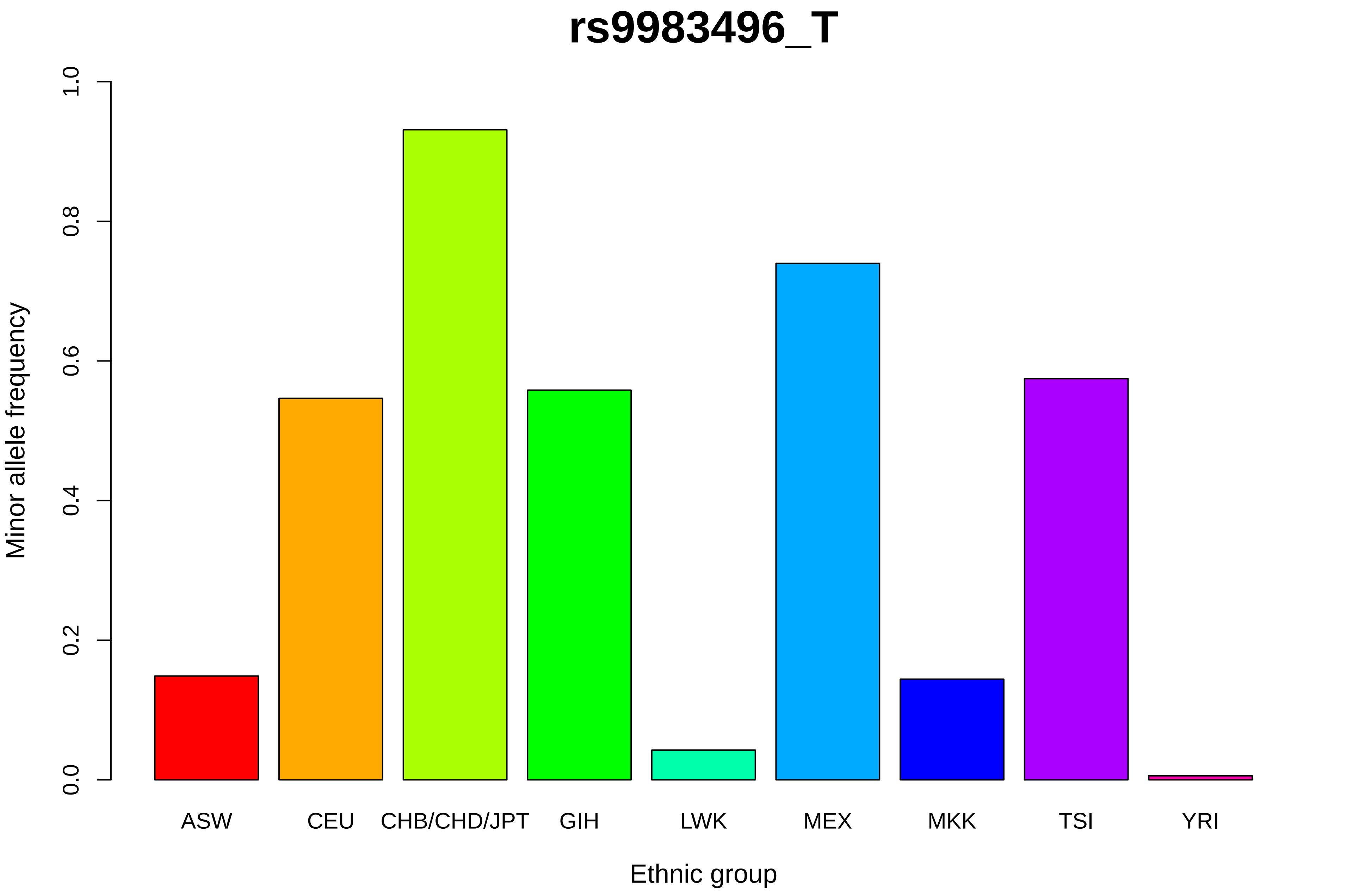
rs9983496_T
Minor allele frequency
0.0
0.2
0.4
0.6
0.8
1.0
ASW
CEU
CHB/CHD/JPT
GIH
LWK
MEX
MKK
TSI
YRI
Ethnic group

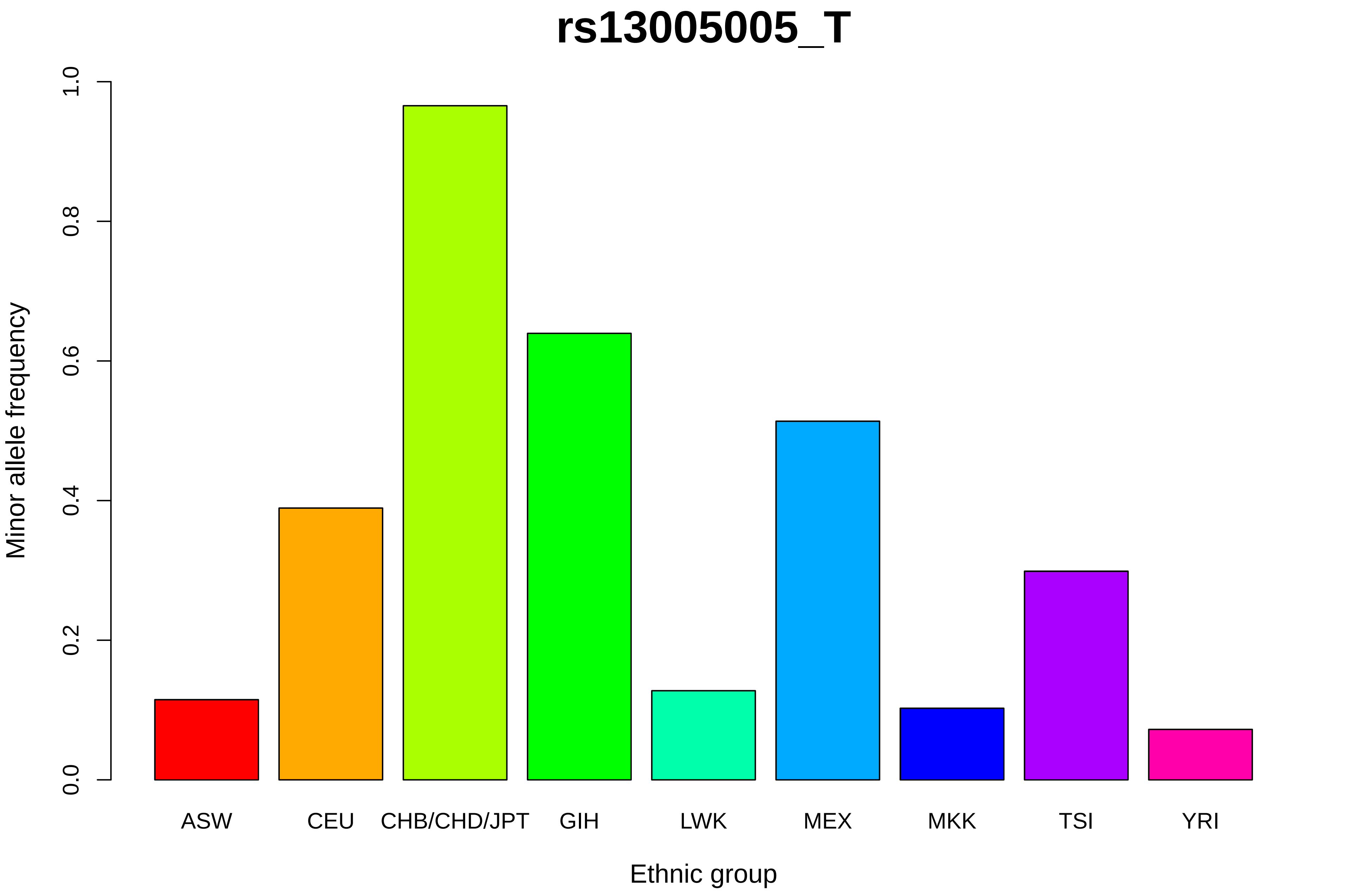

rs13005005_T
Minor allele frequency
0.0
0.2
0.4
0.6
0.8
1.0
ASW
CEU
CHB/CHD/JPT
GIH
LWK
MEX
MKK
TSI
YRI
Ethnic group

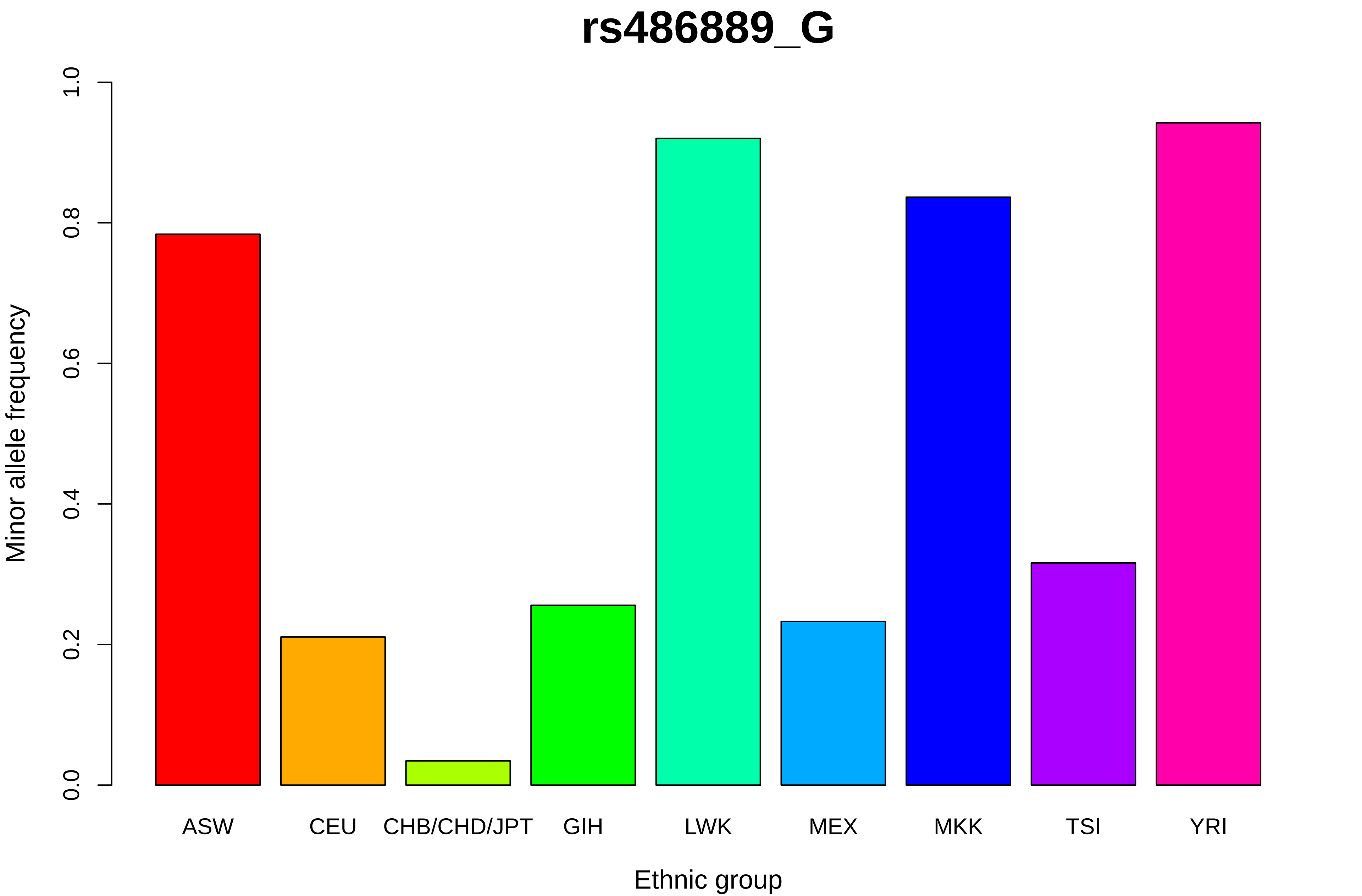
rs486889_G
Minor allele frequency
0.0
0.2
0.4
0.6
0.8
1.0
ASW
CEU
CHB/CHD/JPT
GIH
LWK
MEX
MKK
TSI
YRI
Ethnic group

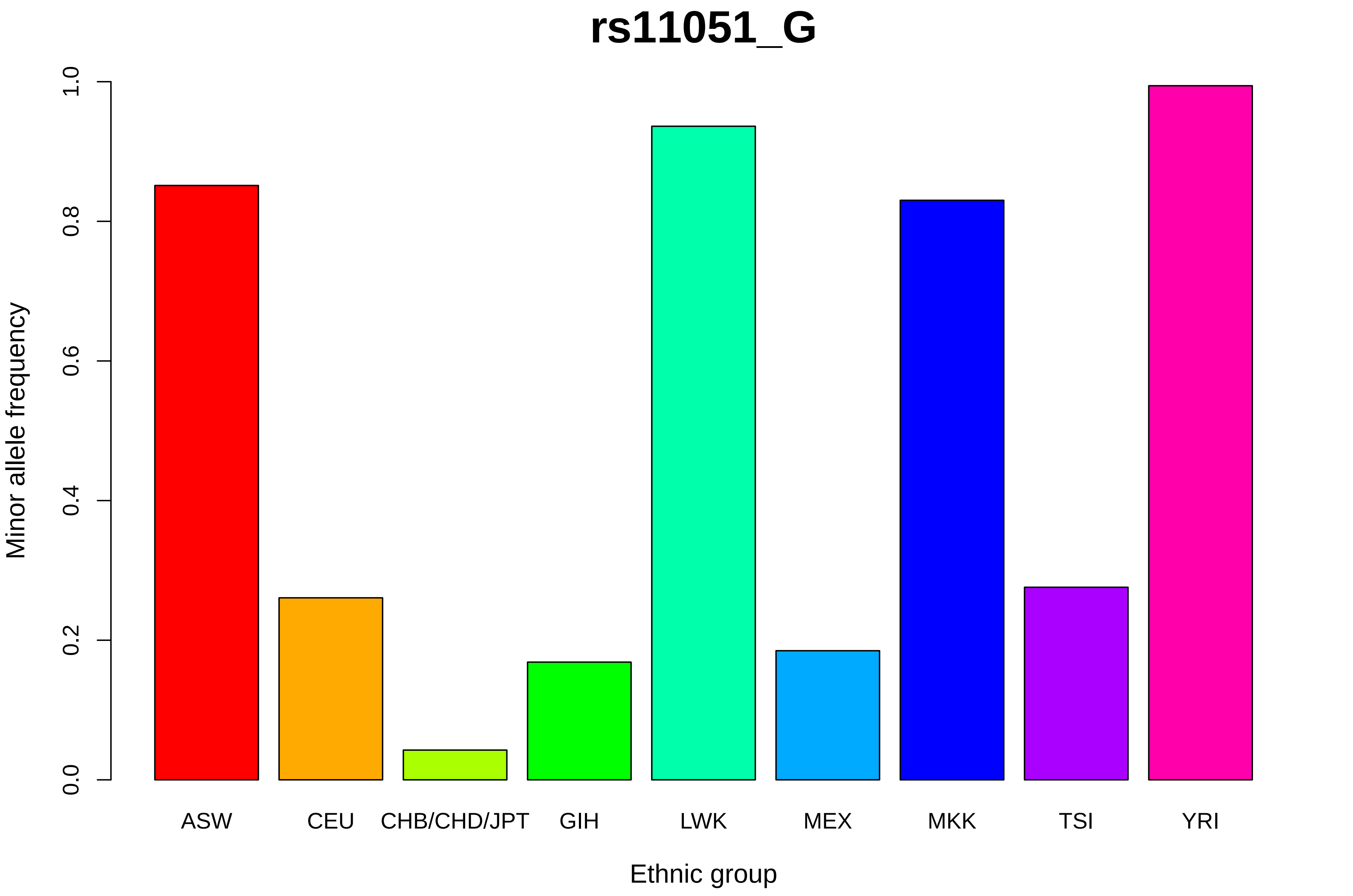
rs11051_G
Minor allele frequency
0.0
0.2
0.4
0.6
0.8
1.0
ASW
CEU
CHB/CHD/JPT
GIH
LWK
MEX
MKK
TSI
YRI
Ethnic group

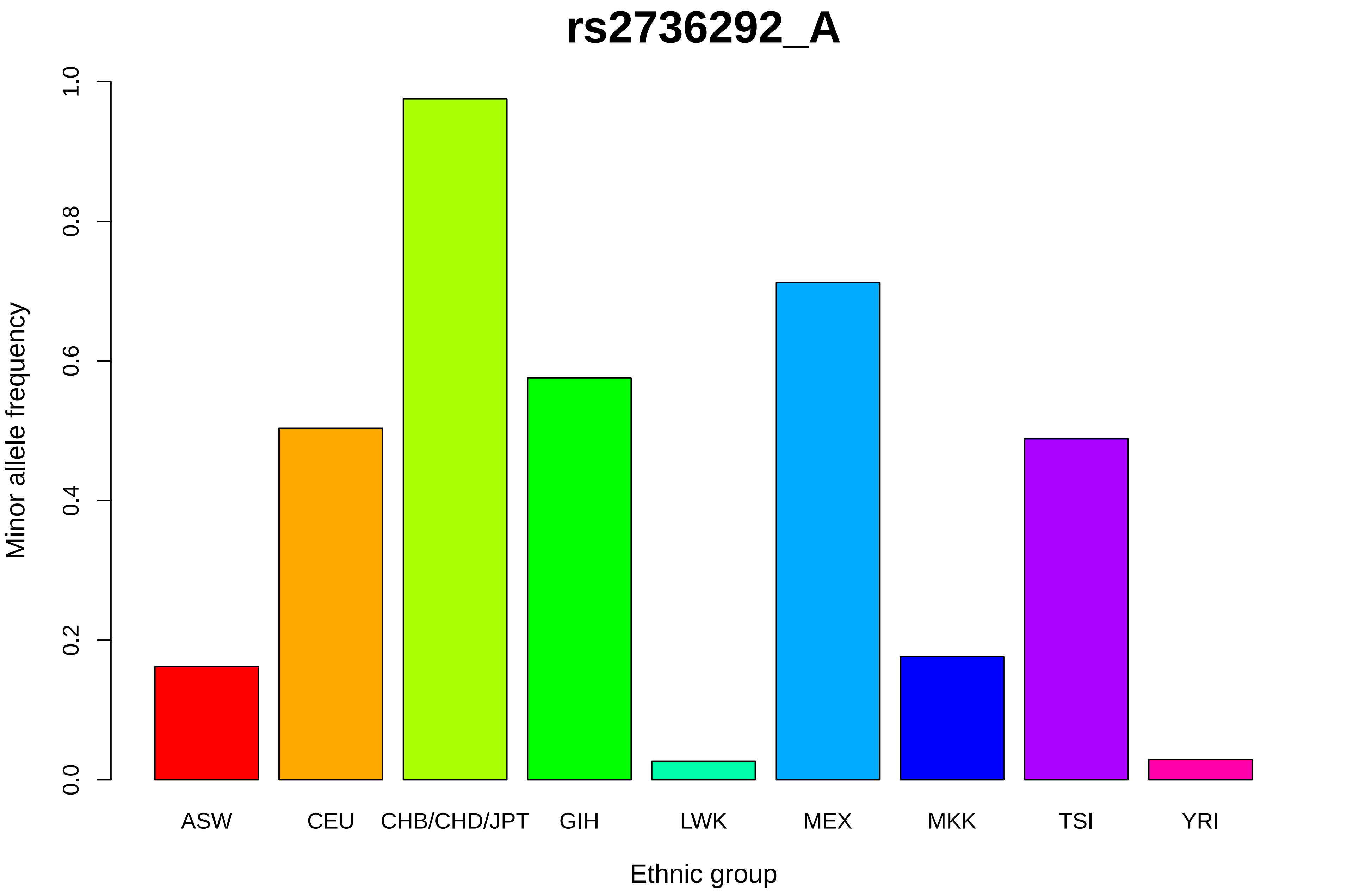

rs2736292_A
Minor allele frequency
0.0
0.2
0.4
0.6
0.8
1.0
ASW
CEU
CHB/CHD/JPT
GIH
LWK
MEX
MKK
TSI
YRI
Ethnic group

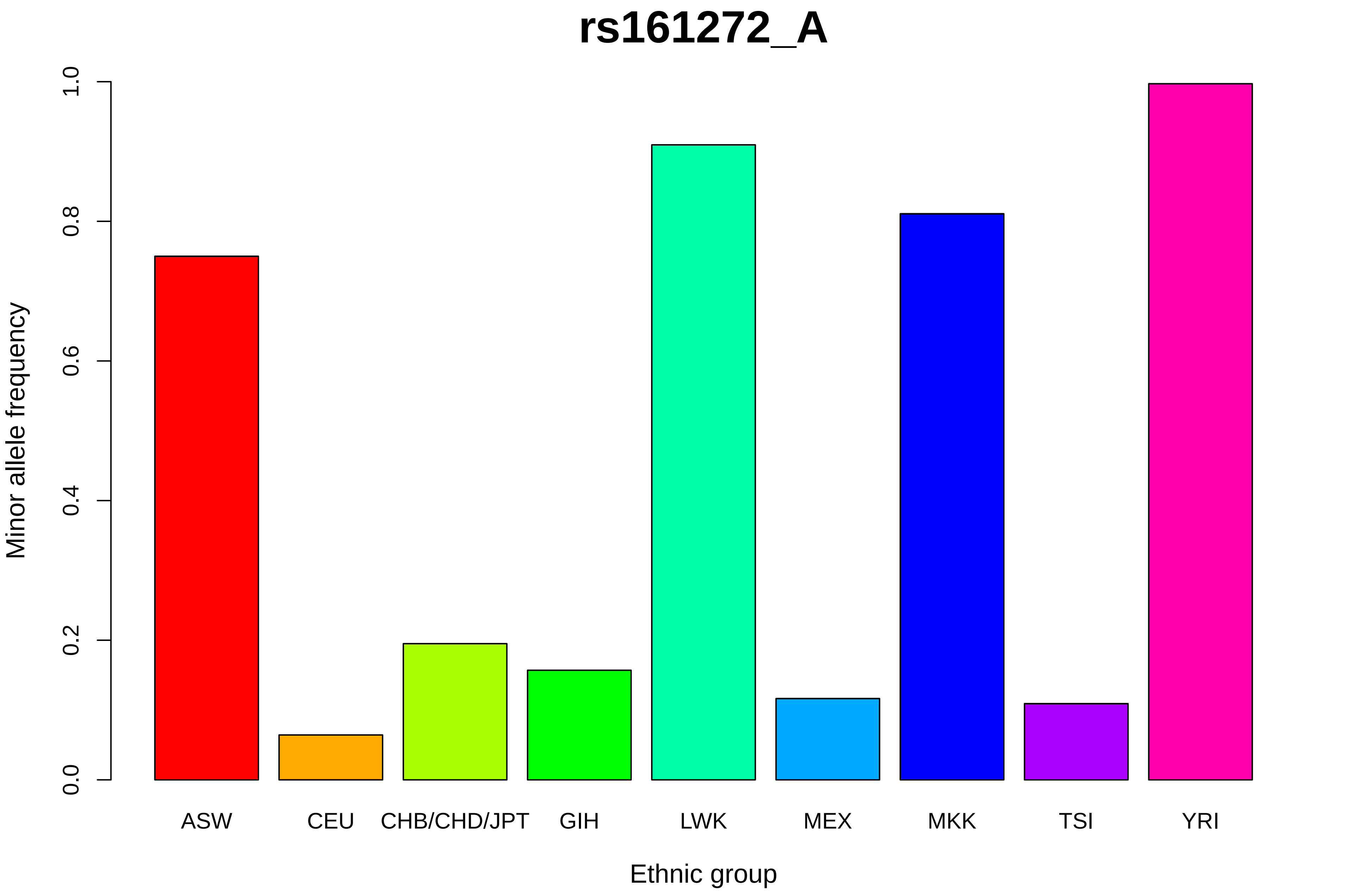
rs161272_A
Minor allele frequency
0.0
0.2
0.4
0.6
0.8
1.0
ASW
CEU
CHB/CHD/JPT
GIH
LWK
MEX
MKK
TSI
YRI
Ethnic group

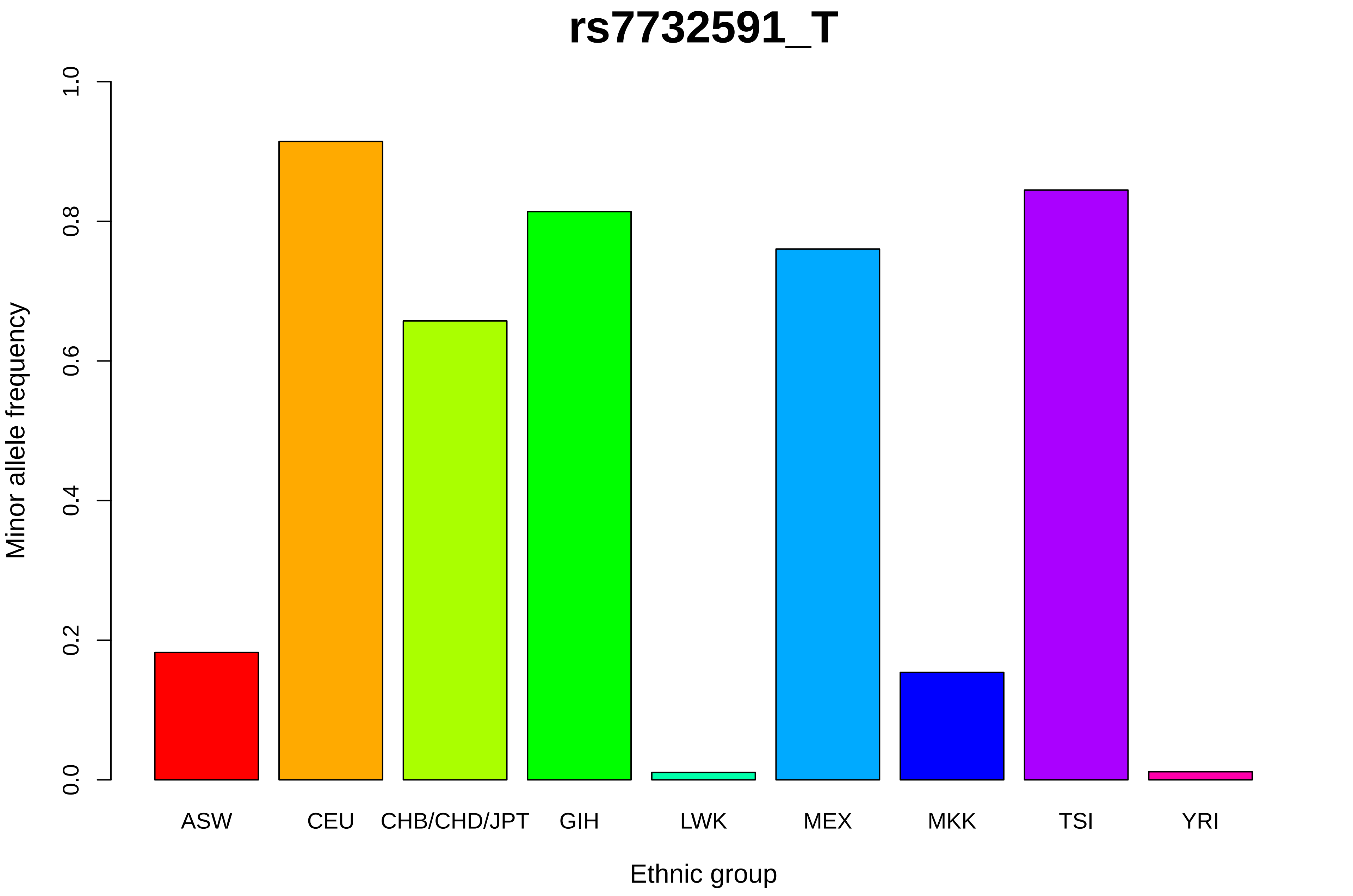

rs7732591_T
Minor allele frequency
0.0
0.2
0.4
0.6
0.8
1.0
ASW
CEU
CHB/CHD/JPT
GIH
LWK
MEX
MKK
TSI
YRI
Ethnic group

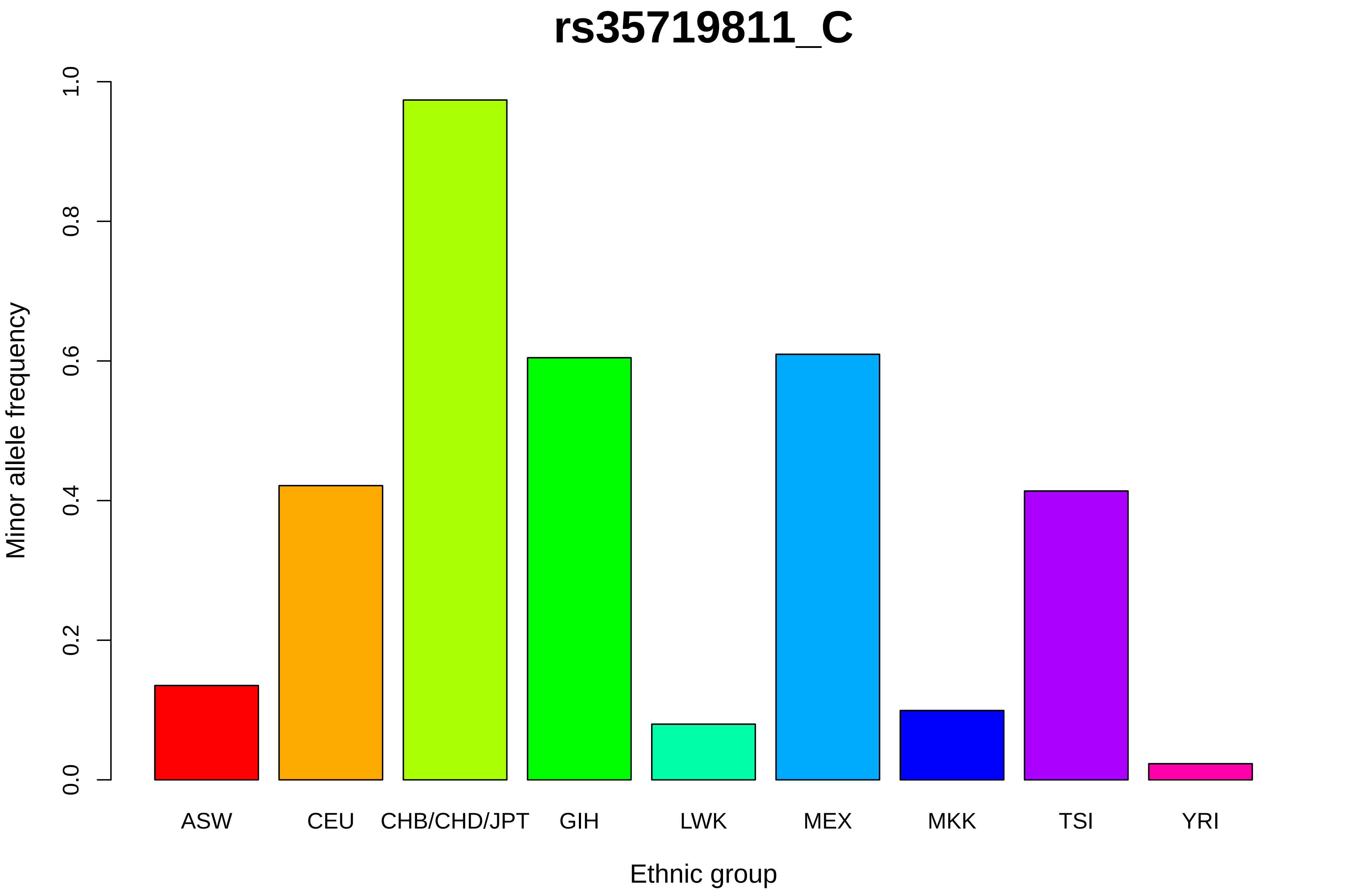
rs35719811_C
Minor allele frequency
0.0
0.2
0.4
0.6
0.8
1.0
ASW
CEU
CHB/CHD/JPT
GIH
LWK
MEX
MKK
TSI
YRI
Ethnic group

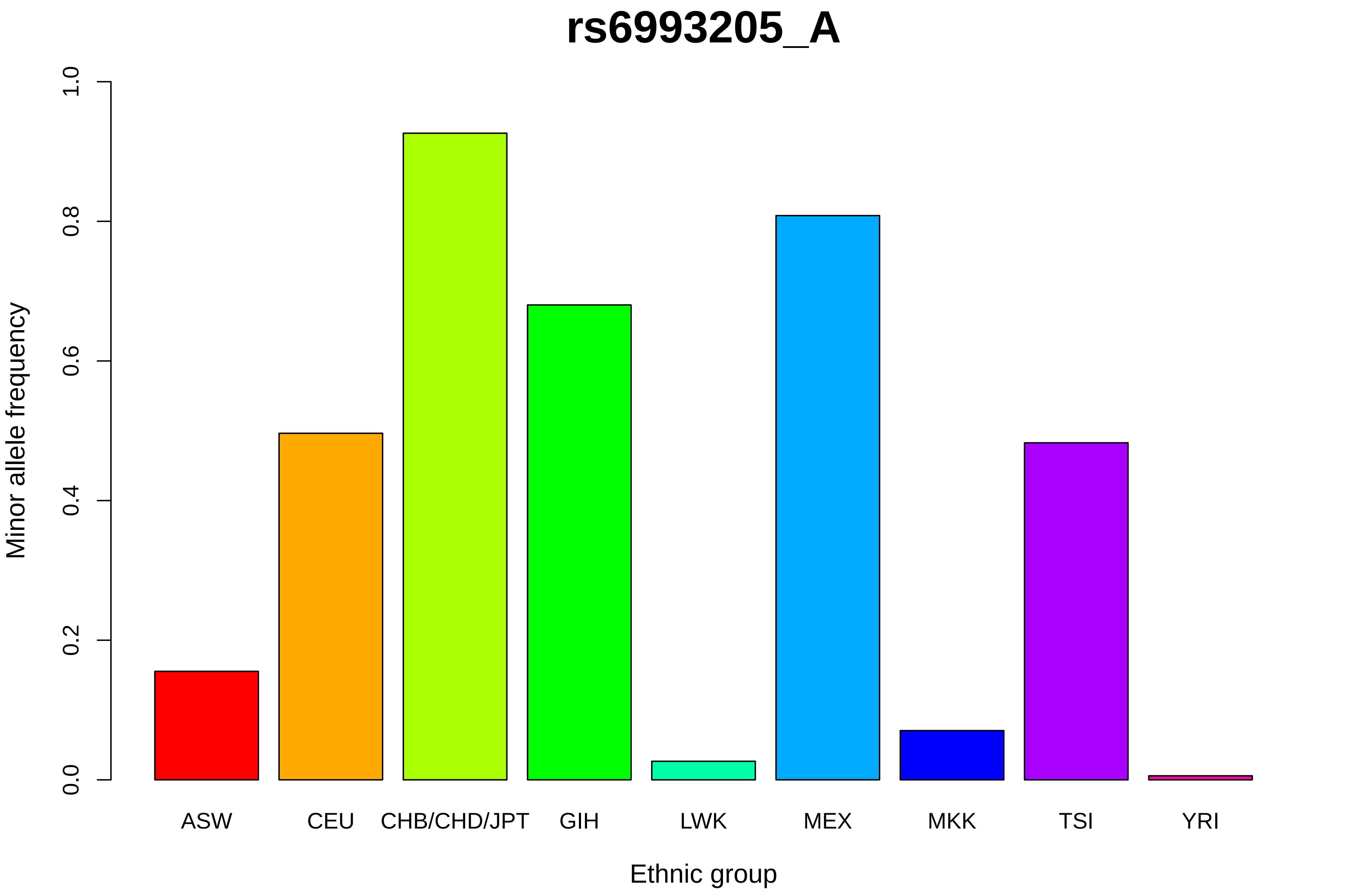
rs6993205_A
Minor allele frequency
0.0
0.2
0.4
0.6
0.8
1.0
ASW
CEU
CHB/CHD/JPT
GIH
LWK
MEX
MKK
TSI
YRI
Ethnic group

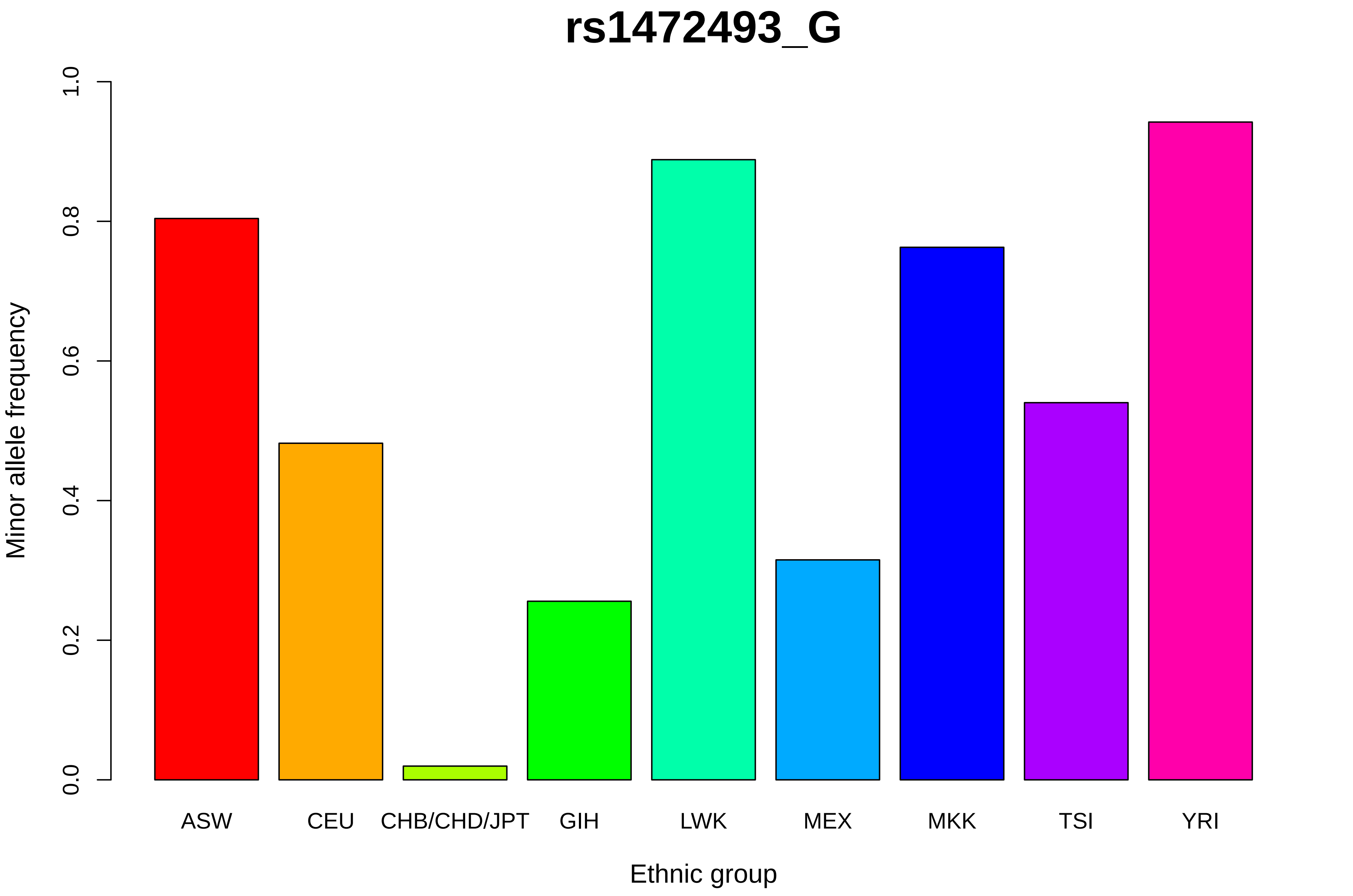
rs1472493_G
Minor allele frequency
0.0
0.2
0.4
0.6
0.8
1.0
ASW
CEU
CHB/CHD/JPT
GIH
LWK
MEX
MKK
TSI
YRI
Ethnic group

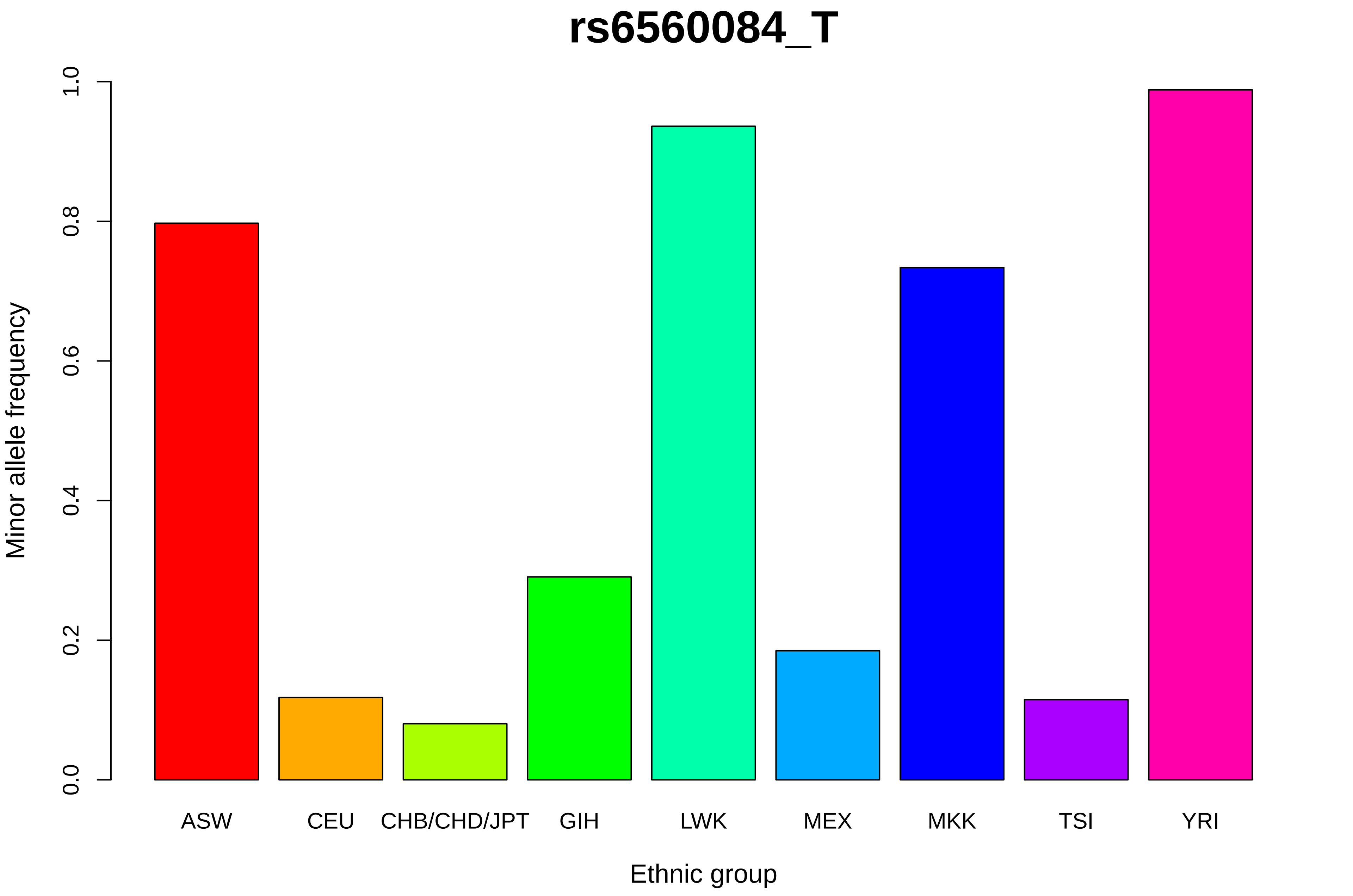
rs6560084_T
Minor allele frequency
0.0
0.2
0.4
0.6
0.8
1.0
ASW
CEU
CHB/CHD/JPT
GIH
LWK
MEX
MKK
TSI
YRI
Ethnic group

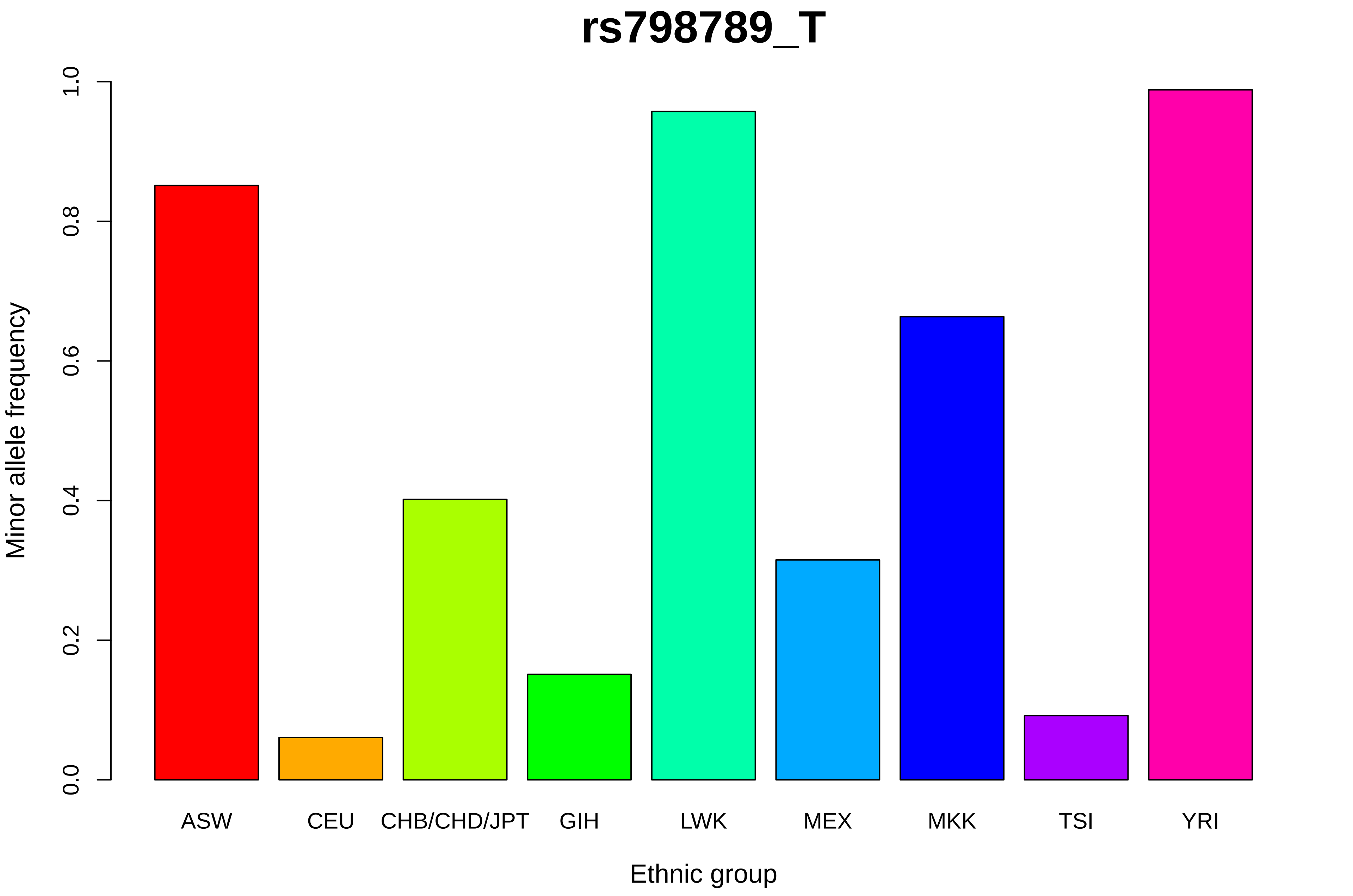

rs798789_T
Minor allele frequency
0.0
0.2
0.4
0.6
0.8
1.0
ASW
CEU
CHB/CHD/JPT
GIH
LWK
MEX
MKK
TSI
YRI
Ethnic group

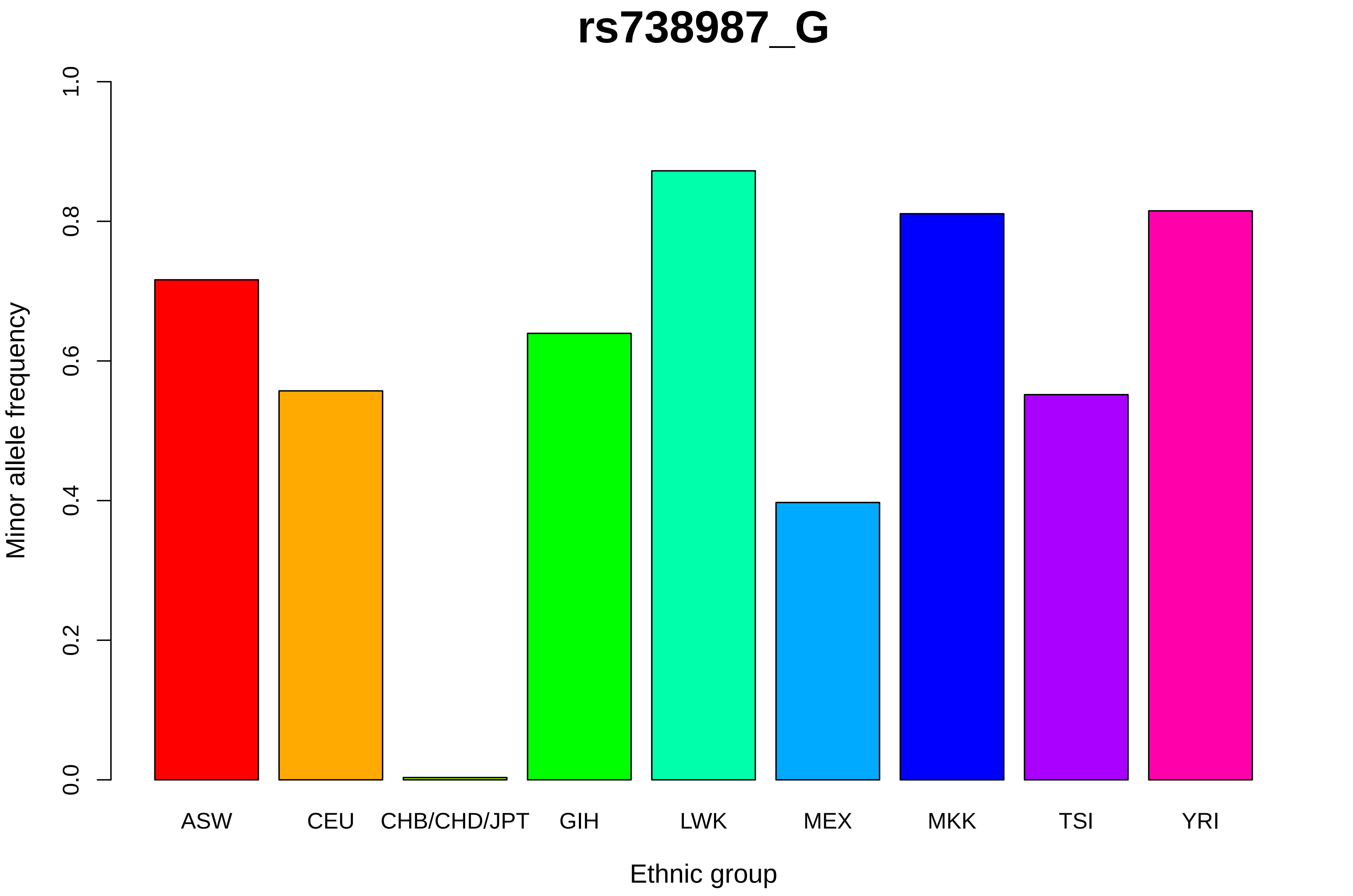

rs738987_G
Minor allele frequency
0.0
0.2
0.4
0.6
0.8
1.0
ASW
CEU
CHB/CHD/JPT
GIH
LWK
MEX
MKK
TSI
YRI
Ethnic group

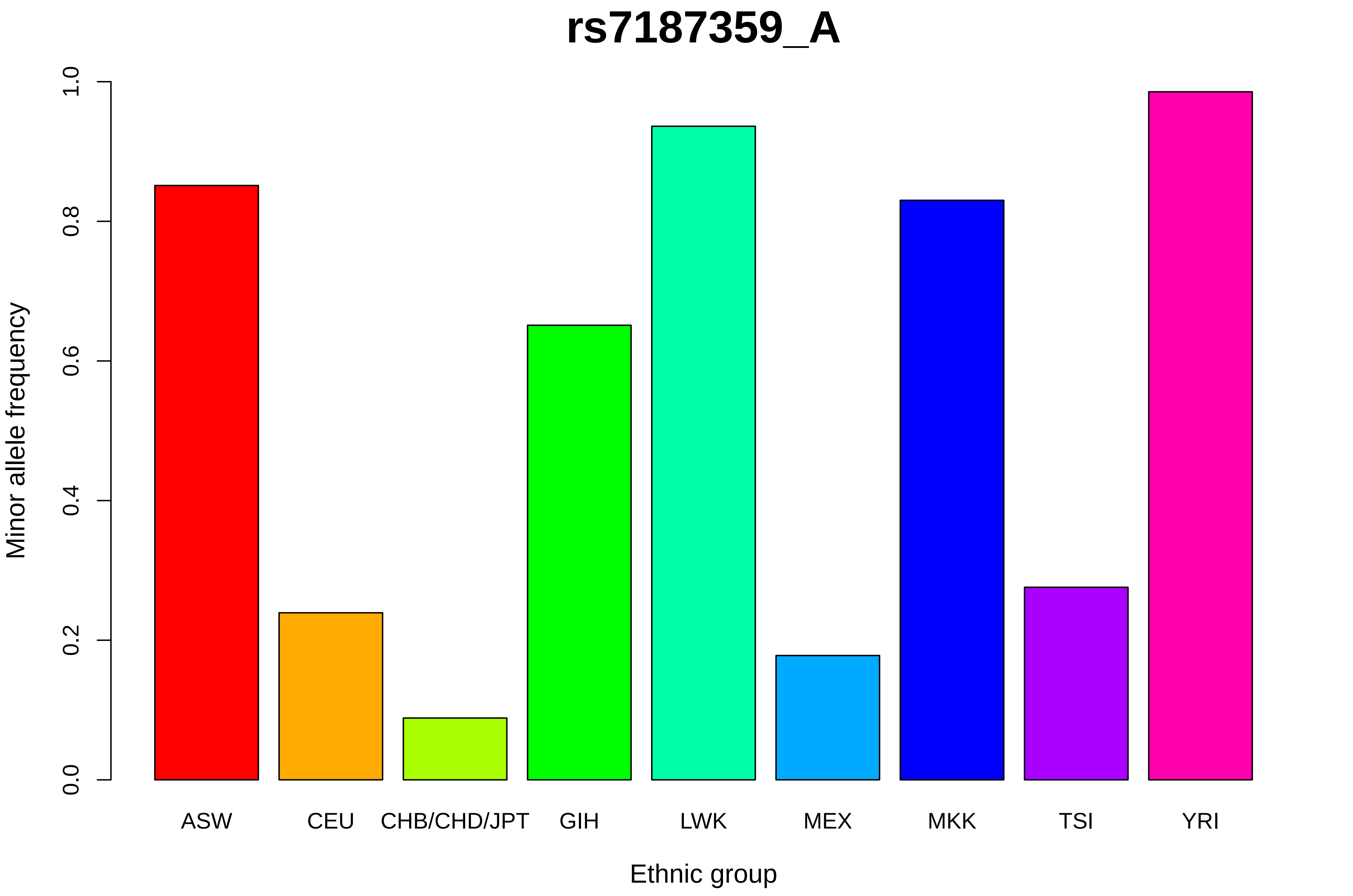
rs7187359_A
Minor allele frequency
0.0
0.2
0.4
0.6
0.8
1.0
ASW
CEU
CHB/CHD/JPT
GIH
LWK
MEX
MKK
TSI
YRI
Ethnic group

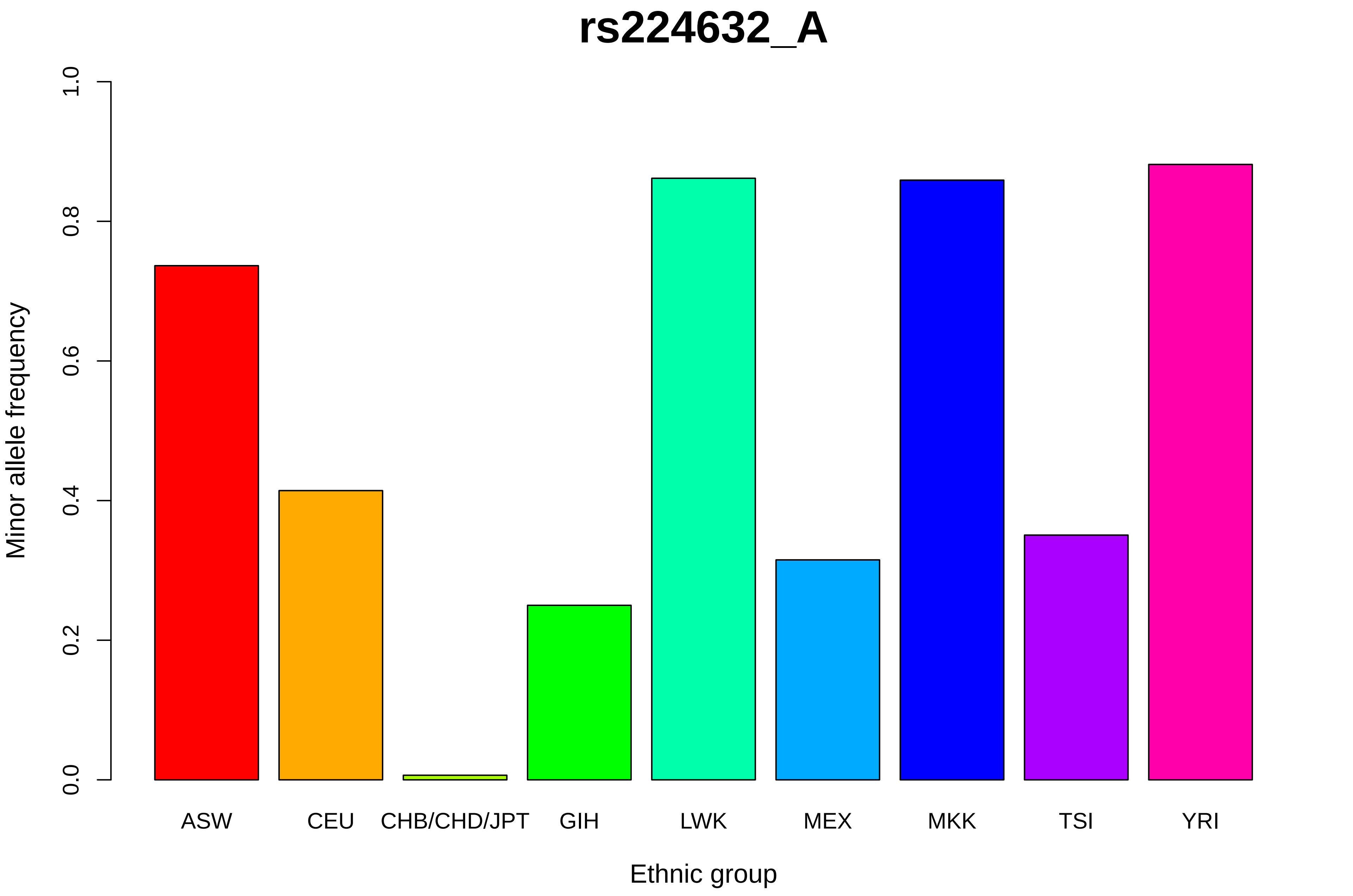
rs224632_A
Minor allele frequency
0.0
0.2
0.4
0.6
0.8
1.0
ASW
CEU
CHB/CHD/JPT
GIH
LWK
MEX
MKK
TSI
YRI
Ethnic group

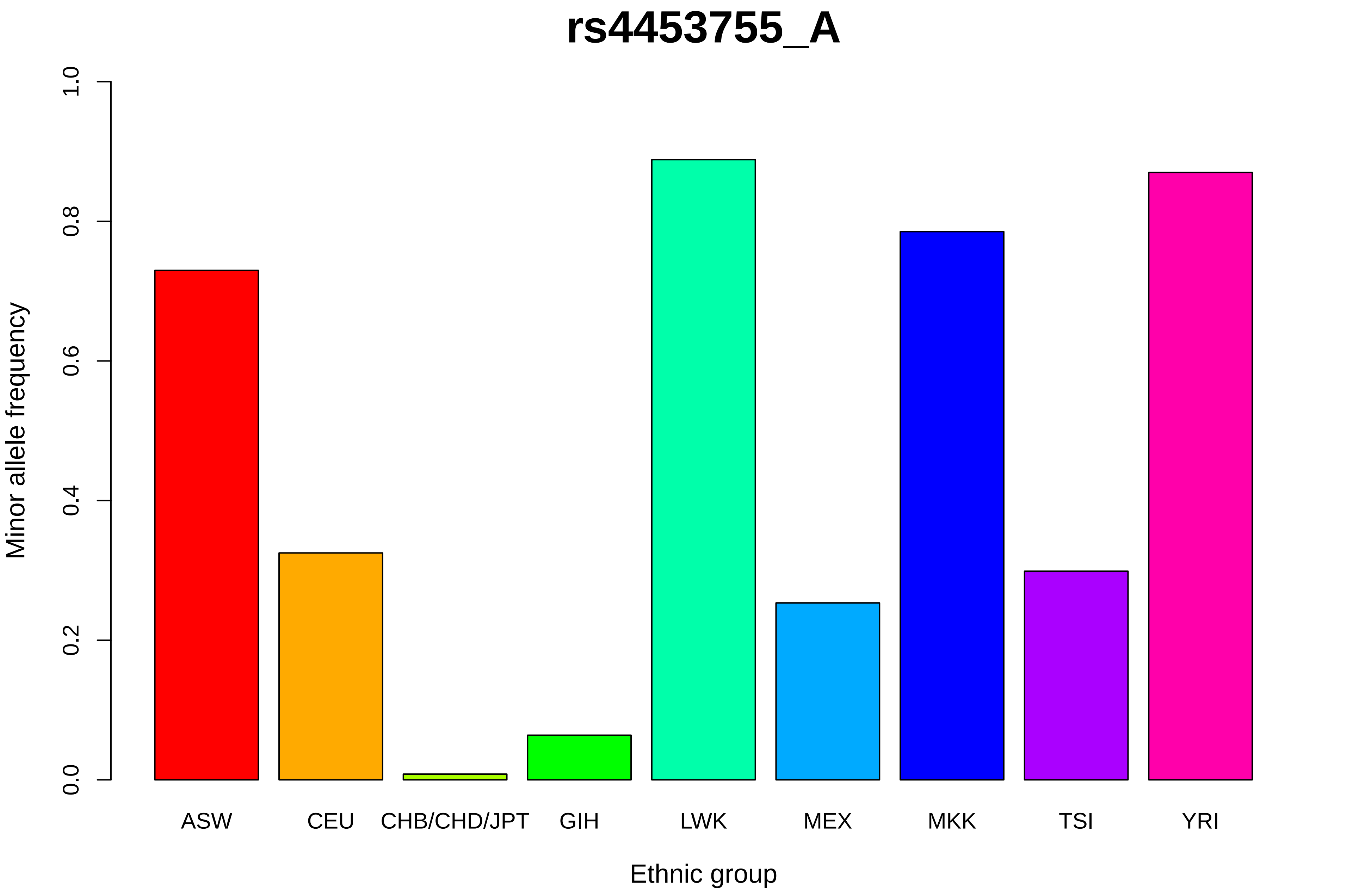
rs4453755_A
Minor allele frequency
0.0
0.2
0.4
0.6
0.8
1.0
ASW
CEU
CHB/CHD/JPT
GIH
LWK
MEX
MKK
TSI
YRI
Ethnic group

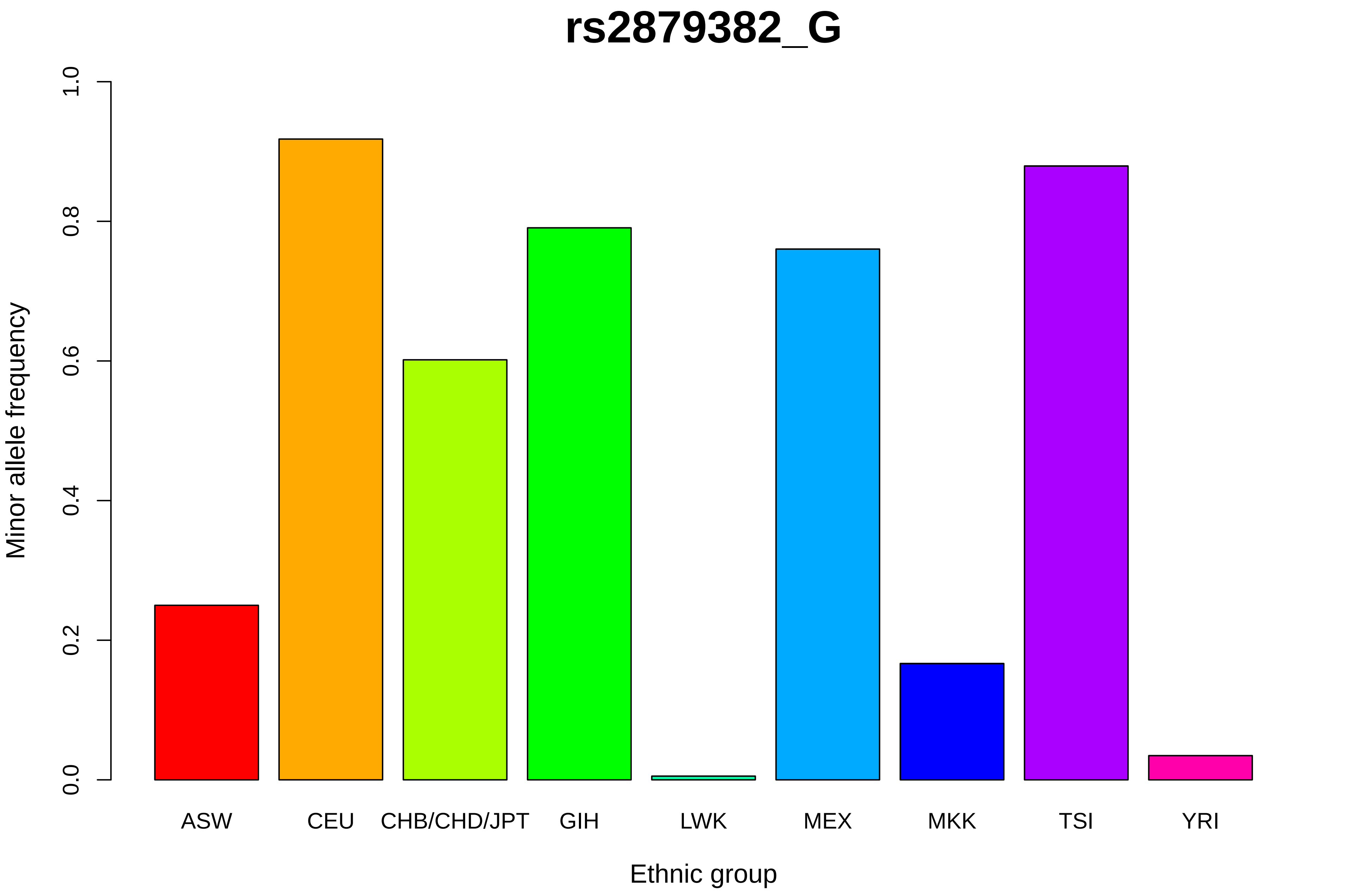
rs2879382_G
Minor allele frequency
0.0
0.2
0.4
0.6
0.8
1.0
ASW
CEU
CHB/CHD/JPT
GIH
LWK
MEX
MKK
TSI
YRI
Ethnic group

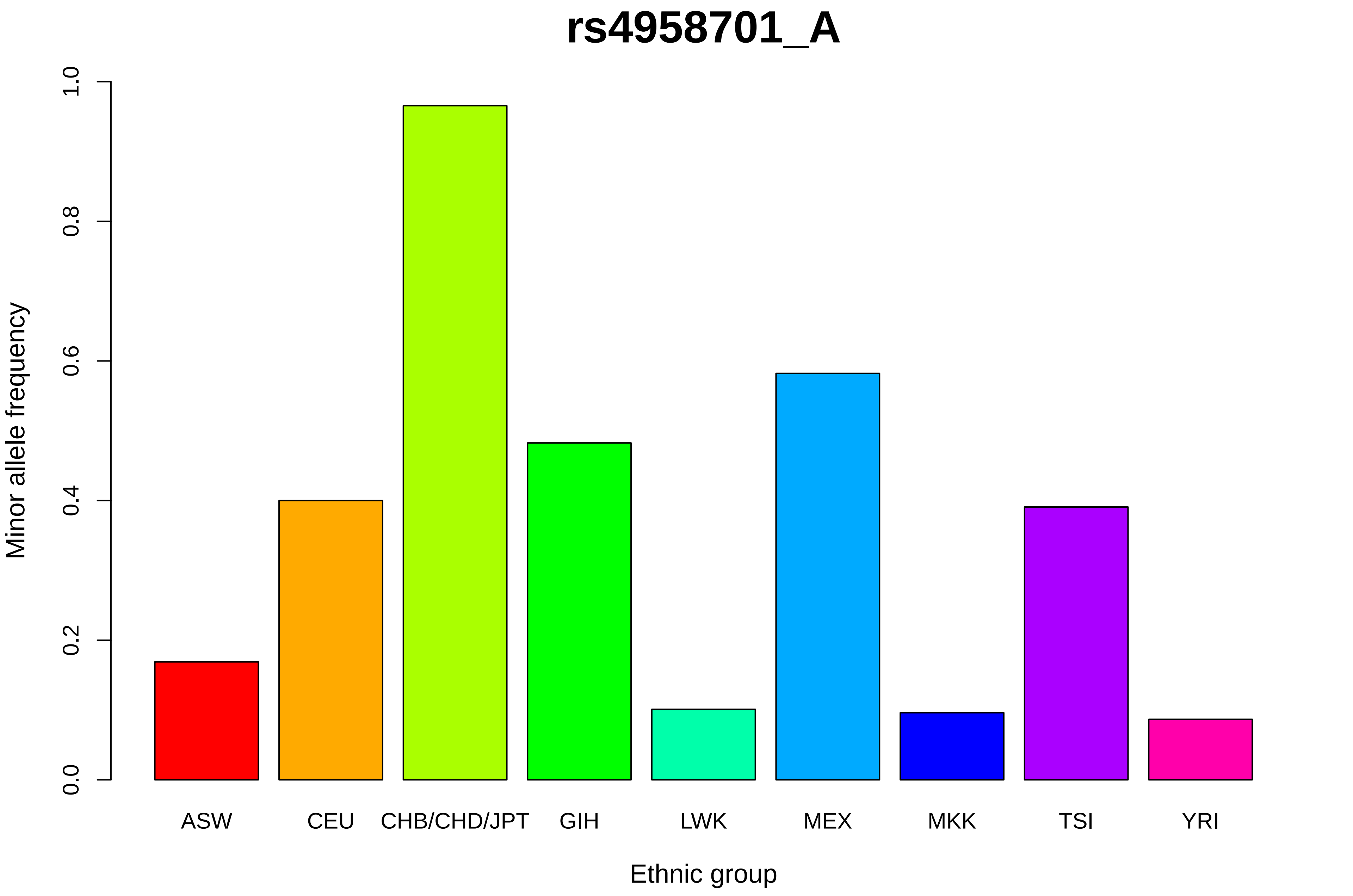
rs4958701_A
Minor allele frequency
0.0
0.2
0.4
0.6
0.8
1.0
ASW
CEU
CHB/CHD/JPT
GIH
LWK
MEX
MKK
TSI
YRI
Ethnic group

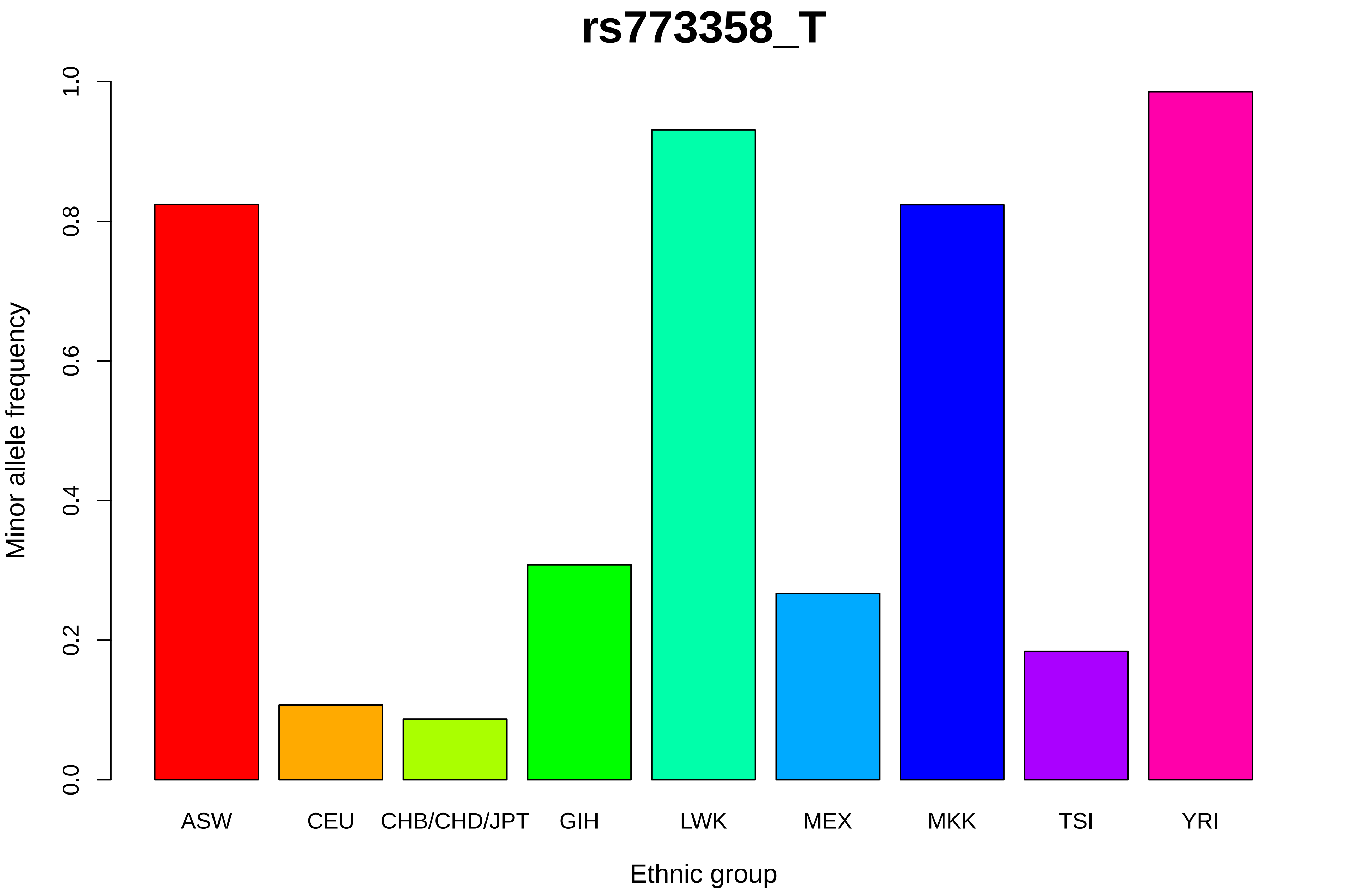
rs773358_T
Minor allele frequency
0.0
0.2
0.4
0.6
0.8
1.0
ASW
CEU
CHB/CHD/JPT
GIH
LWK
MEX
MKK
TSI
YRI
Ethnic group

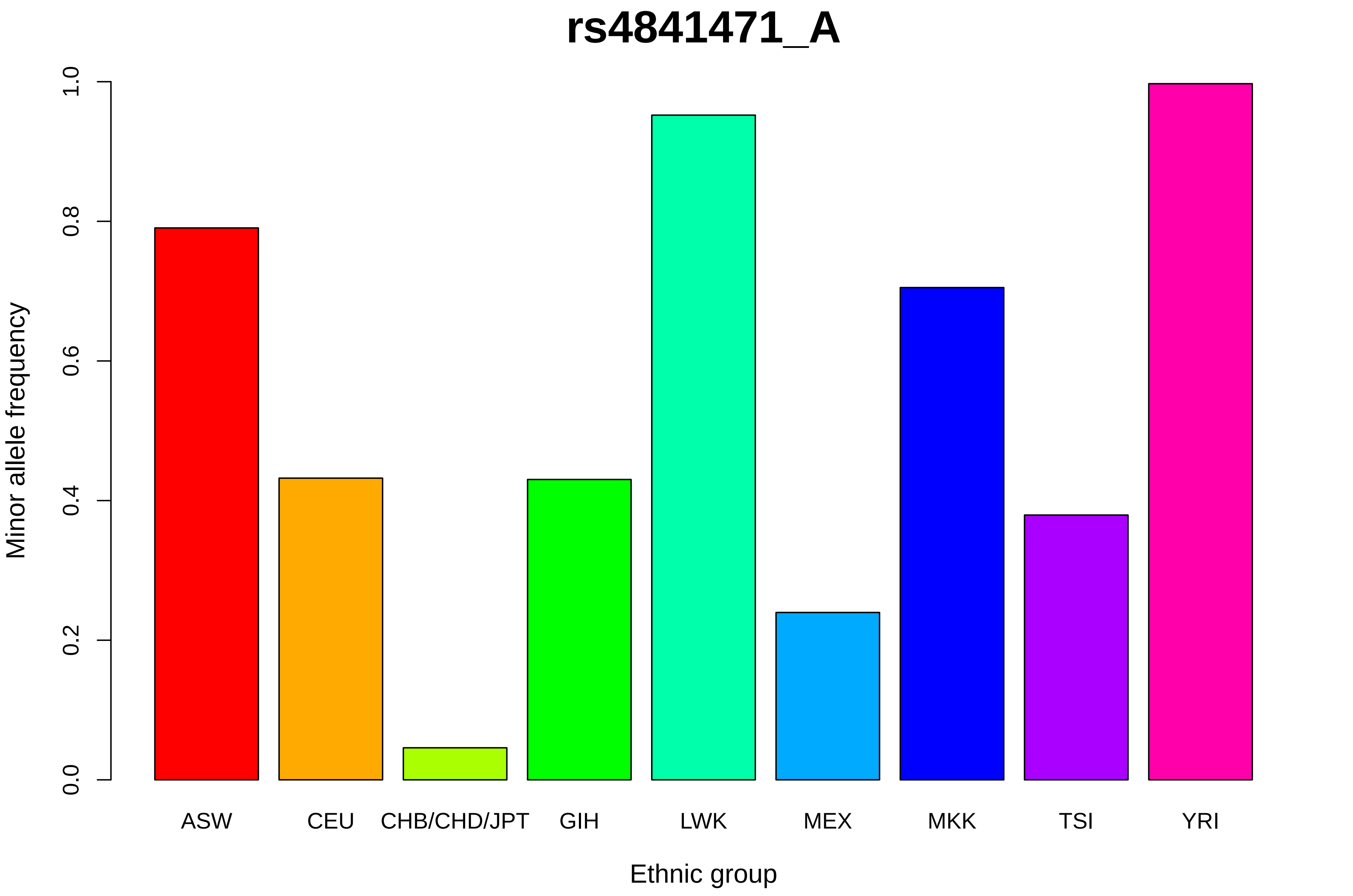
rs4841471_A
Minor allele frequency
0.0
0.2
0.4
0.6
0.8
1.0
ASW
CEU
CHB/CHD/JPT
GIH
LWK
MEX
MKK
TSI
YRI
Ethnic group

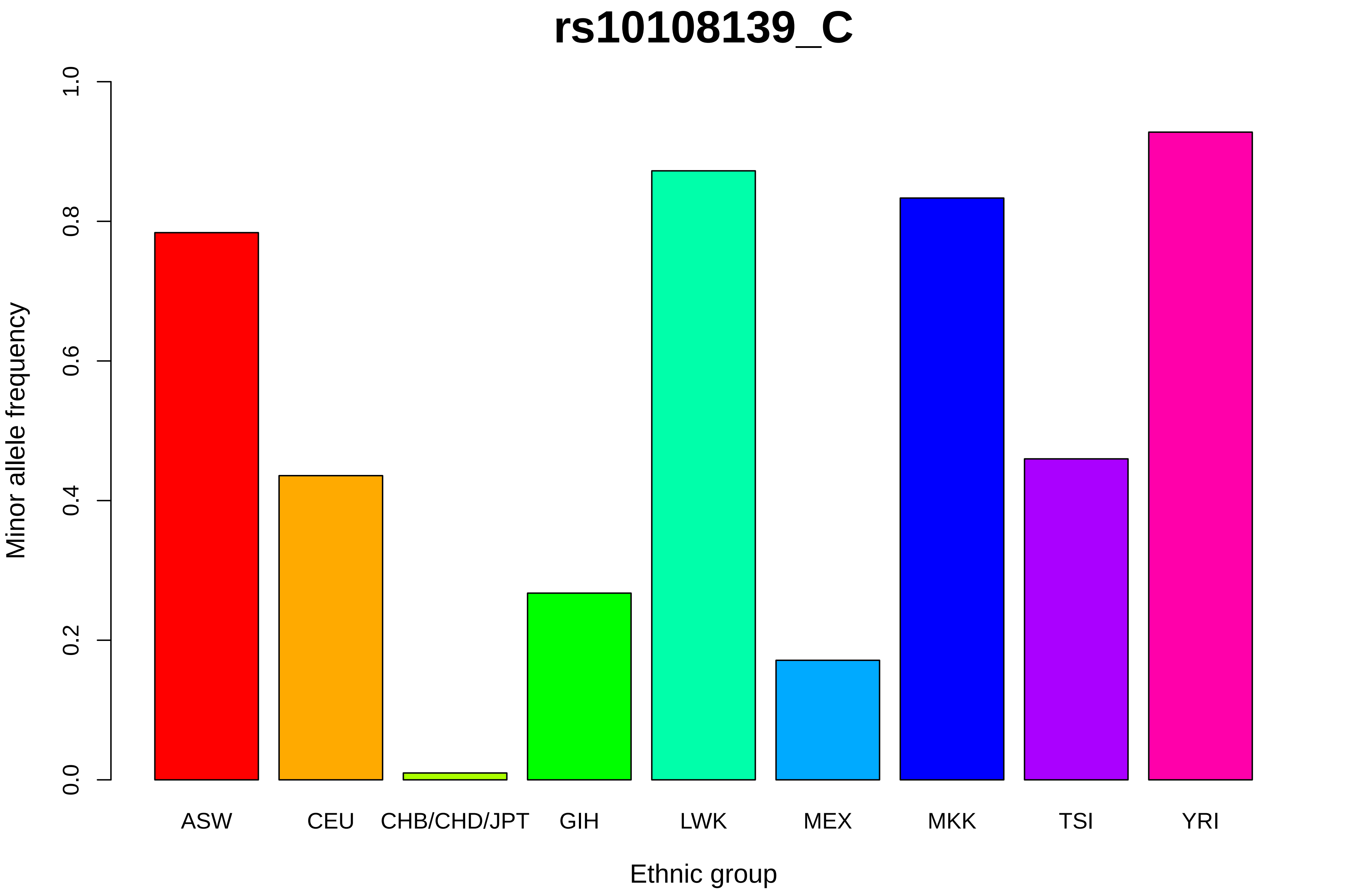
rs10108139_C
Minor allele frequency
0.0
0.2
0.4
0.6
0.8
1.0
ASW
CEU
CHB/CHD/JPT
GIH
LWK
MEX
MKK
TSI
YRI
Ethnic group

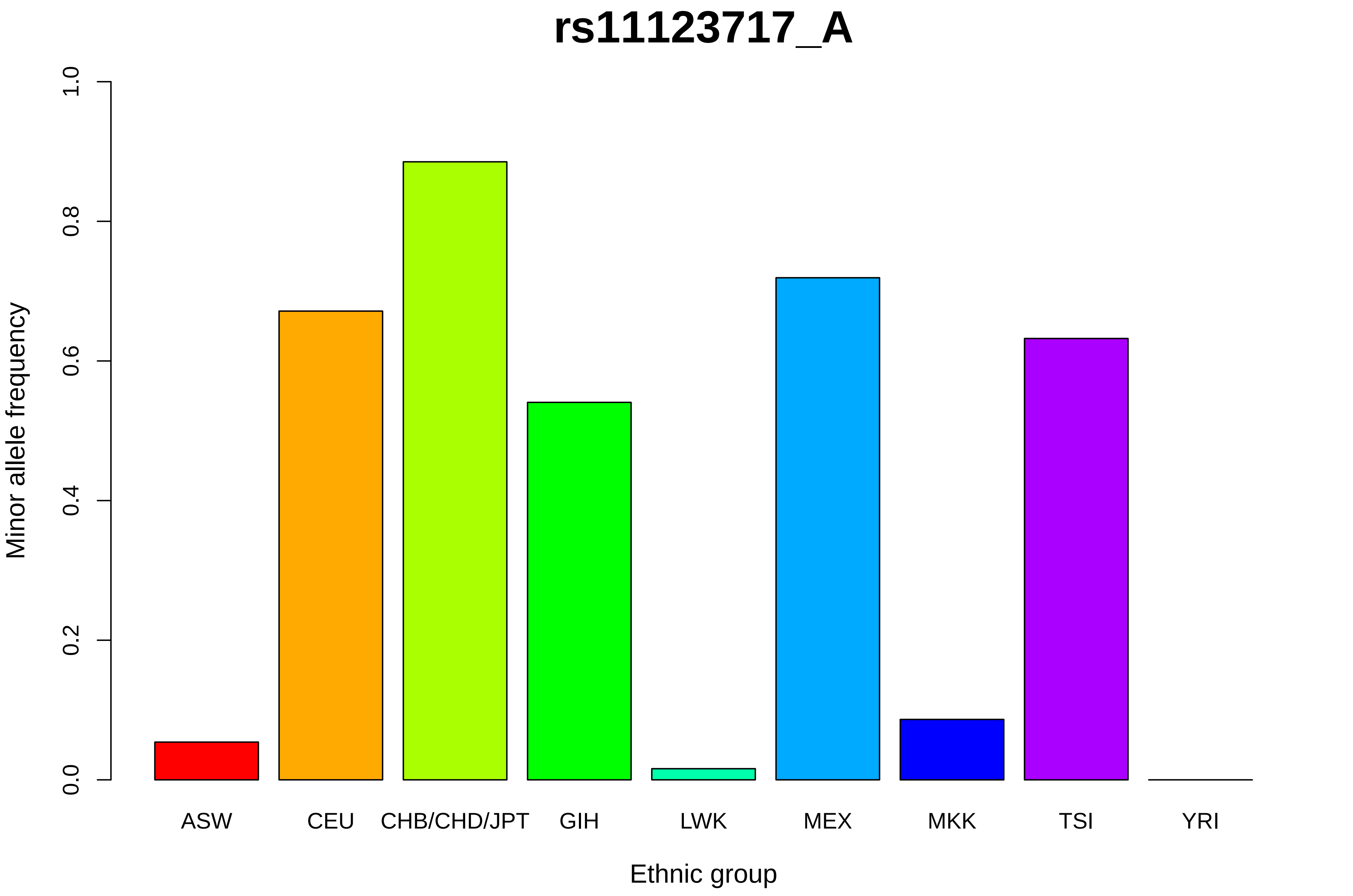
rs11123717_A
Minor allele frequency
0.0
0.2
0.4
0.6
0.8
1.0
ASW
CEU
CHB/CHD/JPT
GIH
LWK
MEX
MKK
TSI
YRI
Ethnic group

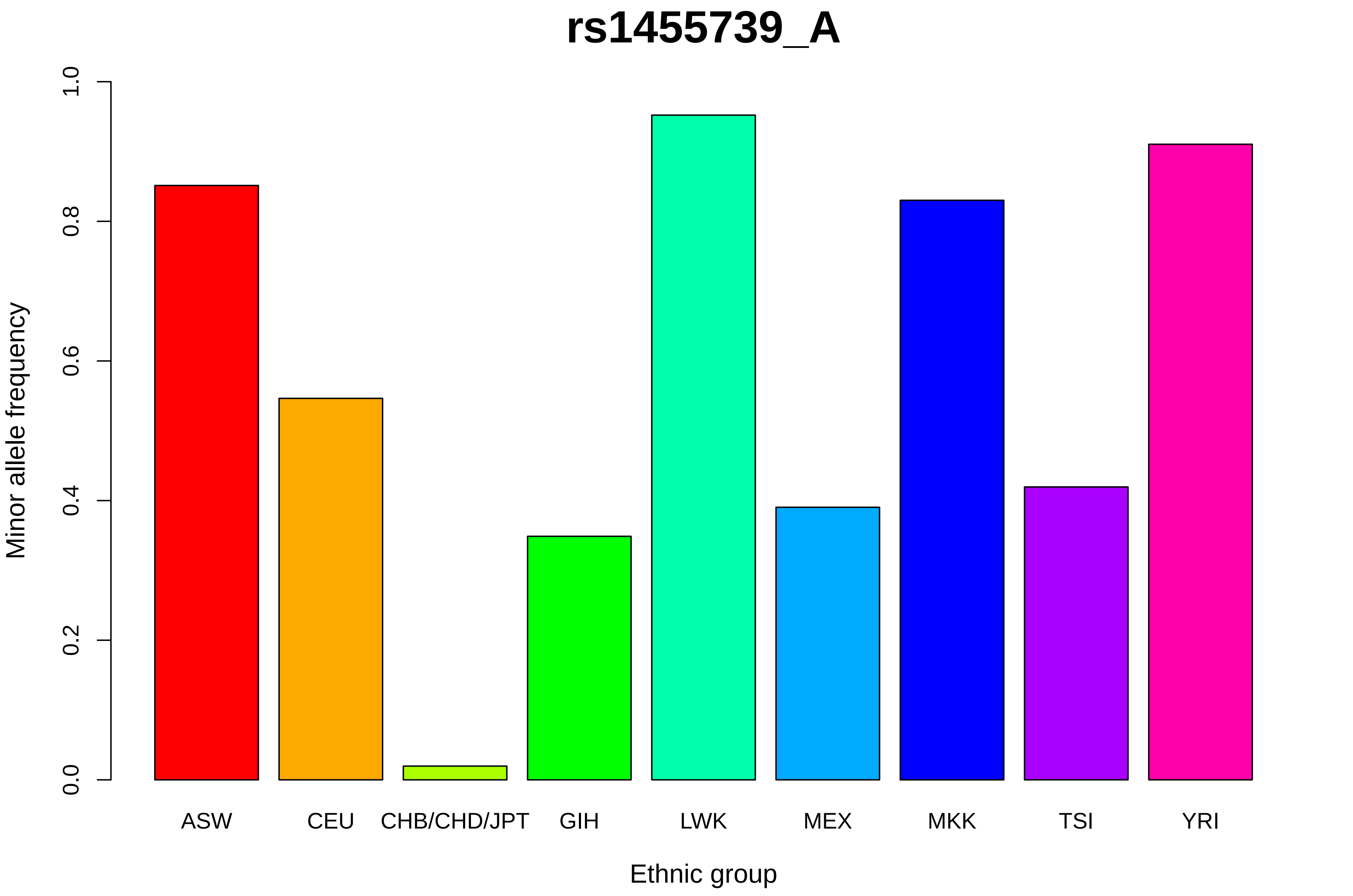
rs1455739_A
Minor allele frequency
0.0
0.2
0.4
0.6
0.8
1.0
ASW
CEU
CHB/CHD/JPT
GIH
LWK
MEX
MKK
TSI
YRI
Ethnic group

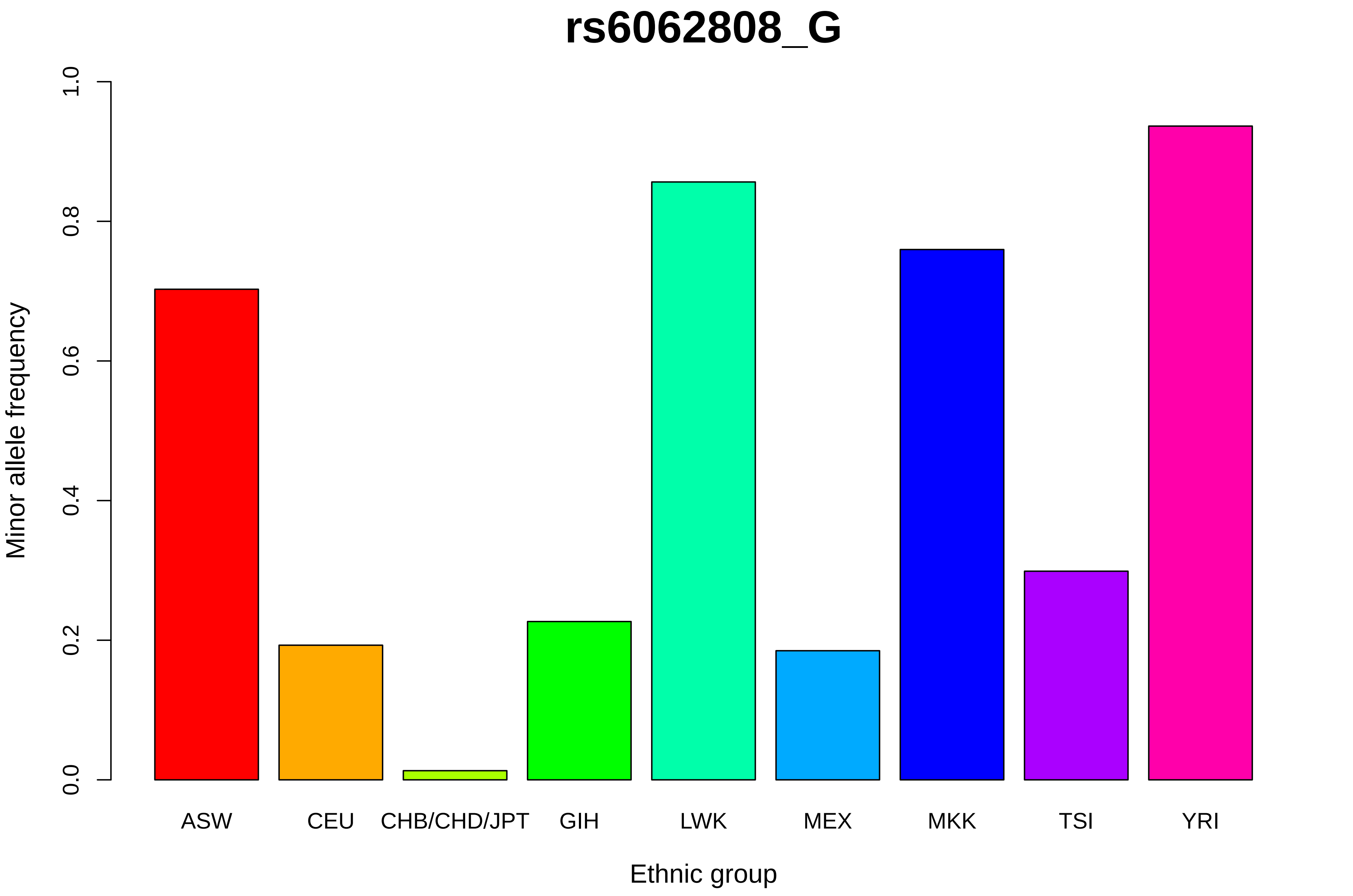
rs6062808_G
Minor allele frequency
0.0
0.2
0.4
0.6
0.8
1.0
ASW
CEU
CHB/CHD/JPT
GIH
LWK
MEX
MKK
TSI
YRI
Ethnic group

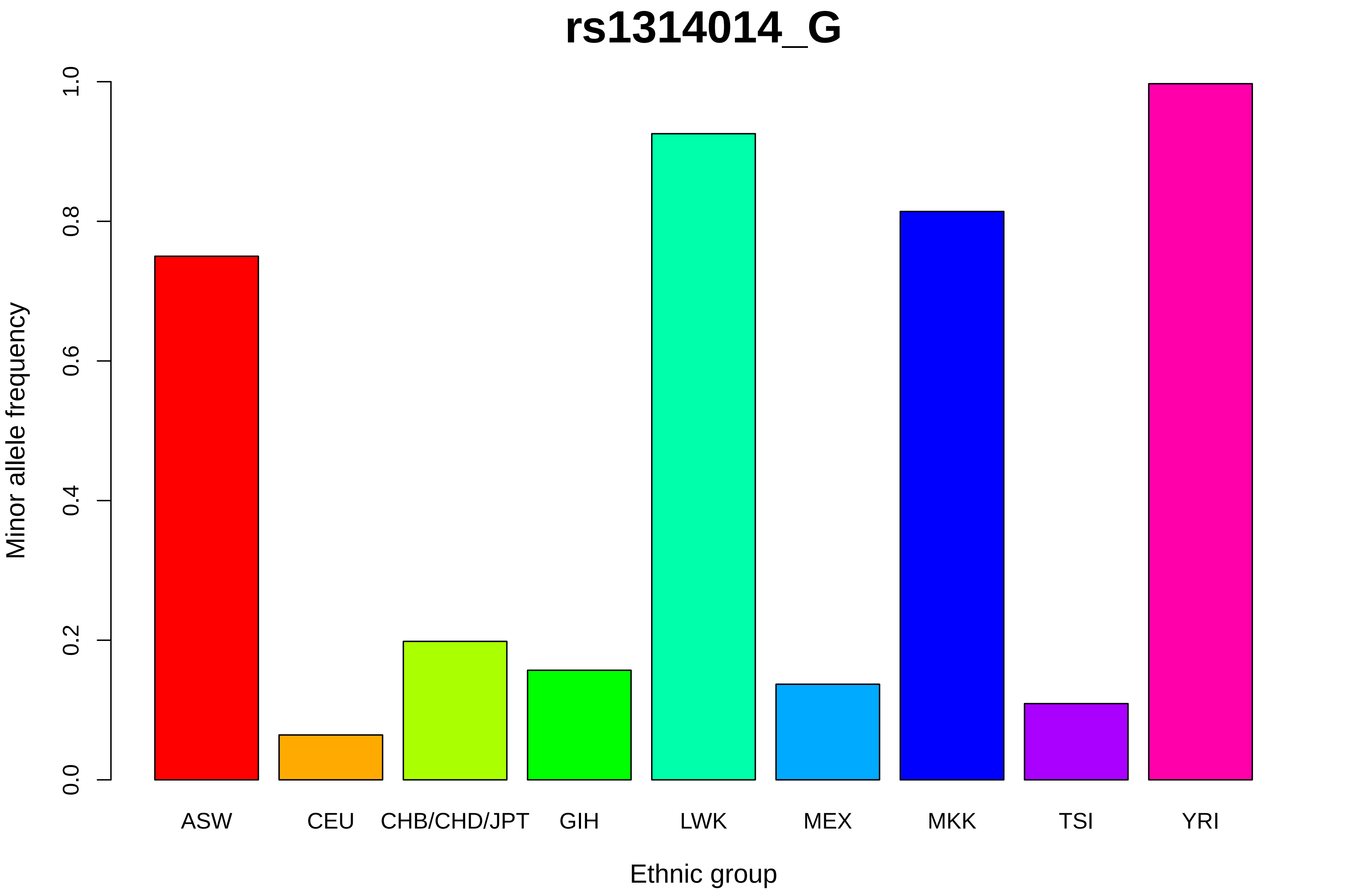
rs1314014_G
Minor allele frequency
0.0
0.2
0.4
0.6
0.8
1.0
ASW
CEU
CHB/CHD/JPT
GIH
LWK
MEX
MKK
TSI
YRI
Ethnic group

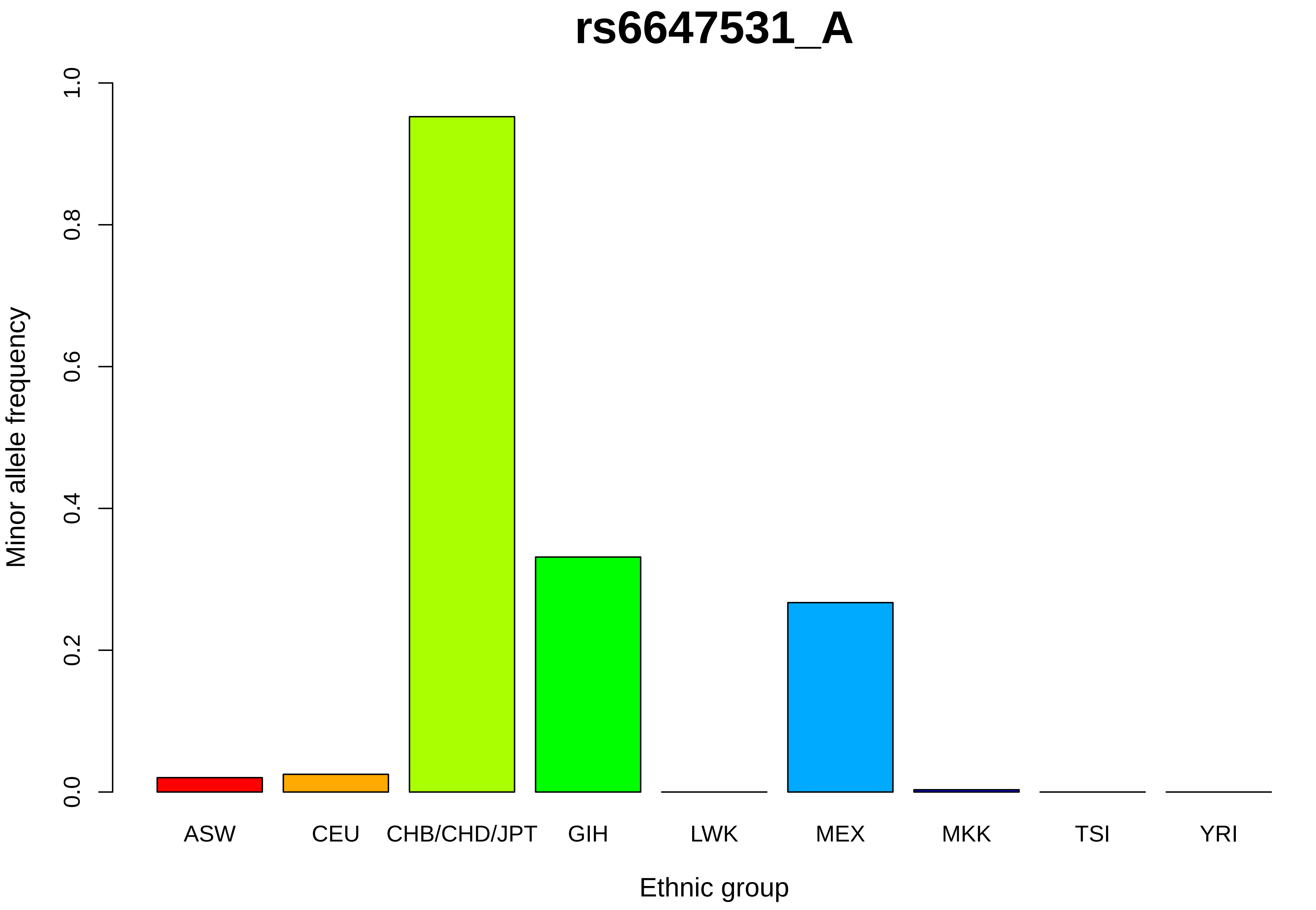
rs6647531_A
Minor allele frequency
0.0
0.2
0.4
0.6
0.8
1.0
ASW
CEU
CHB/CHD/JPT
GIH
LWK
MEX
MKK
TSI
YRI
Ethnic group

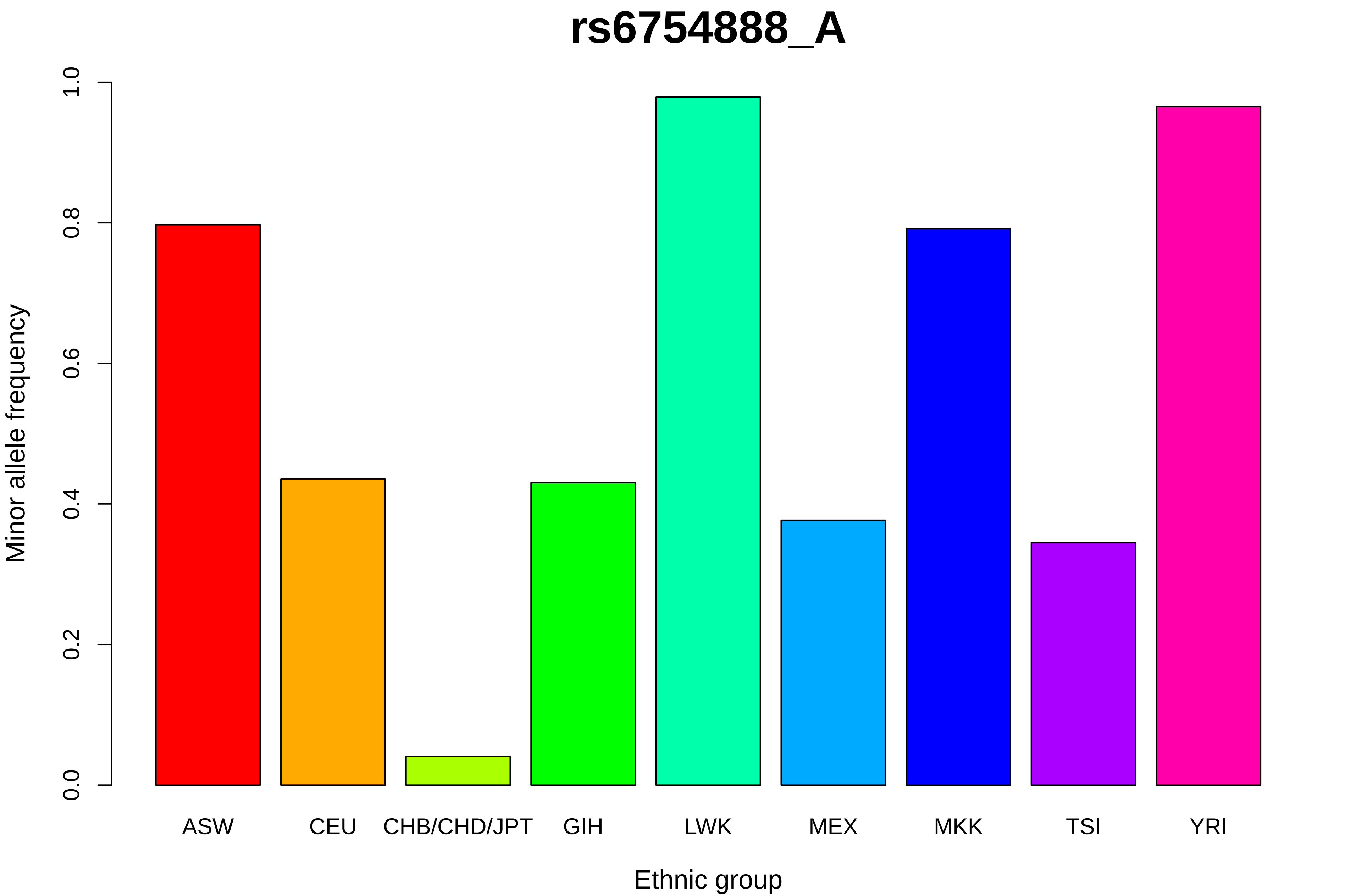
rs6754888_A
Minor allele frequency
0.0
0.2
0.4
0.6
0.8
1.0
ASW
CEU
CHB/CHD/JPT
GIH
LWK
MEX
MKK
TSI
YRI
Ethnic group

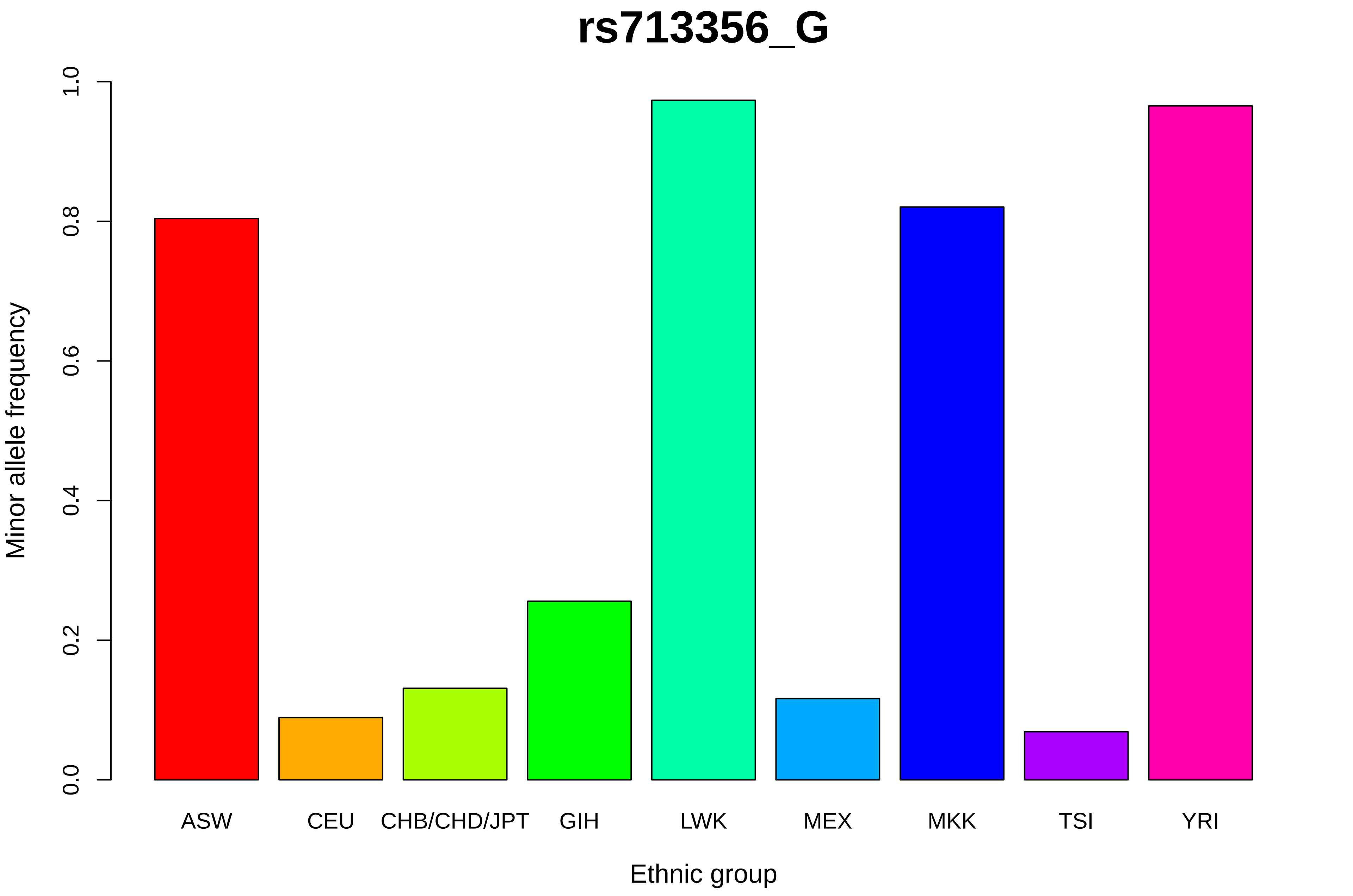
rs713356_G
Minor allele frequency
0.0
0.2
0.4
0.6
0.8
1.0
ASW
CEU
CHB/CHD/JPT
GIH
LWK
MEX
MKK
TSI
YRI
Ethnic group

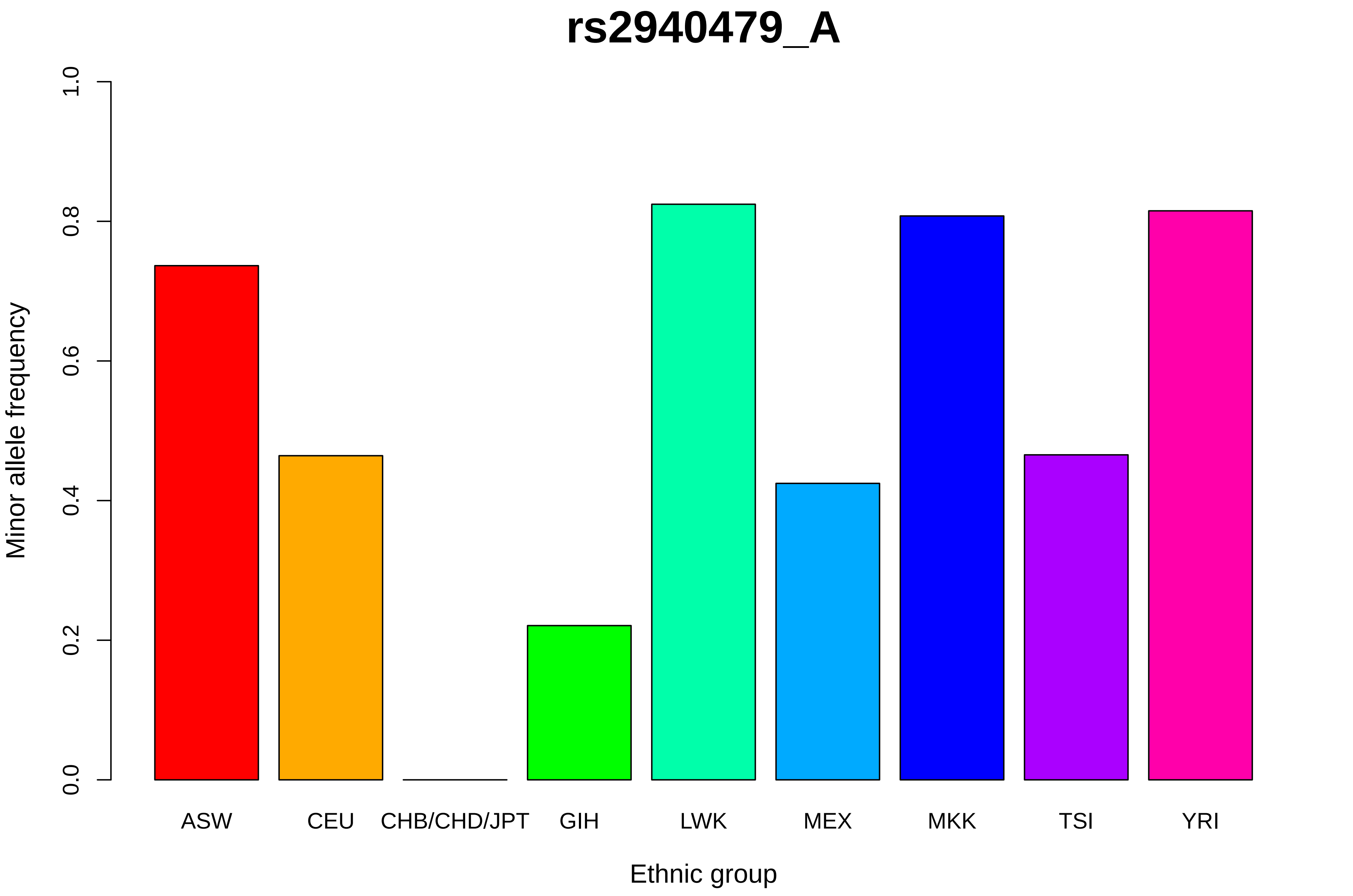
rs2940479_A
Minor allele frequency
0.0
0.2
0.4
0.6
0.8
1.0
ASW
CEU
CHB/CHD/JPT
GIH
LWK
MEX
MKK
TSI
YRI
Ethnic group

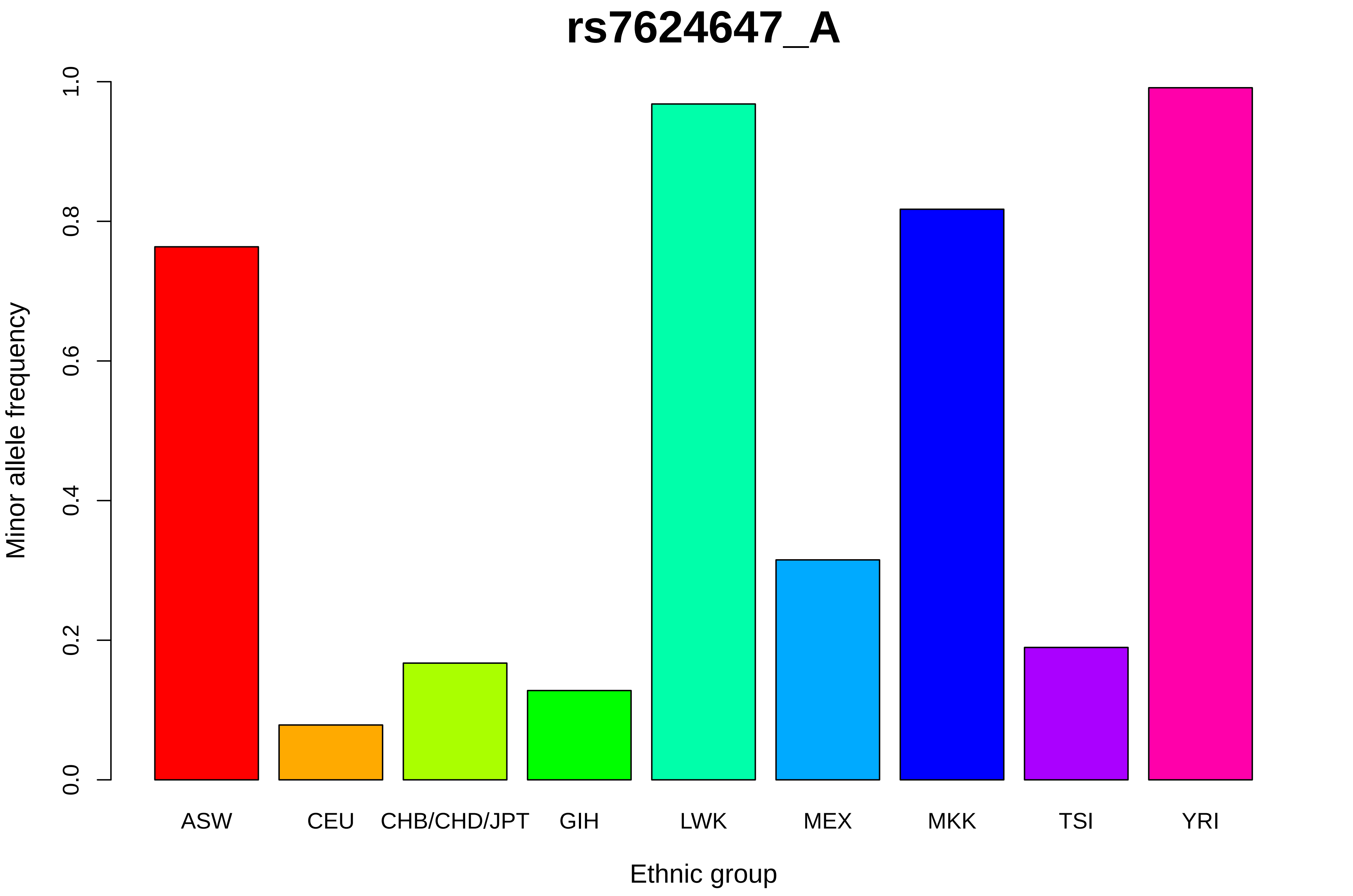
rs7624647_A
Minor allele frequency
0.0
0.2
0.4
0.6
0.8
1.0
ASW
CEU
CHB/CHD/JPT
GIH
LWK
MEX
MKK
TSI
YRI
Ethnic group

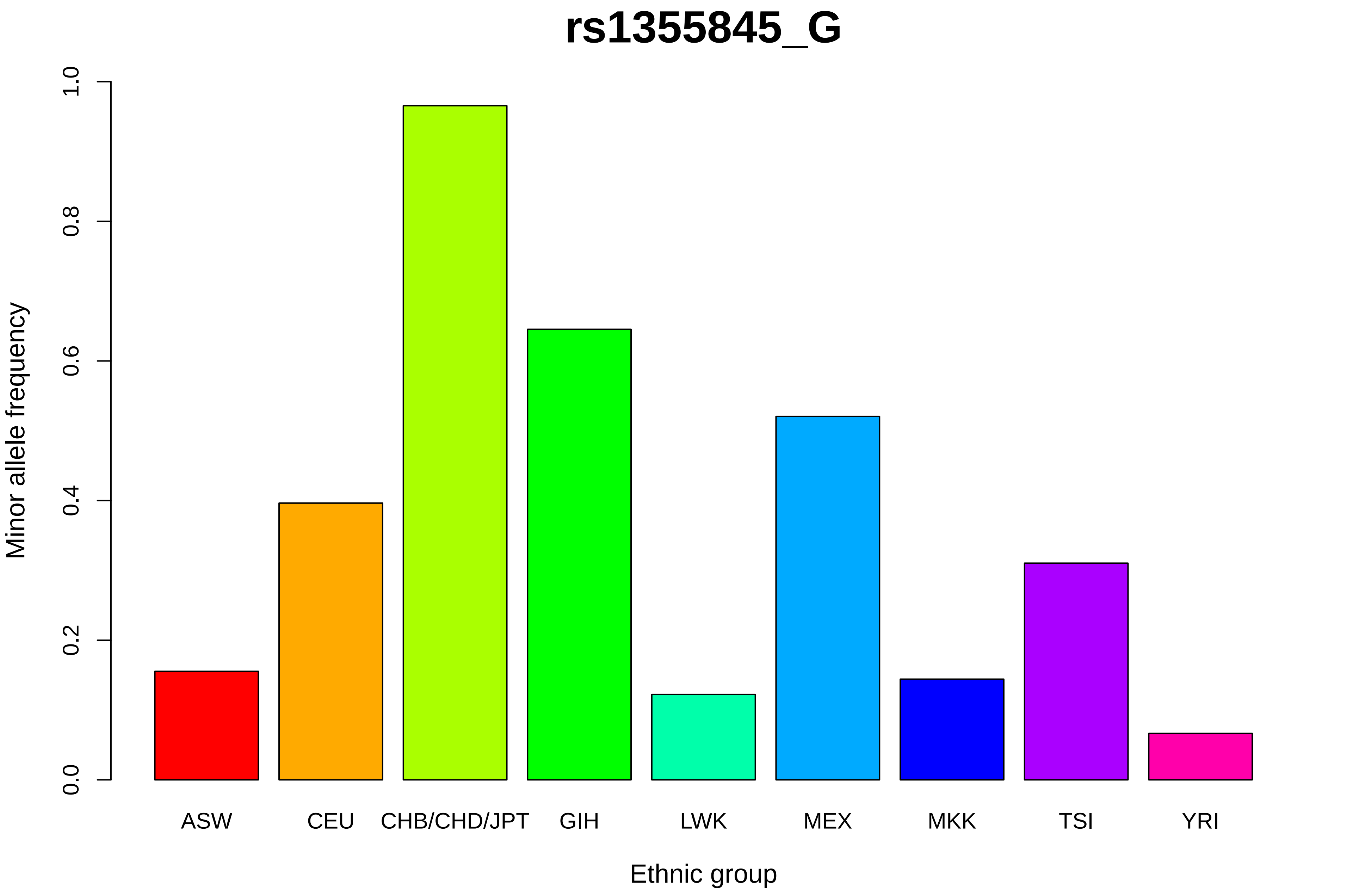
rs1355845_G
Minor allele frequency
0.0
0.2
0.4
0.6
0.8
1.0
ASW
CEU
CHB/CHD/JPT
GIH
LWK
MEX
MKK
TSI
YRI
Ethnic group

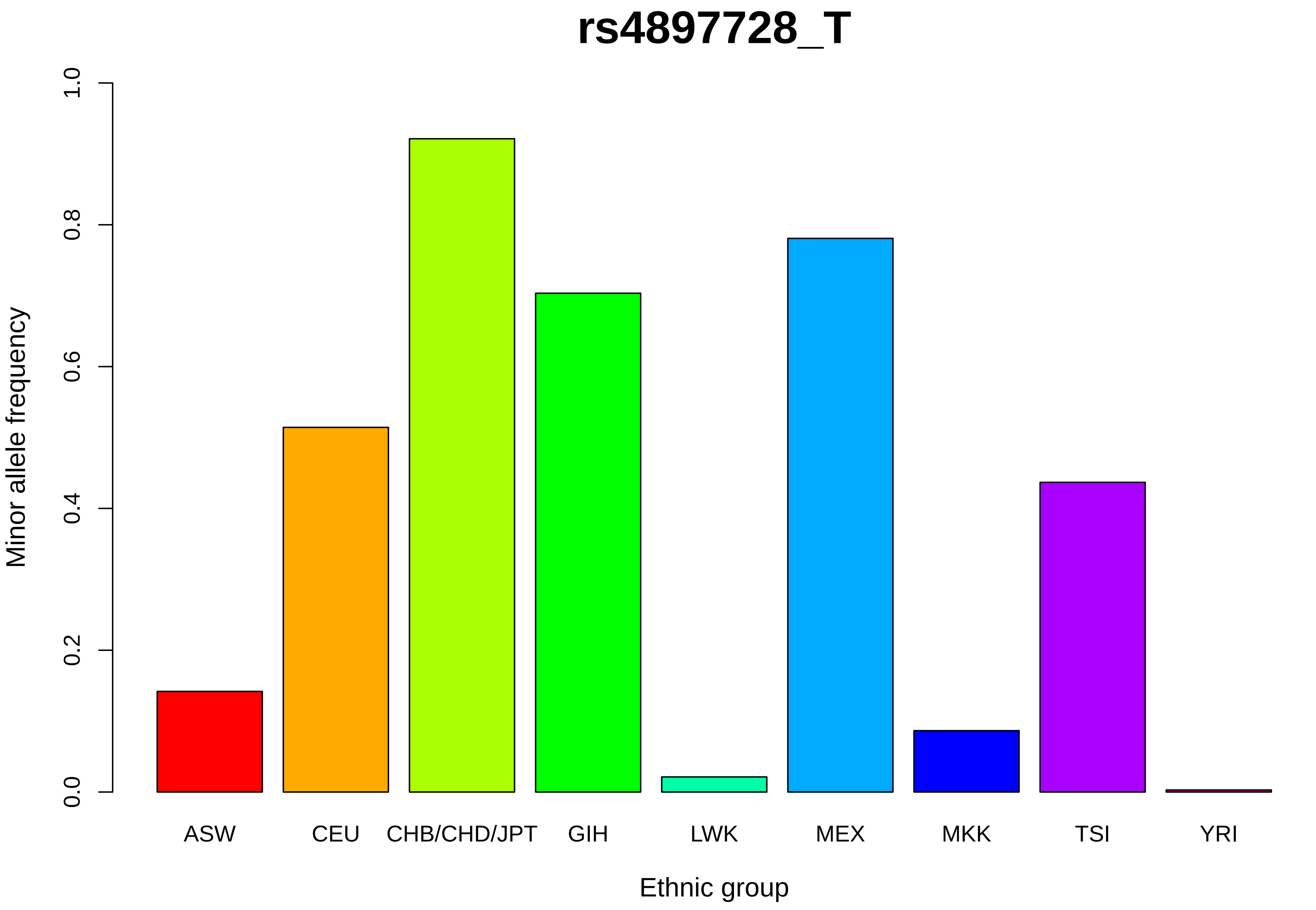
rs4897728_T
Minor allele frequency
0.0
0.2
0.4
0.6
0.8
1.0
ASW
CEU
CHB/CHD/JPT
GIH
LWK
MEX
MKK
TSI
YRI
Ethnic group

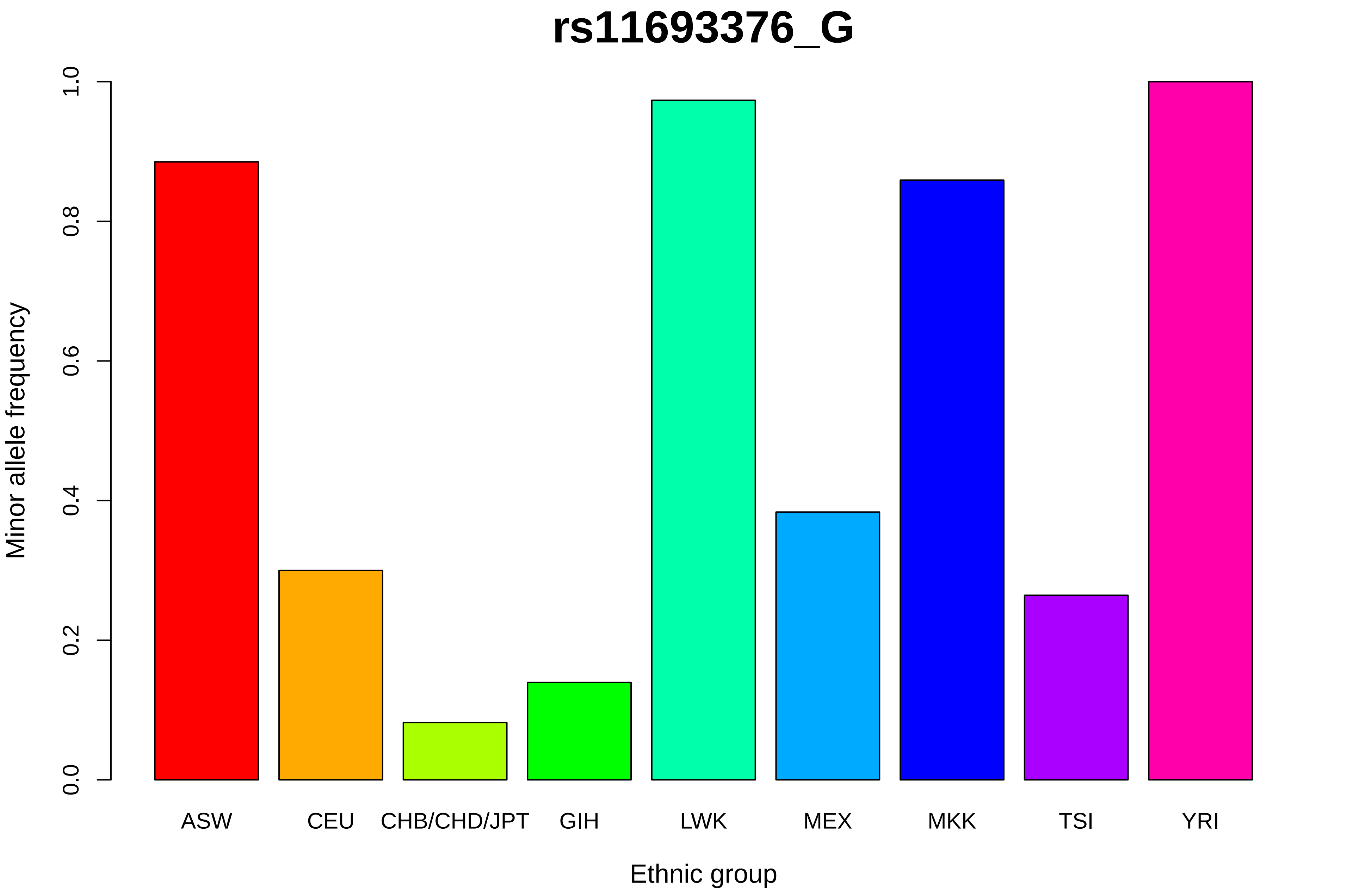
rs11693376_G
Minor allele frequency
0.0
0.2
0.4
0.6
0.8
1.0
ASW
CEU
CHB/CHD/JPT
GIH
LWK
MEX
MKK
TSI
YRI
Ethnic group

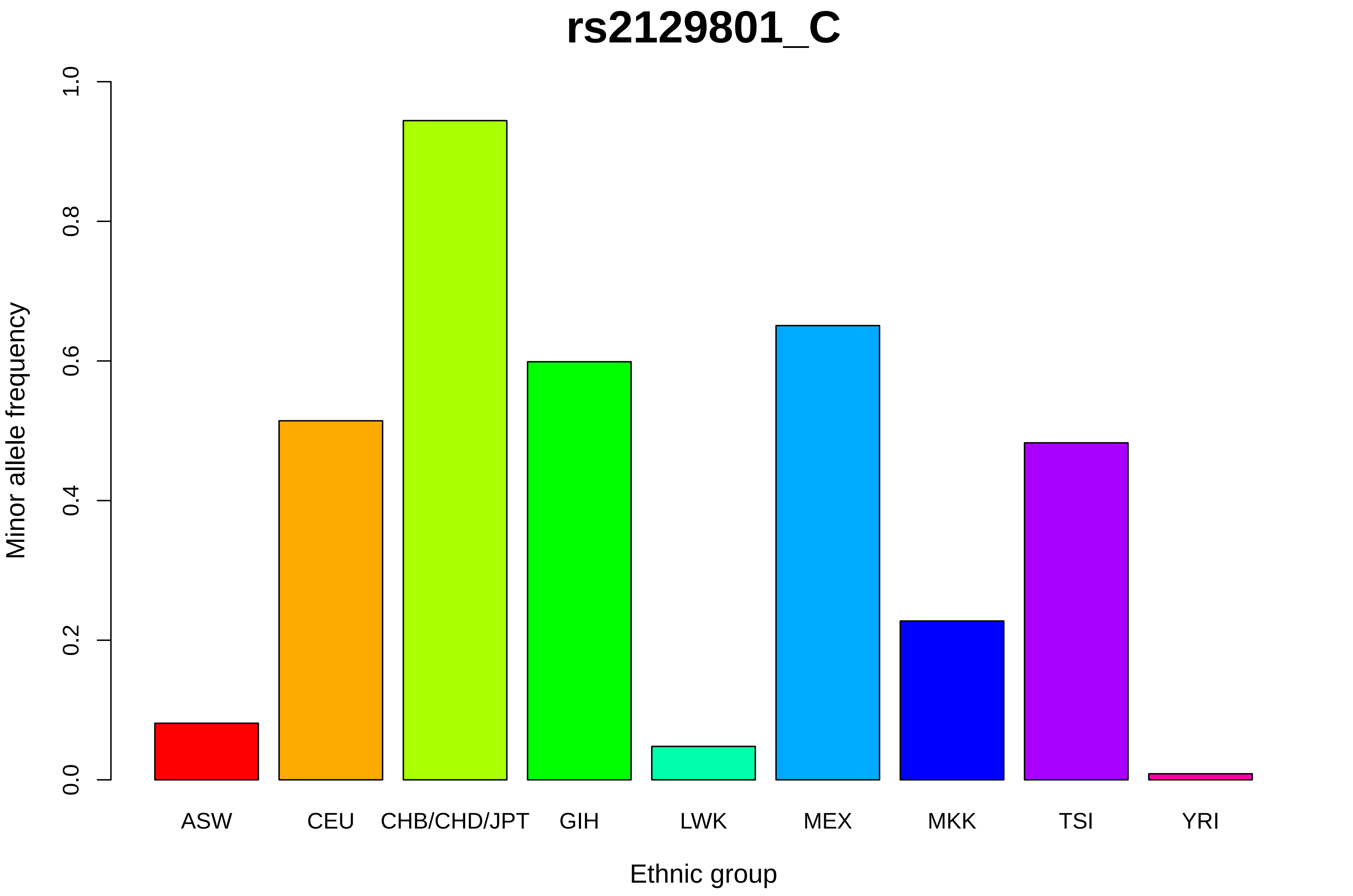
rs2129801_C
Minor allele frequency
0.0
0.2
0.4
0.6
0.8
1.0
ASW
CEU
CHB/CHD/JPT
GIH
LWK
MEX
MKK
TSI
YRI
Ethnic group

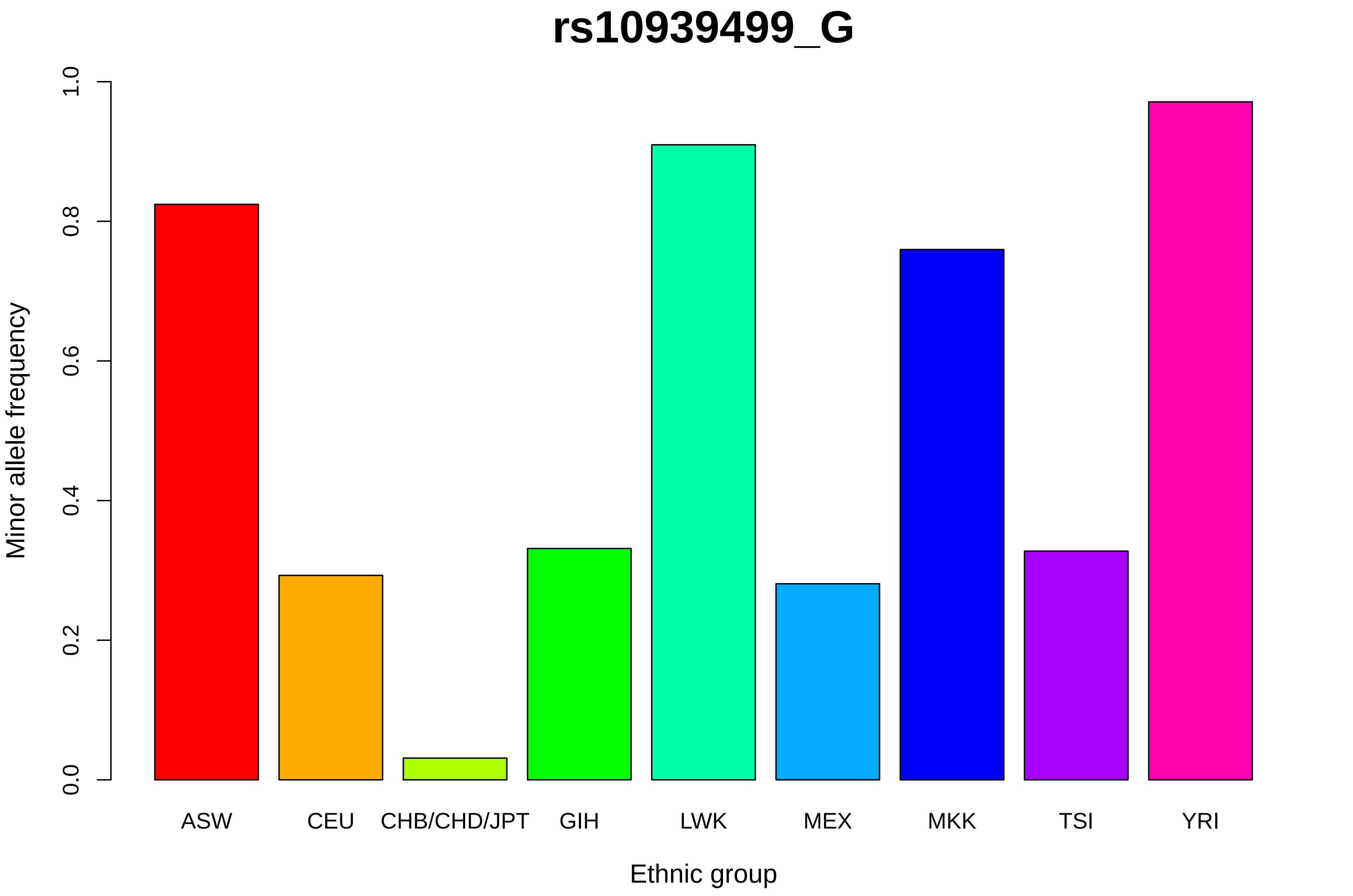
rs10939499_G
Minor allele frequency
0.0
0.2
0.4
0.6
0.8
1.0
ASW
CEU
CHB/CHD/JPT
GIH
LWK
MEX
MKK
TSI
YRI
Ethnic group

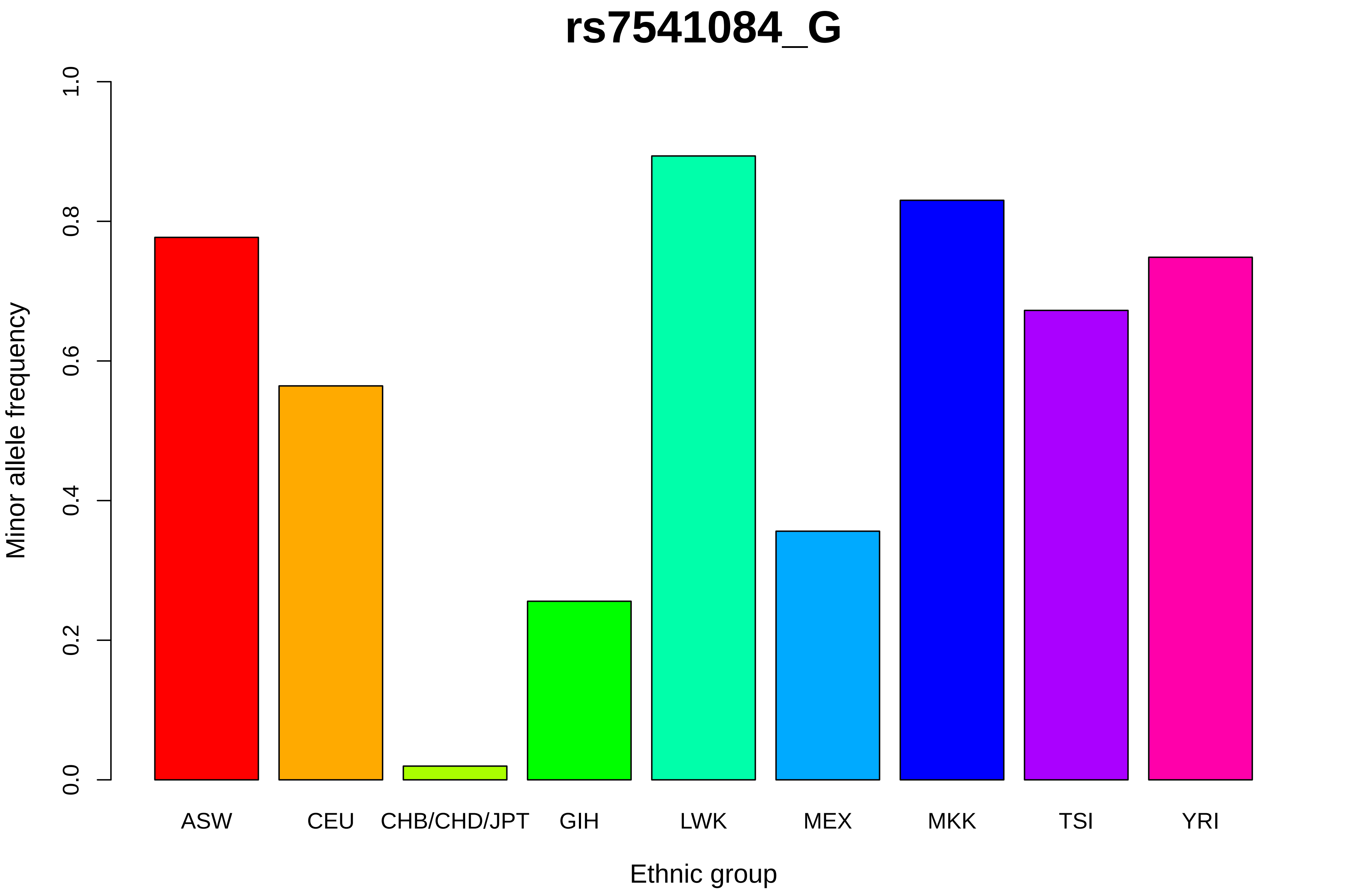
rs7541084_G
Minor allele frequency
0.0
0.2
0.4
0.6
0.8
1.0
ASW
CEU
CHB/CHD/JPT
GIH
LWK
MEX
MKK
TSI
YRI
Ethnic group

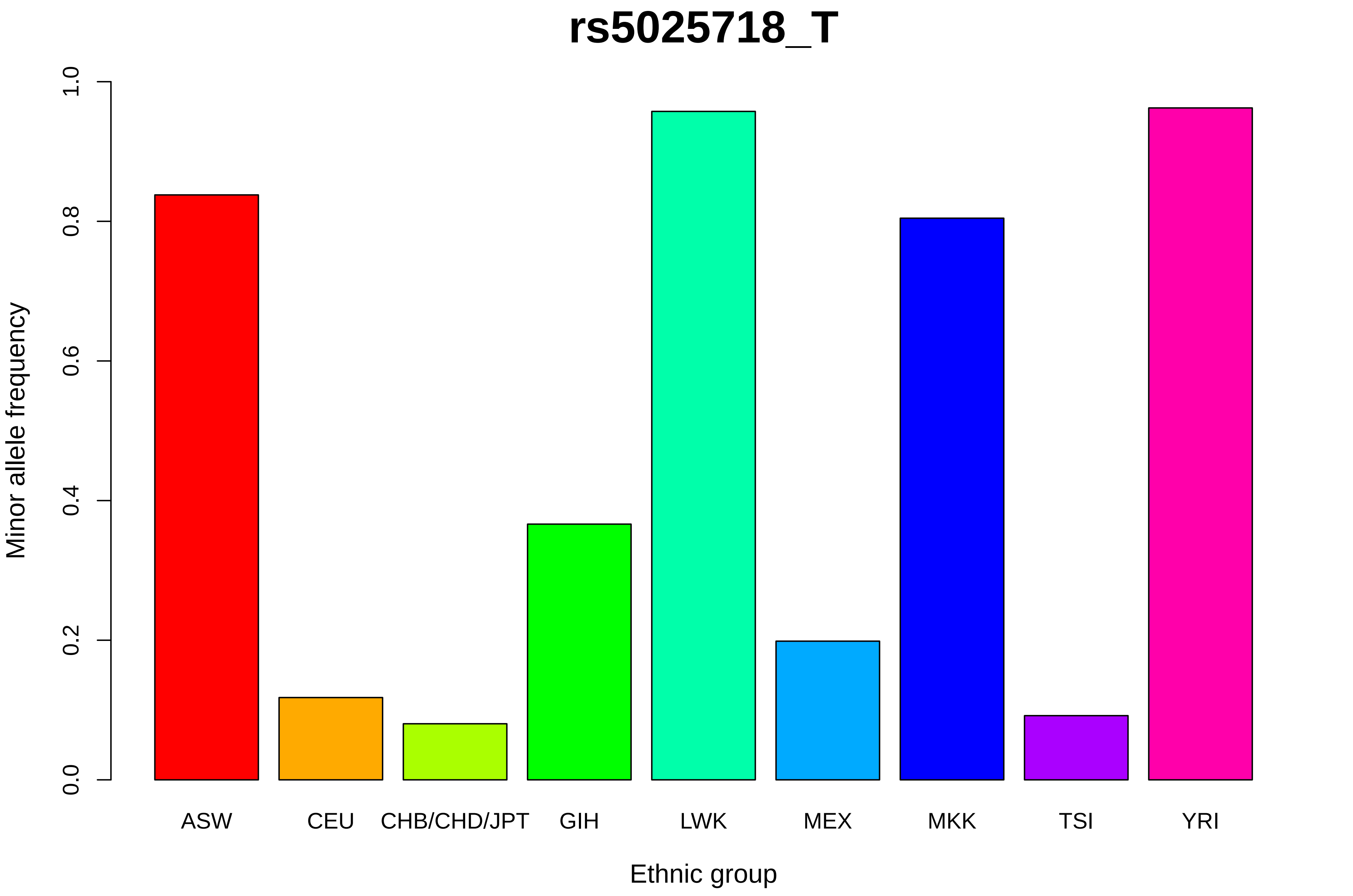
rs5025718_T
Minor allele frequency
0.0
0.2
0.4
0.6
0.8
1.0
ASW
CEU
CHB/CHD/JPT
GIH
LWK
MEX
MKK
TSI
YRI
Ethnic group

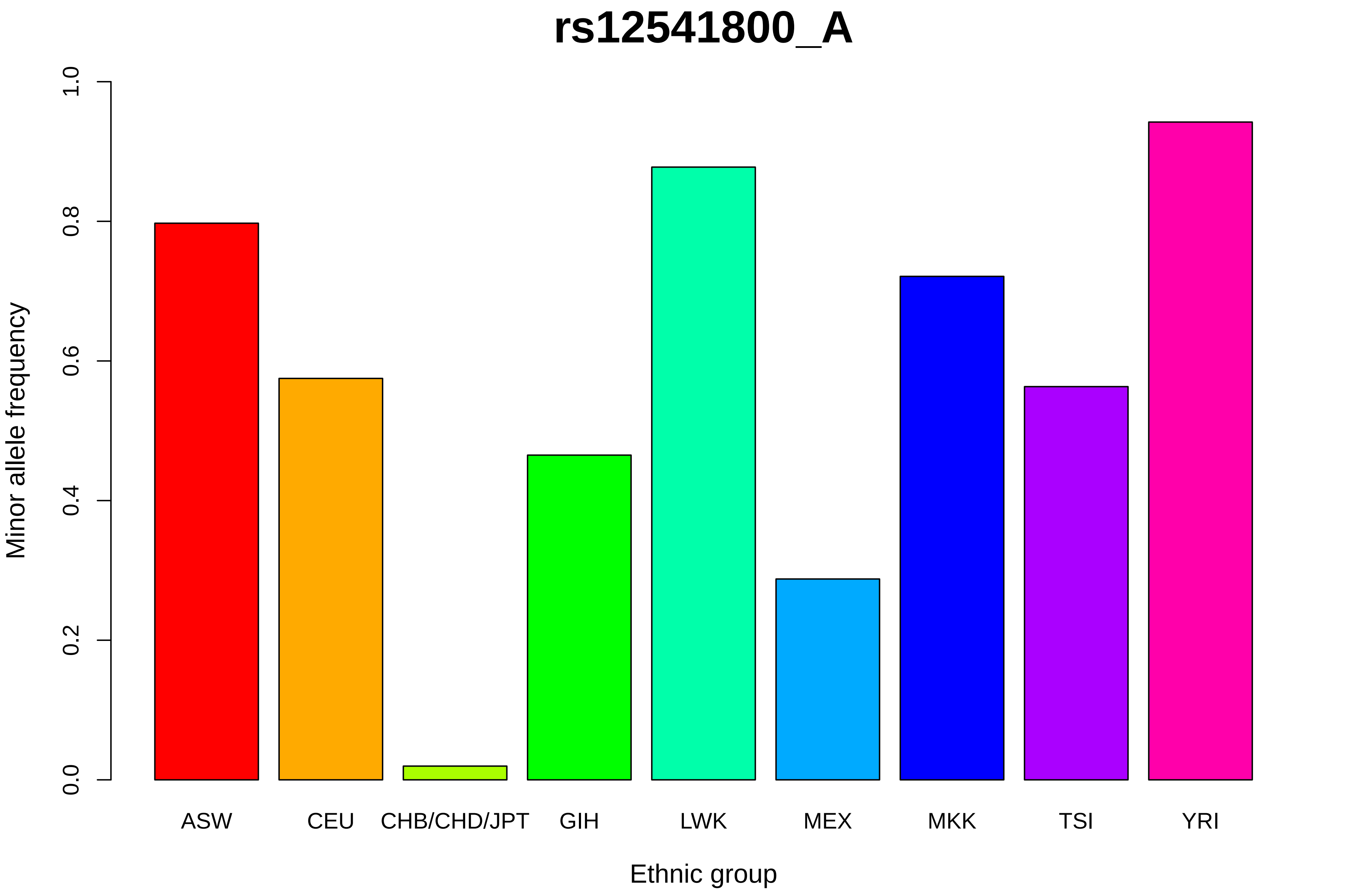
rs12541800_A
Minor allele frequency
0.0
0.2
0.4
0.6
0.8
1.0
ASW
CEU
CHB/CHD/JPT
GIH
LWK
MEX
MKK
TSI
YRI
Ethnic group

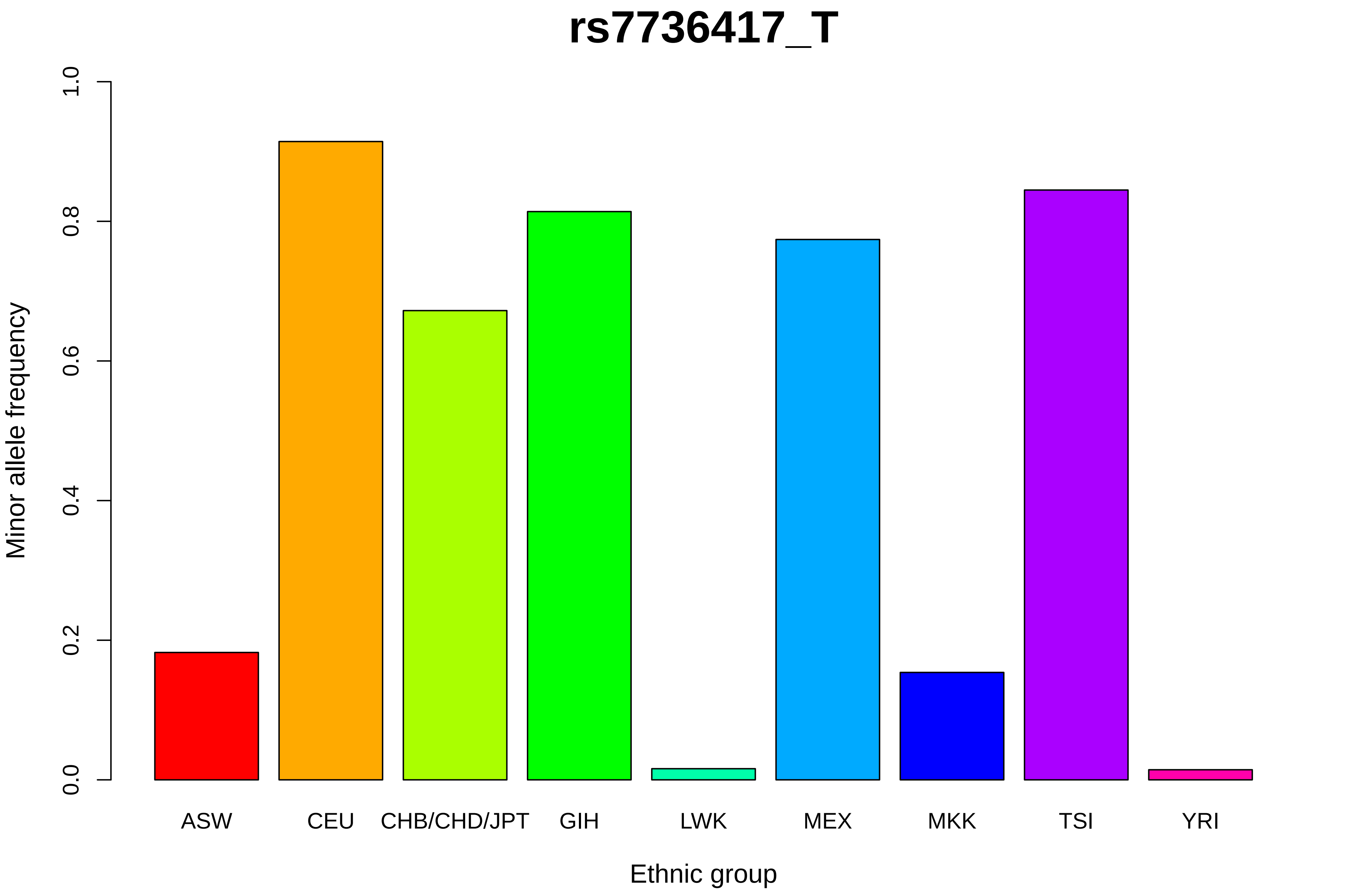
rs7736417_T
Minor allele frequency
0.0
0.2
0.4
0.6
0.8
1.0
ASW
CEU
CHB/CHD/JPT
GIH
LWK
MEX
MKK
TSI
YRI
Ethnic group

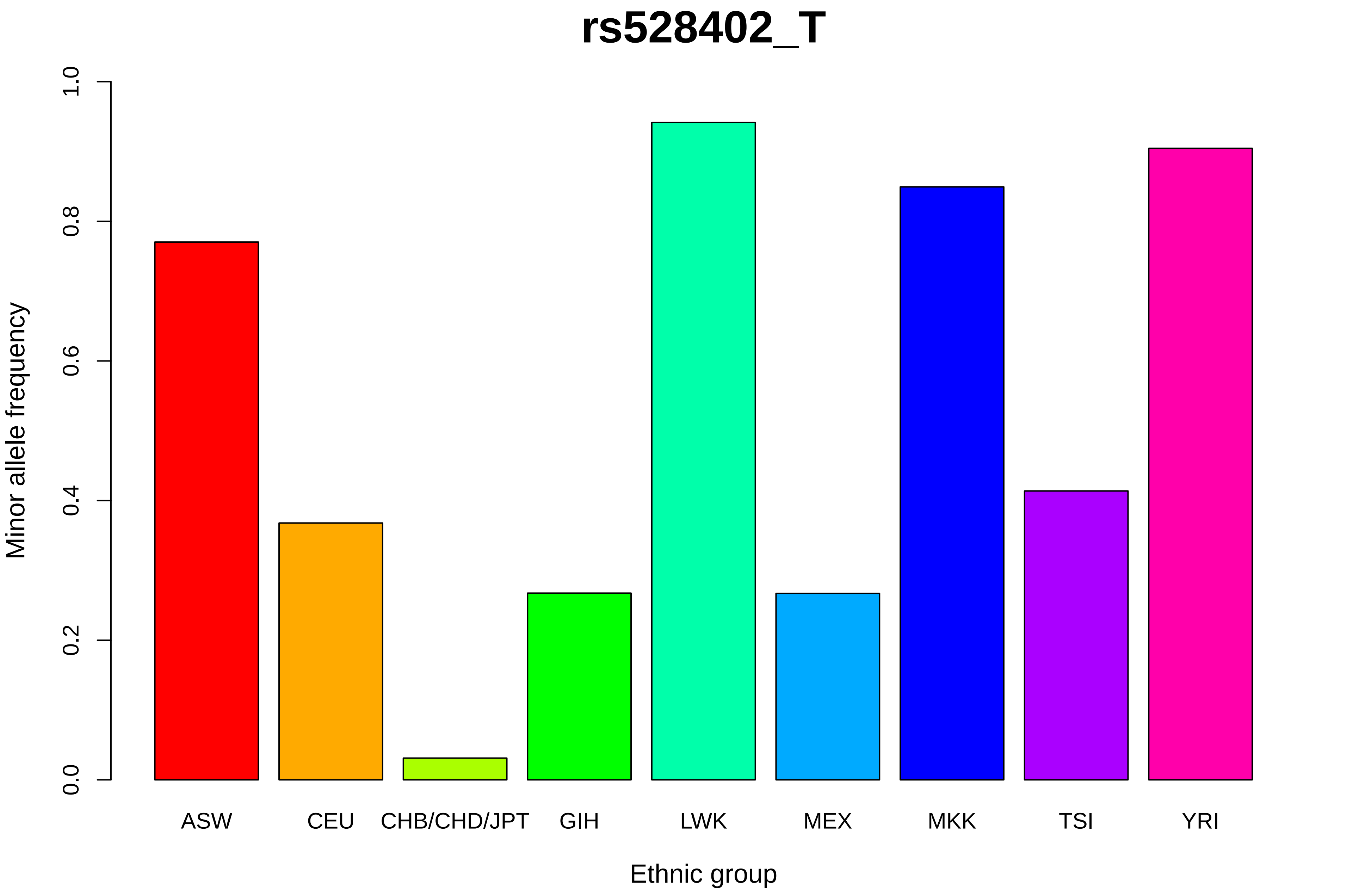
rs528402_T
Minor allele frequency
0.0
0.2
0.4
0.6
0.8
1.0
ASW
CEU
CHB/CHD/JPT
GIH
LWK
MEX
MKK
TSI
YRI
Ethnic group

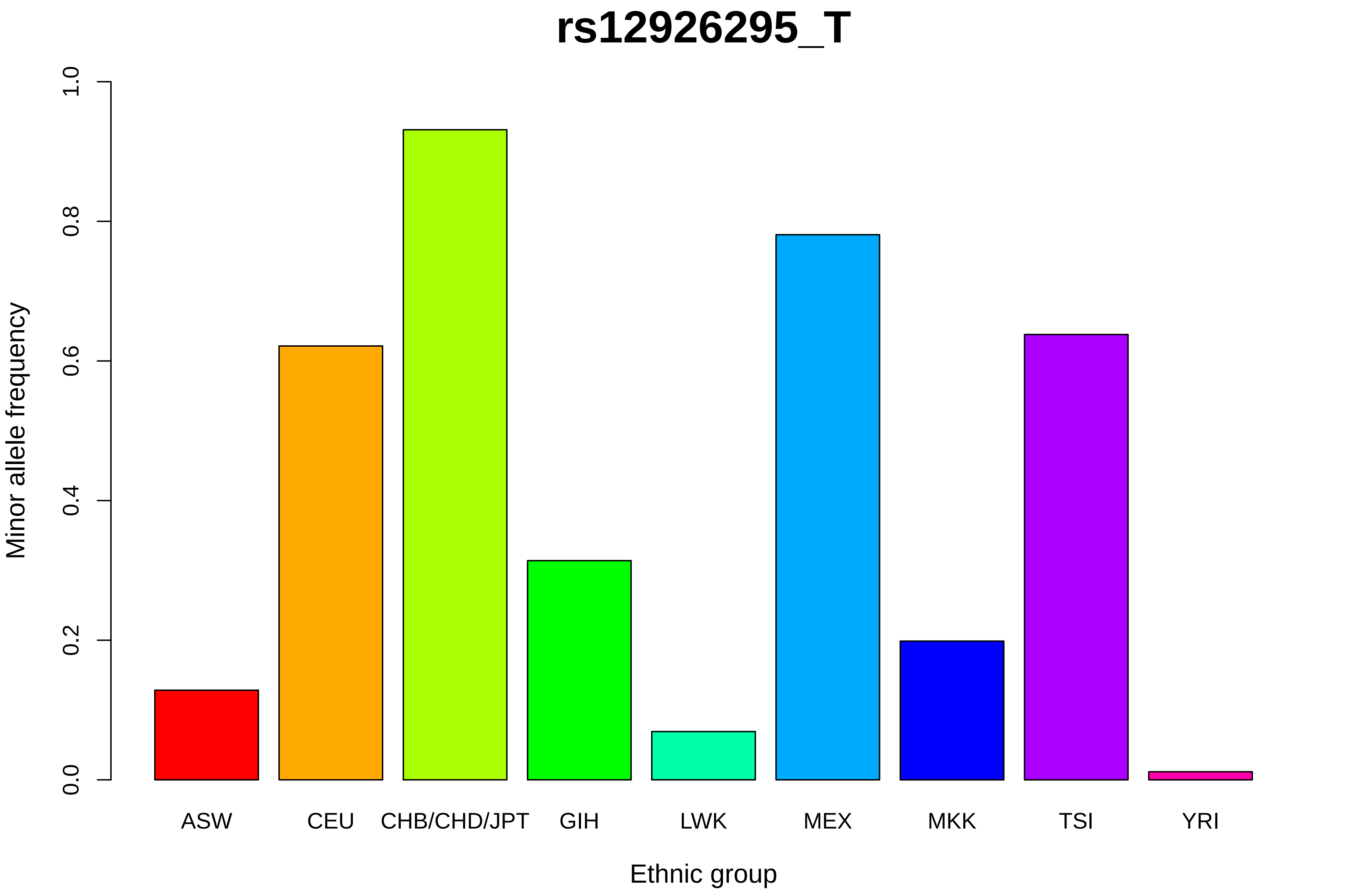
rs12926295_T
Minor allele frequency
0.0
0.2
0.4
0.6
0.8
1.0
ASW
CEU
CHB/CHD/JPT
GIH
LWK
MEX
MKK
TSI
YRI
Ethnic group

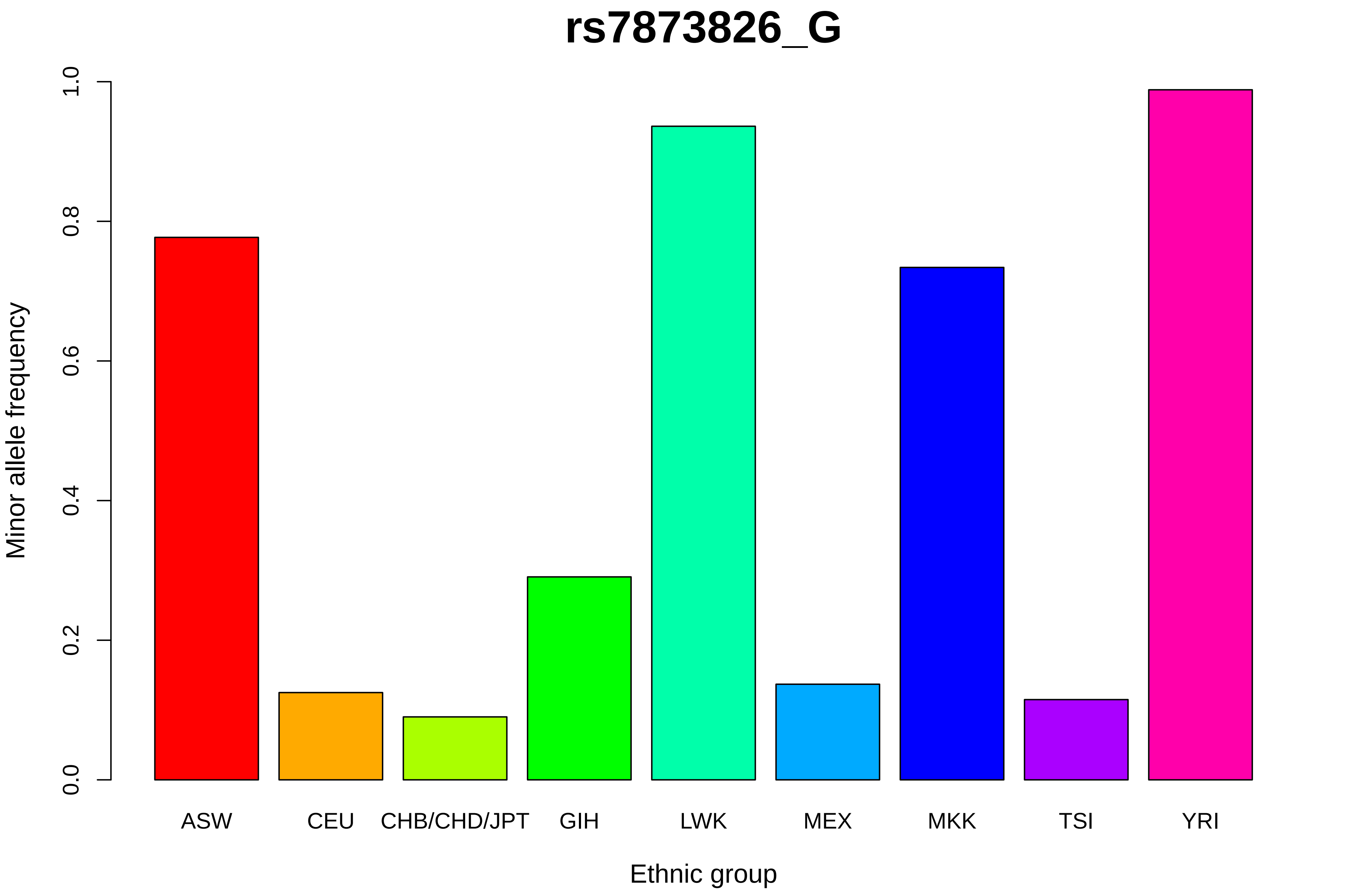
rs7873826_G
Minor allele frequency
0.0
0.2
0.4
0.6
0.8
1.0
ASW
CEU
CHB/CHD/JPT
GIH
LWK
MEX
MKK
TSI
YRI
Ethnic group

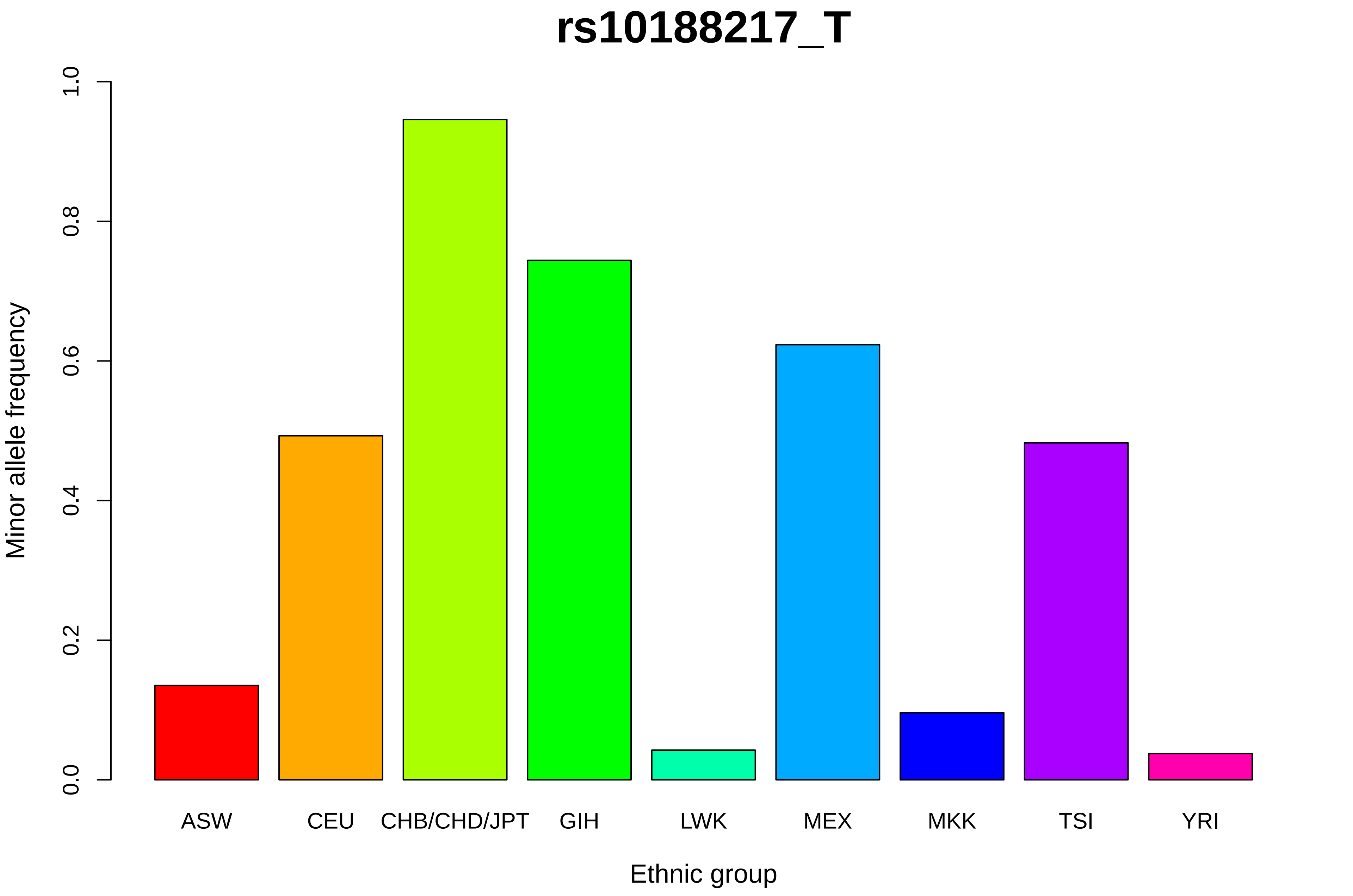
rs10188217_T
Minor allele frequency
0.0
0.2
0.4
0.6
0.8
1.0
ASW
CEU
CHB/CHD/JPT
GIH
LWK
MEX
MKK
TSI
YRI
Ethnic group

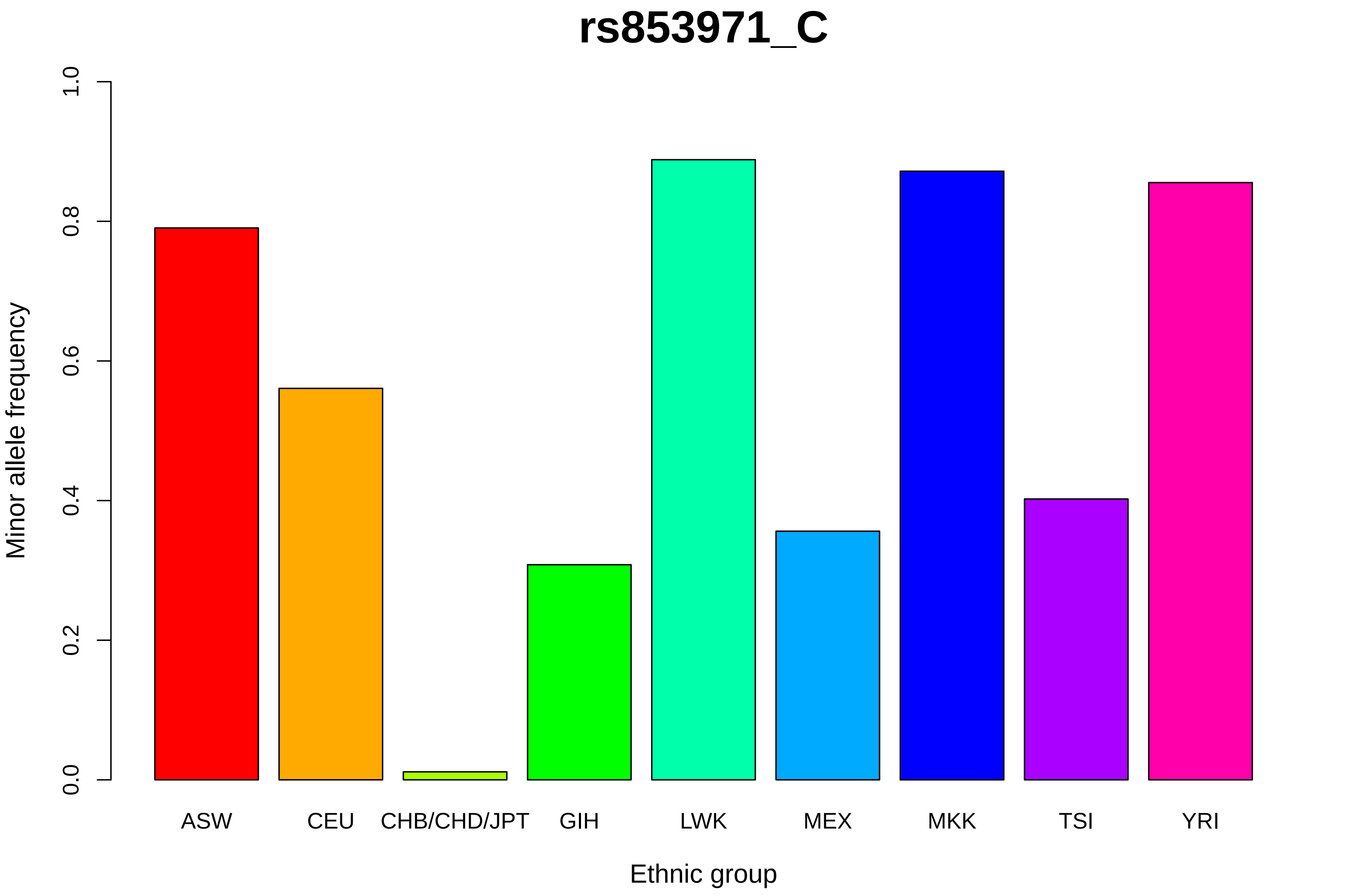
rs853971_C
Minor allele frequency
0.0
0.2
0.4
0.6
0.8
1.0
ASW
CEU
CHB/CHD/JPT
GIH
LWK
MEX
MKK
TSI
YRI
Ethnic group

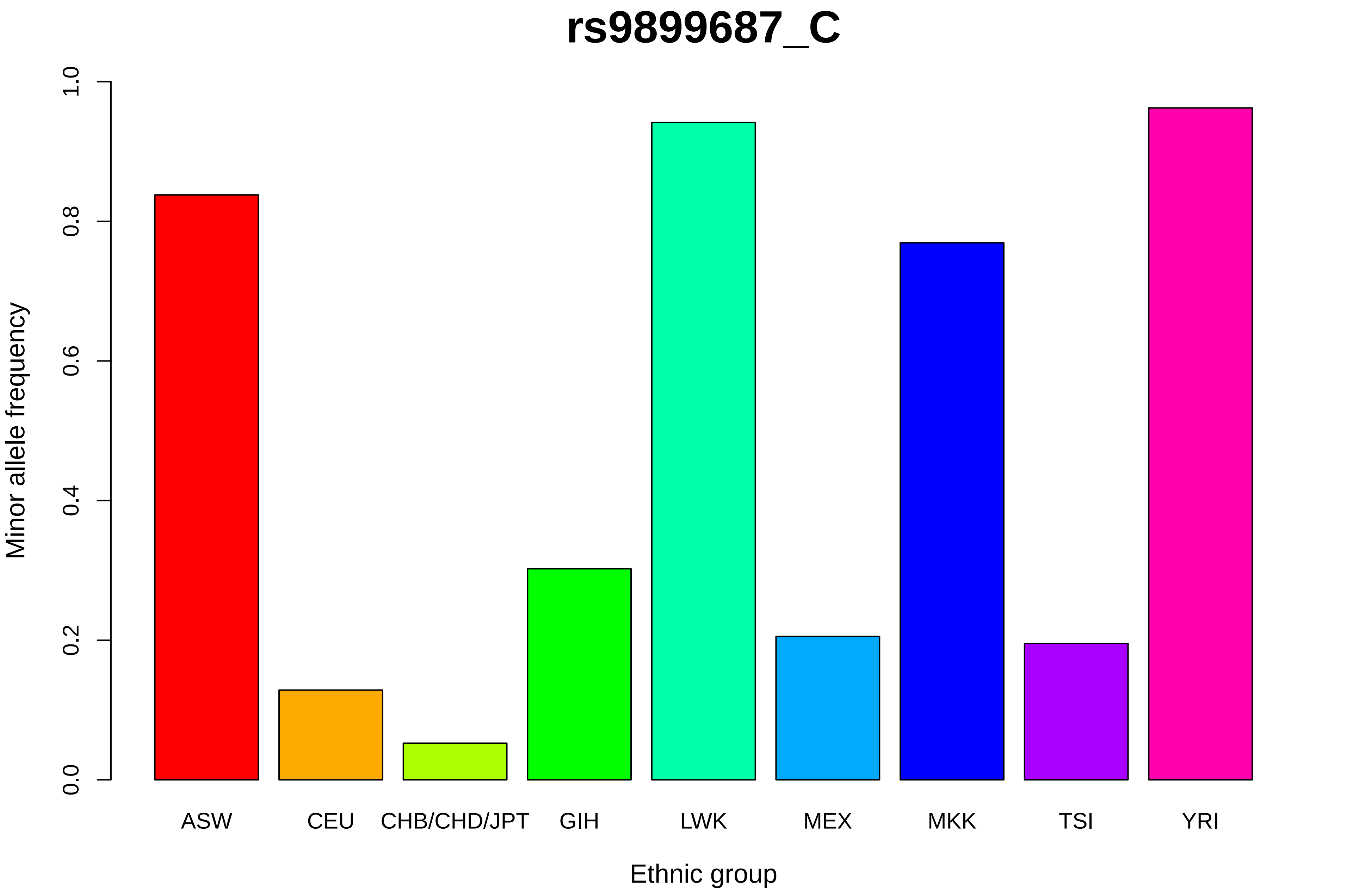
rs9899687_C
Minor allele frequency
0.0
0.2
0.4
0.6
0.8
1.0
ASW
CEU
CHB/CHD/JPT
GIH
LWK
MEX
MKK
TSI
YRI
Ethnic group

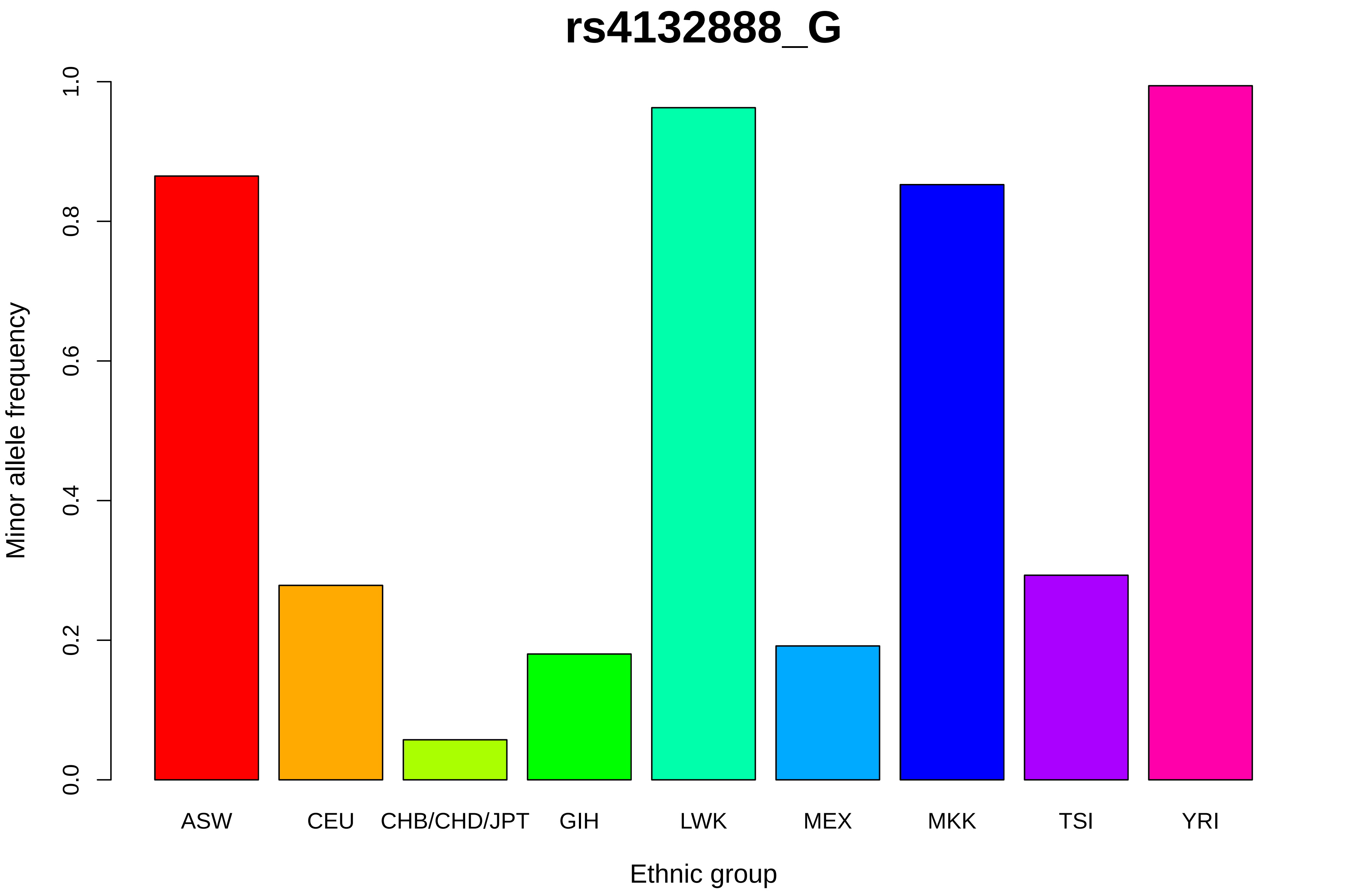

rs4132888_G
Minor allele frequency
0.0
0.2
0.4
0.6
0.8
1.0
ASW
CEU
CHB/CHD/JPT
GIH
LWK
MEX
MKK
TSI
YRI
Ethnic group

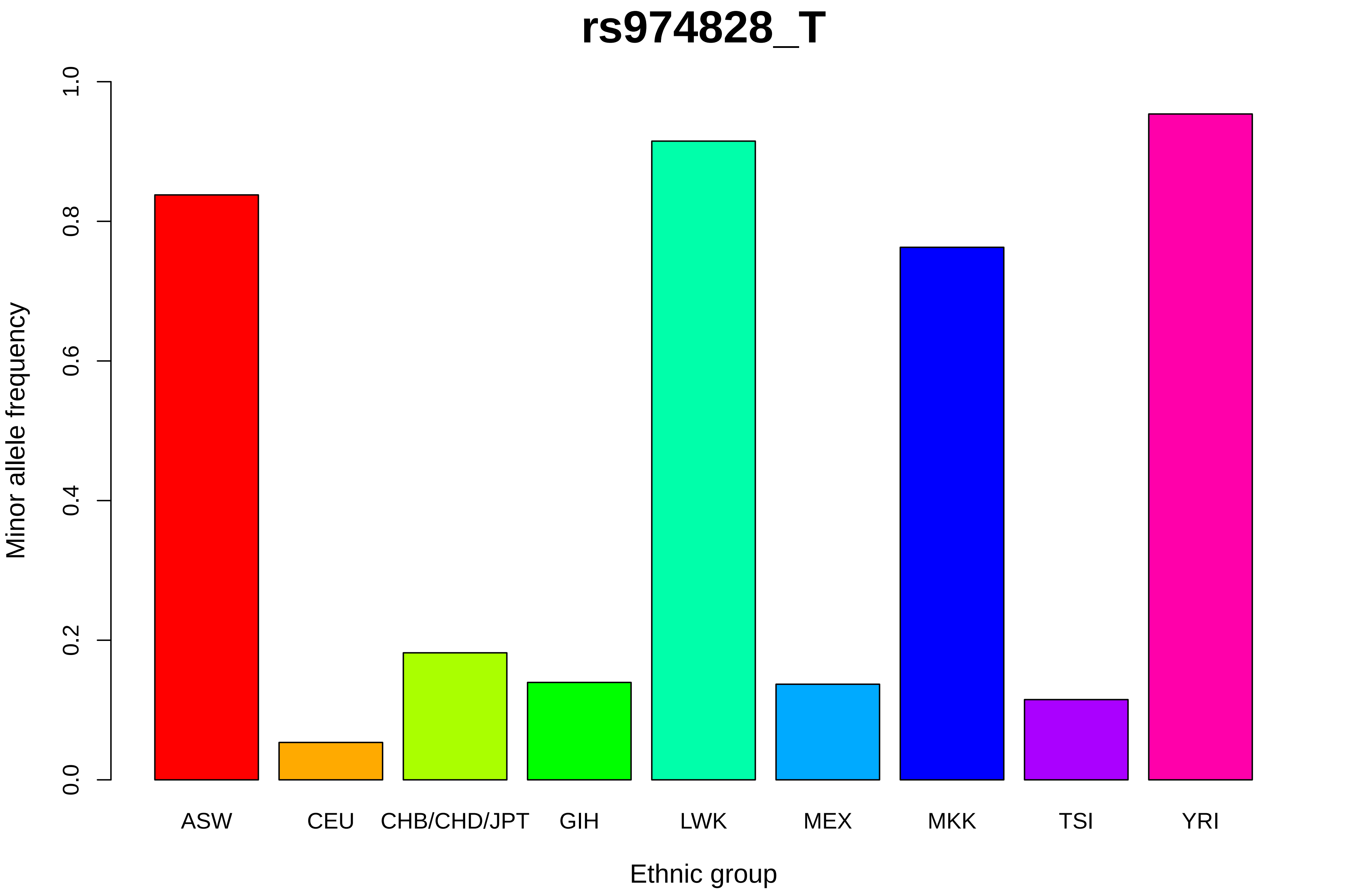
rs974828_T
Minor allele frequency
0.0
0.2
0.4
0.6
0.8
1.0
ASW
CEU
CHB/CHD/JPT
GIH
LWK
MEX
MKK
TSI
YRI
Ethnic group

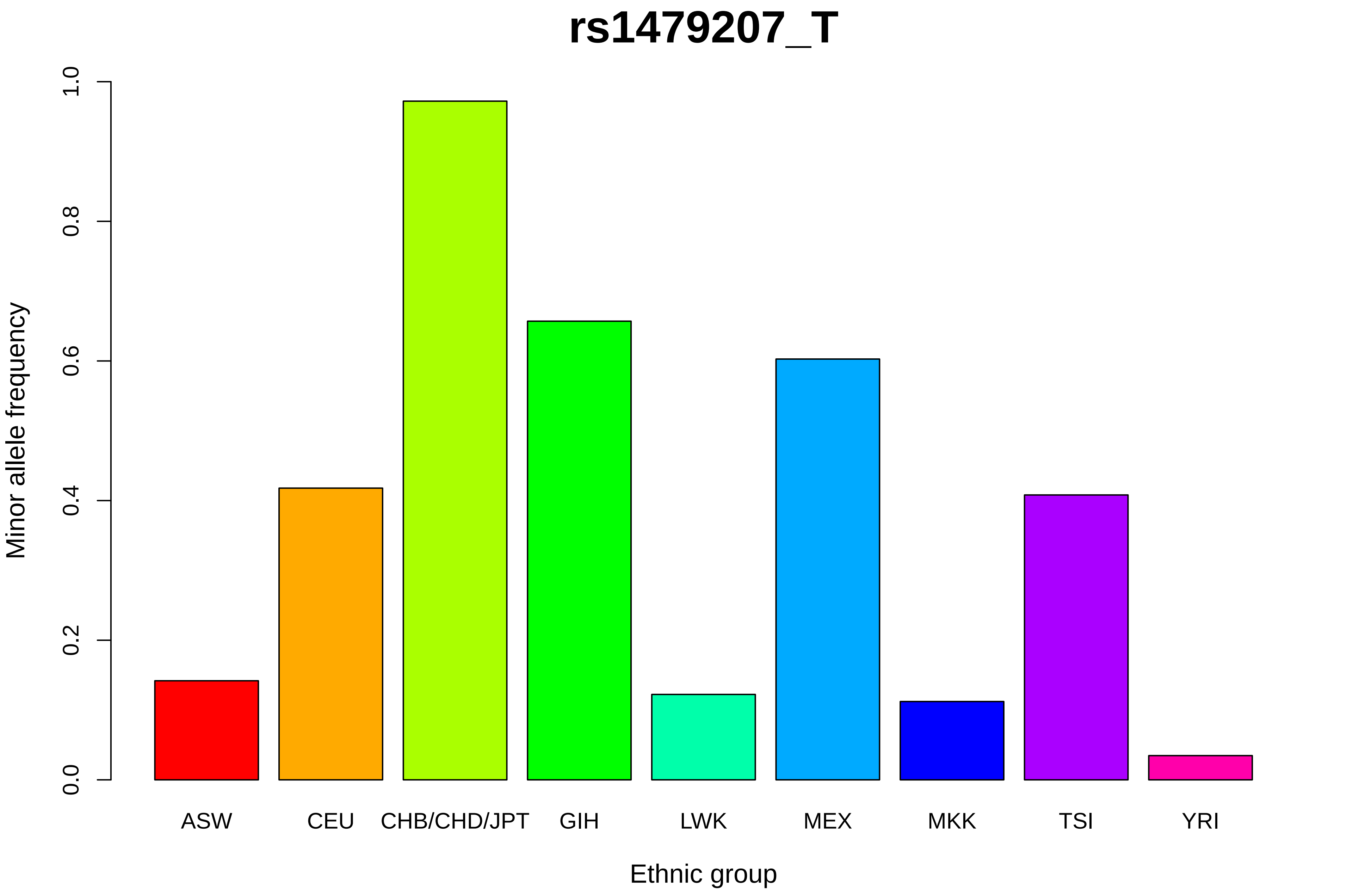

rs1479207_T
Minor allele frequency
0.0
0.2
0.4
0.6
0.8
1.0
ASW
CEU
CHB/CHD/JPT
GIH
LWK
MEX
MKK
TSI
YRI
Ethnic group

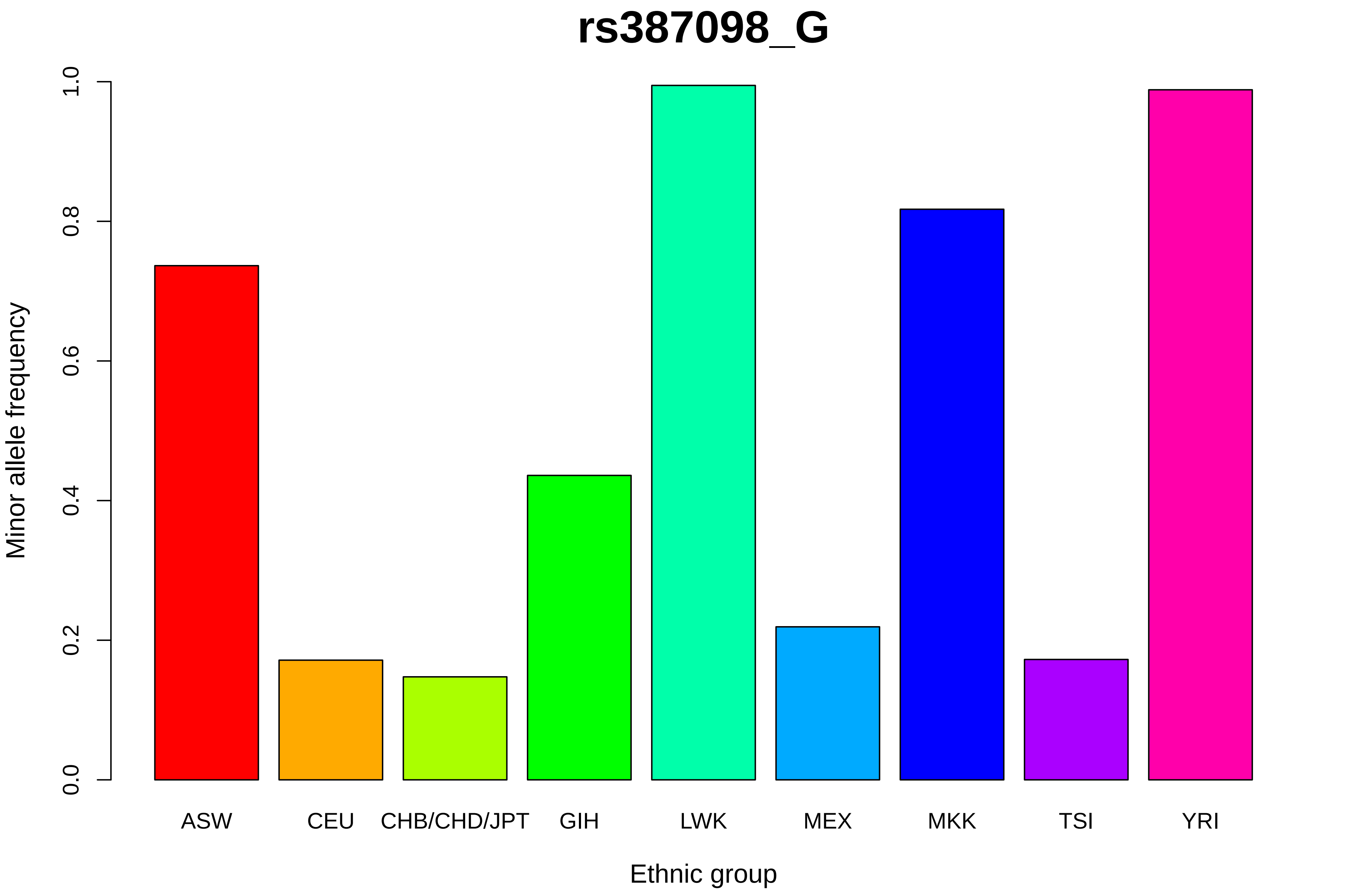
rs387098_G
Minor allele frequency
0.0
0.2
0.4
0.6
0.8
1.0
ASW
CEU
CHB/CHD/JPT
GIH
LWK
MEX
MKK
TSI
YRI
Ethnic group

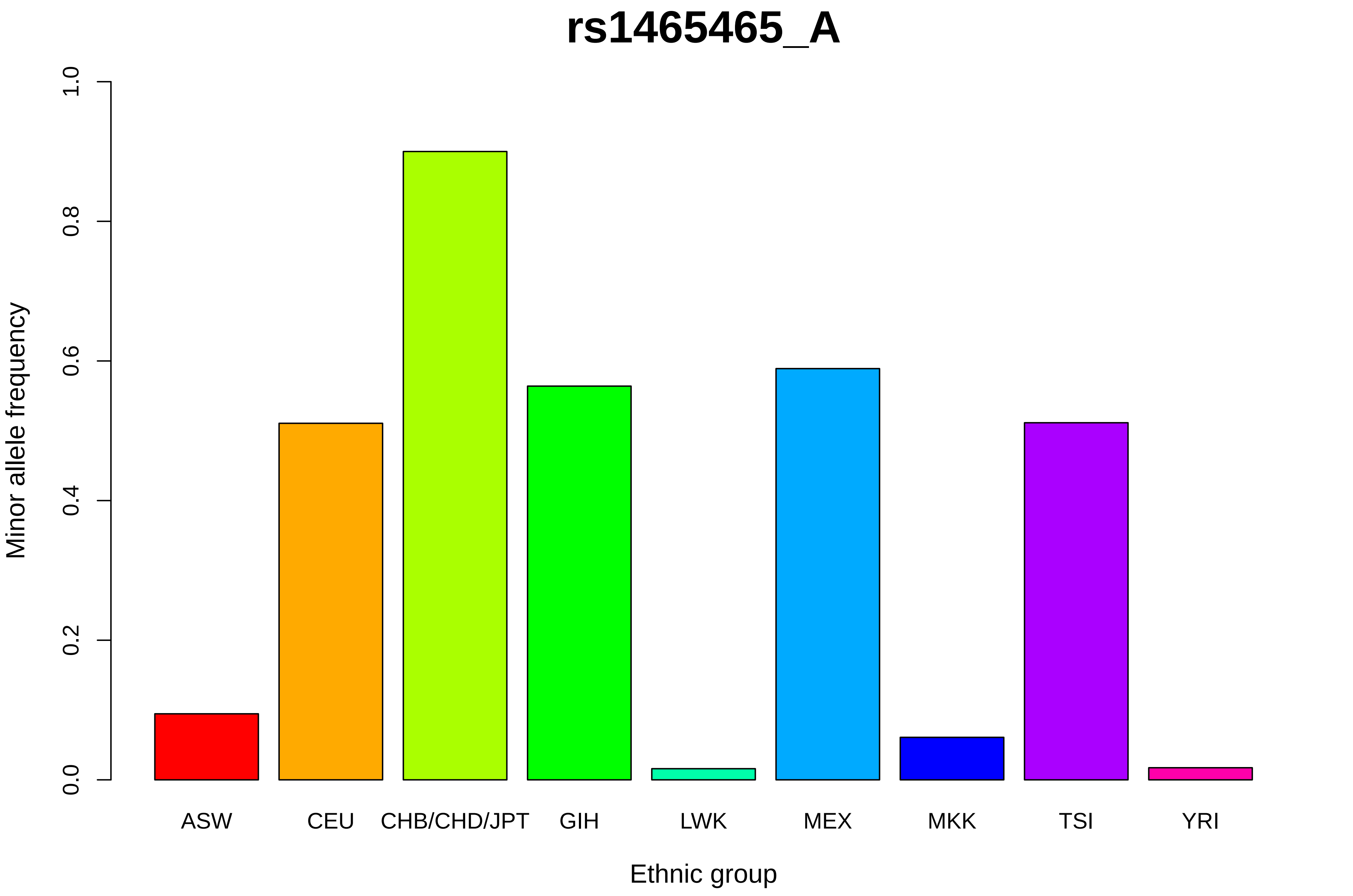

rs1465465_A
Minor allele frequency
0.0
0.2
0.4
0.6
0.8
1.0
ASW
CEU
CHB/CHD/JPT
GIH
LWK
MEX
MKK
TSI
YRI
Ethnic group

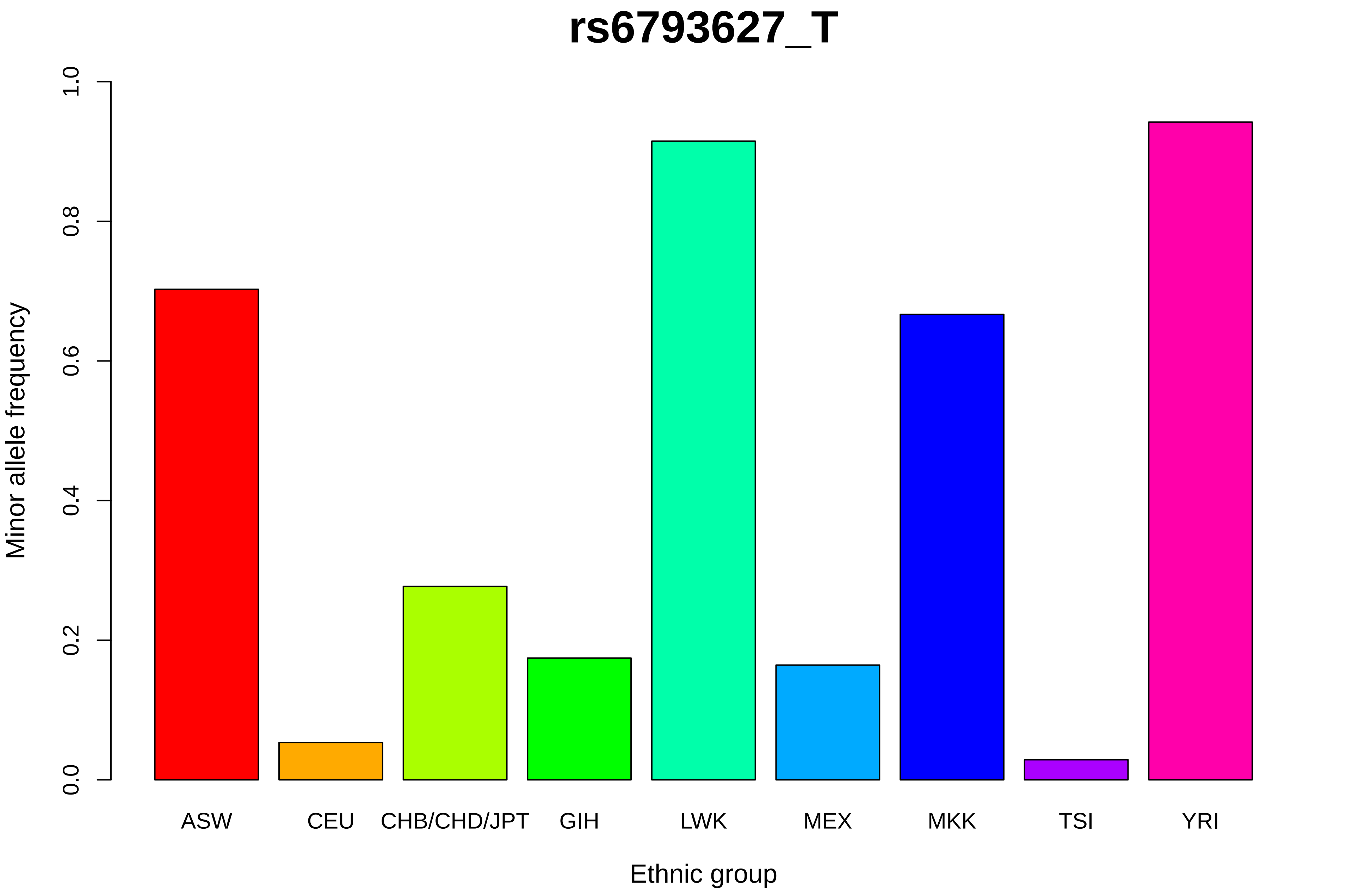

rs6793627_T
Minor allele frequency
0.0
0.2
0.4
0.6
0.8
1.0
ASW
CEU
CHB/CHD/JPT
GIH
LWK
MEX
MKK
TSI
YRI
Ethnic group

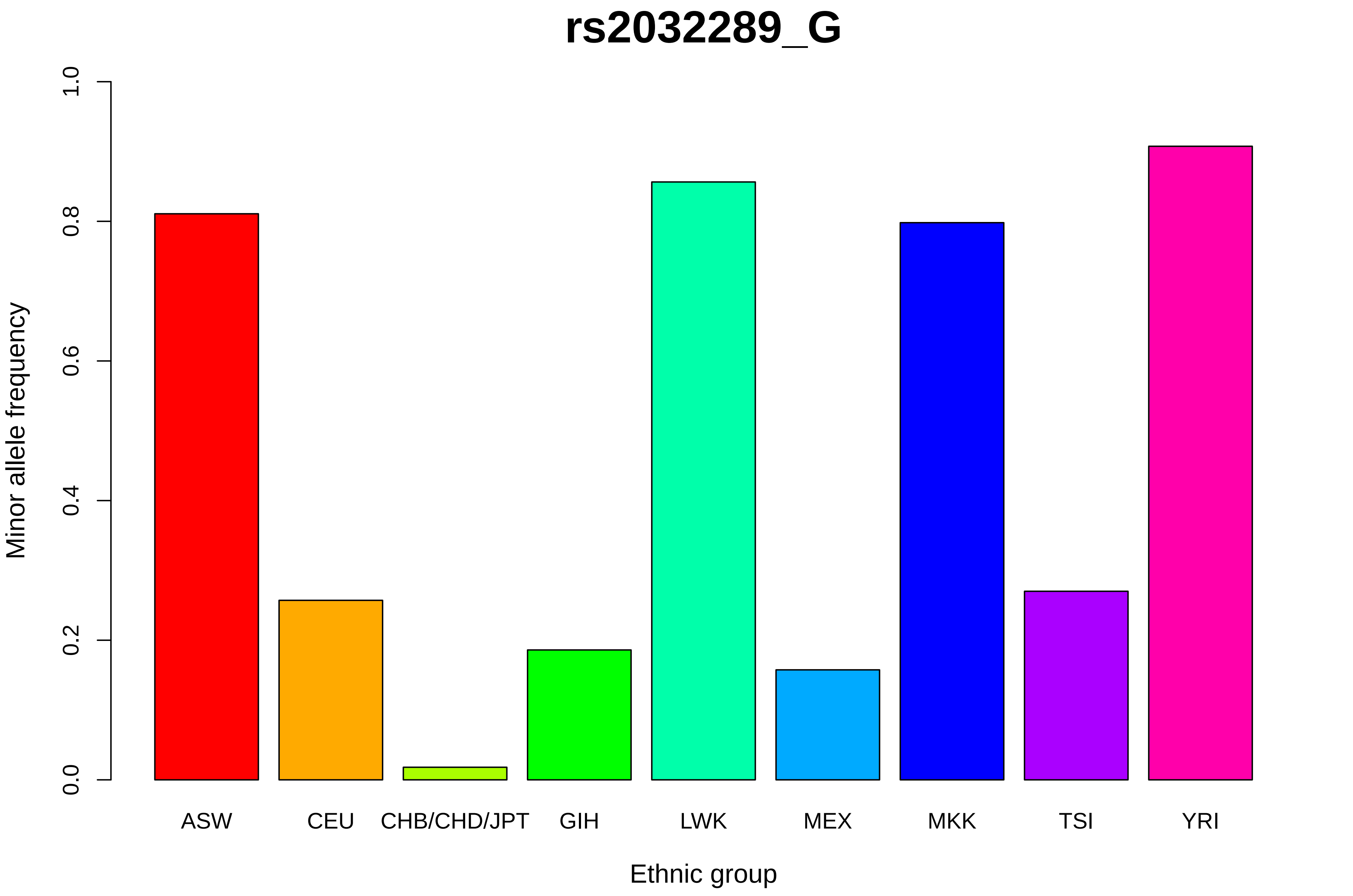
rs2032289_G
Minor allele frequency
0.0
0.2
0.4
0.6
0.8
1.0
ASW
CEU
CHB/CHD/JPT
GIH
LWK
MEX
MKK
TSI
YRI
Ethnic group

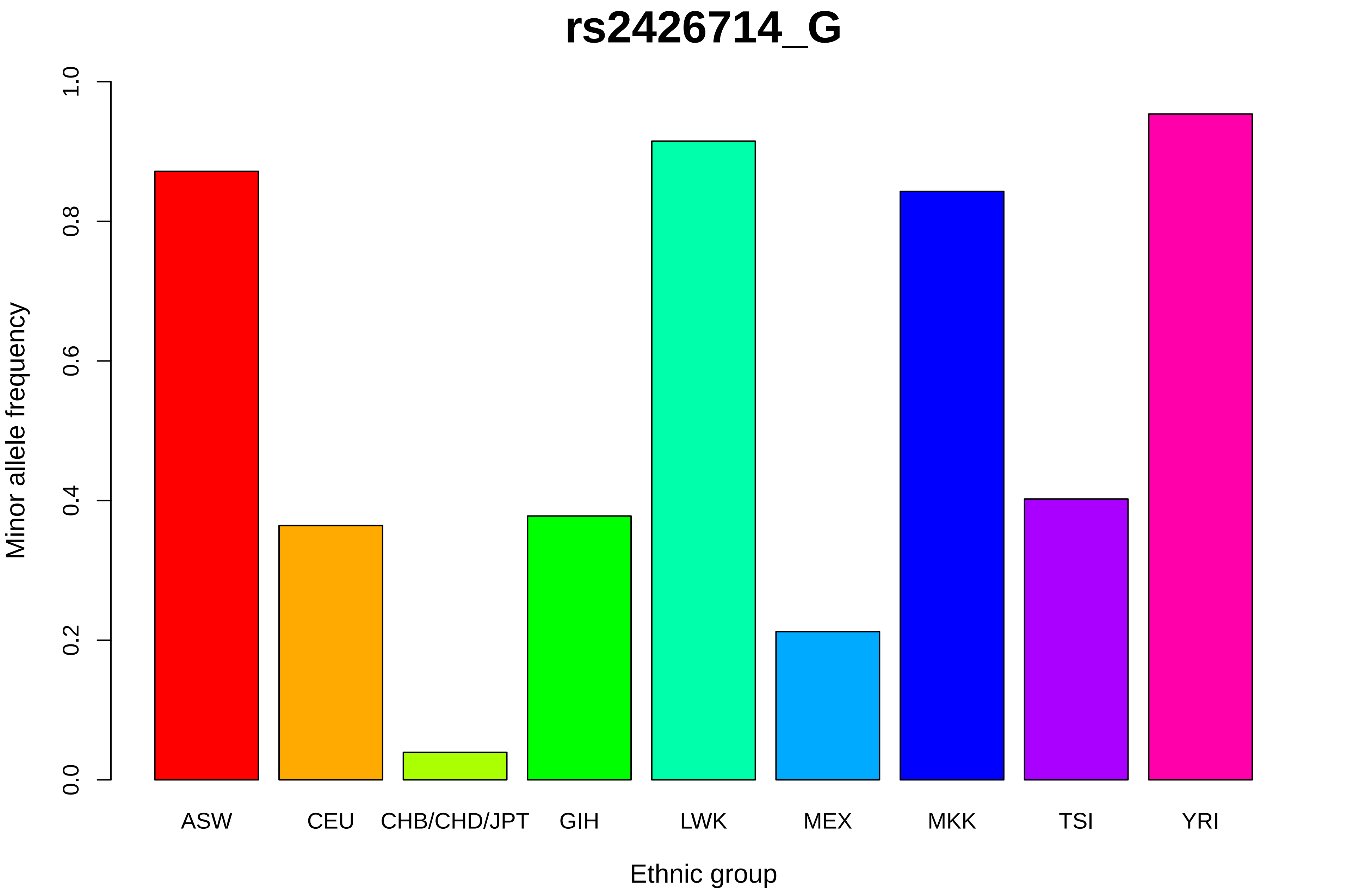
rs2426714_G
Minor allele frequency
0.0
0.2
0.4
0.6
0.8
1.0
ASW
CEU
CHB/CHD/JPT
GIH
LWK
MEX
MKK
TSI
YRI
Ethnic group

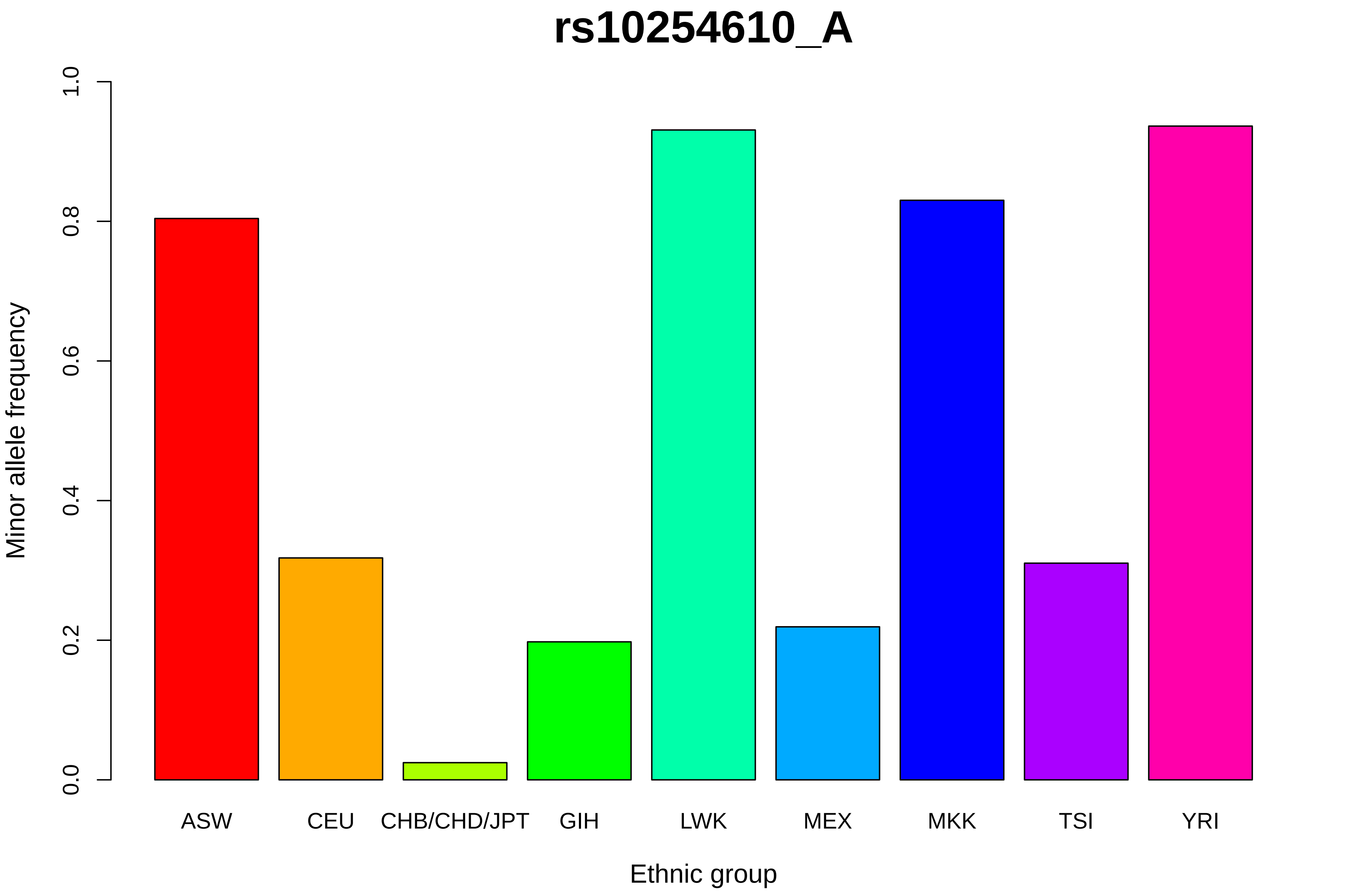
rs10254610_A
Minor allele frequency
0.0
0.2
0.4
0.6
0.8
1.0
ASW
CEU
CHB/CHD/JPT
GIH
LWK
MEX
MKK
TSI
YRI
Ethnic group

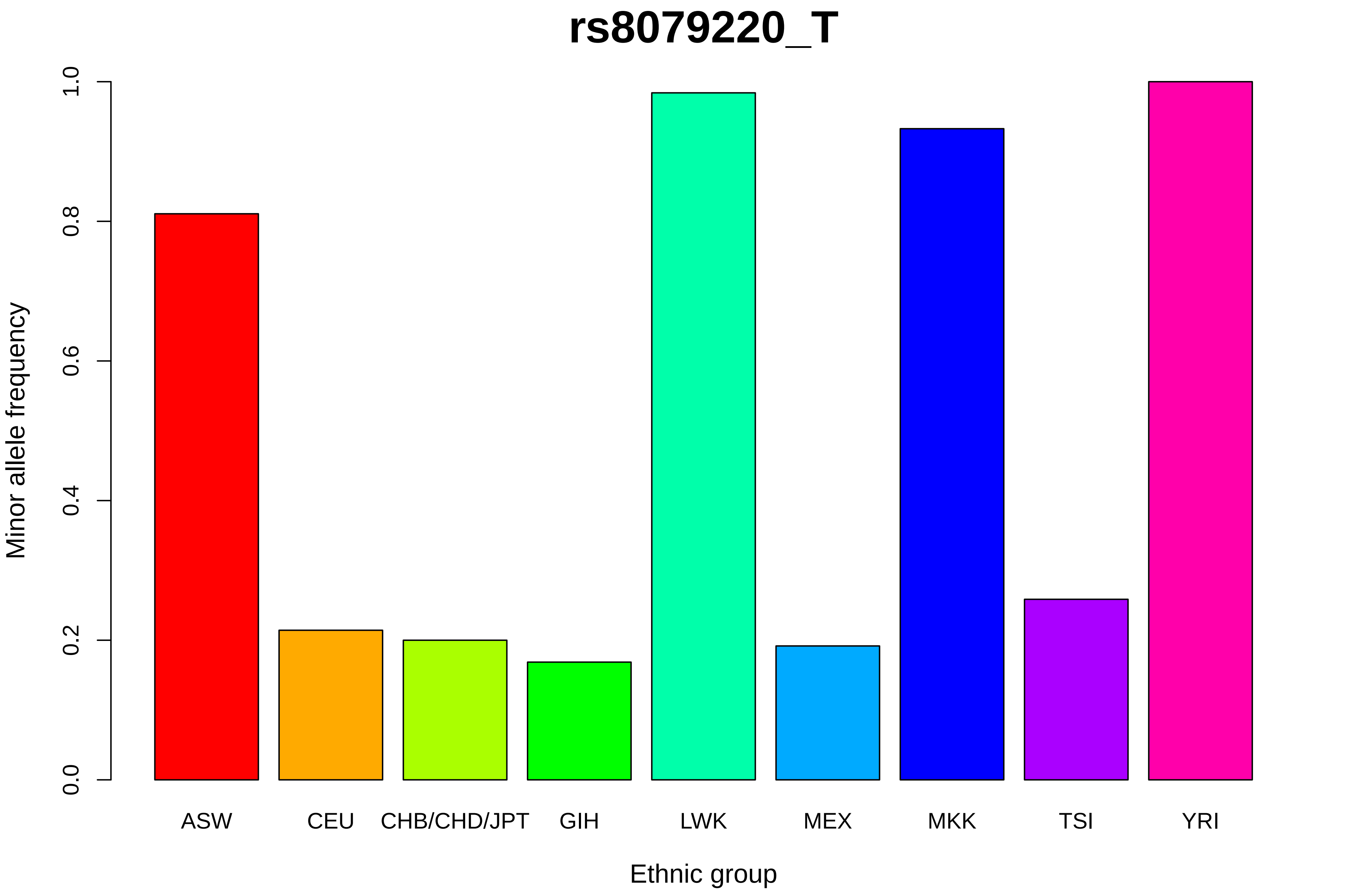
rs8079220_T
Minor allele frequency
0.0
0.2
0.4
0.6
0.8
1.0
ASW
CEU
CHB/CHD/JPT
GIH
LWK
MEX
MKK
TSI
YRI
Ethnic group

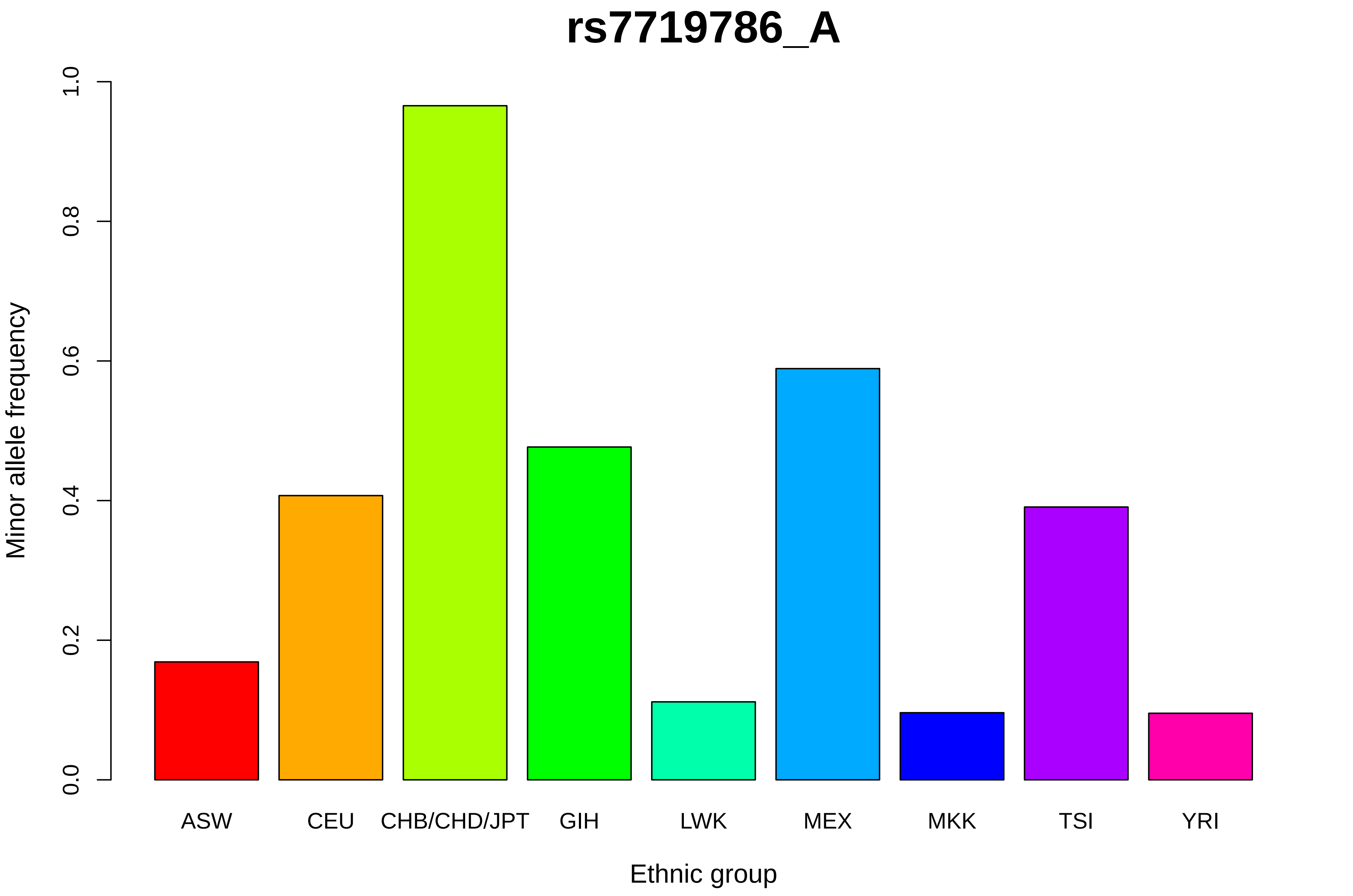
rs7719786_A
Minor allele frequency
0.0
0.2
0.4
0.6
0.8
1.0
ASW
CEU
CHB/CHD/JPT
GIH
LWK
MEX
MKK
TSI
YRI
Ethnic group

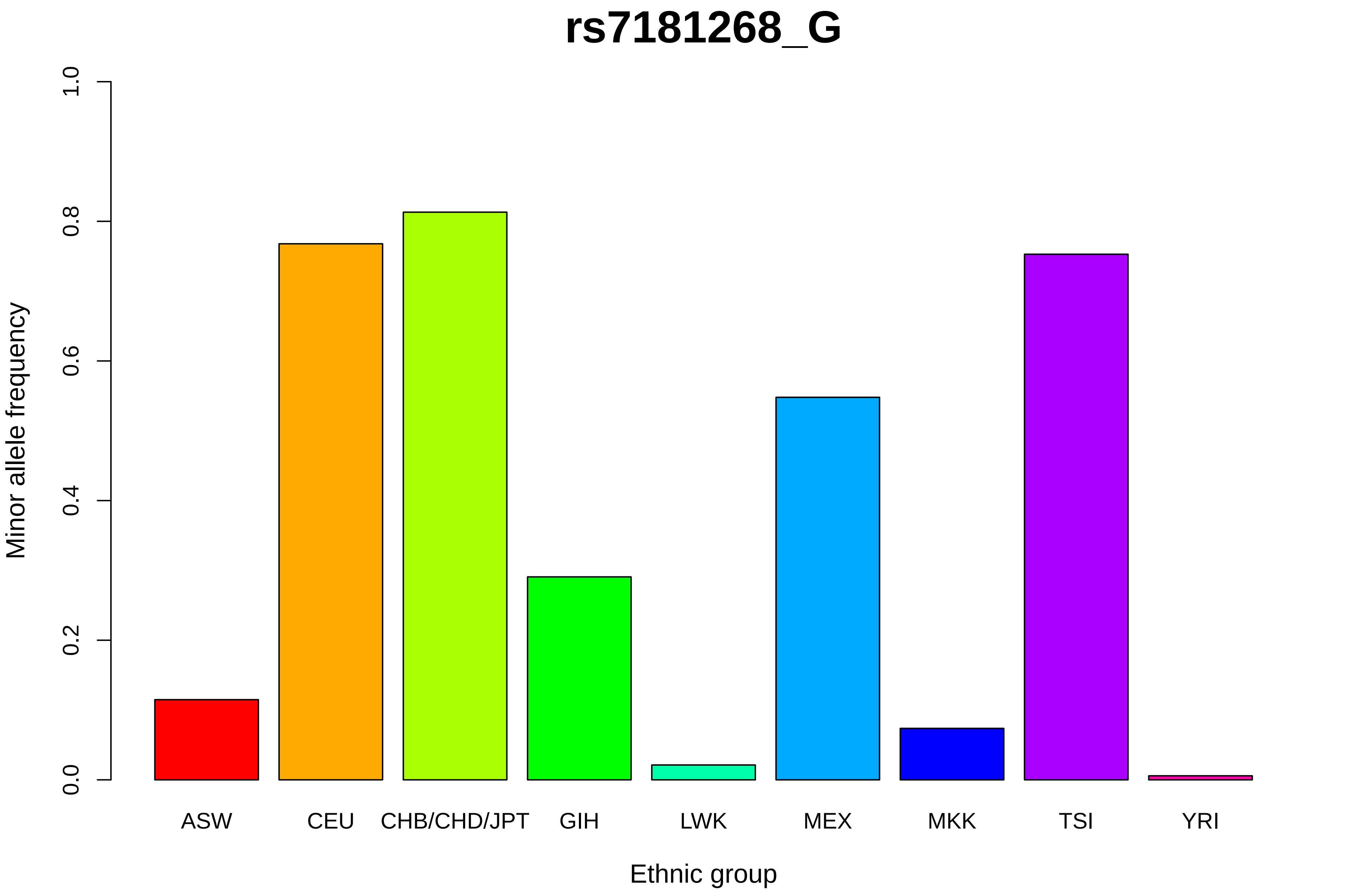
rs7181268_G
Minor allele frequency
0.0
0.2
0.4
0.6
0.8
1.0
ASW
CEU
CHB/CHD/JPT
GIH
LWK
MEX
MKK
TSI
YRI
Ethnic group

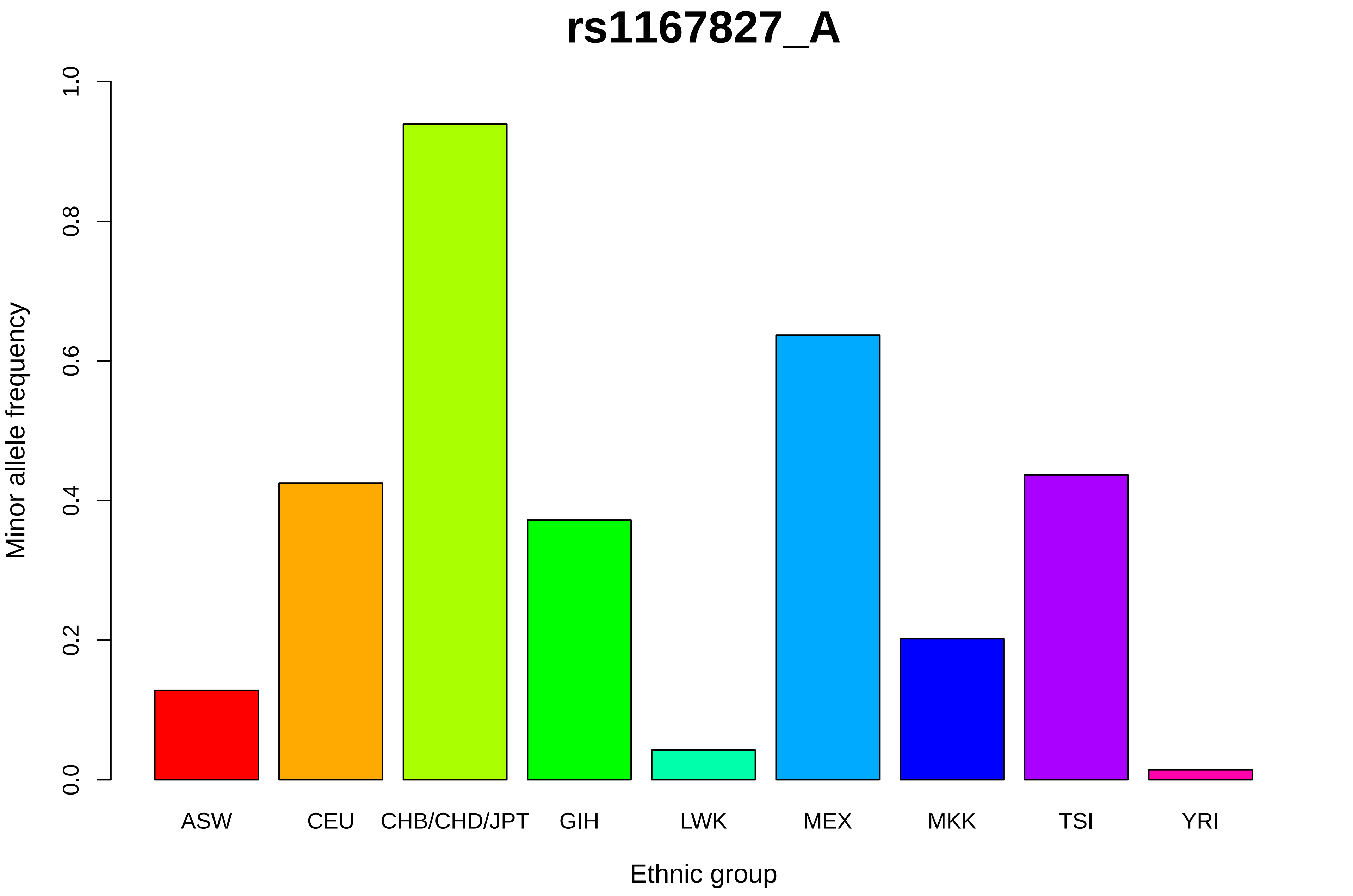

rs1167827_A
Minor allele frequency
0.0
0.2
0.4
0.6
0.8
1.0
ASW
CEU
CHB/CHD/JPT
GIH
LWK
MEX
MKK
TSI
YRI
Ethnic group

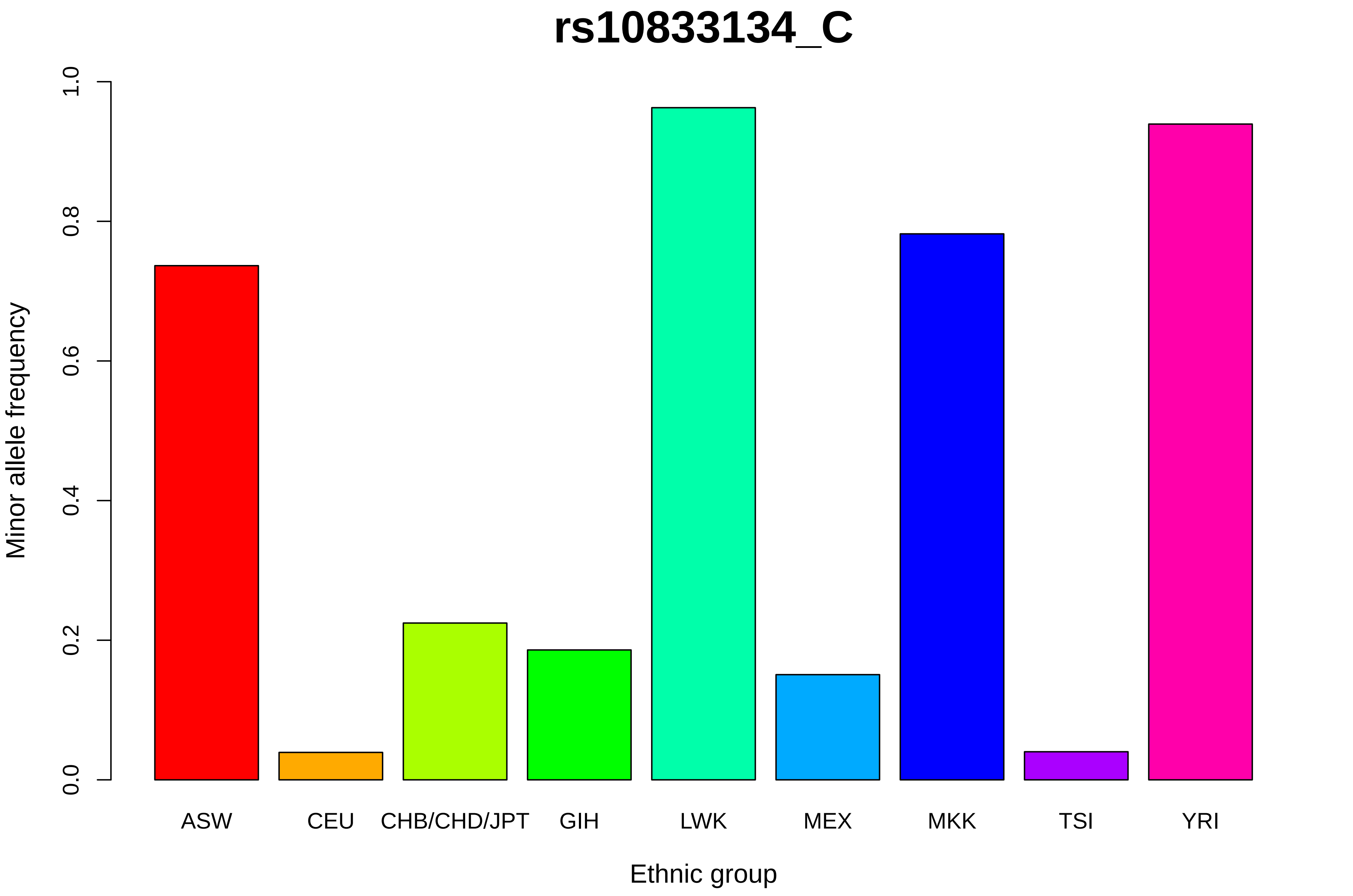
rs10833134_C
Minor allele frequency
0.0
0.2
0.4
0.6
0.8
1.0
ASW
CEU
CHB/CHD/JPT
GIH
LWK
MEX
MKK
TSI
YRI
Ethnic group

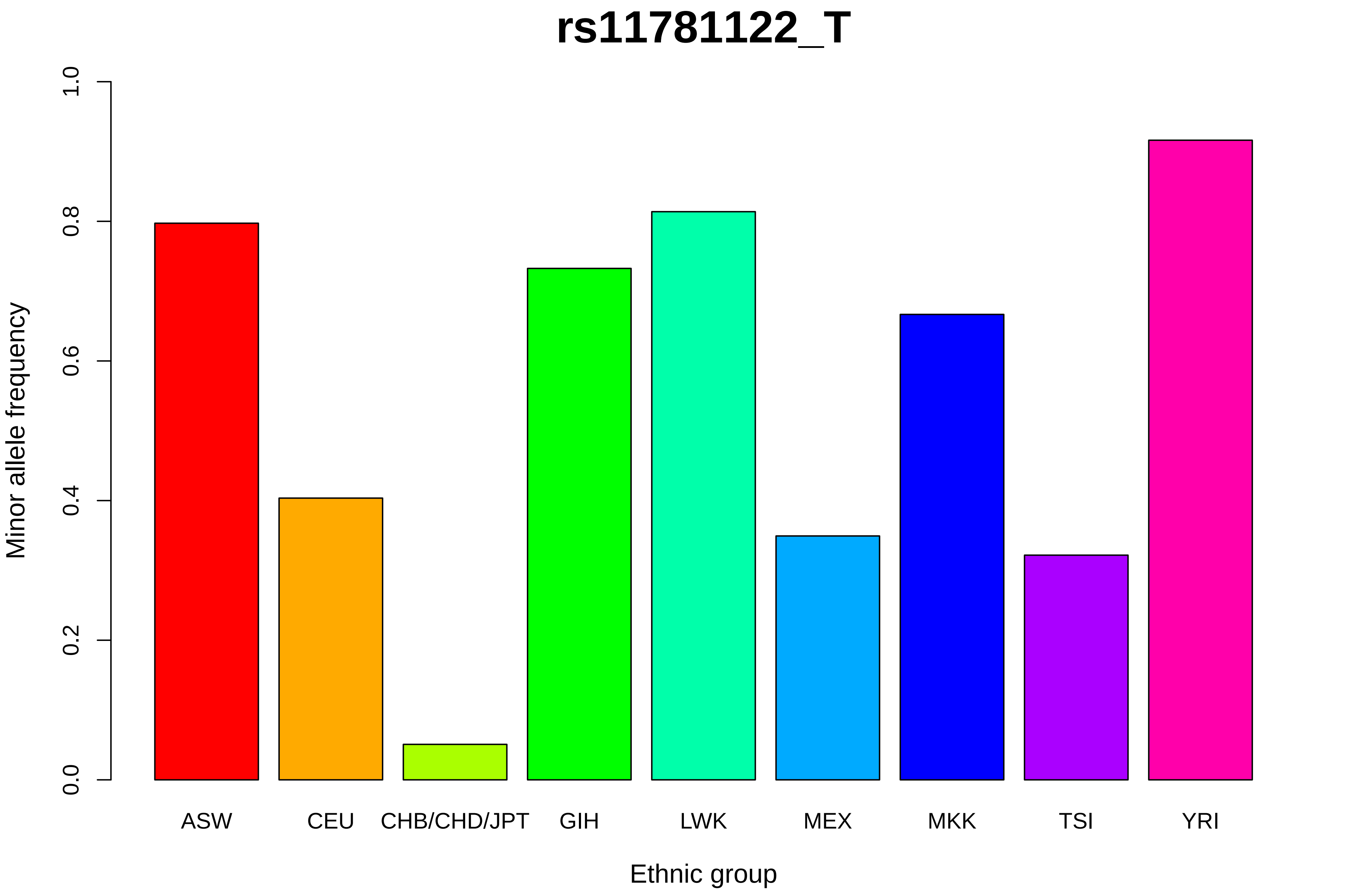
rs11781122_T
Minor allele frequency
0.0
0.2
0.4
0.6
0.8
1.0
ASW
CEU
CHB/CHD/JPT
GIH
LWK
MEX
MKK
TSI
YRI
Ethnic group

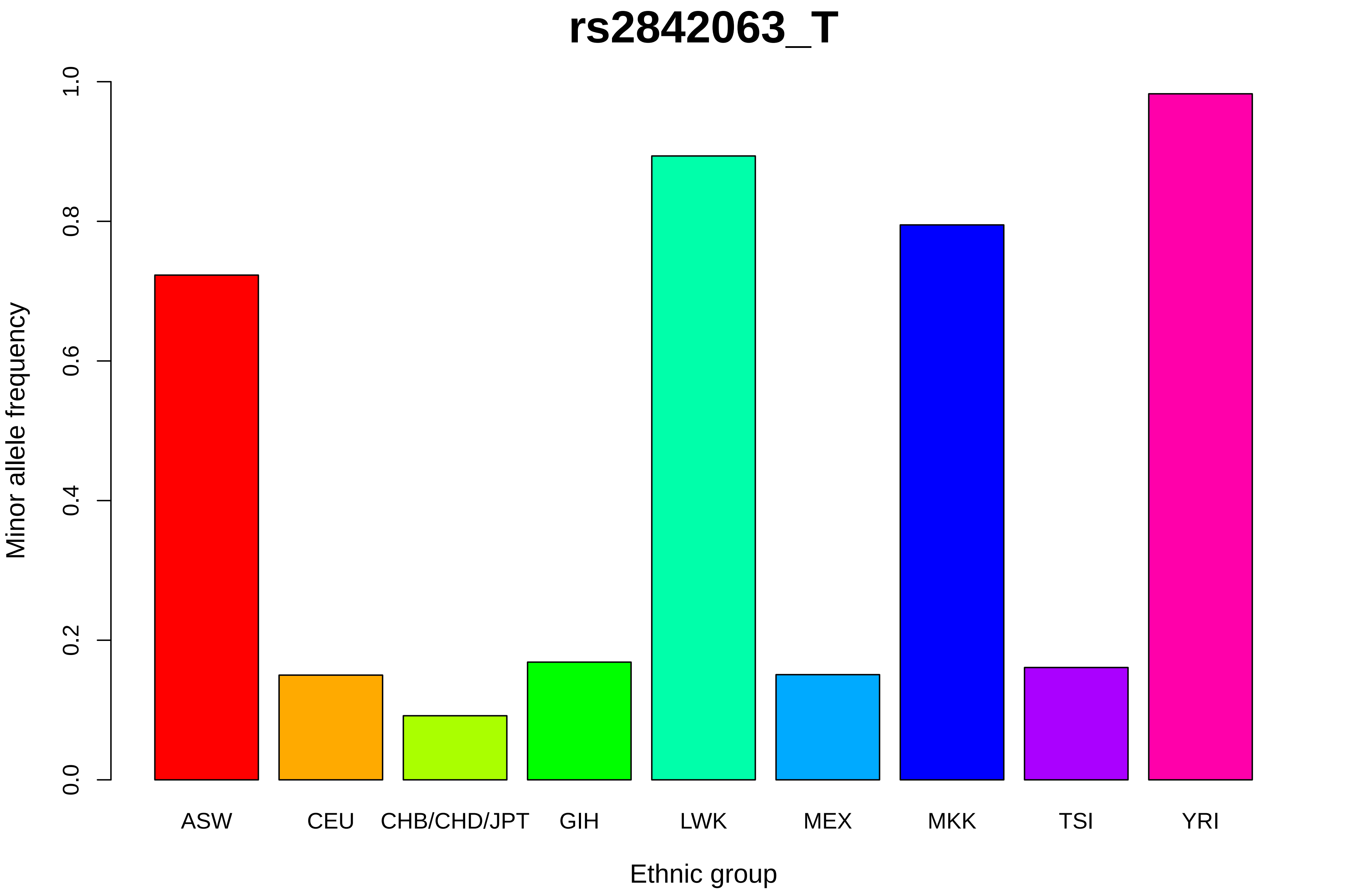

rs2842063_T
Minor allele frequency
0.0
0.2
0.4
0.6
0.8
1.0
ASW
CEU
CHB/CHD/JPT
GIH
LWK
MEX
MKK
TSI
YRI
Ethnic group

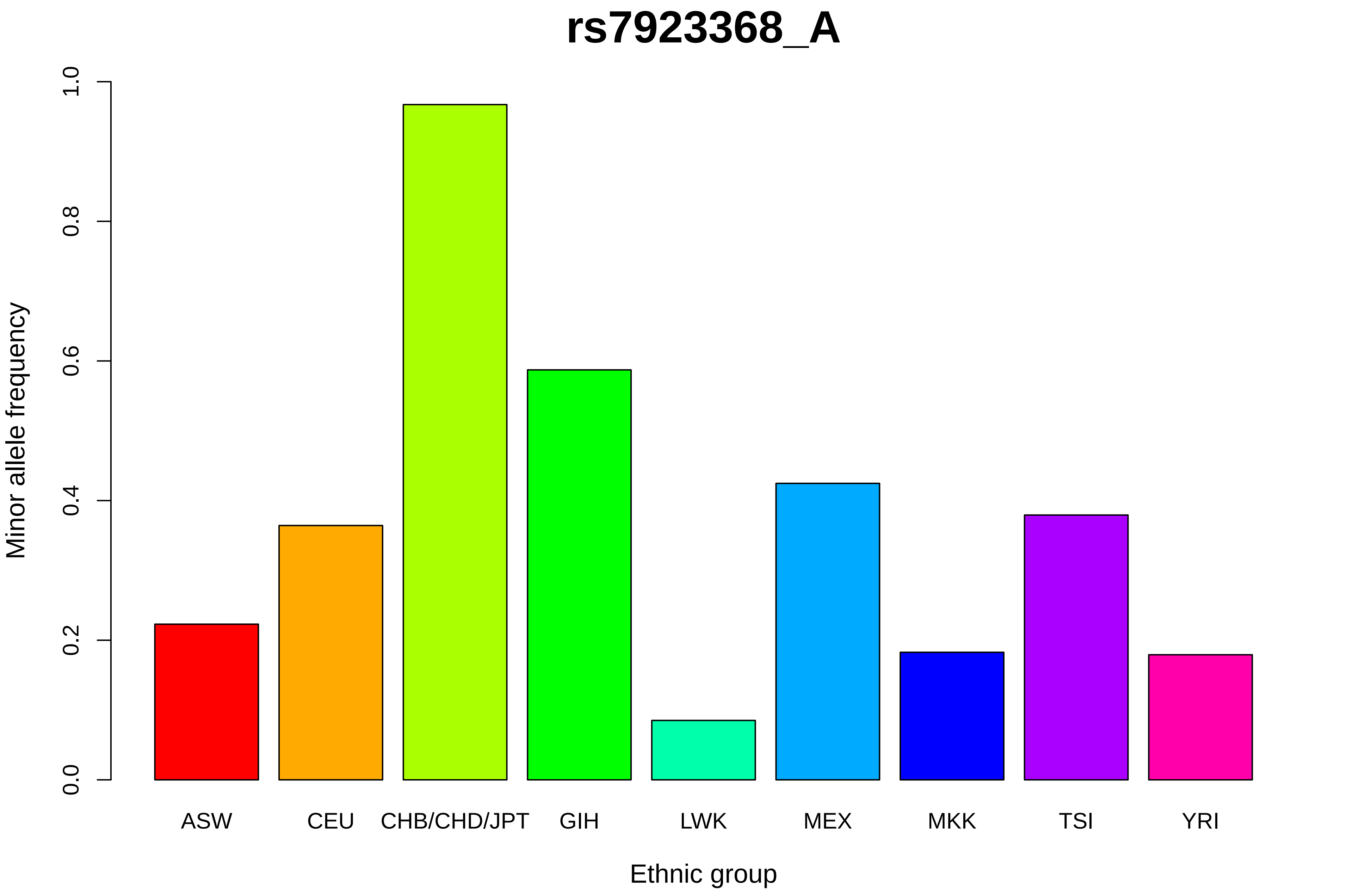
rs7923368_A
Minor allele frequency
0.0
0.2
0.4
0.6
0.8
1.0
ASW
CEU
CHB/CHD/JPT
GIH
LWK
MEX
MKK
TSI
YRI
Ethnic group

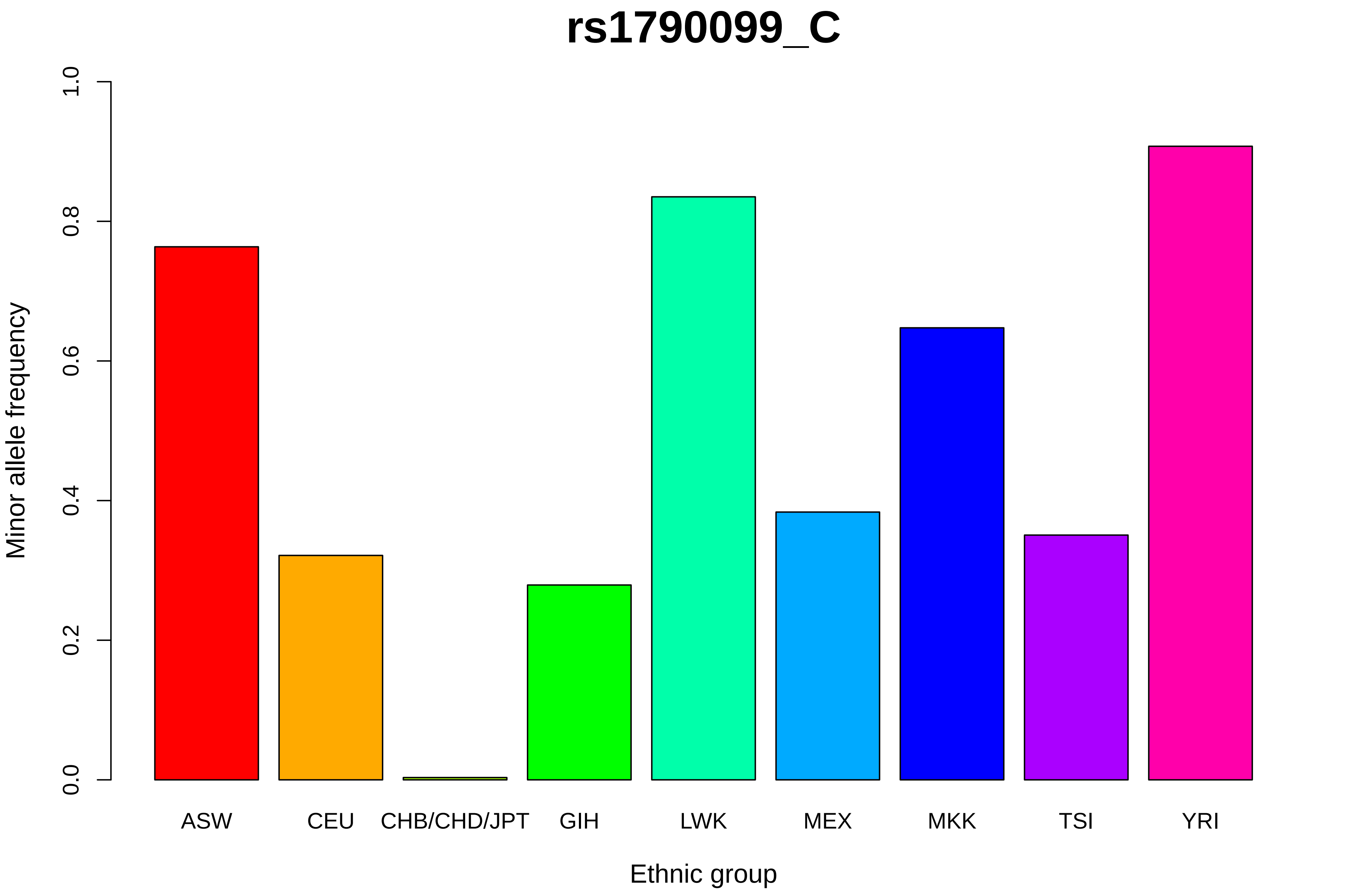
rs1790099_C
Minor allele frequency
0.0
0.2
0.4
0.6
0.8
1.0
ASW
CEU
CHB/CHD/JPT
GIH
LWK
MEX
MKK
TSI
YRI
Ethnic group

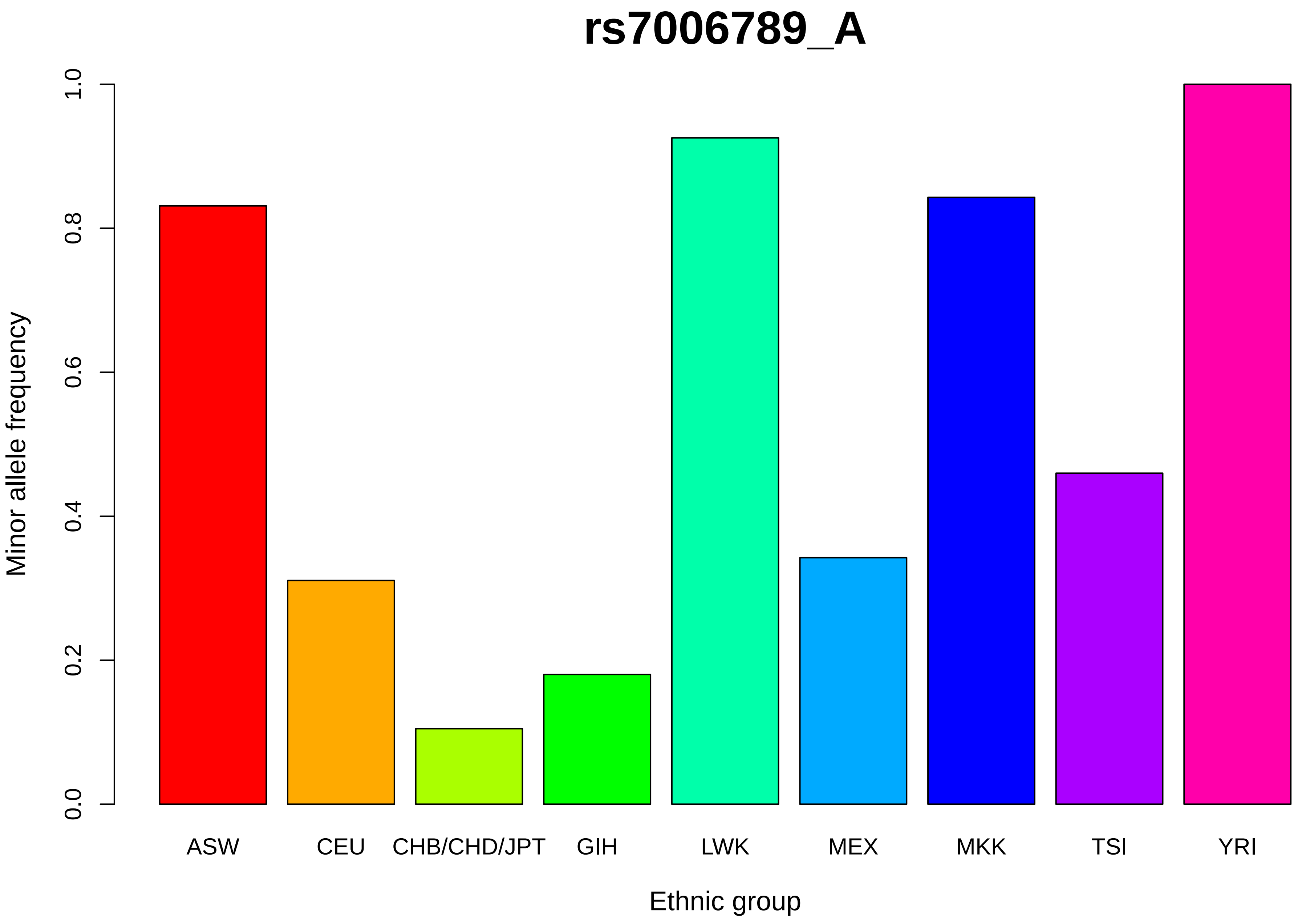

rs7006789_A
Minor allele frequency
0.0
0.2
0.4
0.6
0.8
1.0
ASW
CEU
CHB/CHD/JPT
GIH
LWK
MEX
MKK
TSI
YRI
Ethnic group

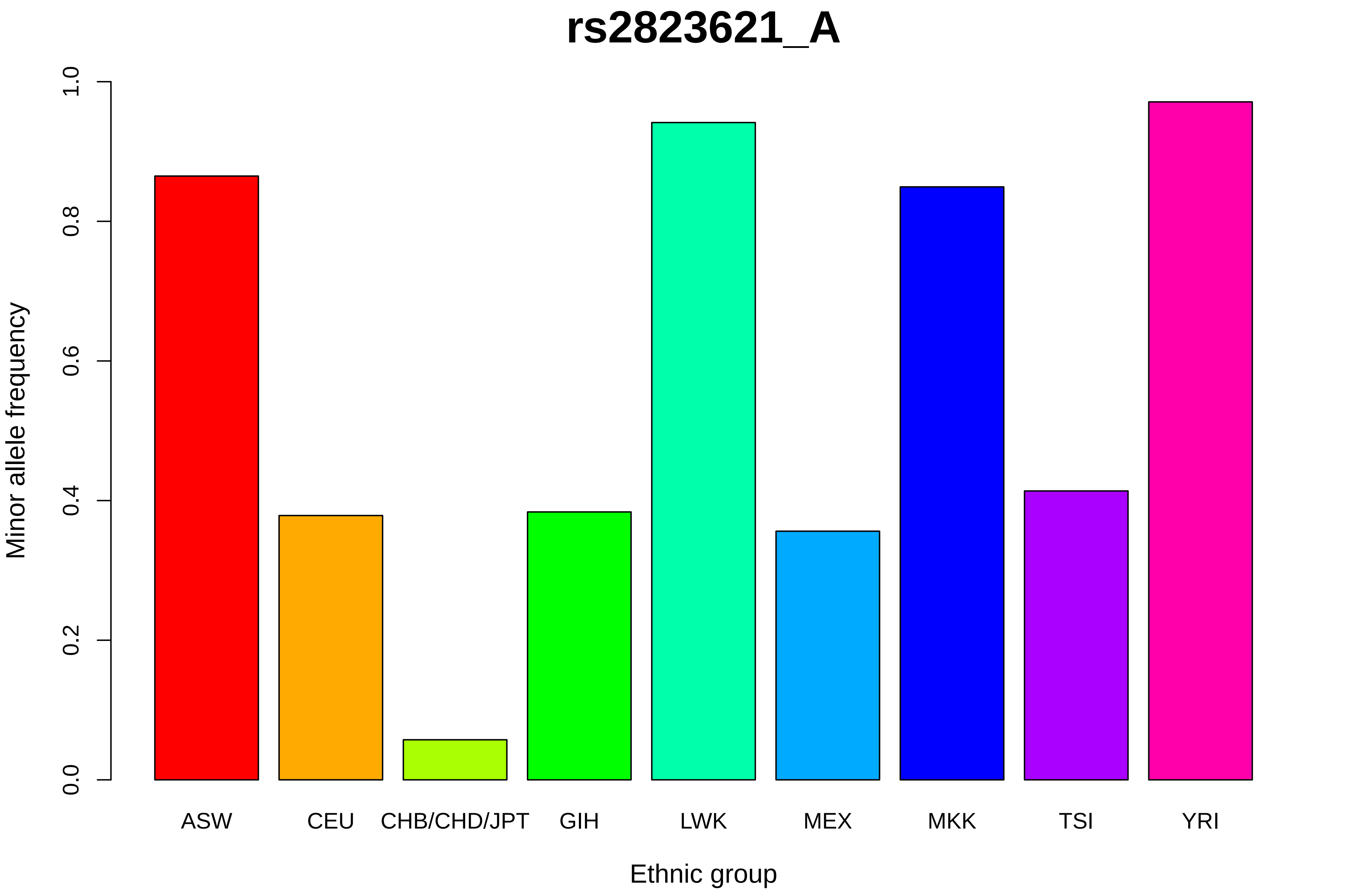
rs2823621_A
Minor allele frequency
0.0
0.2
0.4
0.6
0.8
1.0
ASW
CEU
CHB/CHD/JPT
GIH
LWK
MEX
MKK
TSI
YRI
Ethnic group

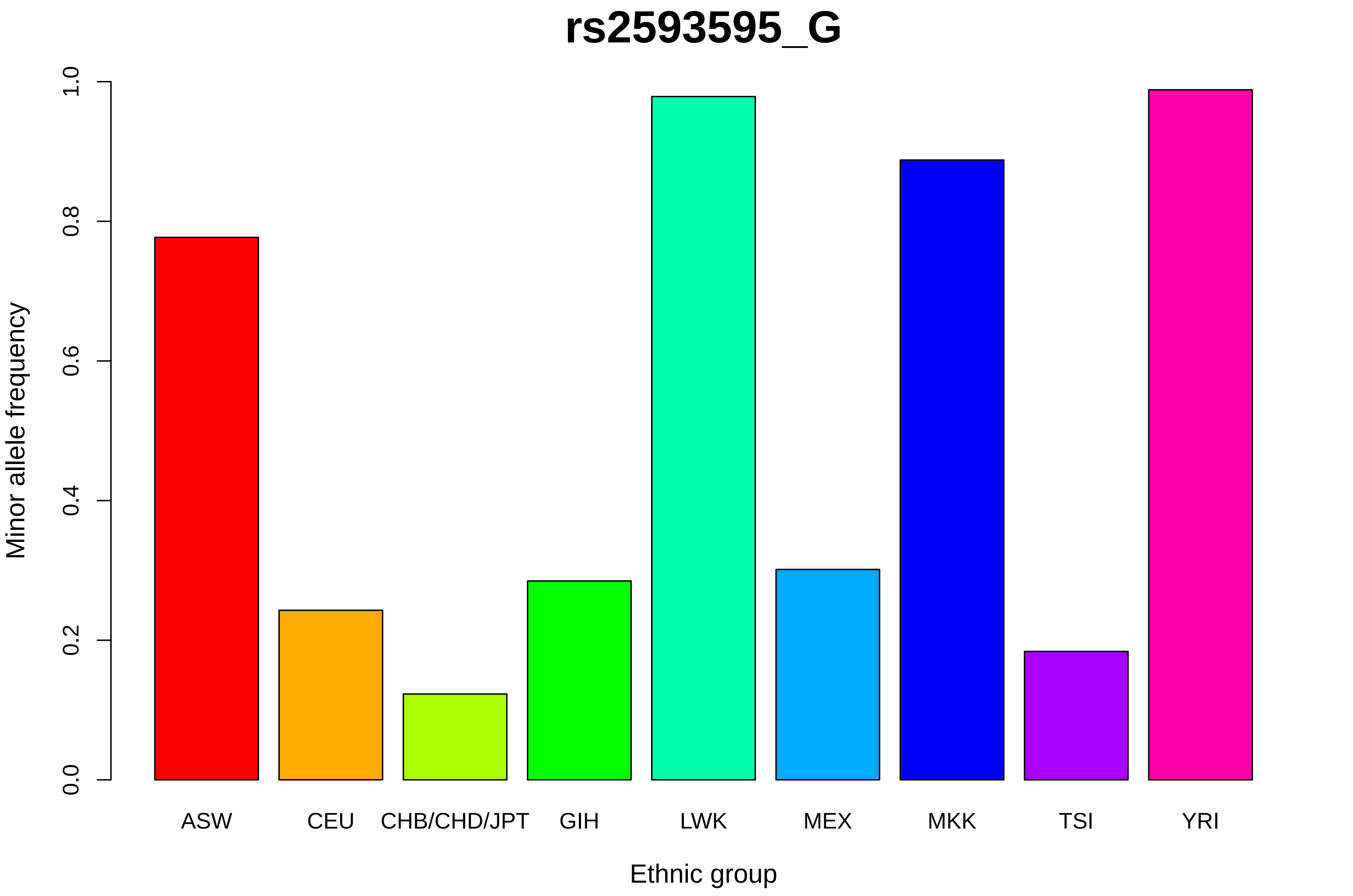

rs2593595_G
Minor allele frequency
0.0
0.2
0.4
0.6
0.8
1.0
ASW
CEU
CHB/CHD/JPT
GIH
LWK
MEX
MKK
TSI
YRI
Ethnic group

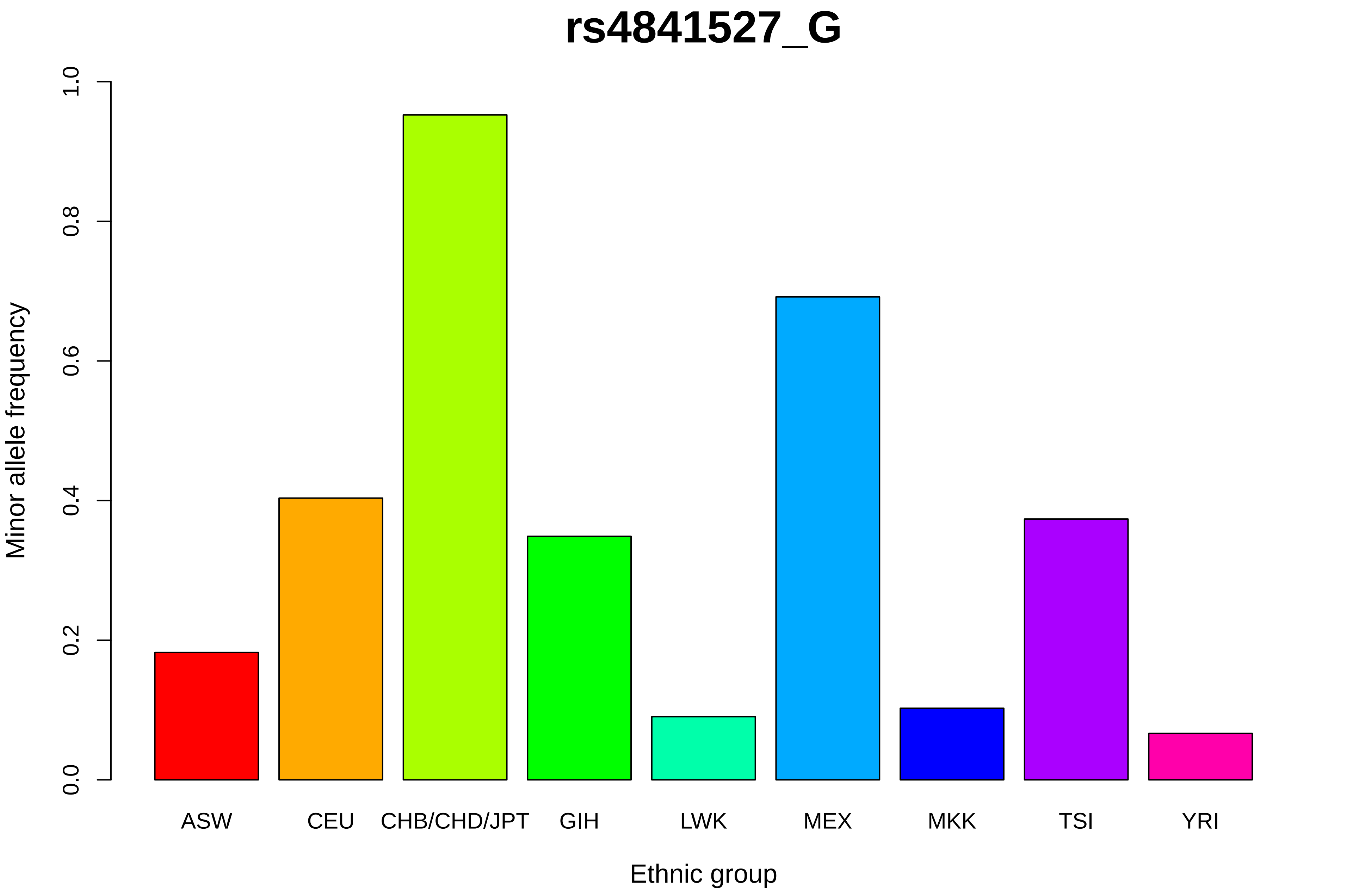
rs4841527_G
Minor allele frequency
0.0
0.2
0.4
0.6
0.8
1.0
ASW
CEU
CHB/CHD/JPT
GIH
LWK
MEX
MKK
TSI
YRI
Ethnic group

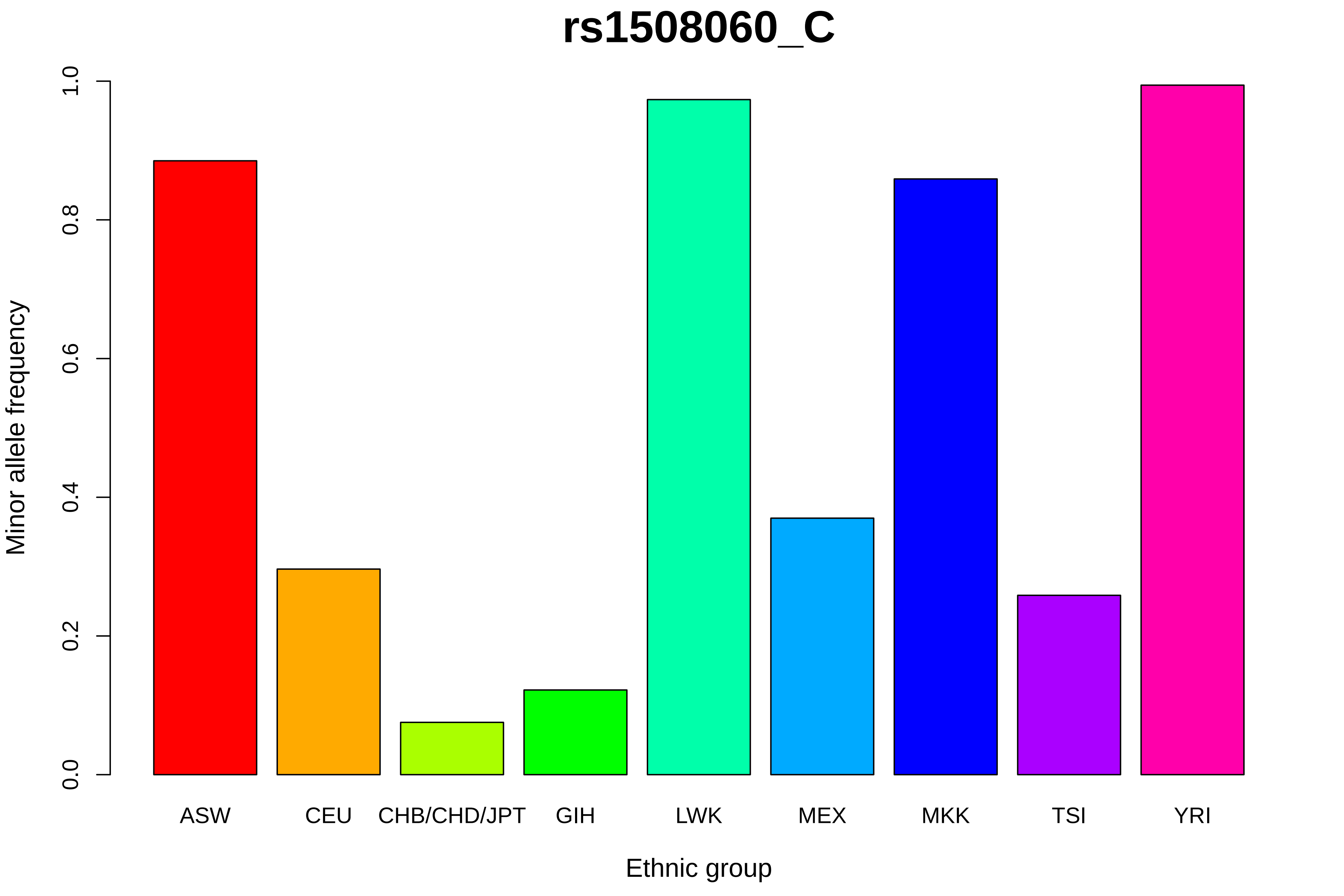
rs1508060_C
Minor allele frequency
0.0
0.2
0.4
0.6
0.8
1.0
ASW
CEU
CHB/CHD/JPT
GIH
LWK
MEX
MKK
TSI
YRI
Ethnic group

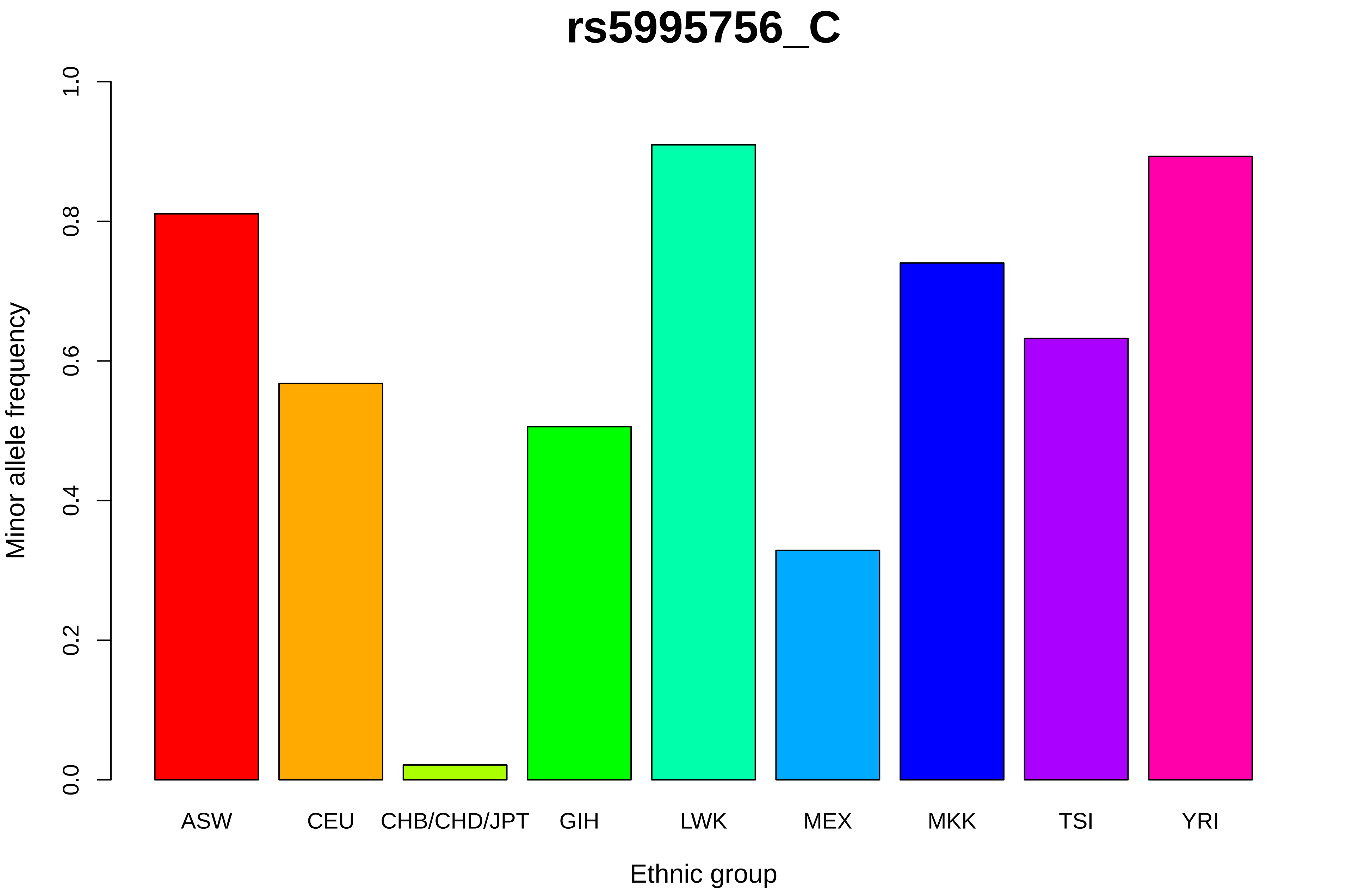

rs5995756_C
Minor allele frequency
0.0
0.2
0.4
0.6
0.8
1.0
ASW
CEU
CHB/CHD/JPT
GIH
LWK
MEX
MKK
TSI
YRI
Ethnic group

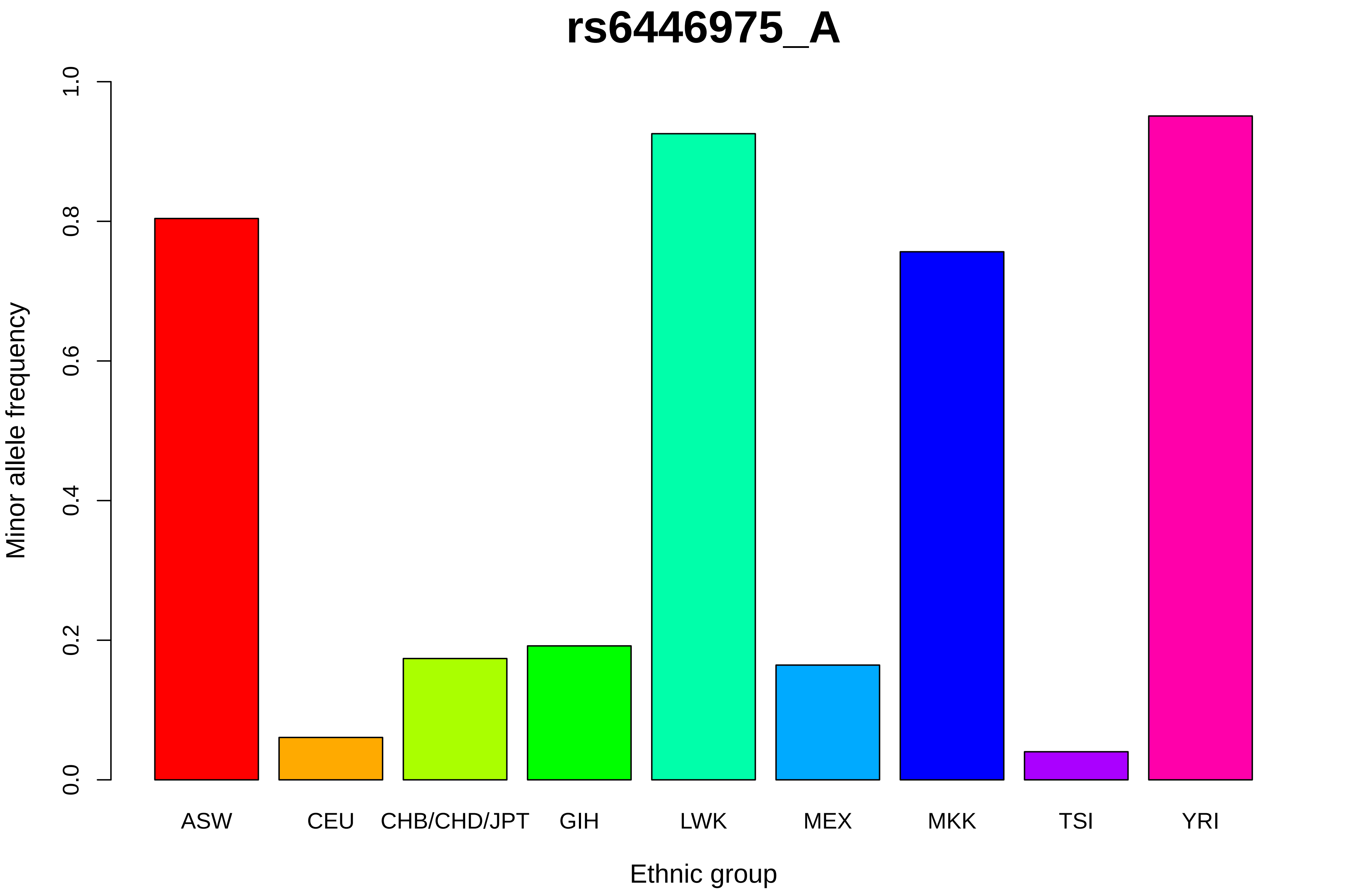
rs6446975_A
Minor allele frequency
0.0
0.2
0.4
0.6
0.8
1.0
ASW
CEU
CHB/CHD/JPT
GIH
LWK
MEX
MKK
TSI
YRI
Ethnic group

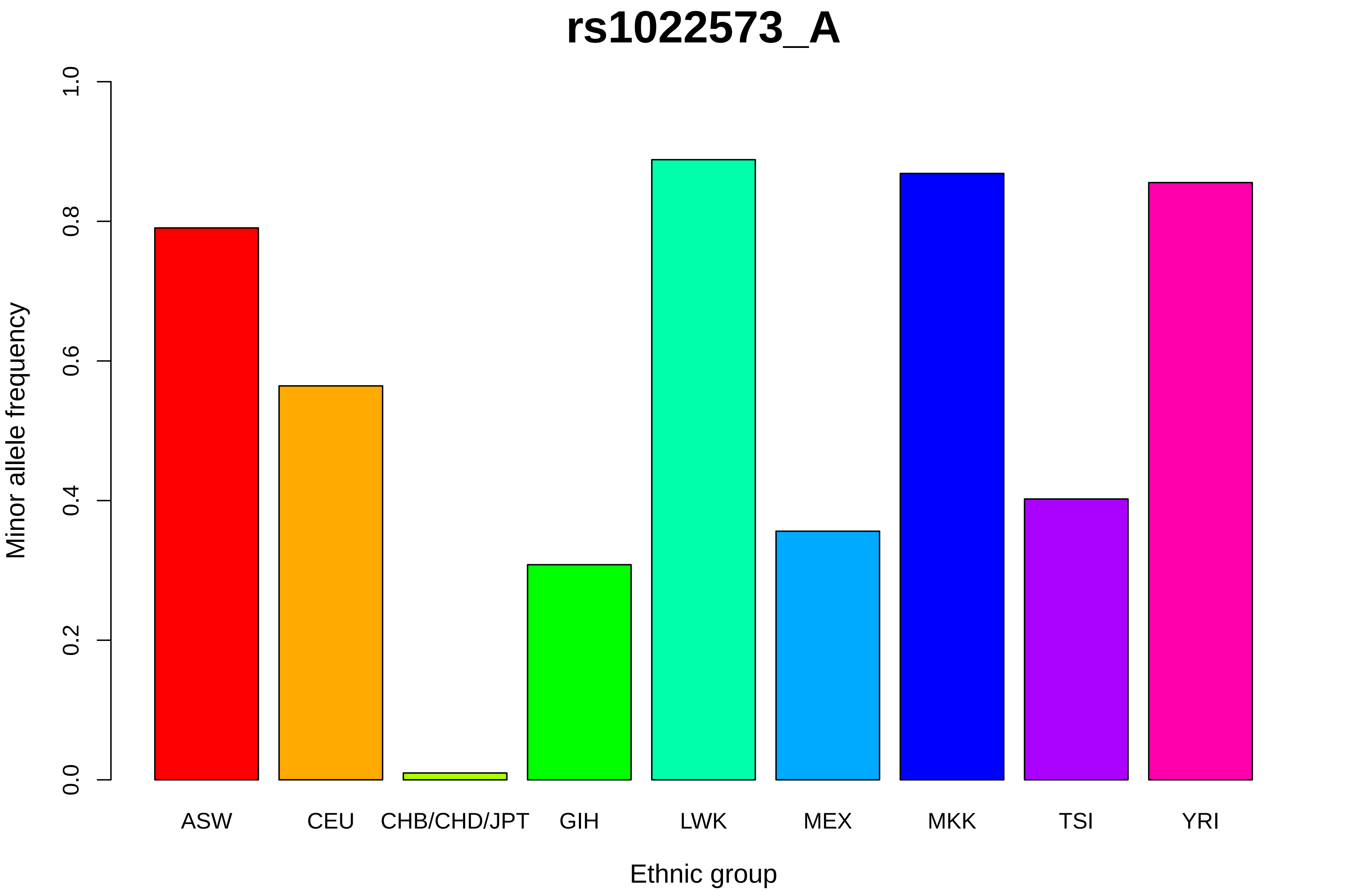
rs1022573_A
Minor allele frequency
0.0
0.2
0.4
0.6
0.8
1.0
ASW
CEU
CHB/CHD/JPT
GIH
LWK
MEX
MKK
TSI
YRI
Ethnic group

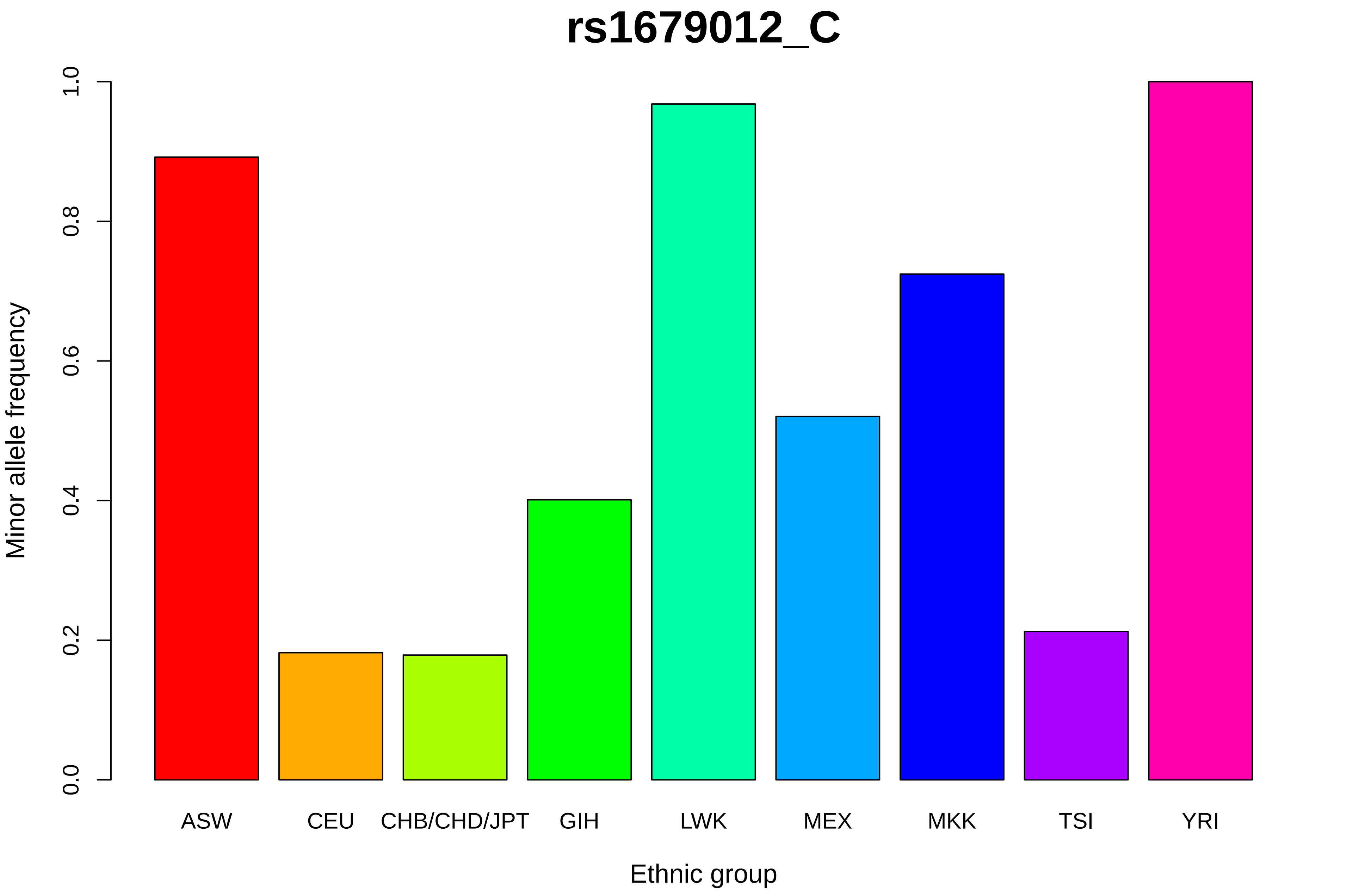
rs1679012_C
Minor allele frequency
0.0
0.2
0.4
0.6
0.8
1.0
ASW
CEU
CHB/CHD/JPT
GIH
LWK
MEX
MKK
TSI
YRI
Ethnic group

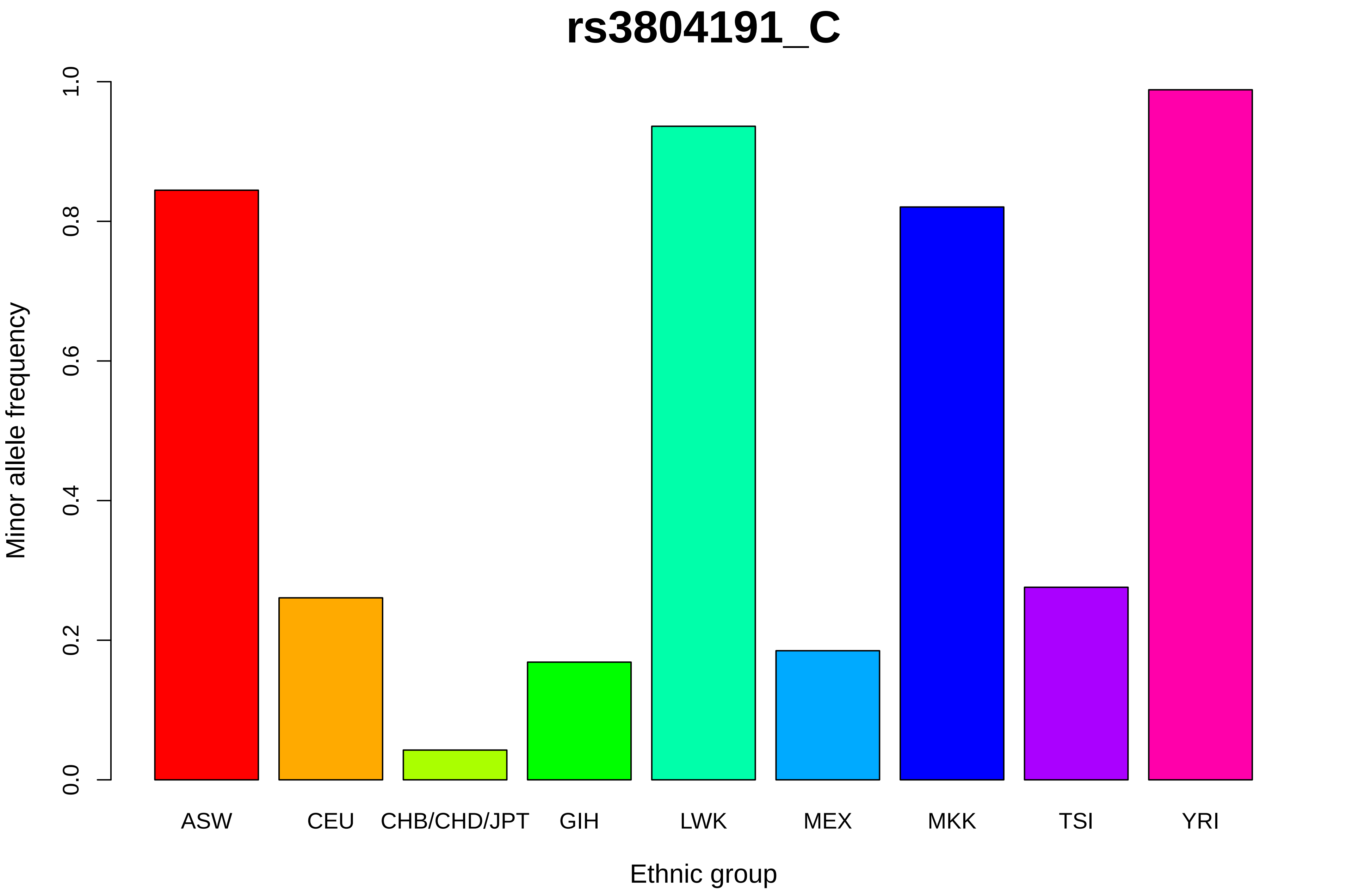
rs3804191_C
Minor allele frequency
0.0
0.2
0.4
0.6
0.8
1.0
ASW
CEU
CHB/CHD/JPT
GIH
LWK
MEX
MKK
TSI
YRI
Ethnic group

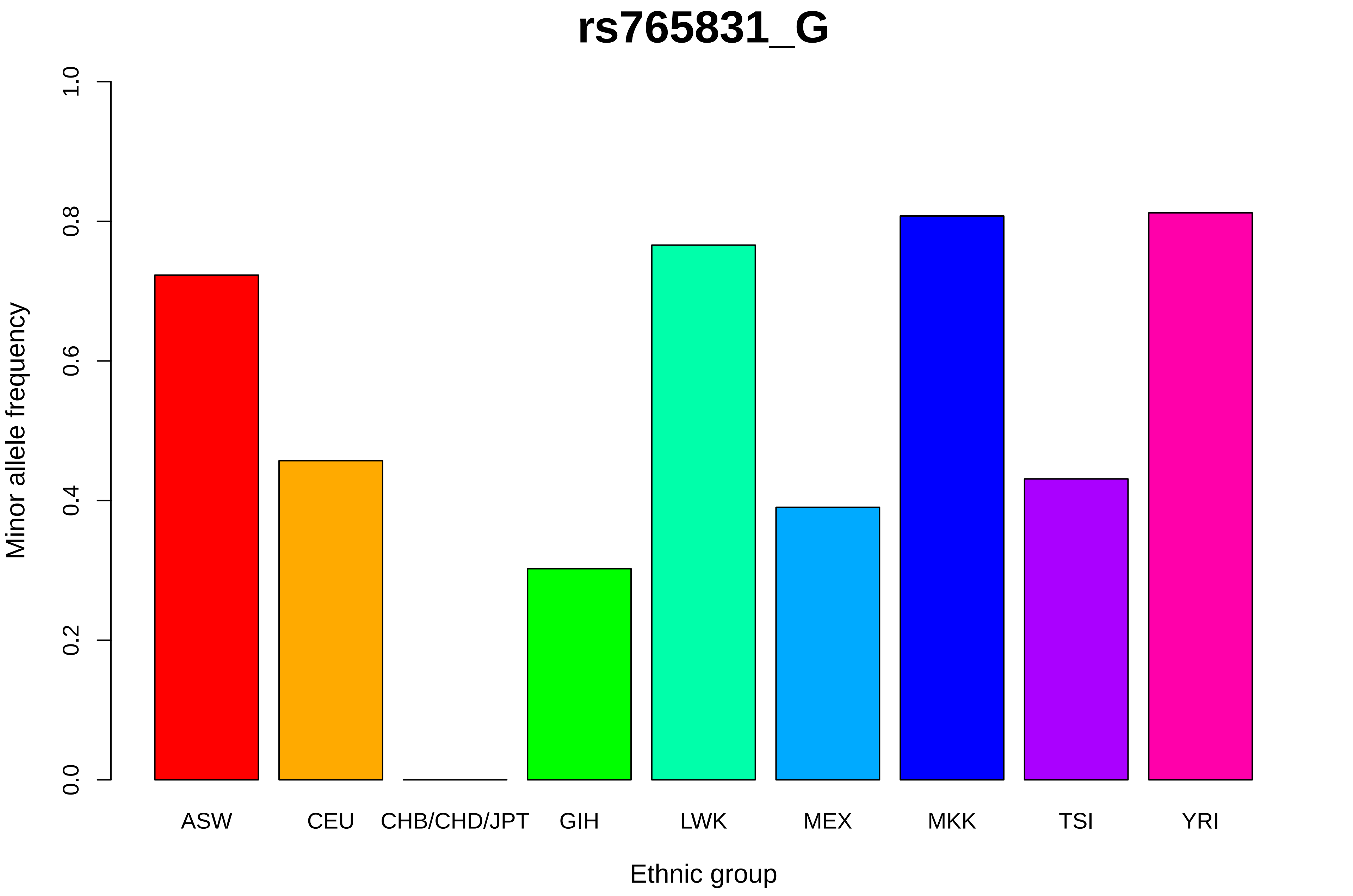

rs765831_G
Minor allele frequency
0.0
0.2
0.4
0.6
0.8
1.0
ASW
CEU
CHB/CHD/JPT
GIH
LWK
MEX
MKK
TSI
YRI
Ethnic group

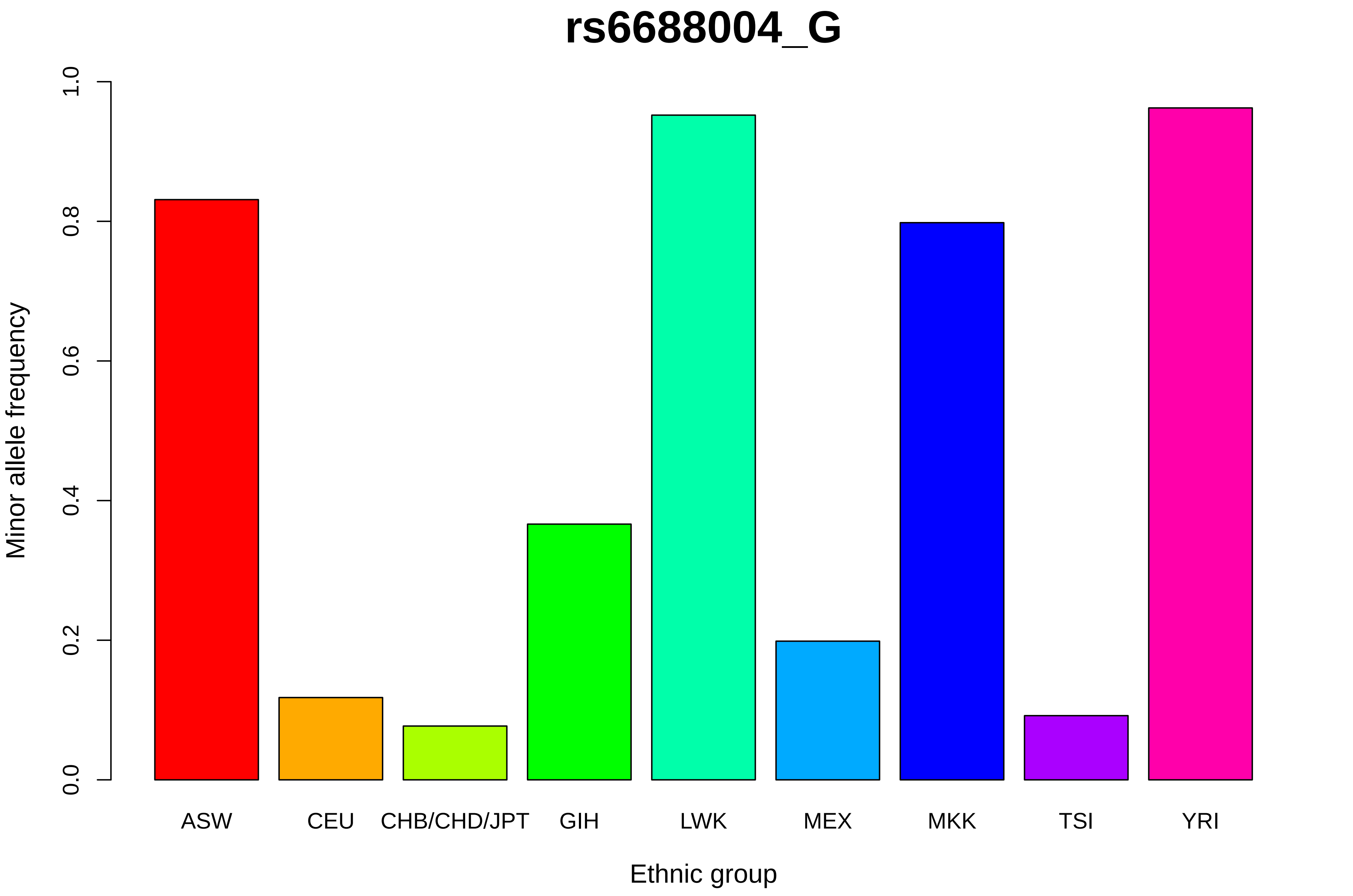
rs6688004_G
Minor allele frequency
0.0
0.2
0.4
0.6
0.8
1.0
ASW
CEU
CHB/CHD/JPT
GIH
LWK
MEX
MKK
TSI
YRI
Ethnic group

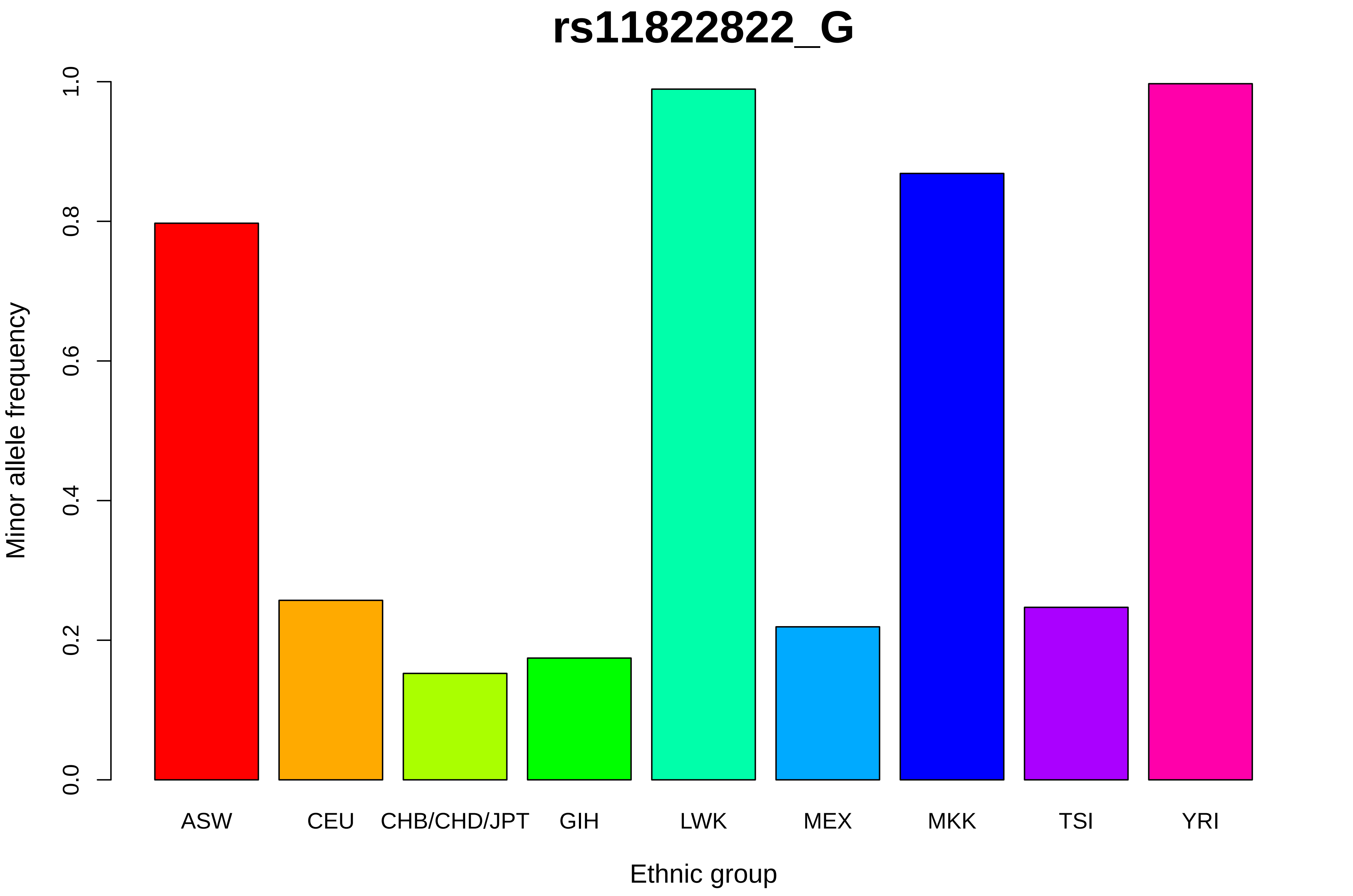
rs11822822_G
Minor allele frequency
0.0
0.2
0.4
0.6
0.8
1.0
ASW
CEU
CHB/CHD/JPT
GIH
LWK
MEX
MKK
TSI
YRI
Ethnic group

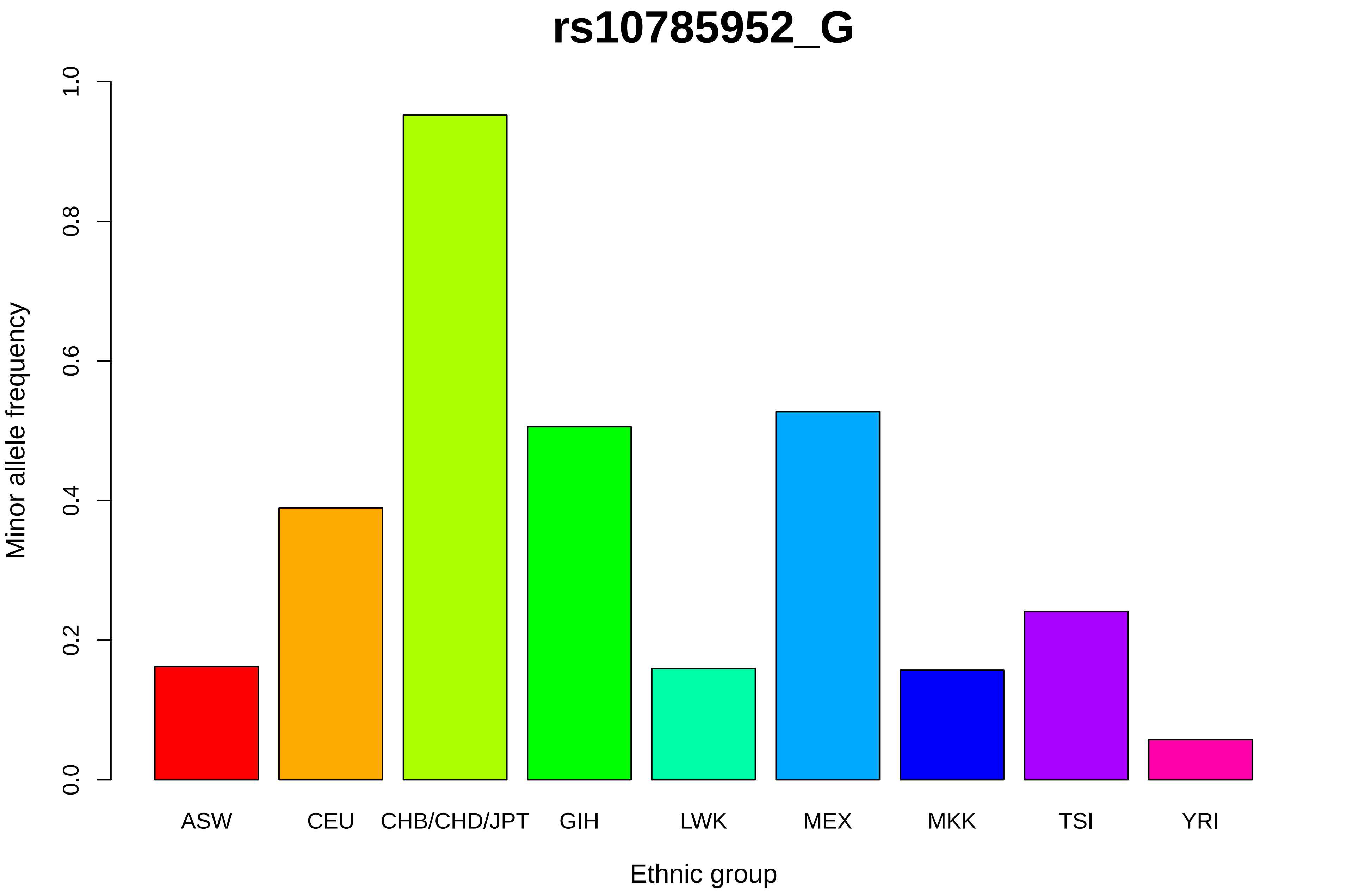
rs10785952_G
Minor allele frequency
0.0
0.2
0.4
0.6
0.8
1.0
ASW
CEU
CHB/CHD/JPT
GIH
LWK
MEX
MKK
TSI
YRI
Ethnic group

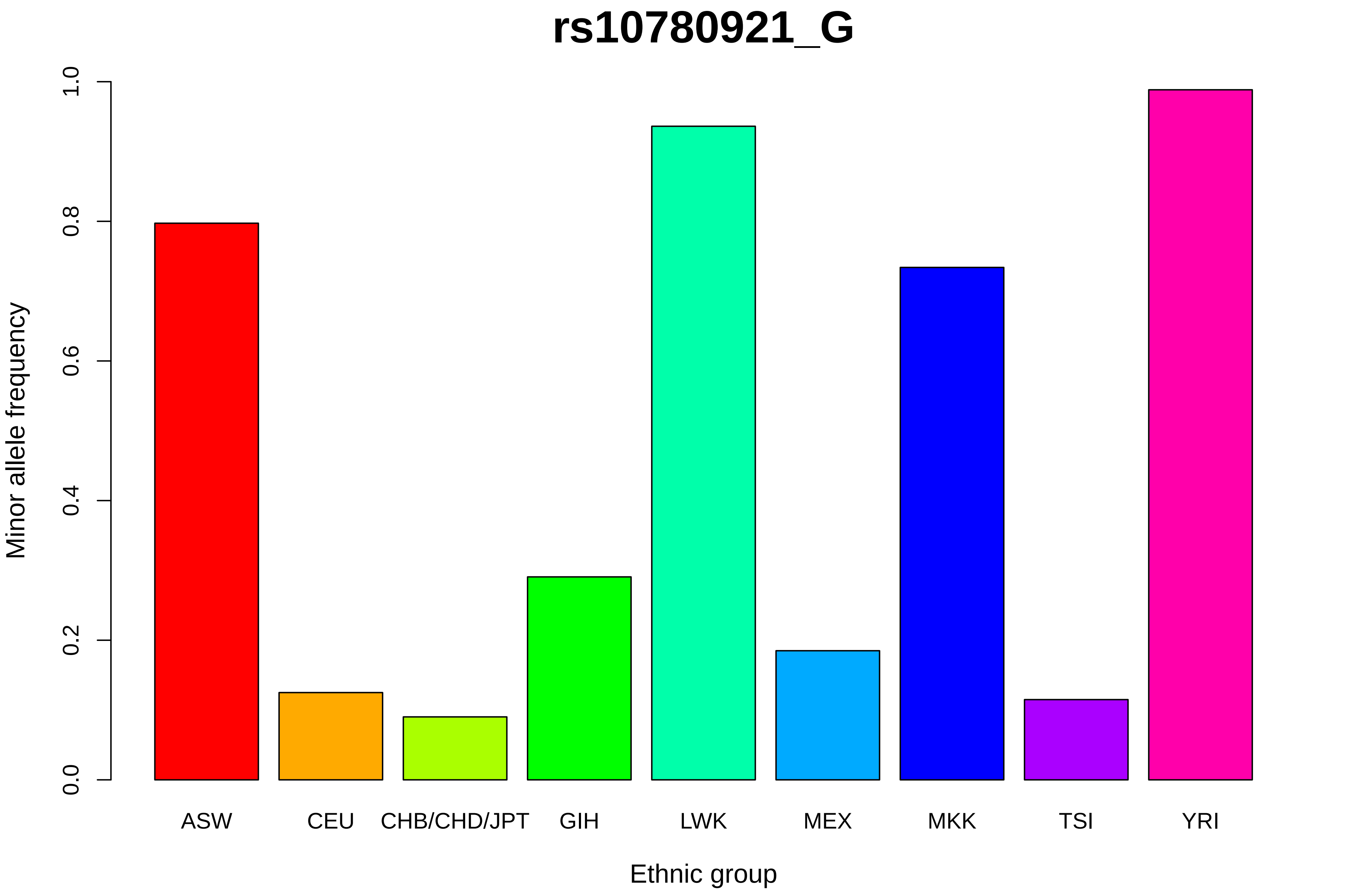
rs10780921_G
Minor allele frequency
0.0
0.2
0.4
0.6
0.8
1.0
ASW
CEU
CHB/CHD/JPT
GIH
LWK
MEX
MKK
TSI
YRI
Ethnic group

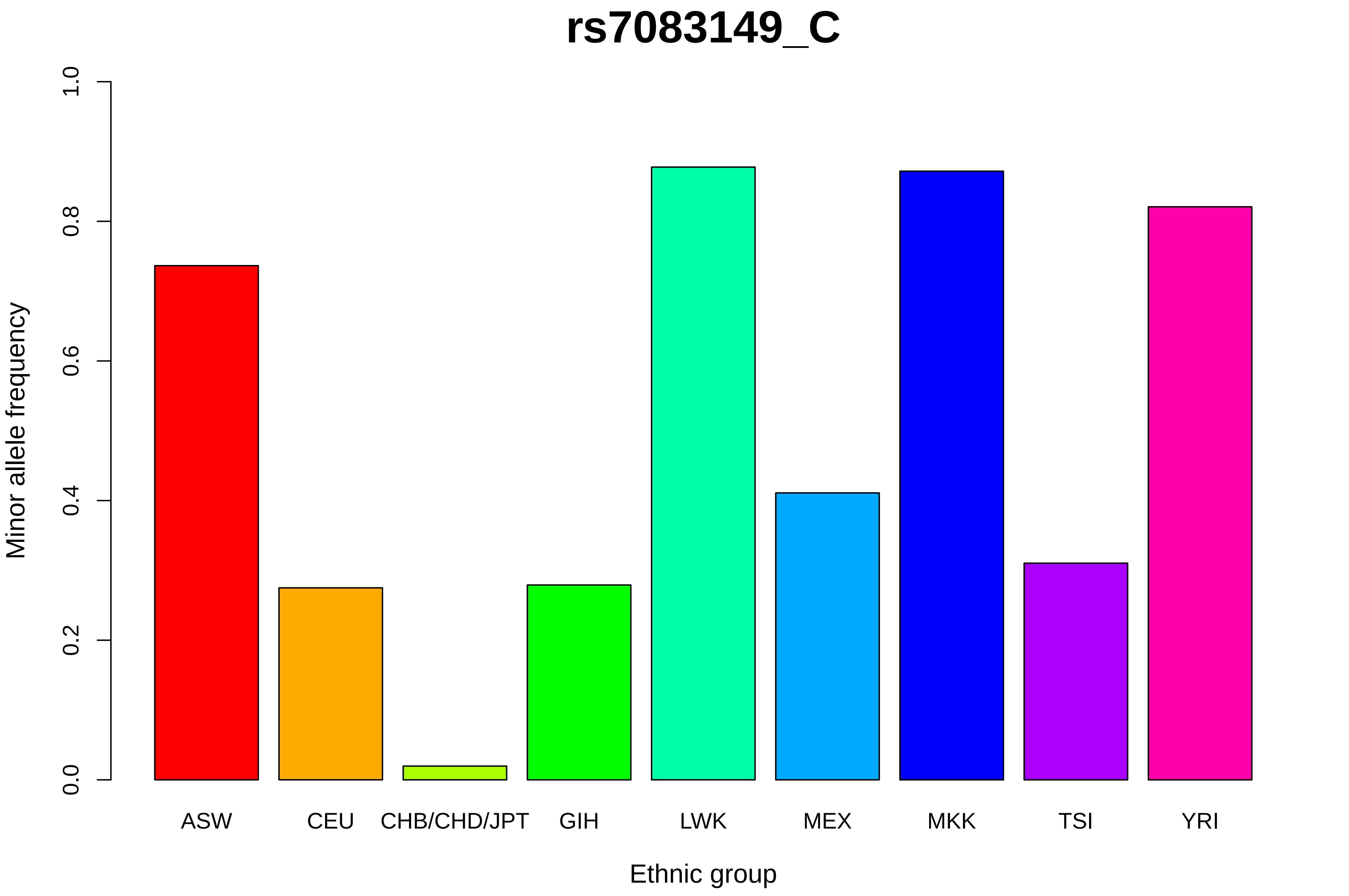

rs7083149_C
Minor allele frequency
0.0
0.2
0.4
0.6
0.8
1.0
ASW
CEU
CHB/CHD/JPT
GIH
LWK
MEX
MKK
TSI
YRI
Ethnic group

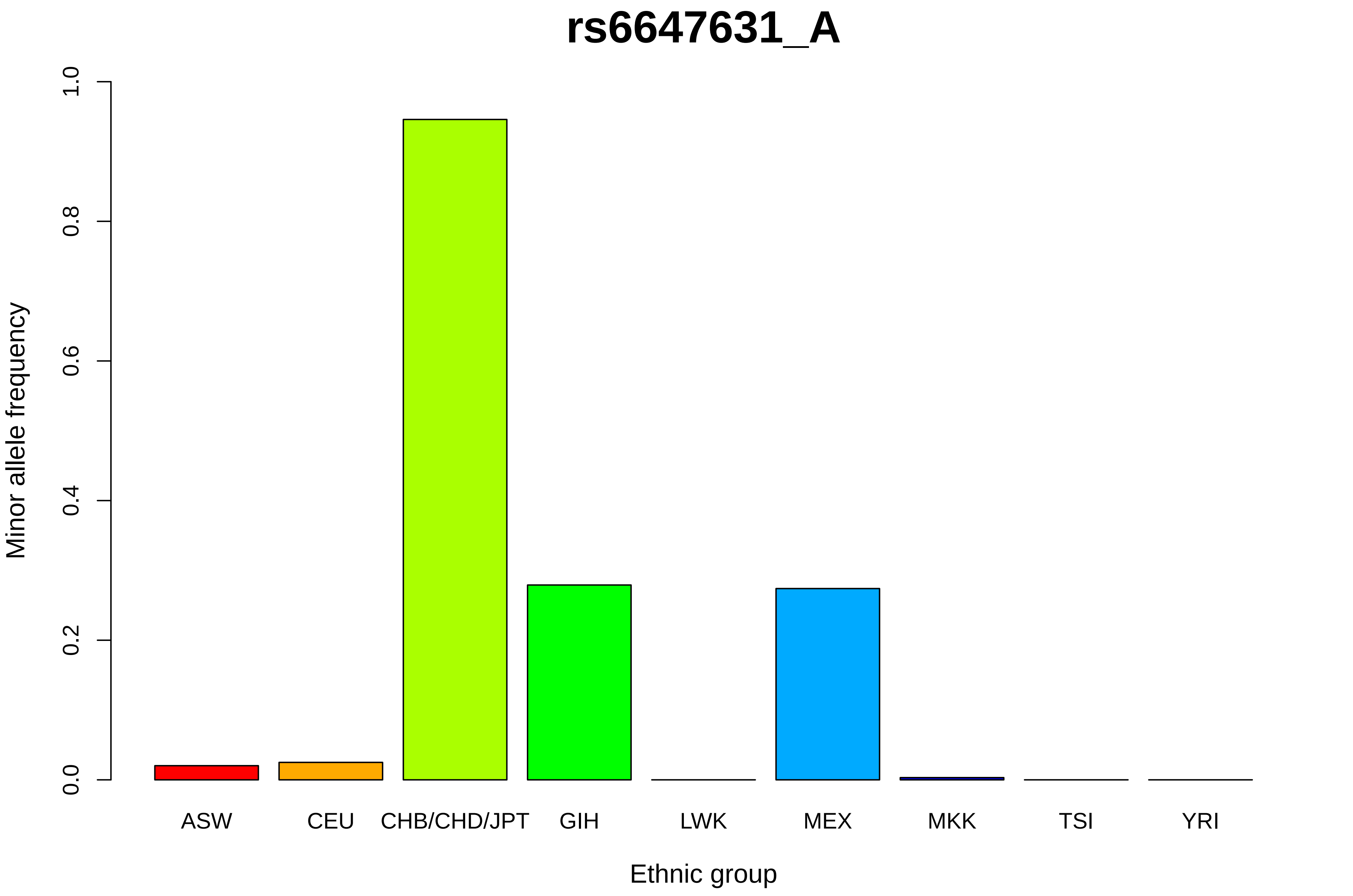
rs6647631_A
Minor allele frequency
0.0
0.2
0.4
0.6
0.8
1.0
ASW
CEU
CHB/CHD/JPT
GIH
LWK
MEX
MKK
TSI
YRI
Ethnic group

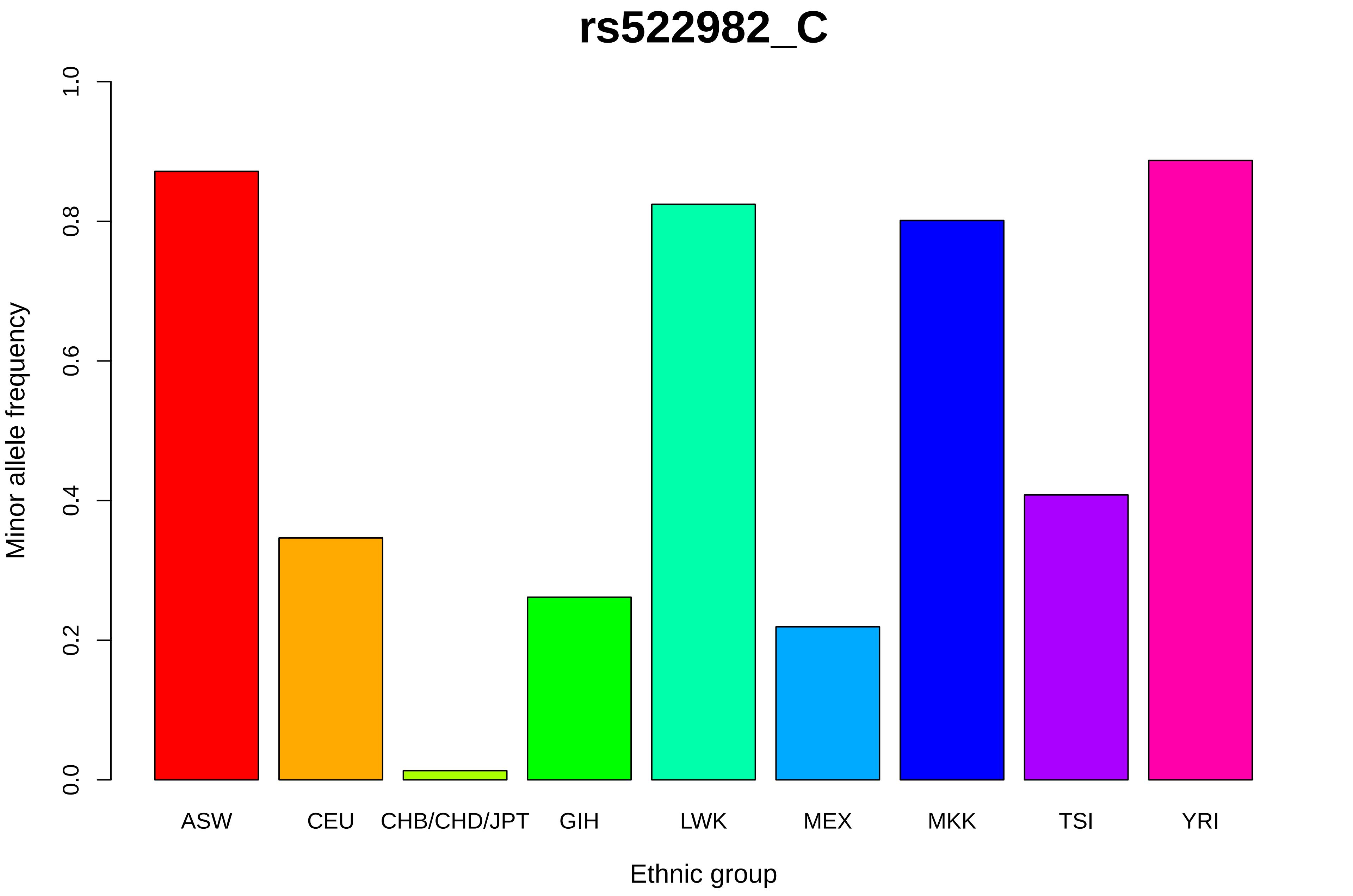

rs522982_C
Minor allele frequency
0.0
0.2
0.4
0.6
0.8
1.0
ASW
CEU
CHB/CHD/JPT
GIH
LWK
MEX
MKK
TSI
YRI
Ethnic group

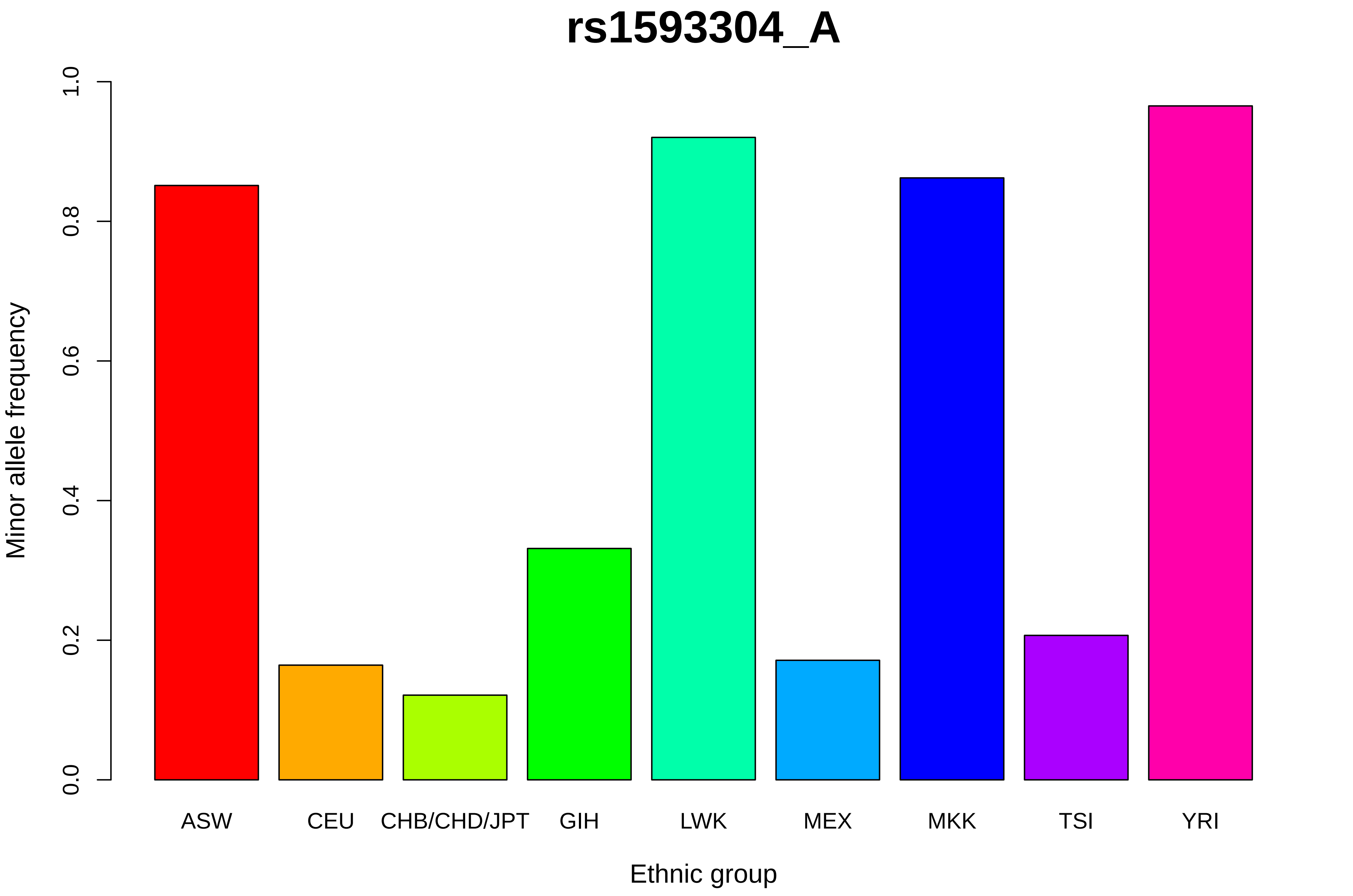

rs1593304_A
Minor allele frequency
0.0
0.2
0.4
0.6
0.8
1.0
ASW
CEU
CHB/CHD/JPT
GIH
LWK
MEX
MKK
TSI
YRI
Ethnic group

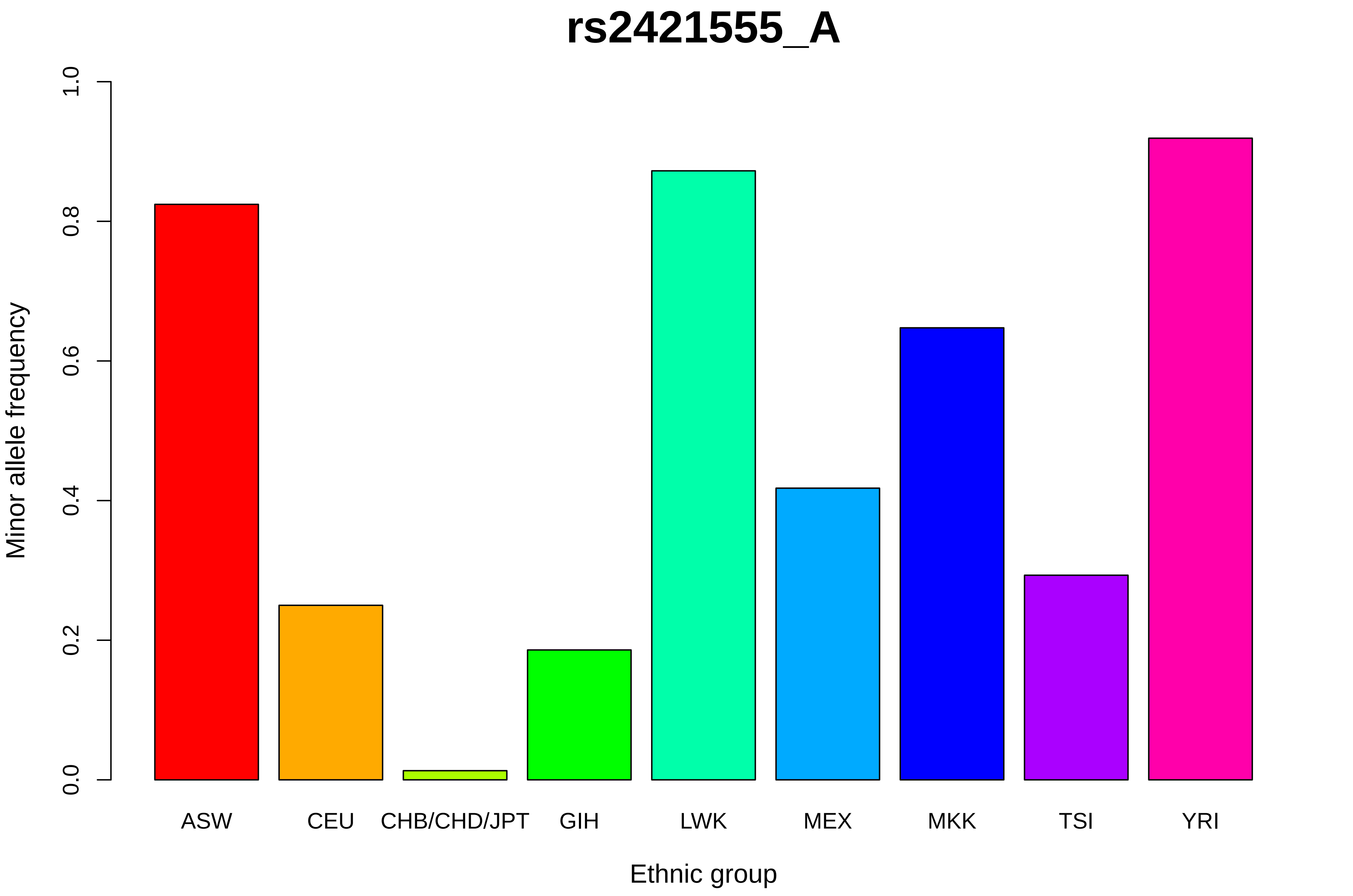
rs2421555_A
Minor allele frequency
0.0
0.2
0.4
0.6
0.8
1.0
ASW
CEU
CHB/CHD/JPT
GIH
LWK
MEX
MKK
TSI
YRI
Ethnic group

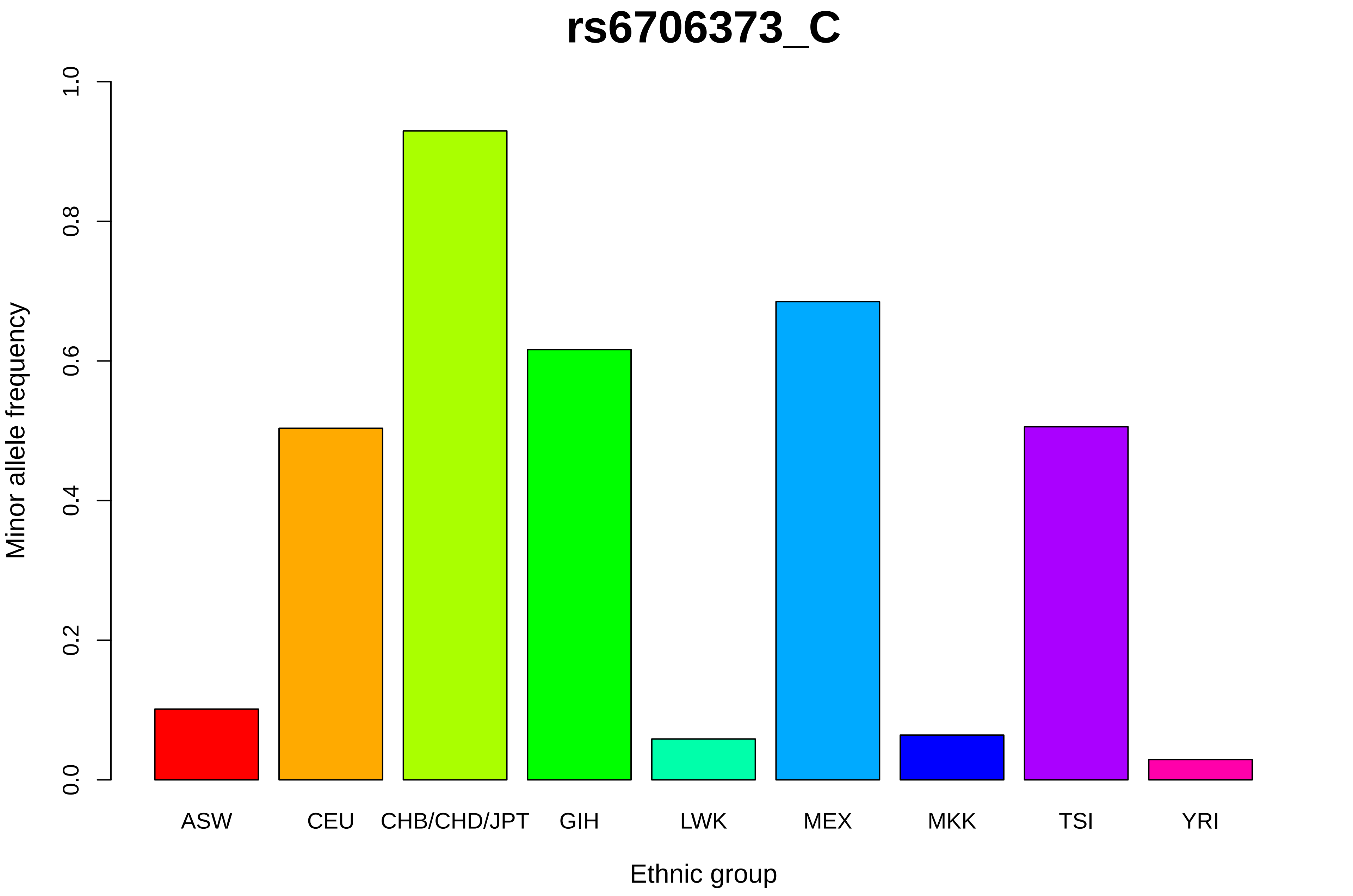
rs6706373_C
Minor allele frequency
0.0
0.2
0.4
0.6
0.8
1.0
ASW
CEU
CHB/CHD/JPT
GIH
LWK
MEX
MKK
TSI
YRI
Ethnic group

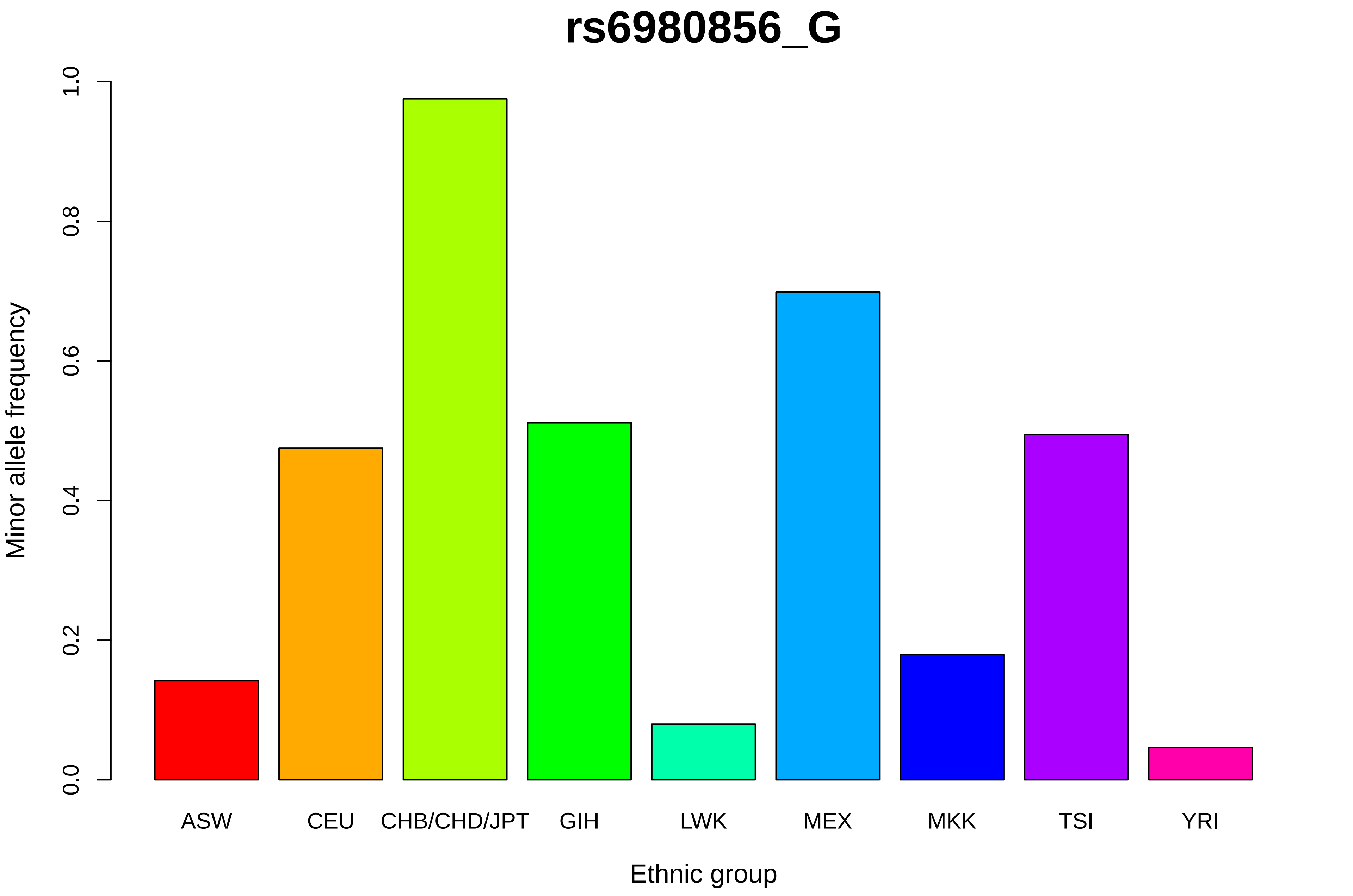
rs6980856_G
Minor allele frequency
0.0
0.2
0.4
0.6
0.8
1.0
ASW
CEU
CHB/CHD/JPT
GIH
LWK
MEX
MKK
TSI
YRI
Ethnic group

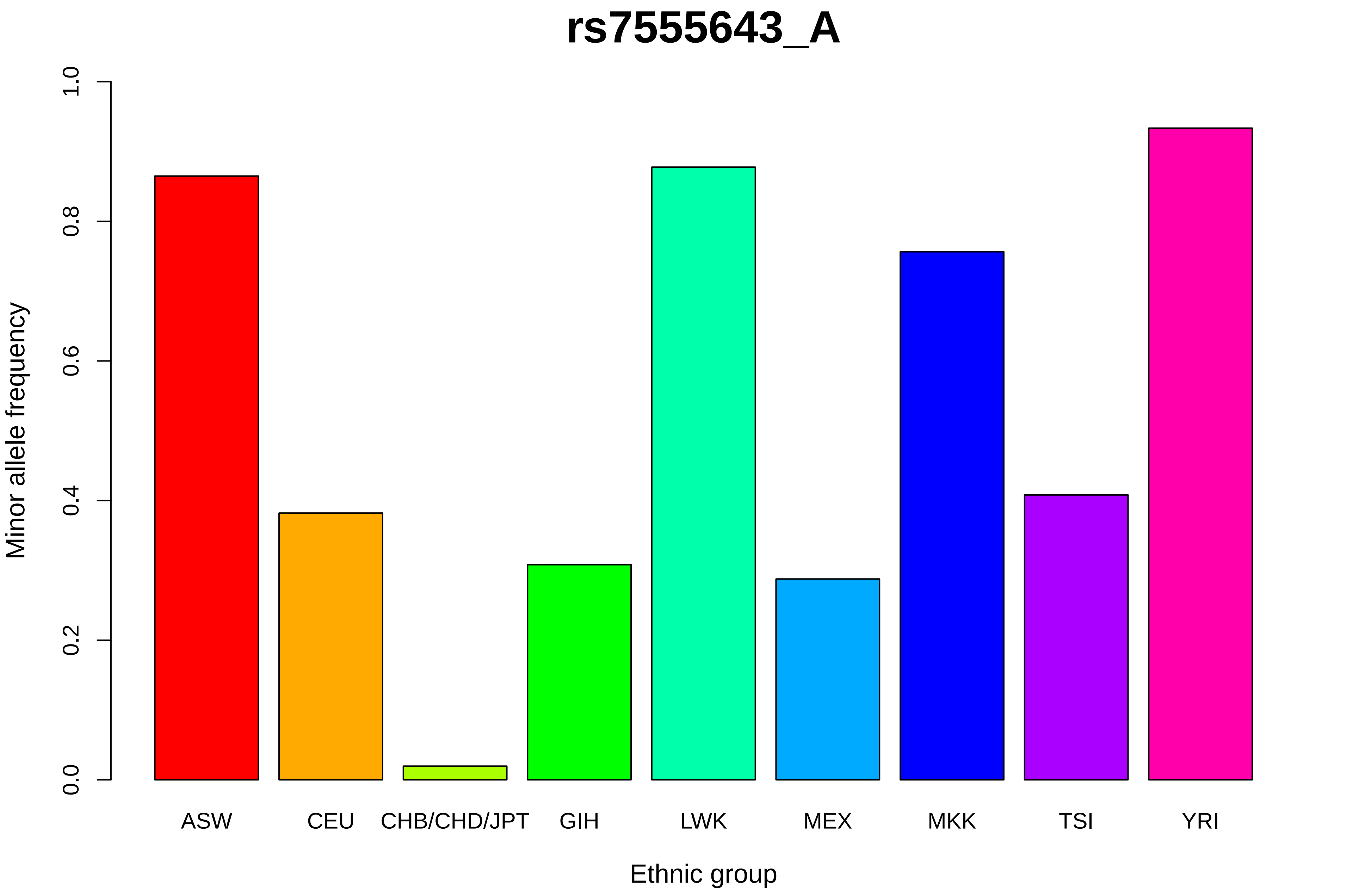
rs7555643_A
Minor allele frequency
0.0
0.2
0.4
0.6
0.8
1.0
ASW
CEU
CHB/CHD/JPT
GIH
LWK
MEX
MKK
TSI
YRI
Ethnic group

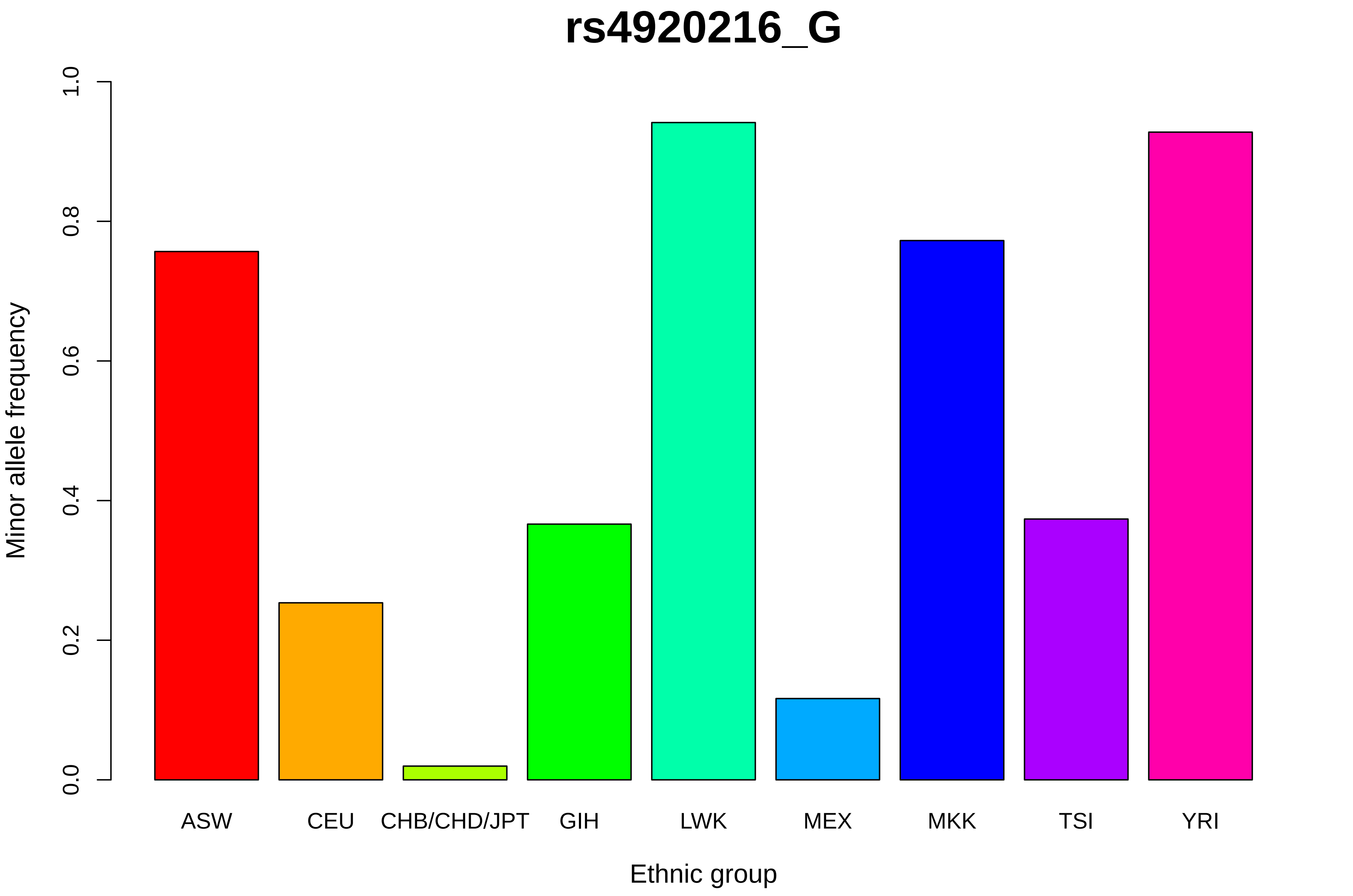
rs4920216_G
Minor allele frequency
0.0
0.2
0.4
0.6
0.8
1.0
ASW
CEU
CHB/CHD/JPT
GIH
LWK
MEX
MKK
TSI
YRI
Ethnic group

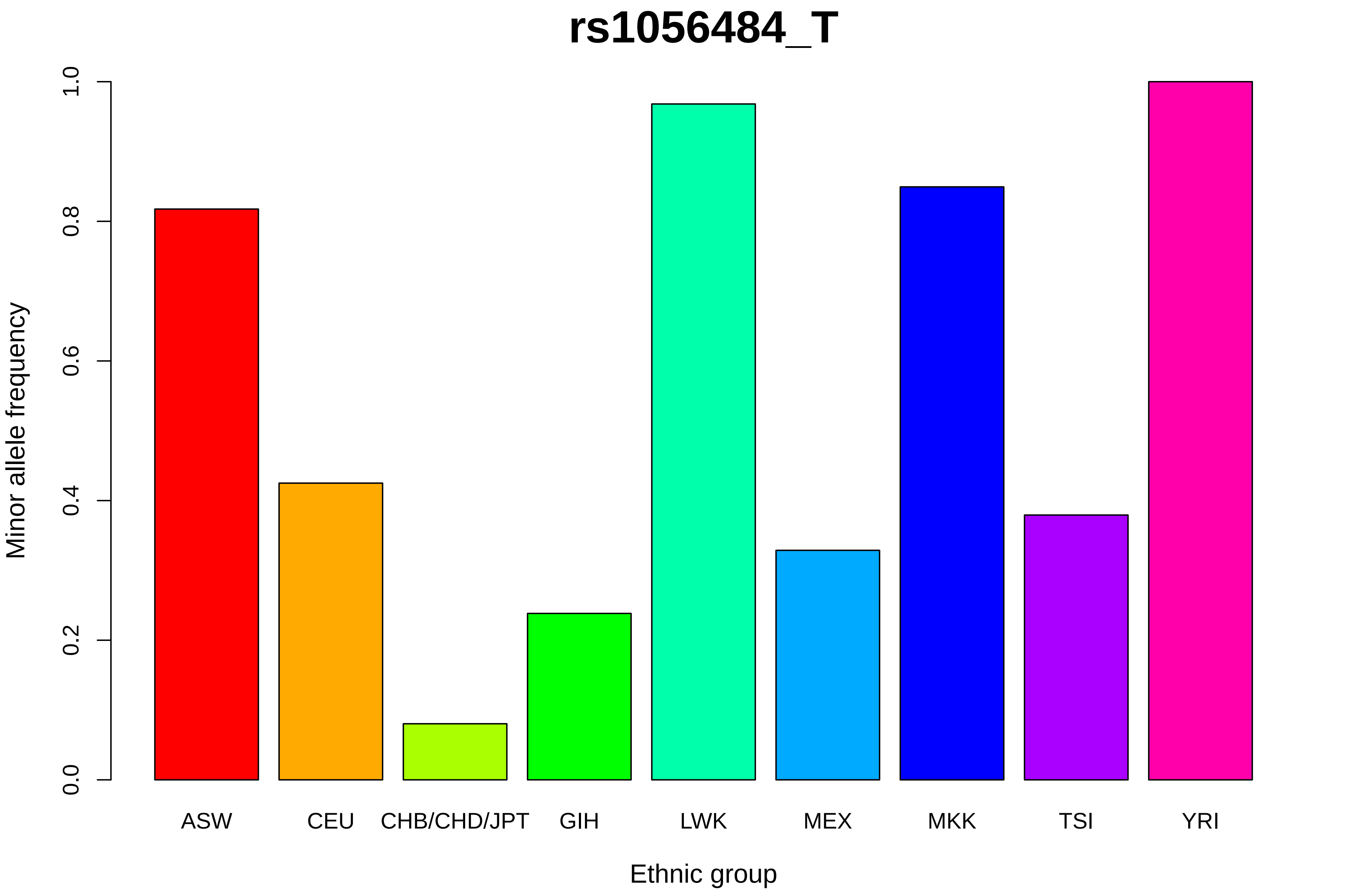
rs1056484_T
Minor allele frequency
0.0
0.2
0.4
0.6
0.8
1.0
ASW
CEU
CHB/CHD/JPT
GIH
LWK
MEX
MKK
TSI
YRI
Ethnic group

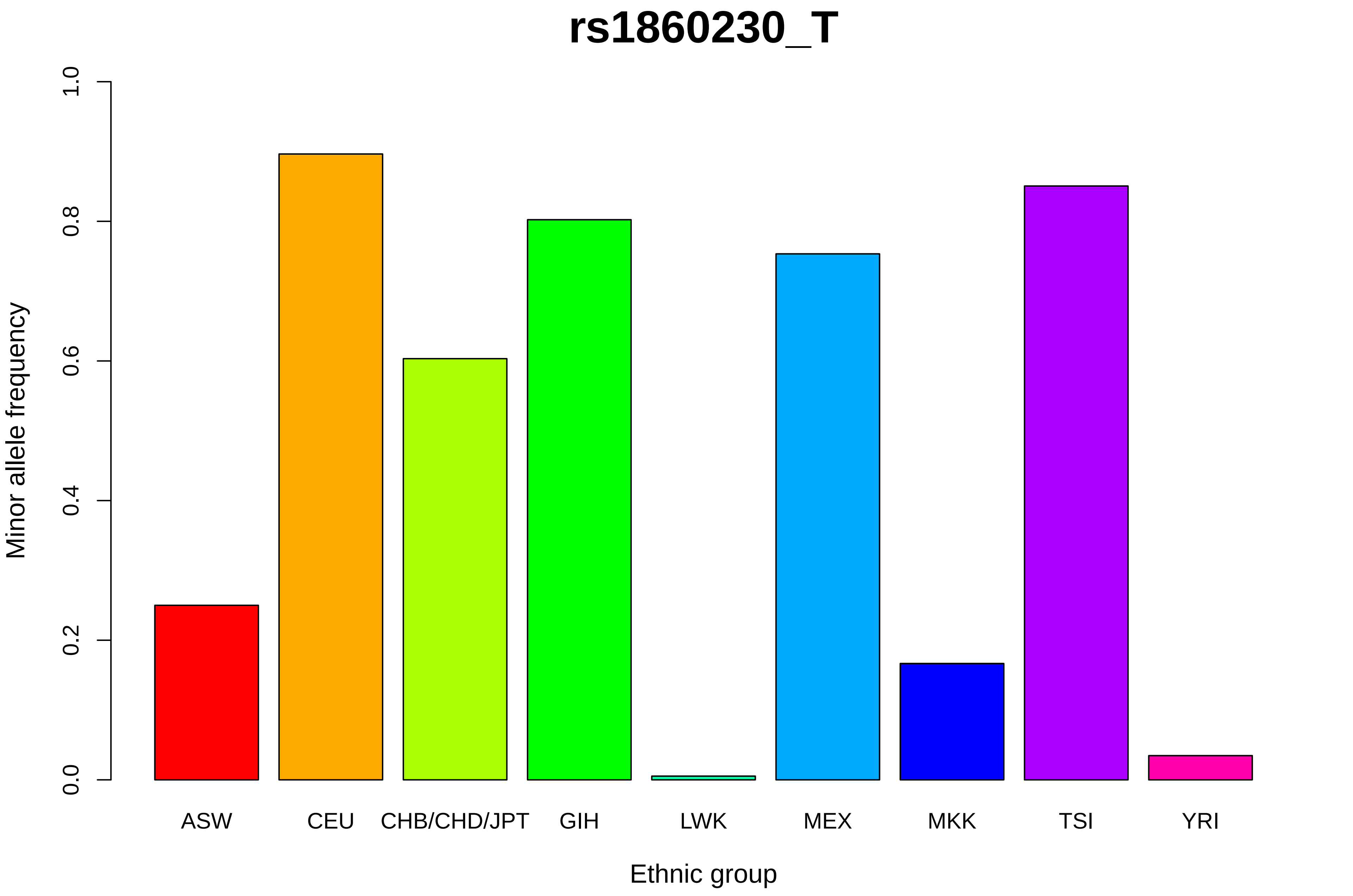
rs1860230_T
Minor allele frequency
0.0
0.2
0.4
0.6
0.8
1.0
ASW
CEU
CHB/CHD/JPT
GIH
LWK
MEX
MKK
TSI
YRI
Ethnic group

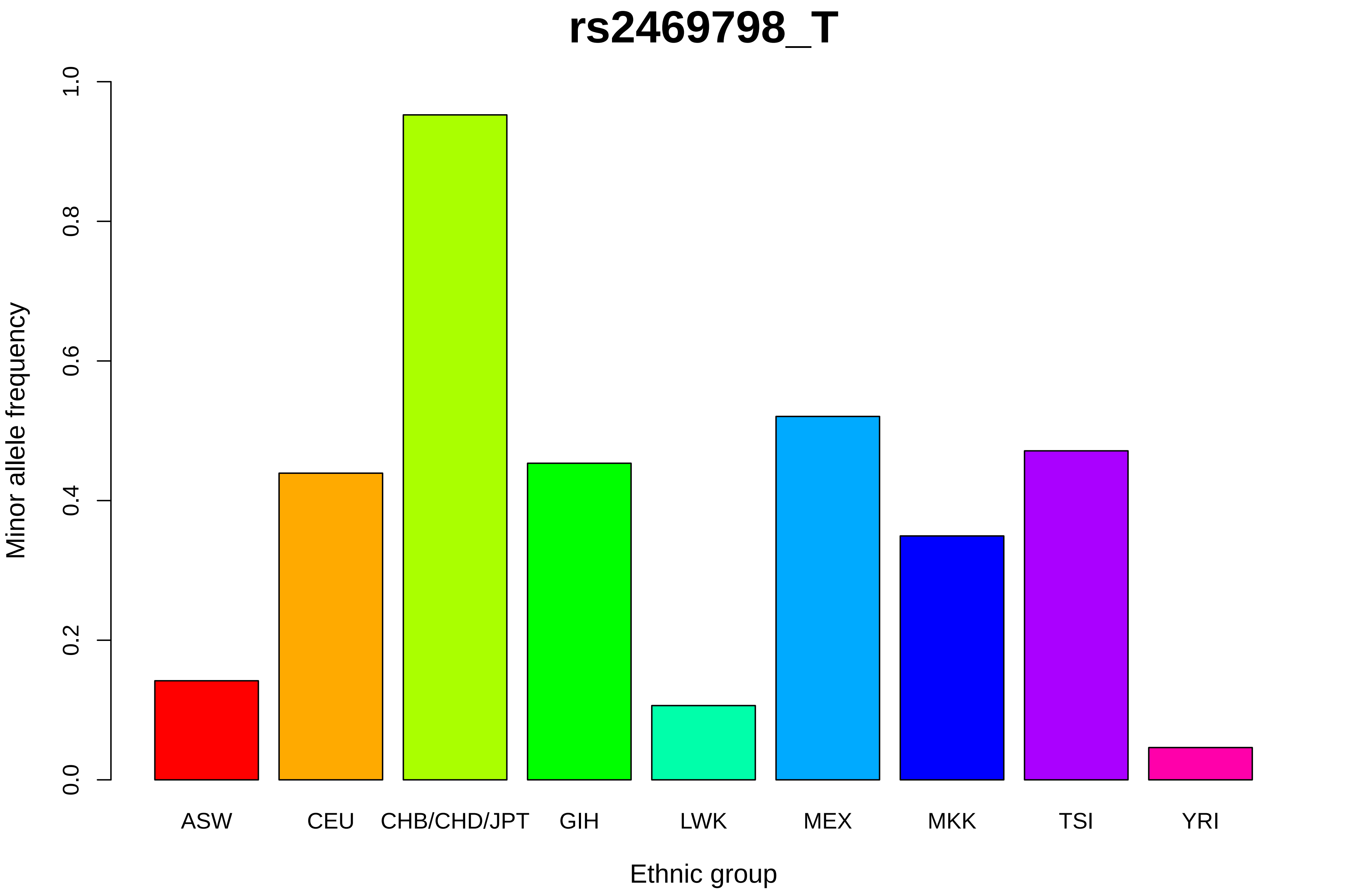
rs2469798_T
Minor allele frequency
0.0
0.2
0.4
0.6
0.8
1.0
ASW
CEU
CHB/CHD/JPT
GIH
LWK
MEX
MKK
TSI
YRI
Ethnic group

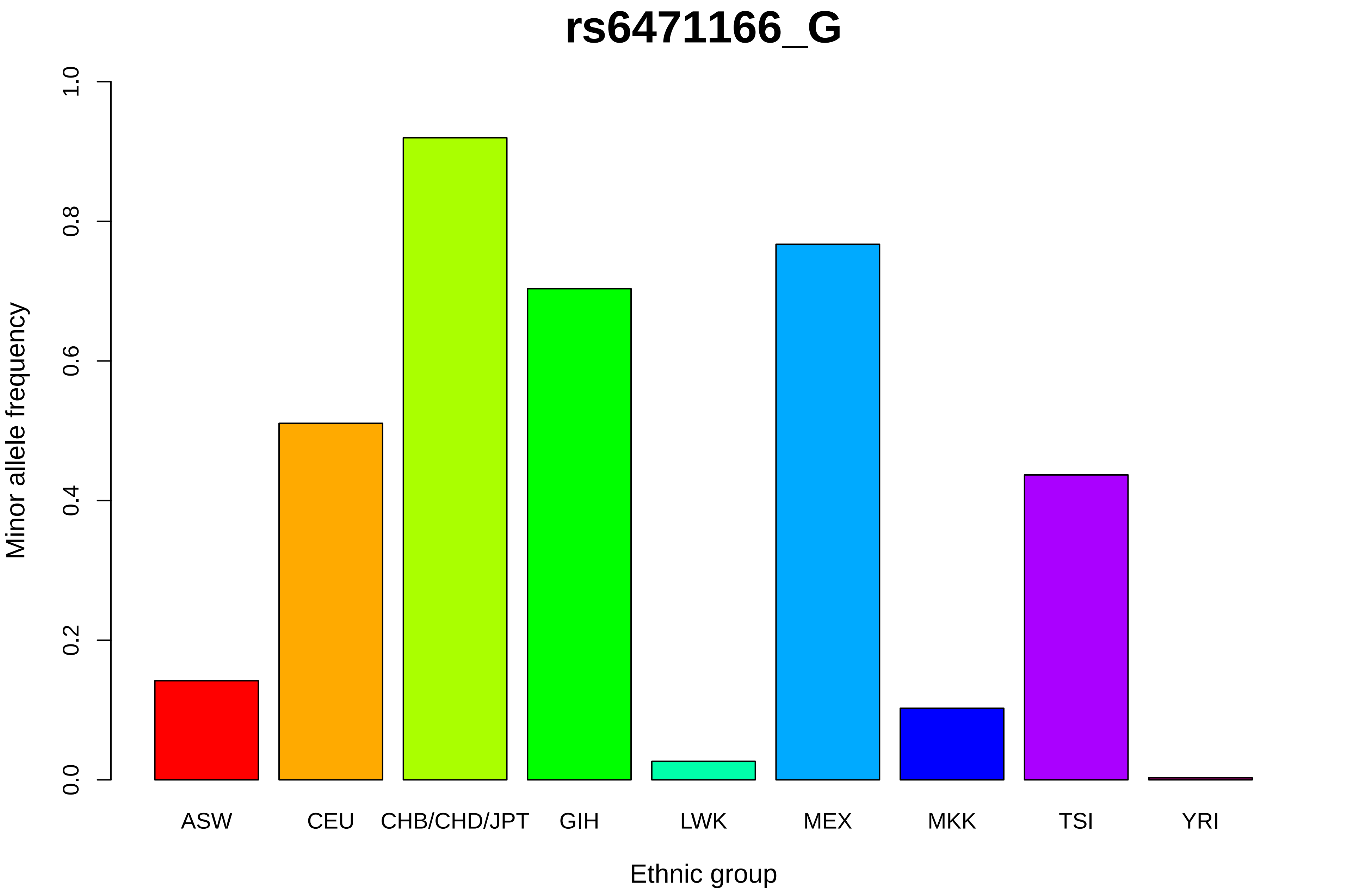
rs6471166_G
Minor allele frequency
0.0
0.2
0.4
0.6
0.8
1.0
ASW
CEU
CHB/CHD/JPT
GIH
LWK
MEX
MKK
TSI
YRI
Ethnic group

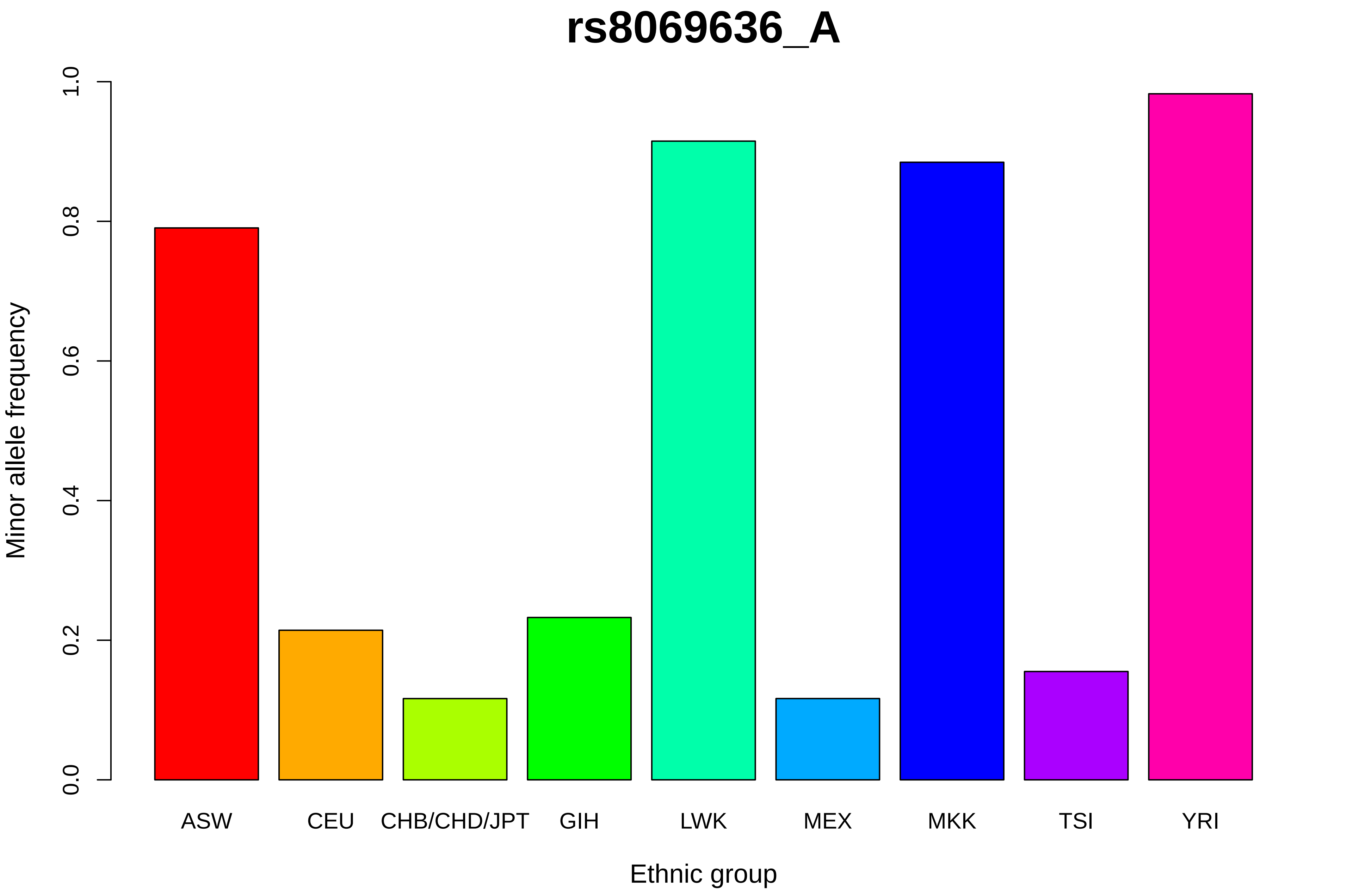
rs8069636_A
Minor allele frequency
0.0
0.2
0.4
0.6
0.8
1.0
ASW
CEU
CHB/CHD/JPT
GIH
LWK
MEX
MKK
TSI
YRI
Ethnic group

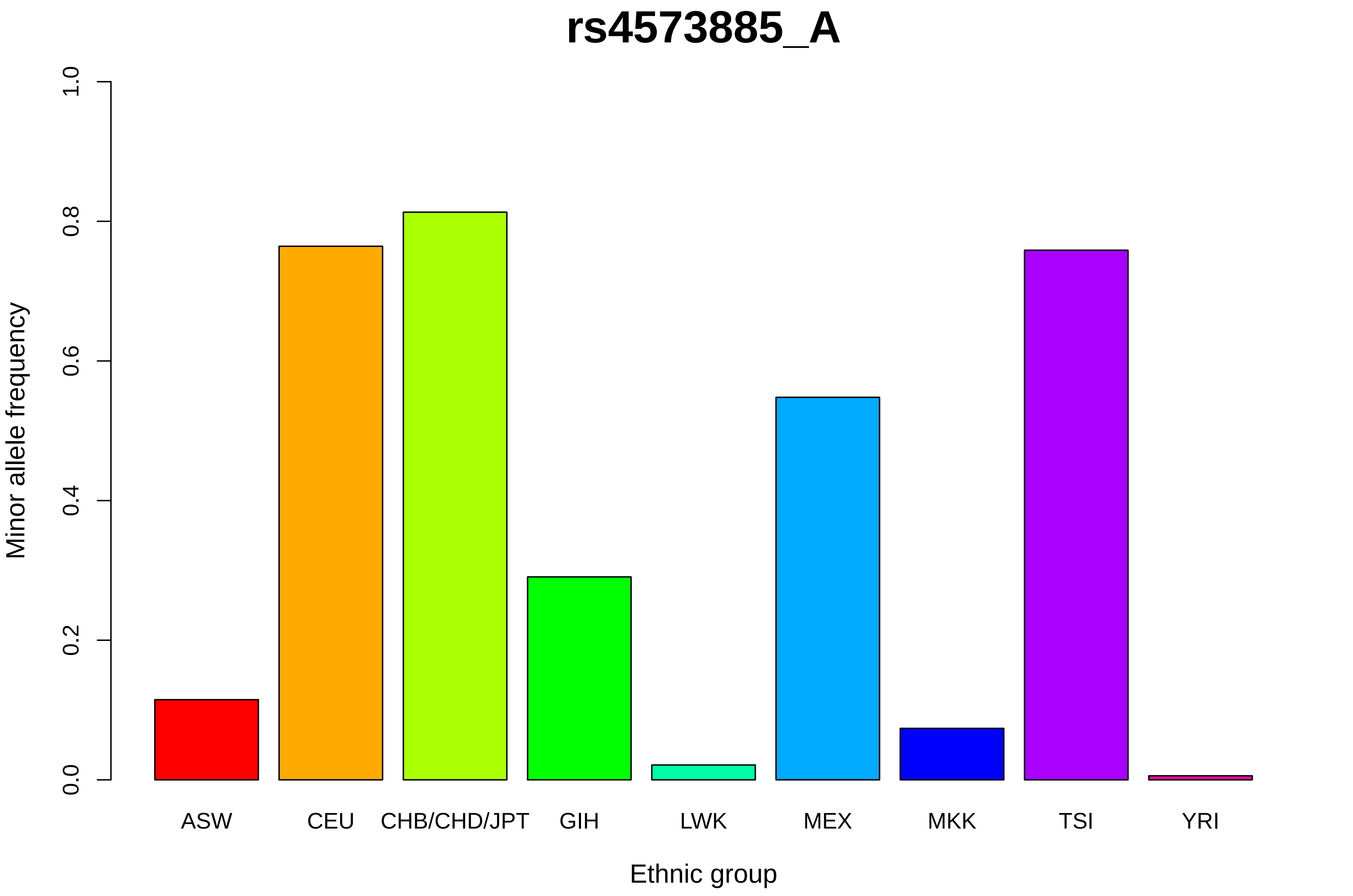
rs4573885_A
Minor allele frequency
0.0
0.2
0.4
0.6
0.8
1.0
ASW
CEU
CHB/CHD/JPT
GIH
LWK
MEX
MKK
TSI
YRI
Ethnic group

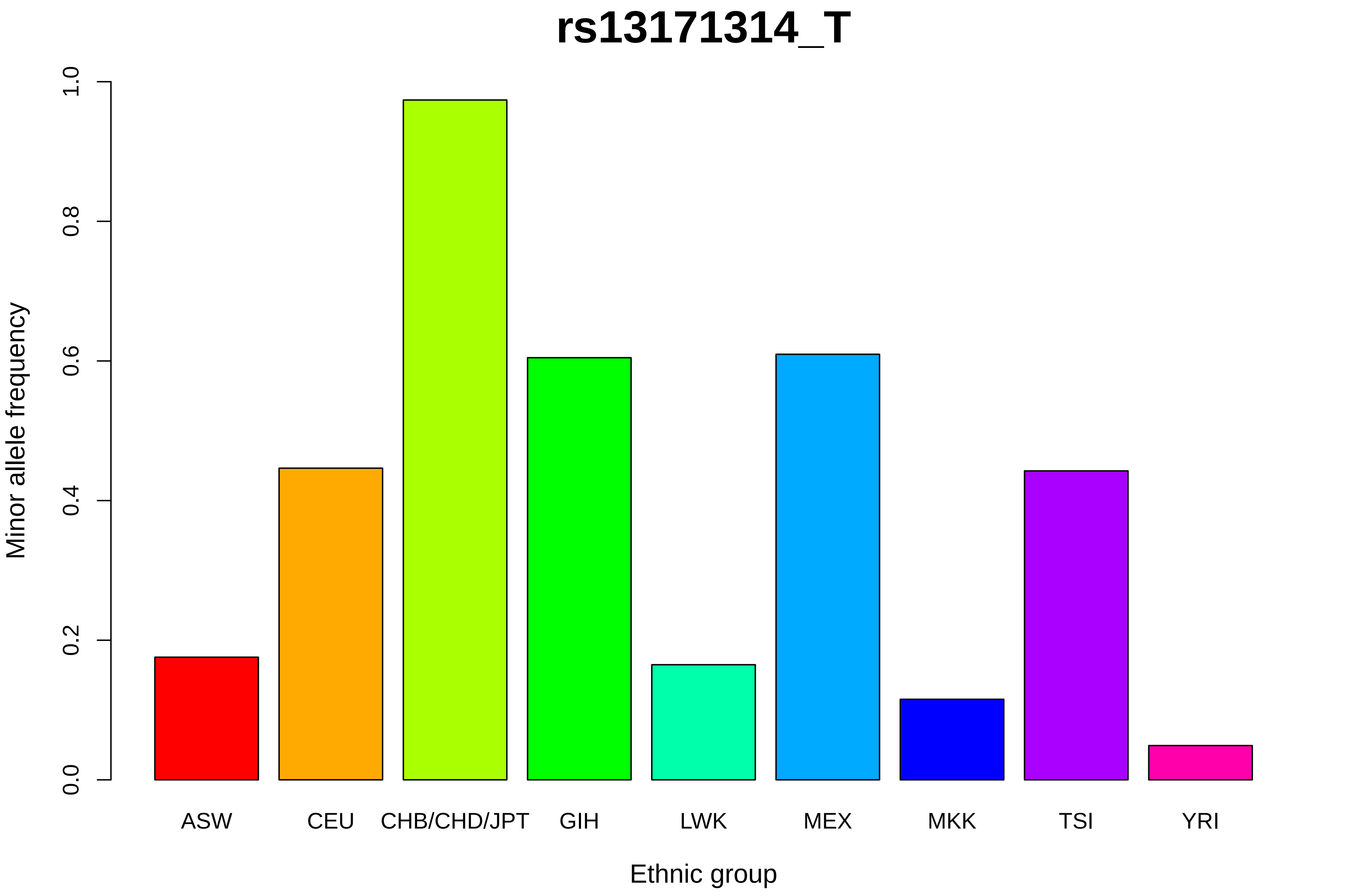
rs13171314_T
Minor allele frequency
0.0
0.2
0.4
0.6
0.8
1.0
ASW
CEU
CHB/CHD/JPT
GIH
LWK
MEX
MKK
TSI
YRI
Ethnic group

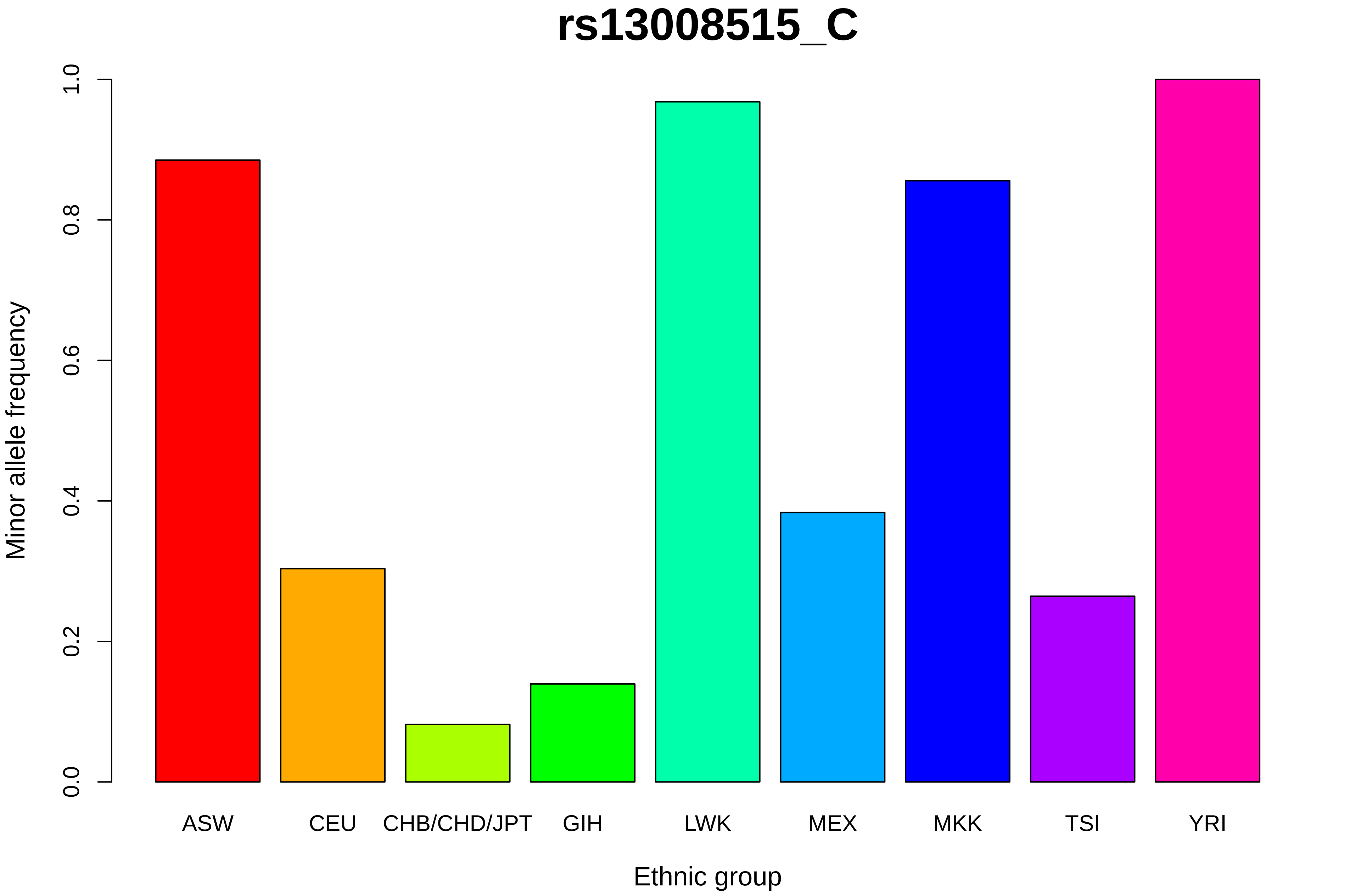

rs13008515_C
Minor allele frequency
0.0
0.2
0.4
0.6
0.8
1.0
ASW
CEU
CHB/CHD/JPT
GIH
LWK
MEX
MKK
TSI
YRI
Ethnic group

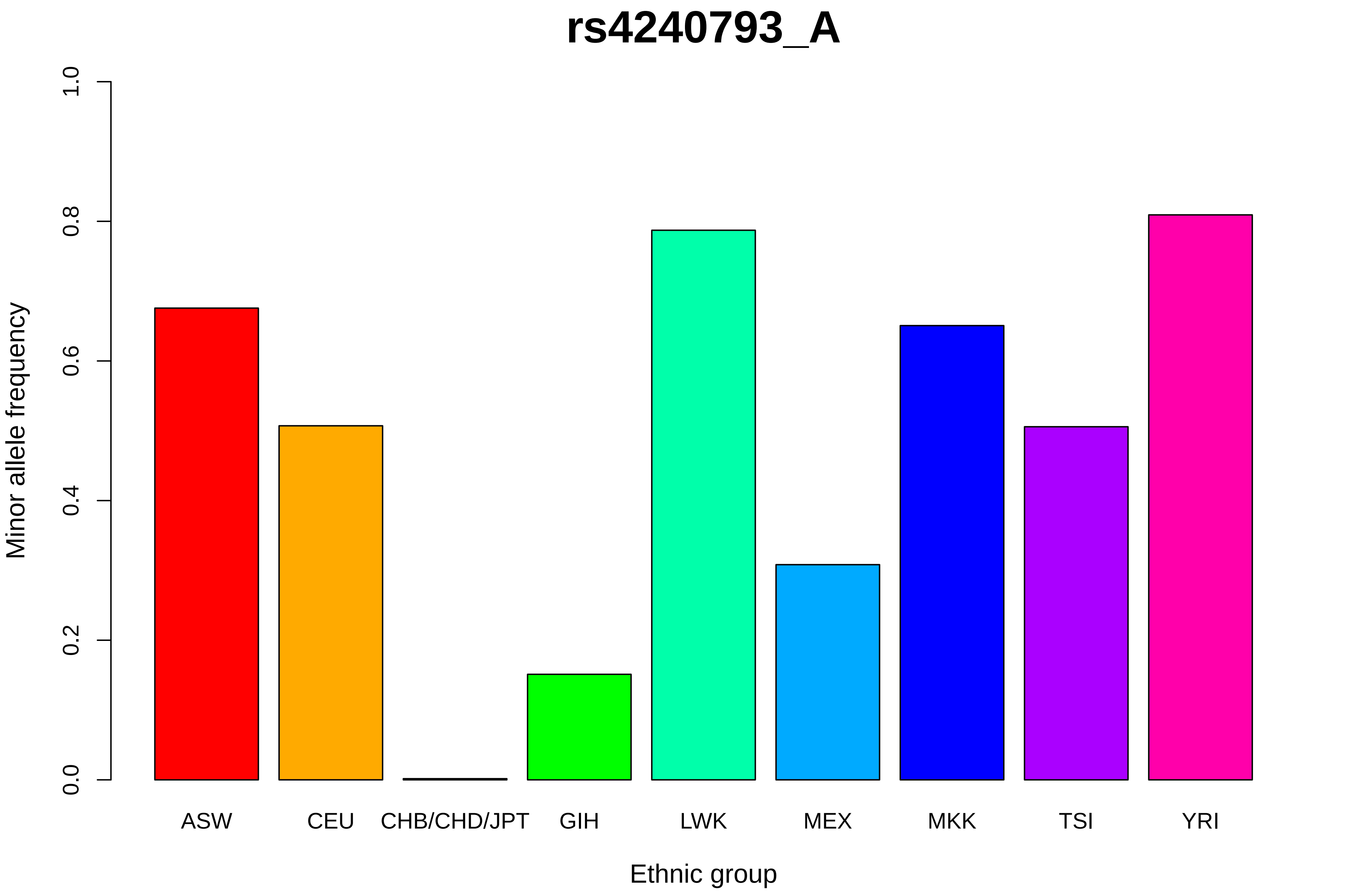
rs4240793_A
Minor allele frequency
0.0
0.2
0.4
0.6
0.8
1.0
ASW
CEU
CHB/CHD/JPT
GIH
LWK
MEX
MKK
TSI
YRI
Ethnic group

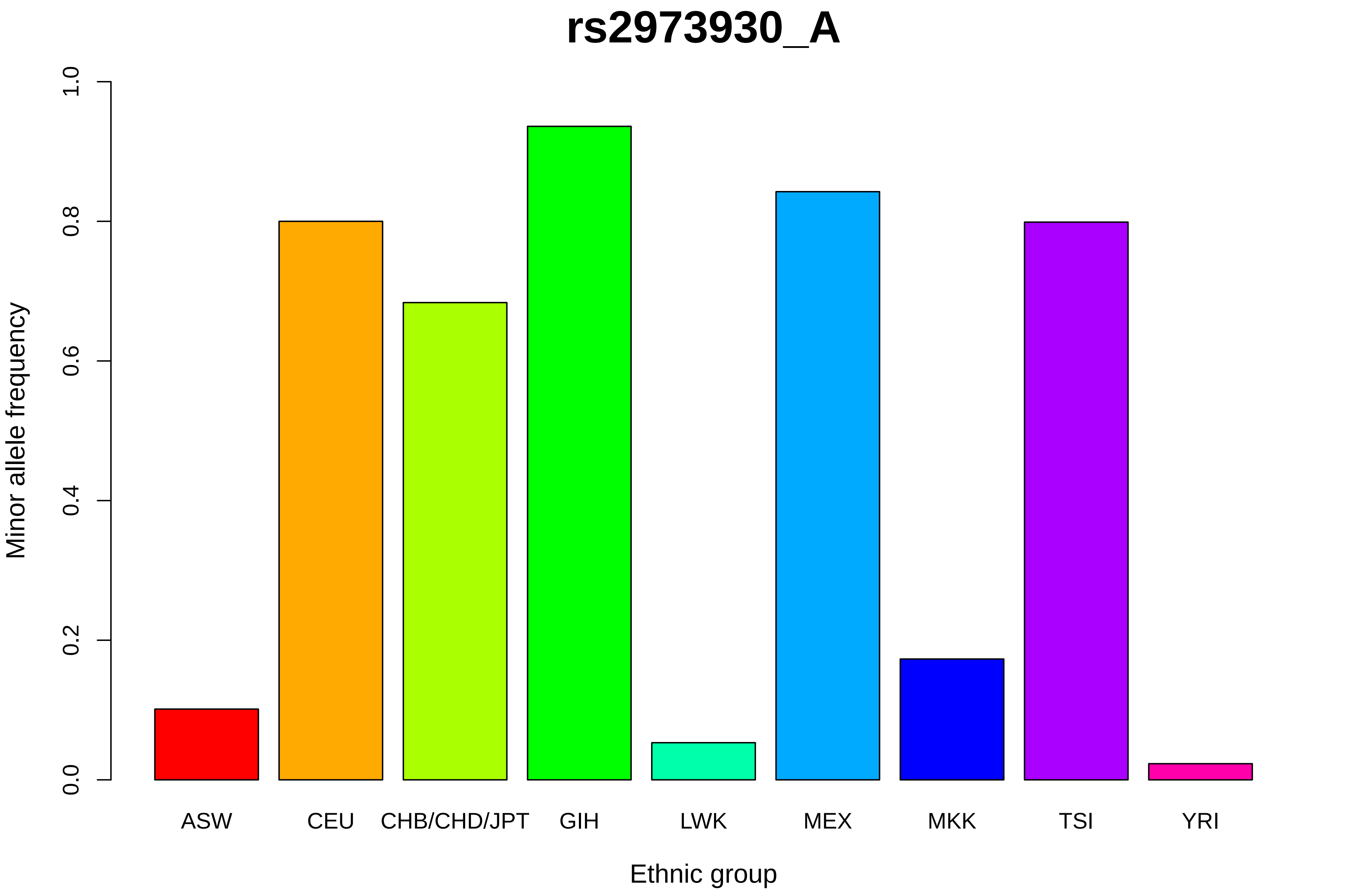
rs2973930_A
Minor allele frequency
0.0
0.2
0.4
0.6
0.8
1.0
ASW
CEU
CHB/CHD/JPT
GIH
LWK
MEX
MKK
TSI
YRI
Ethnic group

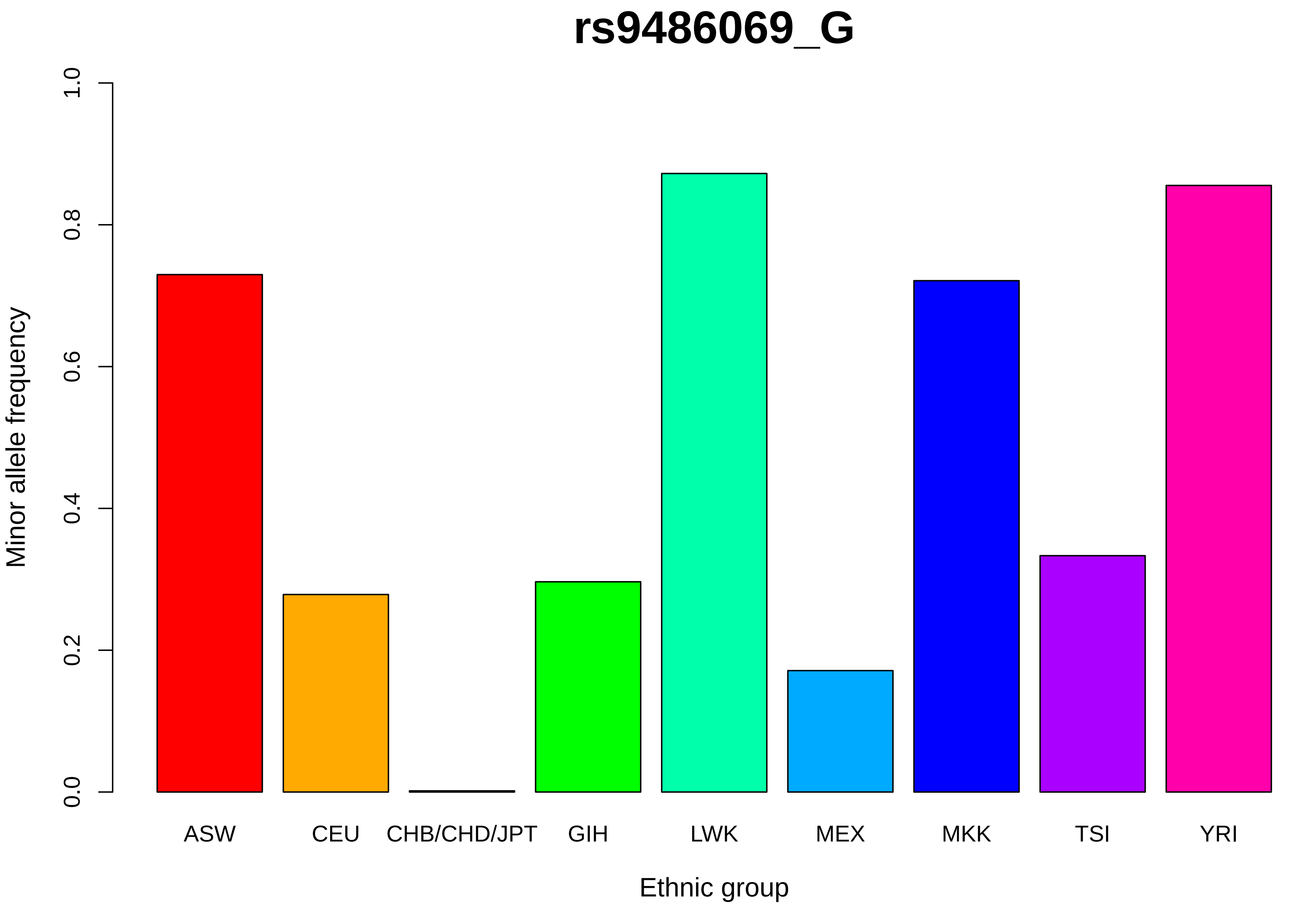
rs9486069_G
Minor allele frequency
0.0
0.2
0.4
0.6
0.8
1.0
ASW
CEU
CHB/CHD/JPT
GIH
LWK
MEX
MKK
TSI
YRI
Ethnic group

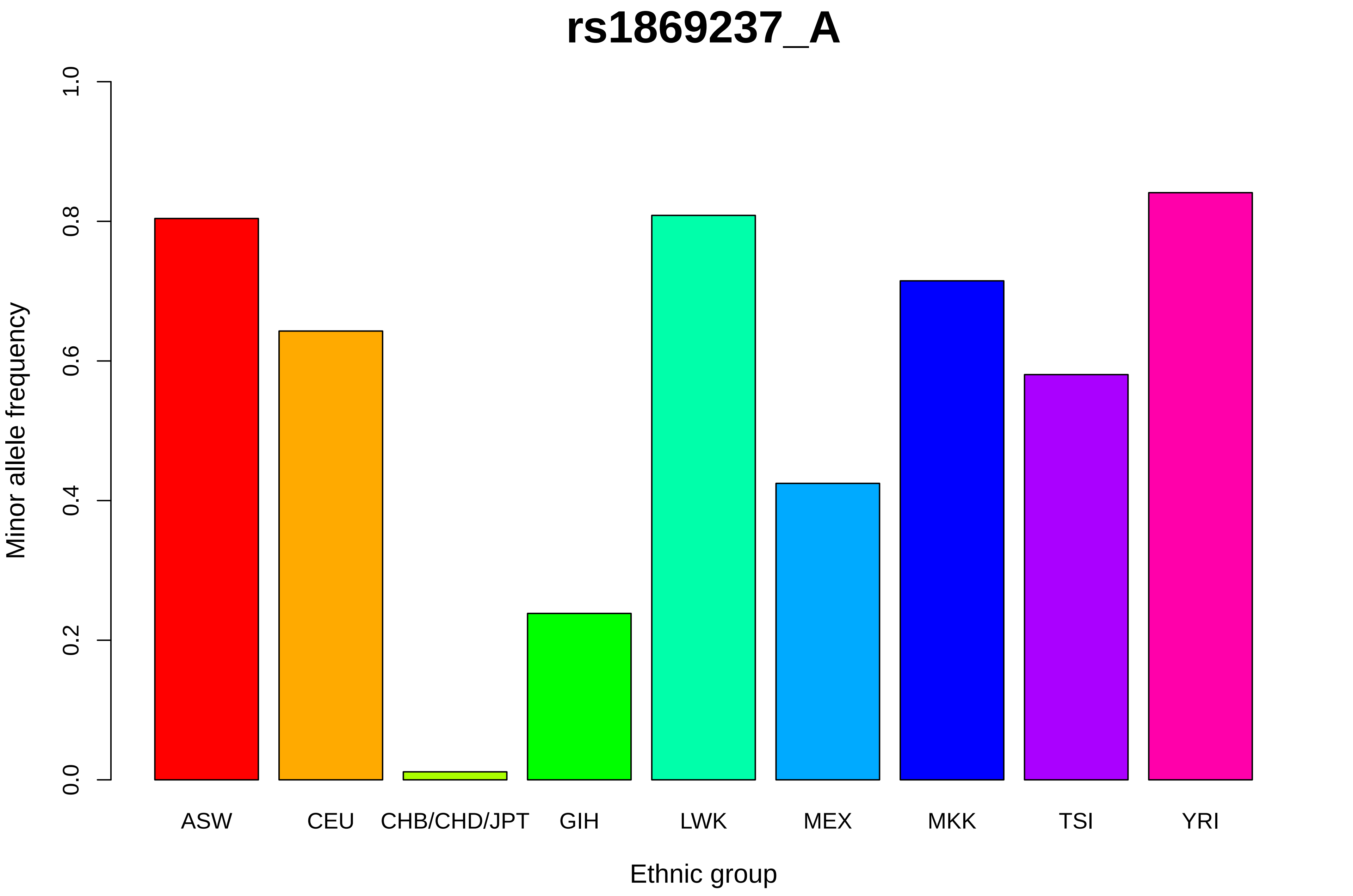

rs1869237_A
Minor allele frequency
0.0
0.2
0.4
0.6
0.8
1.0
ASW
CEU
CHB/CHD/JPT
GIH
LWK
MEX
MKK
TSI
YRI
Ethnic group

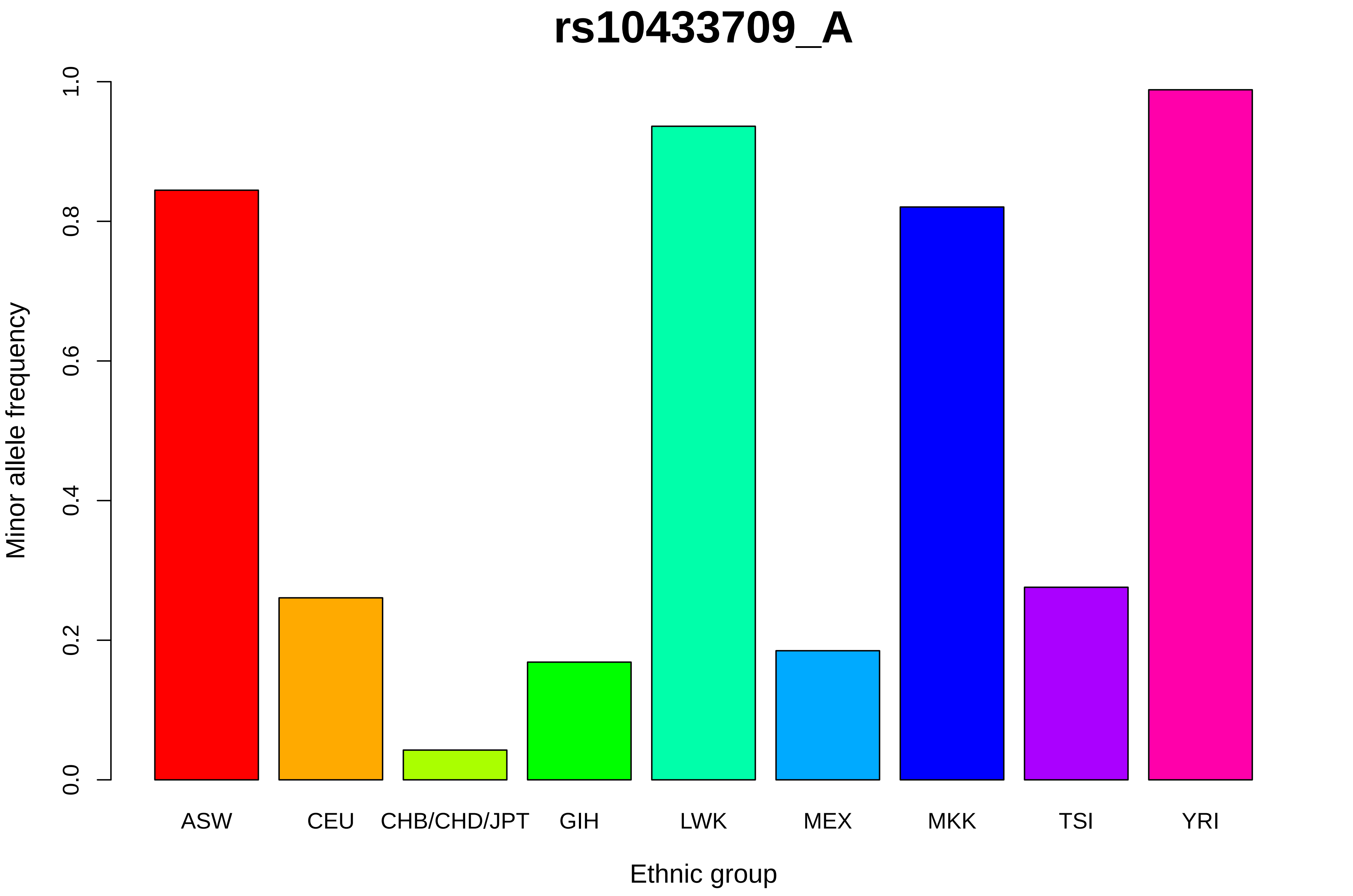

rs10433709_A
Minor allele frequency
0.0
0.2
0.4
0.6
0.8
1.0
ASW
CEU
CHB/CHD/JPT
GIH
LWK
MEX
MKK
TSI
YRI
Ethnic group

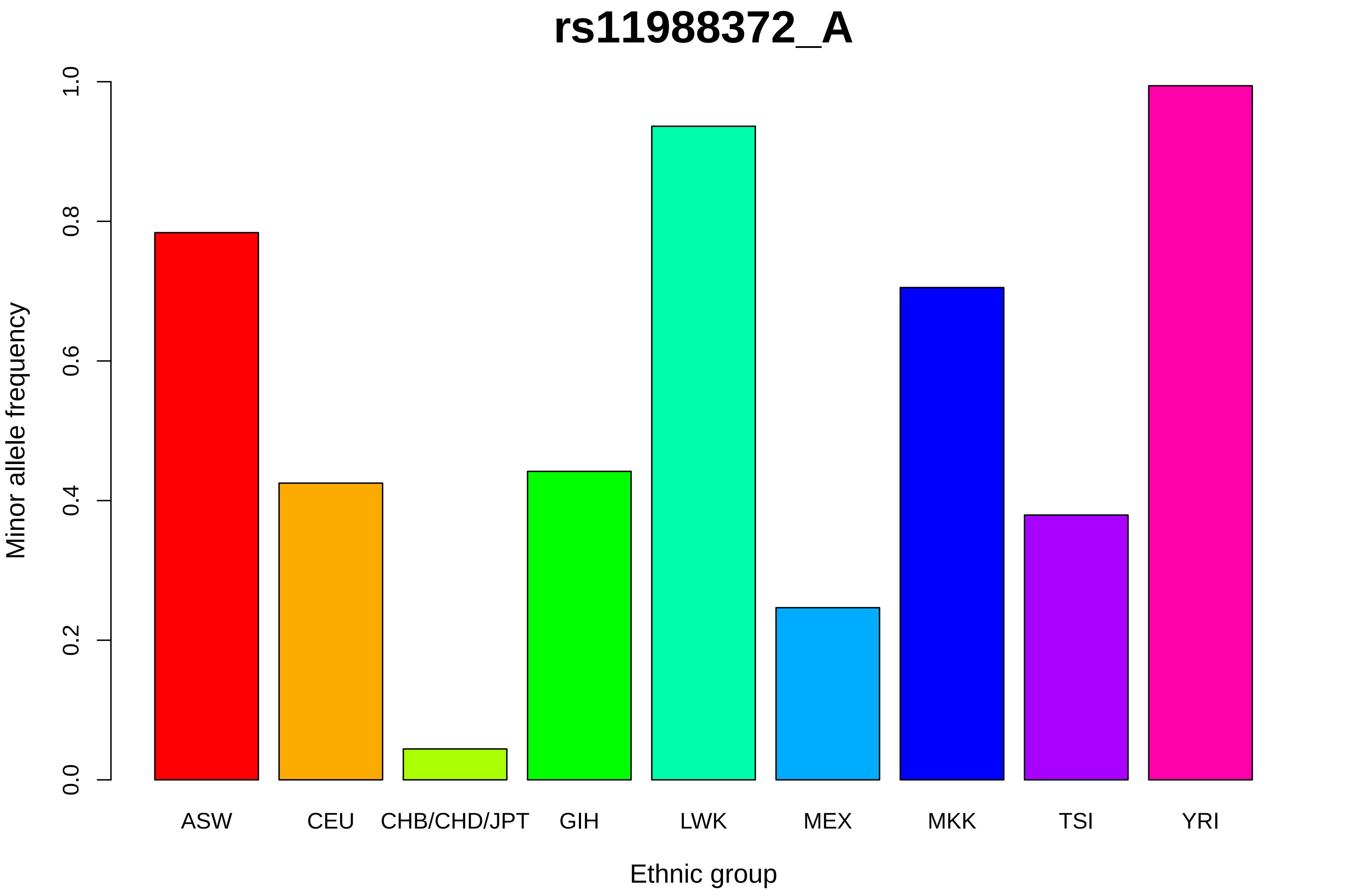
rs11988372_A
Minor allele frequency
0.0
0.2
0.4
0.6
0.8
1.0
ASW
CEU
CHB/CHD/JPT
GIH
LWK
MEX
MKK
TSI
YRI
Ethnic group

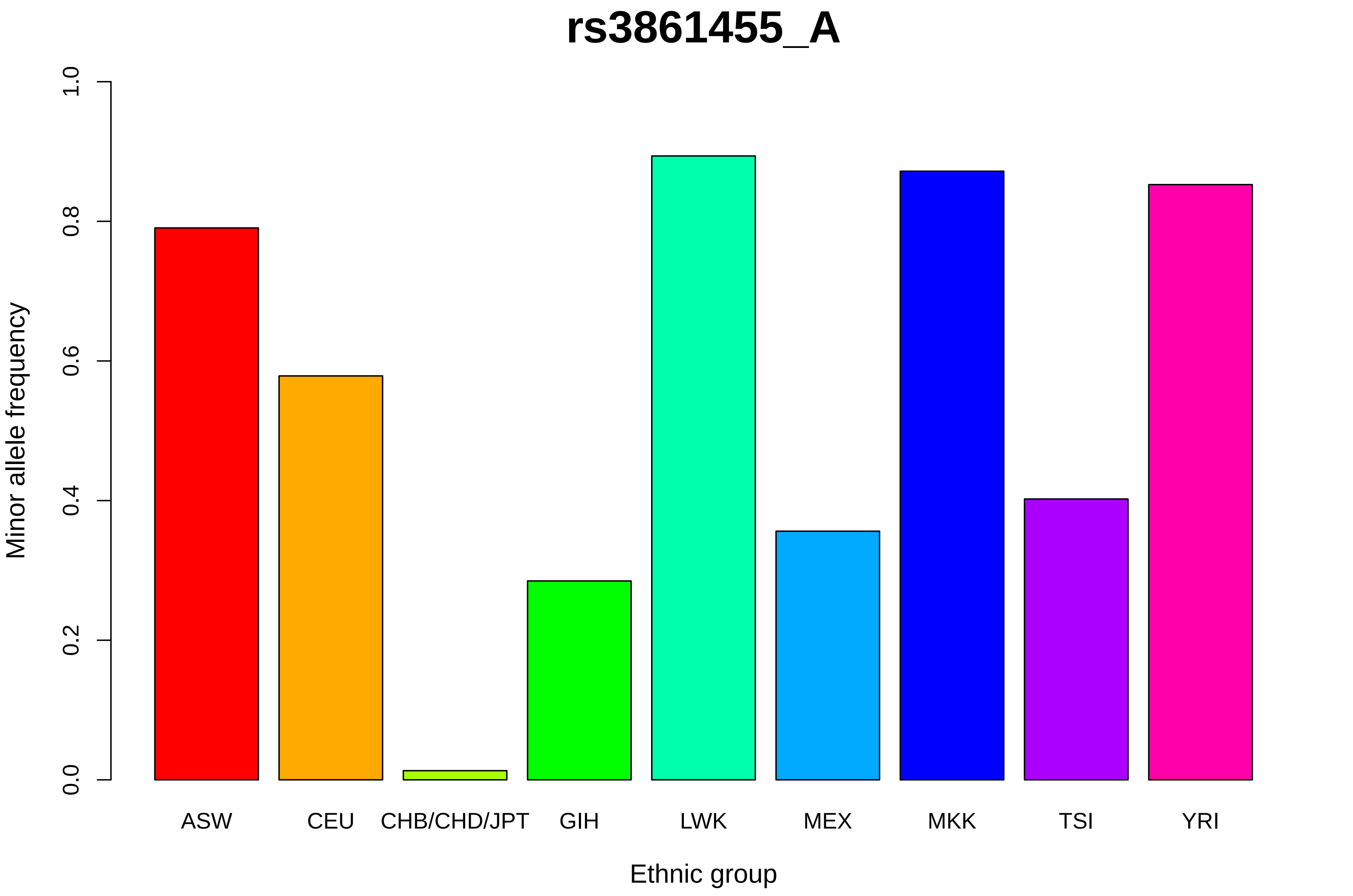
rs3861455_A
Minor allele frequency
0.0
0.2
0.4
0.6
0.8
1.0
ASW
CEU
CHB/CHD/JPT
GIH
LWK
MEX
MKK
TSI
YRI
Ethnic group

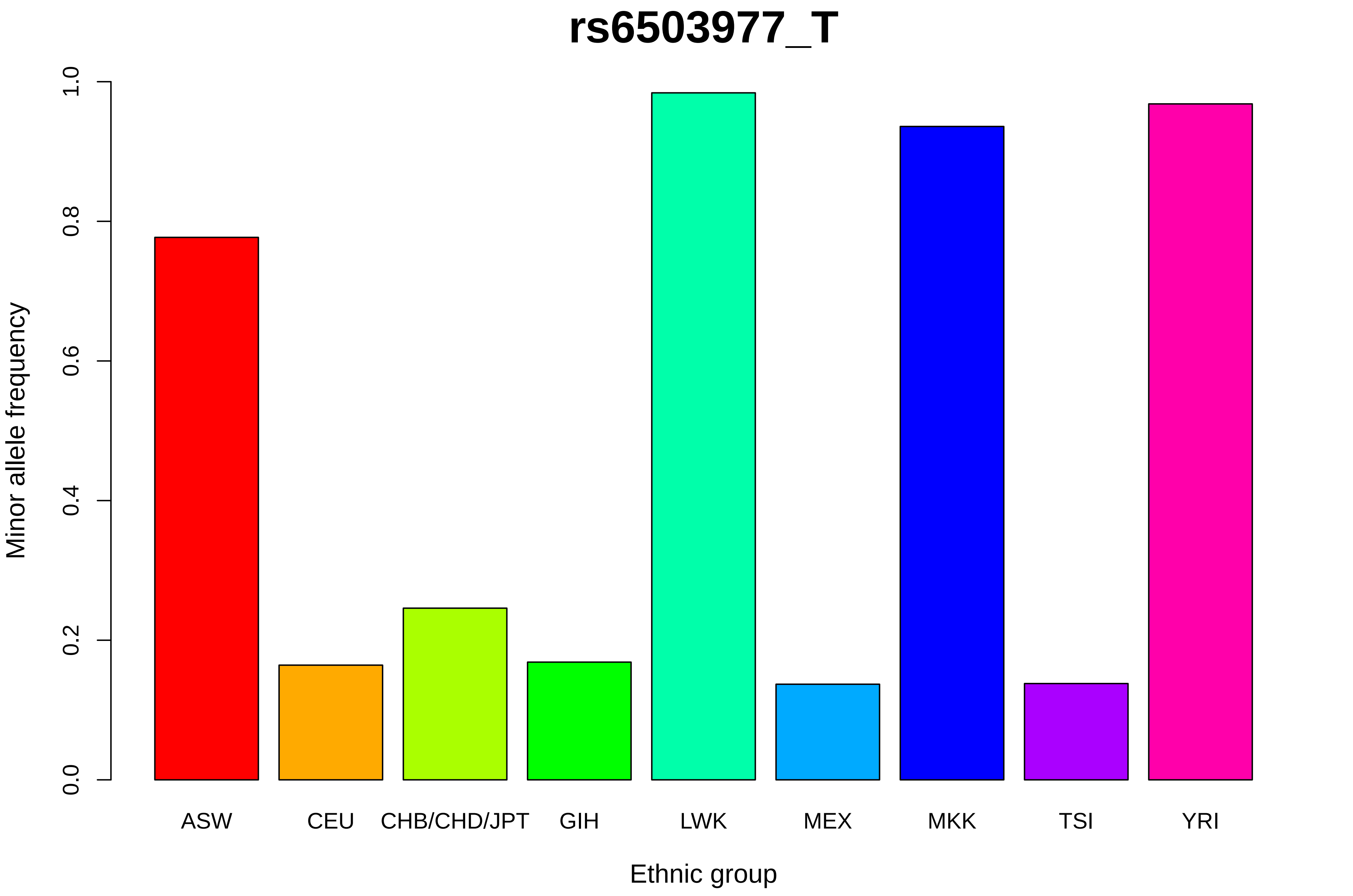
rs6503977_T
Minor allele frequency
0.0
0.2
0.4
0.6
0.8
1.0
ASW
CEU
CHB/CHD/JPT
GIH
LWK
MEX
MKK
TSI
YRI
Ethnic group

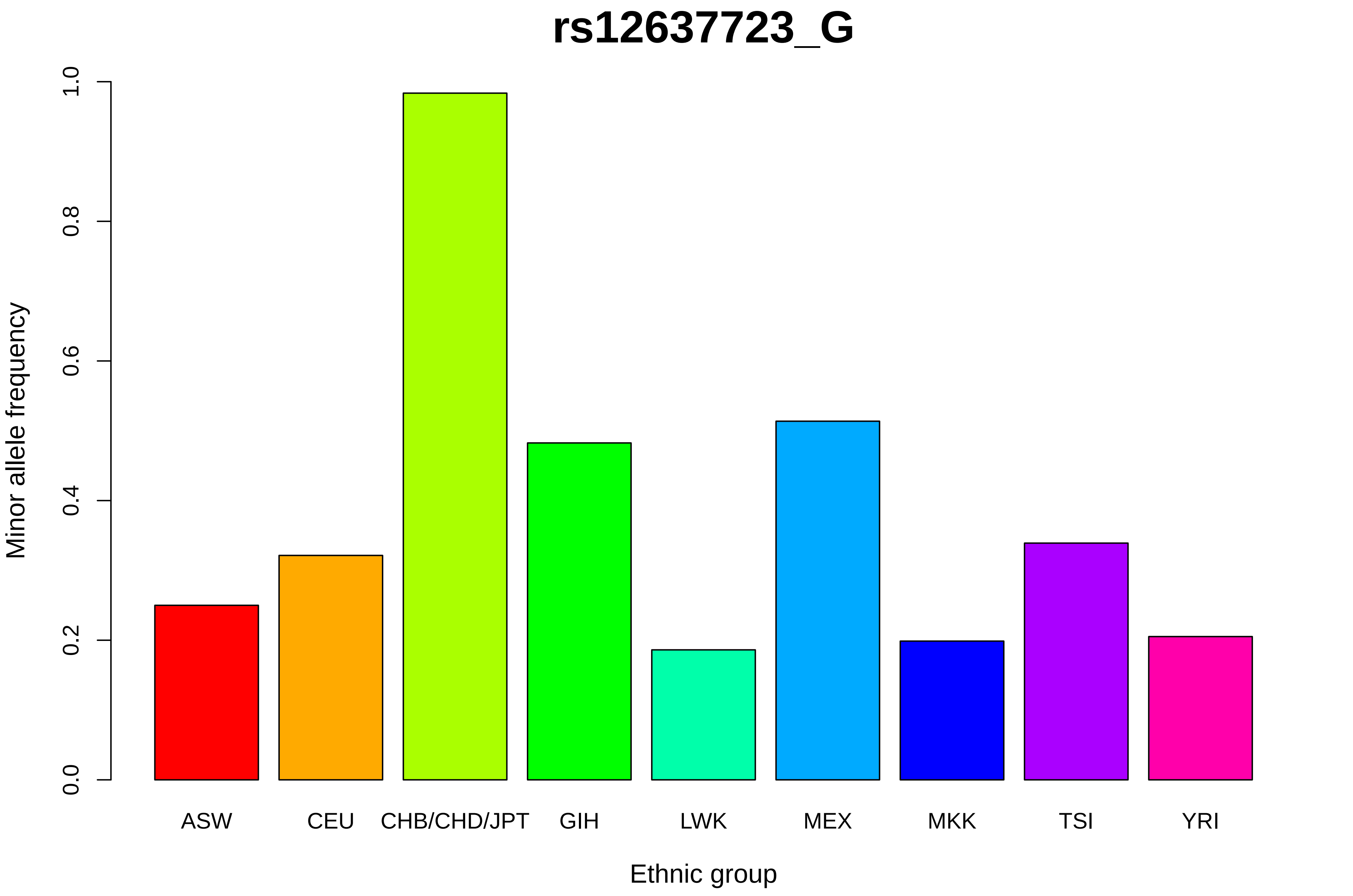
rs12637723_G
Minor allele frequency
0.0
0.2
0.4
0.6
0.8
1.0
ASW
CEU
CHB/CHD/JPT
GIH
LWK
MEX
MKK
TSI
YRI
Ethnic group

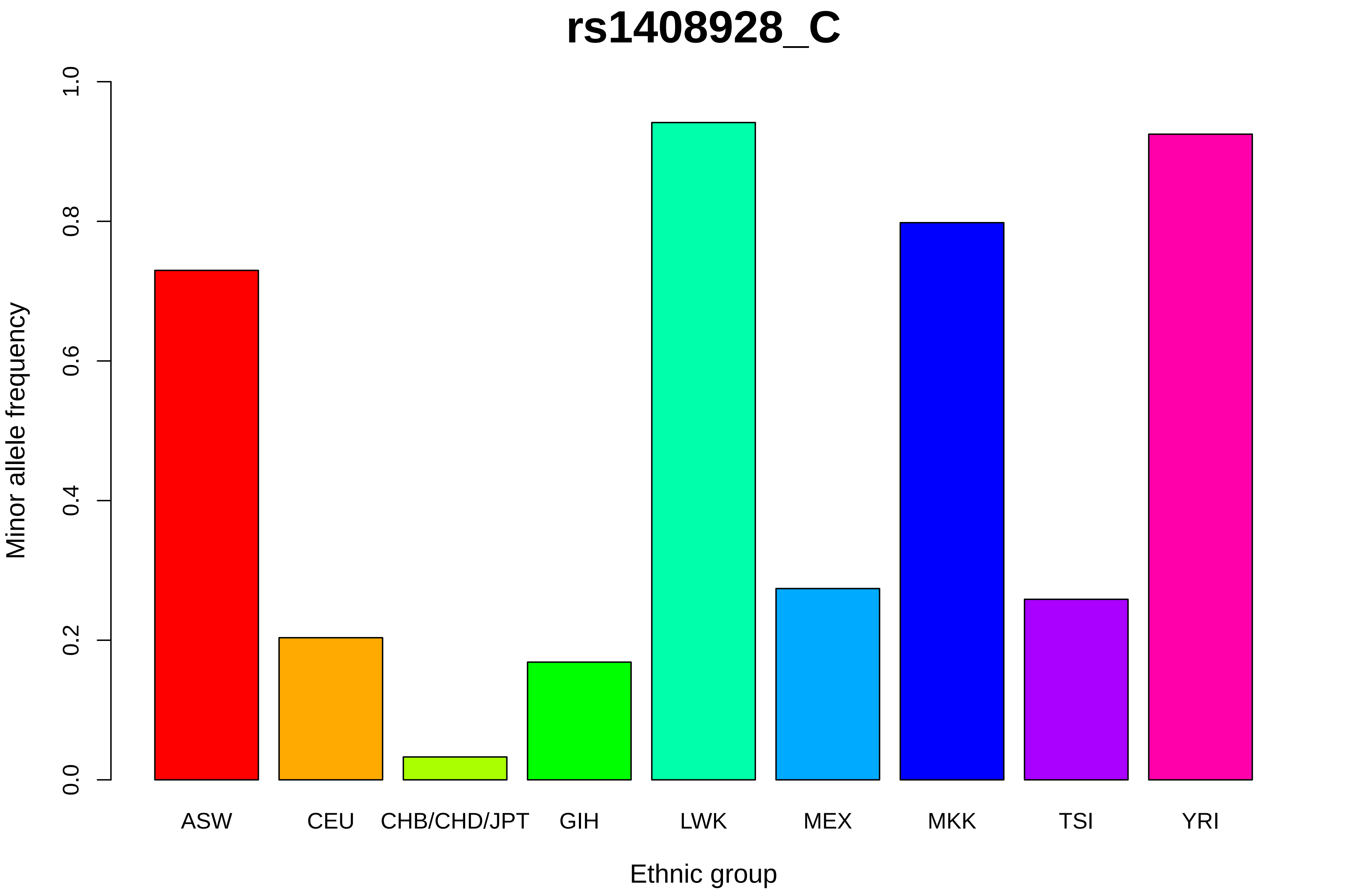
rs1408928_C
Minor allele frequency
0.0
0.2
0.4
0.6
0.8
1.0
ASW
CEU
CHB/CHD/JPT
GIH
LWK
MEX
MKK
TSI
YRI
Ethnic group

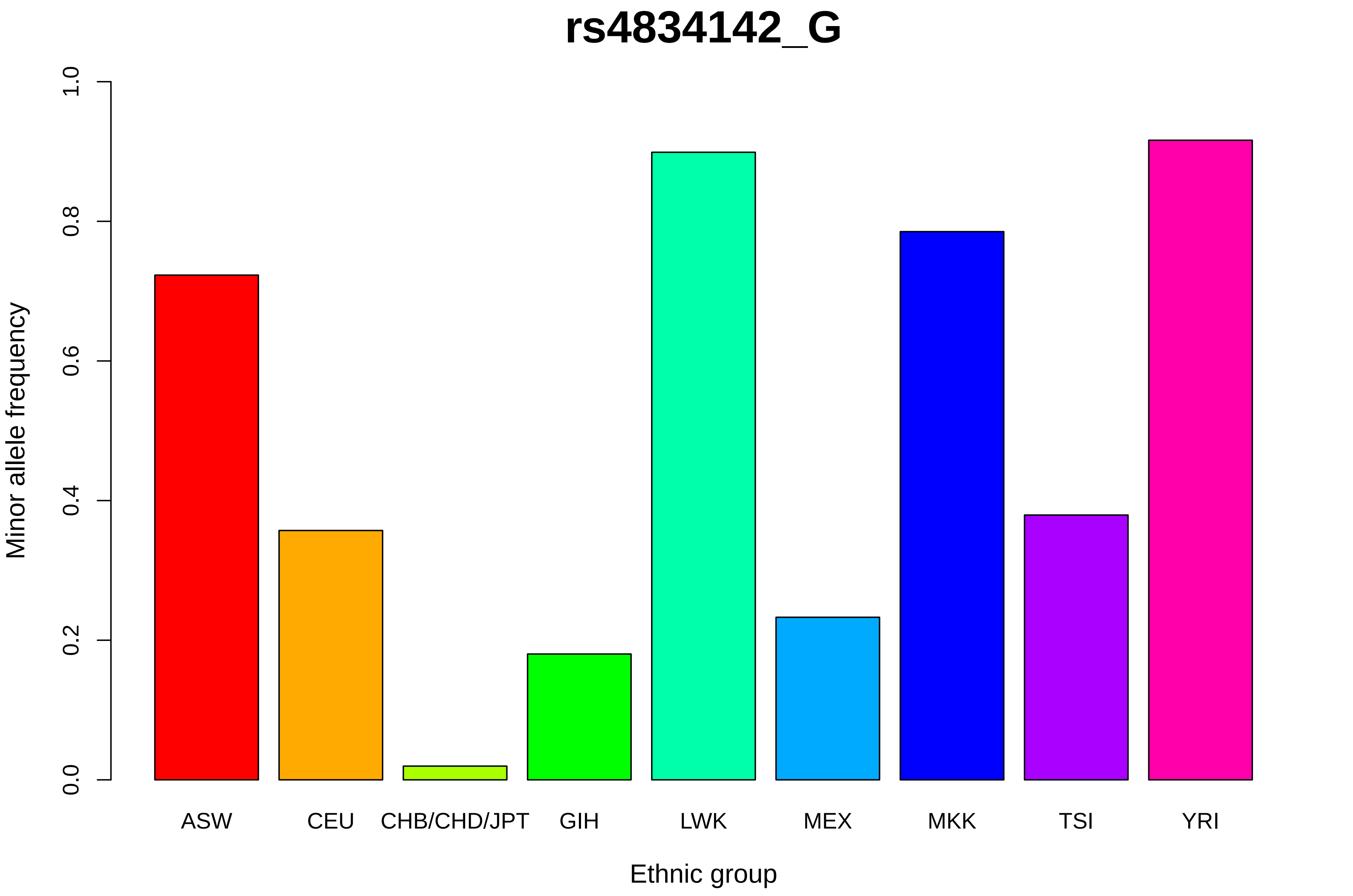
rs4834142_G
Minor allele frequency
0.0
0.2
0.4
0.6
0.8
1.0
ASW
CEU
CHB/CHD/JPT
GIH
LWK
MEX
MKK
TSI
YRI
Ethnic group

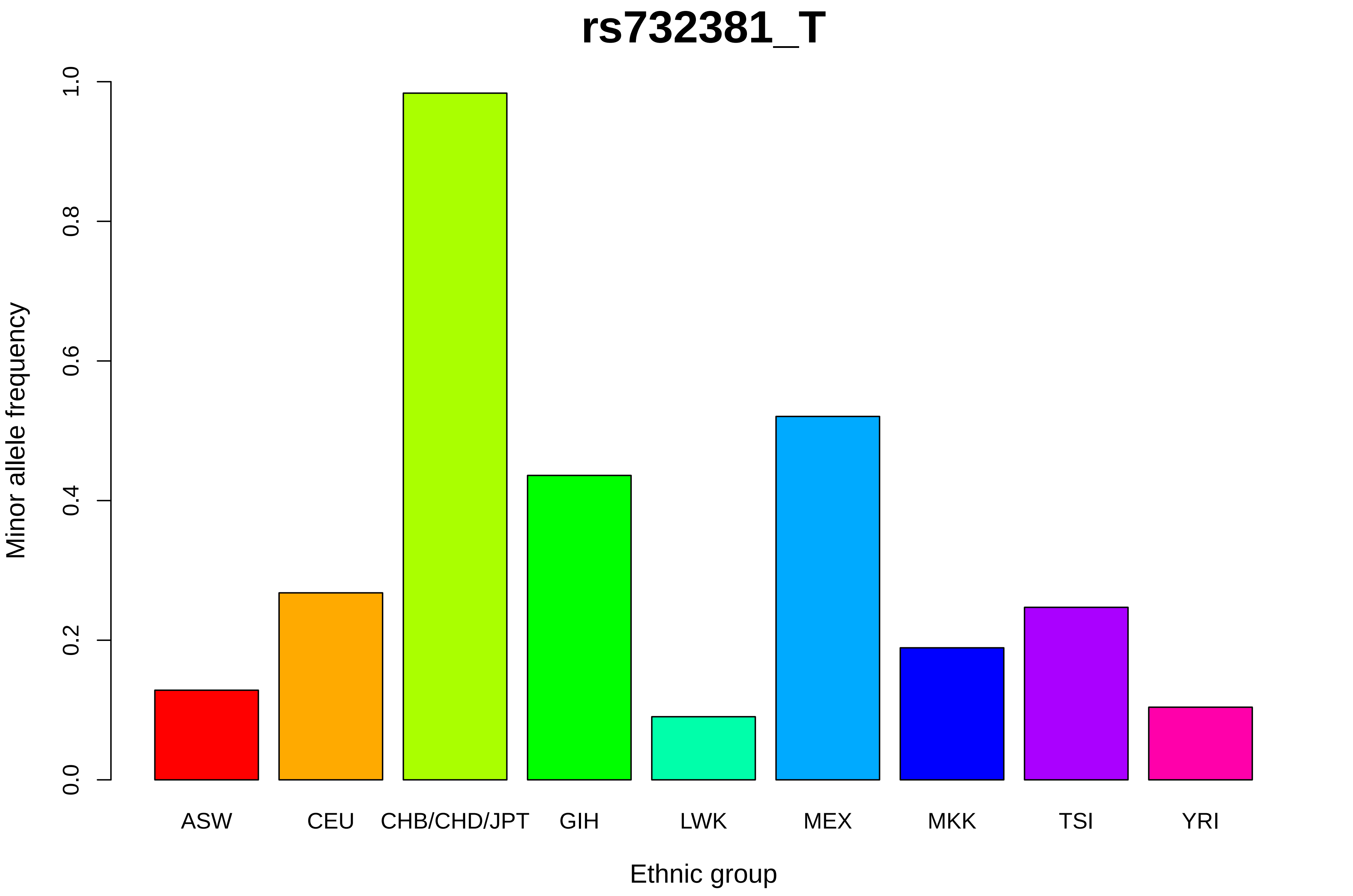
rs732381_T
Minor allele frequency
0.0
0.2
0.4
0.6
0.8
1.0
ASW
CEU
CHB/CHD/JPT
GIH
LWK
MEX
MKK
TSI
YRI
Ethnic group

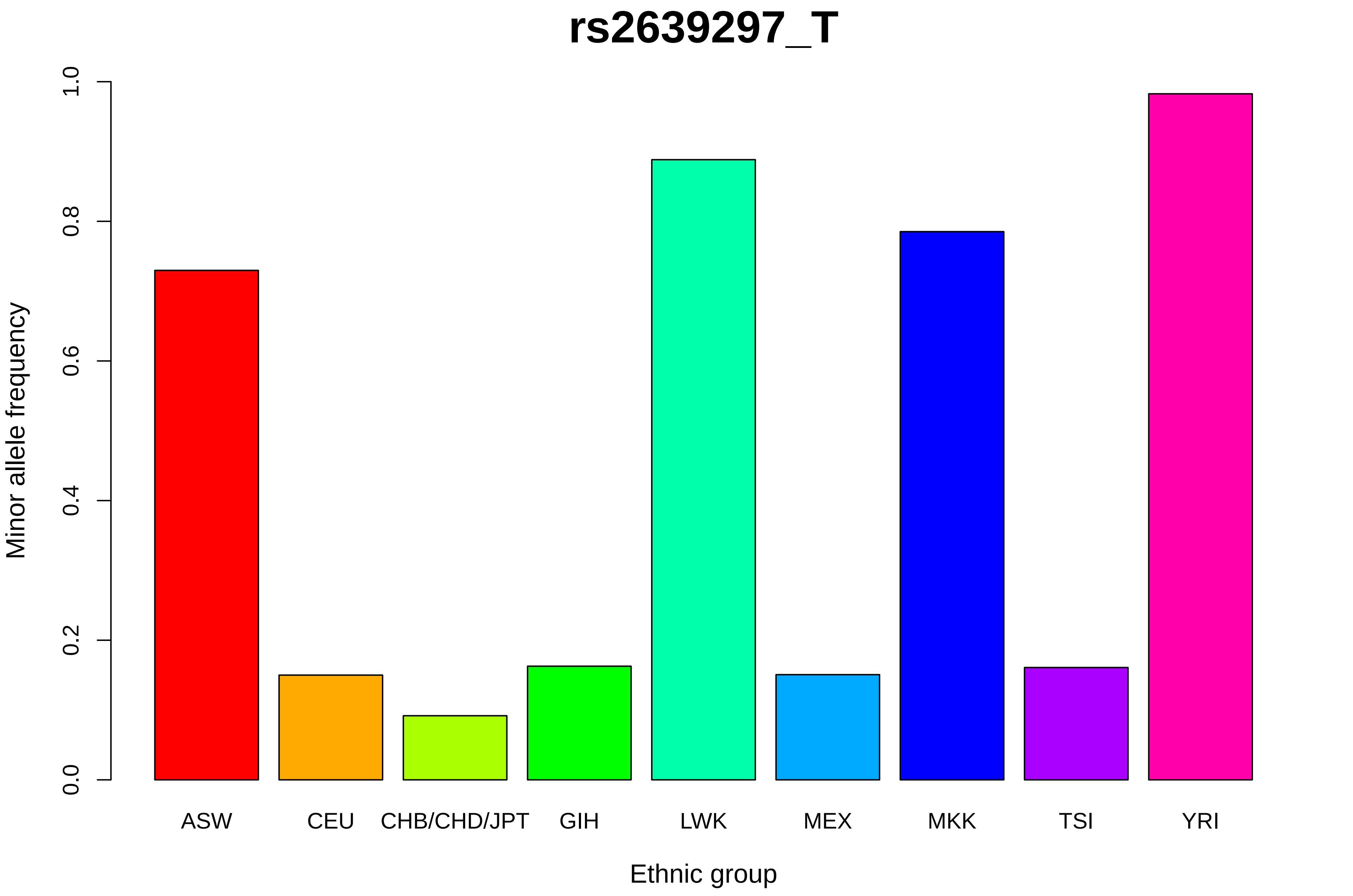
rs2639297_T
Minor allele frequency
0.0
0.2
0.4
0.6
0.8
1.0
ASW
CEU
CHB/CHD/JPT
GIH
LWK
MEX
MKK
TSI
YRI
Ethnic group

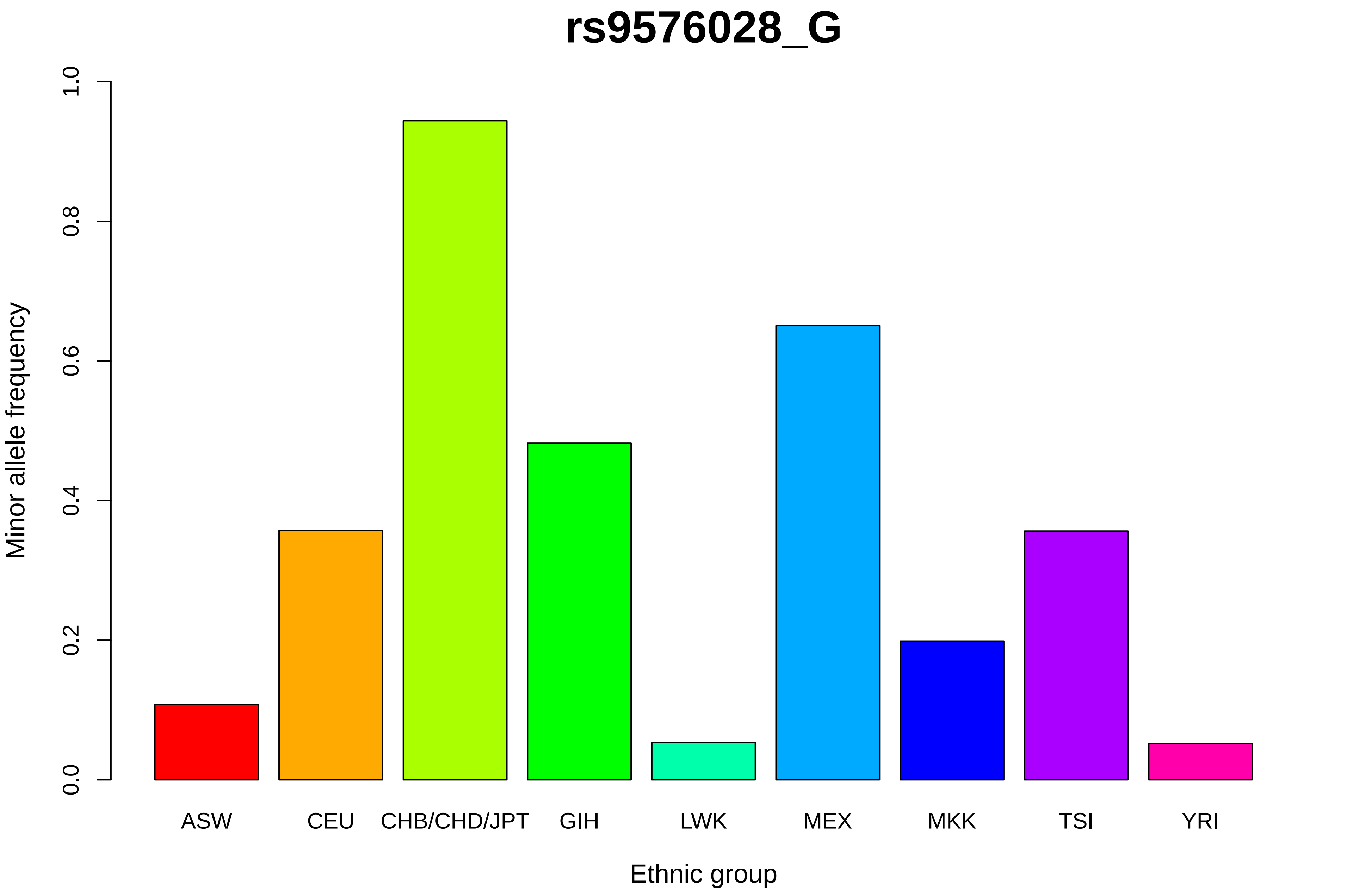
rs9576028_G
Minor allele frequency
0.0
0.2
0.4
0.6
0.8
1.0
ASW
CEU
CHB/CHD/JPT
GIH
LWK
MEX
MKK
TSI
YRI
Ethnic group

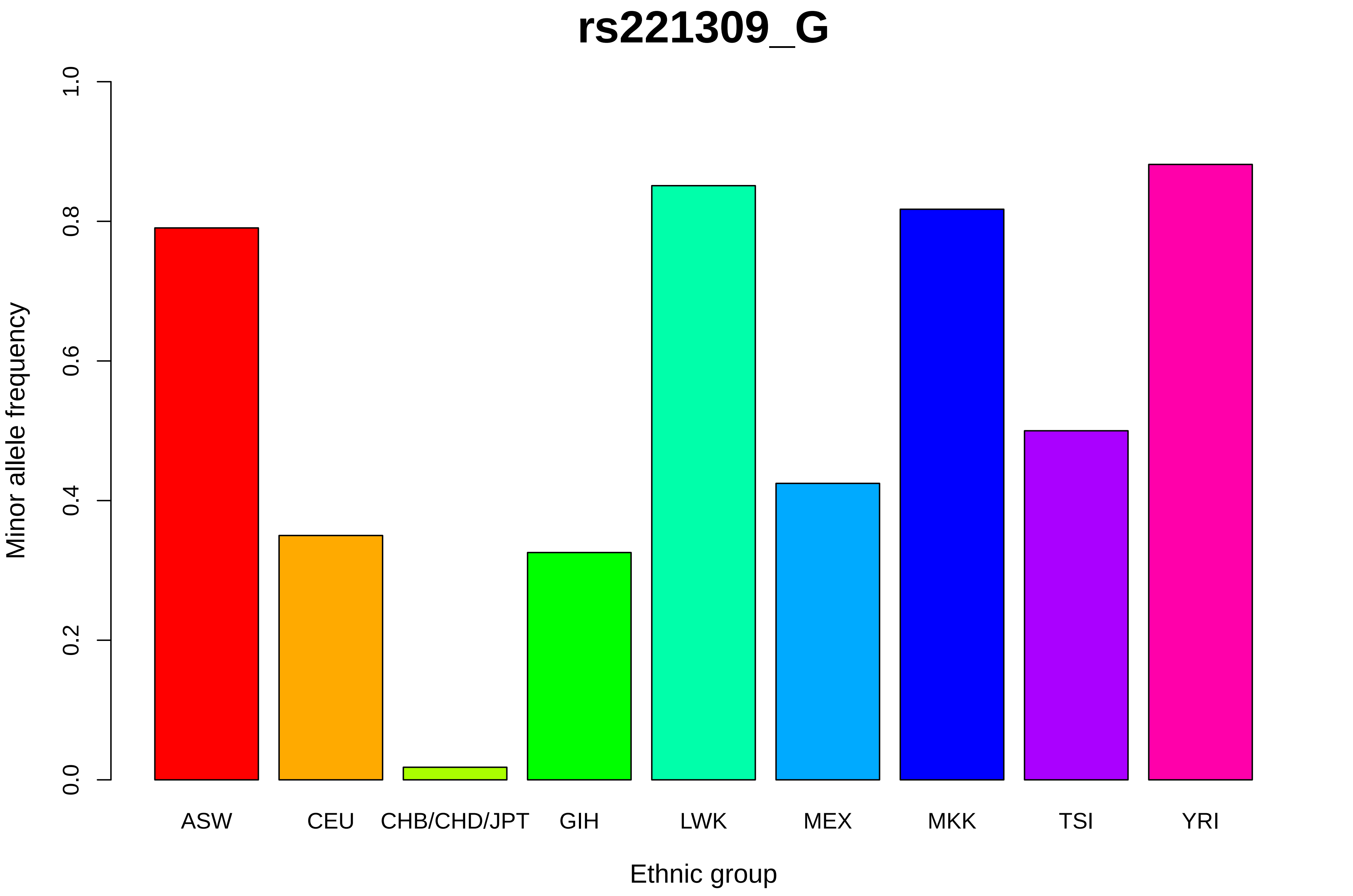
rs221309_G
Minor allele frequency
0.0
0.2
0.4
0.6
0.8
1.0
ASW
CEU
CHB/CHD/JPT
GIH
LWK
MEX
MKK
TSI
YRI
Ethnic group

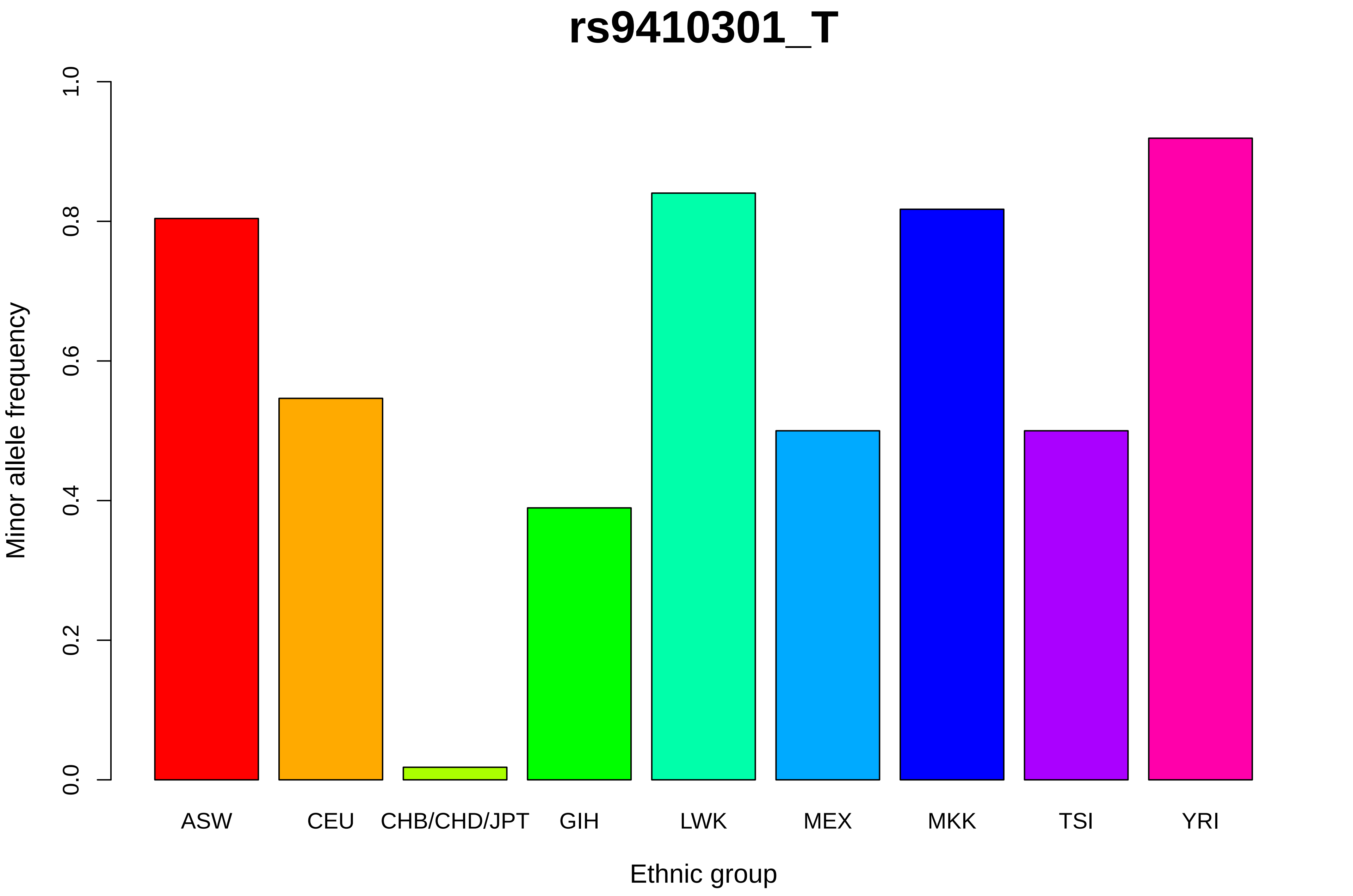
rs9410301_T
Minor allele frequency
0.0
0.2
0.4
0.6
0.8
1.0
ASW
CEU
CHB/CHD/JPT
GIH
LWK
MEX
MKK
TSI
YRI
Ethnic group

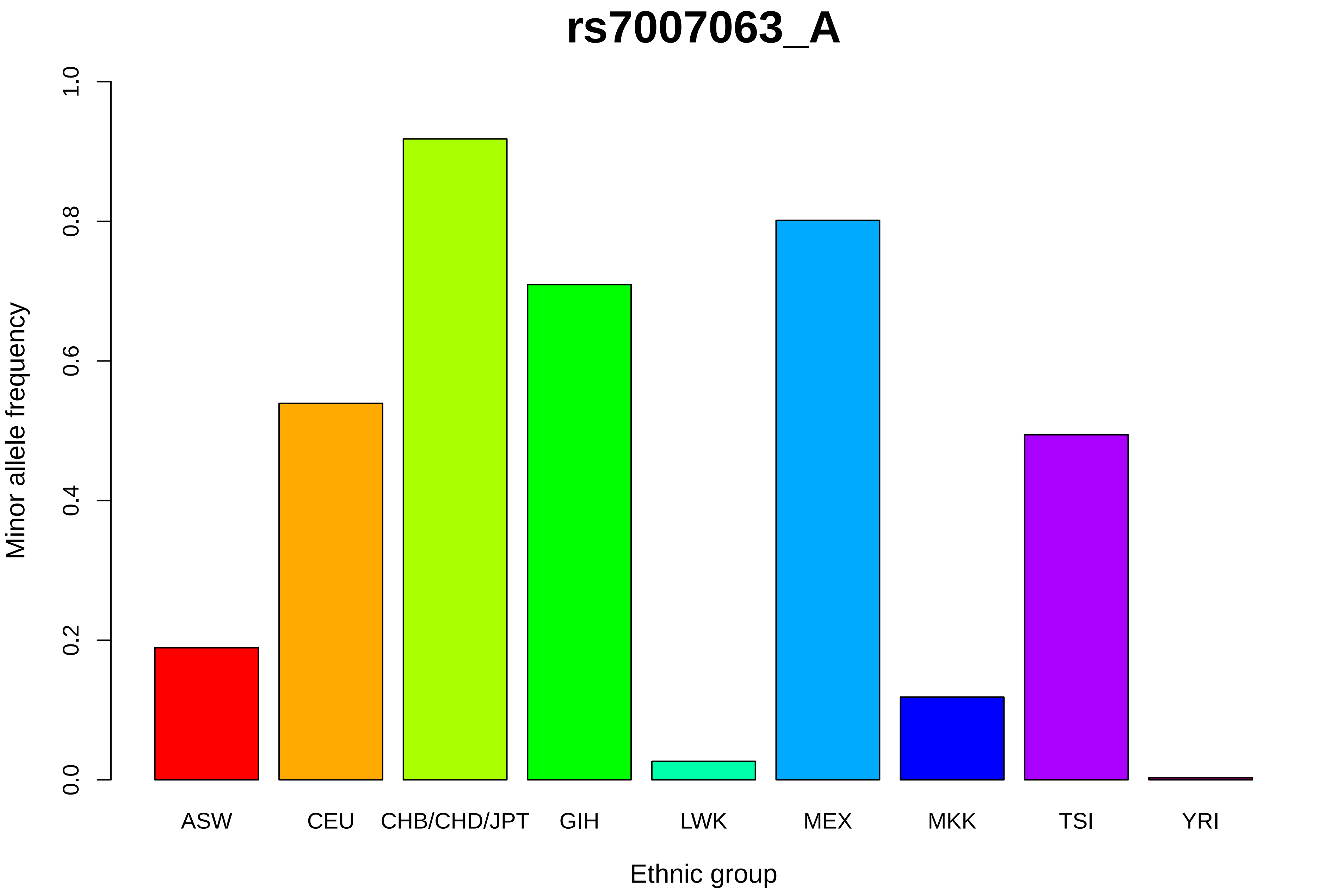

rs7007063_A
Minor allele frequency
0.0
0.2
0.4
0.6
0.8
1.0
ASW
CEU
CHB/CHD/JPT
GIH
LWK
MEX
MKK
TSI
YRI
Ethnic group

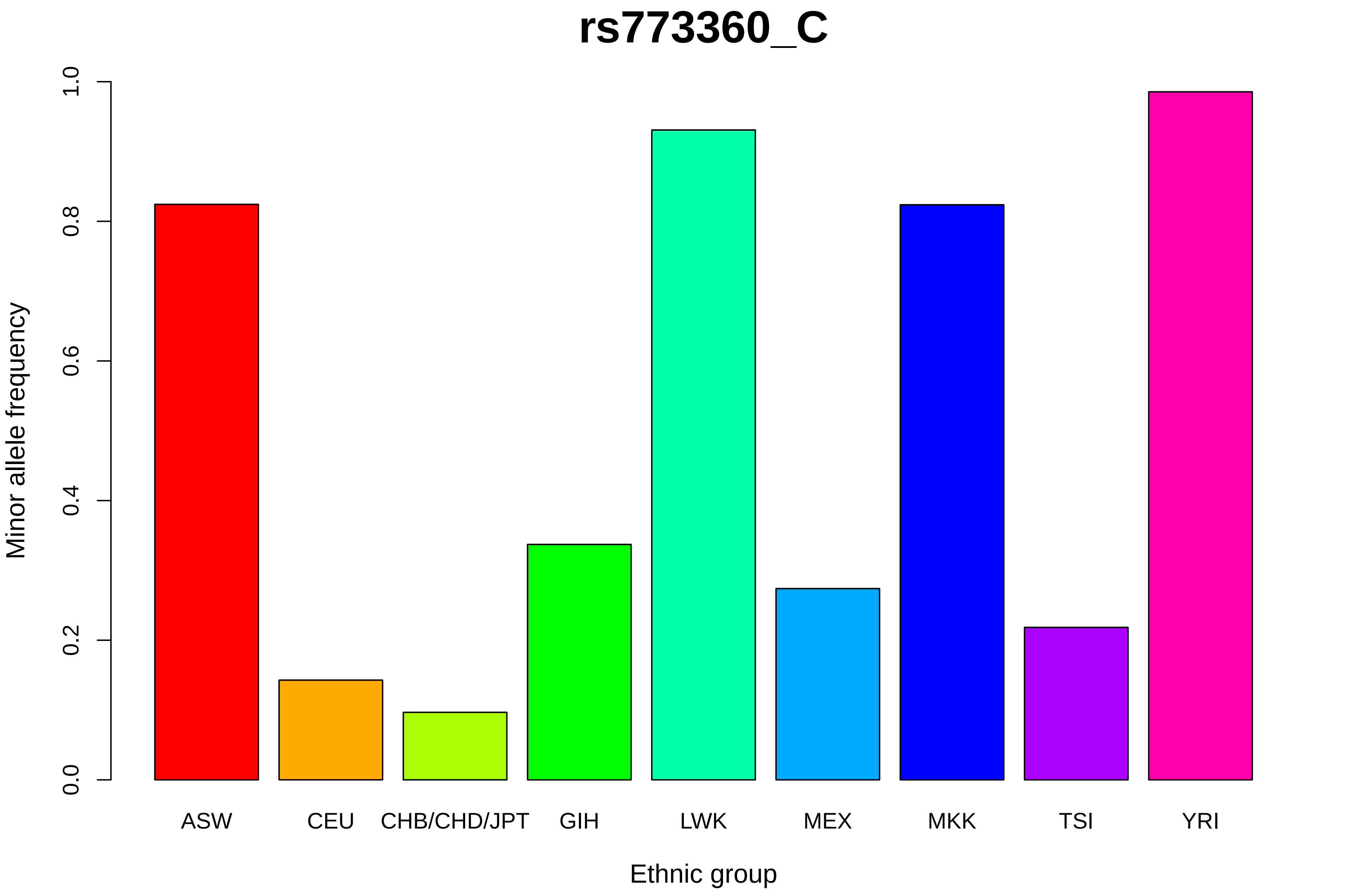
rs773360_C
Minor allele frequency
0.0
0.2
0.4
0.6
0.8
1.0
ASW
CEU
CHB/CHD/JPT
GIH
LWK
MEX
MKK
TSI
YRI
Ethnic group

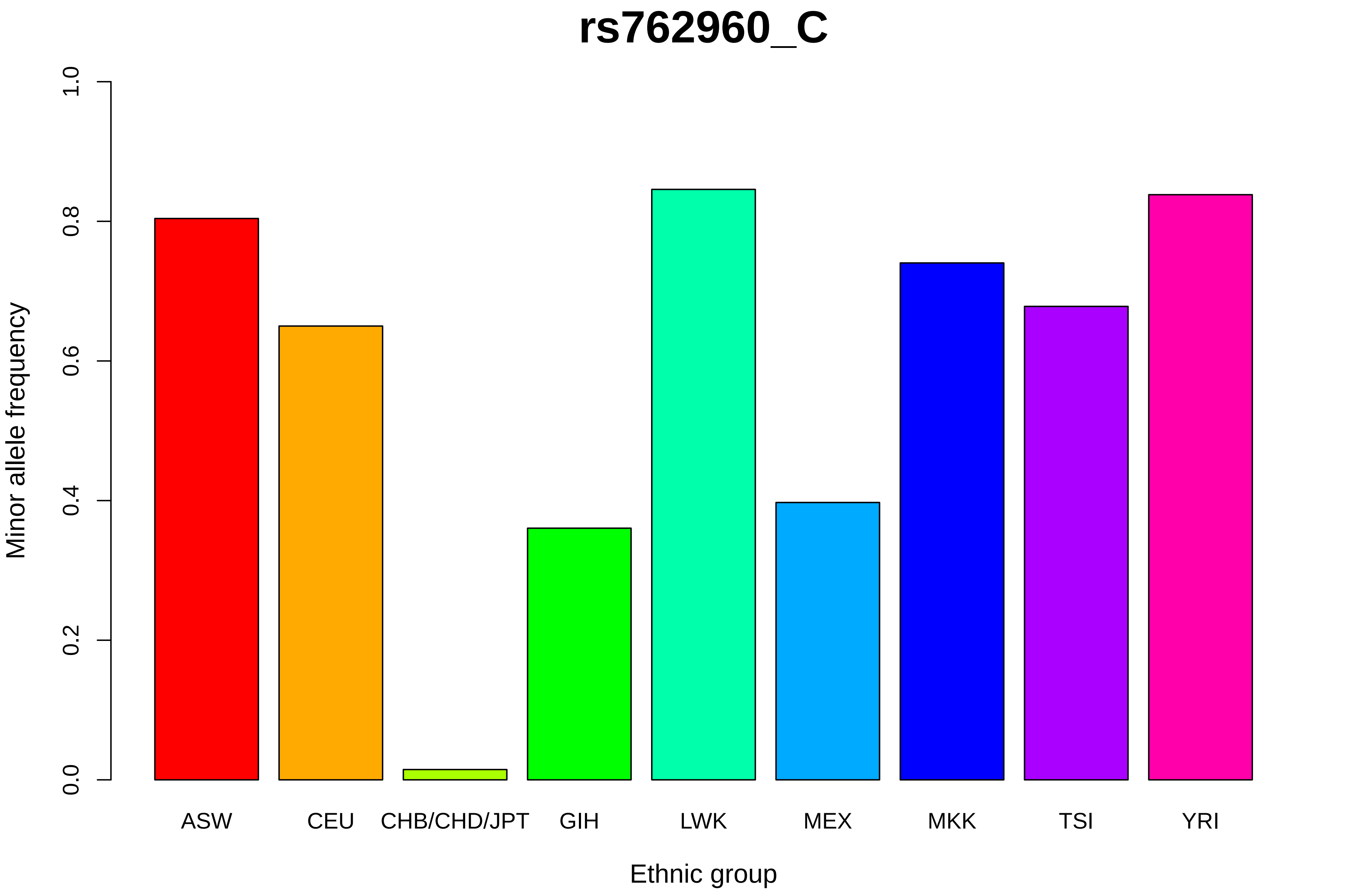

rs762960_C
Minor allele frequency
0.0
0.2
0.4
0.6
0.8
1.0
ASW
CEU
CHB/CHD/JPT
GIH
LWK
MEX
MKK
TSI
YRI
Ethnic group

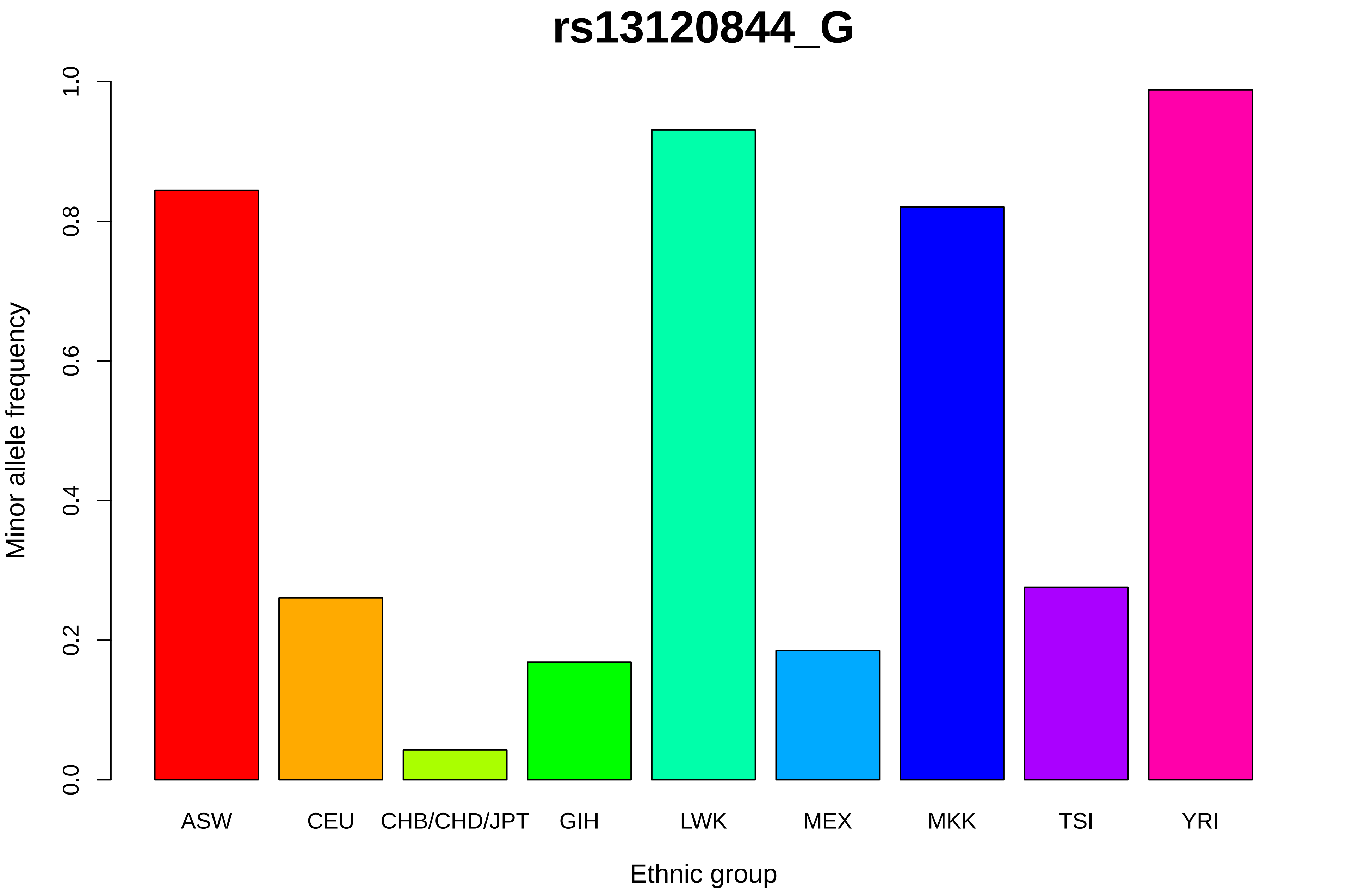

rs13120844_G
Minor allele frequency
0.0
0.2
0.4
0.6
0.8
1.0
ASW
CEU
CHB/CHD/JPT
GIH
LWK
MEX
MKK
TSI
YRI
Ethnic group

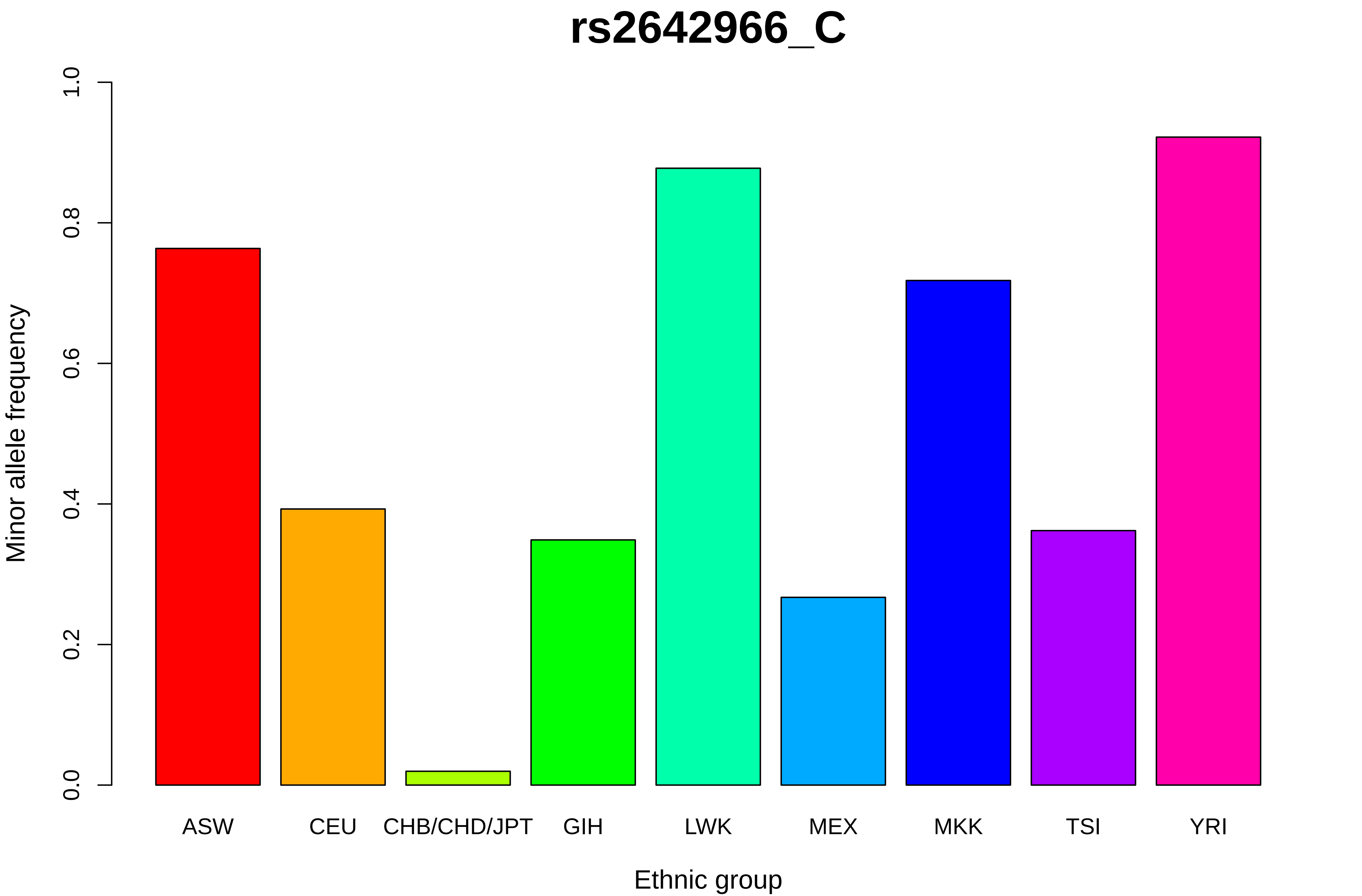

rs2642966_C
Minor allele frequency
0.0
0.2
0.4
0.6
0.8
1.0
ASW
CEU
CHB/CHD/JPT
GIH
LWK
MEX
MKK
TSI
YRI
Ethnic group

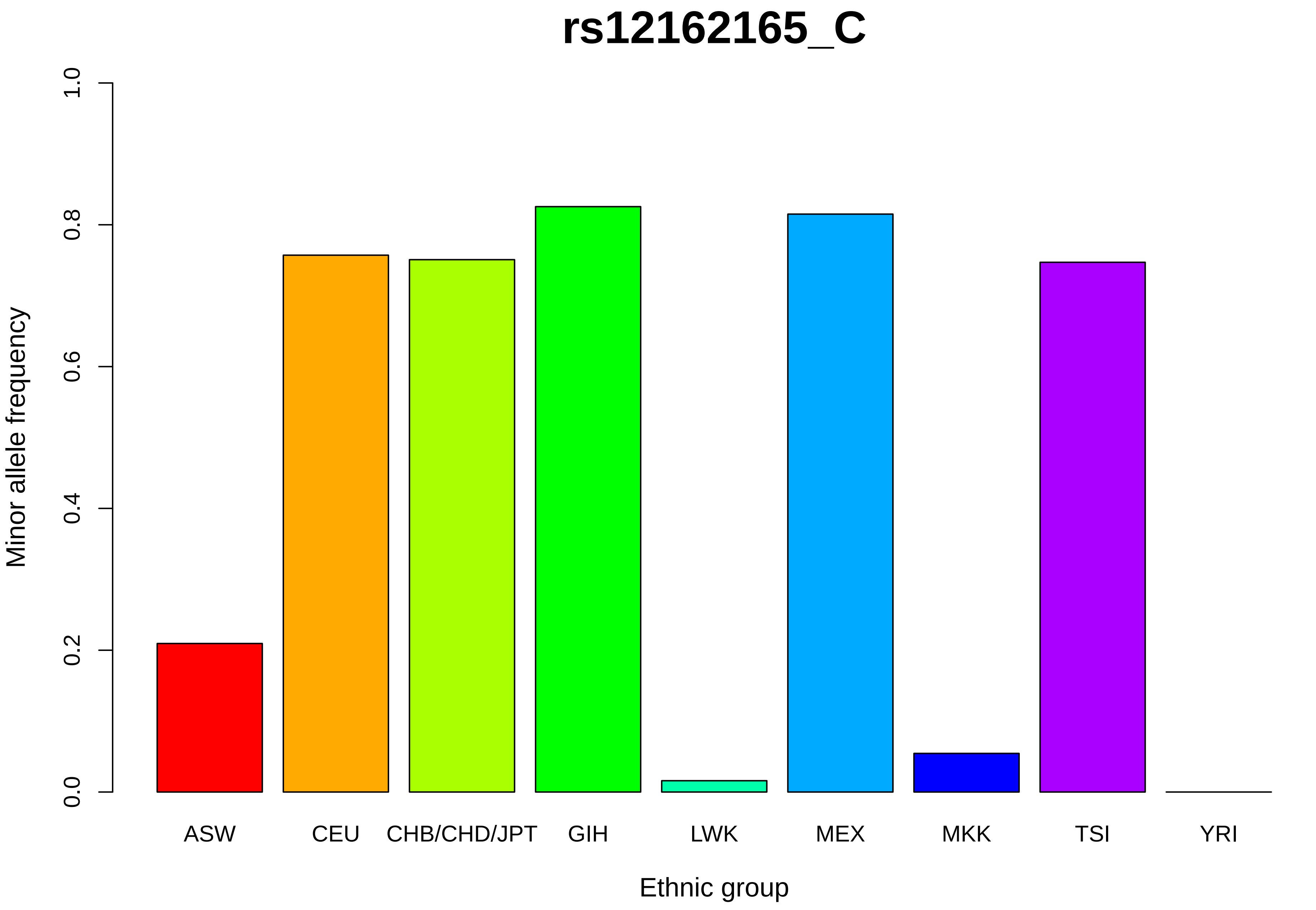
rs12162165_C
Minor allele frequency
0.0
0.2
0.4
0.6
0.8
1.0
ASW
CEU
CHB/CHD/JPT
GIH
LWK
MEX
MKK
TSI
YRI
Ethnic group

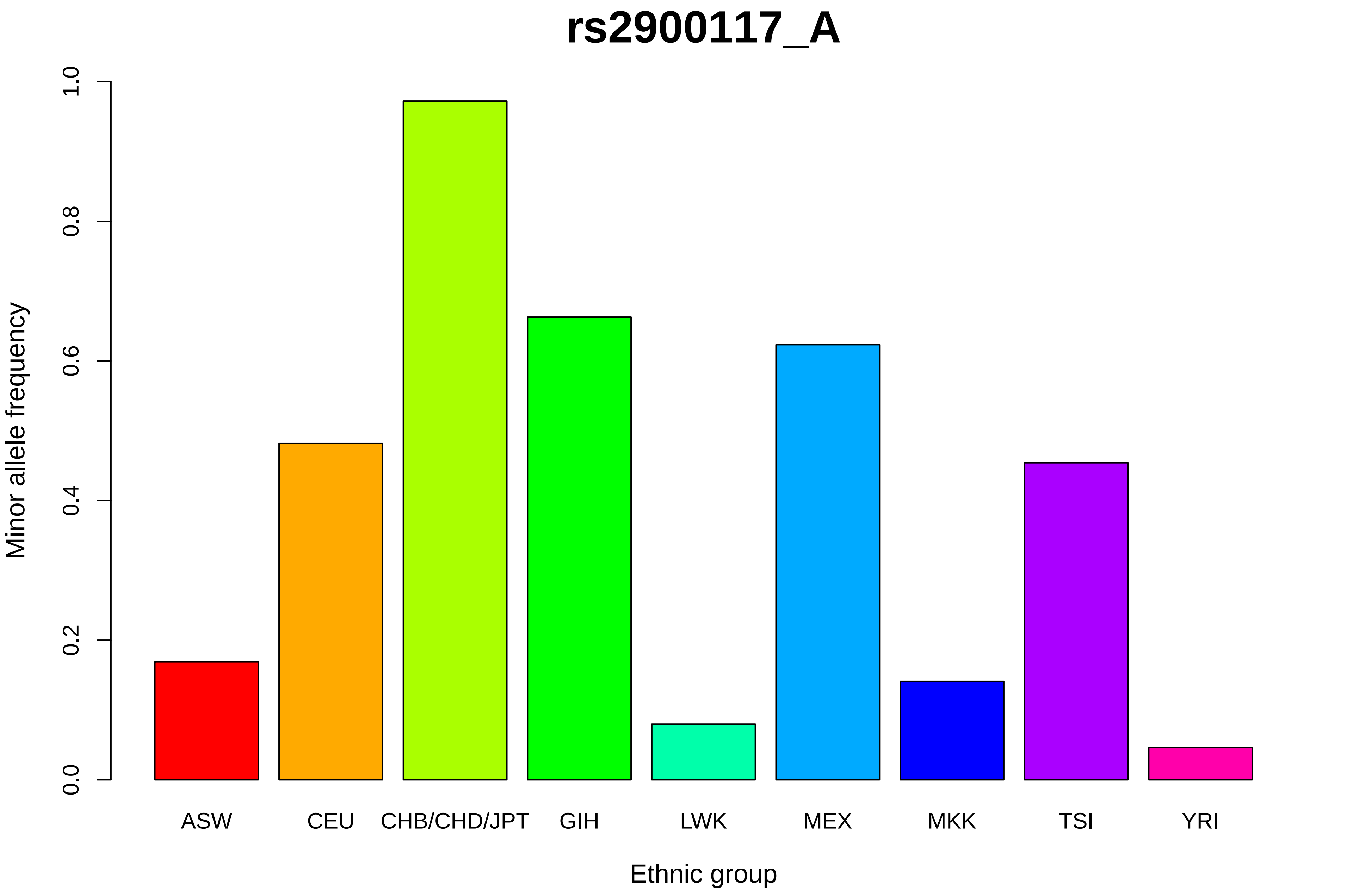
rs2900117_A
Minor allele frequency
0.0
0.2
0.4
0.6
0.8
1.0
ASW
CEU
CHB/CHD/JPT
GIH
LWK
MEX
MKK
TSI
YRI
Ethnic group

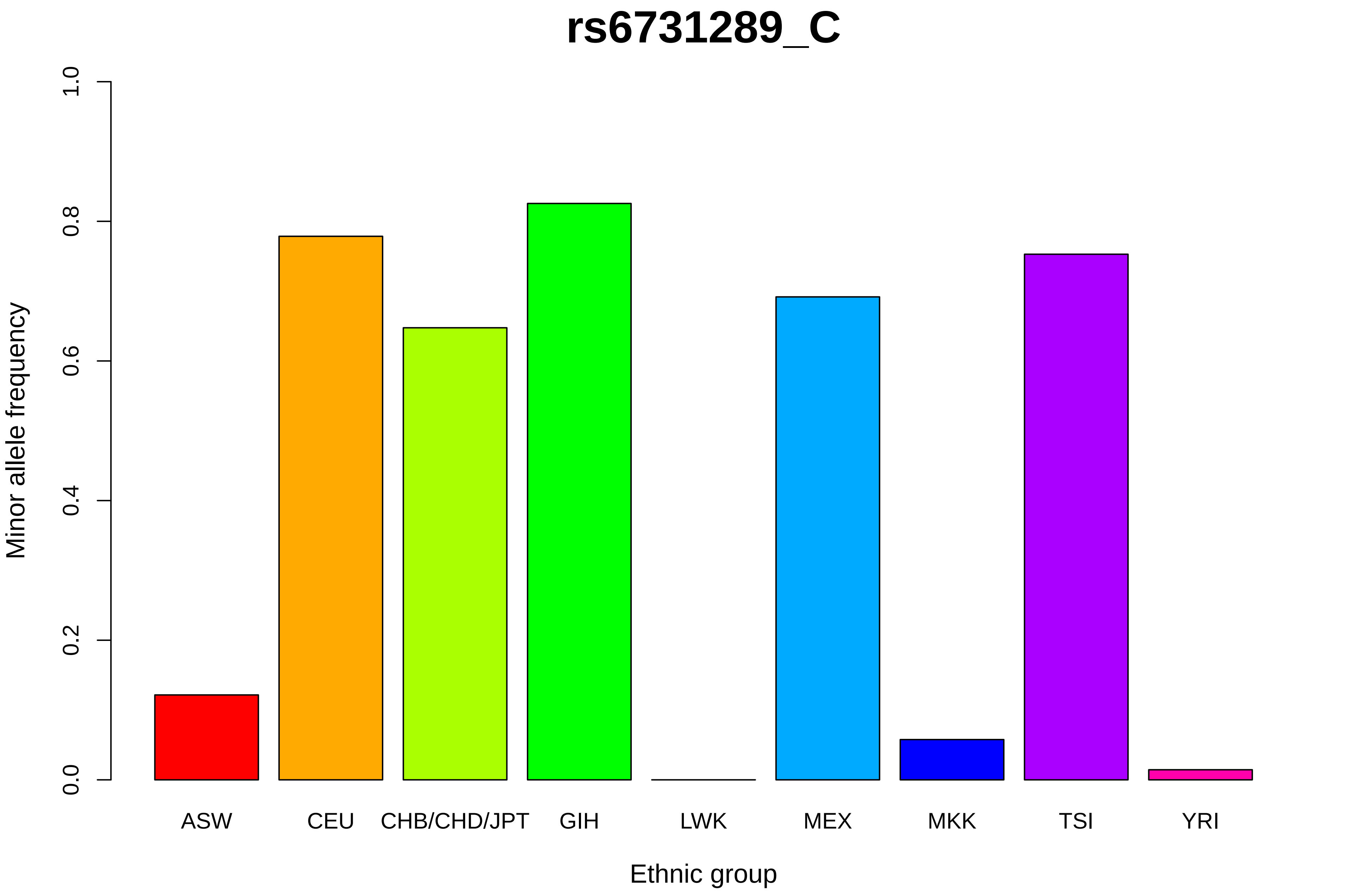
rs6731289_C
Minor allele frequency
0.0
0.2
0.4
0.6
0.8
1.0
ASW
CEU
CHB/CHD/JPT
GIH
LWK
MEX
MKK
TSI
YRI
Ethnic group

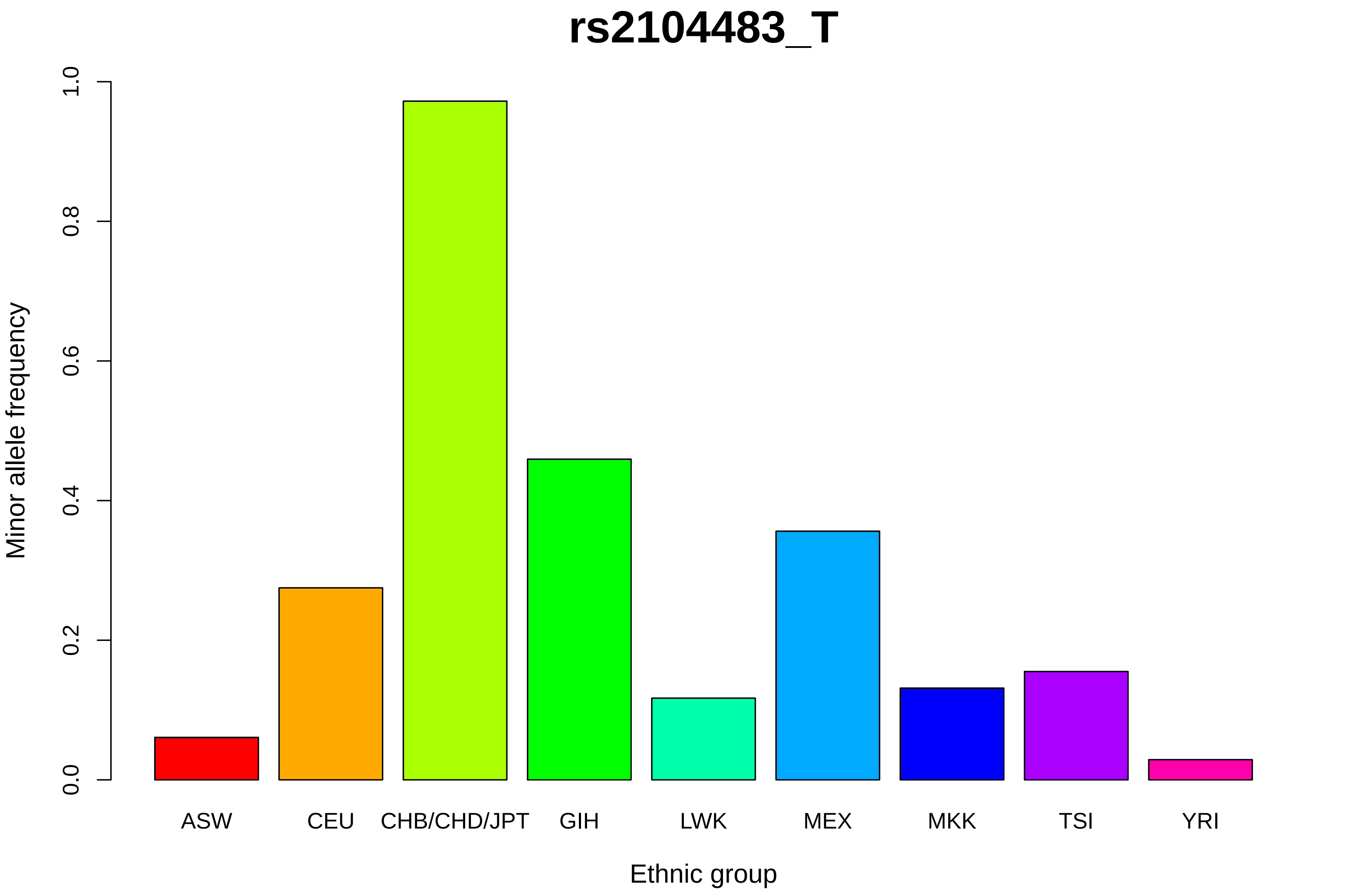
rs2104483_T
Minor allele frequency
0.0
0.2
0.4
0.6
0.8
1.0
ASW
CEU
CHB/CHD/JPT
GIH
LWK
MEX
MKK
TSI
YRI
Ethnic group

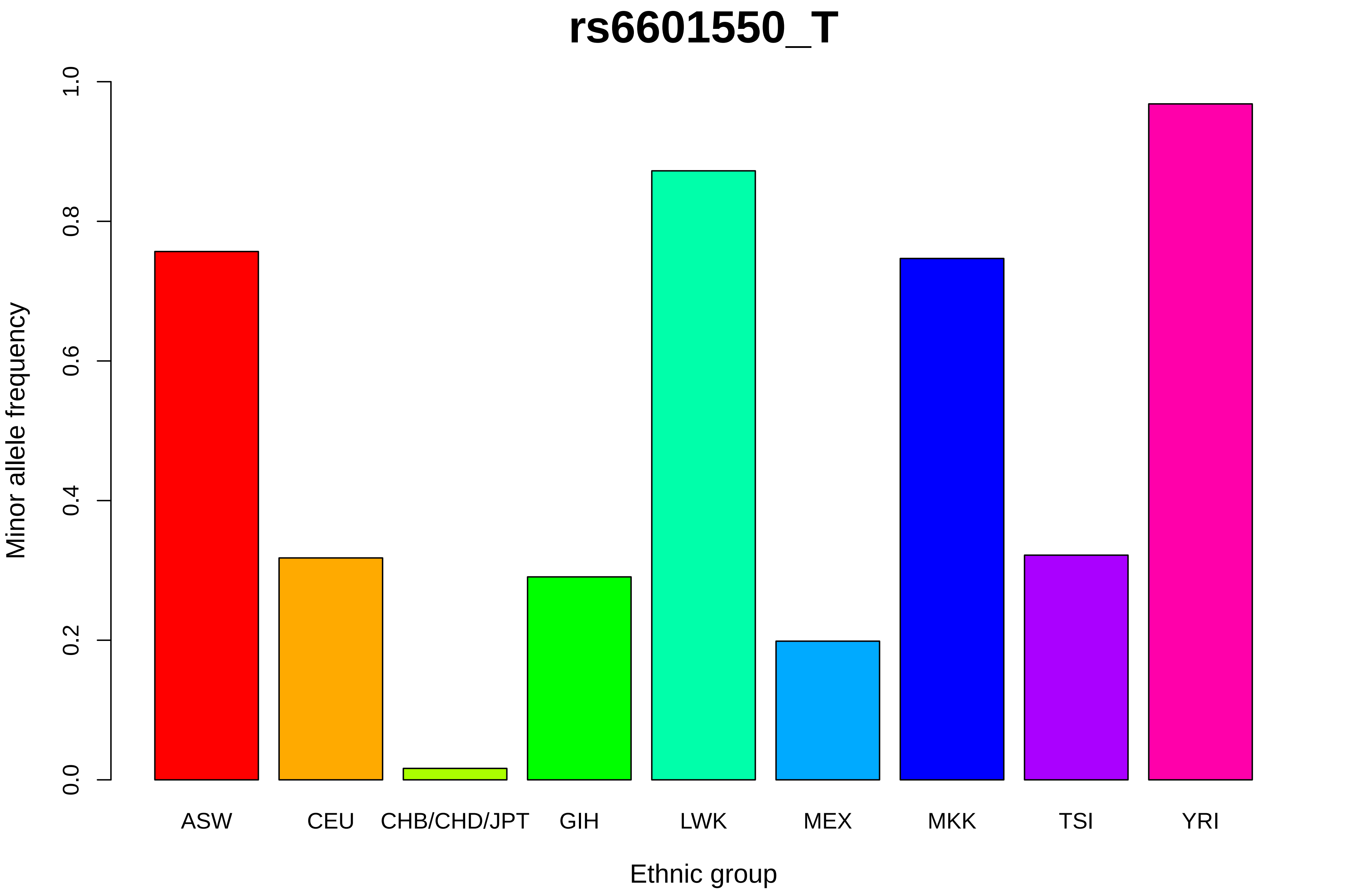
rs6601550_T
Minor allele frequency
0.0
0.2
0.4
0.6
0.8
1.0
ASW
CEU
CHB/CHD/JPT
GIH
LWK
MEX
MKK
TSI
YRI
Ethnic group

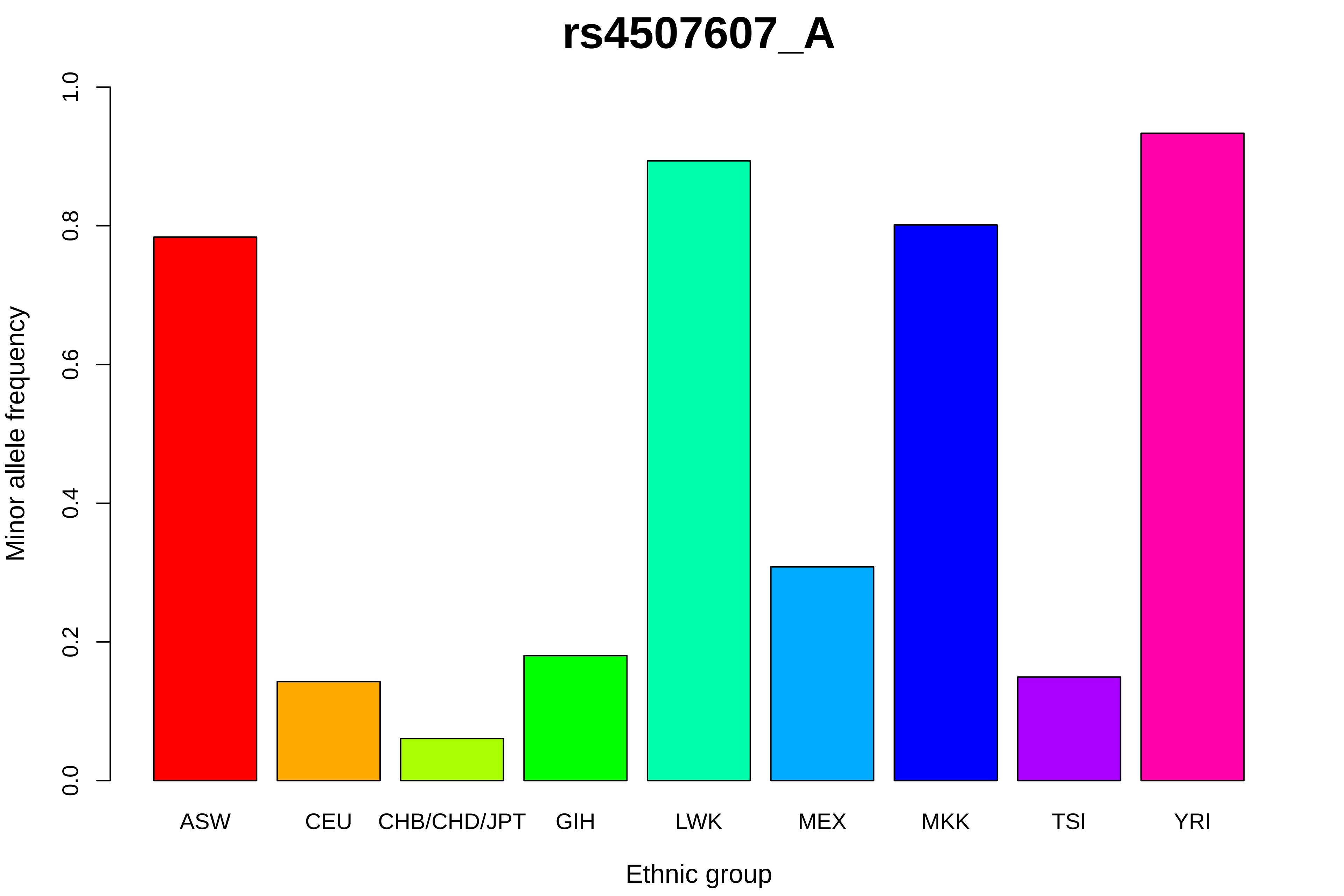
rs4507607_A
Minor allele frequency
0.0
0.2
0.4
0.6
0.8
1.0
ASW
CEU
CHB/CHD/JPT
GIH
LWK
MEX
MKK
TSI
YRI
Ethnic group

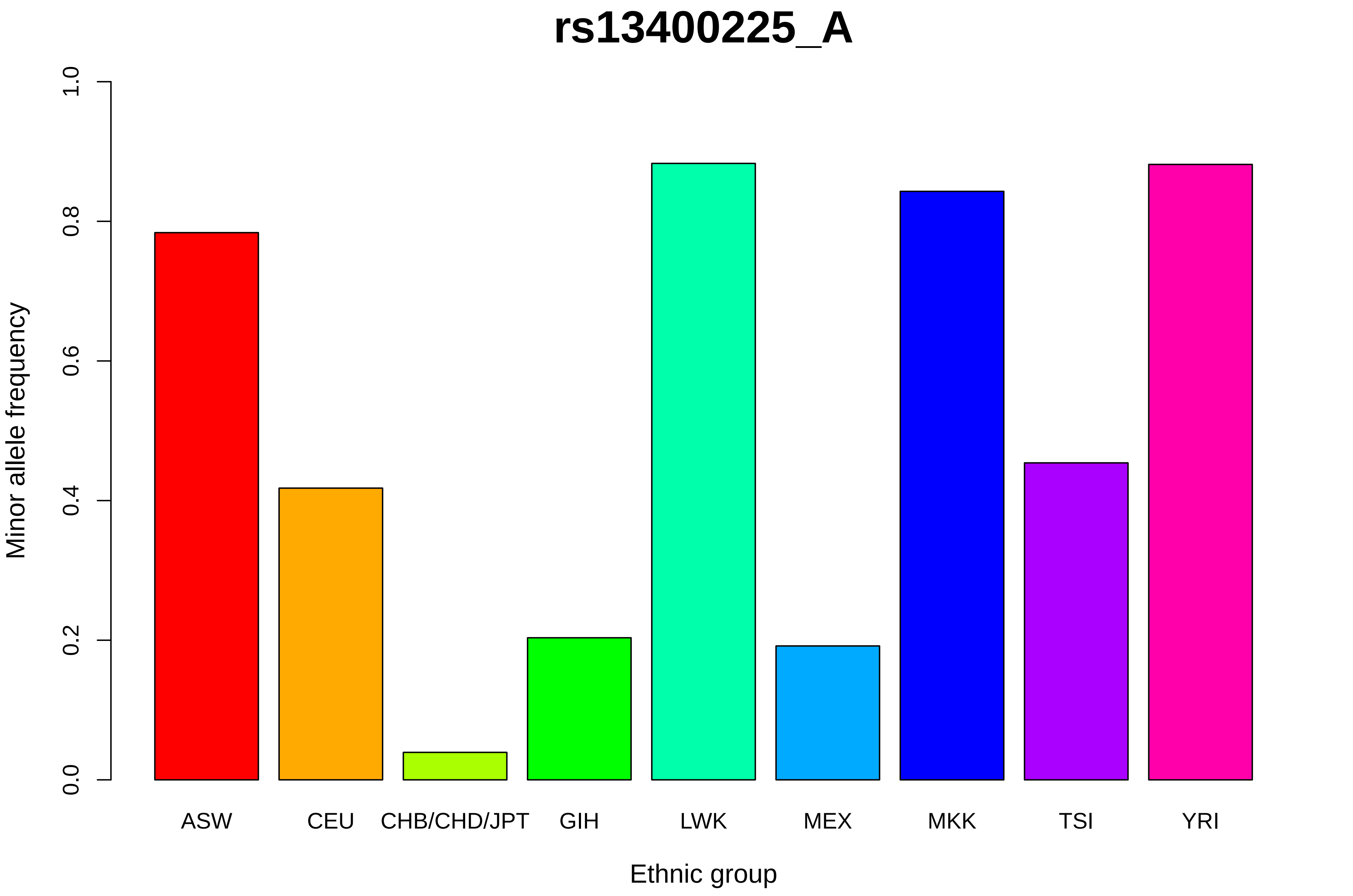
rs13400225_A
Minor allele frequency
0.0
0.2
0.4
0.6
0.8
1.0
ASW
CEU
CHB/CHD/JPT
GIH
LWK
MEX
MKK
TSI
YRI
Ethnic group

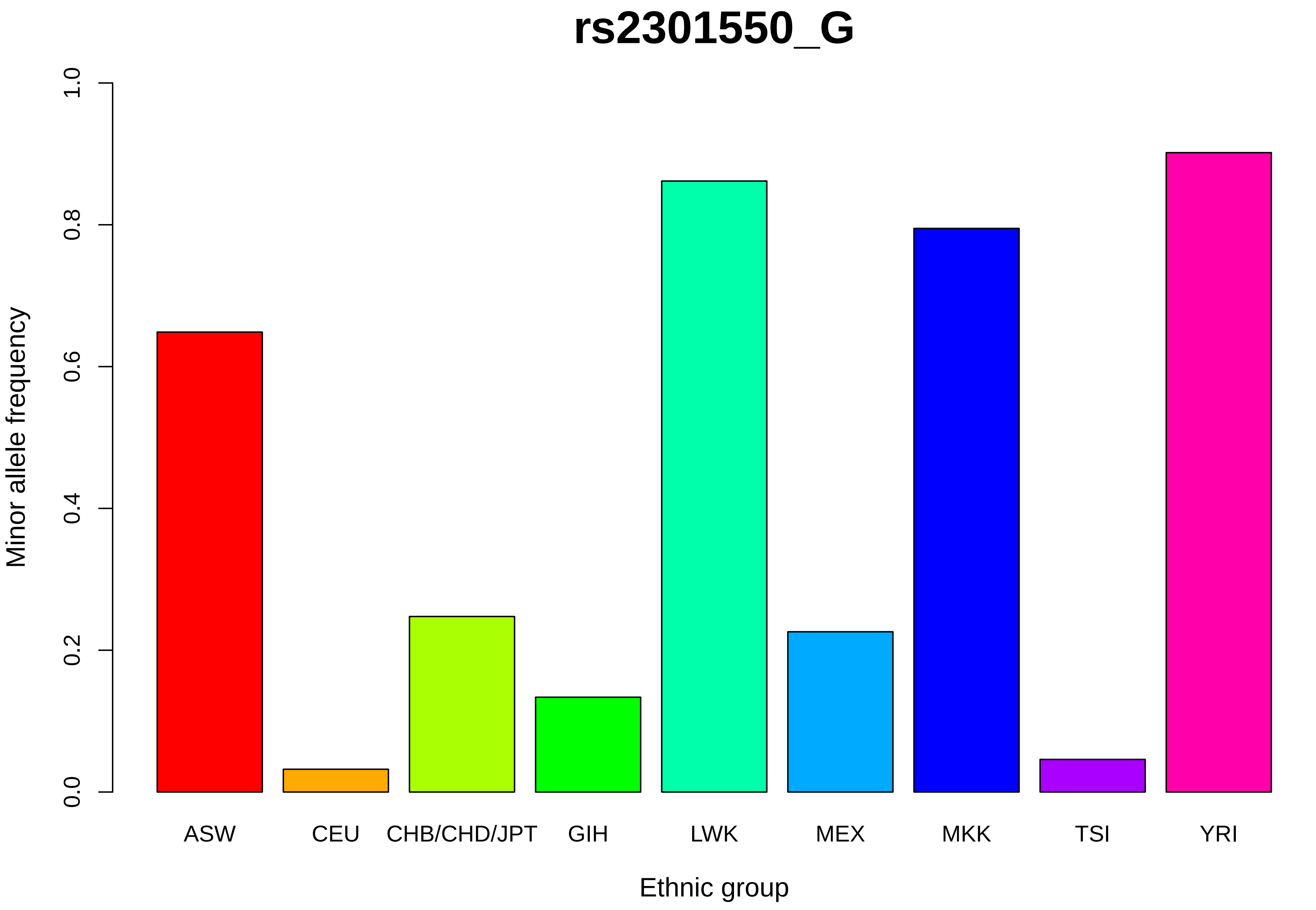

rs2301550_G
Minor allele frequency
0.0
0.2
0.4
0.6
0.8
1.0
ASW
CEU
CHB/CHD/JPT
GIH
LWK
MEX
MKK
TSI
YRI
Ethnic group

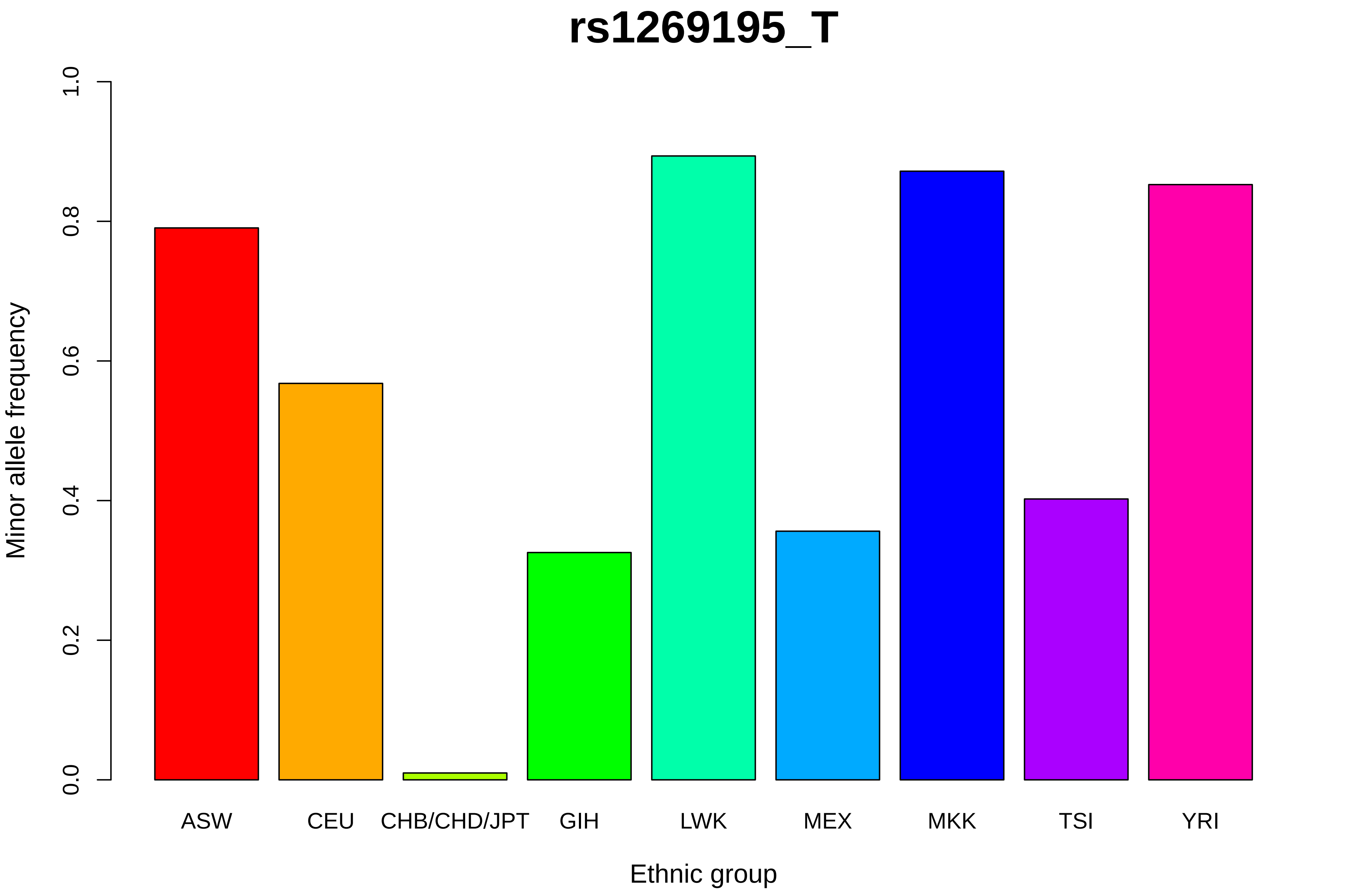
rs1269195_T
Minor allele frequency
0.0
0.2
0.4
0.6
0.8
1.0
ASW
CEU
CHB/CHD/JPT
GIH
LWK
MEX
MKK
TSI
YRI
Ethnic group

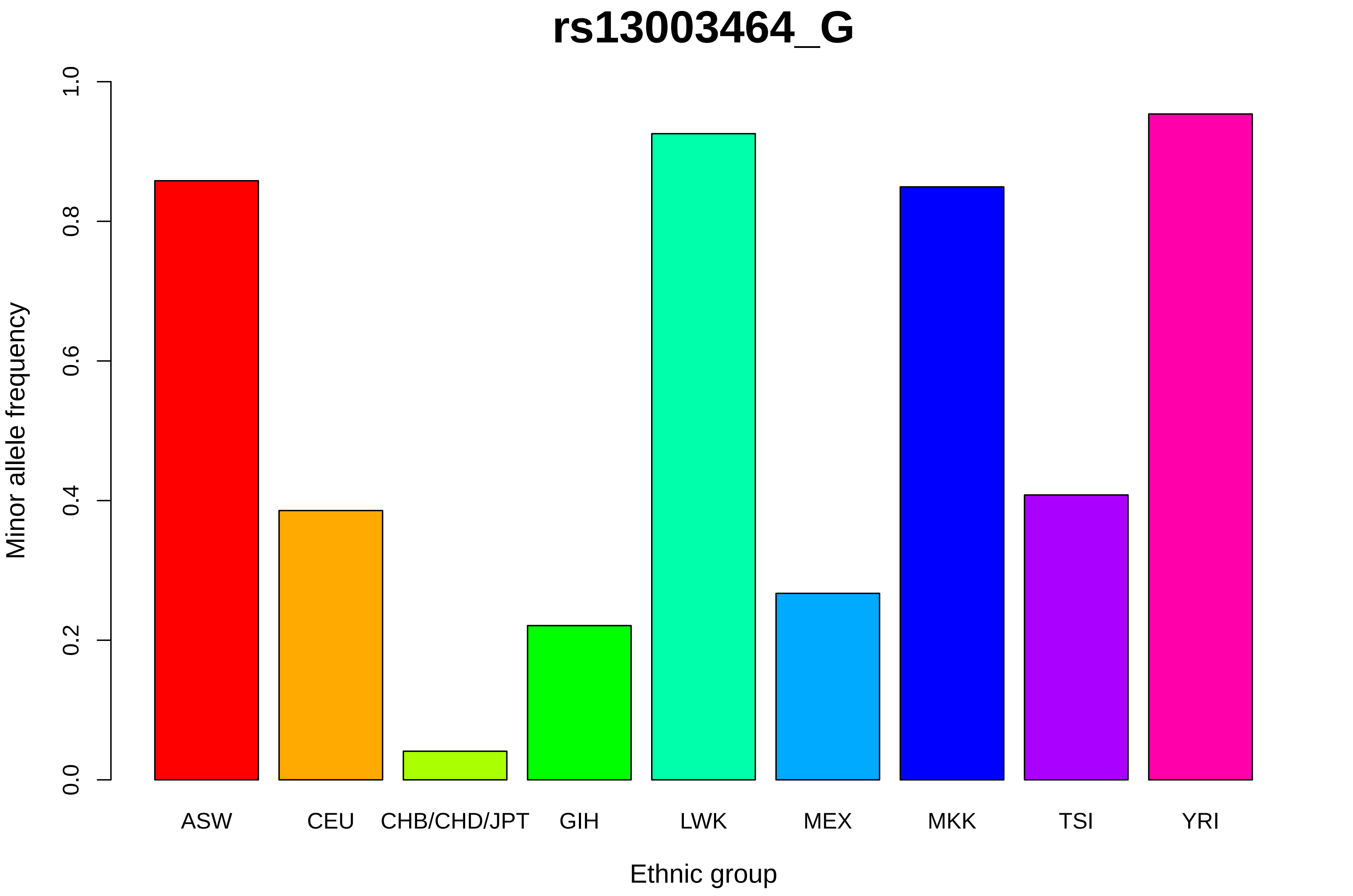

rs13003464_G
Minor allele frequency
0.0
0.2
0.4
0.6
0.8
1.0
ASW
CEU
CHB/CHD/JPT
GIH
LWK
MEX
MKK
TSI
YRI
Ethnic group

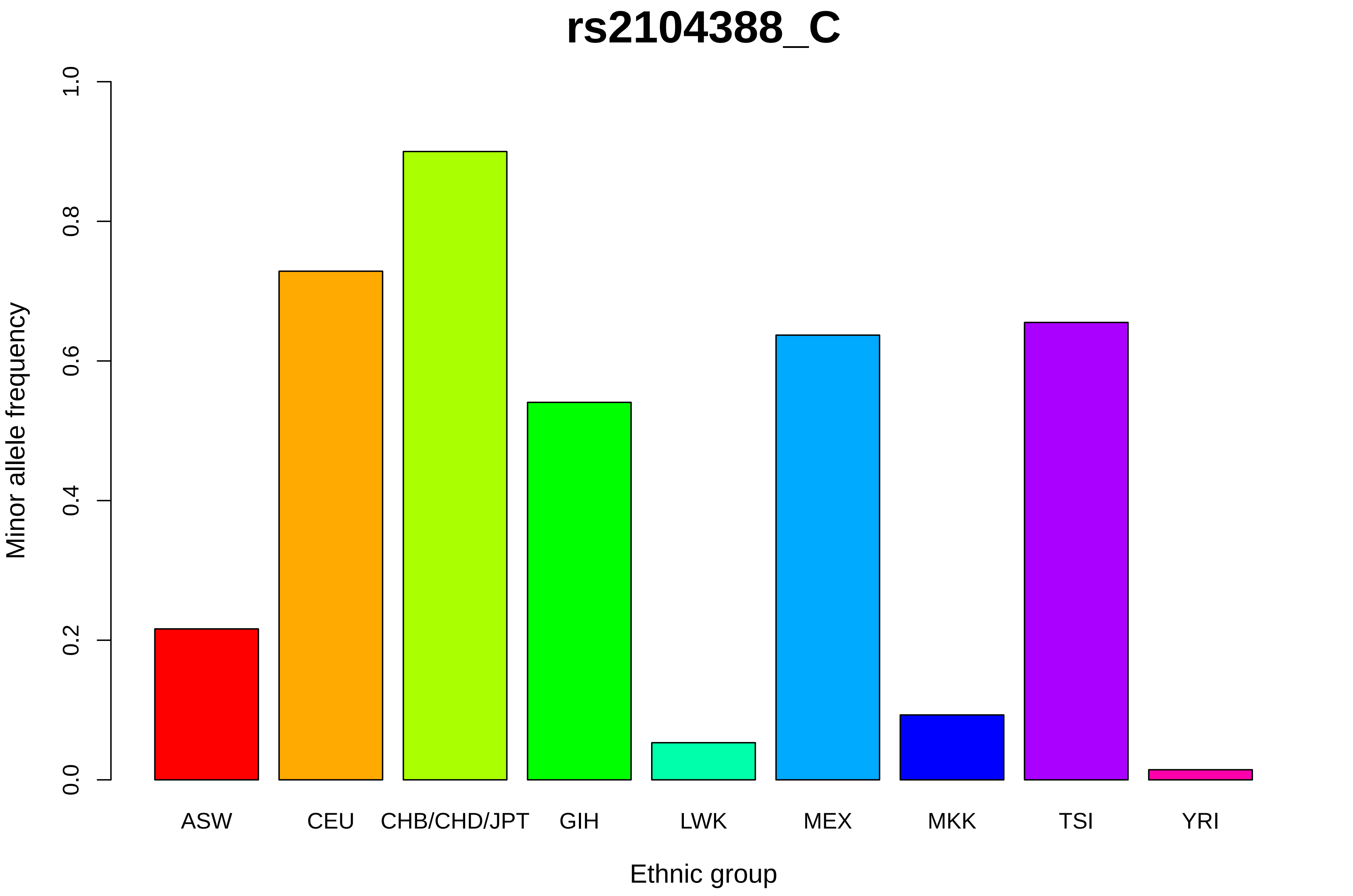
rs2104388_C
Minor allele frequency
0.0
0.2
0.4
0.6
0.8
1.0
ASW
CEU
CHB/CHD/JPT
GIH
LWK
MEX
MKK
TSI
YRI
Ethnic group

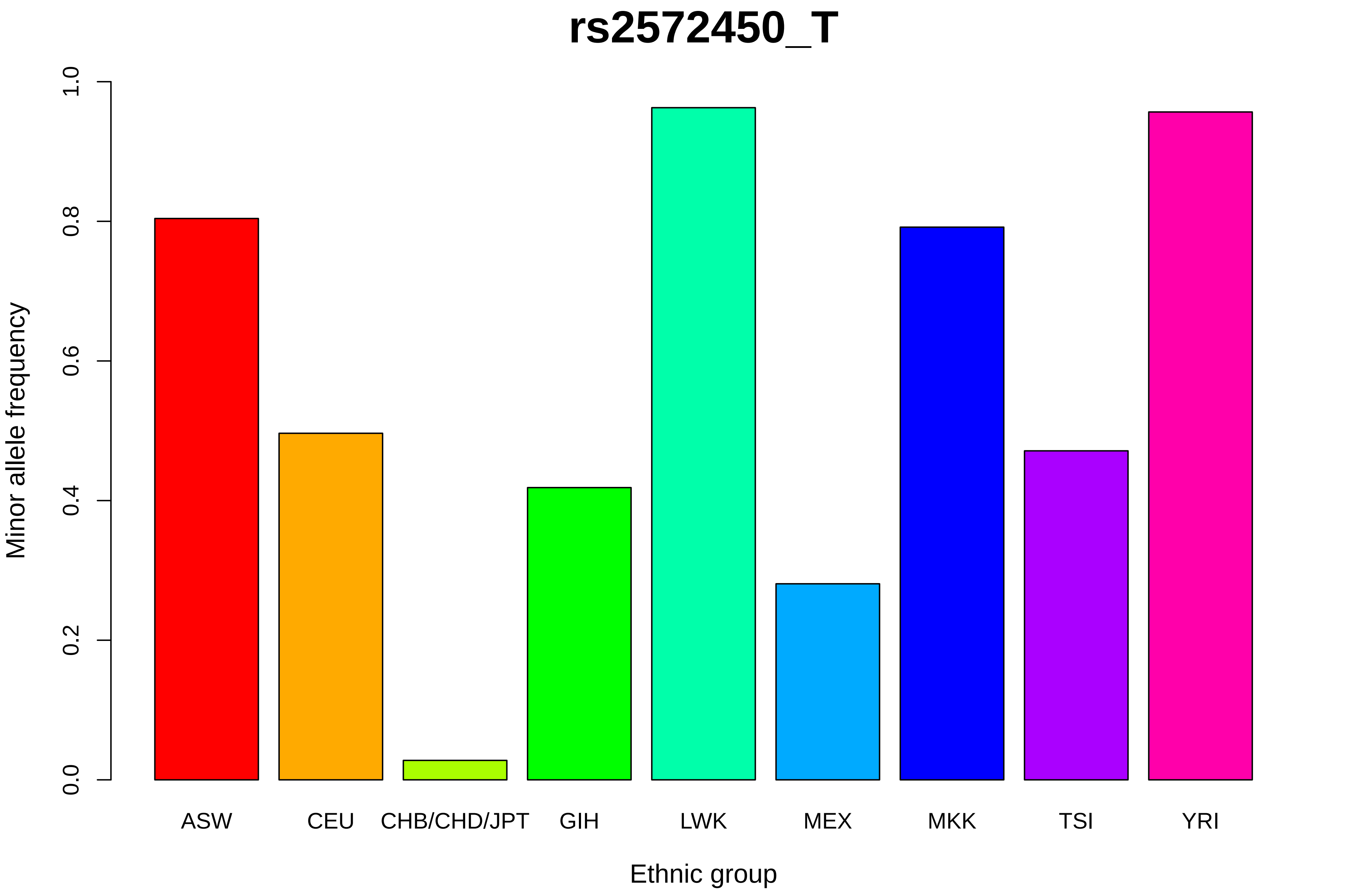
rs2572450_T
Minor allele frequency
0.0
0.2
0.4
0.6
0.8
1.0
ASW
CEU
CHB/CHD/JPT
GIH
LWK
MEX
MKK
TSI
YRI
Ethnic group

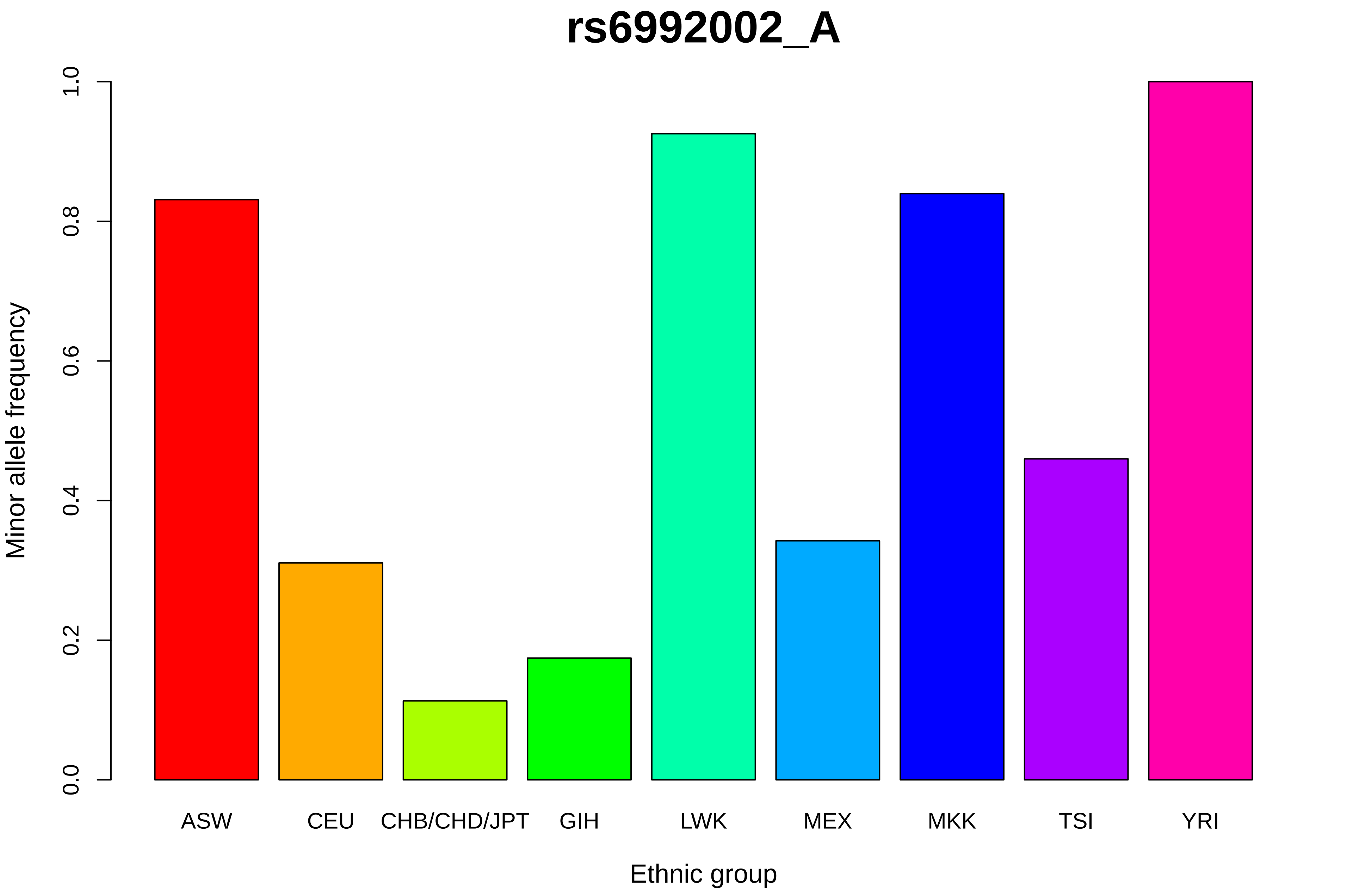
rs6992002_A
Minor allele frequency
0.0
0.2
0.4
0.6
0.8
1.0
ASW
CEU
CHB/CHD/JPT
GIH
LWK
MEX
MKK
TSI
YRI
Ethnic group

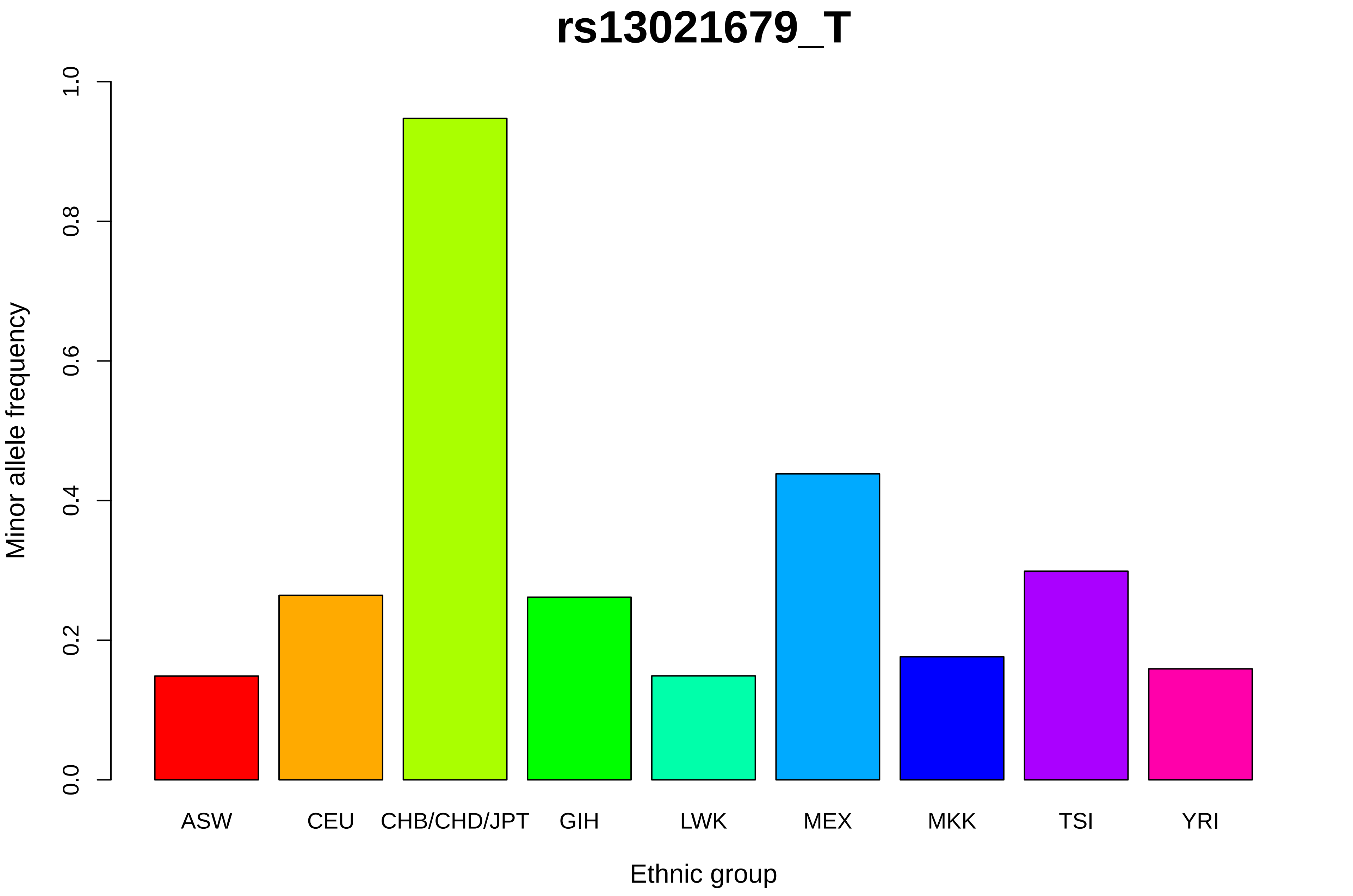
rs13021679_T
Minor allele frequency
0.0
0.2
0.4
0.6
0.8
1.0
ASW
CEU
CHB/CHD/JPT
GIH
LWK
MEX
MKK
TSI
YRI
Ethnic group

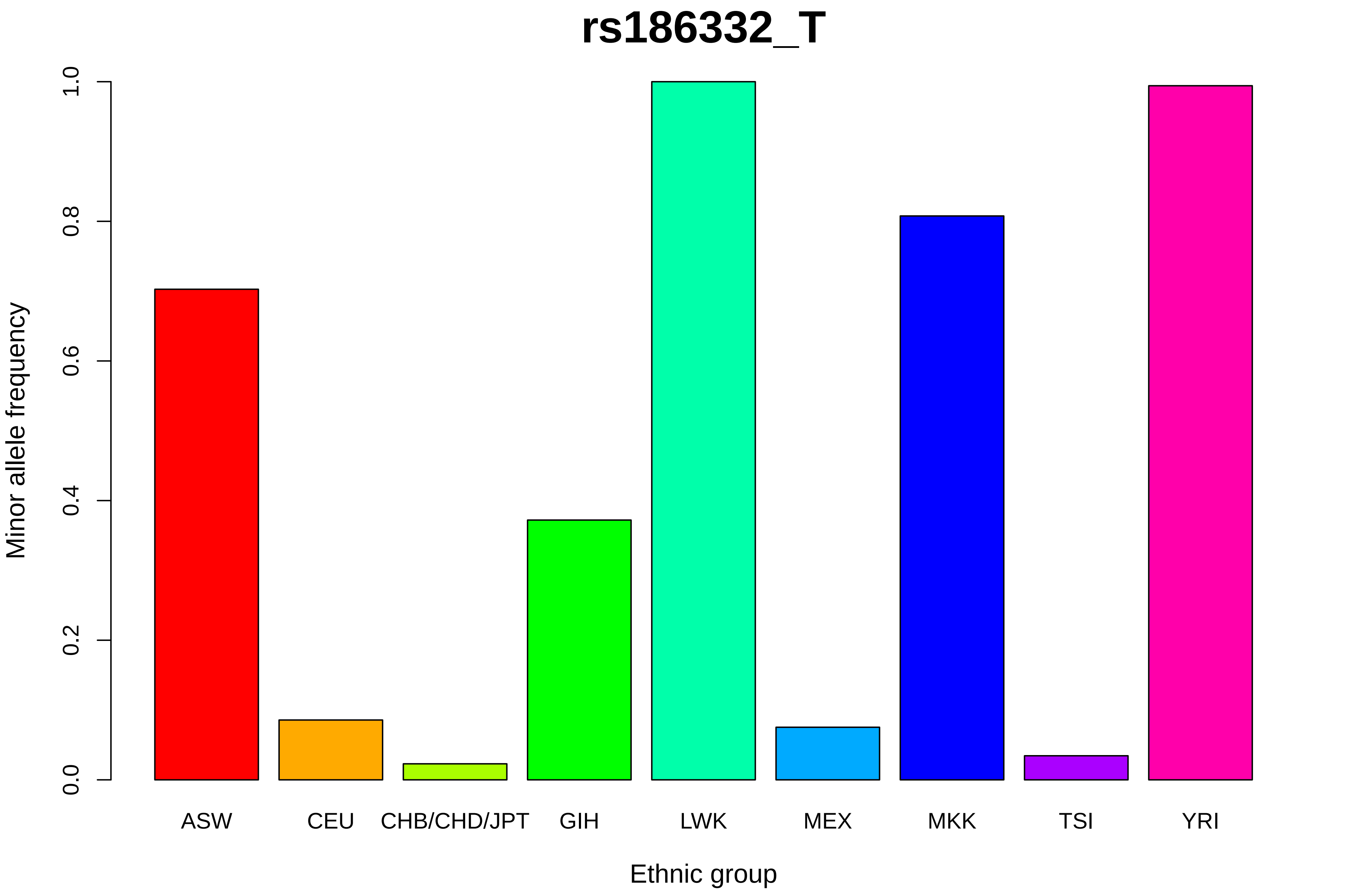

rs186332_T
Minor allele frequency
0.0
0.2
0.4
0.6
0.8
1.0
ASW
CEU
CHB/CHD/JPT
GIH
LWK
MEX
MKK
TSI
YRI
Ethnic group

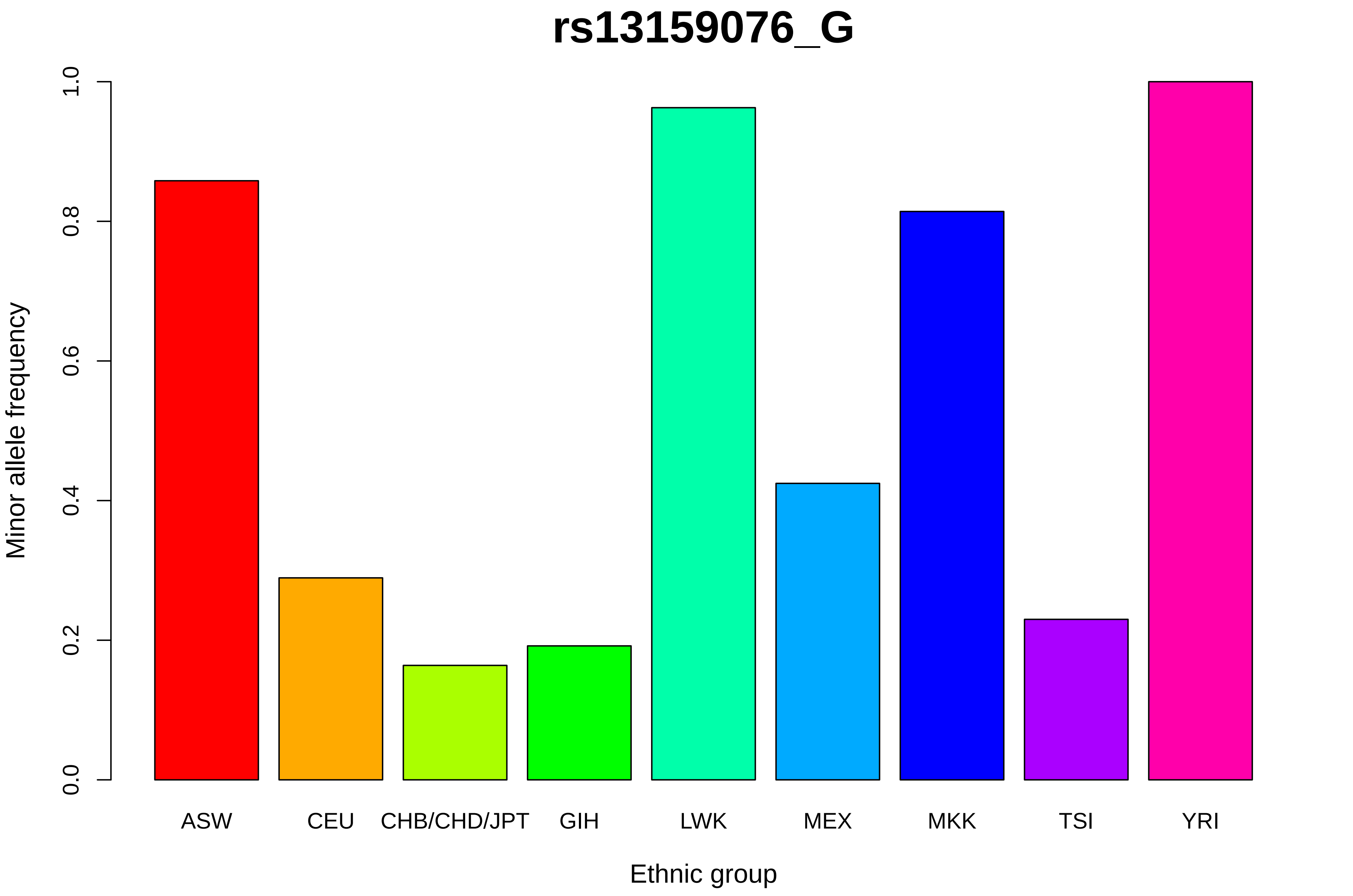
rs13159076_G
Minor allele frequency
0.0
0.2
0.4
0.6
0.8
1.0
ASW
CEU
CHB/CHD/JPT
GIH
LWK
MEX
MKK
TSI
YRI
Ethnic group

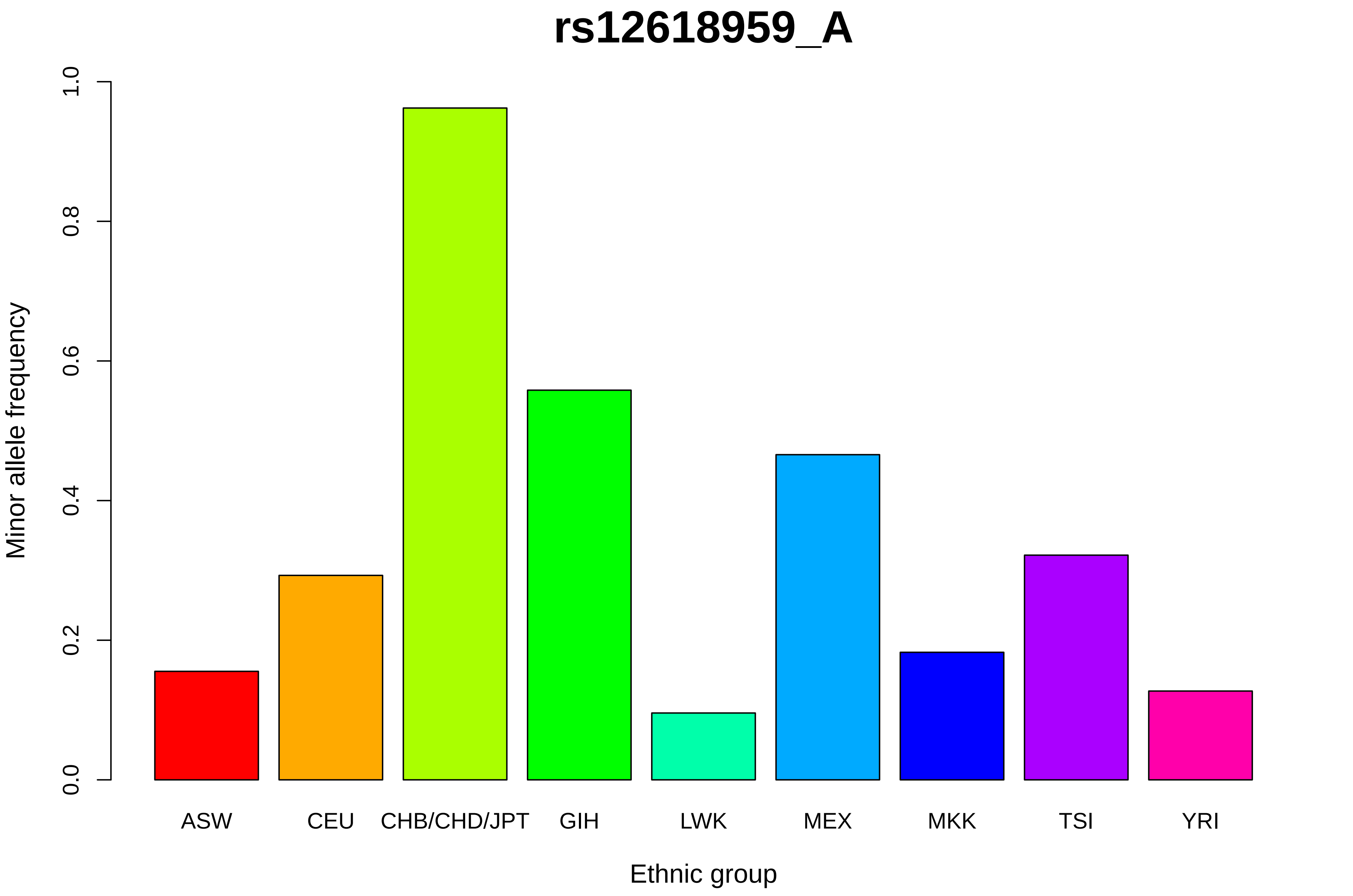
rs12618959_A
Minor allele frequency
0.0
0.2
0.4
0.6
0.8
1.0
ASW
CEU
CHB/CHD/JPT
GIH
LWK
MEX
MKK
TSI
YRI
Ethnic group

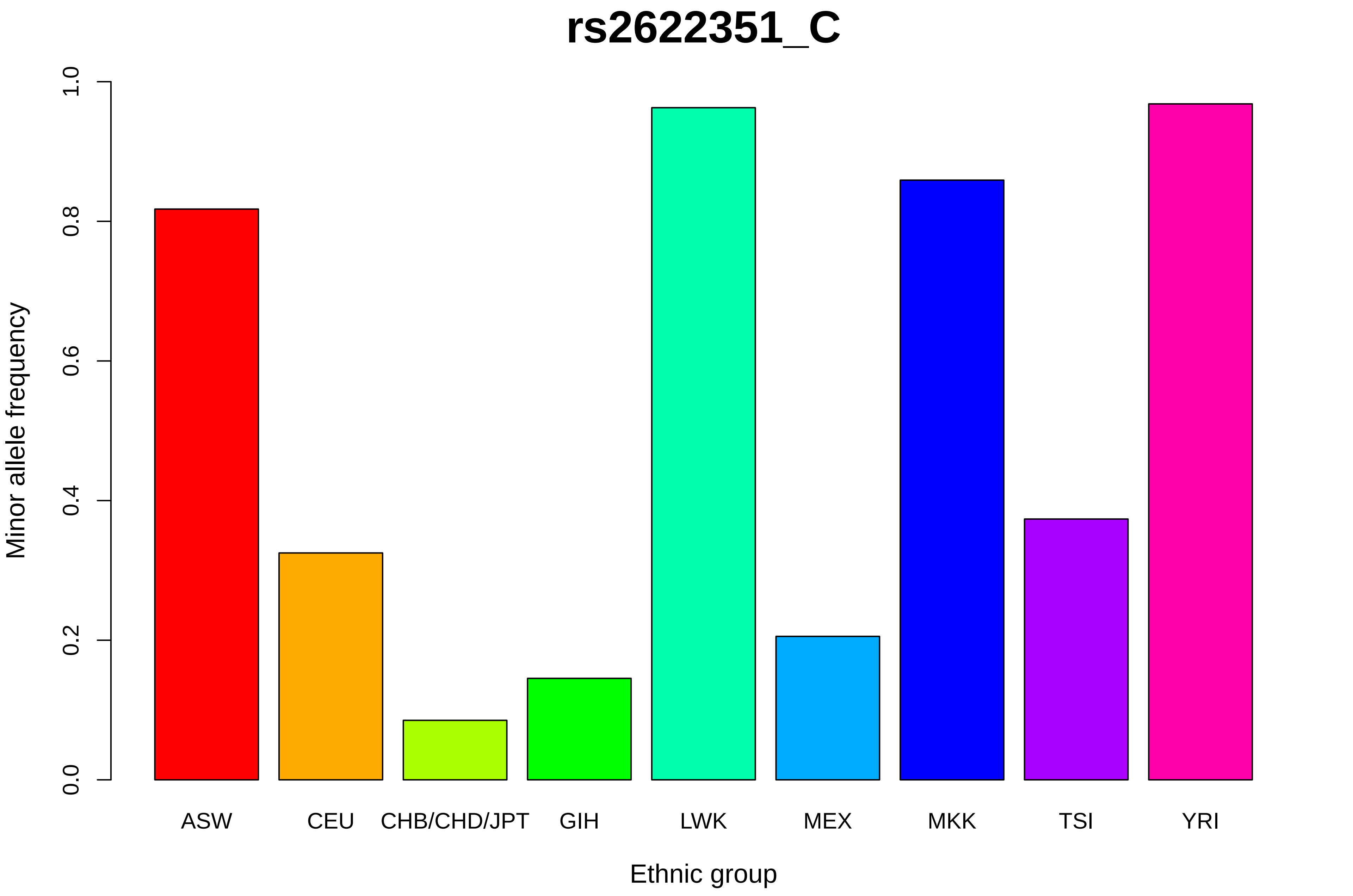
rs2622351_C
Minor allele frequency
0.0
0.2
0.4
0.6
0.8
1.0
ASW
CEU
CHB/CHD/JPT
GIH
LWK
MEX
MKK
TSI
YRI
Ethnic group

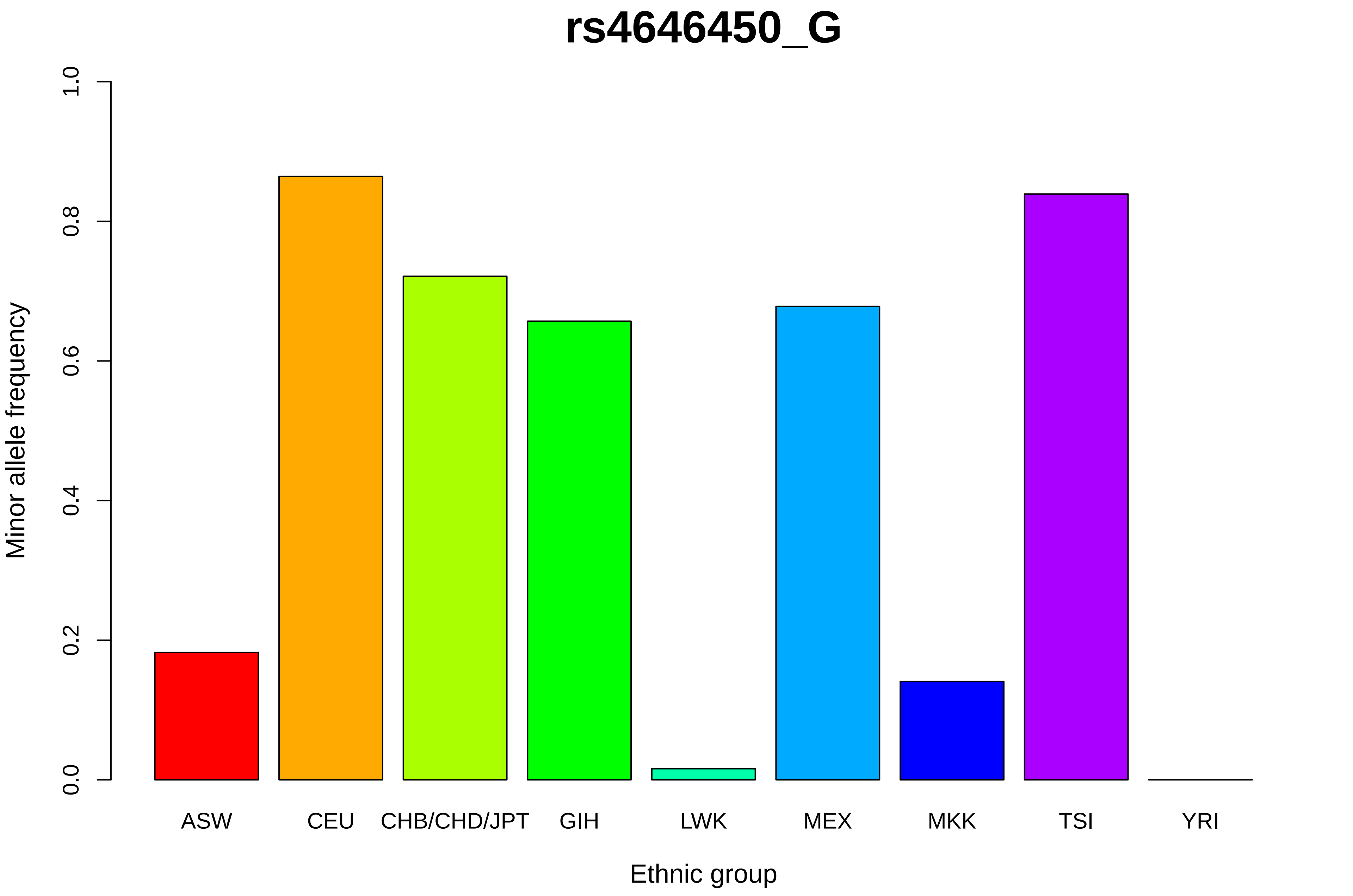
rs4646450_G
Minor allele frequency
0.0
0.2
0.4
0.6
0.8
1.0
ASW
CEU
CHB/CHD/JPT
GIH
LWK
MEX
MKK
TSI
YRI
Ethnic group

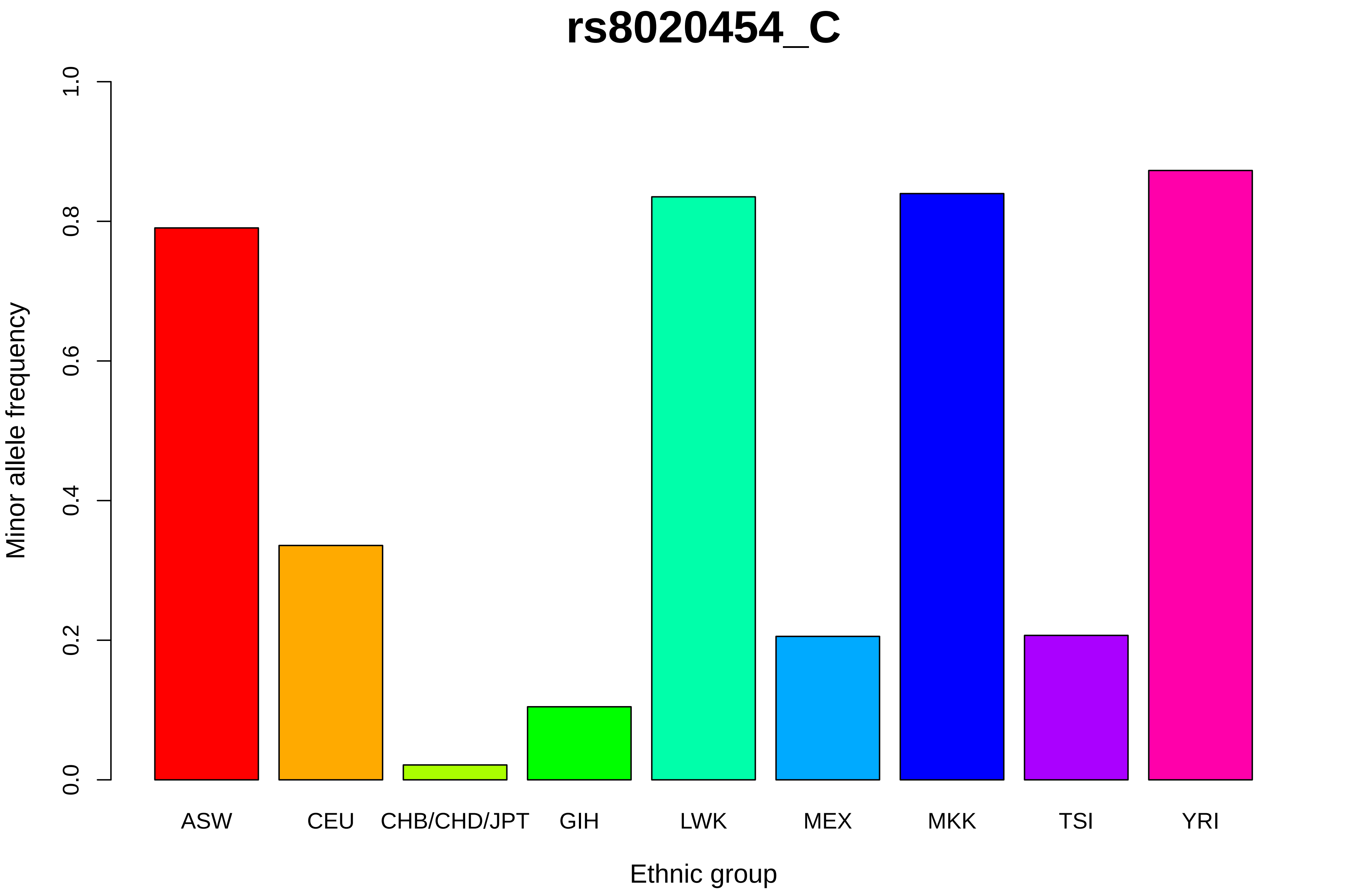

rs8020454_C
Minor allele frequency
0.0
0.2
0.4
0.6
0.8
1.0
ASW
CEU
CHB/CHD/JPT
GIH
LWK
MEX
MKK
TSI
YRI
Ethnic group

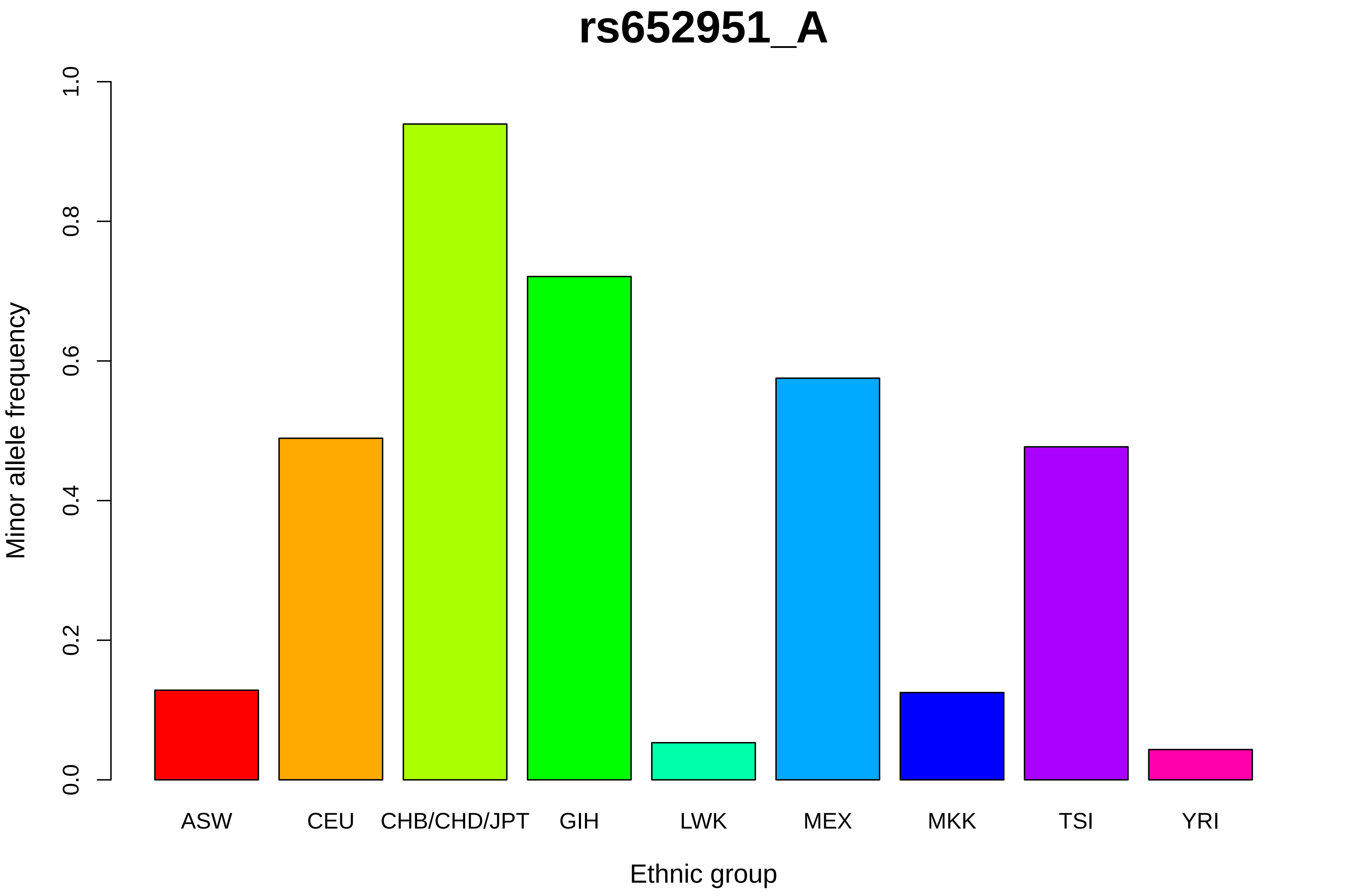
rs652951_A
Minor allele frequency
0.0
0.2
0.4
0.6
0.8
1.0
ASW
CEU
CHB/CHD/JPT
GIH
LWK
MEX
MKK
TSI
YRI
Ethnic group

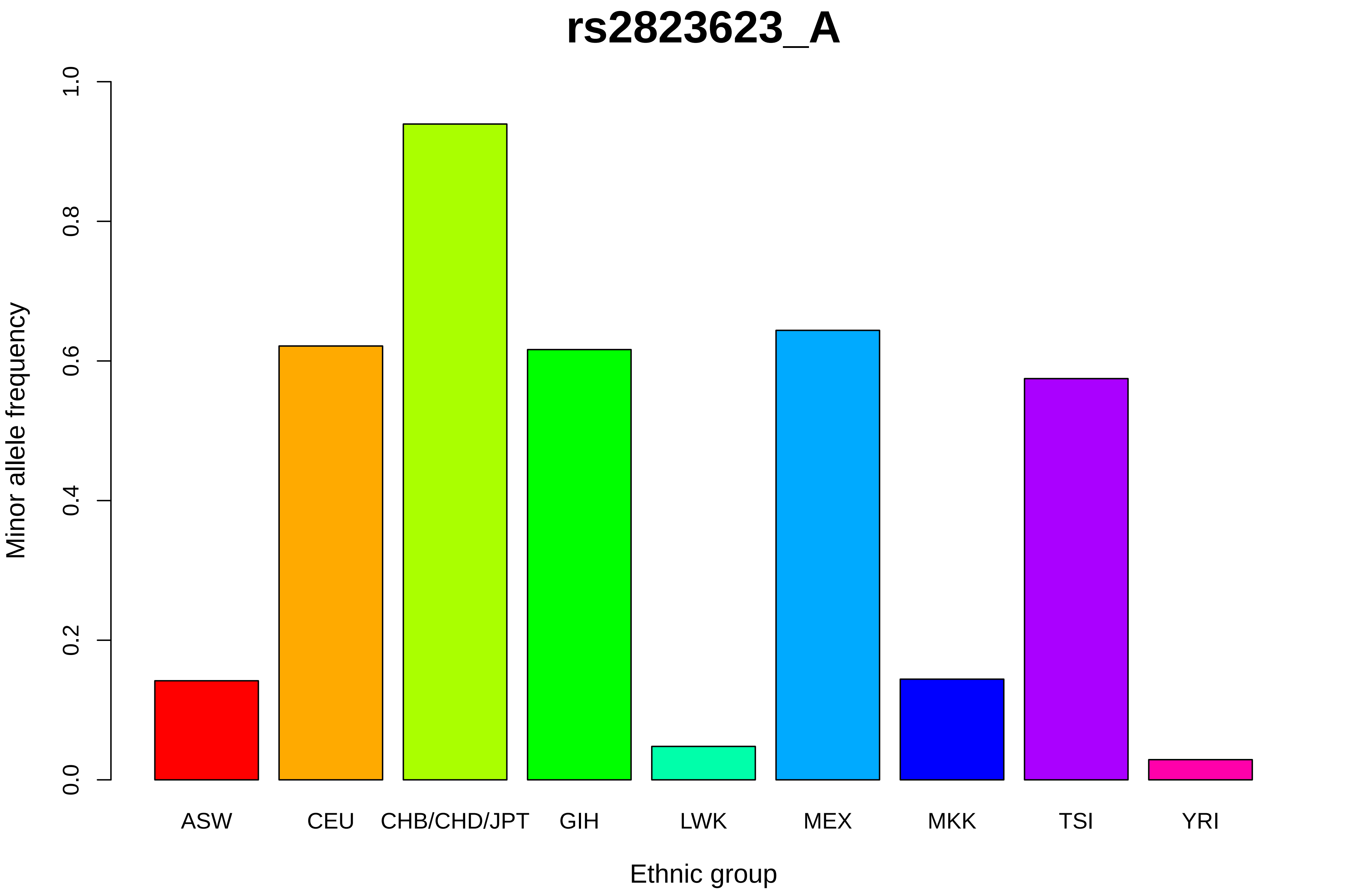
rs2823623_A
Minor allele frequency
0.0
0.2
0.4
0.6
0.8
1.0
ASW
CEU
CHB/CHD/JPT
GIH
LWK
MEX
MKK
TSI
YRI
Ethnic group

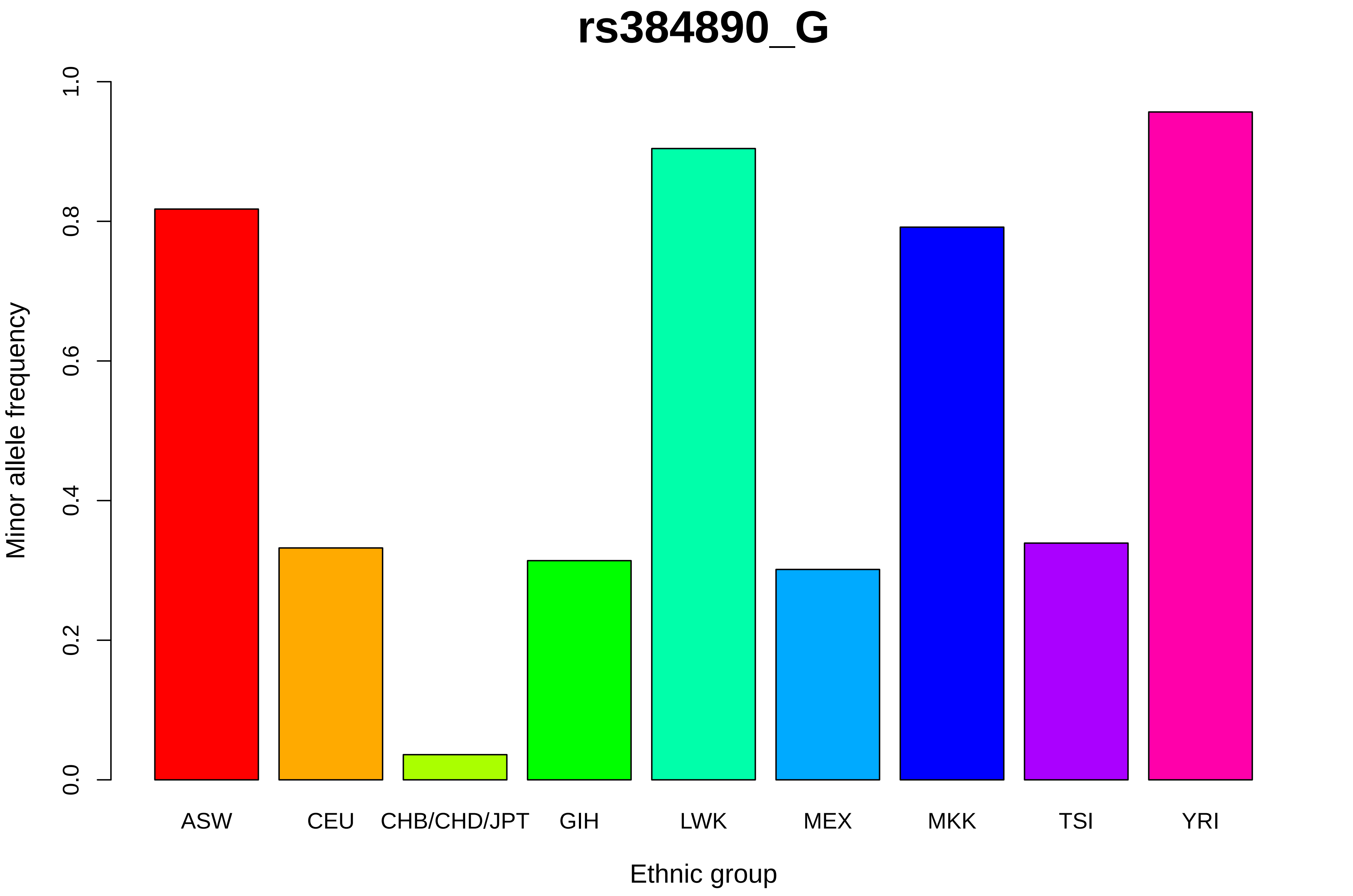

rs384890_G
Minor allele frequency
0.0
0.2
0.4
0.6
0.8
1.0
ASW
CEU
CHB/CHD/JPT
GIH
LWK
MEX
MKK
TSI
YRI
Ethnic group

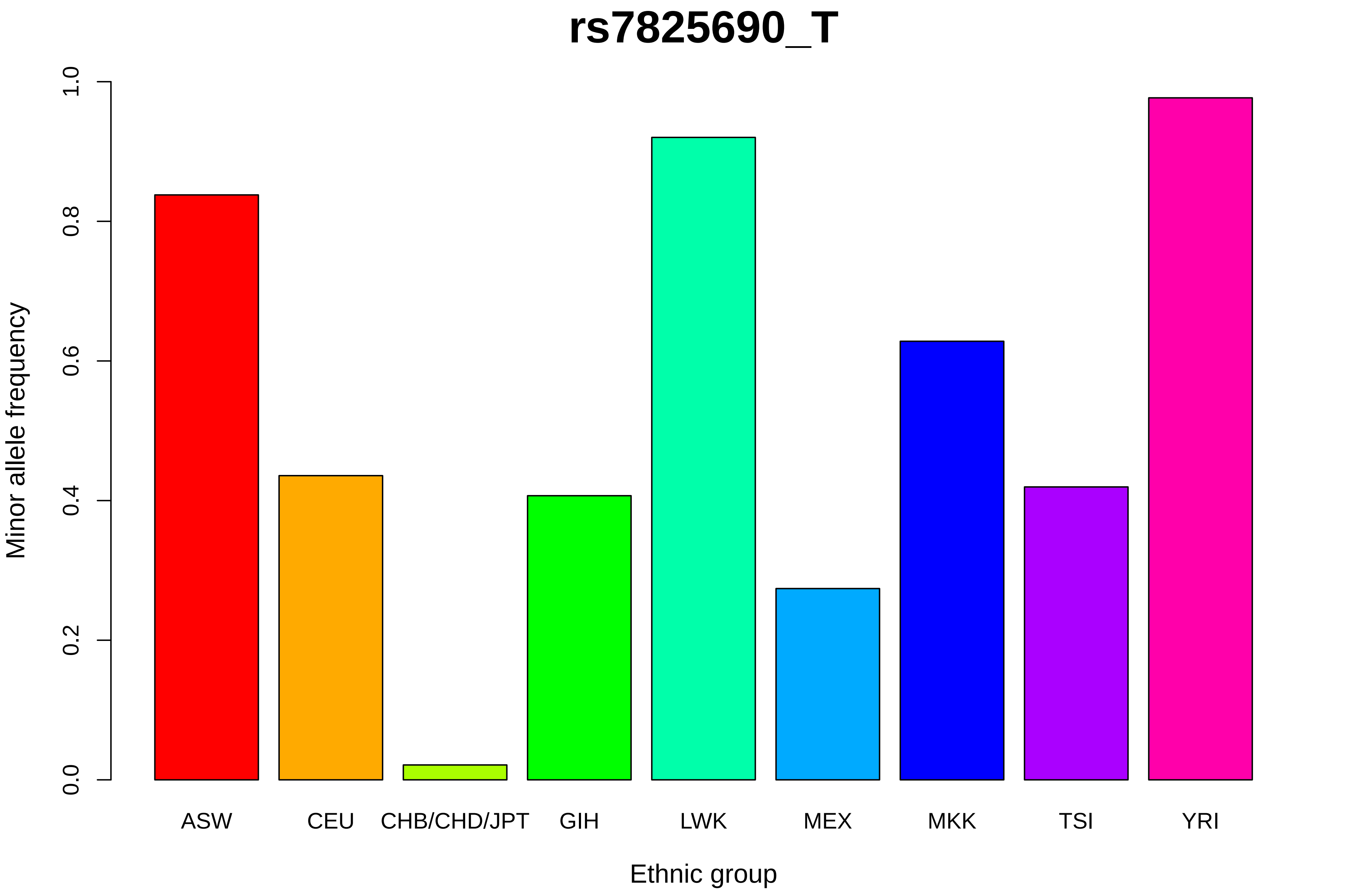
rs7825690_T
Minor allele frequency
0.0
0.2
0.4
0.6
0.8
1.0
ASW
CEU
CHB/CHD/JPT
GIH
LWK
MEX
MKK
TSI
YRI
Ethnic group

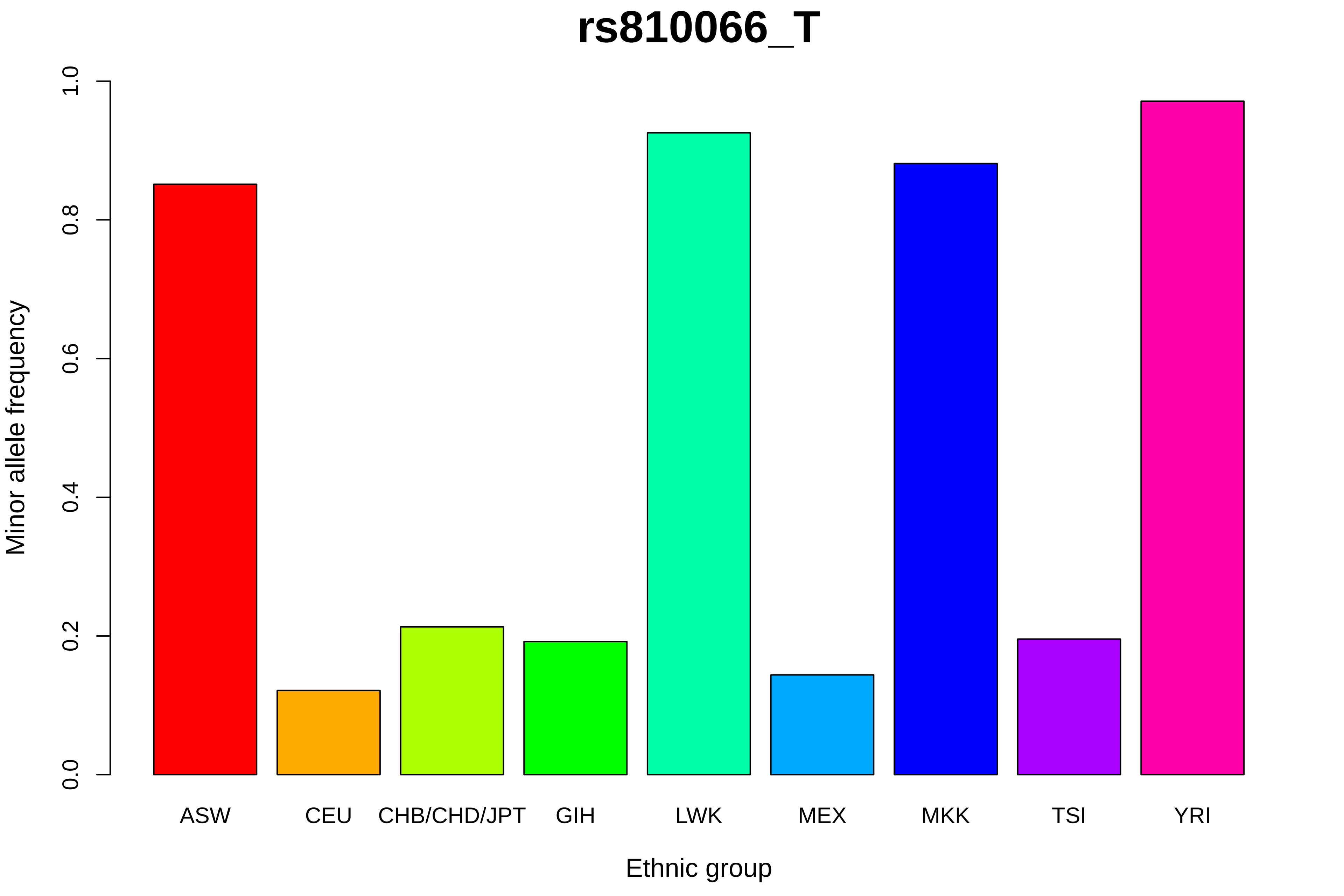
rs810066_T
Minor allele frequency
0.0
0.2
0.4
0.6
0.8
1.0
ASW
CEU
CHB/CHD/JPT
GIH
LWK
MEX
MKK
TSI
YRI
Ethnic group

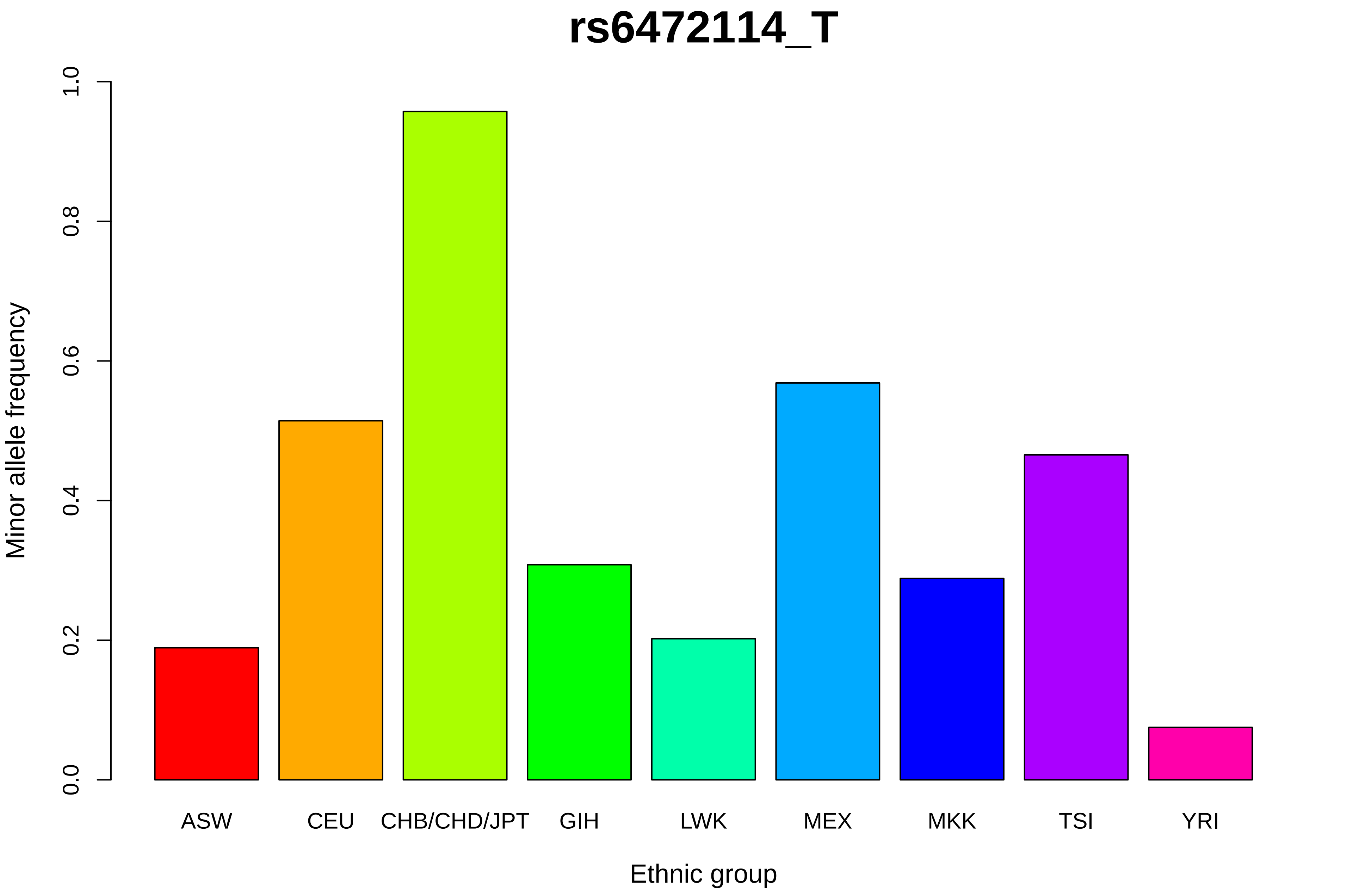
rs6472114_T
Minor allele frequency
0.0
0.2
0.4
0.6
0.8
1.0
ASW
CEU
CHB/CHD/JPT
GIH
LWK
MEX
MKK
TSI
YRI
Ethnic group

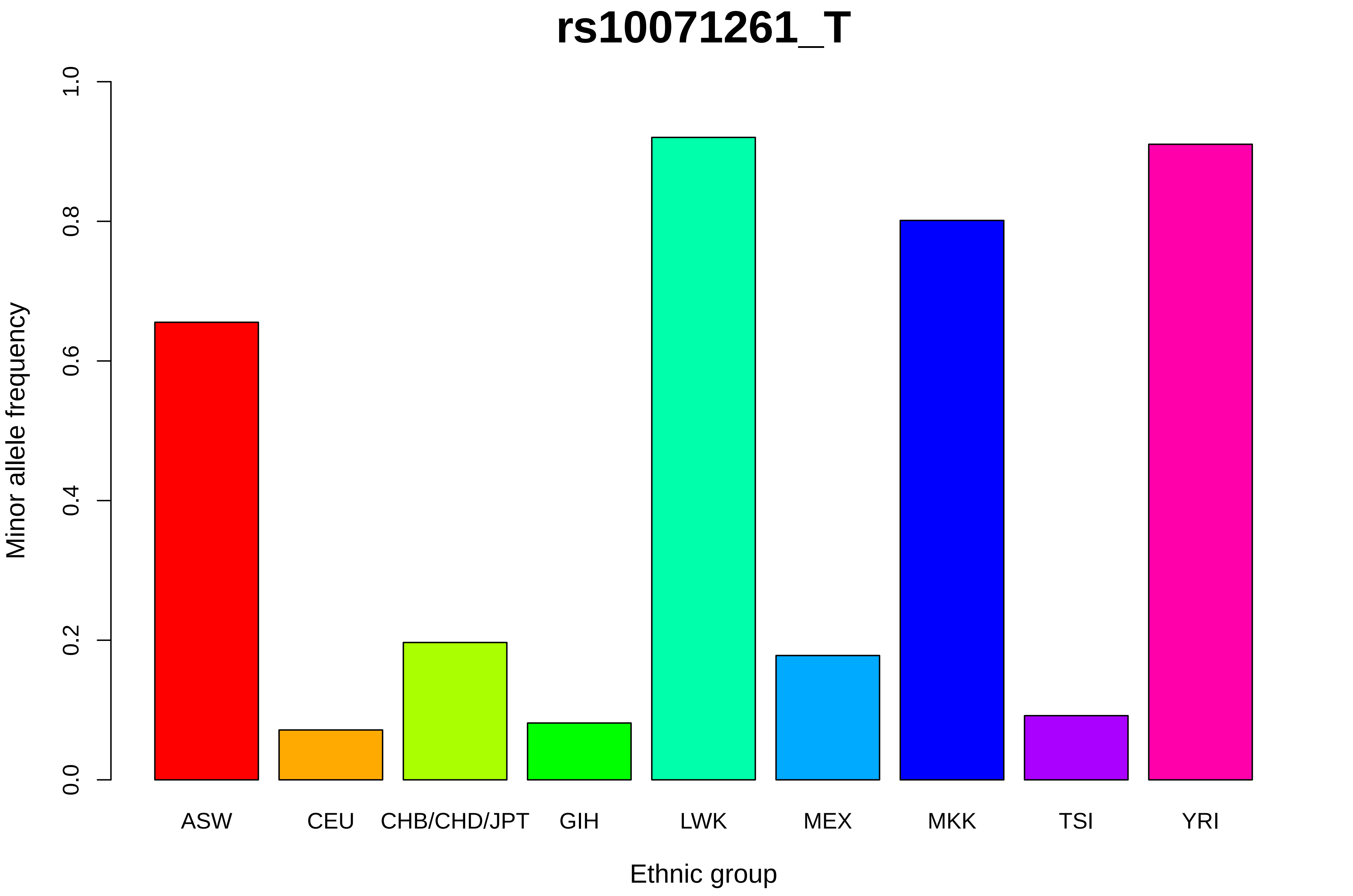
rs10071261_T
Minor allele frequency
0.0
0.2
0.4
0.6
0.8
1.0
ASW
CEU
CHB/CHD/JPT
GIH
LWK
MEX
MKK
TSI
YRI
Ethnic group

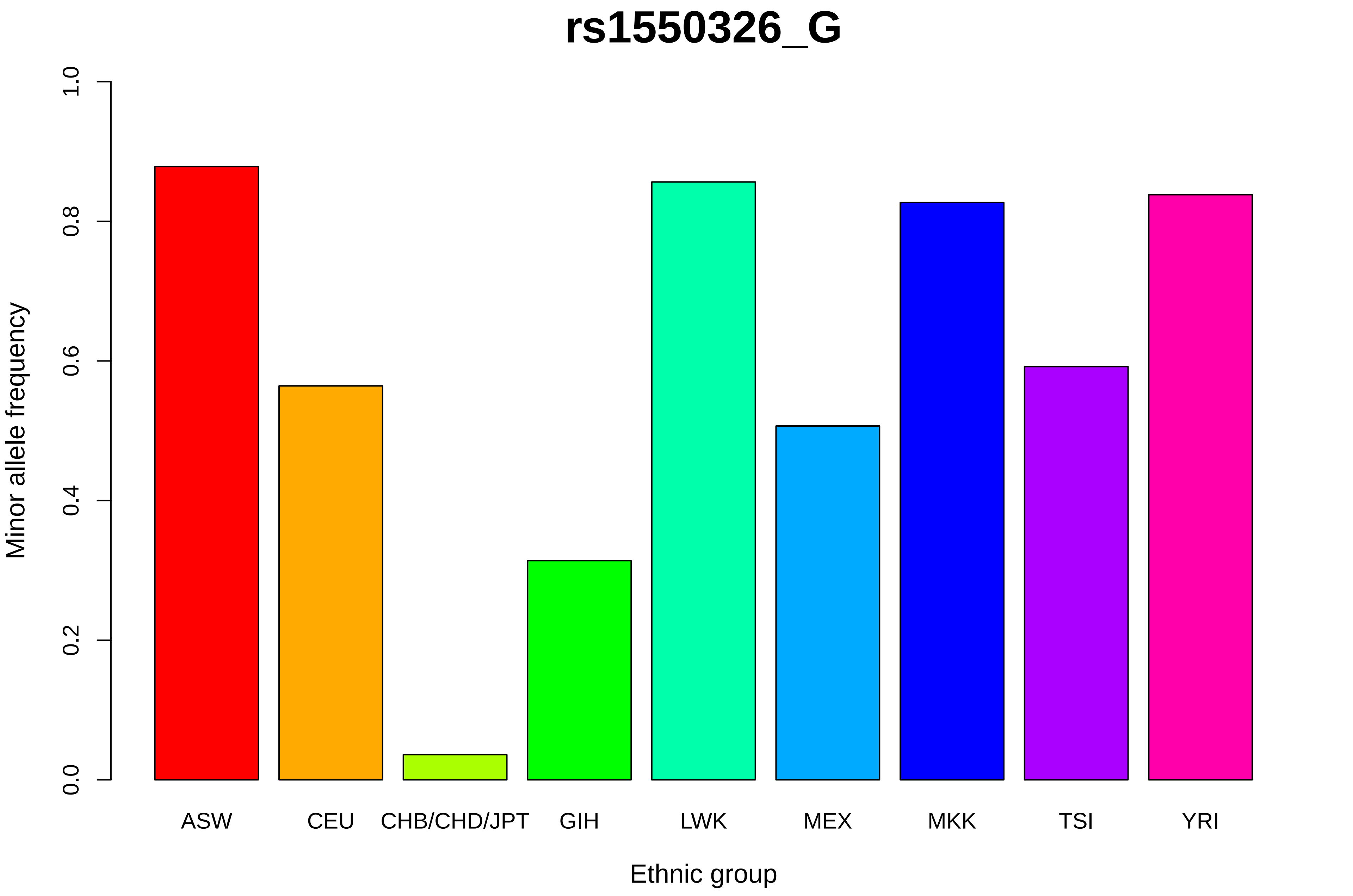
rs1550326_G
Minor allele frequency
0.0
0.2
0.4
0.6
0.8
1.0
ASW
CEU
CHB/CHD/JPT
GIH
LWK
MEX
MKK
TSI
YRI
Ethnic group

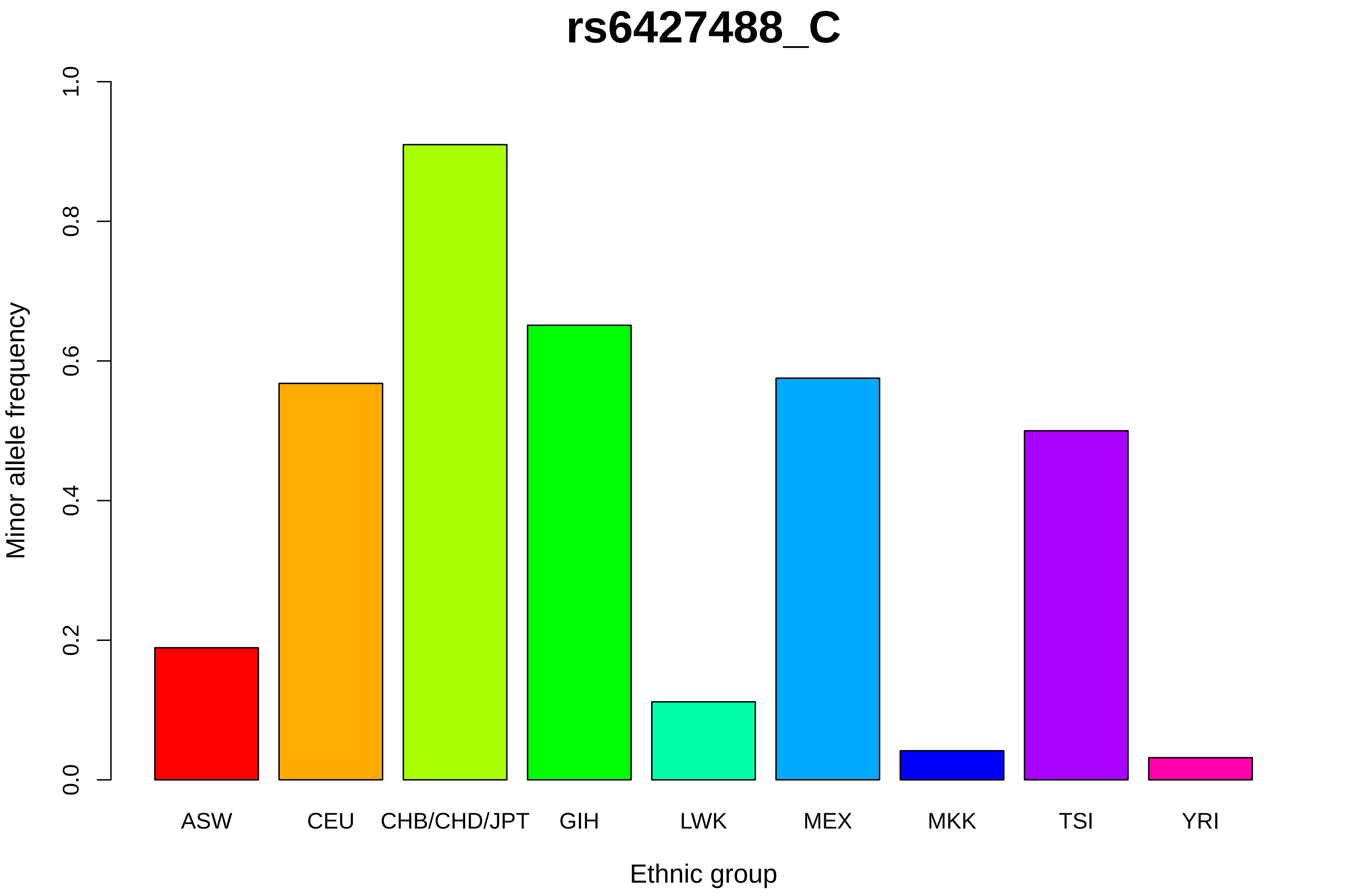

rs6427488_C
Minor allele frequency
0.0
0.2
0.4
0.6
0.8
1.0
ASW
CEU
CHB/CHD/JPT
GIH
LWK
MEX
MKK
TSI
YRI
Ethnic group

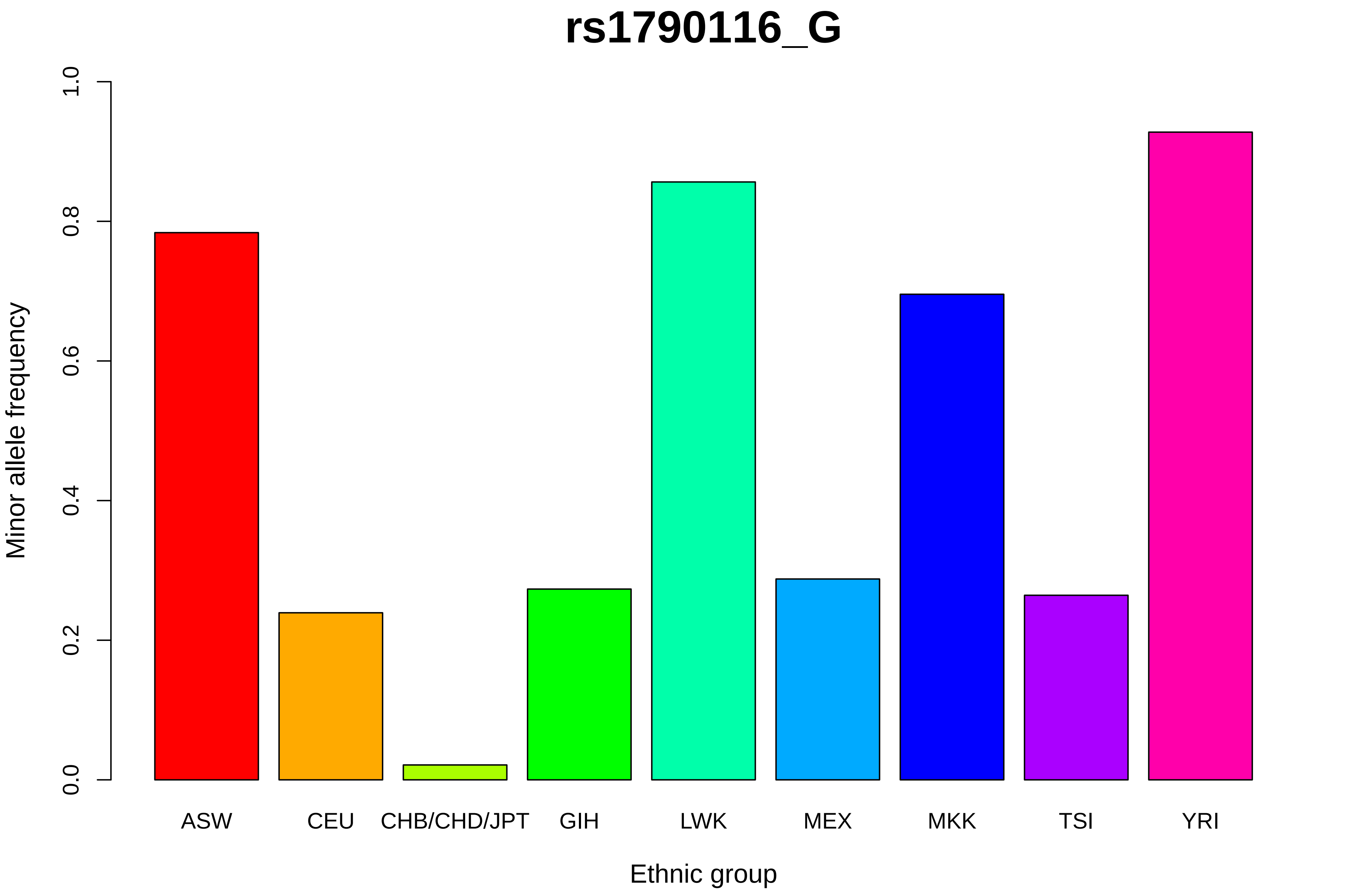
rs1790116_G
Minor allele frequency
0.0
0.2
0.4
0.6
0.8
1.0
ASW
CEU
CHB/CHD/JPT
GIH
LWK
MEX
MKK
TSI
YRI
Ethnic group

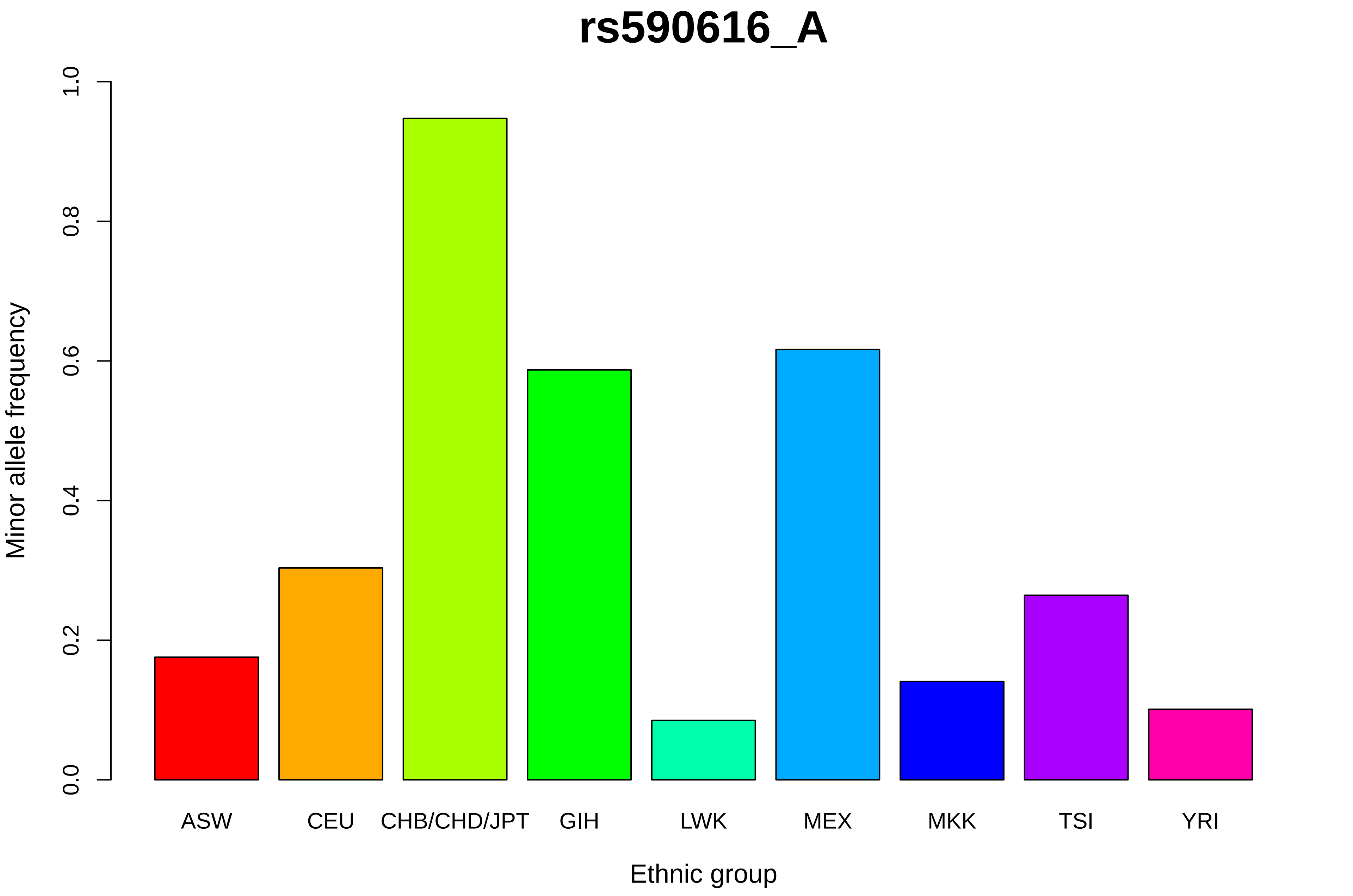
rs590616_A
Minor allele frequency
0.0
0.2
0.4
0.6
0.8
1.0
ASW
CEU
CHB/CHD/JPT
GIH
LWK
MEX
MKK
TSI
YRI
Ethnic group

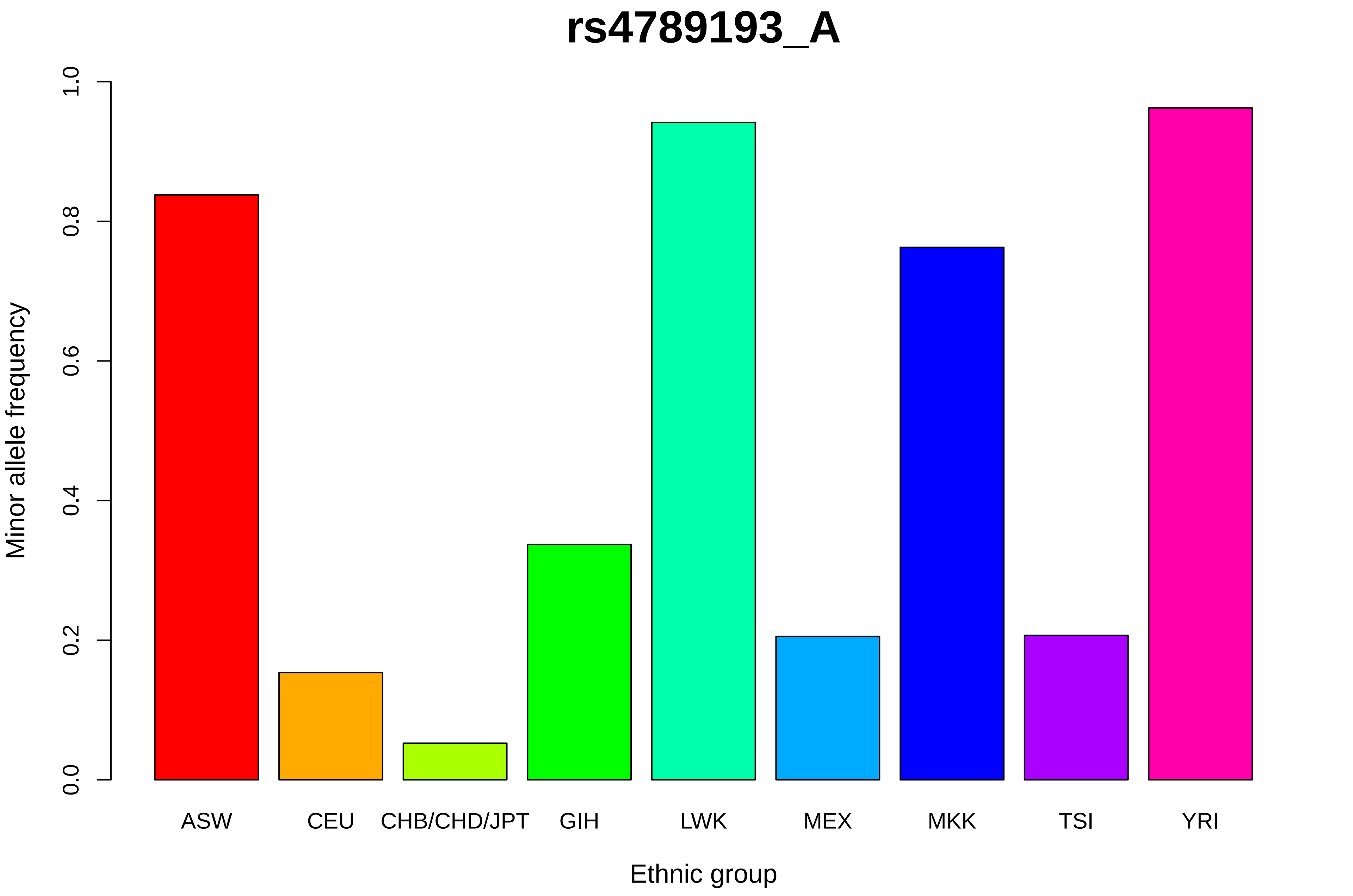
rs4789193_A
Minor allele frequency
0.0
0.2
0.4
0.6
0.8
1.0
ASW
CEU
CHB/CHD/JPT
GIH
LWK
MEX
MKK
TSI
YRI
Ethnic group

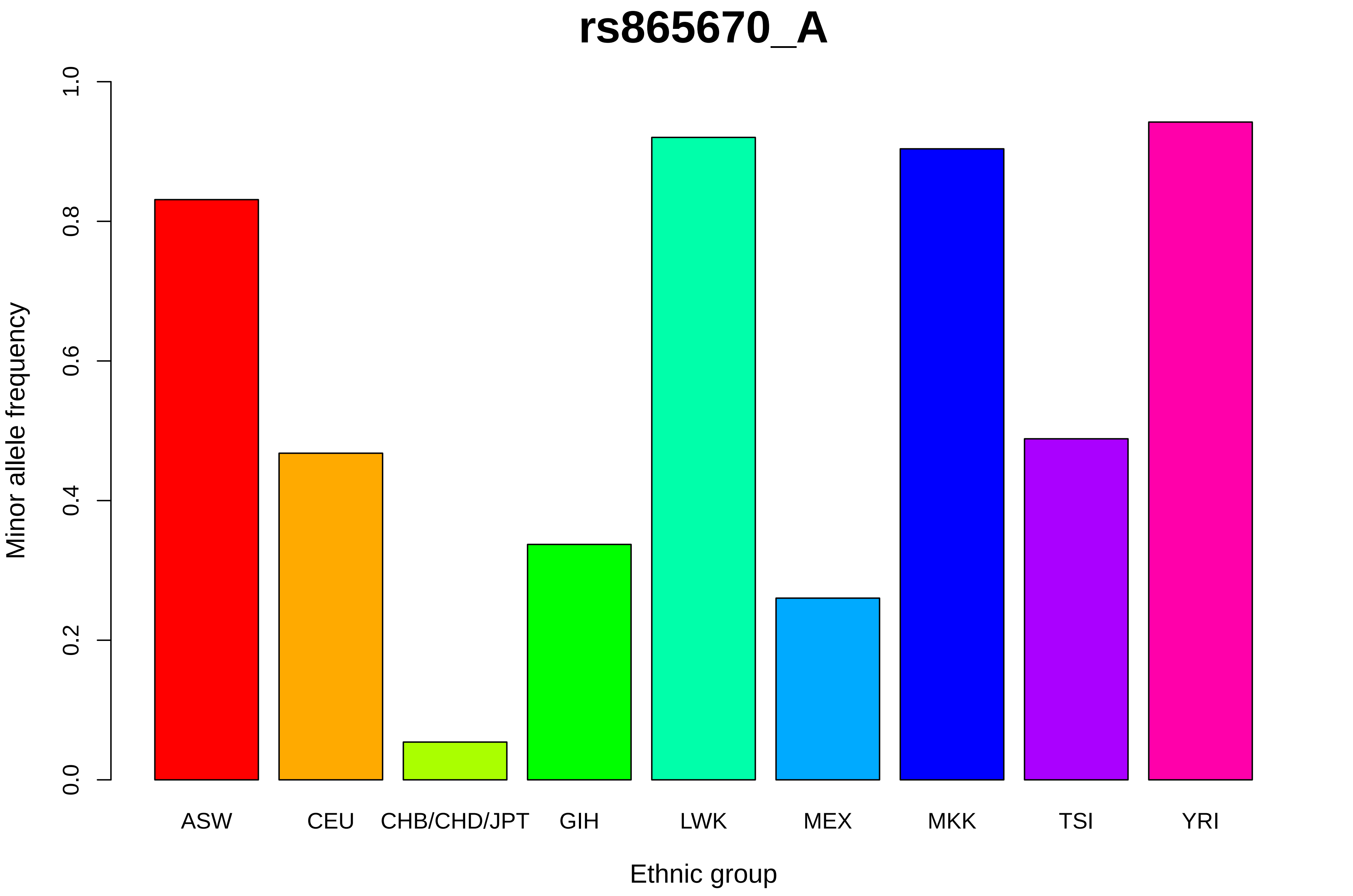

rs865670_A
Minor allele frequency
0.0
0.2
0.4
0.6
0.8
1.0
ASW
CEU
CHB/CHD/JPT
GIH
LWK
MEX
MKK
TSI
YRI
Ethnic group

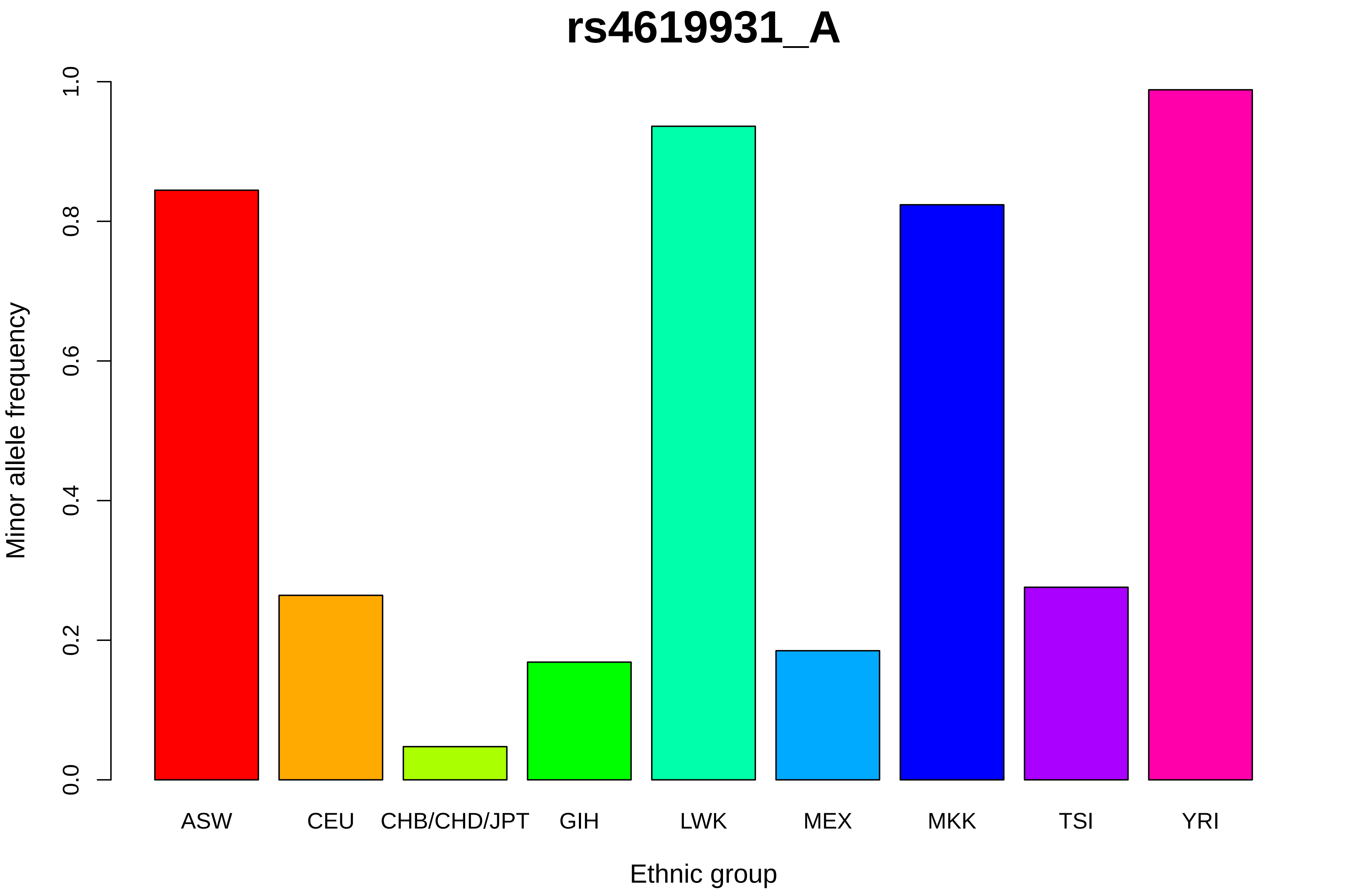
rs4619931_A
Minor allele frequency
0.0
0.2
0.4
0.6
0.8
1.0
ASW
CEU
CHB/CHD/JPT
GIH
LWK
MEX
MKK
TSI
YRI
Ethnic group

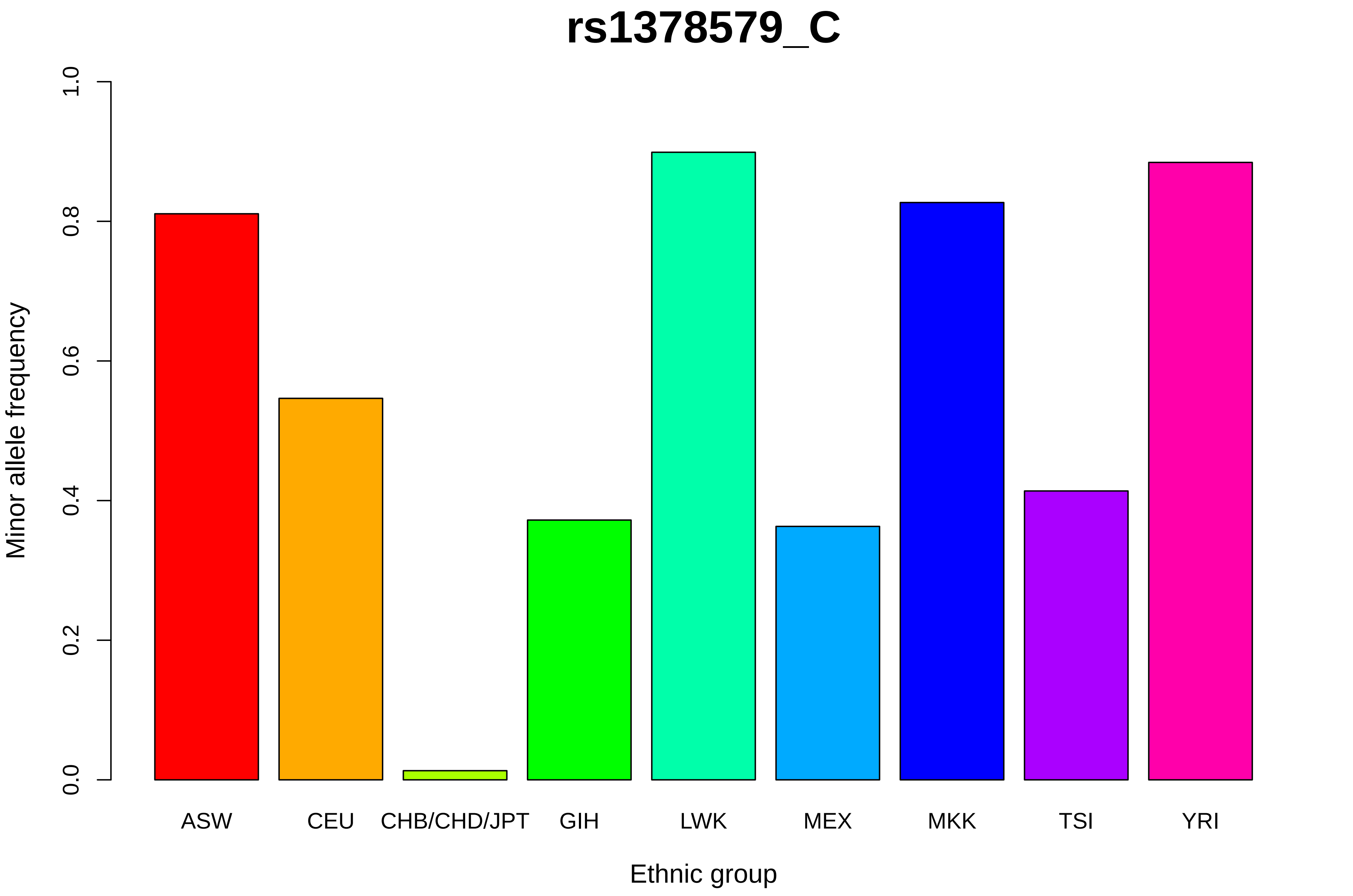
rs1378579_C
Minor allele frequency
0.0
0.2
0.4
0.6
0.8
1.0
ASW
CEU
CHB/CHD/JPT
GIH
LWK
MEX
MKK
TSI
YRI
Ethnic group

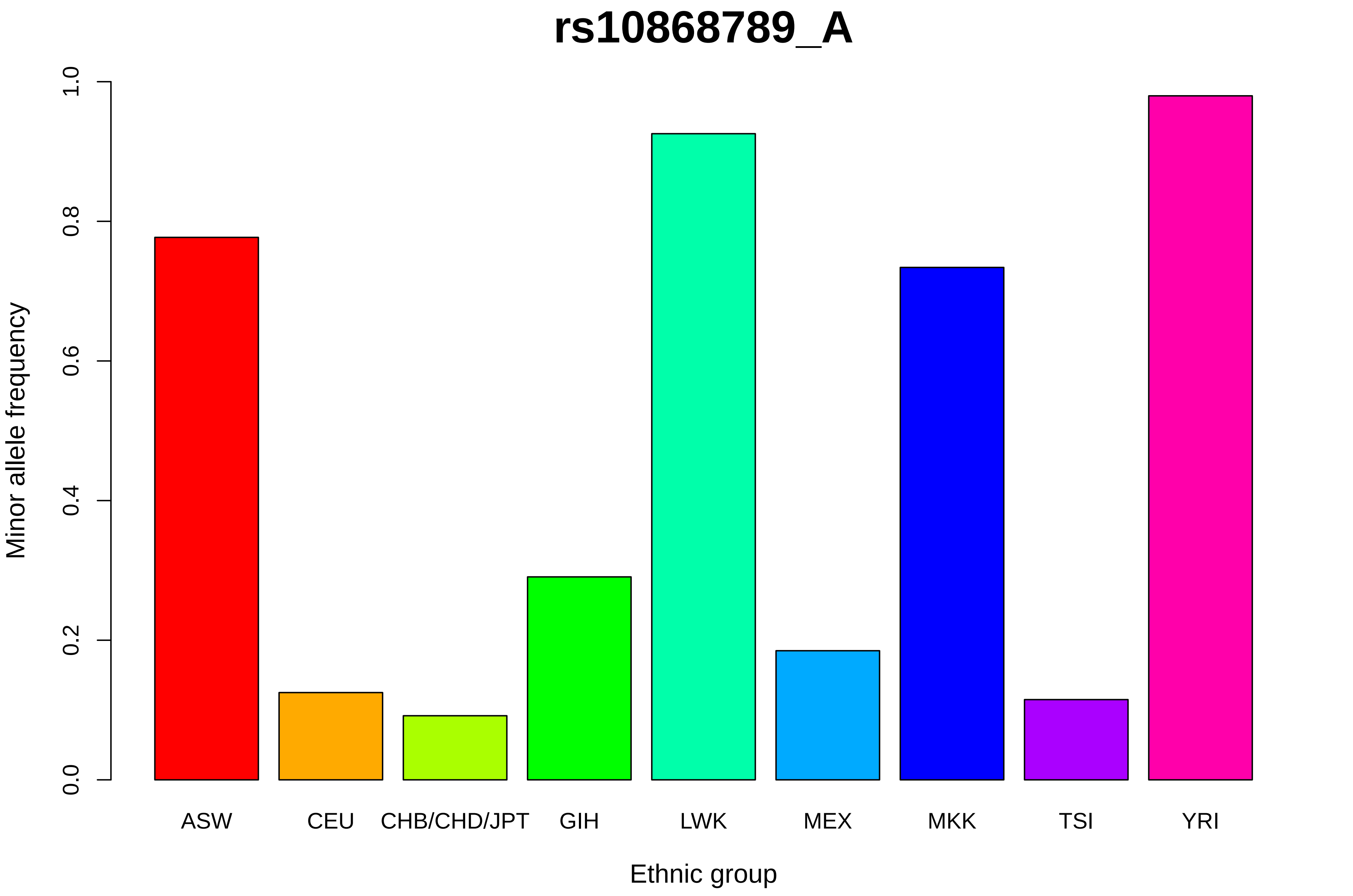
rs10868789_A
Minor allele frequency
0.0
0.2
0.4
0.6
0.8
1.0
ASW
CEU
CHB/CHD/JPT
GIH
LWK
MEX
MKK
TSI
YRI
Ethnic group

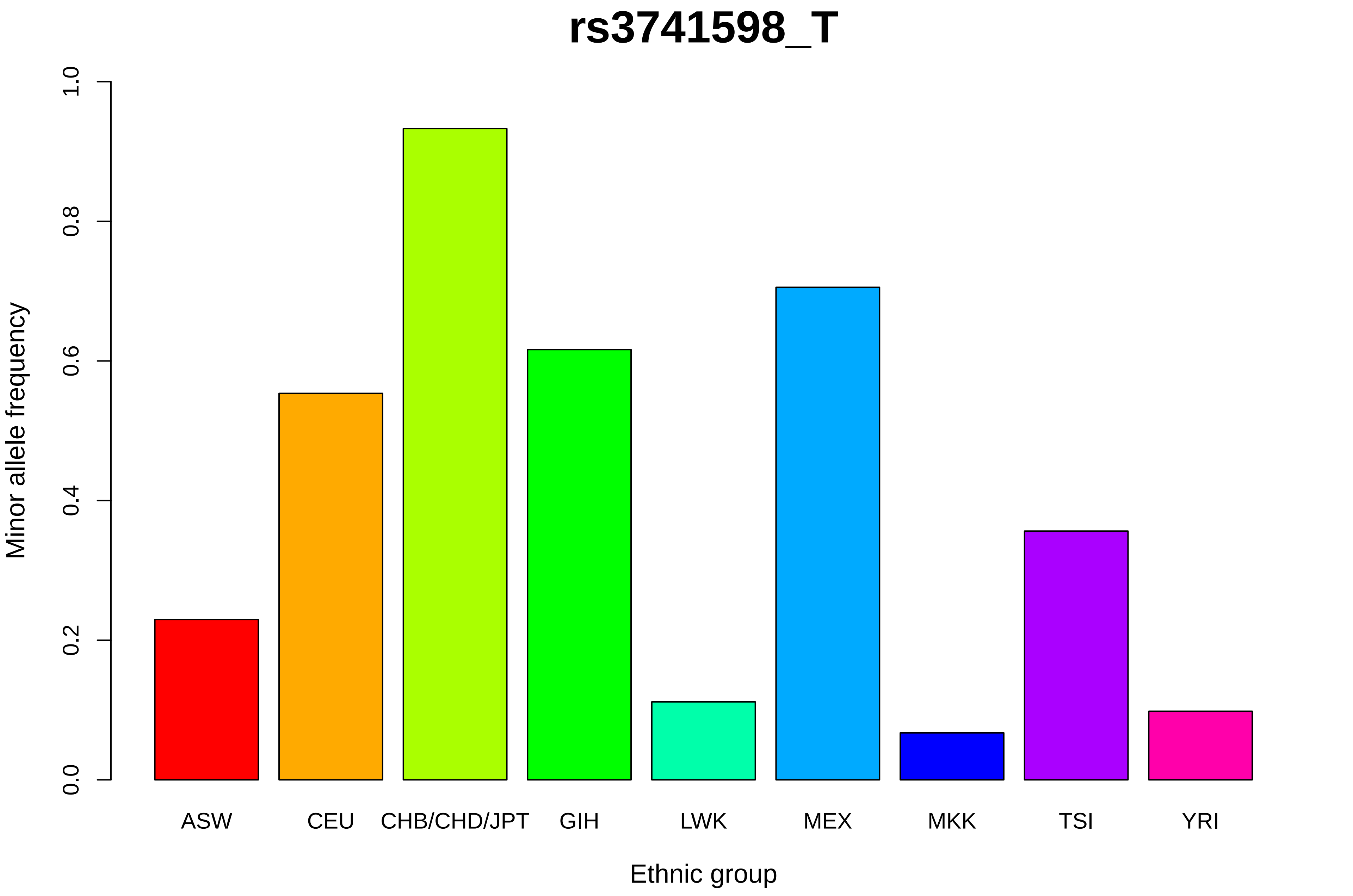
rs3741598_T
Minor allele frequency
0.0
0.2
0.4
0.6
0.8
1.0
ASW
CEU
CHB/CHD/JPT
GIH
LWK
MEX
MKK
TSI
YRI
Ethnic group

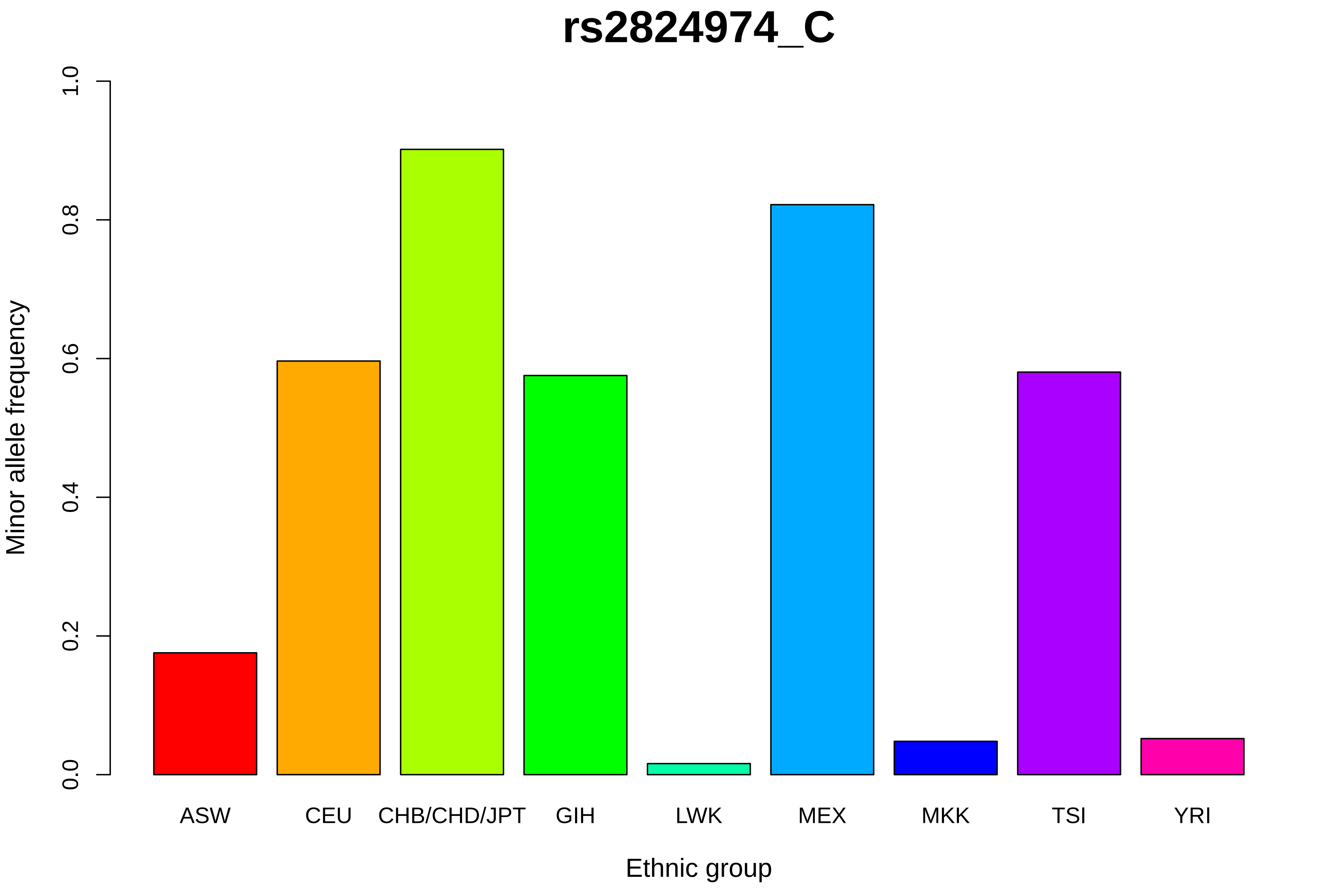
rs2824974_C
Minor allele frequency
0.0
0.2
0.4
0.6
0.8
1.0
ASW
CEU
CHB/CHD/JPT
GIH
LWK
MEX
MKK
TSI
YRI
Ethnic group

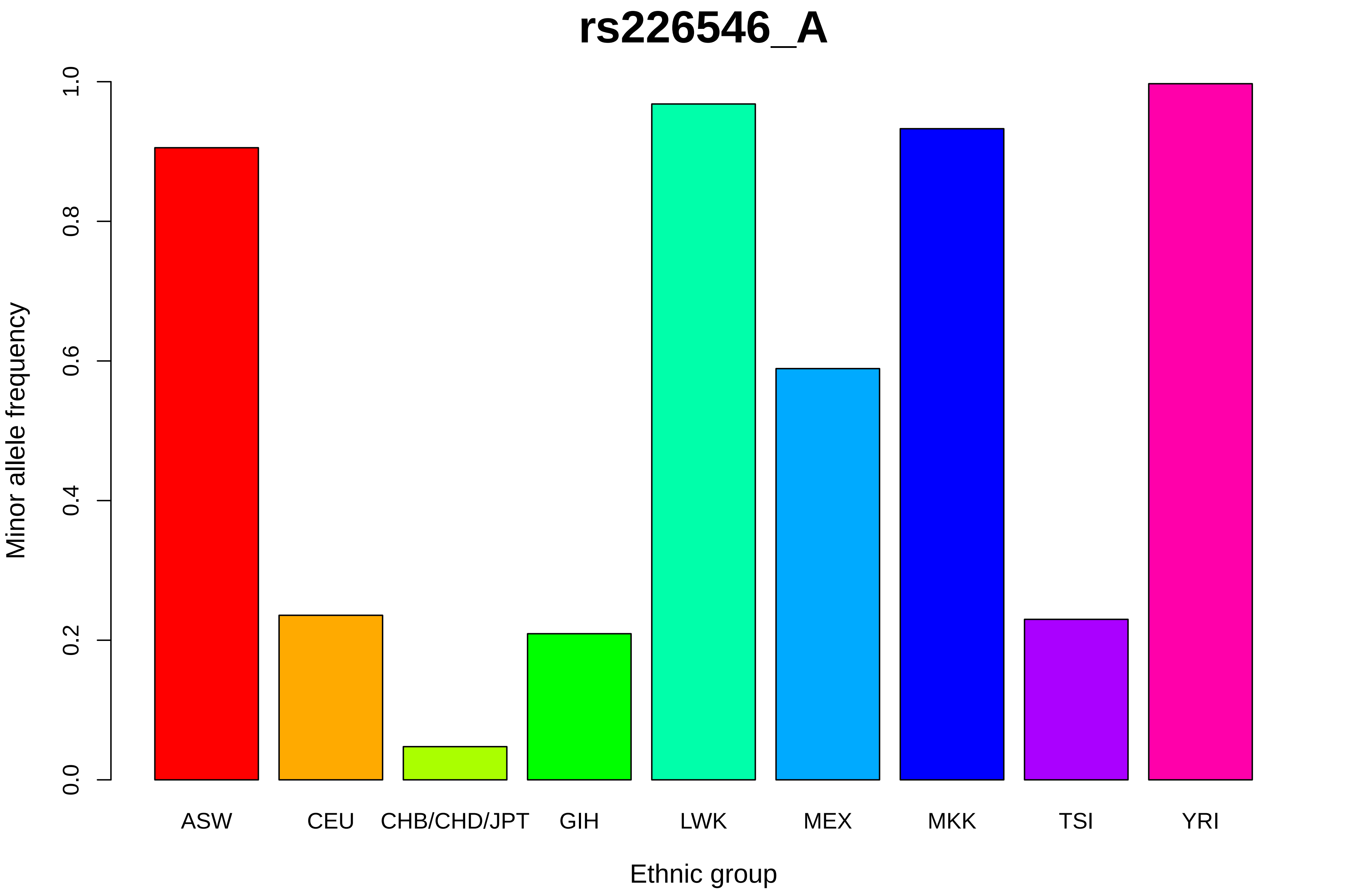
rs226546_A
Minor allele frequency
0.0
0.2
0.4
0.6
0.8
1.0
ASW
CEU
CHB/CHD/JPT
GIH
LWK
MEX
MKK
TSI
YRI
Ethnic group

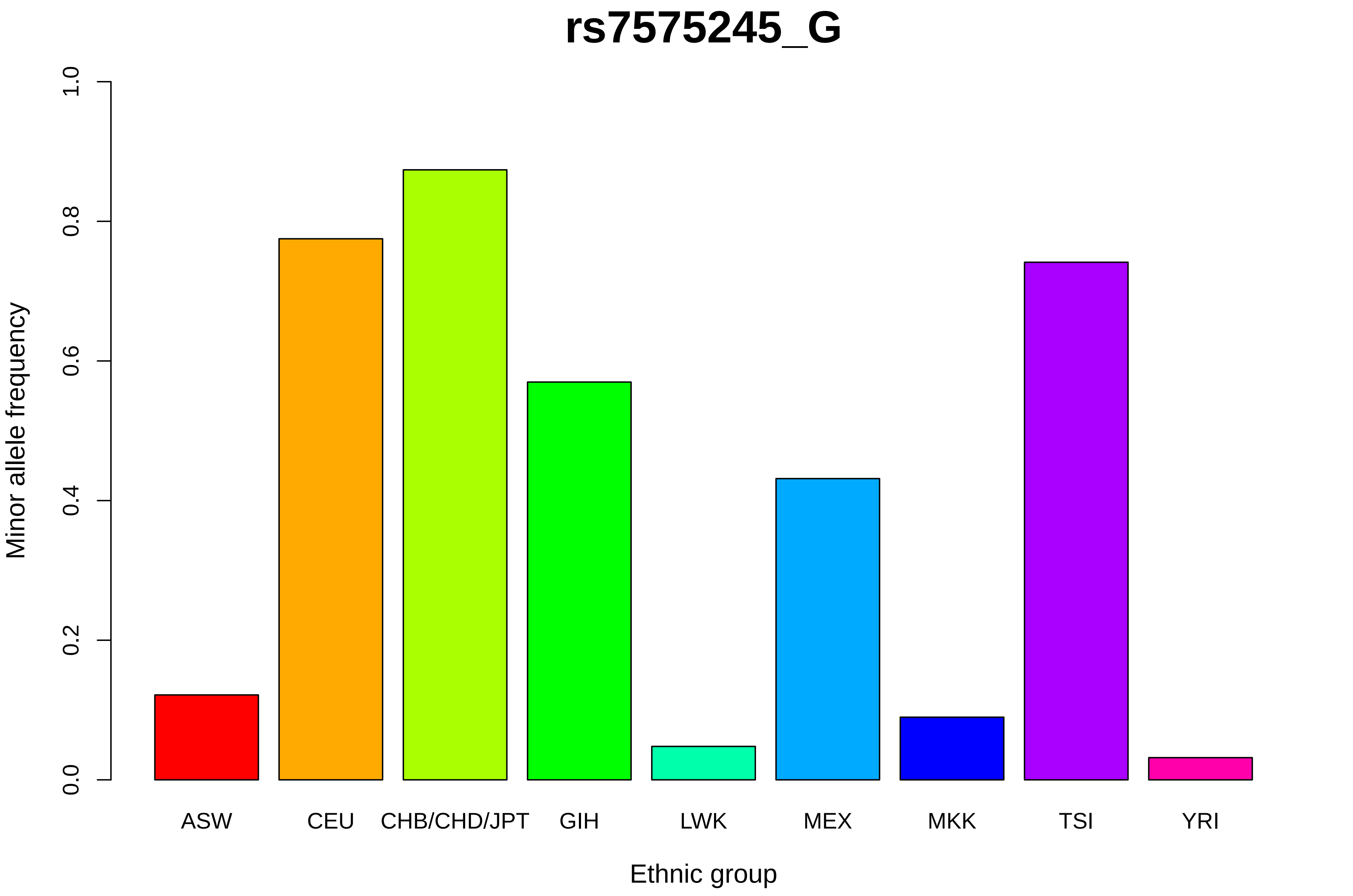

rs7575245_G
Minor allele frequency
0.0
0.2
0.4
0.6
0.8
1.0
ASW
CEU
CHB/CHD/JPT
GIH
LWK
MEX
MKK
TSI
YRI
Ethnic group

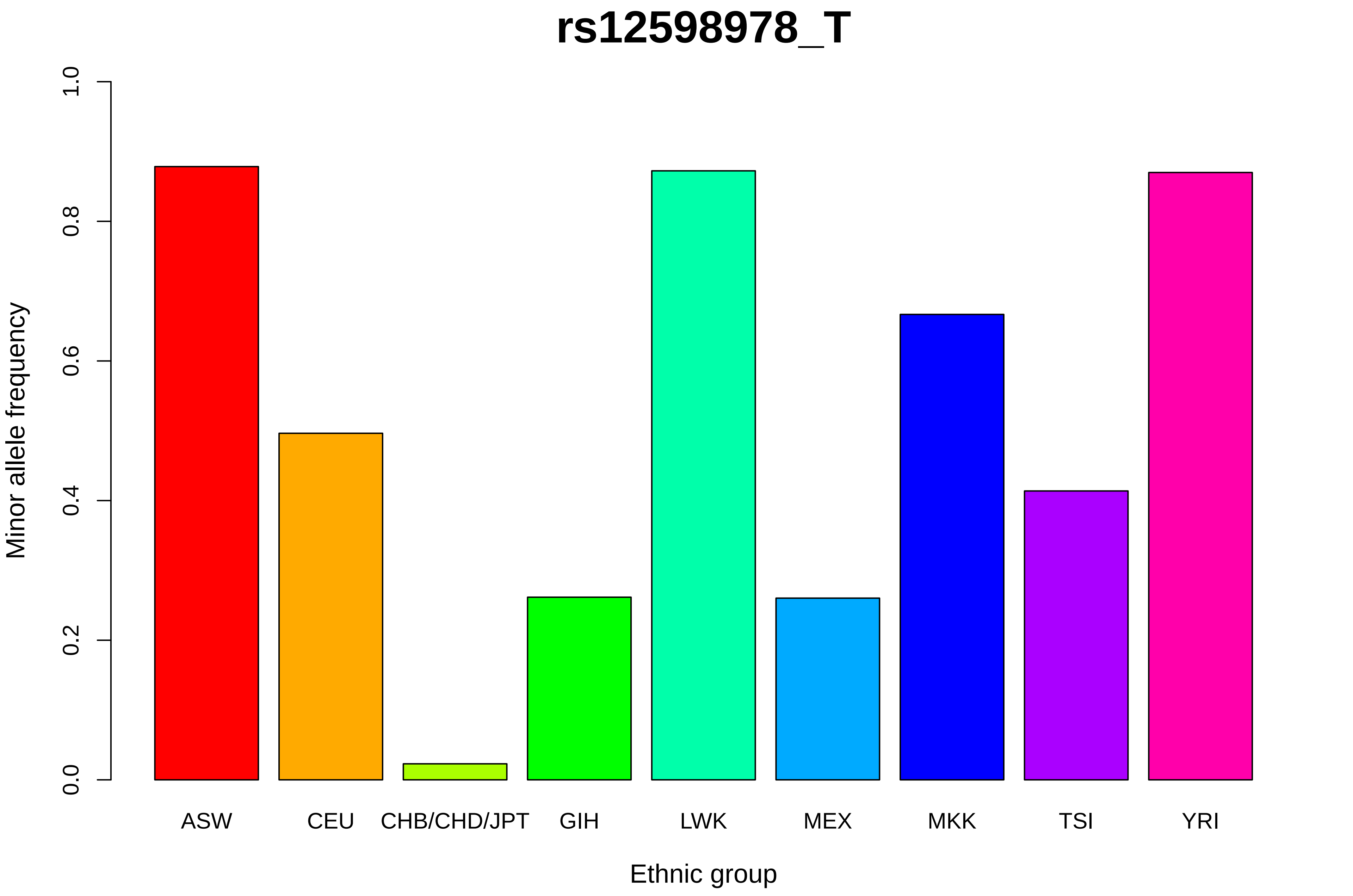

rs12598978_T
Minor allele frequency
0.0
0.2
0.4
0.6
0.8
1.0
ASW
CEU
CHB/CHD/JPT
GIH
LWK
MEX
MKK
TSI
YRI
Ethnic group

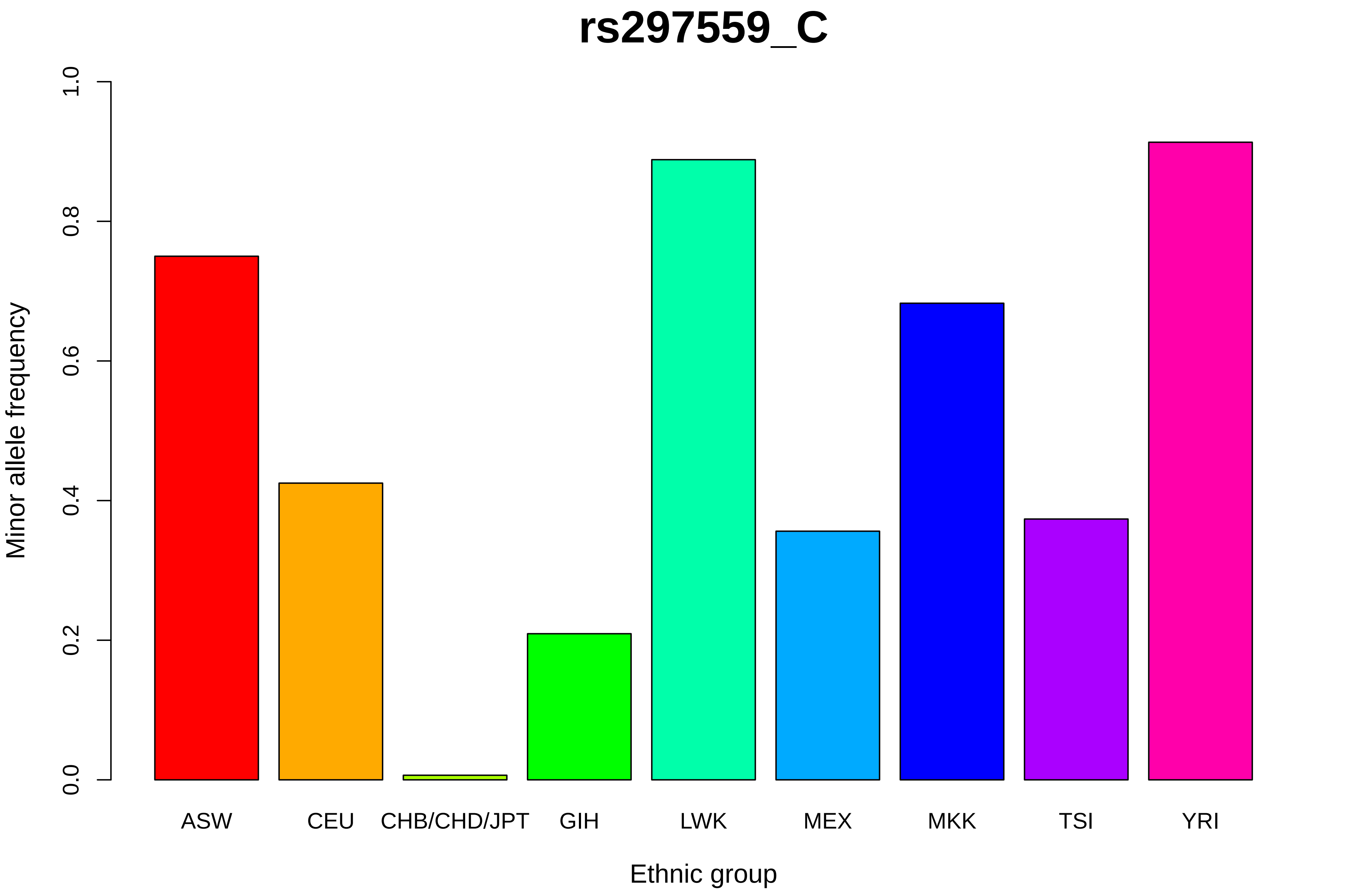

rs297559_C
Minor allele frequency
0.0
0.2
0.4
0.6
0.8
1.0
ASW
CEU
CHB/CHD/JPT
GIH
LWK
MEX
MKK
TSI
YRI
Ethnic group

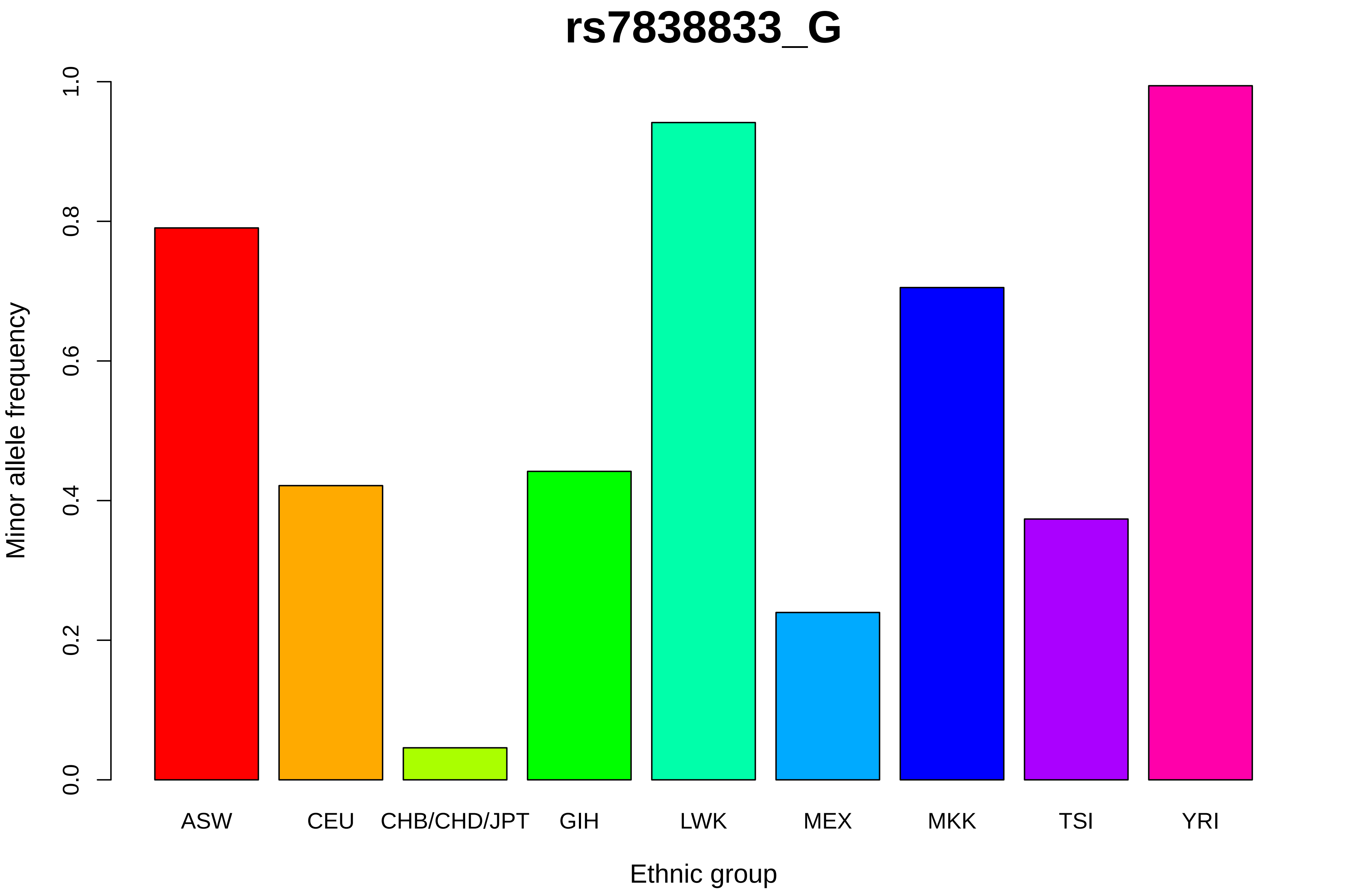

rs7838833_G
Minor allele frequency
0.0
0.2
0.4
0.6
0.8
1.0
ASW
CEU
CHB/CHD/JPT
GIH
LWK
MEX
MKK
TSI
YRI
Ethnic group

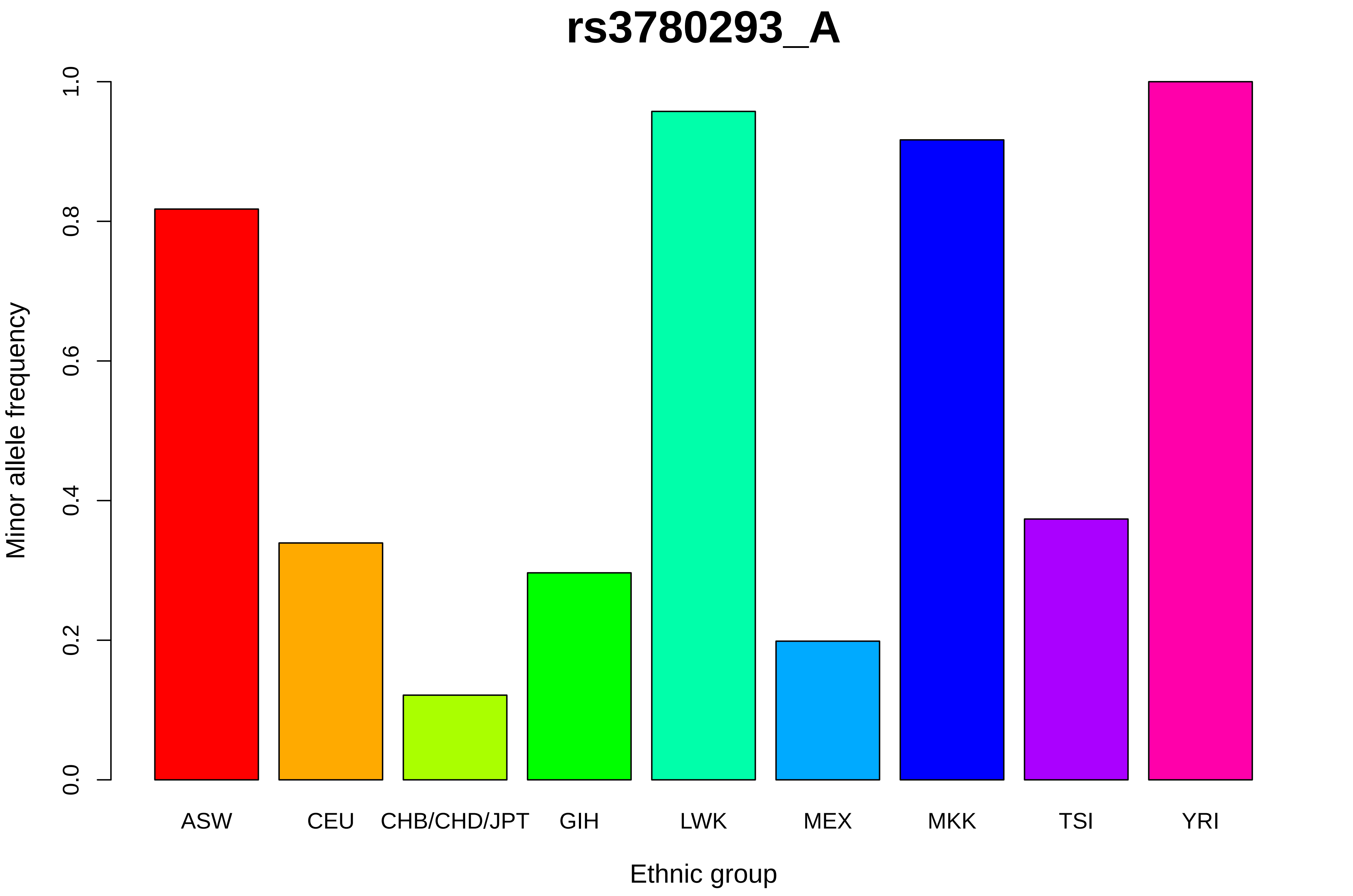
rs3780293_A
Minor allele frequency
0.0
0.2
0.4
0.6
0.8
1.0
ASW
CEU
CHB/CHD/JPT
GIH
LWK
MEX
MKK
TSI
YRI
Ethnic group

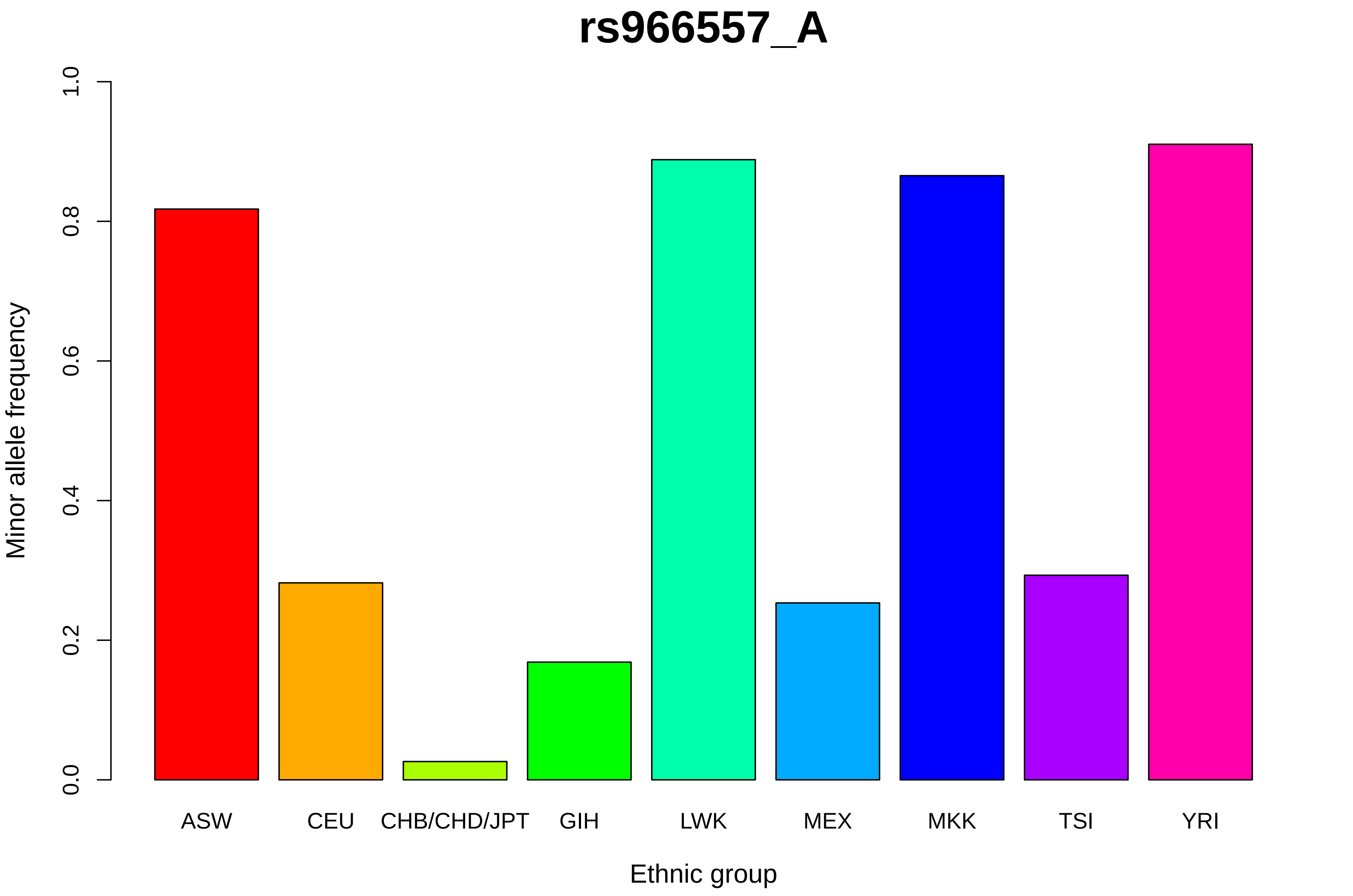

rs966557_A
Minor allele frequency
0.0
0.2
0.4
0.6
0.8
1.0
ASW
CEU
CHB/CHD/JPT
GIH
LWK
MEX
MKK
TSI
YRI
Ethnic group

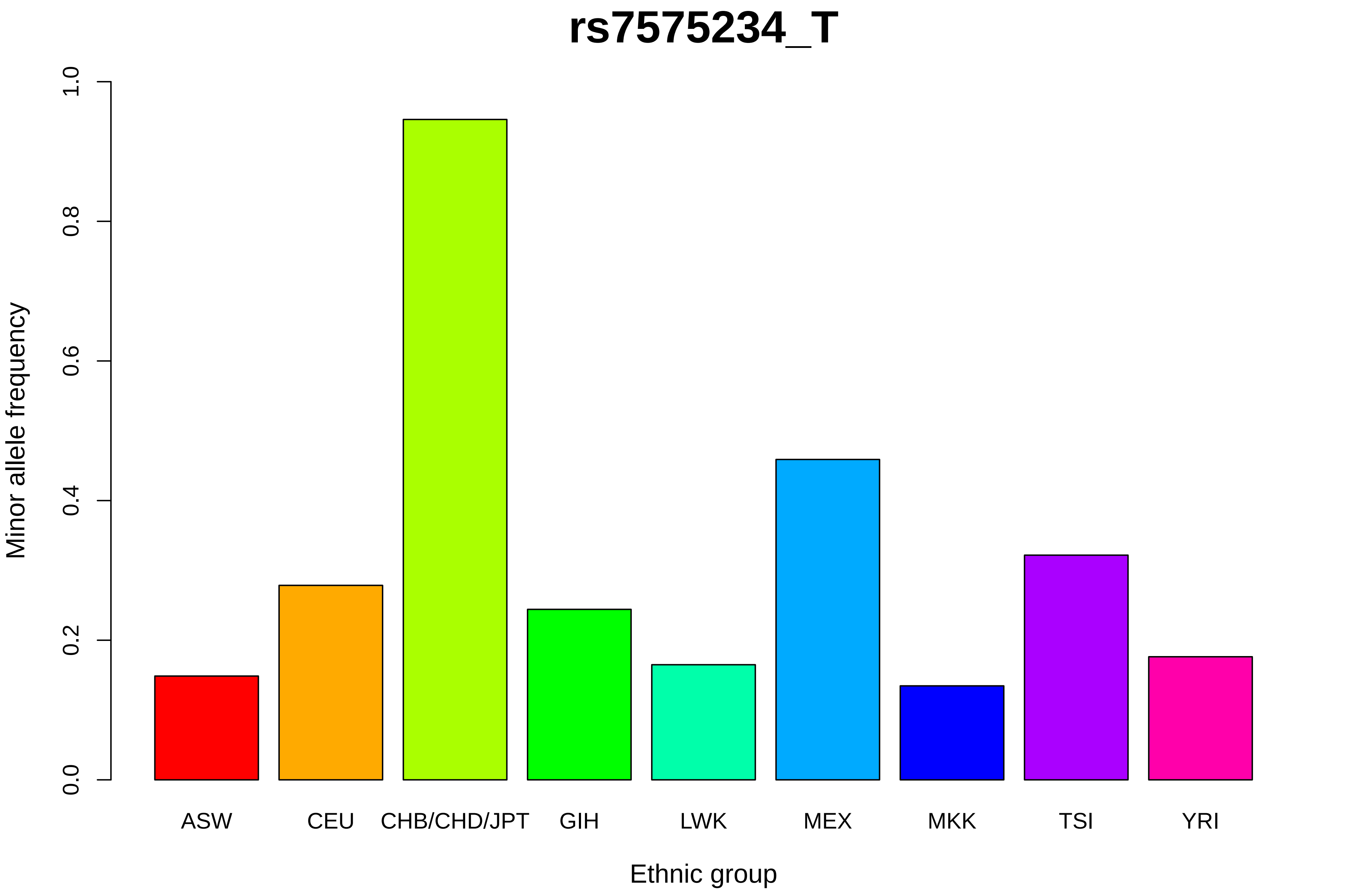

rs7575234_T
Minor allele frequency
0.0
0.2
0.4
0.6
0.8
1.0
ASW
CEU
CHB/CHD/JPT
GIH
LWK
MEX
MKK
TSI
YRI
Ethnic group

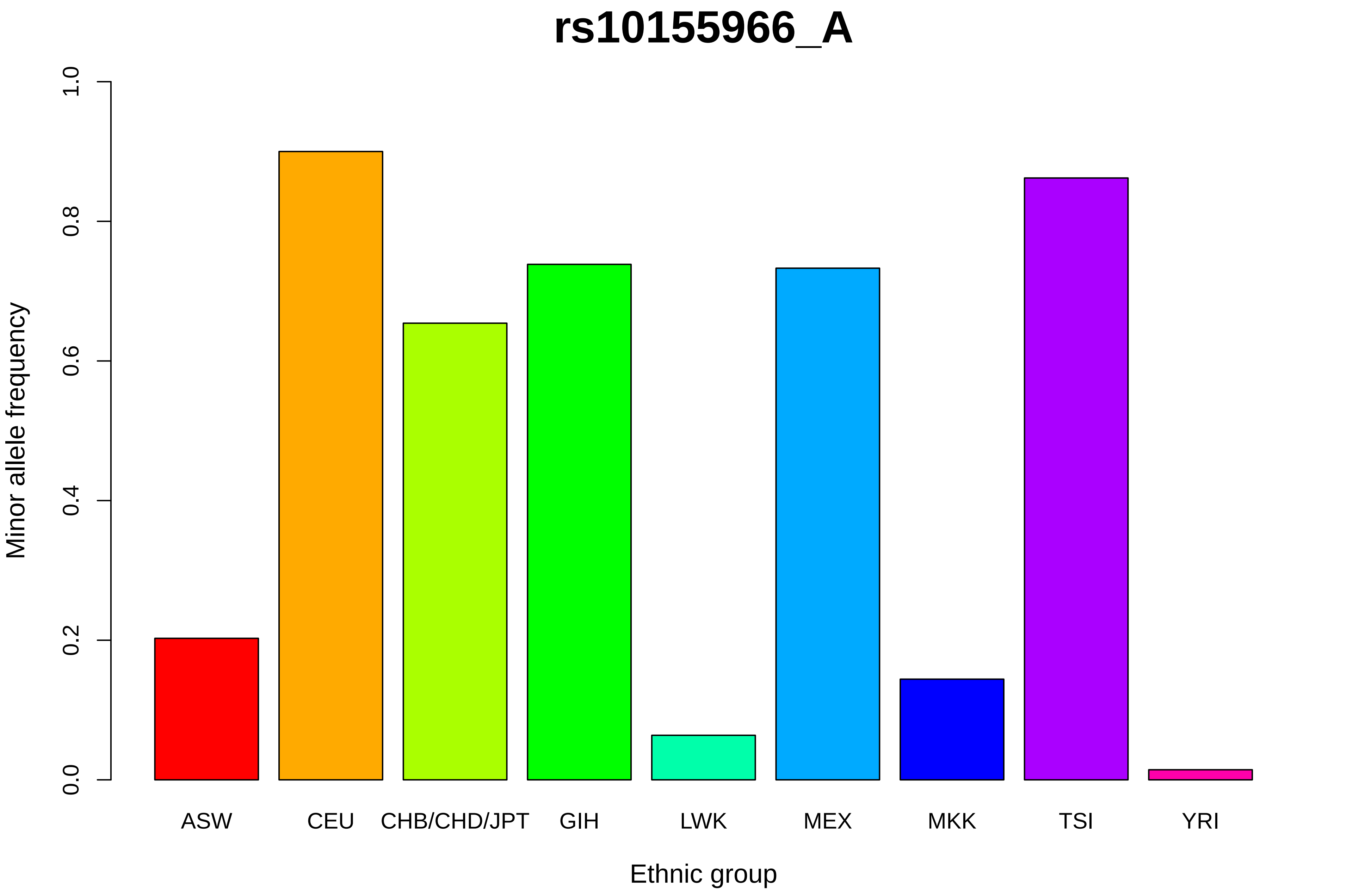
rs10155966_A
Minor allele frequency
0.0
0.2
0.4
0.6
0.8
1.0
ASW
CEU
CHB/CHD/JPT
GIH
LWK
MEX
MKK
TSI
YRI
Ethnic group

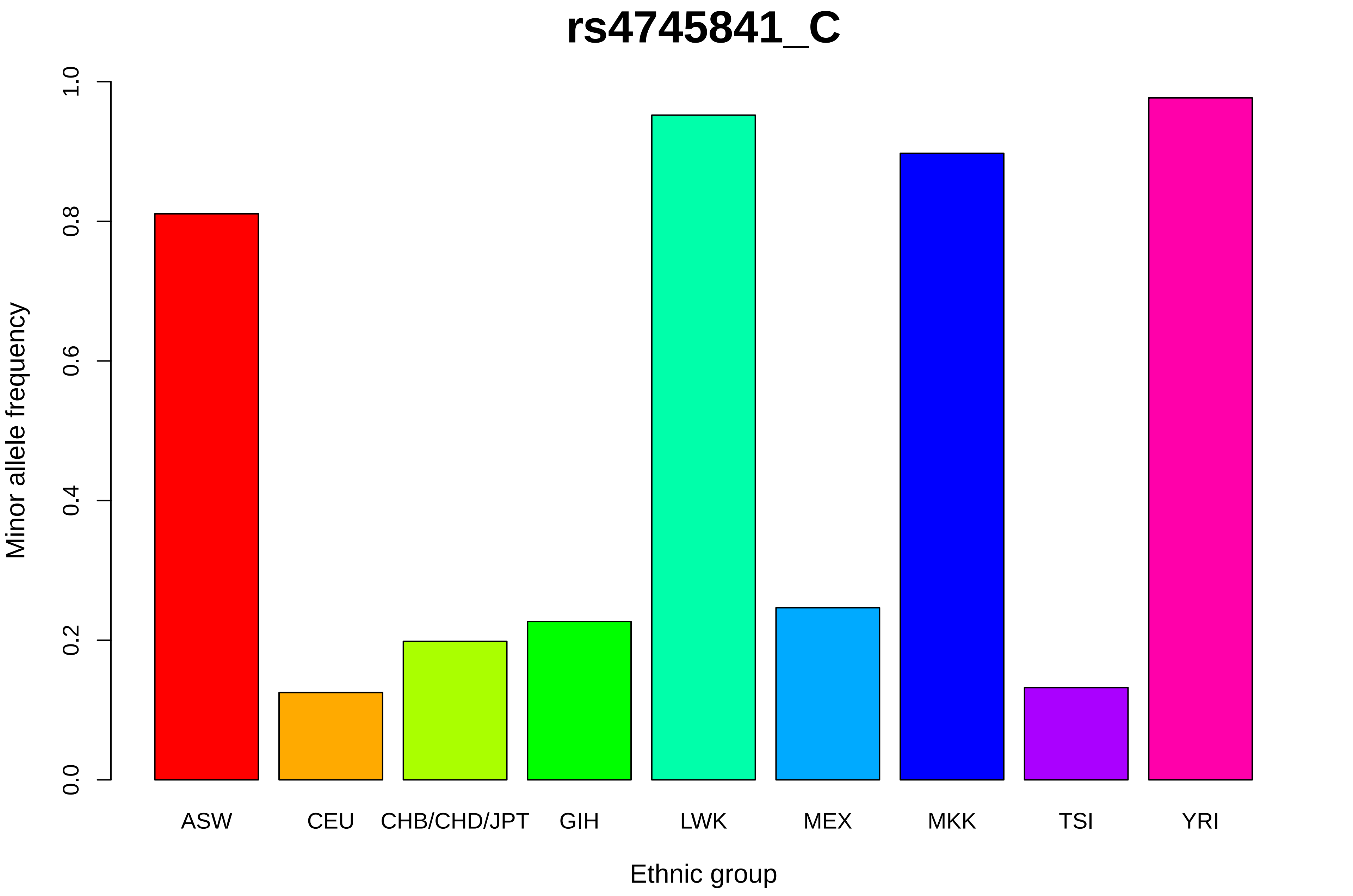
rs4745841_C
Minor allele frequency
0.0
0.2
0.4
0.6
0.8
1.0
ASW
CEU
CHB/CHD/JPT
GIH
LWK
MEX
MKK
TSI
YRI
Ethnic group

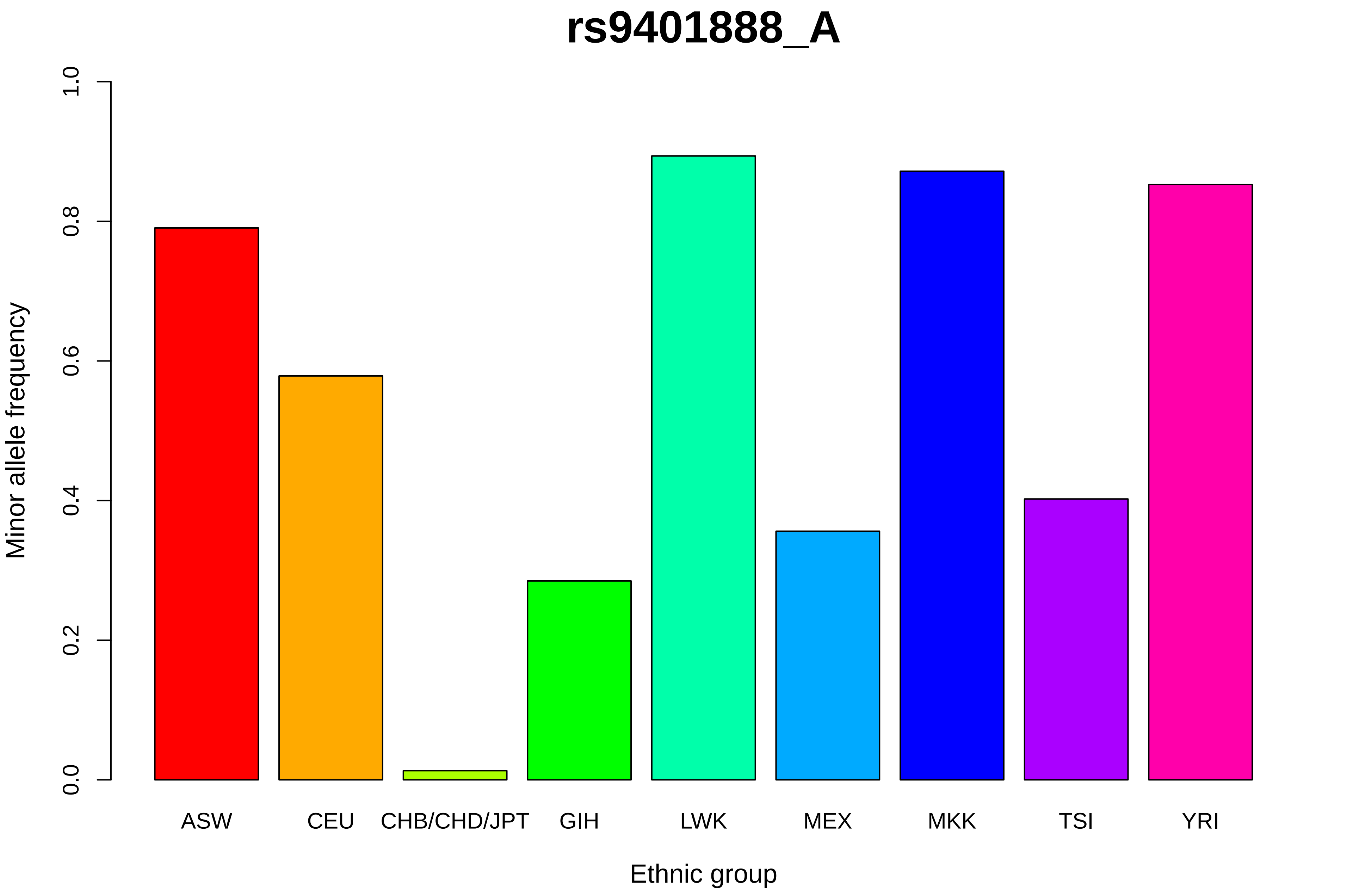
rs9401888_A
Minor allele frequency
0.0
0.2
0.4
0.6
0.8
1.0
ASW
CEU
CHB/CHD/JPT
GIH
LWK
MEX
MKK
TSI
YRI
Ethnic group

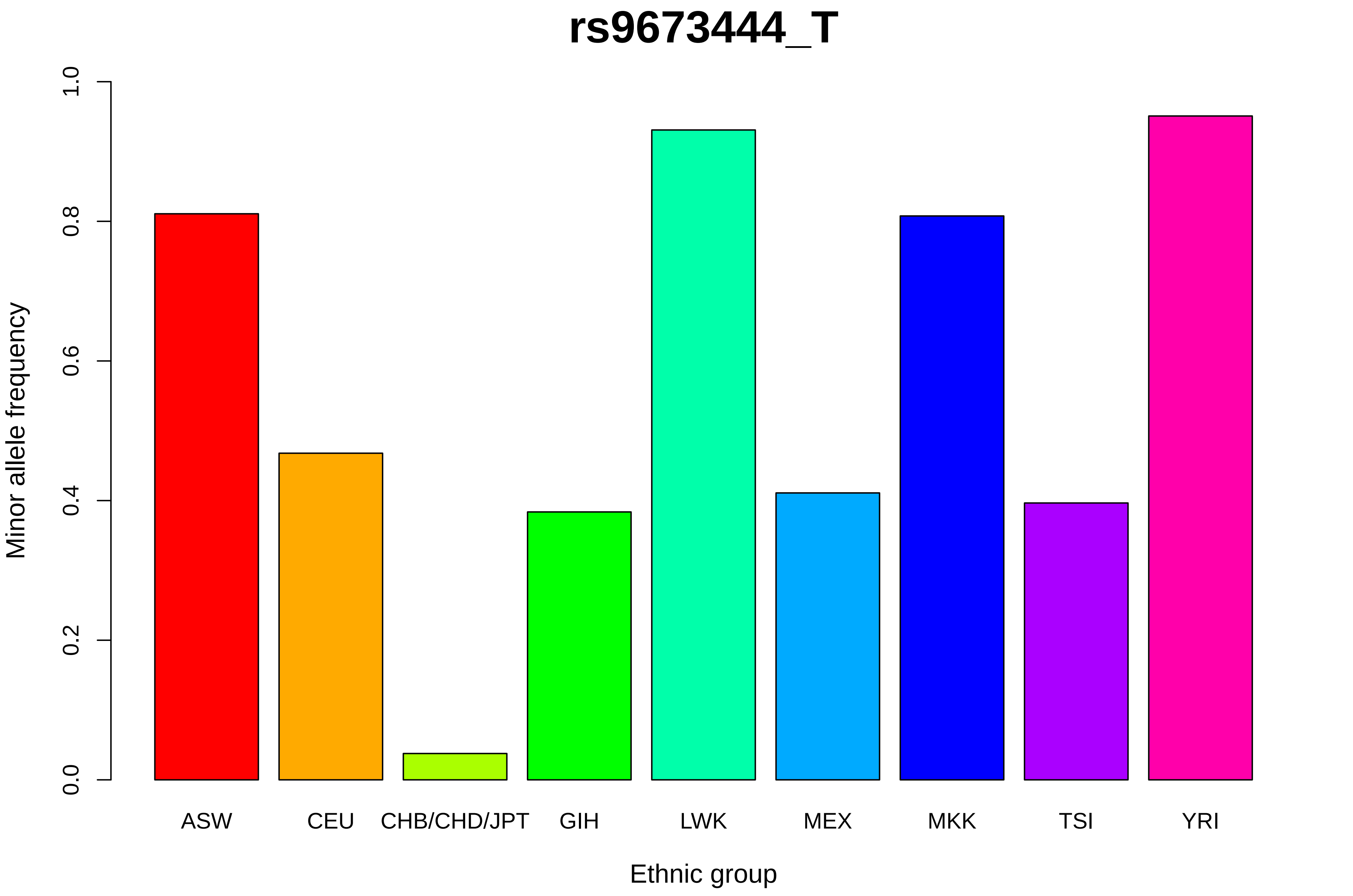
rs9673444_T
Minor allele frequency
0.0
0.2
0.4
0.6
0.8
1.0
ASW
CEU
CHB/CHD/JPT
GIH
LWK
MEX
MKK
TSI
YRI
Ethnic group

rs1456235_T

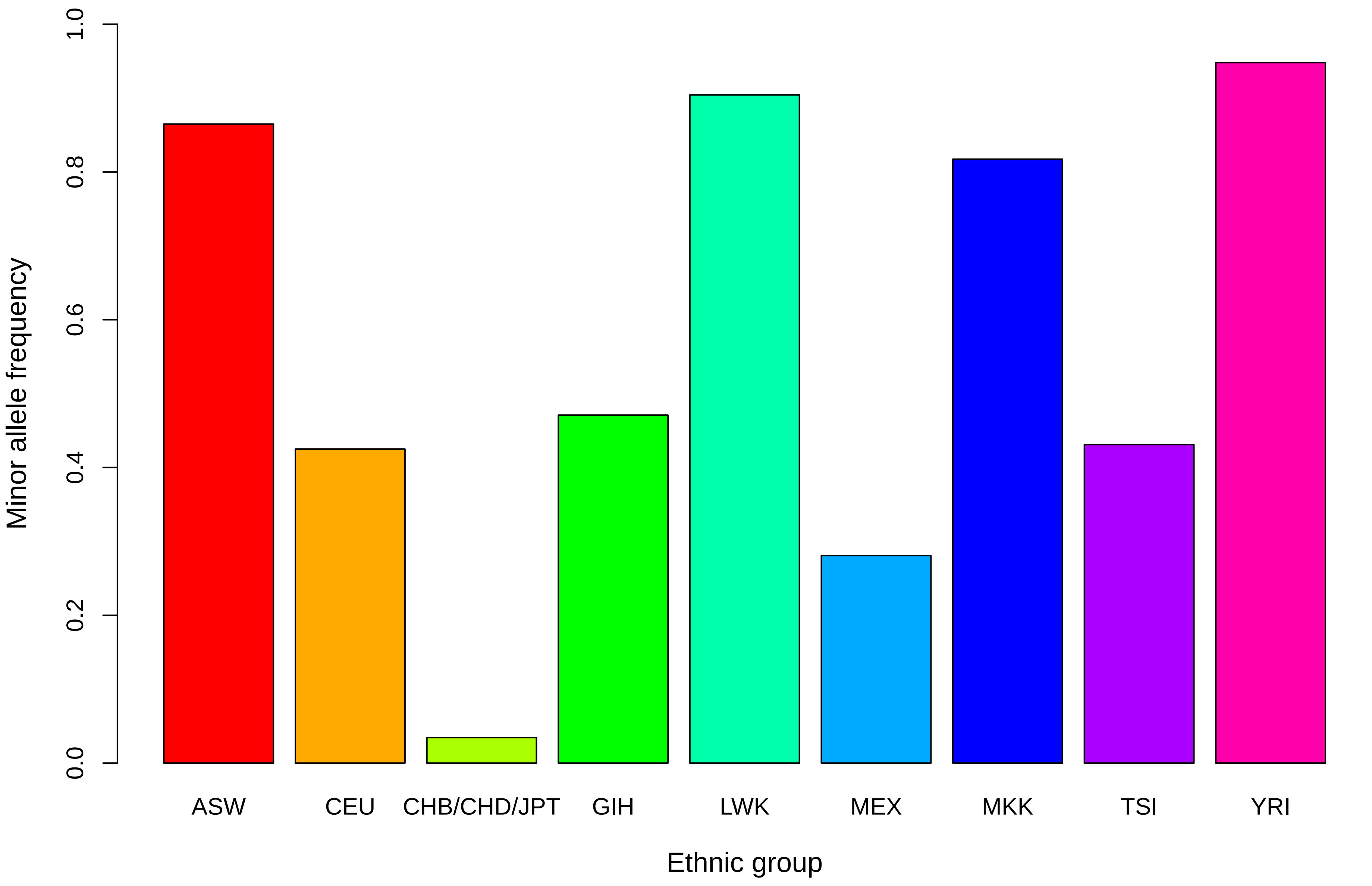

Minor allele frequency
0.0
0.2
0.4
0.6
0.8
1.0
ASW
CEU
CHB/CHD/JPT
GIH
LWK
MEX
MKK
TSI
YRI
Ethnic group

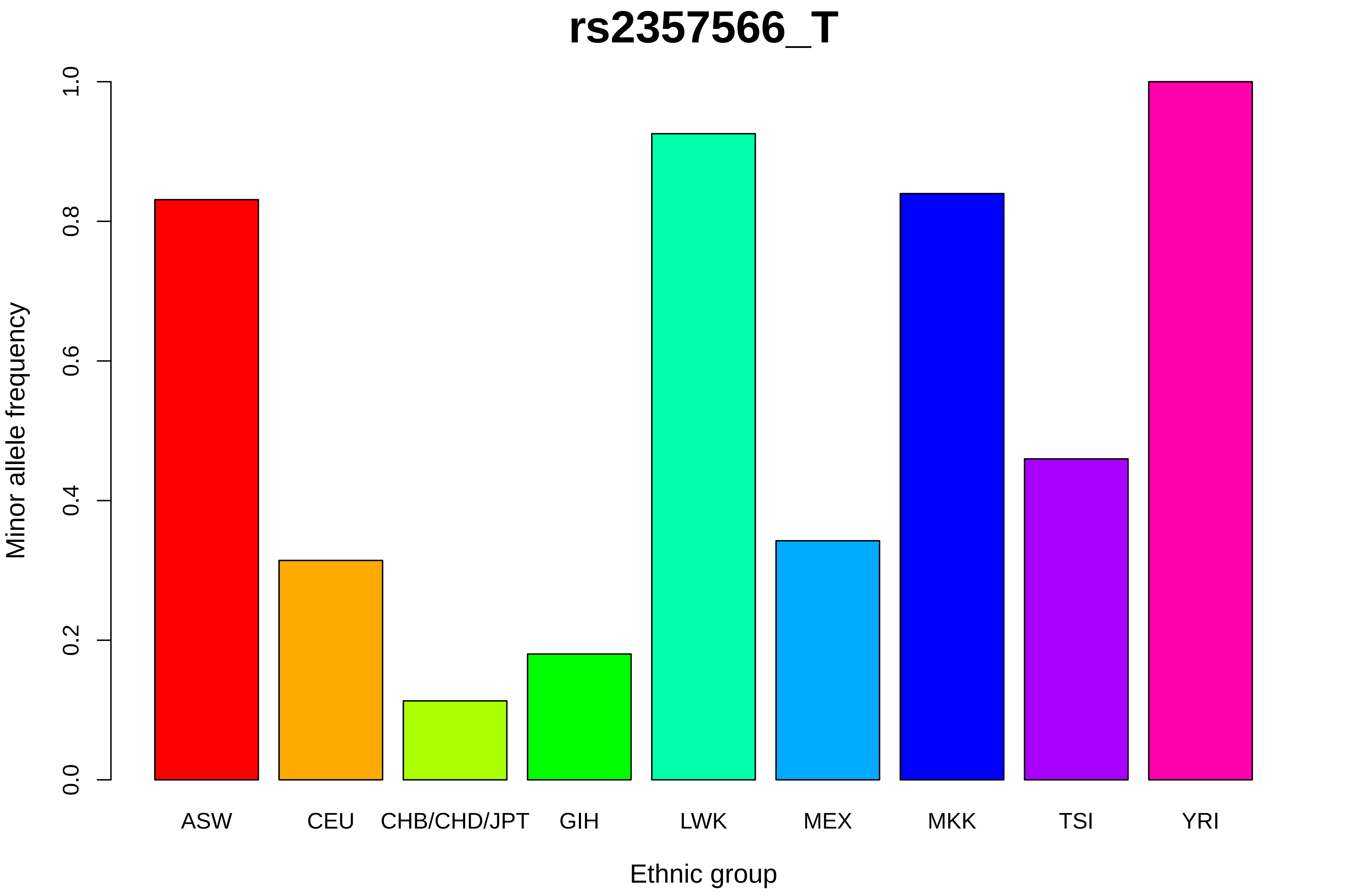
rs2357566_T
Minor allele frequency
0.0
0.2
0.4
0.6
0.8
1.0
ASW
CEU
CHB/CHD/JPT
GIH
LWK
MEX
MKK
TSI
YRI
Ethnic group

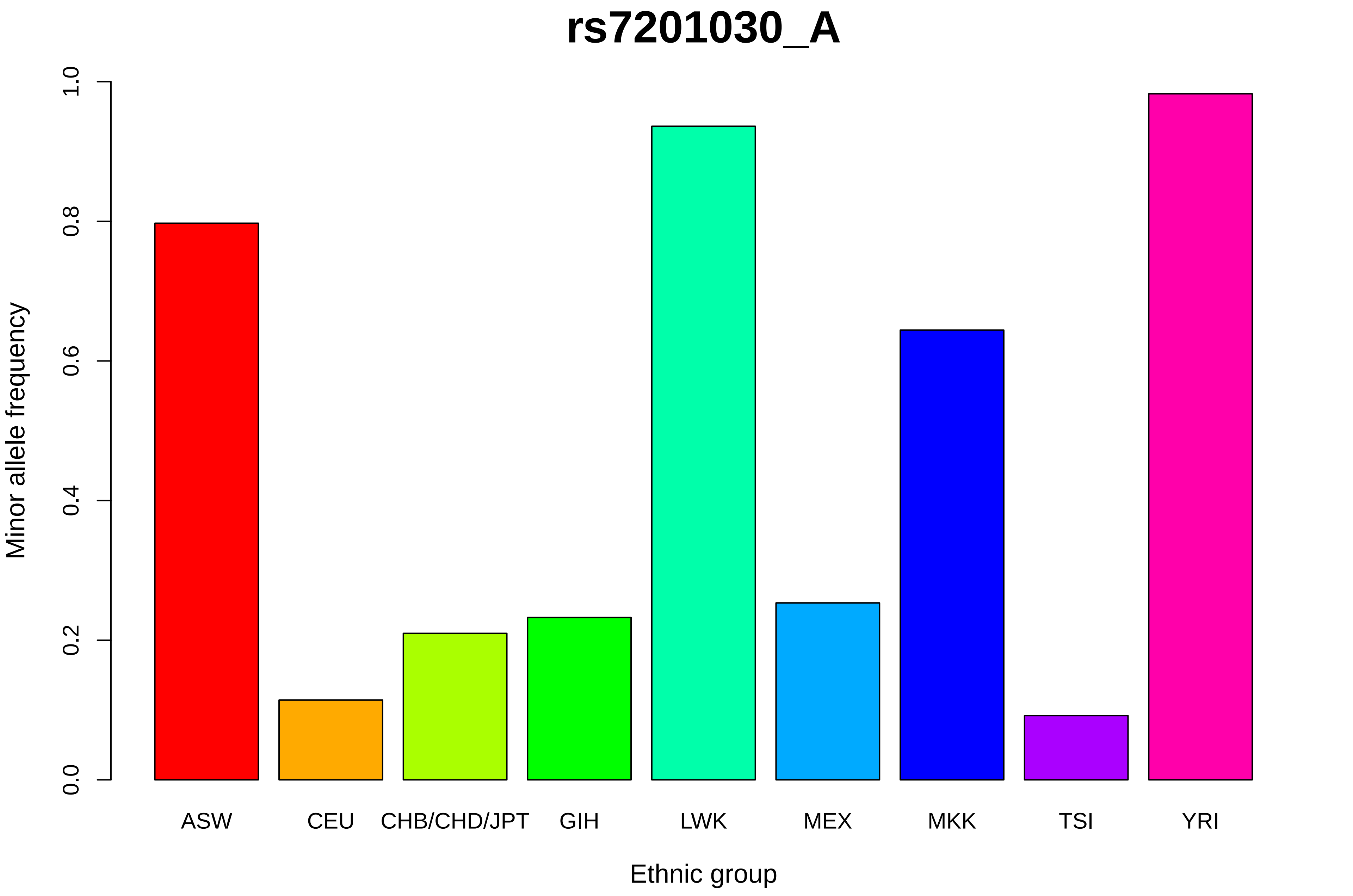
rs7201030_A
Minor allele frequency
0.0
0.2
0.4
0.6
0.8
1.0
ASW
CEU
CHB/CHD/JPT
GIH
LWK
MEX
MKK
TSI
YRI
Ethnic group

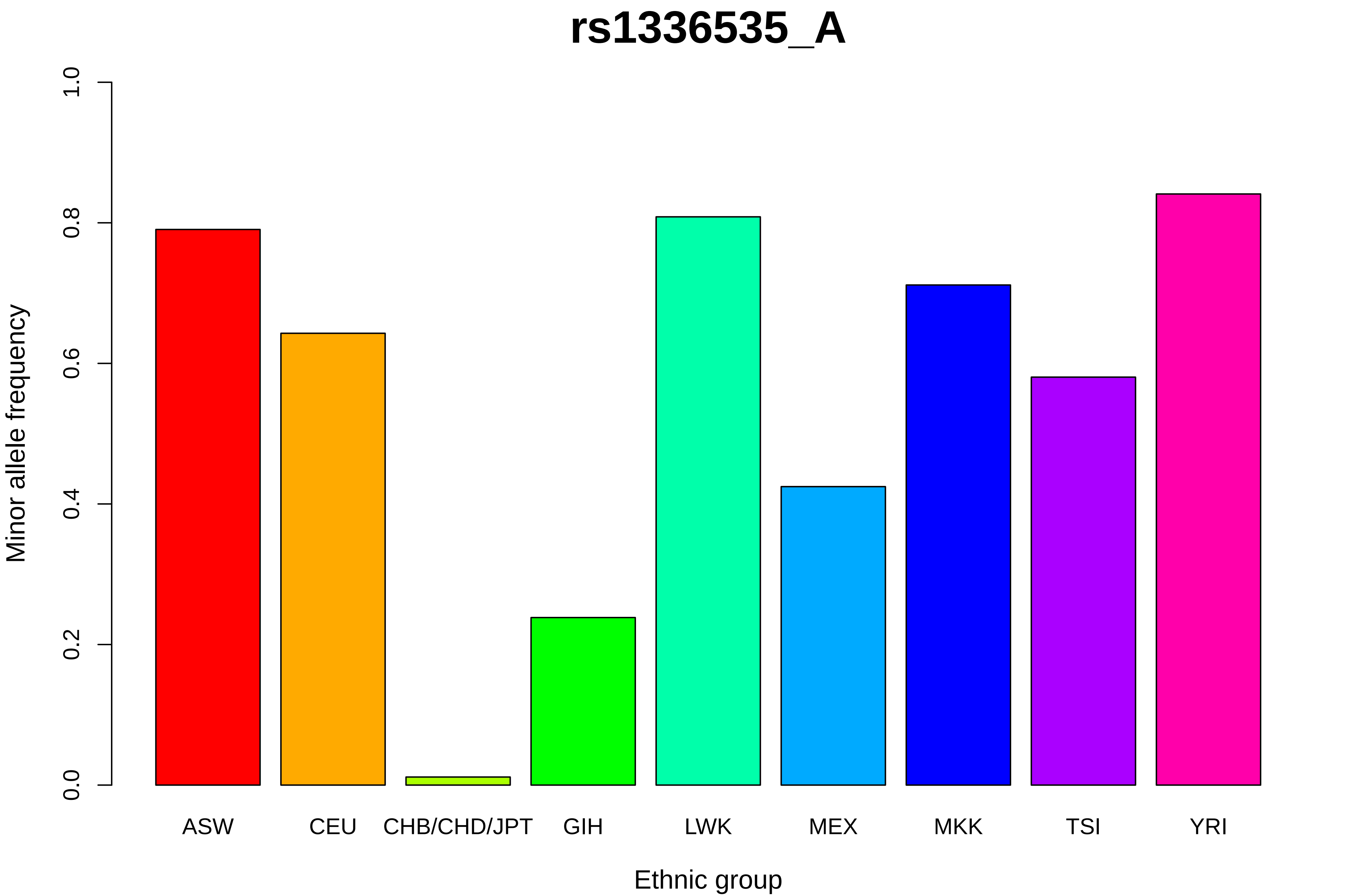
rs1336535_A
Minor allele frequency
0.0
0.2
0.4
0.6
0.8
1.0
ASW
CEU
CHB/CHD/JPT
GIH
LWK
MEX
MKK
TSI
YRI
Ethnic group

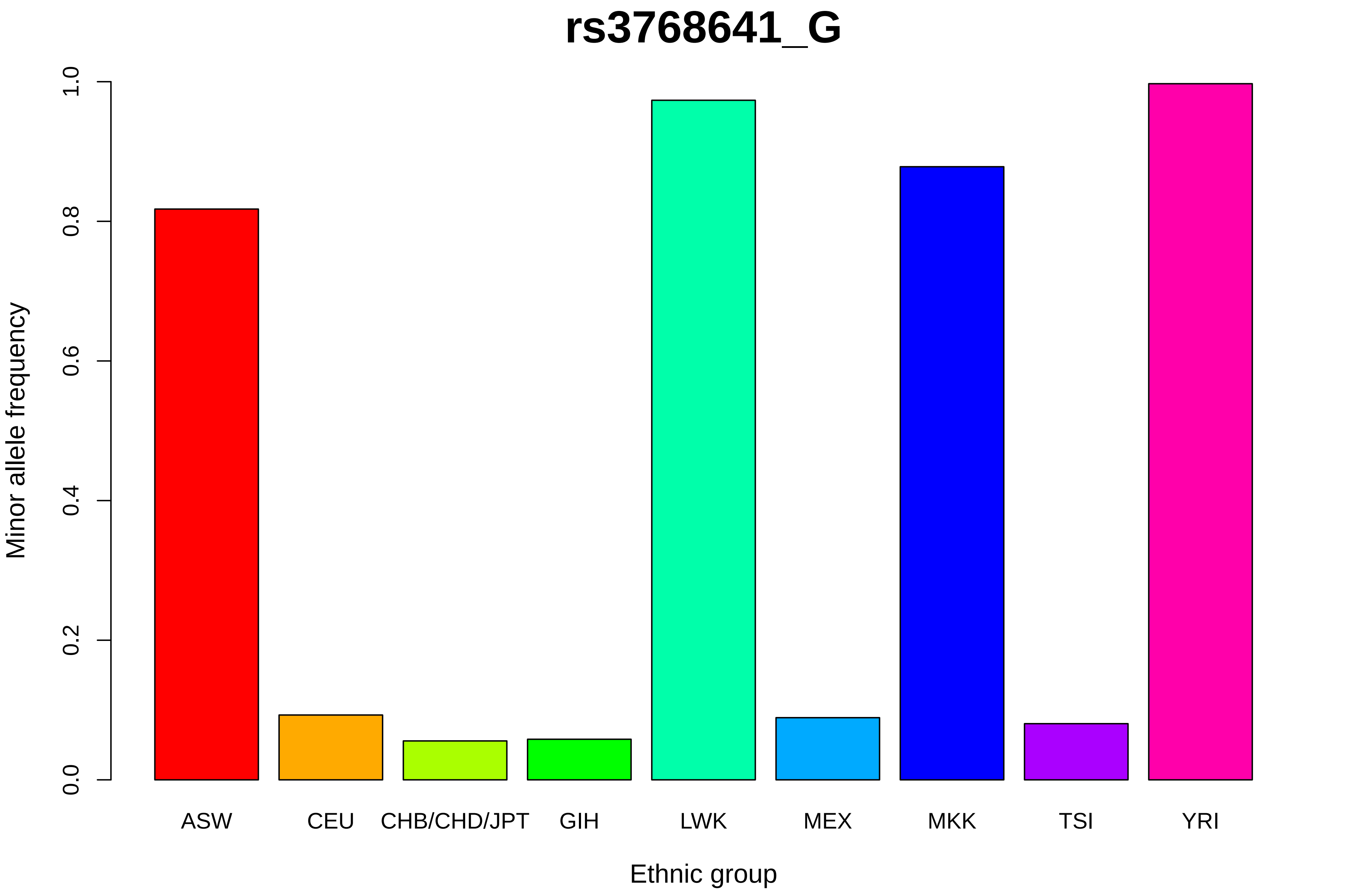
rs3768641_G
Minor allele frequency
0.0
0.2
0.4
0.6
0.8
1.0
ASW
CEU
CHB/CHD/JPT
GIH
LWK
MEX
MKK
TSI
YRI
Ethnic group

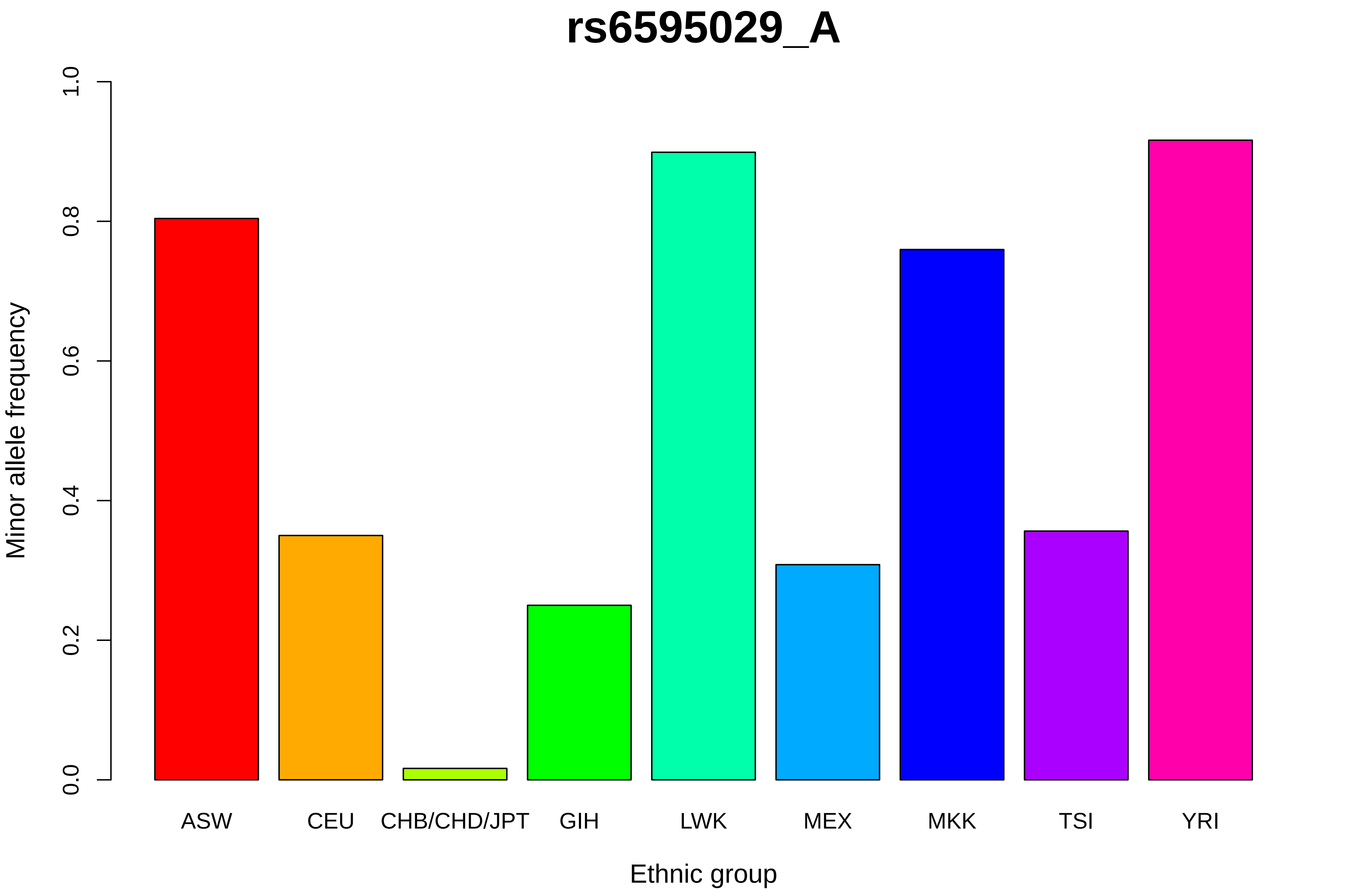
rs6595029_A
Minor allele frequency
0.0
0.2
0.4
0.6
0.8
1.0
ASW
CEU
CHB/CHD/JPT
GIH
LWK
MEX
MKK
TSI
YRI
Ethnic group

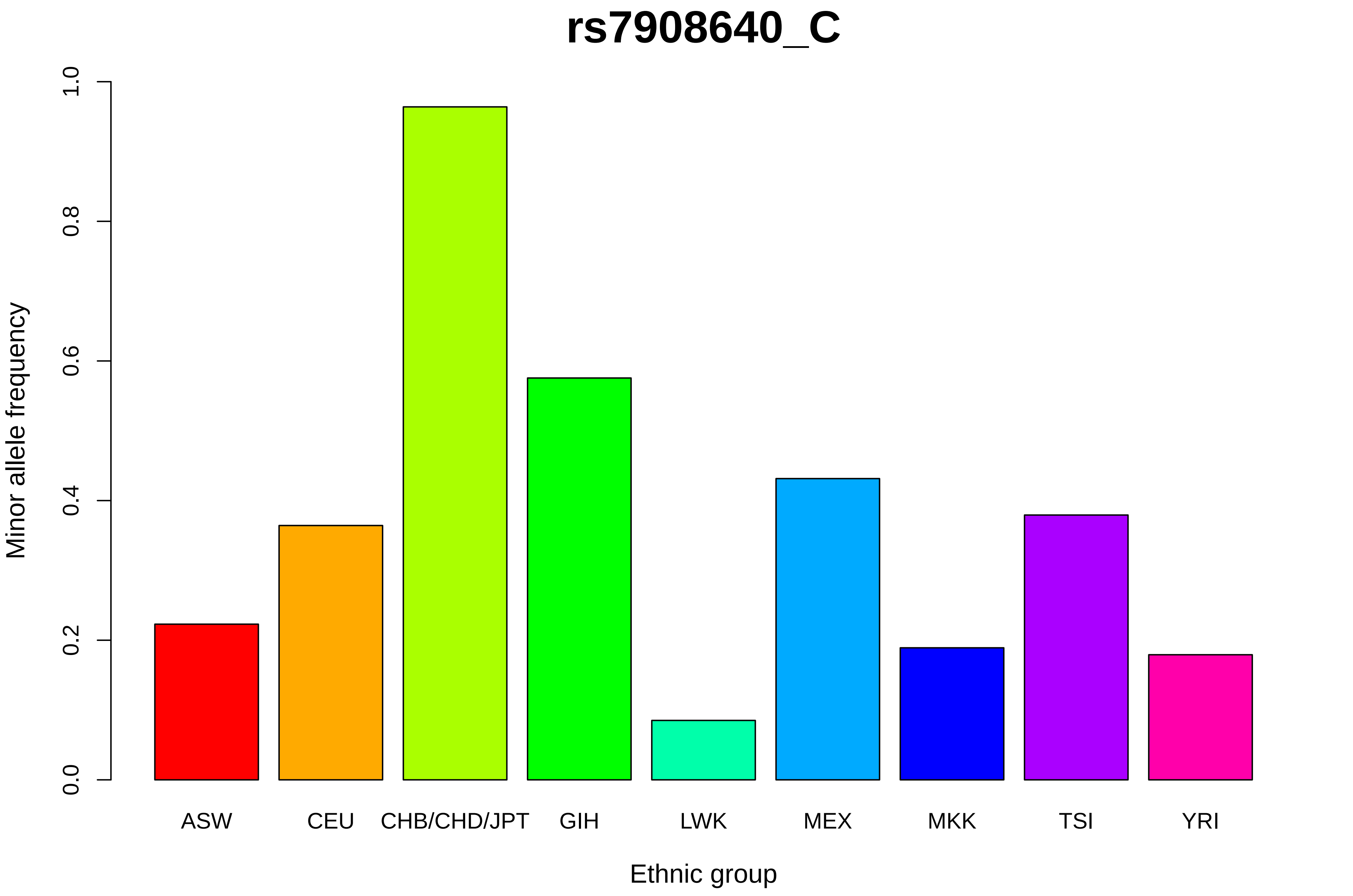
rs7908640_C
Minor allele frequency
0.0
0.2
0.4
0.6
0.8
1.0
ASW
CEU
CHB/CHD/JPT
GIH
LWK
MEX
MKK
TSI
YRI
Ethnic group

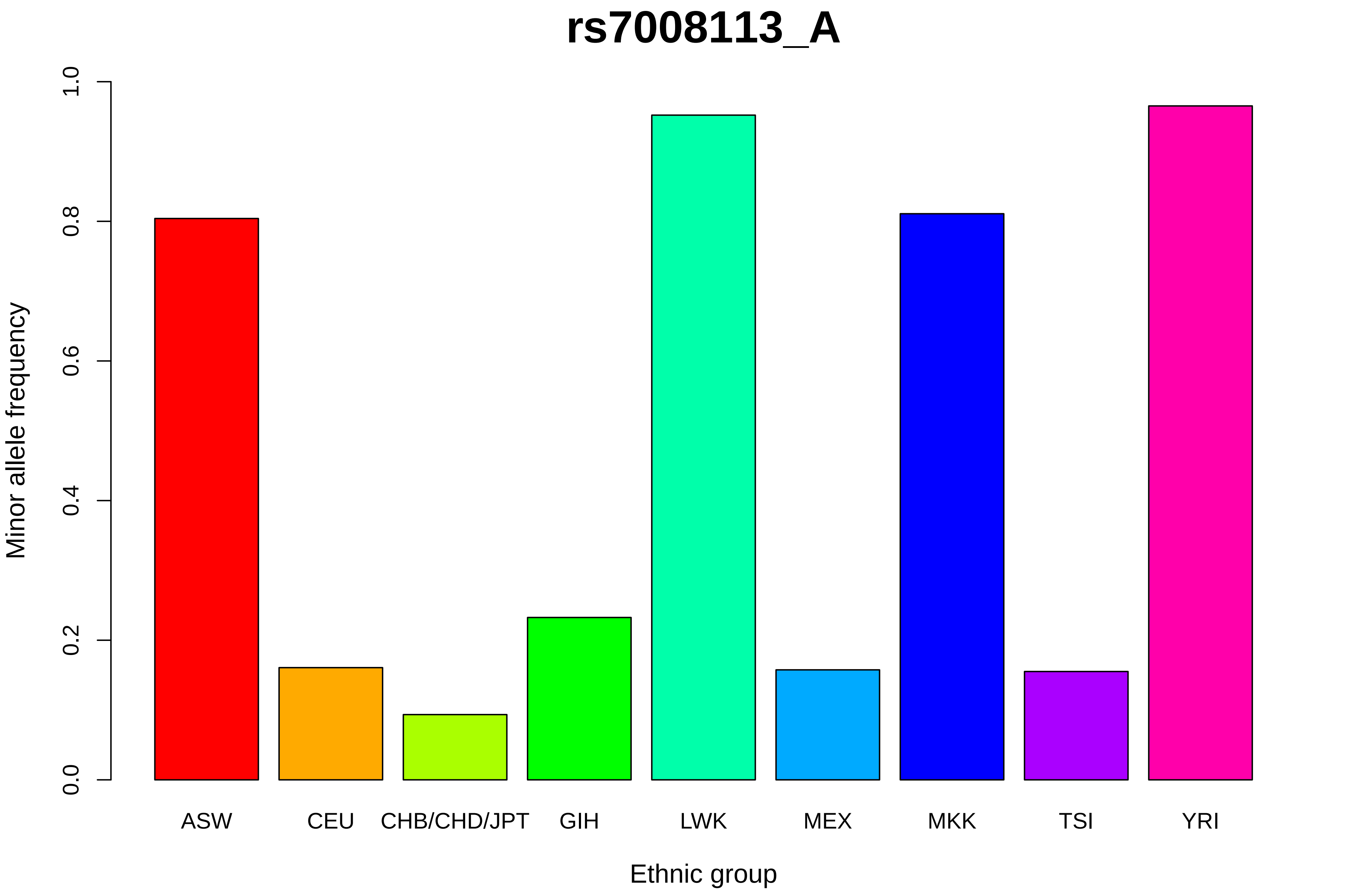

rs7008113_A
Minor allele frequency
0.0
0.2
0.4
0.6
0.8
1.0
ASW
CEU
CHB/CHD/JPT
GIH
LWK
MEX
MKK
TSI
YRI
Ethnic group

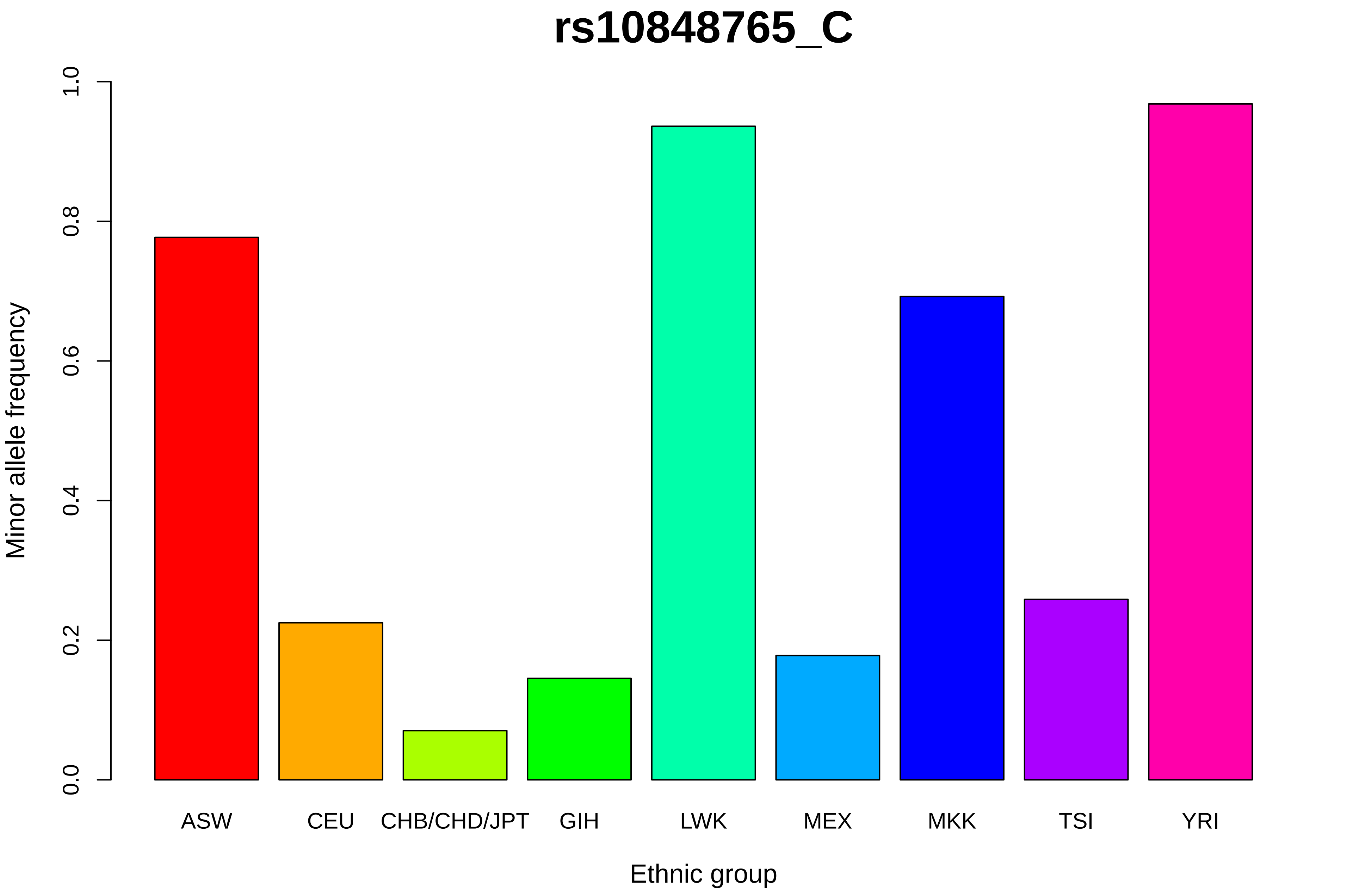
rs10848765_C
Minor allele frequency
0.0
0.2
0.4
0.6
0.8
1.0
ASW
CEU
CHB/CHD/JPT
GIH
LWK
MEX
MKK
TSI
YRI
Ethnic group

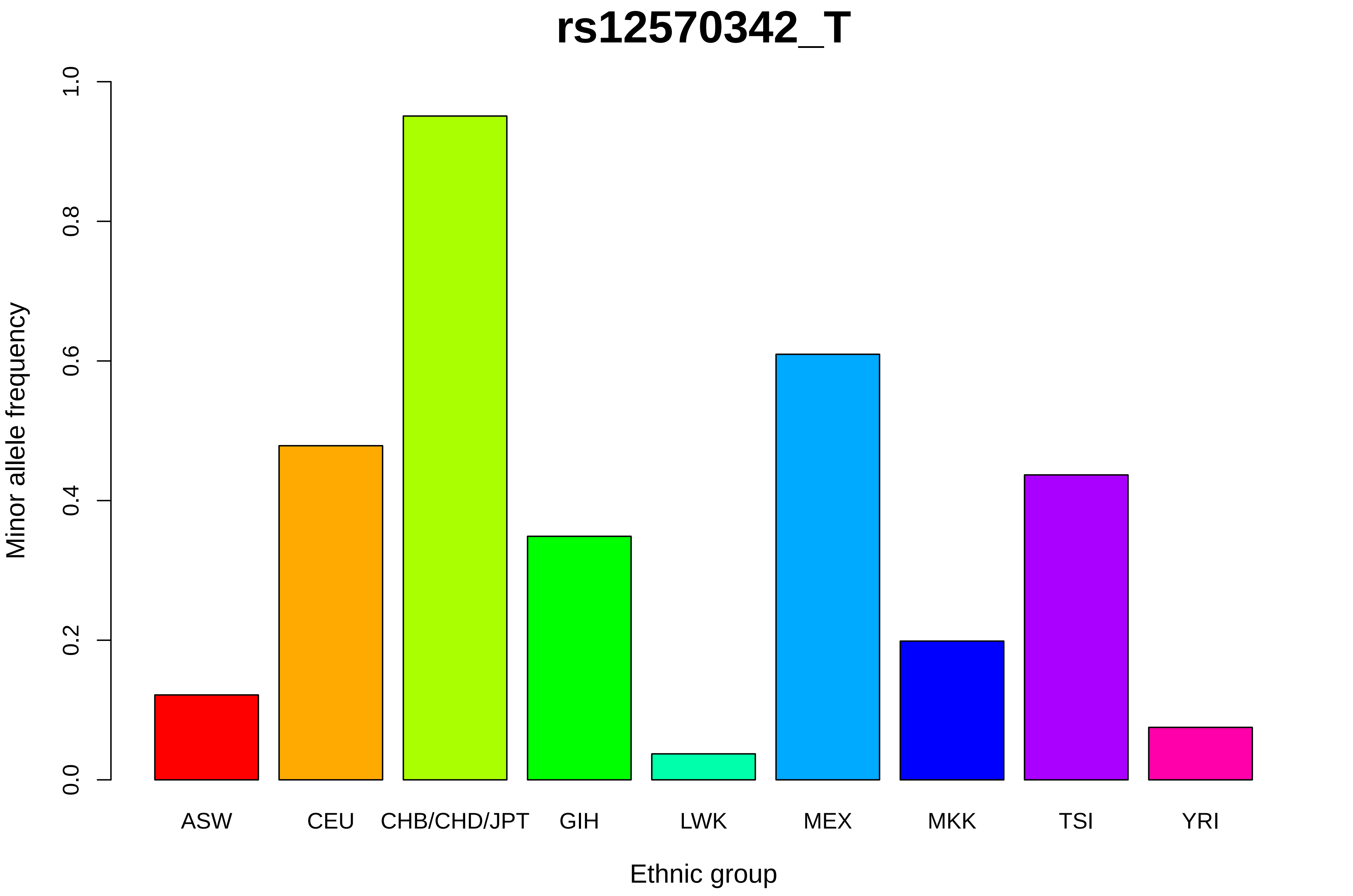
rs12570342_T
Minor allele frequency
0.0
0.2
0.4
0.6
0.8
1.0
ASW
CEU
CHB/CHD/JPT
GIH
LWK
MEX
MKK
TSI
YRI
Ethnic group

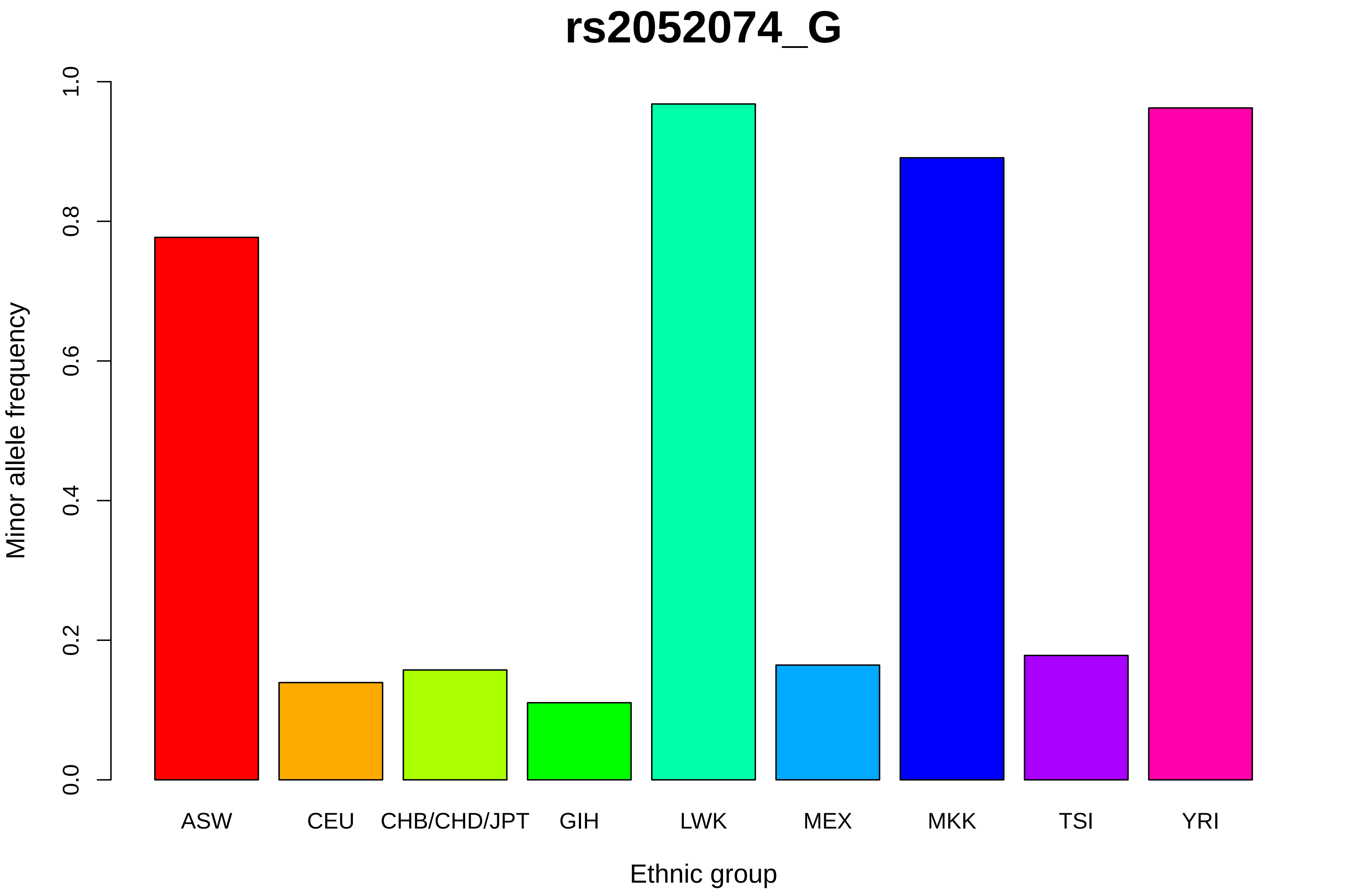
rs2052074_G
Minor allele frequency
0.0
0.2
0.4
0.6
0.8
1.0
ASW
CEU
CHB/CHD/JPT
GIH
LWK
MEX
MKK
TSI
YRI
Ethnic group

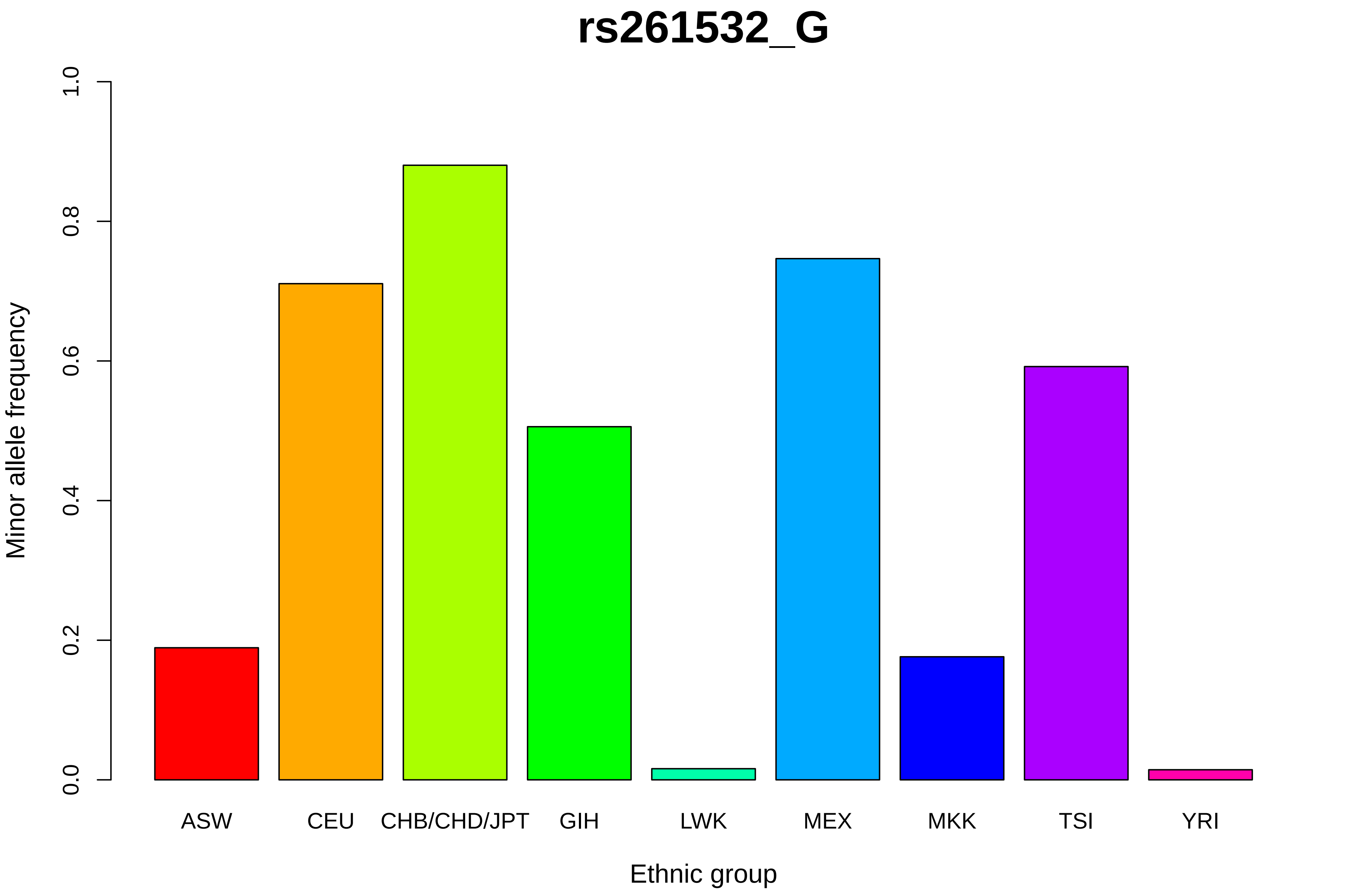

rs261532_G
Minor allele frequency
0.0
0.2
0.4
0.6
0.8
1.0
ASW
CEU
CHB/CHD/JPT
GIH
LWK
MEX
MKK
TSI
YRI
Ethnic group

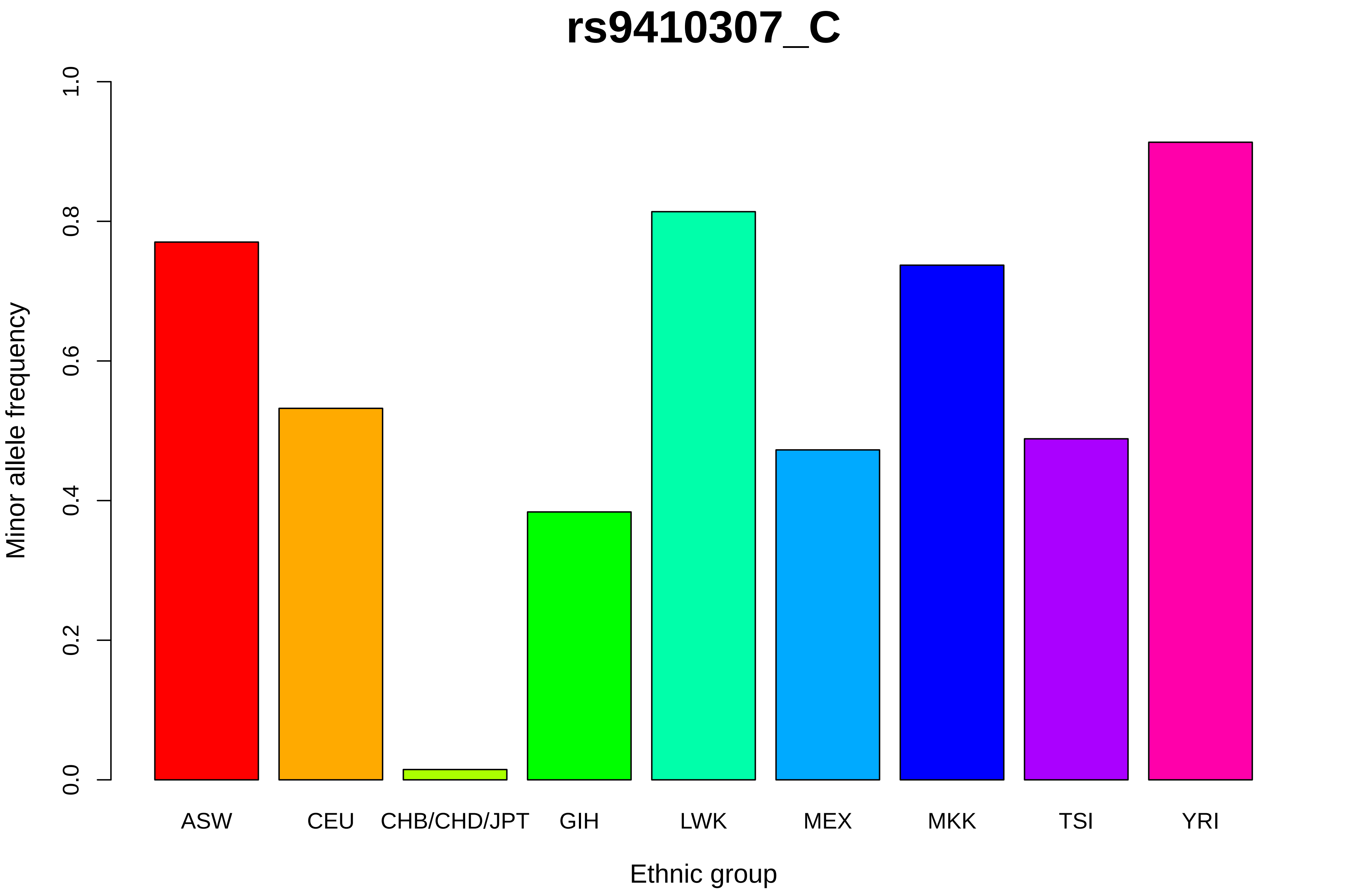
rs9410307_C
Minor allele frequency
0.0
0.2
0.4
0.6
0.8
1.0
ASW
CEU
CHB/CHD/JPT
GIH
LWK
MEX
MKK
TSI
YRI
Ethnic group

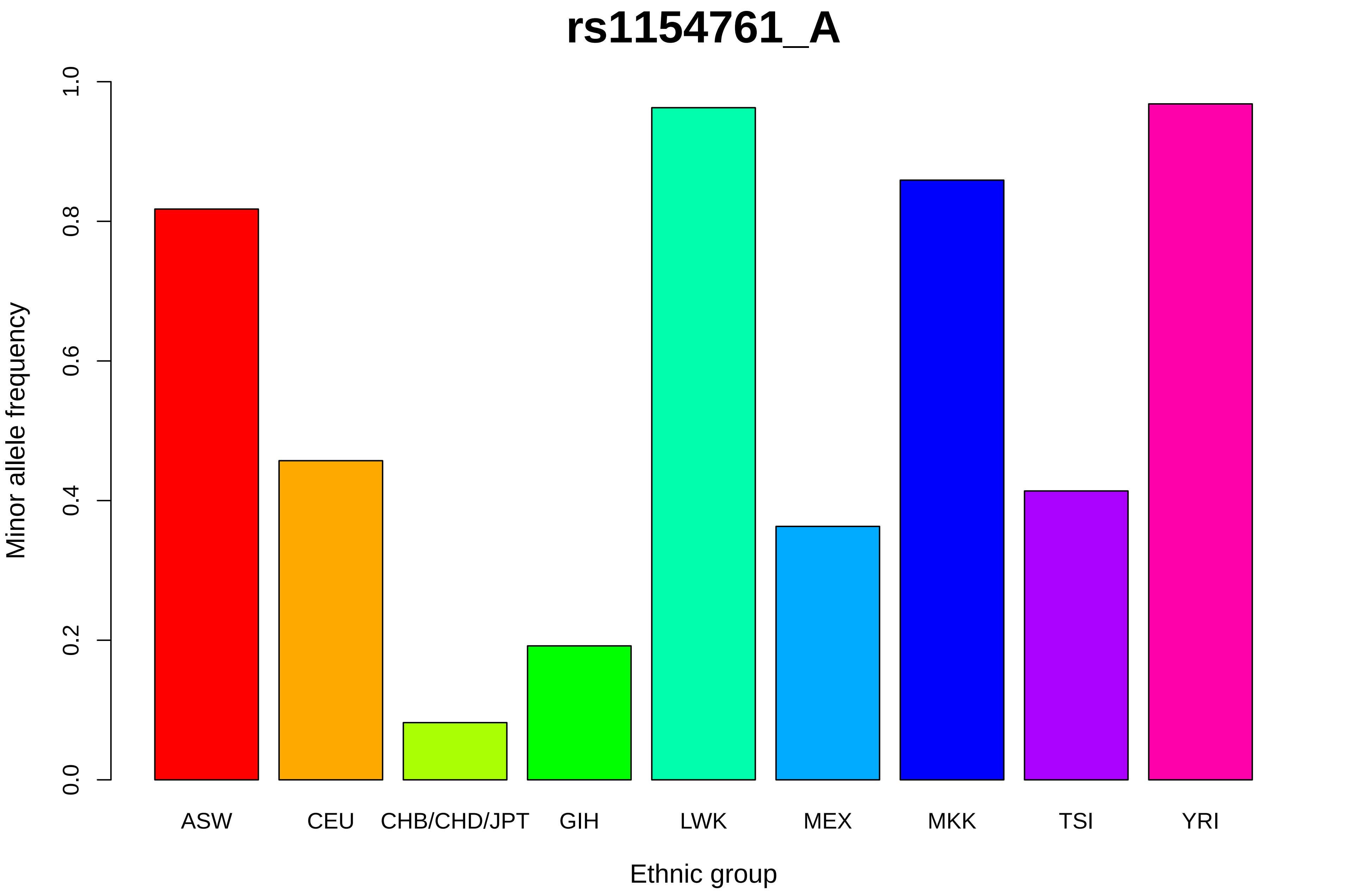

rs1154761_A
Minor allele frequency
0.0
0.2
0.4
0.6
0.8
1.0
ASW
CEU
CHB/CHD/JPT
GIH
LWK
MEX
MKK
TSI
YRI
Ethnic group

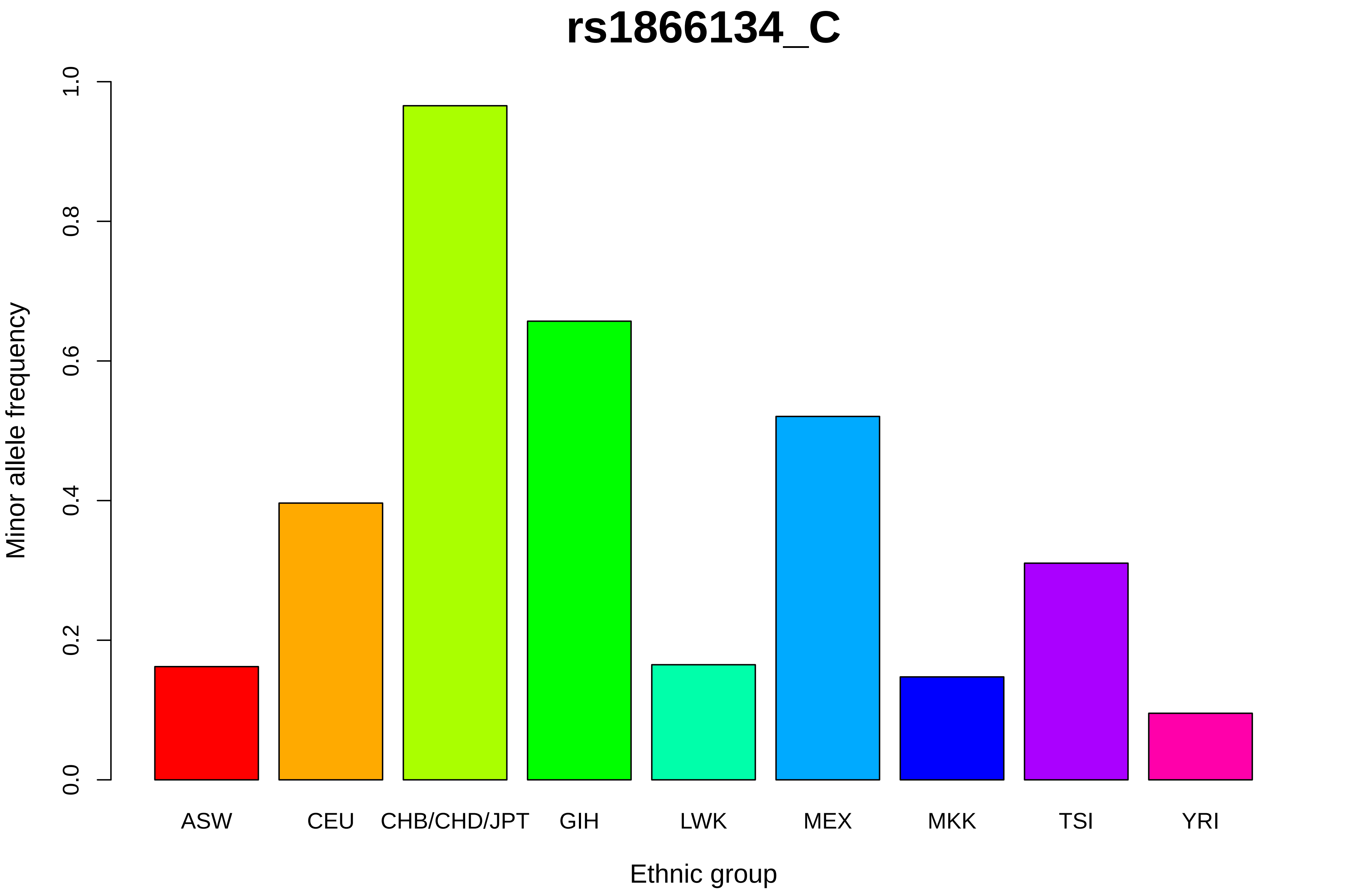
rs1866134_C
Minor allele frequency
0.0
0.2
0.4
0.6
0.8
1.0
ASW
CEU
CHB/CHD/JPT
GIH
LWK
MEX
MKK
TSI
YRI
Ethnic group

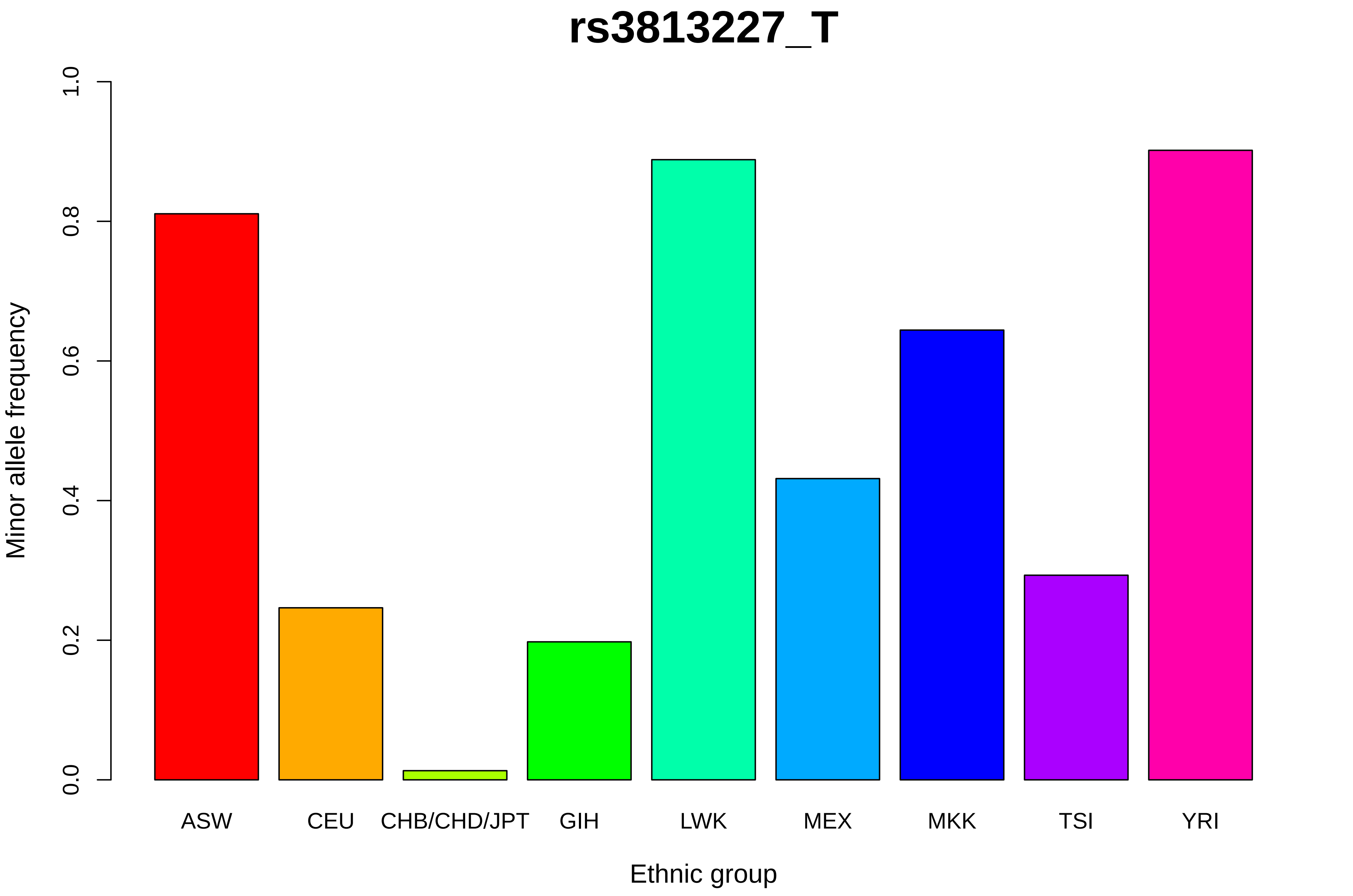
rs3813227_T
Minor allele frequency
0.0
0.2
0.4
0.6
0.8
1.0
ASW
CEU
CHB/CHD/JPT
GIH
LWK
MEX
MKK
TSI
YRI
Ethnic group

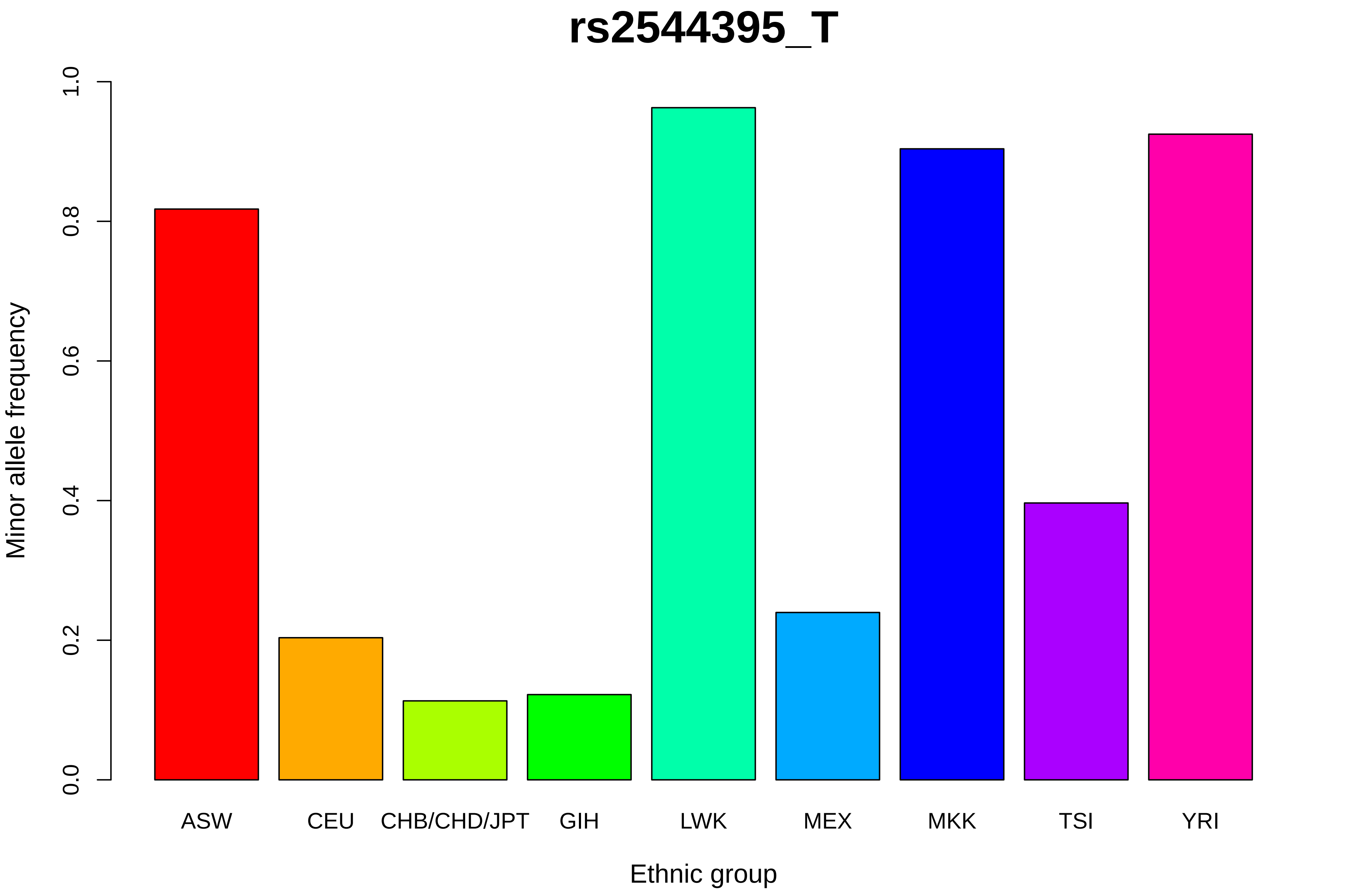
rs2544395_T
Minor allele frequency
0.0
0.2
0.4
0.6
0.8
1.0
ASW
CEU
CHB/CHD/JPT
GIH
LWK
MEX
MKK
TSI
YRI
Ethnic group

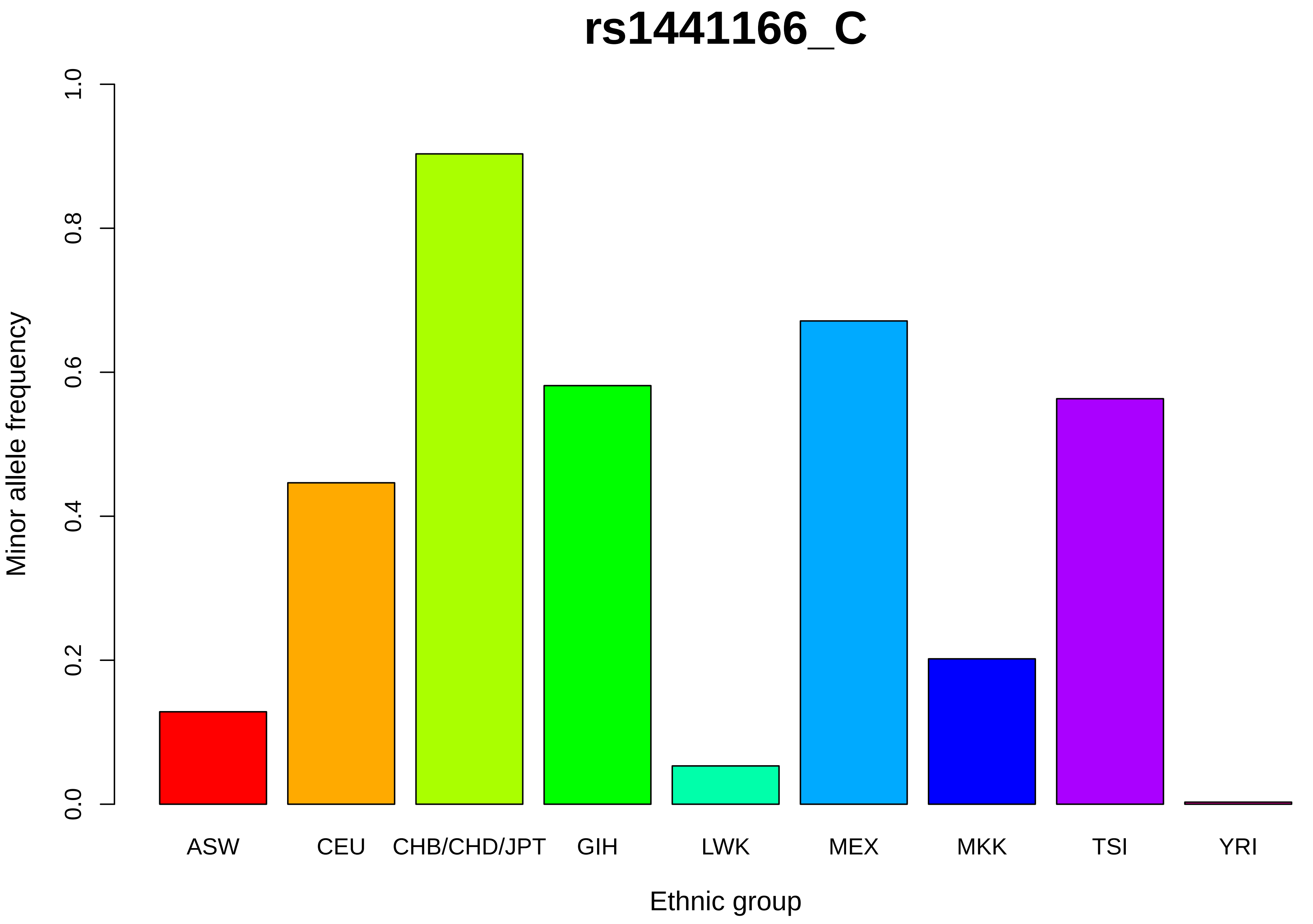

rs1441166_C
Minor allele frequency
0.0
0.2
0.4
0.6
0.8
1.0
ASW
CEU
CHB/CHD/JPT
GIH
LWK
MEX
MKK
TSI
YRI
Ethnic group

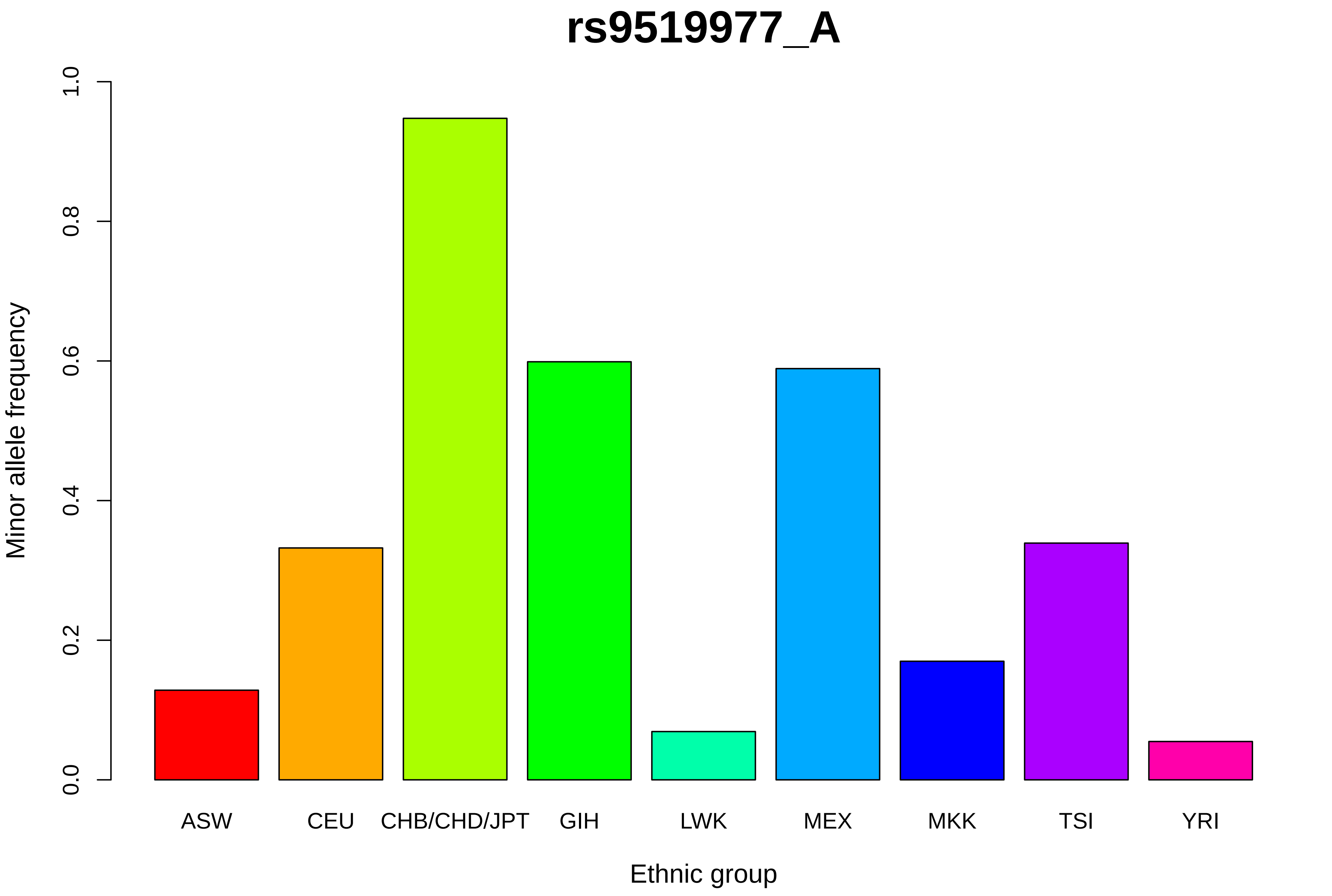
rs9519977_A
Minor allele frequency
0.0
0.2
0.4
0.6
0.8
1.0
ASW
CEU
CHB/CHD/JPT
GIH
LWK
MEX
MKK
TSI
YRI
Ethnic group

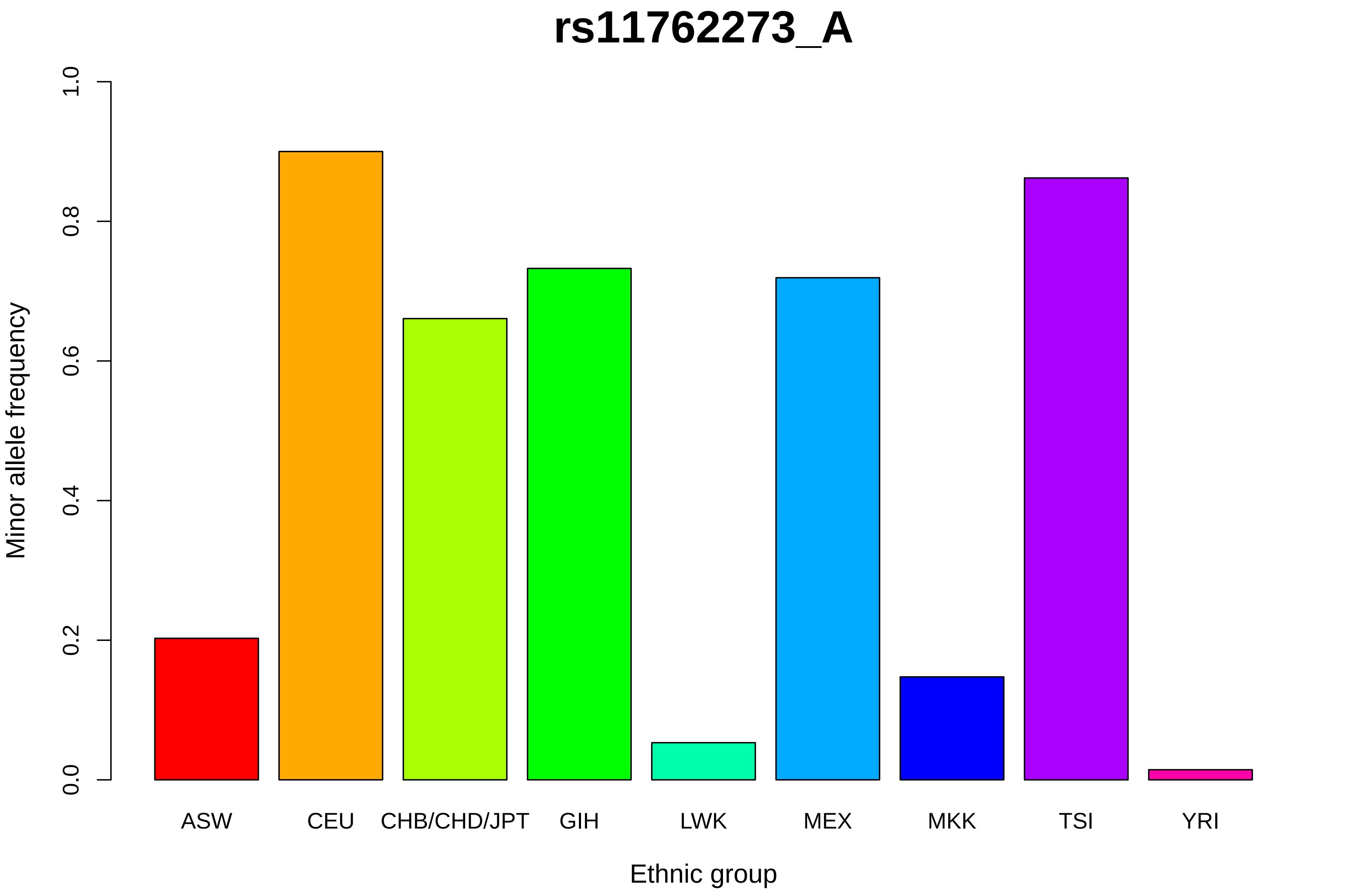
rs11762273_A
Minor allele frequency
0.0
0.2
0.4
0.6
0.8
1.0
ASW
CEU
CHB/CHD/JPT
GIH
LWK
MEX
MKK
TSI
YRI
Ethnic group

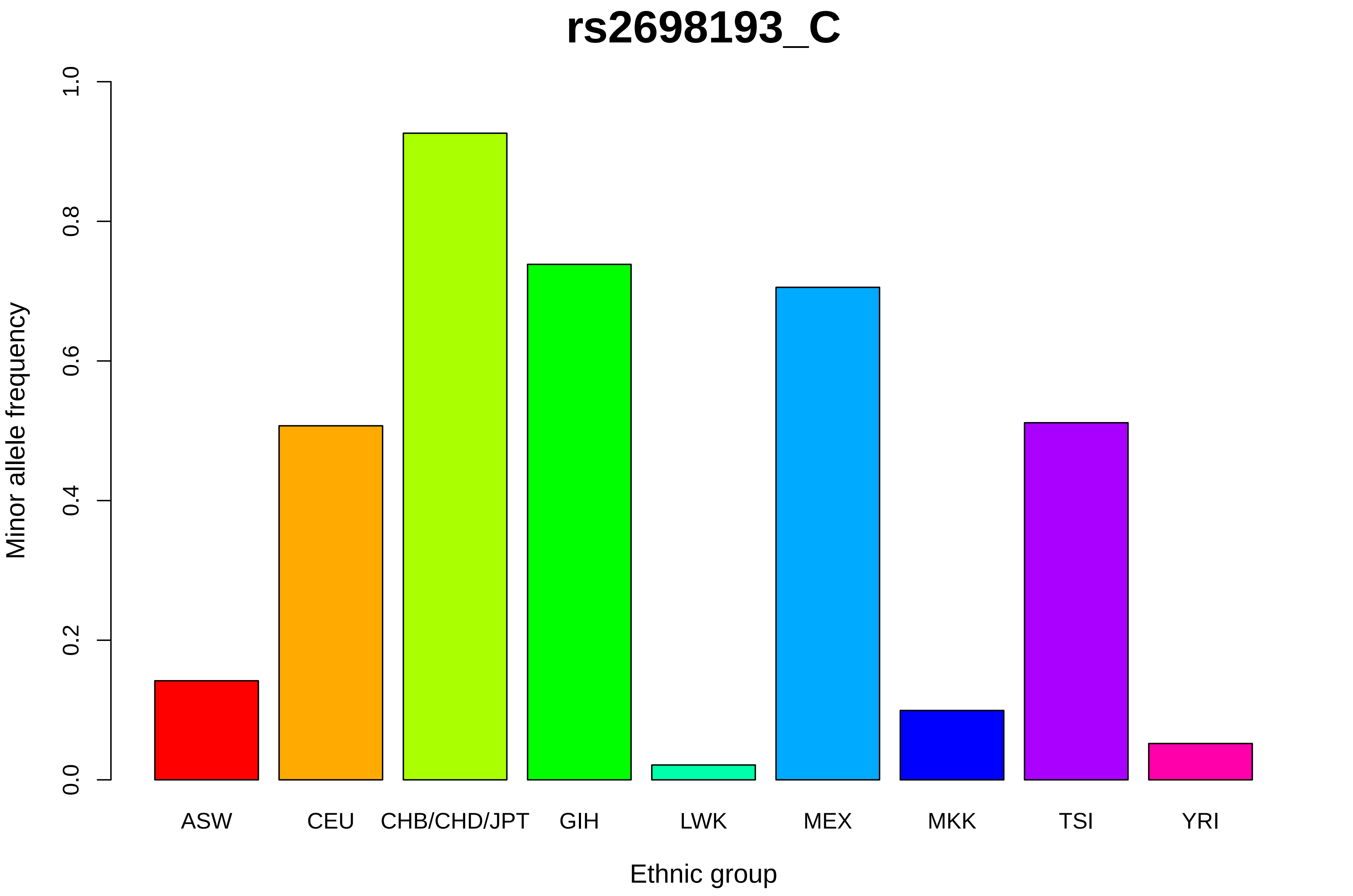
rs2698193_C
Minor allele frequency
0.0
0.2
0.4
0.6
0.8
1.0
ASW
CEU
CHB/CHD/JPT
GIH
LWK
MEX
MKK
TSI
YRI
Ethnic group

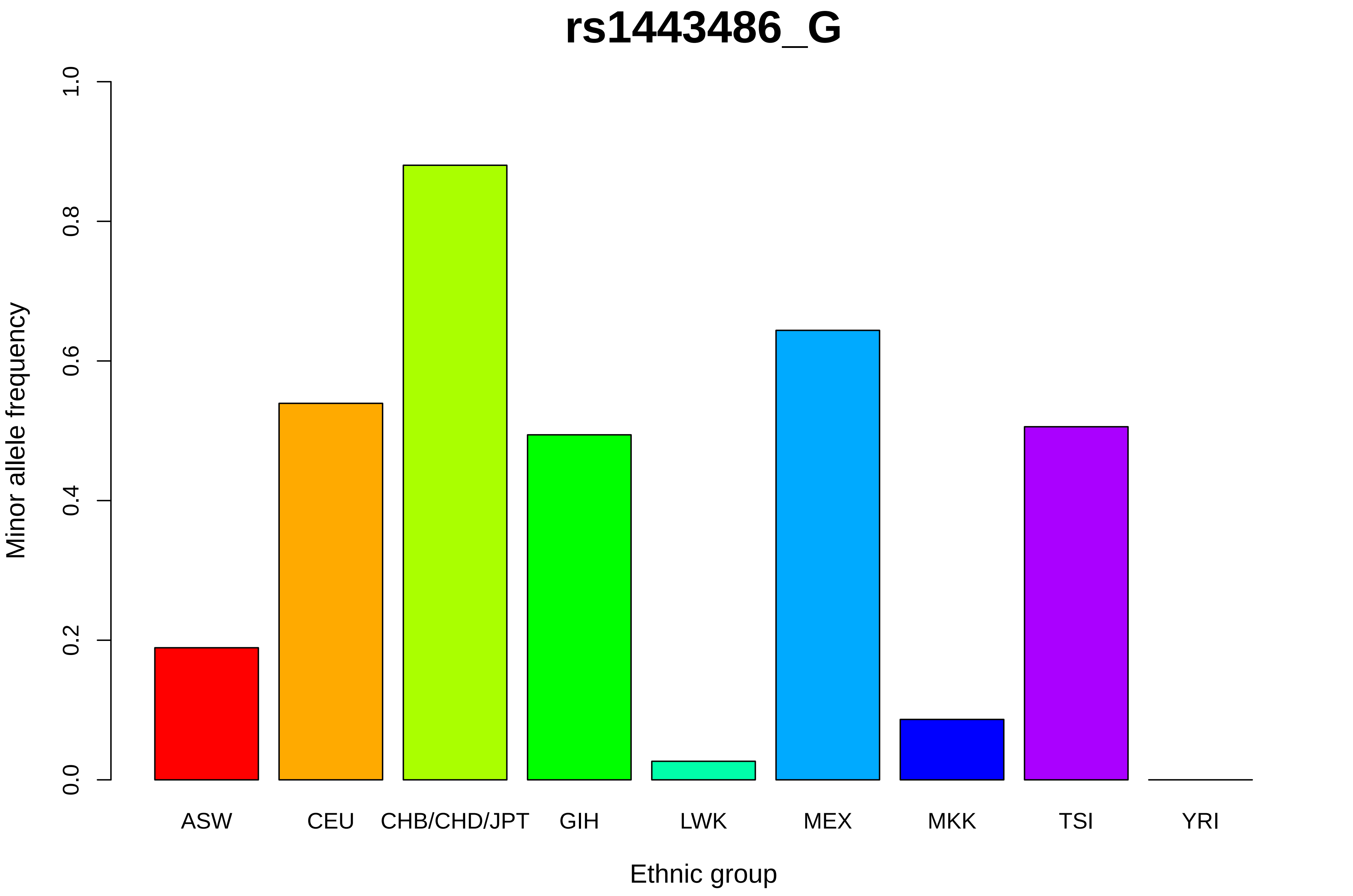
rs1443486_G
Minor allele frequency
0.0
0.2
0.4
0.6
0.8
1.0
ASW
CEU
CHB/CHD/JPT
GIH
LWK
MEX
MKK
TSI
YRI
Ethnic group

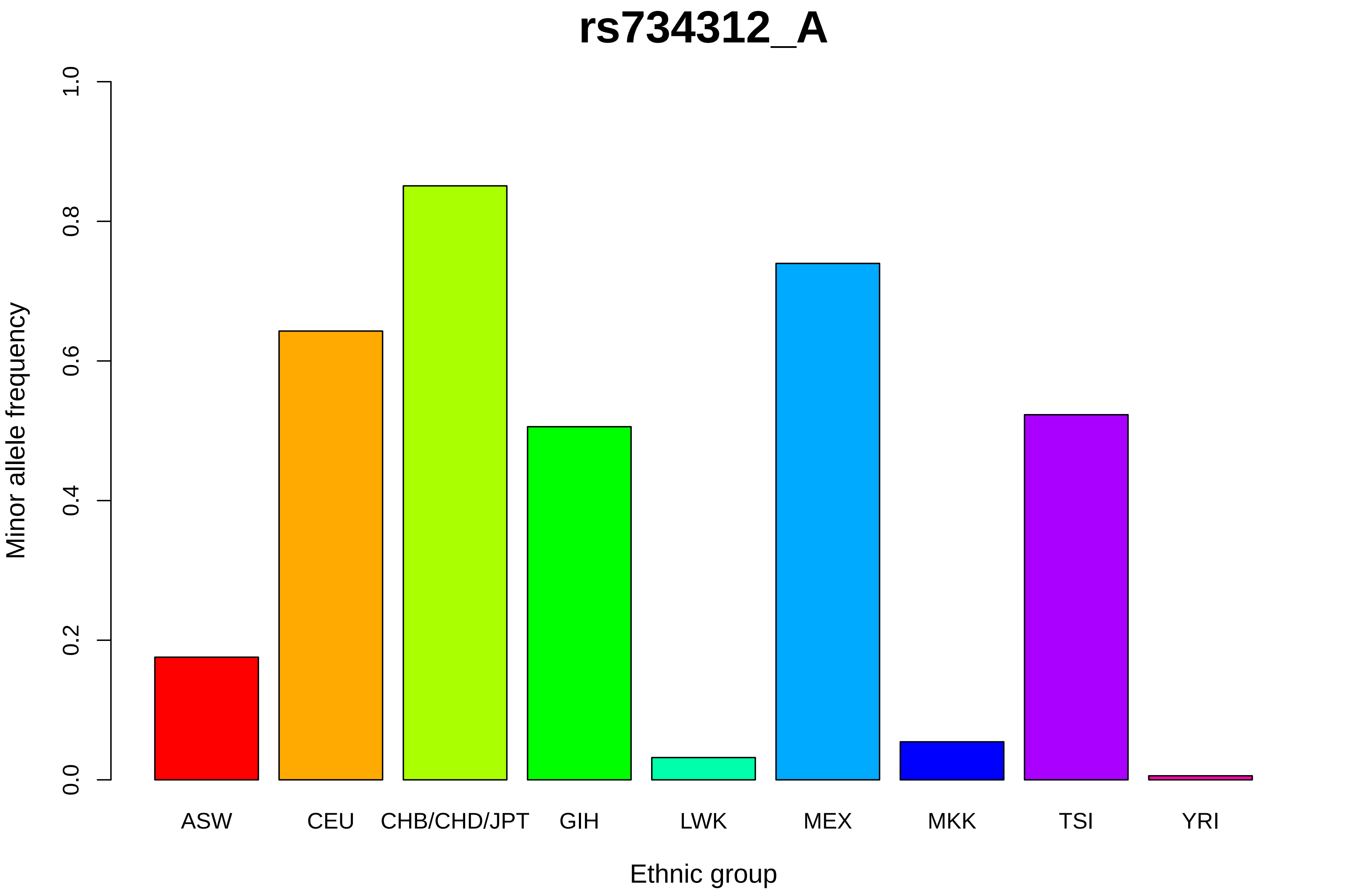
rs734312_A
Minor allele frequency
0.0
0.2
0.4
0.6
0.8
1.0
ASW
CEU
CHB/CHD/JPT
GIH
LWK
MEX
MKK
TSI
YRI
Ethnic group

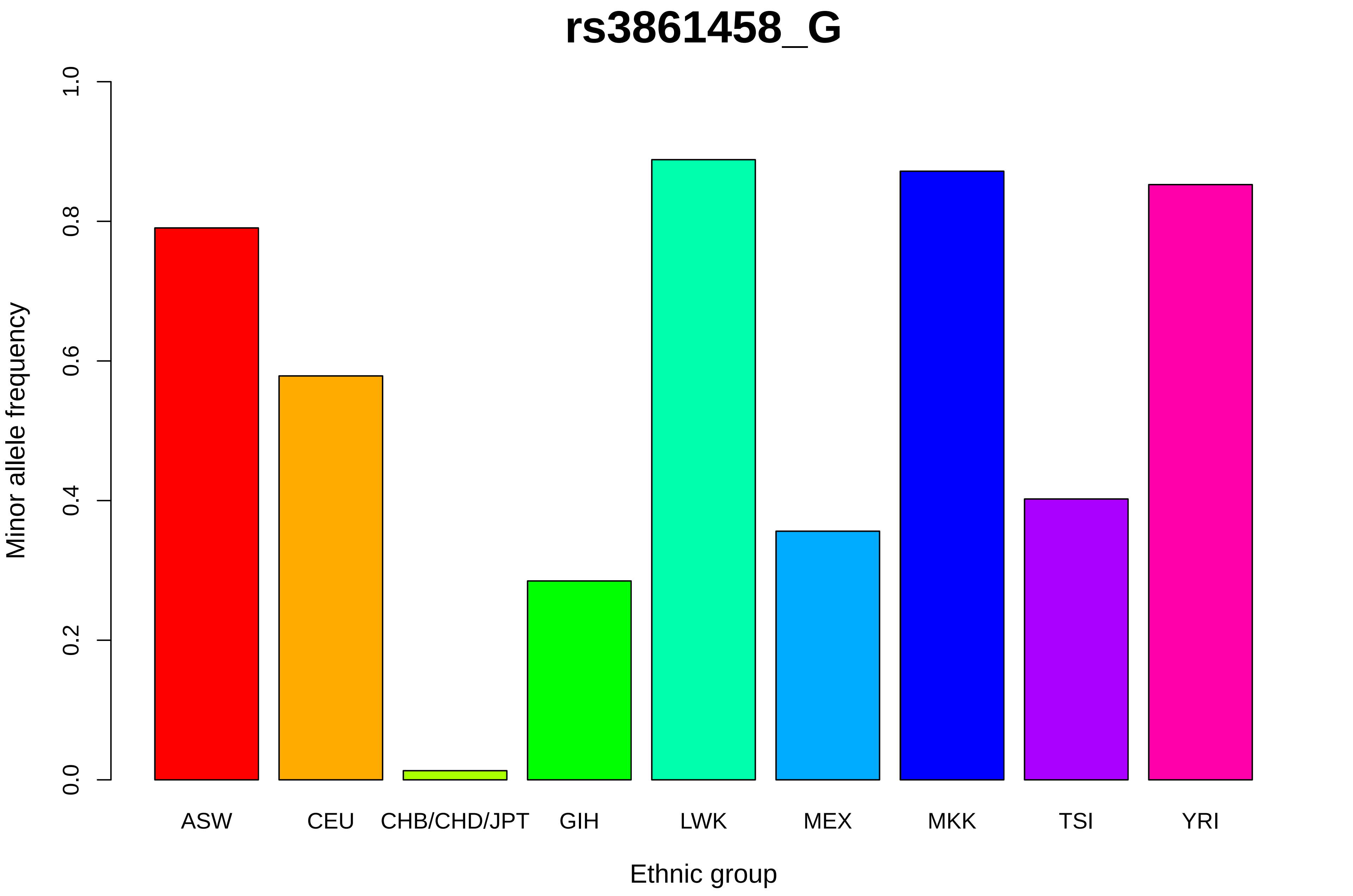
rs3861458_G
Minor allele frequency
0.0
0.2
0.4
0.6
0.8
1.0
ASW
CEU
CHB/CHD/JPT
GIH
LWK
MEX
MKK
TSI
YRI
Ethnic group

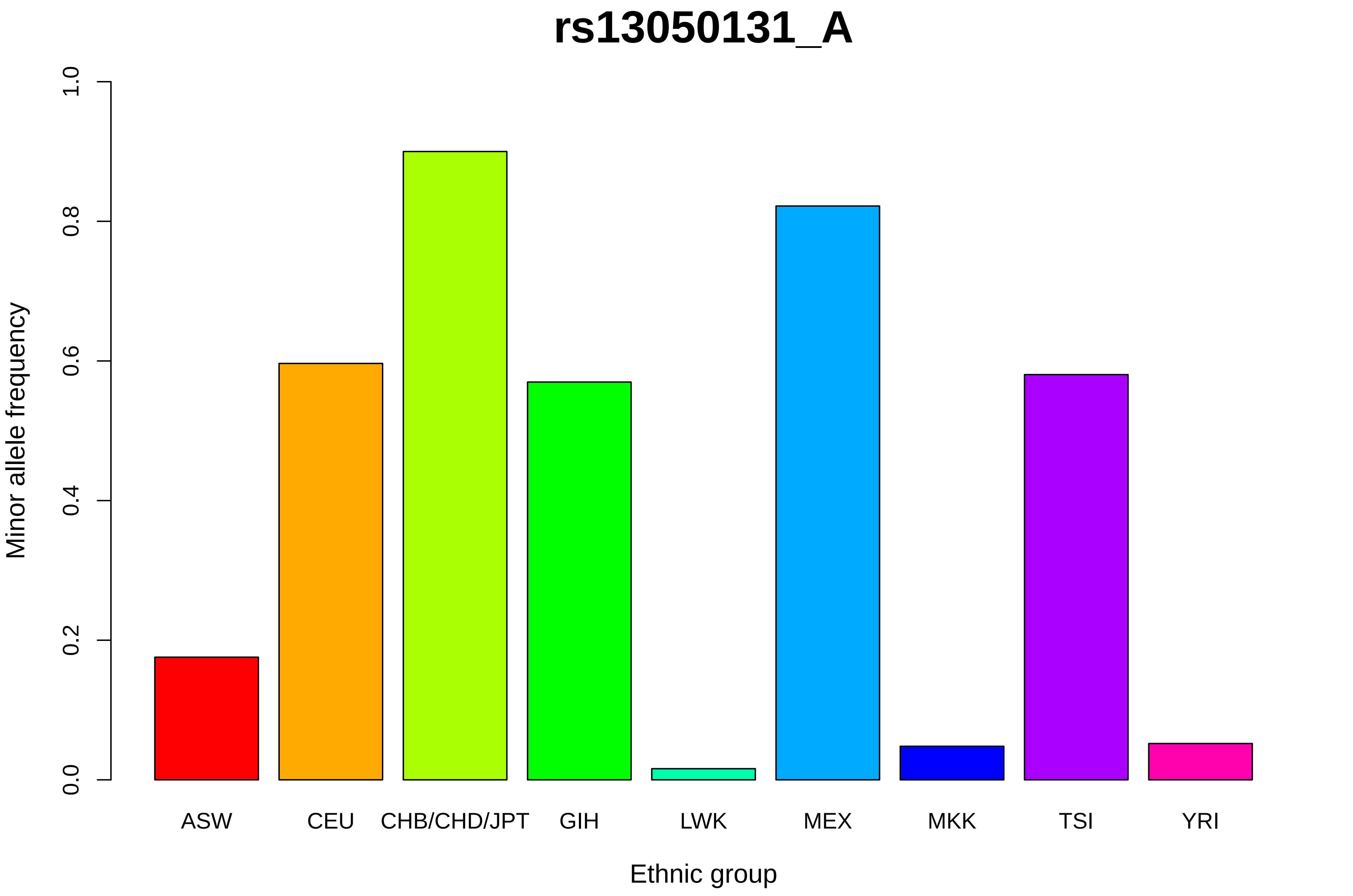
rs13050131_A
Minor allele frequency
0.0
0.2
0.4
0.6
0.8
1.0
ASW
CEU
CHB/CHD/JPT
GIH
LWK
MEX
MKK
TSI
YRI
Ethnic group

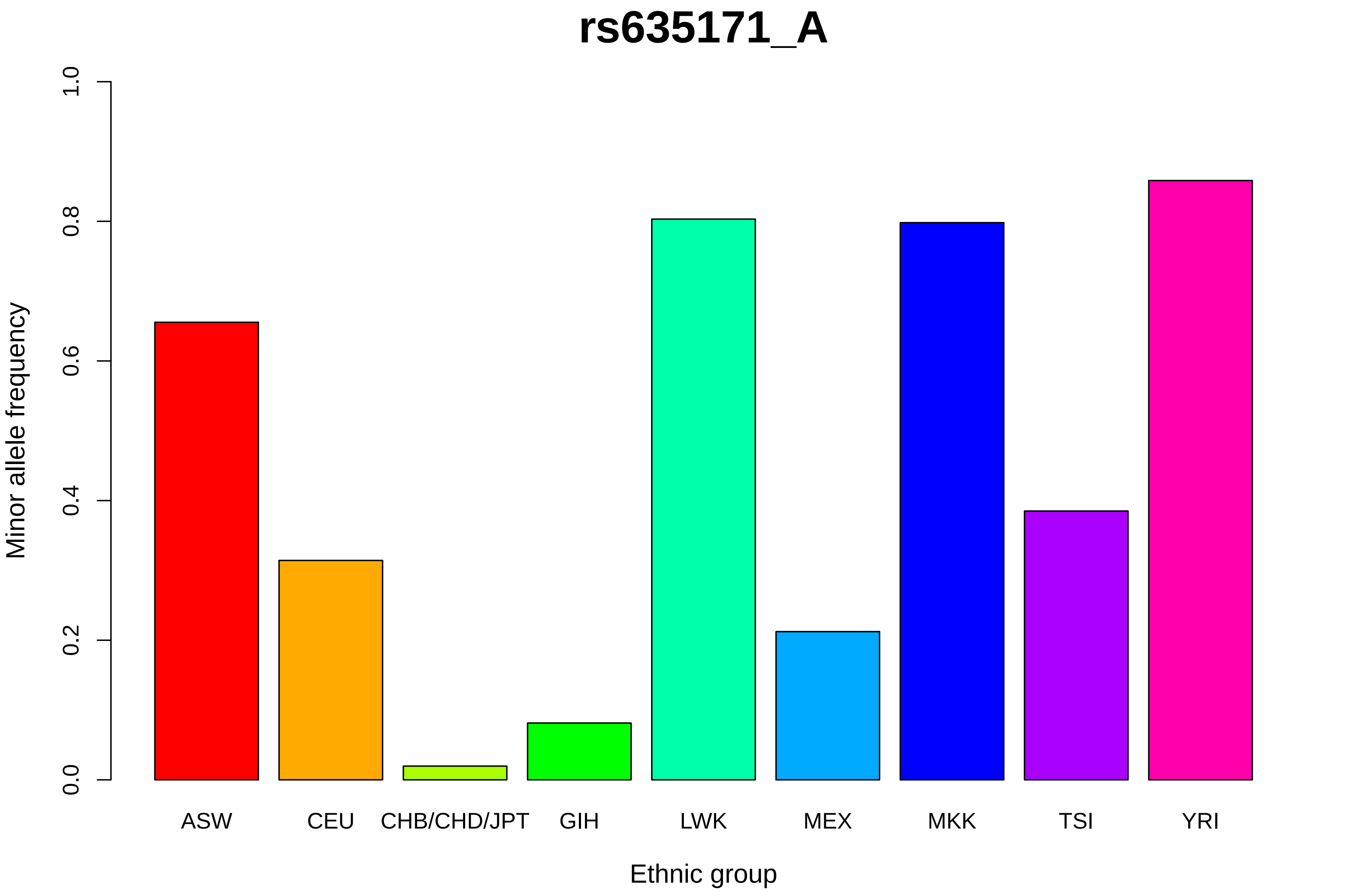
rs635171_A
Minor allele frequency
0.0
0.2
0.4
0.6
0.8
1.0
ASW
CEU
CHB/CHD/JPT
GIH
LWK
MEX
MKK
TSI
YRI
Ethnic group

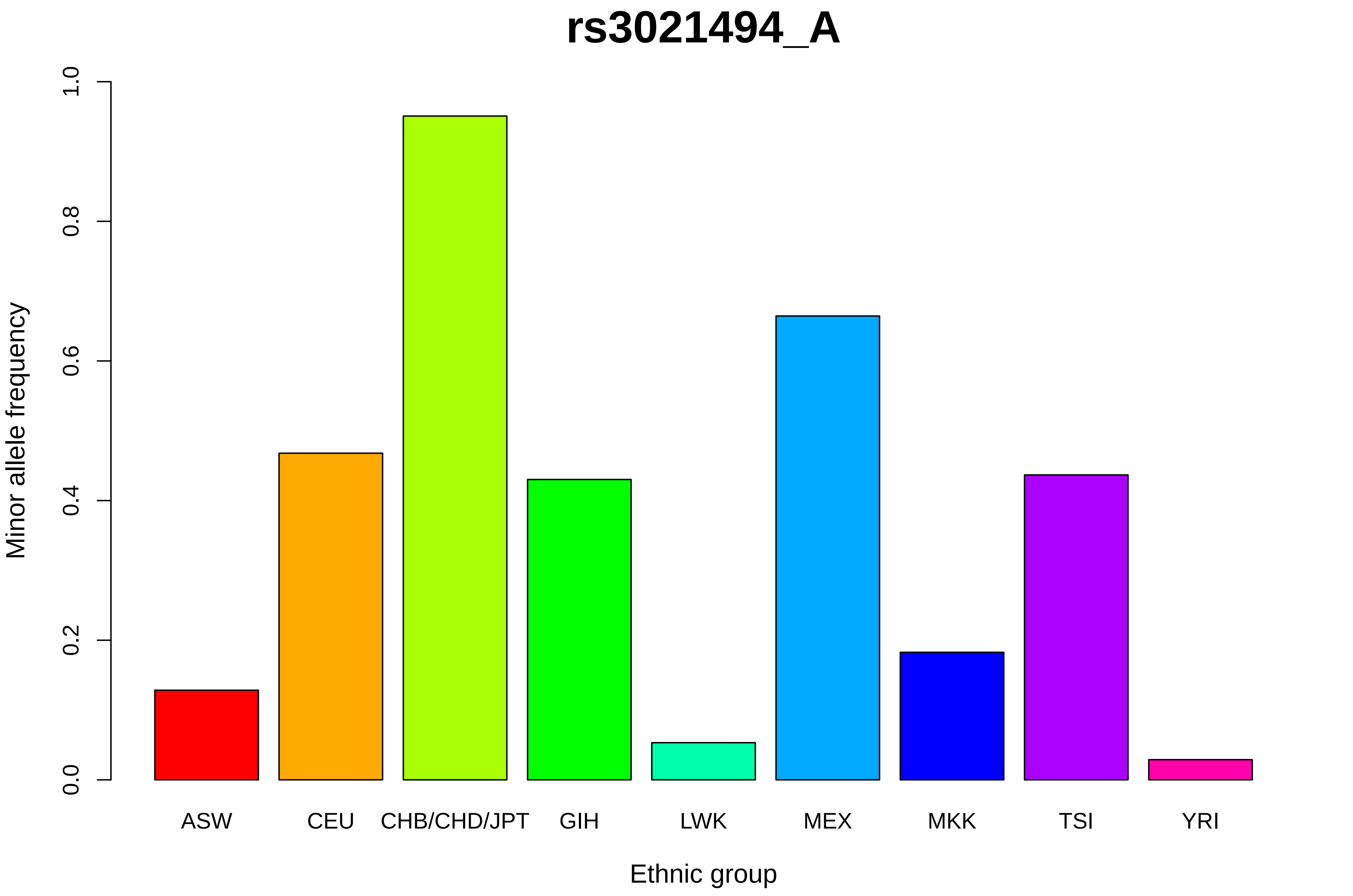
rs3021494_A
Minor allele frequency
0.0
0.2
0.4
0.6
0.8
1.0
ASW
CEU
CHB/CHD/JPT
GIH
LWK
MEX
MKK
TSI
YRI
Ethnic group

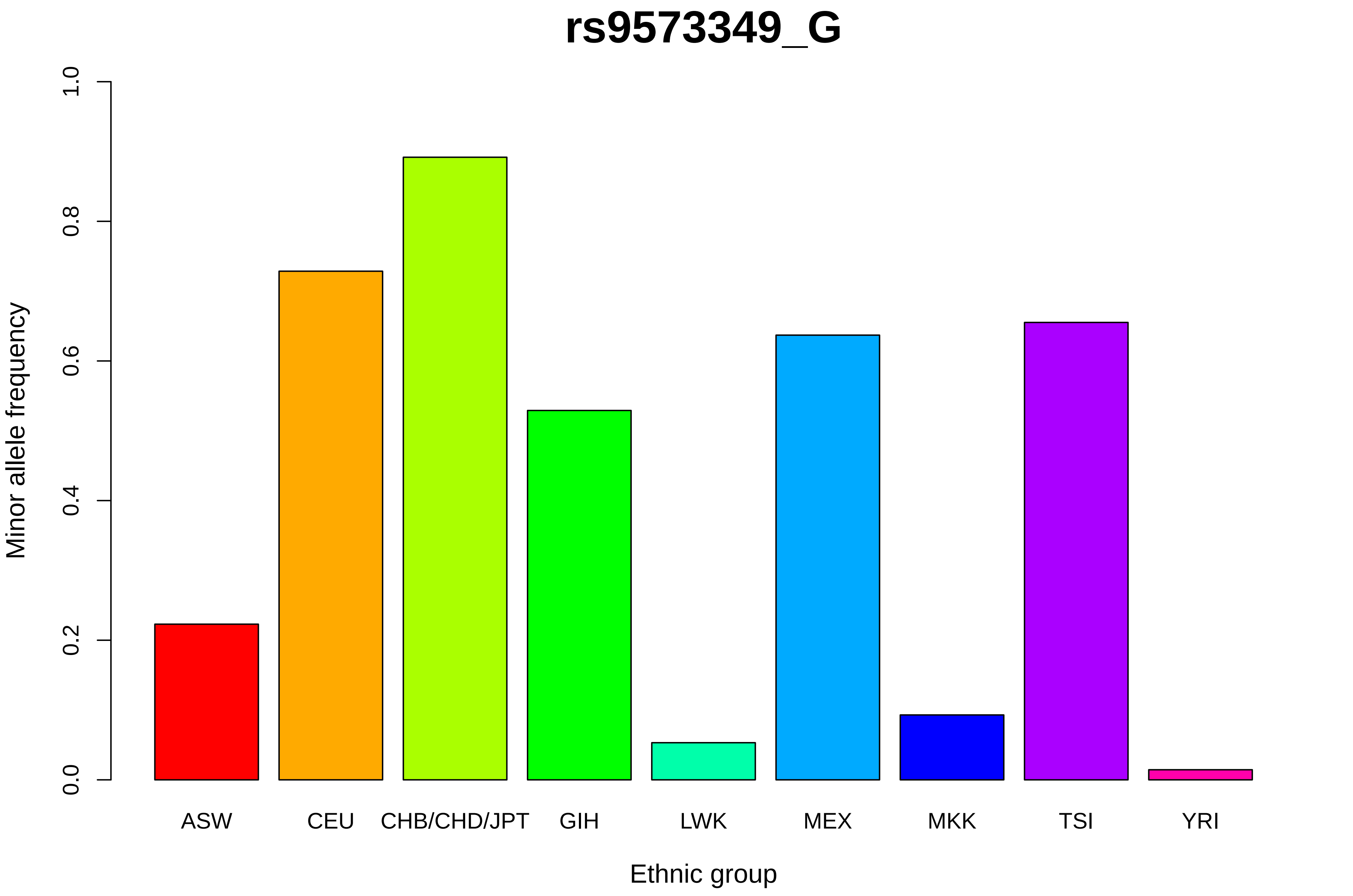
rs9573349_G
Minor allele frequency
0.0
0.2
0.4
0.6
0.8
1.0
ASW
CEU
CHB/CHD/JPT
GIH
LWK
MEX
MKK
TSI
YRI
Ethnic group

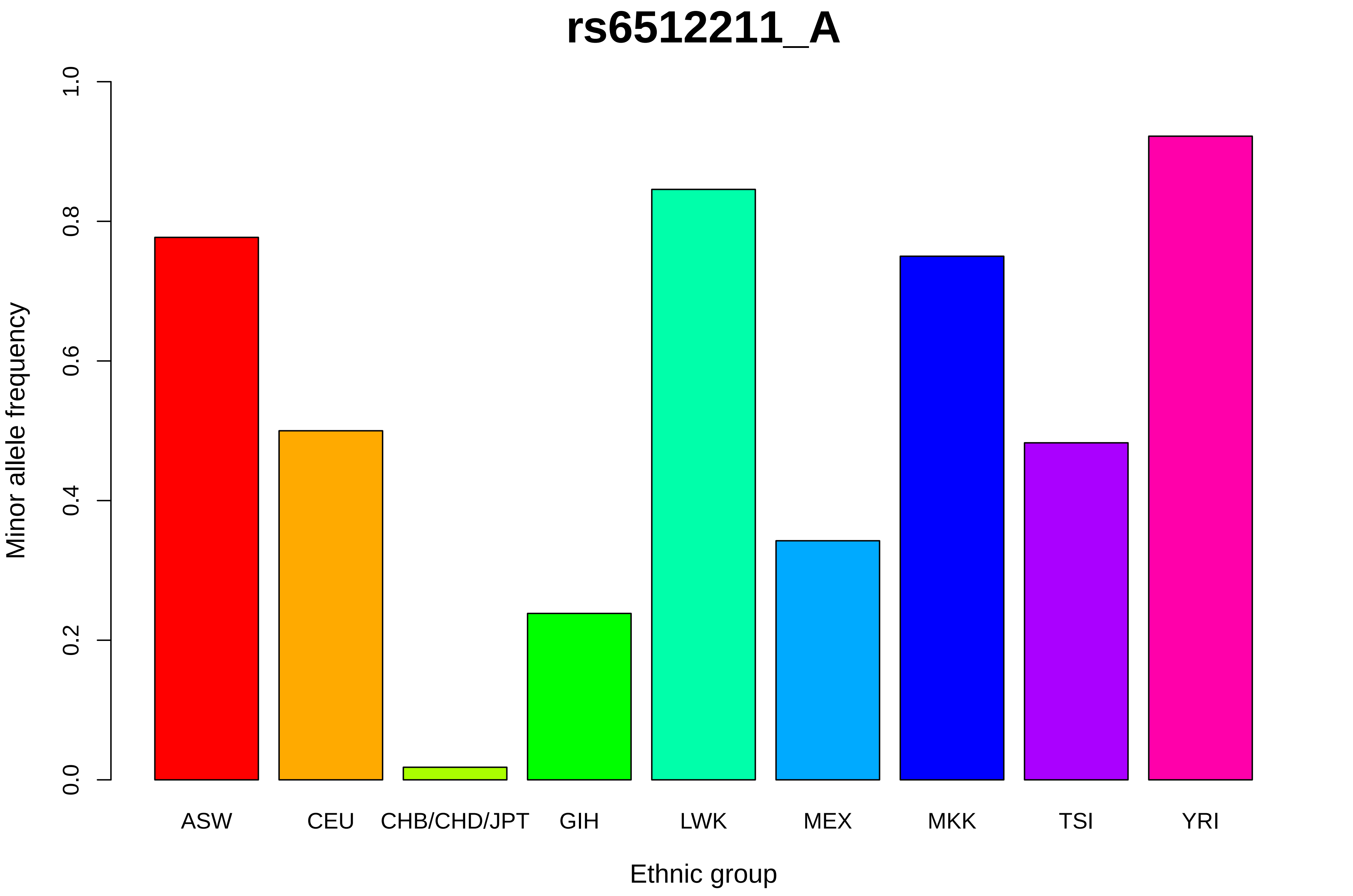
rs6512211_A
Minor allele frequency
0.0
0.2
0.4
0.6
0.8
1.0
ASW
CEU
CHB/CHD/JPT
GIH
LWK
MEX
MKK
TSI
YRI
Ethnic group

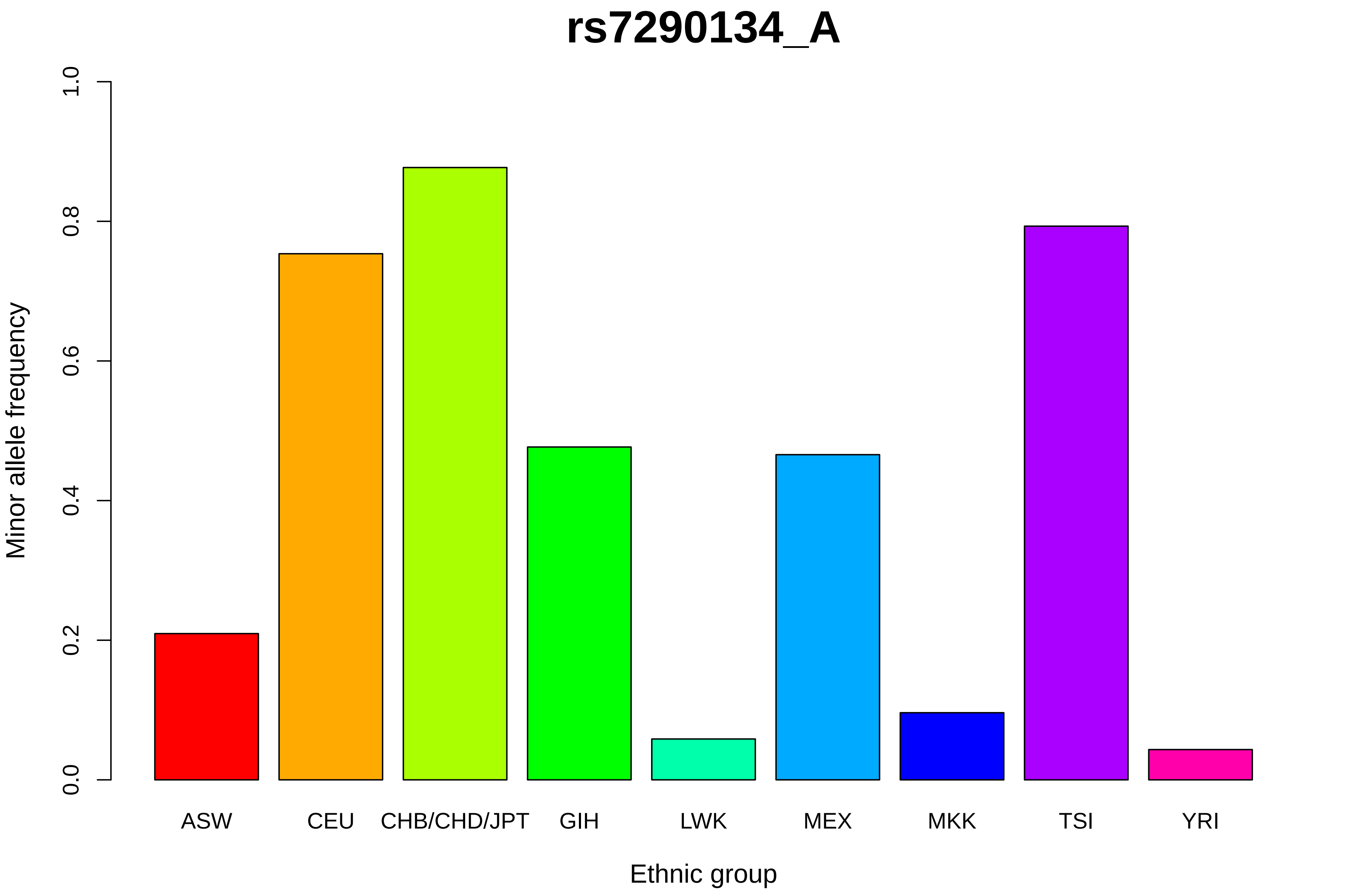
rs7290134_A
Minor allele frequency
0.0
0.2
0.4
0.6
0.8
1.0
ASW
CEU
CHB/CHD/JPT
GIH
LWK
MEX
MKK
TSI
YRI
Ethnic group
